# Food Compass 2.0 is an improved nutrient profiling system to characterize healthfulness of foods and beverages

**DOI:** 10.1038/s43016-024-01053-3

**Published:** 2024-10-08

**Authors:** Eden M. Barrett, Peilin Shi, Jeffrey B. Blumberg, Meghan O’Hearn, Renata Micha, Dariush Mozaffarian

**Affiliations:** 1 Food is Medicine Institute, Friedman School of Nutrition Science & Policy, Tufts University, Boston, MA USA; 2 The George Institute for Global Health, Faculty of Medicine and Health, University of New South Wales, Sydney, New South Wales Australia; 3 Food Systems for the Future, Chicago, IL USA; 4 Department of Food Science and Nutrition, University of Thessaly, Volos, Greece; 5 Tufts University School of Medicine and Tufts Medical Center, Boston, MA USA

**Keywords:** Nutrition, Health policy, Epidemiology, Risk factors

## Abstract

Food Compass is a nutrient profiling system used to assess the healthfulness of diverse foods, beverages and meals. Here we present a revised version of Food Compass (Food Compass 2.0) incorporating new data on specific ingredients and the latest diet–health evidence. Food Compass 2.0 has been validated against health outcomes in a population from the United States and demonstrates enhanced ability to characterize foods and beverages based on their healthfulness.

## Abstract (web summary)

Nutrition profiling systems inform nutritional and health policies to prevent disease and promote health. This study presents a revised version of the Food Compass nutrient profiling system that incorporates the latest evidence linking diet and health—and which has been validated against health outcomes in a US population.

## Main

The escalating health burdens of diet-related non-communicable diseases require population-level strategies. Nutrient profiling systems (NPSs), quantitative algorithms that evaluate and rank healthfulness of foods and beverages, are increasingly common tools for governments and industry to make decisions around promoting healthier eating, including for front-of-pack and menu labelling, thresholds to restrict food marketing, eligibility for health claims, food procurement policies, portfolio reformulation targets, and health-conscious investment strategies^1–3^.

Many existing NPSs have important limitations, including a focus on mostly negative nutrients, lack of assessment of many food ingredients or emerging nutrients of relevance, omission of processing characteristics, inconsistent scoring across food categories and for mixed products and meals, and scoring per food weight (confounded by water content). To address these challenges, the Food Compass was developed in 2021 as an NPS that captures nine holistic domains of product characteristics, including components such as nutrient ratios as indicators of fat, carbohydrate and mineral quality; food ingredients of greatest health relevance; and food processing, phytonutrient and additive characteristics—all per 100 kcal (ref. ^4^). Previous investigations demonstrated that the Food Compass facilitates a more balanced and universal assessment of foods and beverages with uniform scoring criteria to minimize subjectivity, enhance consistency, and score mixed foods and meals; has strong validity against other common NPSs; associates with improved health risk factors and prevalent disease conditions; and independently predicts total mortality when applied to diets of individuals^4,5^.

At the same time, as a highly promising NPS, Food Compass was intended to be reviewed and improved based on emerging evidence, availability of new data and scientific feedback from the community. In this report, we present our methods, results and validation to develop Food Compass 2.0 as an improved NPS.

**Fig. 1:**
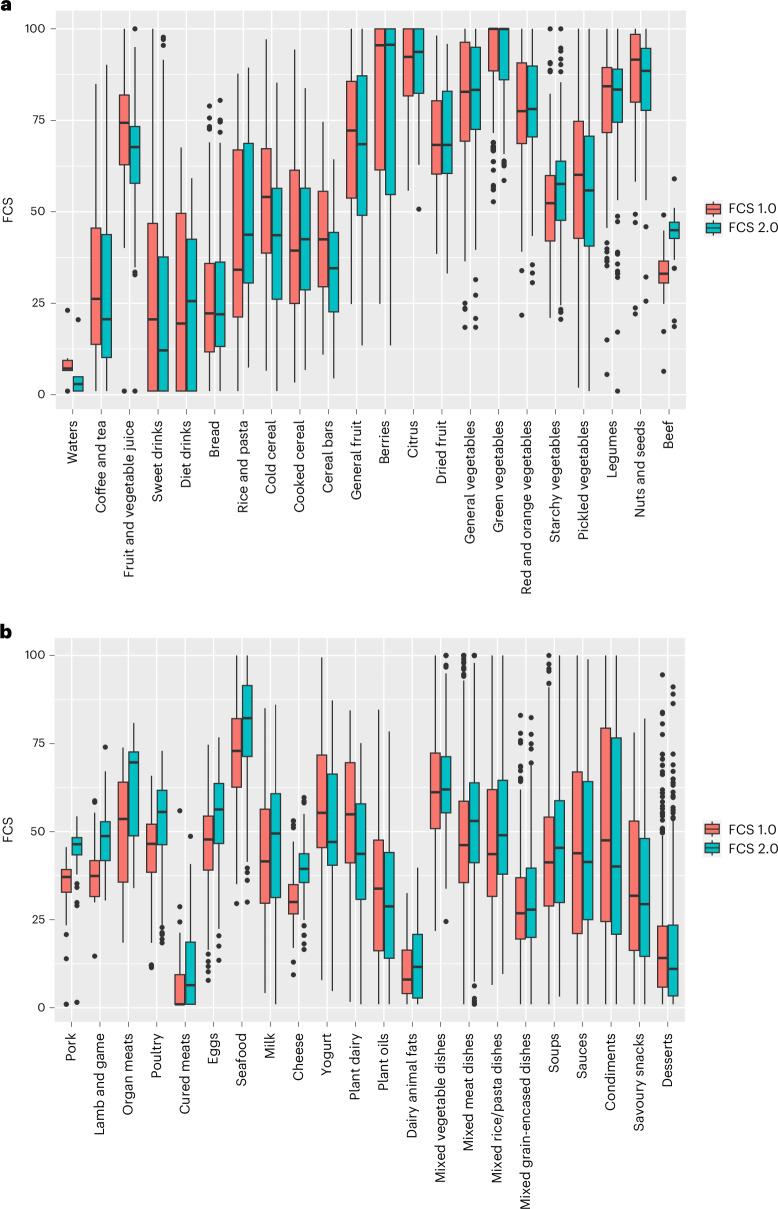
Updated and original FCSs for products consumed by US adults. **a**,**b**, Data are from 9,273 products reported within NHANES 2001/02–2017/18 for subcategories 1–22 (**a**) and 23–44 (**b**). Standard box plots are shown, with horizontal lines representing the median value, bounds of boxes representing the 25th (lower bound) and 75th (upper bound) percentile values, whiskers representing 1.5 × interquartile range from the 25th percentile (for the lower whisker) and the 75th percentile (for the upper whisker), and the black dots beyond these bounds representing outliers.

Details of the specific updates as part of Food Compass 2.0 are provided in Supplementary Table 1. A comparison of the original Food Compass versus Food Compass 2.0 across the 9,273 unique food and beverage items showed similar mean Food Compass scores (FCSs) for some major food groups (for example, nuts, legumes, sauces/condiments), but meaningful shifts in others (Supplementary Fig. 1 and Supplementary Table 2). Among food subgroups, notable FCS declines (mean ± SD) included cold cereals (from 51 ± 21 to 41 ± 20), plant-based dairy (54 ± 21 to 43 ± 20), cereal bars (42 ± 16 to 34 ± 15), and fruit and vegetable juices (from 72 ± 15 to 66 ± 14); while increases included beef (33 ± 6 to 44 ± 6), pork (35 ± 8 to 44 ± 9), seafood (72 ± 14 to 81 ± 14), lamb and game (39 ± 8 to 49 ± 8), eggs (46 ± 13 to 54 ± 13), and rice and pasta (43 ± 26 to 49 ± 23) (Fig. 1 and Supplementary Table 3). Within these subcategories, changes at the individual food level varied. For example, within eggs, the FCS of a whole egg fried without fat increased from 48 to 62, whereas egg substitute decreased in score from 50 to 45 (other examples in Supplementary Table 4). The original Food Compass versus Food Compass 2.0 scores for all 9,273 foods and beverages are provided in Supplementary Table 5.

**Fig. 2:**
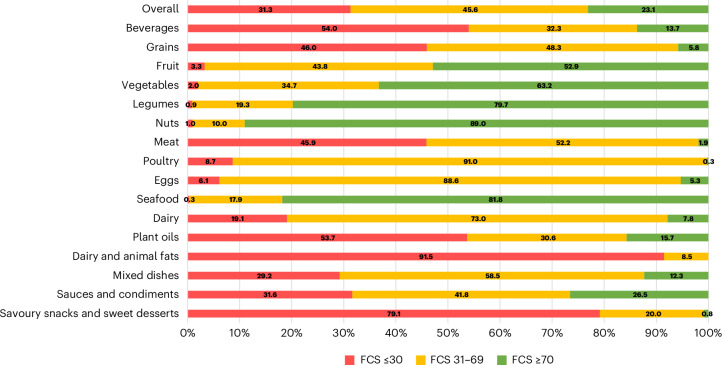
Percentages of FCSs ≤30, 31–69 and ≥70. Foods and beverages scoring ≤30 are those to be minimized, foods and beverages scoring 31–69 are those to be consumed in moderation, and foods and beverages scoring ≥70 are those to be encouraged. The major food groups meat, poultry, eggs, fats and oils, and legumes and nuts have been split to show detail. Meat includes beef, pork, lamb and game, organ meats, and cured meat.

Among all products, 23% scored FCS ≥70 (previously 22%); 46%, FCS 31–69 (previously 46%); and 31%, FCS ≤30 (previously 33%); but with meaningful variation by food category (Fig. 2). For example, most beverages (54%) and animal fats (92%) scored ≤30; whereas most meat, poultry, eggs and dairy scored 31–69 (52%, 91%, 89% and 73%, respectively). Most products within seafood, legumes, nuts, vegetables and fruits scored ≥70 (82%, 80%, 89%, 63% and 53%, respectively; with lower-scoring items often including high added sugars or other additives).

Compared to other common NPSs such as Health Star Rating (HSR), Nutri-Score or NOVA processing classification, Food Compass 2.0 demonstrated meaningful overlap but also further important differentiation (Supplementary Fig. 2 and Supplementary Table 5). For example, among products with the highest HSR (5.0), 82% had FCS ≥70, but product scores ranged from 100 for chia seeds to 60 for low-fat cottage cheese to 10 for fat-free margarine. Among products with the lowest HSR (0.5), 86% had FCS ≤30, but product scores included 84 for dried shrimp, 26 for cooked pork bacon and 1 for chocolate-covered caramel candy. Similarly, within NOVA group 1, 49% of products had FCS ≥70, but scores ranged from 100 for raw blackberries to 59 for rotisserie chicken breast with skin to 12 for rice noodles. Within NOVA group 4, 68% of items had FCS ≤30, but scores ranged from 87 for low-fat fruit yogurt to 53 for flavoured instant oatmeal to 1 for cola-flavoured soft drink. Compared to the original Food Compass, Food Compass 2.0 still exhibited substantial discrimination against HSR, Nutri-Score and NOVA (Supplementary Table 6). Intercorrelations became modestly more concordant between Food Compass 2.0 and NOVA for all categories, such as for grains (previously *r* = 0.07, now *r* = 0.31) and dairy (from 0.31 to 0.58), and between FCS 2.0 and HSR for grains (from 0.27 to 0.42) and legumes, nuts and seeds (from 0.46 to 0.55); and modestly less concordant for fats and oils (from 0.47 to 0.36). Given the high correlation between Nutri-Score and HSR (*r* = 0.83), the strength and direction of changes in concordance of Food Compass against Nutri-Score were similar to those for HSR.

Food Compass 2.0 also performed well when the scores of individual food products were extended to score the daily diets of individuals and then validated against health outcomes. In a nationally representative population of 47,099 US adults, the energy-weighted average FCS of foods and beverages consumed was calculated for each person (referred to as i.FCS). The mean score was 36.6 ± 10.8, consistent with a relatively poor average diet. The i.FCS correlated highly with HEI-2015 (*r* = 0.78), a validated measure of a healthy dietary pattern. After multivariable adjustment, each 1 s.d. (10.8 points) higher i.FCS was associated with more favourable body mass index (−0.56 kg m^−2^ (95% confidence interval (C1) −0.65, −0.47)), systolic blood pressure (−0.55 mm Hg (95% CI −0.77, −0.34)), diastolic blood pressure (−0.46 mm Hg (95% CI −0.63, −0.29)), low-density lipoprotein cholesterol (−1.49 mg dl^−1^ (95% CI −2.10, −0.87)), high-density lipoprotein cholesterol (1.61 mg dl^−1^ (95% CI 1.41, 1.81)), total cholesterol to high-density lipoprotein ratio (−0.12 (95% CI −0.13, −0.10)), haemoglobin A1c (−0.02% (95% CI −0.02, −0.01)) and fasting plasma glucose (−0.36 mg dl^−1^ (95% CI −0.67, −0.05)); and with a lower prevalence of metabolic syndrome (odds ratio (OR), 0.86; 95% CI, 0.83, 0.89), cardiovascular disease (OR, 0.92; 95% CI, 0.88, 0.96), cancer (OR, 0.93; 95% CI, 0.89, 0.98) and lung disease (OR, 0.90; 95% CI, 0.87, 0.94); and higher prevalence of optimal cardiometabolic health (OR, 1.22; 95% CI, 1.14, 1.30) (Supplementary Table 7). The updated i.FCS also associated with lower all-cause mortality (per 1 s.d., hazard ratio, 0.92; 95% CI, 0.88, 0.95) and with a 24% lower risk in the highest i.FCS quintile versus the lowest (hazard ratio, 0.76; 95% CI, 0.68, 0.84) (Supplementary Fig. 3 and Supplementary Table 8).

These findings present an updated, validated Food Compass NPS. Maintaining the strengths of the core principles and framework, these modifications better reflect the scientific evidence on processing, added sugar, dietary fibre, dairy fat, artificial additives and trace lipids. One impactful update was providing positive points for non-ultraprocessed foods, rather than only negative points for ultraprocessed foods. Accumulating scientific evidence demonstrates the health benefits of minimally processed foods^6,7^, and Food Compass 2.0 therefore better distinguishes products across the range of food processing for both animal- and plant-source foods. Similarly, Food Compass 2.0 better accounts for research suggesting the relatively neutral health effects of dairy fat^8,9^; and for harms of added sugar as not just an additive but also a food ingredient^10^. Notably, although artificial additives were attributes in the original Food Compass, data for their scoring were not previously available, which has now been rectified. Consequently, scores of highly processed foods with multiple artificial additives have decreased. These changes provide FCS values that more appropriately reflect dietary guidelines for their consumption.

Although individual items in any category had specific changes in their score, in general, Food Compass 2.0 provides higher scores for minimally processed animal foods such as seafood, dairy, meat, poultry and eggs; and lower scores for processed cereals, beverages, flavoured yogurts, and processed plant-based egg, meat and dairy alternatives. At the same time, few products (∼10%) had a score change exceeding 10 points, highlighting the relative stability of the Food Compass framework and its principles. Food Compass 2.0 also provides important differentiation within categories of processing; for example, blueberries (FCS 100) and white rice (FCS 23) are both NOVA type 1, but their substantially different nutritional effects are appropriately reflected in their FCS. Although scores of starch-rich refined staples such as white rice or bread are low when consumed alone, Food Compass 2.0 allows scoring of mixed meals and provides higher scores to recipes that integrate starchy staples with other healthful ingredients. This supports alignment of Food Compass with both modern nutrition science and with diverse food cultures that value traditional dietary patterns. In conclusion, the updated Food Compass demonstrates improved ability to characterize foods and beverages based on healthfulness, with continued discrimination against existing NPSs, and demonstrated validity against healthful dietary patterns and health outcomes. We encourage researchers, policymakers, retailers, manufacturers and all stakeholders interested in identifying and encouraging healthier food and beverages to use Food Compass 2.0 (the full algorithm for its calculation is presented in the Supplementary Information). Given the foundational principles of Food Compass and the global reach of many packaged foods, we expect Food Compass 2.0 to have validity in many contexts, as has been shown already in Greece^11^, Korea^12^ and China^13^. We are also engaging in collaborative efforts to adapt Food Compass for use in different world regions, which could include adapting certain scoring parameters to align with regional differences in food supply. We hope that the holistic nature and demonstrated validity of Food Compass encourage food manufacturers to measure and report more of the important attributes in the scoring. At the same time, we are also developing and evaluating adaptations of Food Compass to be scored from a smaller set of more commonly available nutrient and ingredient information, leveraging imputation and estimation techniques, to facilitate scoring of all products in the marketplace with currently available data.

## Methods

Because this study used deidentified, publicly available data from the National Health and Nutrition Examination Survey (NHANES), institutional review board approval was not required. The NHANES protocol was approved by the institutional review board at the National Center for Health Statistics, with all participants providing informed written consent. Participants were also compensated and received a report of their medical findings.

Detailed methods are presented in Supplementary Methods 1 and 2. Briefly, we compared the original and updated FCS for 9,273 unique foods and beverages in a nationally representative dataset, assessing face validity, convergent and discriminant validity, and criterion validity including associations with health outcomes. Key updates include (1) broader discrimination in scoring of food processing; (2) inclusion of added sugar, a major potential energy source, in the food ingredients domain; (3) higher scoring weight for dietary fibre as a positive attribute; (4) lower scoring weight for dairy fat as a negative attribute; and (5) new data collection on additives (for example, artificial sweeteners) which were in the original algorithm but previously unscored due to insufficient data. Other updates included neutral scoring for fruit and vegetable juice as food ingredients; and greater scoring weight to long-chain omega-3 fatty acids as compared to other lipids. Details of these revisions and their rationales are provided in Supplementary Table 1. Details of the scoring process for each attribute, domain and the overall FCS are provided in Supplementary Tables 9 and 10. By design, FCSs are scaled to range from 1 (least healthy) to 100 (most healthy). Although scores can be considered continuously, general recommendations have also been proposed at FCS ≥70 (foods to be encouraged), FCS 31–69 (foods to be consumed in moderation) and FCS ≤30 (foods to be minimized)^4,5^, which may be useful when strict cut-offs for healthy and unhealthy products are required.

### Reporting summary

Further information on research design is available in the Nature Portfolio Reporting Summary linked to this article.

## Supplementary information

Supplementary Information Supplementary Tables 1–10, Supplementary Figs. 1–3 and Supplementary Methods 1 and 2.

Reporting Summary

## Data Availability

The attribute- and domain-scoring algorithm used to generate Food Compass is available in Supplementary Tables 9 and 10. All data used in this analysis are publicly available from the following US Department of Agriculture (USDA) and Centers for Disease Control sources: (1) nutrient composition data for foods reported in NHANES dietary recalls (USDA Food and Nutrient Database for Dietary Studies 2001–2018, https://www.ars.usda.gov/northeast-area/beltsville-md-bhnrc/beltsville-human-nutrition-research-center/food-surveys-research-group/docs/fndds-download-databases/); (2) food ingredients data for foods reported in NHANES dietary recalls (USDA Food Pattern Equivalents Database, 2001–2018, https://www.ars.usda.gov/northeast-area/beltsville-md-bhnrc/beltsville-human-nutrition-research-center/food-surveys-research-group/docs/fped-overview/); (3) flavonoid data for select foods reported in NHANES dietary recalls (USDA Flavonoid Database, 2007–2010, ars.usda.gov/northeast-area/beltsville-md-bhnrc/beltsville-human-nutrition-research-center/food-surveys-research-group/docs/fndds-flavonoid-database/); (4) national dietary recall, sociodemographic, physical activity, smoking, cardiometabolic biomarker and prevalent condition data for US adults (NHANES 1999–2018, wwwn.cdc.gov/nchs/nhanes/Default.aspx); (5) all-cause and cause-specific mortality data for US adults (National Death Index 1999–2018, https://www.cdc.gov/nchs/data-linkage/mortality-public.htm). The Nutri-Score categorizations for each food and beverage were calculated using the 2023 updated algorithm^14^. The Health Star Rating values for each food and beverage were calculated using the publicly available online calculator and guidance: www.healthstarrating.gov.au/internet/healthstarrating/publishing.nsf/Content/excel-calculator, www.healthstarrating.gov.au/internet/healthstarrating/publishing.nsf/Content/guide-for-industry. The generated Food Compass, HSR, Nutri-Score and NOVA food-processing classification scores for each of the 9,273 food items in the dataset are available in Supplementary Table 5.
