# Supplementary material for: Food Compass 2.0 is an improved nutrient profiling system to characterize healthfulness of foods and beverages
Source: Nat Food. 2024 Oct 8;5(11):911–5. doi: 10.1038/s43016-024-01053-3 (PMC11578874; doi:10.1038/s43016-024-01053-3)
Supplement: Supplementary file 1 — Supplementary Tables 1–10, Supplementary Figs. 1–3 and Supplementary Methods 1 and 2. [file 43016_2024_1053_MOESM1_ESM.pdf]

nature food

Supplementary information https://doi.org/10.1038/s43016-024-01053-3

Food Compass 2.0 is an improved nutrient profiling system to characterize healthfulness of foods and beverages

In the format provided by the authors and unedited

Table S1. Updates to the Food Compass algorithm (Food Compass 2.0)

Domain affected	Updated approach (FCS 2.0)	Previous approach	Rationale
Nutrient Ratio	Dairy products (yogurt, cheeses, milk, and dairy fats) are given half weight for the scoring of the Unsaturated: saturated fat ratio attribute.	All products are given full weight for the scoring of the Unsaturated: saturated fat ratio attribute.	Lack of meaningful evidence in long-term epidemiologic cohorts or interventional studies for harms of dairy fat as opposed to other animal fats.
Food-based ingredients	Refined carbohydrate (encompassing refined grains *and* added sugars) is included as an attribute.	Refined grain is included as an attribute.	To achieve stronger penalising of foods and beverages high in added sugar.
Food-based ingredients	Fruit and vegetable juices are no longer scored as attributes.	Fruit and vegetable juices are included as attributes but are half-weighted.	Equivocal evidence for health associations of juice consumption compared to whole fruit and vegetables.
Additives	The low score target (i.e., maximum negative points scorable) for the added sugars attribute is assigned to products with ≥60% of kilocalories from added sugars.	The low score target (i.e., maximum negative points scorable) for the added sugars attribute is assigned to products with 100% of kilocalories from added sugars.	To achieve stronger penalising of foods and beverages high in added sugar.
Additives	For products where ingredient data were available, artificial sweeteners/flavors/colors, partially hydrogenated oils, interesterified or hydrogenated oils, high fructose corn syrup and monosodium glutamate were scored as attributes. Each attribute was scored as “present” (-1) or “not present” (0) on the ingredient list.	Artificial sweeteners/flavors/colors, partially hydrogenated oils, interesterified or hydrogenated oils, high fructose corn syrup and monosodium glutamate were not scored within the Food Compass.	Improved data collection methods. We can now score these attributes for a subset of products within the Food and Nutrient Database for Dietary Studies (FNDDS) that include brand information. Using this information, we sourced ingredients lists for these products.
Processing	Points allocated to products within the NOVA attribute is as follows:<br>NOVA group 1 (minimally or unprocessed): 10 points<br>NOVA group 2 (culinary ingredients): 7.5 points<br>NOVA group 3 (processed): 5 points<br>NOVA group 4 (ultra-processed): -10 points	Points allocated to products within the NOVA attribute is as follows:<br>NOVA group 1 (minimally or unprocessed): 0 points<br>NOVA group 2 (culinary ingredients): -2.5 points<br>NOVA group 3 (processed): -5 points<br>NOVA group 4 (ultra-processed): -10 points	In order to emphasise the relevance of ultra-processing more strongly within the FCS and improve differentiation between foods based on level of processing.
Specific lipids	Only long-chain omega-3’s and trans fats receive a full weight; other specific lipids	All specific lipids receive a full weight. The domain receives a half-weight.	The scientific evidence for health impacts of long-chain omega-3’s

	receive a half weight. The domain receives a half-weight.		and trans fats is considerably greater than for ALA, MCFAs or dietary cholesterol.
Fiber and protein	Full weight is given to the domain, but within the domain protein is only given a half weight.	Half weight is given to the domain.	Dietary fiber is strongly related to numerous health outcomes, and with stronger relative evidence than for total protein which generally has mixed or even harmful associations.

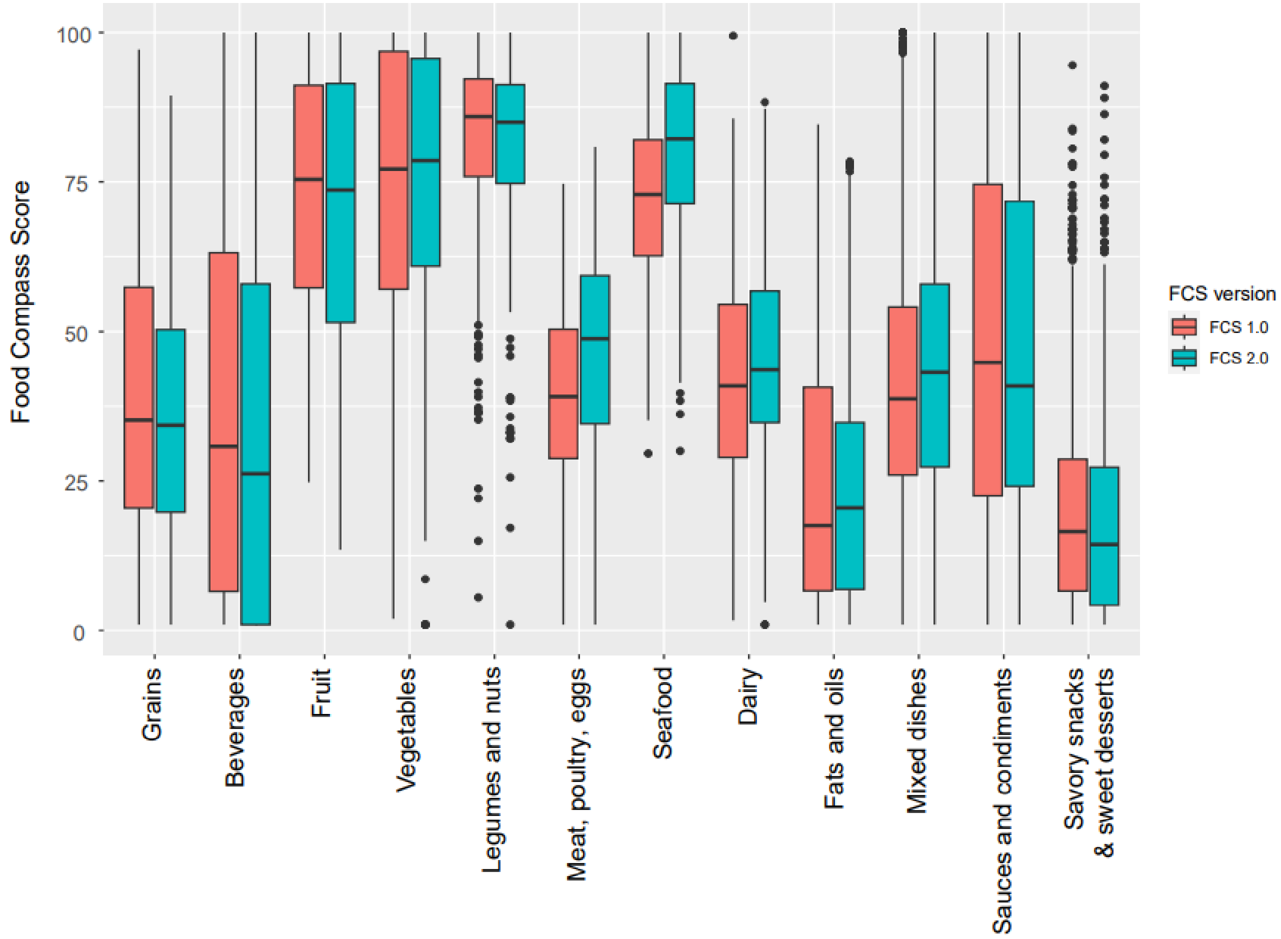

Figure S1. Updated (blue) and original (red) Food Compass Scores (FCS) for 9273 products consumed by US adults based on NHANES 2001/02-2017-18, across 12 major food categories. Standard box plots are shown, with horizontal lines representing the median value, bounds of boxes representing the 25th (lower bound) and 75th (upper bound) percentile values, whiskers representing 1.5*interquartile range from the 25th percentile (for the lower whisker) and the 75th percentile (for the upper whisker), and the black dots beyond these bounds representing outliers. Meat includes beef, pork, lamb and game, organ meats, and cured meat.

Table S2. Food Compass scores for 9273 unique foods and beverages consumed by US adults (NHANES 01/02-17/18), overall and within 12 major food groups

	n	Original FCS mean ±SD	FCS 2.0 mean ±SD	Change	Original FCS median (IQR)	FCS 2.0 median (IQR)	Change
**Overall**	9273	45.8 ± 27.7	47.3 ± 27.9	+1.5	43.6 (24.3-65.3)	46.9 (24.7-67.4)	+3.3
Beverages	409	36.6 ± 30.3	32.1 ± 28.7	-4.5	30.8 (6.5-63.2)	26.2 (1.0-57.9)	-4.6
Grains	1005	38.1 ± 22.3	35.7 ± 20.0	-2.4	35.2 (20.5-57.4)	34.3 (19.8-50.3)	-0.9
Fruit	242	72.7 ± 20.3	70.5 ± 22.8	-2.2	75.4 (57.2-91.2)	73.7 (51.5-91.5)	-1.7
Vegetables	1468	74.7 ± 21.9	76.2 ± 20.5	+1.5	77.3 (57.1-97.0)	78.7 (61.0-95.8)	+1.4
Legumes and nuts	312	81.7 ± 16.0	80.8 ± 15.8	-0.9	86.0 (75.8-92.2)	84.9 (74.7-91.3)	-1.1
Meat* poultry eggs	1069	36.2 ± 17.6	44.1 ± 19.4	+7.9	39.1 (28.8-50.4)	48.7 (34.6-59.4)	+9.6
Seafood	330	71.8 ± 14.0	80.5 ± 13.8	+8.7	72.9 (62.5-82.1)	82.2 (71.3-91.5)	+9.3
Dairy	319	42.3 ± 17.6	44.6 ± 16.9	+2.3	40.5 (28.4-54.4)	43.2 (34.6-56.4)	+2.7
Fats and oils	178	26.6 ± 24.3	24.9 ± 22.0	-1.7	18.0 (6.5-40.8)	20.6 (6.9-35.6)	+2.6
Mixed dishes	2551	41.0 ± 20.6	44.0 ± 21.0	+3.0	38.7 (26.0-54.1)	43.2 (27.4-57.9)	+4.5
Sauces and condiments	196	47.8 ± 29.1	47.0 ± 28.0	-0.8	44.8 (22.5-74.8)	40.9 (24.1-71.8)	-3.9
Savory snacks and sweet desserts	1194	20.4 ± 17.7	18.4 ± 16.9	-2.0	16.5 (6.6-28.6)	14.4 (4.2-27.3)	-2.1

*Meat includes beef, pork, lamb and game, organ meats, and cured meat.

Table S3. Food Compass scores for 9273 unique foods and beverages consumed by US adults (NHANES 01/02-17/18), overall and within 44 food subgroups

	n	Original FCS mean ±SD	FCS 2.0 mean ±SD	Change	Original FCS median (IQR)	FCS 2.0 median (IQR)	Change
Waters	6	9.2 ± 7.4	5.5 ± 7.6	-3.7	7.2 (6.6-10.0)	2.9 (1.0-4.9)	-4.3
Coffee and tea	106	29.7 ± 21.7	27.2 ± 23.2	-2.5	26.2 (13.6-46.3)	20.6 (10.2-44.3)	-5.6
Fruit and vegetable juices	79	72.4 ± 15.3	65.5 ± 14.3	-6.9	74.3 (62.6-82.6)	67.7 (57.7-73.3)	-6.6
Sweet drinks	199	28.0 ± 29.1	23.1 ± 27.0	-4.9	20.6 (1.0-47.4)	12.1 (1.0-37.9)	-8.5
Diet drinks	19	25.4 ± 24.0	23.6 ± 20.9	-1.8	19.5 (1.0-51.7)	25.6 (1.0-44.7)	+6.1
Bread	390	25.9 ± 18.4	25.7 ± 17.1	-0.2	22.2 (11.7-35.9)	22.0 (13.2-36.3)	-0.2
Rice and pasta	98	42.5 ± 25.7	49.1 ± 22.5	+6.6	34.2 (20.9-67.4)	43.7 (30.4-68.7)	+9.5
Cold cereal	243	51.1 ± 20.7	41.4 ± 19.5	-9.7	54.1 (38.4-67.3)	43.6 (25.6-56.5)	-10.5
Cooked cereal	179	42.1 ± 19.4	43.3 ± 17.2	+1.2	39.4 (24.9-61.5)	42.5 (28.6-56.5)	+3.1
Cereal bars	95	42.3 ± 16.0	33.6 ± 14.6	-8.7	42.5 (29.3-55.8)	34.6 (22.5-45.1)	-7.9
General fruit	150	69.7 ± 20.2	67.1 ± 22.8	-2.6	72.2 (53.7-85.8)	68.5 (49.0-87.2)	-3.7
Berries	37	80.9 ± 22.9	77.4 ± 27.0	-3.5	95.5 (61.4-100.0)	95.7 (54.7-100.0)	+0.2
Citrus	17	88.6 ± 13.4	87.8 ± 14.8	-0.8	92.3 (81.7-100.0)	93.7 (82.4-100.0)	+1.4
Dried fruit	38	69.6 ± 14.3	69.6 ± 16.0	0.0	68.3 (60.3-80.4)	68.3 (59.9-83.2)	0.0
General vegetables	458	80.8 ± 16.8	82.0 ± 15.5	+1.2	82.8 (69.2-96.3)	83.3 (72.5-95.0)	+0.5
Green vegetables	394	93.2 ± 10.4	93.0 ± 10.0	-0.2	100.0 (88.8-100.0)	100.0 (86.4-100.0)	0.0
Red and orange vegetables	144	77.4 ± 16.3	78.3 ± 15.5	+0.9	77.5 (68.5-90.9)	78.1 (70.3-89.9)	+0.6
Starchy vegetables	429	51.9 ± 13.0	56.0 ± 13.5	+4.1	52.4 (42.0-59.9)	57.6 (47.6-63.8	+5.2
Pickled vegetables	43	56.7 ± 27.0	54.0 ± 28.1	-2.7	60.1 (40.6-76.2)	55.8 (39.8-71.1)	-4.3

Legumes	212	79.2 ± 15.9	78.9 ± 16.1	-0.3	84.3 (71.5-89.4)	83.4 (74.4-89.0)	-0.9
Nuts and seeds	100	87.1 ± 15.0	85.0 ± 14.2	-2.1	91.6 (79.8-98.5)	88.5 (77.4-94.7)	-3.1
Beef	91	33.2 ± 5.5	44.3 ± 5.5	+11.1	33.1 (30.5-36.6)	45.0 (42.7-47.2)	+11.9
Pork	82	34.9 ± 7.5	44.4 ± 8.5	+9.5	37.1 (32.8-39.3)	46.4 (43.4-48.3)	+9.3
Lamb and game	48	38.5 ± 8.3	49.2 ± 8.4	+10.7	37.4 (31.6-42.0)	48.7 (41.8-52.8)	+11.3
Organ meats	13	51.2 ± 17.1	61.7 ± 14.7	+10.5	53.6 (35.7-64.0)	69.6 (48.8-72.6)	+16.0
Poultry	401	44.4 ± 10.0	52.3 ± 12.2	+7.9	46.5 (38.5-52.1)	55.6 (46.3-61.7)	+9.1
Cured meats	189	6.0 ± 7.5	10.5 ± 10.1	+4.5	1.0 (1.0-9.4)	6.4 (1.0-18.6)	+5.4
Eggs	245	46.2 ± 12.5	54.3 ± 12.6	+8.1	47.8 (39.1-54.4)	56.3 (46.7-63.7)	+8.5
Seafood	330	71.8 ± 14.0	80.5 ± 13.8	+8.7	72.9 (62.5-82.1)	82.2 (71.3-91.5)	+9.3
Milk	147	41.9 ± 16.3	45.7 ± 18.1	+3.8	41.3 (28.3-54.4)	48.9 (30.5-60.4)	+7.6
Cheese	93	31.6 ± 8.8	38.9 ± 8.7	+7.3	30.0 (26.7-35.0)	39.4 (35.6-43.7)	+9.4
Yogurt	48	57.2 ± 17.3	53.1 ± 19.2	-4.1	55.3 (45.4-71.8)	47.1 (40.2-68.8)	-8.2
Plant dairy	31	53.6 ± 21.0	43.2 ± 19.6	-10.4	54.9 (38.6-70.2)	43.7 (29.9-61.3)	-11.2
Plant oils	107	37.4 ± 25.4	32.3 ± 24.2	-5.2	34.2 (16.4-48.7)	28.8 (14.2-47.3)	-5.4
Dairy and animal fats	71	10.2 ± 8.2	13.7 ± 11.3	+3.5	8.0 (4.0-16.6)	11.6 (2.6-21.0)	+3.6
Mixed vegetable dishes	183	62.4 ± 16.4	64.1 ± 14.9	+1.7	61.2 (50.8-72.6)	62.0 (55.2-71.4)	+0.8
Mixed meat dishes	628	48.0 ± 19.7	52.9 ± 19.5	+4.9	46.2 (35.5-58.6)	53.0 (41.1-63.9)	+6.8
Mixed rice and pasta dishes	515	46.7 ± 19.7	50.8 ± 18.7	+4.1	43.6 (31.5-62.0)	49.0 (37.8-64.6)	+5.4
Mixed grain-encased dishes	933	28.4 ± 14.7	29.9 ± 14.9	+1.5	26.8 (19.5-36.9)	27.9 (20.0-39.6)	+1.1
Soups	292	42.9 ± 19.6	45.8 ± 20.8	+2.9	41.3 (28.8-54.2)	45.4 (29.7-58.8)	+4.1
Sauces	110	45.2 ± 27.0	46.3 ± 24.6	+1.1	43.9 (21.1-67.3)	41.4 (24.8-64.5)	-2.5

Condiments	86	51.2 ± 31.5	47.8 ± 31.8	-3.4	47.5 (24.1-79.9)	40.1 (20.3-76.6)	-7.4
Savory snacks	253	33.1 ± 21.7	30.4 ± 20.0	-2.7	31.8 (16.3-53.0)	29.4 (14.6-48.0)	-2.4
Desserts	941	16.9 ± 14.7	15.2 ± 14.3	-1.7	14.1 (5.9-23.2)	11.0 (3.4-23.5)	-3.1

Table S4. Examples of changes in Food Compass Score (FCS) of individual unique foods and beverages consumed by US adults, when applying the original FCS compared to the updated FCS (FCS 2.0)[a]

	Original FCS	FCS 2.0	Change
**1. Beef category (mean ± SD)**	**33 ± 6**	**44 ± 6**	**+11**
Beef, roast, roasted, lean only eaten	35	48	+13
Beef, short ribs, cooked, lean and fat eaten	27	37	+10
Ground beef patty, breaded, cooked	41	45	+3
**2. Organ meats category (mean ± SD)**	**51 ± 17**	**62 ± 15**	**+11**
Beef liver, fried	60	70	+10
Kidney, cooked	64	73	+9
Liver paste or pate, chicken	50	56	+6
**3. Lamb and game category (mean ± SD)**	**39 ± 8**	**49 ± 8**	**+10**
Venison/deer, roasted	39	51	+12
Lamb, shoulder, cooked, lean only eaten	39	49	+10
Venison/deer steak, breaded or floured	55	59	+4
**4. Pork category (mean ± SD)**	**35 ± 8**	**44 ± 9**	**+9**
Ham, fresh, cooked, lean only eaten	40	51	+11
Pork roast, loin, cooked, lean and fat eaten	38	48	+10
Pork, pickled	1	2	+1
**5. Seafood category (mean ± SD)**	**72 ± 14**	**81 ± 14**	**+9**
Cod, battered, fried	64	75	+11
Salmon, raw	97	100	+3
Fish stick, patty or nugget from fast food	40	41	+1
**6. Eggs category (mean ± SD)**	**46 ± 13**	**54 ± 13**	**+8**
Egg, whole, fried no added fat	48	62	+14
Spanish omelet, made with onions, peppers, tomatoes, and mushrooms	68	76	+8
Egg substitute, not specified as to powdered, frozen, or liquid	50	45	-5
**7. Poultry category (mean ± SD)**	**44 ± 10**	**52 ± 12**	**+8**
Chicken thigh, rotisserie, skin eaten	43	54	+11
Chicken breast, grilled without sauce, skin not eaten	61	68	+7
Chicken nuggets	27	26	-1
**8. Cheese category (mean ± SD)**	**32 ± 9**	**39 ± 9**	**+7**
Brie cheese	27	38	+11
Ricotta cheese	40	47	+7
Imitation cheese, American or cheddar type	27	25	-2
**9. Rice and pasta category (mean ± SD)**	**43 ± 26**	**49 ± 23**	**+6**
Rice noodles, cooked	1	12	+11

Rice, brown, cooked	65	69	+4
Quinoa, no fat added	88	89	+1
**10. Cold cereal category (mean ± SD)**	**51 ± 21**	**41 ± 20**	**-10**
Kellogg's Froot Loops	46	29	-17
Kellogg's Cocoa Krispies	28	16	-12
Kellogg's All-Bran	62	59	-3
**11. Plant dairy category (mean ± SD)**	**54 ± 21**	**43 ± 20**	**-9**
Soy milk, light, chocolate	66	49	-17
Almond milk, unsweetened	83	73	-10
Coconut milk, used in cooking	36	41	+5
**12. Cereal bars category (mean ± SD)**	**42 ± 16**	**34 ± 16**	**-8**
Kellogg's Nutri-Grain Cereal Bar	48	35	-13
Nature Valley Chewy Trail Mix Granola Bar	39	31	-8
Breakfast bar, date, with yogurt coating	59	55	-4
**13. Fruit and vegetable juices category (mean ± SD)**	**72 ± 15**	**66 ± 14**	**-6**
Peach juice, with sugar	48	34	-14
Mixed vegetable juice	100	95	-5
100% Cranberry juice	58	55	-3
**14. Plant oils category (mean ± SD)**	**37 ± 25**	**32 ± 24**	**-5**
Margarine-oil blend, tub	46	37	-9
Olive oil	80	78	-2
Coconut oil	3	13	+10
**15. Sweet drinks category (mean ± SD)**	**28 ± 29**	**23 ± 27**	**-5**
High protein ready-to-drink nutritional drink	88	70	-18
Soft drink, cola	1	1	0
Fruit smoothie with whole fruit and dairy	72	74	+2
**16. Yogurt category (mean ± SD)**	**57 ± 17**	**53 ± 19**	**-4**
Yogurt, fruit, non-fat milk, light	99	87	-12
Yogurt, Greek, chocolate, non-fat	72	61	-11
Yogurt, Greek, low fat milk, plain	77	82	+5

a. Example products are shown from subcategories with the greatest positive (subcategories 1-9) and negative (subcategories 10-16) mean change in FCS (from FCS 1.0 to 2.0). Example products were selected to demonstrate the spectrum of changes in FCS at the individual product level.

Table S5. Updated (FCS 2.0) and original (FCS 1.0) Food Compass Scores, NOVA classification, Nutri-Score category and Health Star Rating for each of 9,273 unique foods and beverages consumed by U.S. adults.

See pages at the end of this supplement (pages 32-286)

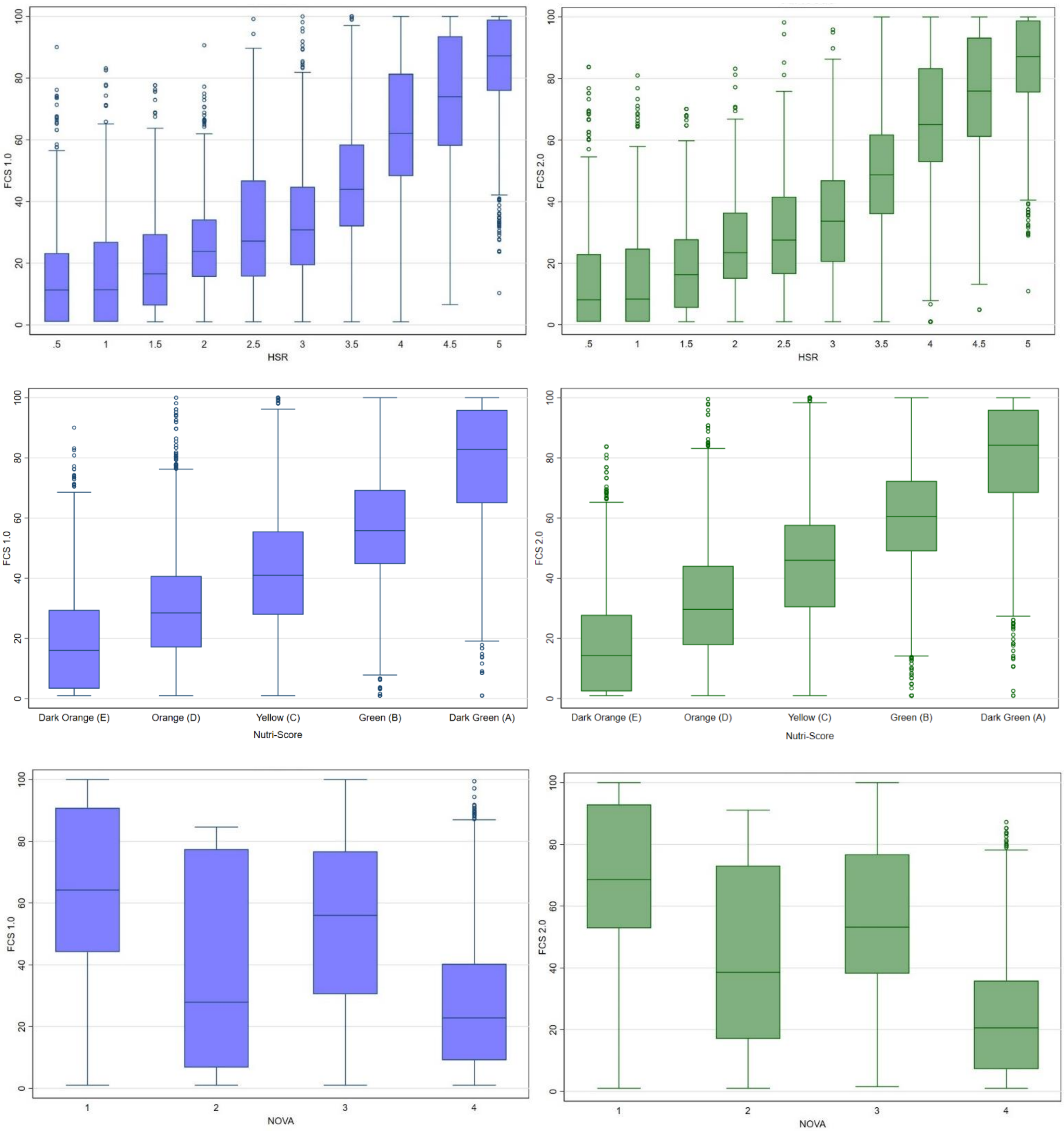

Figure S2. Updated (green) and original (blue) Food Compass scores according to HSR category (top), Nutri-Score category (middle) and NOVA category (bottom) for unique foods and beverages consumed in the United States (FNDDS 01/02-17/18). Standard box plots are shown, with horizontal lines representing the median value, bounds of boxes representing the 25th (lower bound) and 75th (upper bound) percentile values, whiskers representing 1.5*interquartile range from the 25th percentile (for the lower whisker) and the 75th percentile (for the upper whisker), and the black dots beyond these bounds representing outliers. For the HSR box plots, 9273 unique products were included. For the NOVA box plots, 4906 unique individual products (i.e. excluding mixed dishes and meals with non-integer NOVA scores) were included.

Table S6. Correlations between the Food Compass (Original and FCS 2.0), the Australian and New Zealand Health Star Rating (HSR), the Nutri-Score, and the NOVA food classification system

Food Category	n	Original FCS vs. HSR	FCS 2.0 vs. HSR	Original FCS vs. Nutri-Score	FCS 2.0 vs. Nutri-Score	Original FCS vs. NOVA	FCS 2.0 vs. NOVA
Beverages	409	0.48	0.52	0.21	0.25	0.65	0.72
Grains	1005	0.27	0.42	0.24	0.42	0.07	0.31
Fruit	242	0.77	0.77	0.50	0.50	0.78	0.83
Vegetables	1468	0.37	0.40	0.68	0.69	0.21	0.29
Legumes, Nuts and Seeds	312	0.46	0.55	0.34	0.41	0.50	0.61
Meat*, poultry and eggs	1069	0.66	0.67	0.74	0.72	0.48	0.61
Fish and seafood	330	0.50	0.53	0.41	0.45	0.43	0.53
Dairy	319	0.51	0.53	0.66	0.65	0.31	0.58
Fats and oils	178	0.47	0.36	0.42	0.33	0.27	0.48
Mixed dishes	2551	0.62	0.63	0.61	0.63	0.56	0.68
Sauces and condiments	196	0.57	0.59	0.55	0.57	0.38	0.49
Savory snacks and sweet desserts	1194	0.38	0.45	0.30	0.36	0.20	0.32
**Overall**	9273	0.70	0.73	0.69	0.72	0.56	0.68

9273 unique food items were included from across FNDDS 2001-02 to 2017-18. HSR and Nutri-Score numerical scores (0.5-5 and 1-5, respectively) were used to assess Spearman correlations (values shown) with the original and updated (2.0) Food Compass score (FCS). NOVA non-integer scores were used for mixed dishes (an energy-weighted average based on the NOVA classification of the ingredients in the dish). To facilitate a clearer representation of the correlation between FCS and NOVA in the same direction, the sign of NOVA was flipped (multiplied by -1).

* Meat includes beef, pork, lamb and game, organ meats, and cured meat.

Table S7. Multivariable-adjusted, cross-sectional associations of the individual Food Compass 2.0 Score (i.FCS) with major clinical risk factors and prevalent health conditions among U.S. adults, 1999–2018

Clinical risk factors	Difference[a]	*P*-value[b]
**BMI, kg/m²**		
per SD (10.8 points)[c]	-0.56 (-0.65, -0.47)***	<0.001
Quintiles		
Q1	0.00 (ref)	
Q2	-0.34 (-0.62, -0.06)*	0.019
Q3	-0.59 (-0.88, -0.30)***	<0.001
Q4	-0.82 (-1.11, -0.53)***	<0.001
Q5	-1.61 (-1.90, -1.32)***	<0.001
**Systolic blood pressure, mmHg**		
per SD	-0.55 (-0.77, -0.34)***	<0.001
Quintiles		
Q1	0.00 (ref)	
Q2	-0.66 (-1.26, -0.06)*	0.032
Q3	-0.84 (-1.52, -0.15)*	0.018
Q4	-0.97 (-1.66, -0.28)**	<0.01
Q5	-1.71 (-2.38, -1.04)***	<0.001
**Diastolic blood pressure, mmHg**		
per SD	-0.46 (-0.63, -0.29)***	<0.001
Quintiles		
Q1	0.00 (ref)	
Q2	-0.23 (-0.71, 0.24)	0.333
Q3	-0.77 (-1.27, -0.27)**	<0.01
Q4	-0.65 (-1.16, -0.13)*	0.015
Q5	-1.32 (-1.85, -0.80)***	<0.001
**LDL-C, mg/dL**		
per SD	-1.49 (-2.10, -0.87)***	<0.001
Quintiles		
Q1	0.00 (ref)	
Q2	-0.70 (-2.46, 1.07)	0.440
Q3	-2.08 (-3.89, -0.27)*	0.026
Q4	-1.36 (-3.22, 0.49)	0.153
Q5	-3.97 (-5.73, -2.22)***	<0.001
**HDL-C, mg/dL**		
per SD	1.61 (1.41, 1.81)***	<0.001
Quintiles		
Q1	0.00 (ref)	
Q2	1.14 (0.56, 1.71)***	<0.001
Q3	2.16 (1.58, 2.73)***	<0.001

Q4	2.77 (2.20, 3.35)***	<0.001
Q5	4.20 (3.61, 4.79)***	<0.001
**Triglycerides, mg/d**		
per SD	-0.96 (-2.55, 0.64)	0.242
Quintiles		
Q1	0.00 (ref)	
Q2	0.76 (-3.31, 4.84)	0.714
Q3	2.59 (-1.73, 6.90)	0.242
Q4	-0.94 (-5.54, 3.66)	0.688
Q5	-1.18 (-6.02, 3.67)	0.635
**TC: HDL ratio, 1 unit**		
per SD	-0.12 (-0.13, -0.10)***	<0.001
Quintiles		
Q1	0.00 (ref)	
Q2	-0.07 (-0.13, -0.01)*	0.026
Q3	-0.15 (-0.21, -0.10)***	<0.001
Q4	-0.19 (-0.24, -0.13)***	<0.001
Q5	-0.30 (-0.35, -0.25)***	<0.001
**HbA1c, %**		
per SD	-0.02 (-0.02, -0.01)***	<0.001
Quintiles		
Q1	0.00 (ref)	
Q2	-0.00 (-0.02, 0.02)	0.989
Q3	-0.00 (-0.02, 0.02)	0.990
Q4	-0.02 (-0.05, -0.00)*	0.041
Q5	-0.04 (-0.07, -0.02)***	<0.001
**Fasting plasma glucose, (mg/dL)**		
per SD	-0.36 (-0.67, -0.05)*	0.024
Quintiles		
Q1	0.00 (ref)	
Q2	-0.31 (-1.14, 0.53)	0.475
Q3	0.01 (-0.84, 0.86)	0.980
Q4	-0.52 (-1.40, 0.35)	0.243
Q5	-1.26 (-2.14, -0.38)**	<0.01
**Prevalent health conditions**		
**Metabolic syndrome[d]**		
per SD	0.86 (0.83, 0.89)***	<0.001
Quintiles		
Q1	0.00 (ref)	
Q2	0.88 (0.80, 0.97)*	0.014
Q3	0.82 (0.75, 0.90)***	<0.001
Q4	0.78 (0.70, 0.87)***	<0.001
Q5	0.65 (0.59, 0.71)***	<0.001

**Diabetes**[e]		
per SD	0.97 (0.92, 1.03)	0.284
Quintiles		
Q1	0.00 (ref)	
Q2	0.97 (0.84, 1.11)	0.628
Q3	0.99 (0.85, 1.14)	0.860
Q4	0.97 (0.83, 1.12)	0.642
Q5	0.88 (0.75, 1.04)	0.131
**CVD**[f]		
per SD	0.92 (0.88, 0.96)***	<0.001
Quintiles		
Q1	0.00 (ref)	
Q2	0.99 (0.83, 1.17)	0.878
Q3	0.97 (0.84, 1.13)	0.708
Q4	0.90 (0.77, 1.04)	0.152
Q5	0.86 (0.74, 0.99)*	0.035
**Cancer**		
per SD	0.93 (0.89, 0.98)**	<0.01
Quintiles		
Q1	0.00 (ref)	
Q2	0.96 (0.83, 1.12)	0.604
Q3	0.91 (0.79, 1.07)	0.256
Q4	0.77 (0.67, 0.89)***	<0.001
Q5	0.86 (0.74, 1.00)	0.051
**Lung disease**		
per SD	0.90 (0.87, 0.94)***	<0.001
Quintiles		
Q1	0.00 (ref)	
Q2	0.92 (0.83, 1.02)	0.123
Q3	0.84 (0.77, 0.92)***	<0.001
Q4	0.78 (0.70, 0.88)***	<0.001
Q5	0.73 (0.65, 0.82)***	<0.001
**Optimal cardiometabolic health**[g]		
per SD	1.22 (1.14, 1.30)***	<0.001
Quintiles		
Q1	0.00 (ref)	
Q2	1.13 (0.95, 1.35)	0.182
Q3	1.28 (1.07, 1.53)**	<0.01
Q4	1.33 (1.09, 1.61)**	<0.01
Q5	1.79 (1.48, 2.16)***	<0.001

BMI body mass index, CI confidence interval, CVD cardiovascular disease, HbA1c hemoglobin A1c, HDL-C high-density lipoprotein cholesterol, i.FCS individual dietary Food Compass Score, LDL low density lipoprotein cholesterol, TC:HDL total cholesterol to HDL-cholesterol ratio.

[a] Survey-weighted, multivariable-adjusted linear or logistic regression models incorporated

NHANES dietary recall sample weights to account for the complex survey design and response rates and provide nationally representative effect estimates and associated 95% confidence interval for the non-institutionalized U.S. population. All models adjusted for: age (years), age^2 (years), sex, race/ethnicity (Mexican-American, other Hispanic, non-Hispanic White, non-Hispanic Black, Asian/other race), education level (<HS graduate, HS graduate, some college or associates degree, ≥ college graduate), income (poverty: income ratio), smoking status (nonsmoker, former smoker, current smoker); total physical activity (MET-hours/week), alcohol use (%energy), and self-reported diabetes (yes, no).

[b] *P*-values were calculated using two-sided t-test (alpha=0.05).

[c] Individual Food Compass Score (i.FCS) calculated as the energy-weighted mean of FCS of all foods consumed, as reported in up to two 24 hr recalls per person, with a potential range from 1 to 100.

[d] Defined according to AHA/NHLBI as the presence of three or more of: HDL-C< 40mg/dL (men)/ <50mg/dL (women) or on drug treatment for low HDL-C; triglycerides ≥150mg/dL or on drug treatment for elevated triglycerides; blood pressure ≥130 SBP or ≥85 DBP or on antihypertensive drug treatment with a history of hypertension; WC≥102 cm(men)/ ≥88cm (women); and fasting plasma glucose ≥100mg/dL or on drug treatment for elevated glucose.

[e] Defined based on presence of at least one of: HbA1c level > 6.5%, fasting plasma glucose > 126mg/dL, or use of diabetes medications.

[f] Defined based on the presence of both (1) self-reported coronary heart disease, heart failure, stroke, myocardial infarction, or angina and also (2) cardiovascular medication use including use of angina, hypertension, or lipid medications.

[g] Defined based on optimal levels for each of the following: adiposity (BMI <25 kg/m2 and WC ≤88cm (women)/WC ≤102 cm (men)); blood glucose (FPG <100mg/dL and HbA1c <5.7% and not taking diabetes medication); blood lipids (TC:HDL <3.5:1 and not taking lipid lowering medication); blood pressure (SBP <120mmHg, DBP <80mmHg and not taking blood-pressure lowering medication); and history of CVD (no self-reported CHD, heart failure, myocardial infarction, stroke, or angina).

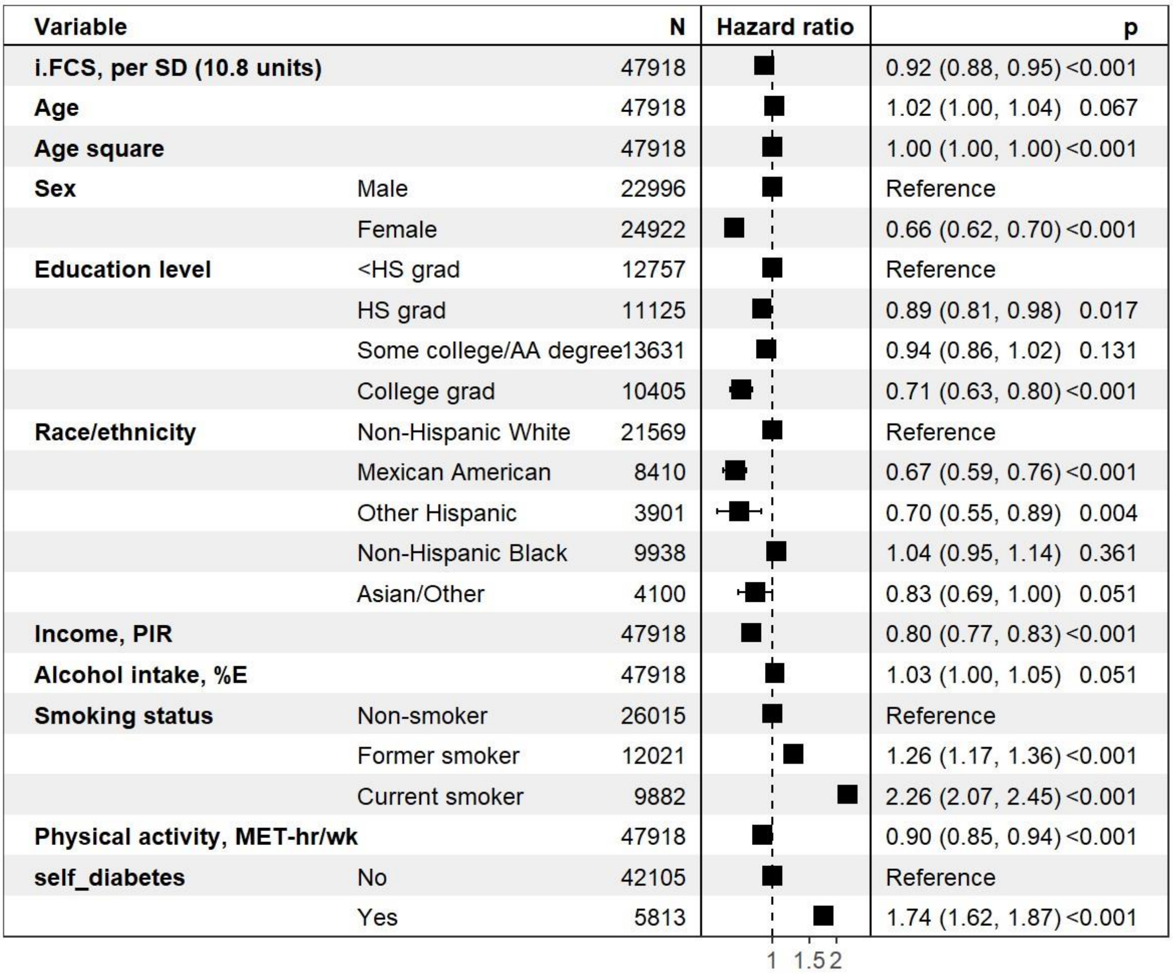

Variable		N	Hazard ratio		p
**i.FCS, per SD (10.8 units)**		47918		0.92 (0.88, 0.95)	<0.001
**Age**		47918		1.02 (1.00, 1.04)	0.067
**Age square**		47918		1.00 (1.00, 1.00)	<0.001
**Sex**	Male	22996		Reference	
	Female	24922		0.66 (0.62, 0.70)	<0.001
**Education level**	<HS grad	12757		Reference	
	HS grad	11125		0.89 (0.81, 0.98)	0.017
	Some college/AA degree	13631		0.94 (0.86, 1.02)	0.131
	College grad	10405		0.71 (0.63, 0.80)	<0.001
**Race/ethnicity**	Non-Hispanic White	21569		Reference	
	Mexican American	8410		0.67 (0.59, 0.76)	<0.001
	Other Hispanic	3901		0.70 (0.55, 0.89)	0.004
	Non-Hispanic Black	9938		1.04 (0.95, 1.14)	0.361
	Asian/Other	4100		0.83 (0.69, 1.00)	0.051
**Income, PIR**		47918		0.80 (0.77, 0.83)	<0.001
**Alcohol intake, %E**		47918		1.03 (1.00, 1.05)	0.051
**Smoking status**	Non-smoker	26015		Reference	
	Former smoker	12021		1.26 (1.17, 1.36)	<0.001
	Current smoker	9882		2.26 (2.07, 2.45)	<0.001
**Physical activity, MET-hr/wk**		47918		0.90 (0.85, 0.94)	<0.001
**self_diabetes**	No	42105		Reference	
	Yes	5813		1.74 (1.62, 1.87)	<0.001

Figure S3. Independent prospective associations of the individual Food Compass 2.0 Score (i.FCS) and each sociodemographic factor included in the multivariable model with all-cause mortality among U.S. adults, 1999–2018. The i.FCS for each person was calculated as the energy weighted mean of the FCS of all unique foods and beverages consumed, based on up to two 24 hr recalls per person, with a potential range from 1 to 100. Boxes represent the multivariable-adjusted hazard ratio; and error bars, the 95% CI; based on survey-weighted Cox proportional hazard models incorporating NHANES dietary recall sample weights to account for the complex survey design and response rates and provide nationally representative estimates for the noninstitutionalized U.S. population. All variables in the Figure were included together in the multivariable model. *P*-values were calculated using two-sided Wald test (alpha=0.05). AA Associates Degree, grad graduate, HS high school, i.FCS individual Food Compass Score, MET metabolic equivalent of task, %E percent energy, PIR ratio of family income to poverty threshold, SD standard deviation.

Table S8. Multivariable adjusted, survey-weighted prospective analysis of individual Food Compass Score (i.FCS) with all-cause and cause-specific mortality among U.S adults, 1999-2018

i.FCS	N for deaths	Mortality (Hazard Ratio [95% CI])[a]	*P*-value[b]
**All-cause**			
Per SD (10.8 points)	7481	0.92 (0.88, 0.95)	<0.001
Quintiles			
Q1	1330	1.0 (ref)	
Q2	1427	0.80 (0.73, 0.89)	<0.001
Q3	1549	0.85 (0.77, 0.95)	0.003
Q4	1647	0.82 (0.75, 0.90)	<0.001
Q5	1528	0.76 (0.68, 0.84)	<0.001
**CMD-specific[c]**			
Per SD (10.8 points)	2619	0.95 (0.89, 1.01)	0.113
Quintiles			
Q1	419	1.0 (ref)	
Q2	473	0.84 (0.70, 1.00)	0.052
Q3	551	0.91 (0.77, 1.08)	0.278
Q4	600	0.90 (0.77, 1.06)	0.195
Q5	576	0.81 (0.67, 0.98)	0.031
**Cancer-specific[d]**			
Per SD (10.8 points)	1691	0.92 (0.85, 1.00)	0.052
Quintiles			
Q1	329	1.0 (ref)	
Q2	335	0.78 (0.63, 0.96)	0.022
Q3	336	0.80 (0.65, 1.00)	0.048
Q4	358	0.79 (0.63, 0.98)	0.029
Q5	333	0.77 (0.62, 0.96)	0.019

[a] Survey-weighted Cox proportional hazard models incorporated NHANES dietary sample weights to account for the complex survey design and response rates and provide nationally representative estimates the hazard ratio and associated 95% confidence interval for the non-institutionalized U.S. population. Study time used as time variable in analysis (where baseline data collection: t = 0 yr). Models were adjusted for age (years), age^2 (years), sex, race/ethnicity (Mexican-American, other Hispanic, non-Hispanic White, non-Hispanic Black, Asian/other race), education level (<HS graduate, HS graduate, some college or associates degree, ≥ college graduate), income (poverty: income ratio), smoking status (non-smoker, former smoker, current smoker); total physical activity (MET-hours/week), alcohol use (%energy), and self-reported diabetes (yes, no).

[b] P-values were calculated using two-sided Wald test (alpha=0.05).

[c] Cardiometabolic disease (CMD)-specific mortality is the sum of deaths from coronary heart disease, stroke, and diabetes.

[d] Cancer-specific mortality includes deaths from all cancer types.

CI, confidence interval; CMD, cardiometabolic disease; i.FCS, individual, dietary Food Compass Score.

Supplementary Methods 1

Part 1. Application and validation

The FCS algorithm was updated (see Table S1 for details and rationales) based on updated evidence, availability of new data, and feedback from the scientific community. We compared FCS 2.0 to the original FCS by repeating the analyses conducted previously and described in detail elsewhere (1, 2). Briefly, we applied the original and updated Food Compass algorithm to 9273 unique foods and beverages reported in the National Health and Nutrition Examination Survey (NHANES) from the years 2001-02 to 2017-18, contained within FNDDS 2001-02 to 2017-18. Products were scored in their form as consumed. We excluded infant formula, baby foods, specialized dietary foods, alcohol, and products providing <5 kcal per 100g. Attributes with missing values were handled as in our 2022 FCS publication and described briefly in **Text S2**. The final score distributions were calculated overall and stratified into 12 major food groups and 44 nested food subgroups.

We assessed face validity by evaluating FCS values across food groups and subgroups, considering the distribution of scores and the specific foods scoring higher, middle, and lower in each group. The convergent and discriminate validity of Food Compass 2.0 was assessed by comparing it to the NOVA processing classification system (3), the 2023 Nutri-Score algorithm (4) and the 2020 Australian and New Zealand Health Star Rating (HSR) algorithm (5). The NOVA classification for homemade mixed dishes that could include various ingredients of differing NOVA classes was calculated as the energy-weighted sum of the NOVA score of its ingredients.

Part 2. Validation of individual's Food Compass 2.0 Score (i.FCS)

We further evaluated the validity of the FCS 2.0 by examining the associations between an individual's FCS 2.0 based on cumulative scores of items consumed and a validated healthy dietary pattern (construct validity) and major health outcomes (criterion validity).

Study design and participants

We used nationally representative data among 47,999 adults aged 20–85 years enrolled in 10 NHANES cycles, 1999–2000 through 2017–2018. The mean (SD) age was 47.2 years (17.1) and 52.2% were female. NHANES collects information on sociodemographic factors, clinical risk factors, health conditions, and dietary and other lifestyle habits from recurrent cross-sectional nationally representative samples. Through linkage to the National Death Index (NDI), we prospectively evaluated mortality outcomes among 47,918 NHANES adult participants across all cycles (excluding 81 participants who did not have linked NDI information). NHANES was approved by the Institutional Review Board at the National Center for Health Statistics (NCHS) and all participants provided informed written consent. Details of NHANES data collection methods, survey design and sampling are provided in our 2022 FCS publication (2).

Dietary assessment and calculation of i.FCS

Dietary intake was assessed in NHANES using standardized 24 hr dietary recalls, administered by trained NHANES interviewers, using United States Department of Agriculture's Automated Multiple Pass Method (6). We used all complete and reliable (as defined previously (2)) dietary records available as determined by the NCHS staff, averaging the two recalls for each individual when available (two recalls were taken for 87% of respondents from 2003-04 cycles onwards).

To extend FCS for specific products to an individual's overall diet, the FCS score for each item reported in a person's diet was weighted by its percent contribution to that person's total energy intake, and then summed, to calculate an individual's Food Compass Score (i.FCS). Energy from alcohol intake was excluded from the i.FCS calculation and included as a covariate in all models. For products reported by participants within the 1999-2000 NHANES survey cycle, where there is no corresponding FNDDS database, nutrition data for each product food code was copied over from the most proximal available year. For the 115 unique products remaining without an exact food code match in other years, the product description was used to assign a food code from the earliest (i.e., most proximal) edition of the FNDDS database. The i.FCS for an individual could theoretically range from 1 to 100.

Outcomes

The i.FCS was validated against three different outcomes: (a) the Healthy Eating Index (HEI) 2015, a validated measure of a healthy dietary pattern; (b) prevalence of major clinical risk factors and health conditions; and (c) prospective risk of all-cause and cause-specific mortality.

HEI 2015, a measure based on adherence to the 2015–2020 Dietary Guidelines for Americans (DGAs), was calculated for each individual. HEI 2015 is calculated based on key food groups and nutrients emphasized in the DGAs, resulting in a score ranging from 0 to 100.

Major clinical risk factors assessed included body mass index (BMI); systolic and diastolic blood pressure (mm Hg); HDL-cholesterol, LDL-cholesterol, triglycerides (mg/dL), and the total cholesterol: HDL ratio; and HbA1c (%) and fasting plasma

glucose (mg/dL). Health conditions assessed included the presence of metabolic syndrome, diabetes, cardiovascular disease (CVD), cancer, and lung disease; as well as optimal cardiometabolic health, characterized by optimal levels of adiposity, blood glucose, blood lipids, blood pressure, and absence of prior clinical CVD **(Table S7**). Missing data were handled as detailed previously (2).

NHANES survey data were linked to the NDI mortality data from the Public-Use Linked Mortality Files (7), allowing prospective assessment of mortality through Jan 1, 2019. Deaths were adjudicated by the NCHS using standardized procedures. Our primary endpoint was mortality from all causes with secondary outcomes of deaths from cardiometabolic disease (sum of deaths from coronary heart disease, stroke, and diabetes) and from all cancer.

Statistical analysis

The relationship of i.FCS with HEI 2015 was assessed using Spearman correlation. To assess relationships with clinical risk factors, prevalent health conditions, and mortality outcomes, i.FCS was evaluated by each quintile and per each standard deviation (SD) unit. We used survey-weighted linear regressions with robust standard errors to estimate the association of i.FCS with continuous risk factors; and survey-weighted logistic regression to estimate the odds ratio (OR) and 95% confidence interval (CI) for the association with prevalent health conditions. Multivariable models were used to minimize potential confounding, with covariates including age and age-squared (continuous), sex, race/ethnicity (non-Hispanic White, non-Hispanic Black, other Hispanic, Mexican-American, Asian/other), education (<high school graduate, high school graduate, some college education, college graduate or more), income (based on family poverty: income ratio,

continuous), smoking status (non-smoker, former, current), alcohol use (percentage energy contribution from alcohol, continuous), total physical activity (MET-hours per week, continuous), and self-reported prevalent diabetes. Detailed information on how these variables were assessed and derived has been reported (2).

Survival analyses used survey-weighted Cox proportional hazard models to estimate the hazard ratio (HR) and 95%CI between i.FCS and risk of all-cause and cause-specific (cardiometabolic and cancer) mortality. Study time was used as the time variable. Participants contributed person-time from the time of their enrolment until death or end-off follow-up on January 1, 2019, whichever came first. We censored early deaths within 3 months of baseline, to minimize reverse causation. Multivariable models were adjusted as described above for the cross-sectional analyses. The proportional hazards assumption was assessed with visual Kaplan-Meier plots and statistical testing of Schoenfeld residuals (and not rejected if $p > 0.05$), adjusted for age and age-squared (continuous).

All analyses incorporated NHANES dietary sample weights to account for the complex survey design and response rates and provide nationally representative estimates for the non-institutionalized U.S. population. Statistical significance was defined as two-tailed alpha=0.05.

Supplementary Methods 2. Missing attribute values in FNDDS 2001-2018 for nutrient profiling

Iodine and trans fats attributes were missing across the entire FNDDS 2001-2018 database, and so were excluded from the scoring of Food Compass in this report. Artificial sweeteners, flavors or colors, partially hydrogenated oils, interesterified or hydrogenated oils, high fructose corn syrup and monosodium glutamate (MSG) attributes were included in scoring in this analysis for items that could be manually matched to a branded food or beverage (n=321, ~95% of all branded items in FNDDS), where the branded item's ingredient list was used to code these attributes.

For total flavonoids, vitamin D, and choline attributes, values were missing for products only in certain survey cycles. Where unique food codes had attribute data available in one or more cycles of the FNDDS database, the attribute value per standard quantity in the most proximal year available was carried backwards or forwards to any year with a missing attribute value. For products without any attribute data available across any database editions, predictive mean matching (PMM) imputation was used to address the remaining missing values. The details of PMM approach used have been published (2).

Table S9. Domains and attributes of the Food Compass 2.0*†

Nutrient Ratios	Vitamins (top 5)	Minerals (top 5)	Food-based Ingredients	Additives	Processing	Specific Lipids (0.5 weight) (top 3)	Fiber & Protein	Phytochemicals (0.5 weight)
Unsaturated: Saturated fat ratio #	Vitamin A	Calcium	Fruits (non-dried and dried)	Added sugar	NOVA classification	ALA	Total fiber	Total flavonoids
Fiber: Carbohydrate ratio	Thiamin (B1)	Phosphorus	Vegetables, non-starchy (non-dried and dried)	Nitrites	Fermentation	EPA + DHA	Total protein	Total carotenoids
Potassium: Sodium ratio	Riboflavin (B2)	Magnesium	Beans & legumes	Artificial sweeteners, flavors, or colors	Frying	Medium-chain fatty acids (MCFA)		
	Niacin (B3)	Iron	Whole grains	Partially hydrogenated oils		Dietary cholesterol		
	Vitamin B6	Zinc	Nuts and seeds	Interesterified or hydrogenated oils		Trans fats §		
	Folate (B9)	Copper	Seafood	High fructose corn syrup				
	Cobalamin (B12)	Selenium	Yogurt	Monosodium glutamate (MSG)				
	Vitamin C	Sodium	Plant oils					
	Vitamin D	Potassium	Refined carbohydrates					
	Vitamin E	Iodine §	Red or processed Meat					
	Vitamin K							
	Choline							

*The Food Compass incorporates 54 individual attributes across 9 domains, each assessed per 100 kcal (418.4 kj) of food product. Attributes are scored across a 10-point range (0 to 10 for beneficial factors; -10 to 0 for harmful factors; and -10 to 10 for nutrient ratios which could range from harmful to beneficial). Attributes with emerging evidence for health impacts, including frying, fermentation, alpha-linoleic acid (ALA), medium-chain fatty acids (MCFAs), dietary cholesterol and total protein, were scored using half weights. Five additives with emerging evidence (all besides added sugar and nitrates) were each scored as -1 (additive present) or 0 (additive not present) for a maximum contribution of -5. Each domain then received a score, calculated as the average of all attributes in that domain (or for food ingredients, as the sum, given contents of ingredients are interdependent). For 3 domains (vitamins, minerals, specific lipids), the highest (absolute value, i.e. negative or positive) 5, 5, and 3 scores, respectively, were calculated. First, many foods with health impacts are rich in one or just a few, but not

numerous, vitamins, minerals, or specific lipids. Thus, averaging attribute scores across all 12 vitamins, for example, would incorporate many very low or zero values, causing this domain to be scored more similarly for most foods and weakening its discrimination of healthful foods with naturally higher levels of a few vitamins. Second, at the other extreme, the scoring approach for these domains constrained the potential impact of excessive fortification of multiple different vitamins, minerals, or specific lipids during processing. All domains scores were then summed, using equal weights for all domains except specific lipids and phytochemicals, where half weights were used. The final Food Compass score (FCS) was scaled across all food and beverage items to range from 1 (least healthful) to 100 (most healthful). For full scoring details of each attribute and the scaling of the final sum, see Table S10.

† Several important nutritional factors are represented in the scoring in more than one way. For example, the relative amount of unsaturated fat, as compared to saturated fat, is represented as a ratio in the Nutrient Ratios domain, while the absolute content of plant oils, rich in plant sources of unsaturated fat, is represented in the Food Ingredients domain. Potassium and sodium were included both as Nutrient Ratios given evidence for their biologic interaction, and separately as Minerals given evidence that their absolute intakes also influence health. Carbohydrate and fiber were included as Nutrient Ratios given evidence that their ratio predicts healthfulness of carbohydrate-rich foods; and separately as total fiber (in Fiber & Protein) given evidence that the absolute intake of dietary fiber, but not total carbohydrate, influences health. Partially hydrogenated oils (emerging, scored with a half-weight) were included in addition to trans fats content (scored -10 to 0 in Specific Lipids) based on emerging evidence that industrial trans fats may have greater adverse health effects than naturally occurring trans fats; and that presence of such partially hydrogenated oils may serve as a marker of more intensive and potentially adverse industrial processing.

# Dairy products (yogurt, cheeses, milk, and dairy fats) are given half weight for the scoring of the Unsaturated: saturated fat ratio within the Nutrient Ratio domain as the evidence for harmful health effects of saturated fat from dairy is less certain.

§ Iodine and trans fats were excluded from scoring in this analysis, as these attributes are not available within in the available USDA databases used (FNDDS, FPED, flavonoid database). Artificial sweeteners, flavors, or colors, partially hydrogenated oils, interesterified or hydrogenated oils, high fructose corn syrup and monosodium glutamate (MSG) were included in scoring in this analysis only for items that could be manually matched to a branded food or beverage (n=321), where the branded item’s ingredient list could be used to code these attributes.

ALA, alpha-linolenic acid. EPA + DHA, eicosapentaenoic acid + docosahexaenoic acid.

Table S10. Scoring principles for the 54 attributes within 9 domains of the Food Compass 2.0*

Attributes (per 100 kcal [418.4 kj])	Attribute Points	Target for low score	Target for high score	Target Reference†
**Nutrient Ratios ‡**				
Unsaturated:Saturated fat ratio (log)	-10 to 10	-0.66	1.77	5th, 95th pctl
Fiber:Carbohydrate ratio (log)	-10 to 10	-7.02	-0.78	5th, 95th pctl
Potassium:Sodium ratio (log)	-10 to 10	-2.02	3.30	5th, 95th pctl
**Vitamins (top 5) §**				
Vitamin A, RAE	0 to 10	0	225 ug	25% RDA
Thiamin (B1)	0 to 10	0	0.3 mg	25% RDA
Riboflavin (B2)	0 to 10	0	0.325 mg	25% RDA
Niacin (B3)	0 to 10	0	4 mg	25% RDA
Vitamin B-6	0 to 10	0	0.325 mg	25% RDA
Folate, DFE (B9)	0 to 10	0	100 ug	25% RDA
Cobalamin (B12)	0 to 10	0	0.6 ug	25% RDA
Vitamin C	0 to 10	0	22.5 mg	25% RDA
Vitamin D (D2 + D3)	0 to 10	0	3.75 ug	25% RDA
Vitamin E (alpha-tocopherol)	0 to 10	0	3.75 mg	25% RDA
Vitamin K (phylloquinone)	0 to 10	0	30 ug	25% AI
Choline, total	0 to 10	0	137.5 mg	25% AI
**Minerals (top 5) §**	0 to 10	0		
Calcium	0 to 10	0	250 mg	25% RDA
Phosphorus	0 to 10	0	175 mg	25% RDA
Magnesium	0 to 10	0	105 mg	25% RDA
Iron	0 to 10	0	4.5 mg	25% RDA
Zinc	0 to 10	0	2.75 mg	25% RDA
Copper	0 to 10	0	0.225 mg	25% RDA
Selenium	0 to 10	0	13.75 ug	25% RDA
Sodium	-10 to 0	575 mg	0	25% RDA
Potassium	0 to 10	0	1175 mg	25% RDA

Iodine	0 to 10	0	37.5 ug	25% RDA
**Food Ingredients (sum) ǁ**				
Fruits	0 to 10	0	1.75 cups	95th pctl
Fruits, dried	0 to 10	0	0.75 cups	95th pctl
Vegetables, non-starchy	0 to 10	0	4.77 cups	95th pctl
Vegetables, non-starchy & dried	0 to 10	0	4.18 cups	95th pctl
Beans and legumes	0 to 10	0	0.50 cups	95th pctl
Whole grains	0 to 10	0	1.12 oz	95th pctl
Nuts and seeds	0 to 10	0	1.35 oz	95th pctl
Seafood	0 to 10	0	3.86 oz	95th pctl
Yogurt	0 to 10	0	0.81 cups	95th pctl
Plant Oils	0 to 10	0	11.31 g	95th pctl
Refined carbohydrates	-10 to 0	1.36 oz	0	95th pctl
Red or processed meat	-10 to 0	2.69 oz	0	95th pctl
**Additives ¶**				
Added sugar, % calories	-10 to 0	60%	0	
Nitrites, % calories from processed meats	-10 to 0	25%	0	
Artificial sweeteners, flavors, or colors	-1, 0	yes	no	
Partially hydrogenated oils	-1, 0	yes	no	
Interesterified or hydrogenated oils	-1, 0	yes	no	
High fructose corn syrup	-1, 0	yes	no	
Monosodium glutamate (MSG)	-1, 0	yes	no	
**Processing €**				
NOVA processing level	-10, 5, 7.5, 10	NOVA=4	NOVA=1	
Fermentation, % calories from fermented foods	0 to 10	0	50%	
Frying	-10, 0	yes	no	
**Specific Lipids (top 3) ¥**				
Cholesterol	-10 to 0	75 mg	0	25% DGA
MCFAs (8:0 + 10:0 + 12:0, g)	0 to 10	0	0.32 g	95th pctl
ALA (18:3n-3)	0 to 10	0	0.4 g	25% AI

EPA + DHA (20:5n-3 + 22:6n-3)	0 to 10	0	62.5 mg	25% REC
Trans fats, % calories	-10 to 0	30%	0	expert consensus
**Fiber & Protein #**				
Total fiber	0 to 10	0	9.5 g	25% AI
Total protein	0 to 10	0	14 g	25% RDA
**Phytochemicals §**				
Total flavonoids	0 to 10	0	23.53 mg	95th pctl
Total carotenoids	0 to 10	0	8746.81 mcg	95th pctl

* Each domain score was calculated as the average of the attribute scores in that domain (or the sum for the food ingredient domain), and the 9 domain scores were summed (with half-weights for specific lipids and phytochemicals). To minimize the influence of outliers, the range of summed scores across all 58,618 scored food and beverage items (FNDDS 2001-2018) was first truncated at the 5th and 95th percentiles (-12.1, 35.0). The final Food Compass Score (FCS) was then scaled across all food and beverage items to range from 1 (least healthful) to 100 (most healthful) using the equation: FCS = [100 – (((max score – unscaled score)/score range))*99)], or [100 – (((35.0– unscaled score)/47.1))*99)].

† When Dietary Reference Intakes (DRIs) varied by population subgroup, the DRI was selected for adults age 19-50 years (and for men when varying by sex). Across attributes, the 25% threshold for DRIs was identified as most consistently distinguishing foods with higher vs. lower levels of these nutrients and was generally similar to the 95% percentile value of content across all foods and beverages reported in NHANES 2015-16. For attributes without DRIs, scoring was based on the distributions of relevant, unique food and beverage items reported in FNDDS 2001-2018. Whether scoring should remain constant for other datasets and nations, or vary according to the local food items, is a topic for further investigation.

‡ Nutrient ratios were each scored on a log-linear scale from -10 to 10. To prevent excessive scoring influence of small amounts of these nutrients in any food item, the unsaturated: saturated fat ratio was not calculated for items with <10% energy from fat; the fiber: carbohydrate ratio, for items with <10% energy from carbohydrate; and potassium: sodium ratio, for items with <10 mg of potassium and sodium per 100 kcal. Dairy products (yogurt, cheeses, milk, and dairy fats) are given half weight for the scoring of the Unsaturated: saturated fat ratio within the Nutrient Ratio domain as the evidence for harmful health effects of saturated fat from dairy is less certain. Absolute levels of fiber were also separately included under Fiber & Protein; and of potassium and sodium, under Minerals; to capture their separate health effects independent of these interactions represented by the ratios.

§ Vitamins, minerals, flavonoids, and carotenoids were each scored on a linear scale from 0 to 10, except sodium which was scored on a linear scale from -10 to 0.

‖ Food-based ingredients were each scored on a linear scale from 0 to 10 for healthful ingredients and from -10 to 0 for harmful ingredients (refined carbohydrates, red or processed meat). Refined carbohydrates was defined as the sum of refined grains and added sugars. The 95th percentile for each food-based ingredient was calculated based on a subset of relevant foods assessed (i.e. yogurt for the food-based yogurt attribute). Separate 95th percentile values were calculated and used for dried vs. non-dried fruits and dried vs. non-dried, non-starchy vegetables given their different water weights. The attribute scores in this domain were summed (not averaged), because contents of each ingredient are mutually interdependent.

¶ Because added sugar content is nonlinear (skewed) across the food supply, it was scored as follows: 0 (0% calories), -1 (<2.5% calories), -2 (<5% calories), -3 (<10% calories), -4 (<15% calories), -5 (<20% calories), -6 (<30% calories), -7 (<40% calories), -8 (<50% calories), -9 (<60% calories), and -10 (≥60% calories). As no threshold has been set for nitrate content, nitrites were scored using percentage energy from processed/cured meats as a proxy. 50% energy from processed/cured meat was assigned the target low score (-10), with linear scaling down to 0% energy from processed/cured meats as the target high score (0). For calculating the domain score, added sugar and nitrites, considered to have stronger evidence for health harms, were each

provided a full weight, while the remaining additives, considered to have emerging and less robust evidence for independent health harms based on more limited studies, were each scored as -1 (additive present) or 0 (additive not present) for a maximum contribution of -5.

€ The NOVA processing classification was scored as -10, 5, 7.5, and 10 for the 4 categories of ultra-processed, processed, culinary ingredients, and un-processed/minimally foods, respectively. For mixed dishes (about 2/3 of all products), NOVA classification was calculated based on an energy-weighted score of the constituent ingredients' NOVA classification, leading to non-integer NOVA classification values. As such, attribute scores for NOVA classification were linearly scaled between the integer NOVA classes 1-4. Fermentation was defined based on energy content from yogurt or cheese. 50% energy from yogurt + cheese was assigned the target high score (10), with linear scaling down to 0% energy from yogurt or cheese as the target low score (0). Additionally, other fermented products were identified by keyword search (i.e., kefir, kombucha, injera, dosa, natto, miso, kimchi, etc.) and assigned the target high score. Frying was defined based on the name and/or cooking description of each food item (e.g., "fried catfish"). For calculating the domain score, the NOVA classification system, considered to have stronger evidence for health harms, was provided a full weight, while fermentation and frying, considered to have emerging and less robust evidence for independent health harms based on more limited studies, were each provided a half weight.

¥ Specific lipids were each scored on a linear scale from 0 to 10 (ALA, EPA+DHA, MCFA) or -10 to 0 (cholesterol, trans fats). For calculating the domain score, ALA, MCFA and cholesterol content were considered to have emerging or less robust evidence for independent health harms and were provided a half weight.

Fiber and protein were each scored on a linear scale from 0 to 10. For calculating the domain score, protein content was considered to have less robust evidence for independent health harms based on more limited studies and was provided a half weight.

AI, adequate intake. ALA, alpha-linolenic acid. DGA, dietary guidelines for Americans. EPA + DHA, eicosapentaenoic acid + docosahexaenoic acid. MCFAs, medium-chain fatty acids. Pctl, percentile. RDA, recommended daily allowance. REC, dietary recommendation. UL, upper limit intake.

Supplementary References

1. Mozaffarian D, El-Abbadi NH, O'Hearn M, Erndt-Marino J, Masters WA, Jacques P, et al. Food Compass is a nutrient profiling system using expanded characteristics for assessing healthfulness of foods. Nature Food 2021;2(10):809-18. doi: 10.1038/s43016-021-00381-y.
2. O'Hearn M, Erndt-Marino J, Gerber S, Lauren BN, Economos C, Wong JB, et al. Validation of Food Compass with a healthy diet, cardiometabolic health, and mortality among U.S. adults, 1999–2018. Nature Communications 2022;13(1):7066. doi: 10.1038/s41467-022-34195-8.
3. Monteiro CA, Cannon G, Levy RB, Moubarac JC, Louzada ML, Rauber F, et al. Ultra-processed foods: what they are and how to identify them. Public Health Nutr 2019;22(5):936-41. doi: 10.1017/s1368980018003762.
4. Merz B, Temme E, Alexiou H, Beulens JWJ, Buyken AE, Bohn T, et al. Nutri-Score 2023 update. Nat Food 2024;5(2):102-10. doi: 10.1038/s43016-024-00920-3.
5. Department of Health. Health Star Rating system Calculator and Style Guide. Internet: http://healthstarrating.gov.au/internet/healthstarrating/publishing.nsf/Content/guide-for-industry (accessed March 29 2023).
6. Moshfegh AJ, Rhodes DG, Baer DJ, Murayi T, Clemens JC, Rumpler WV, et al. The US Department of Agriculture Automated Multiple-Pass Method reduces bias in the collection of energy intakes. Am J Clin Nutr 2008;88(2):324-32. doi: 10.1093/ajcn/88.2.324.
7. National Center for Health Statistics Centers for Disease Control and Prevention. 2015 Public-Use Linked Mortality Files. Internet: https://www.cdc.gov/nchs/data-linkage/mortality-public.htm (accessed 1 August 2023).

Foodcode	Description	Food group	FCS 2.0	FCS 1.0	Difference	NOVA[a]	HSR	Nutri-Score
56204005	Quinoa, no added fat	1000_Grains	89	88	1	1	4	A
57408100	Cereal (Uncle Sam)	1000_Grains	85	97	-12	4	5	A
56200350	Cereal, cooked, instant, NS as to grain	1000_Grains	84	94	-10	4	4	A
56102020	Macaroni, whole wheat, cooked, fat added in cooking	1000_Grains	82	81	1	1	4	B
56133010	Spaghetti, cooked, whole wheat, fat added in cooking	1000_Grains	82	81	1	1	4	B
56102000	Macaroni, whole wheat, cooked, NS as to fat added in cooking	1000_Grains	82	79	3	1	4	A
56102010	Macaroni, whole wheat, cooked, fat not added in cooking	1000_Grains	82	79	3	1	4	A
56113000	Noodles, whole grain, cooked	1000_Grains	82	79	3	1	4	A
56113010	Noodles, cooked, whole wheat, fat not added in cooking	1000_Grains	82	79	3	1	4	A
56132990	Pasta, whole grain, cooked	1000_Grains	82	79	3	1	4	A
56133000	Spaghetti, cooked, whole wheat, fat not added in cooking	1000_Grains	82	79	3	1	4	A
57311700	Nu System Cuisine Toasted Grain Circles	1000_Grains	81	92	-11	4	4	C
56203056	Oatmeal, regular or quick, made with water, no added fat	1000_Grains	81	81	0	3	4	A
56204000	Quinoa, NS as to fat	1000_Grains	81	80	1	2	3.5	B
56204010	Quinoa, fat added	1000_Grains	81	80	1	2	3.5	B
57401100	Cereal, toasted oat	1000_Grains	80	91	-11	4	4	C
55601000	Flour and milk patty	1000_Grains	80	79	1	1	3.5	C
57000100	Cereal, oat, NFS	1000_Grains	79	89	-10	4	4.5	C
56200300	Cereal, cooked, NFS	1000_Grains	79	76	3	1	4	A
57000000	Cereal, NFS	1000_Grains	78	89	-11	4	4	C
56200510	Buckwheat groats, fat added	1000_Grains	78	78	0	2	4	B
56202980	Oatmeal, cooked, regular, NS as to fat added in cooking	1000_Grains	78	75	3	1	3.5	B
56203010	Oatmeal, cooked, regular, fat not added in cooking	1000_Grains	78	75	3	1	3.5	B
56207110	Bulgur, no added fat	1000_Grains	78	71	7	1	4	A
56207130	Bulgur, NS as to fat	1000_Grains	78	71	7	1	4	A
57123000	Cereal (General Mills Cheerios)	1000_Grains	77	89	-12	4	4.5	C
57306120	Malt-O-Meal Puffed Wheat	1000_Grains	77	86	-9	4	5	A
57416000	Cereal, puffed wheat, plain	1000_Grains	77	86	-9	4	5	A
56205205	Rice, wild, 100%, cooked, NS as to fat	1000_Grains	77	77	0	1	3.5	B
56205215	Rice, wild, 100%, cooked, fat added	1000_Grains	77	77	0	1	3.5	B
56207120	Bulgur, fat added	1000_Grains	77	76	1	2	4	B
56200490	Buckwheat groats, NS as to fat	1000_Grains	77	73	4	1	4	B
56200500	Buckwheat groats, no added fat	1000_Grains	77	73	4	1	4	B
57306700	Cereal (Malt-O-Meal Toasted Oat Cereal)	1000_Grains	75	89	-14	4	4.5	C
57206700	Cereal (General Mills Fiber One)	1000_Grains	75	82	-7	4	5	A
57101020	All-Bran with Extra Fiber	1000_Grains	75	78	-3	4	5	A
51201060	Bread, whole wheat, 100%, made from home recipe or purchased at bakery	1000_Grains	75	73	2	1	4	C

51201070	Bread, whole wheat, 100%, made from home recipe or purchased at bakery, toasted	1000_Grains	75	73	2	1	4	C
51300140	Bread, whole wheat, made from home recipe or purchased at bakery	1000_Grains	75	73	2	1	4	C
51300150	Bread, whole wheat, made from home recipe or purchased at bakery, toasted	1000_Grains	75	73	2	1	4	C
57305215	Cereal (Malt-O-Meal Frosted Mini Spooners)	1000_Grains	74	88	-14	4	4.5	B
56203510	Oatmeal, reduced sugar, plain, no added fat	1000_Grains	74	77	-3	3	3.5	C
56203555	Oatmeal, reduced sugar, flavored, no added fat	1000_Grains	74	77	-3	3	3.5	C
56203000	Oatmeal, NS as to regular, quick, or instant, no added fat	1000_Grains	74	74	0	3	4	A
57000050	Kashi cereal, NS as to ready to eat or cooked	1000_Grains	73	81	-8	4	5	A
56203076	Oatmeal, regular or quick, made with non-dairy milk, no added fat	1000_Grains	73	74	-1	3	3.5	B
56205210	Rice, wild, 100%, cooked, no added fat	1000_Grains	73	71	2	1	3.5	B
56205340	Rice, brown and wild, cooked, fat added	1000_Grains	73	71	2	1	3.5	B
56203200	Oatmeal with fruit, cooked	1000_Grains	73	69	4	1	3.5	B
57301500	Cereal (Kashi 7 Whole Grain Puffs)	1000_Grains	72	80	-8	4	5	A
56205012	Rice, brown, cooked, fat added, made with oil	1000_Grains	72	71	1	1	3.5	B
55105210	Pancakes, whole wheat, fat free	1000_Grains	72	66	6	1	3.5	C
56203050	Oatmeal, cooked, regular, fat added in cooking	1000_Grains	71	70	1	2	3.5	B
56203066	Oatmeal, regular or quick, made with milk, no added fat	1000_Grains	71	70	1	3	3.5	B
57417000	Cereal (Post Shredded Wheat)	1000_Grains	70	79	-9	4	5	A
57341000	Cereal (Post Shredded Wheat'n Bran)	1000_Grains	70	78	-8	4	5	A
56205016	Rice, brown, cooked, made with margarine	1000_Grains	70	68	2	1	3.5	B
56205510	Rice, brown, cooked, regular, fat added in cooking	1000_Grains	70	67	3	1	3.5	C
57214000	Cereal (Kellogg's Frosted Mini-Wheats)	1000_Grains	69	82	-13	4	4.5	B
57231250	Cereal (Post Great Grains Double Pecan Whole Grain Cereal)	1000_Grains	69	78	-9	4	4	C
51807000	Injera, Ethiopian bread	1000_Grains	69	76	-7	4	3.5	B
56203211	Oatmeal, cooked, regular, made with milk, fat not added in cooking	1000_Grains	69	65	4	1	3.5	B
56203231	Oatmeal, cooked, regular, made with milk, NS as to fat added in cooking	1000_Grains	69	65	4	1	3.5	B
56205018	Rice, brown, cooked, no added fat	1000_Grains	69	65	4	1	3.5	B
56205310	Rice, brown and wild, cooked, no added fat	1000_Grains	69	65	4	1	3.5	B
57347500	Strawberry Squares Mini-Wheats, Kellogg's (formerly Strawberry Squares)	1000_Grains	68	83	-15	4	4	B
56203086	Oatmeal, instant, plain, made with water, no added fat	1000_Grains	68	74	-6	4	4	A
56202100	Millet, fat added	1000_Grains	68	67	1	2	3.5	B
56203221	Oatmeal, cooked, regular, made with milk, fat added in cooking	1000_Grains	68	66	2	1	3.5	C
56205017	Rice, brown, cooked, fat added, NS as to fat type	1000_Grains	68	65	3	1	3.5	B
56205550	Rice, brown, cooked, instant, fat added in cooking	1000_Grains	68	65	3	1	3.5	C
56201990	Millet, NS as to fat	1000_Grains	68	64	4	1	3.5	B
56202000	Millet, no added fat	1000_Grains	68	64	4	1	3.5	B
56205011	Rice, brown, cooked, NS as to fat	1000_Grains	68	64	4	1	3.5	B

57406100	Cereal (General Mills Total)	1000_Grains	67	79	-12	4	4	C
57106050	Cereal (Post Great Grains Banana Nut Crunch)	1000_Grains	67	76	-9	4	4	C
55105000	Pancakes, buckwheat	1000_Grains	67	68	-1	3	3.5	C
56203075	Oatmeal, regular or quick, made with non-dairy milk, NS as to fat	1000_Grains	67	67	0	3	3.5	B
56203077	Oatmeal, regular or quick, made with non-dairy milk, fat added	1000_Grains	67	67	0	3	3.5	B
57208000	Cereal (Kellogg's All-Bran Complete Wheat Flakes)	1000_Grains	66	74	-8	4	3.5	D
55300060	French toast, from school, NFS [whole grain]	1000_Grains	66	69	-3	4	4	C
55301048	French toast sticks, from school, NFS [whole grain]	1000_Grains	66	69	-3	4	4	C
56117010	Long rice noodles (made from mung beans), cooked, fat added in cooking	1000_Grains	66	65	1	1	4	B
56202900	Oatmeal, from fast food, plain	1000_Grains	66	62	4	1	3.5	B
56205110	Rice, brown, cooked, regular, fat not added in cooking	1000_Grains	66	61	5	1	3.5	C
56205120	Rice, brown, cooked, regular, NS as to fat added in cooking	1000_Grains	66	61	5	1	3.5	C
56205530	Rice, brown, cooked, instant, NS as to fat added in cooking	1000_Grains	66	61	5	1	3.5	C
56205540	Rice, brown, cooked, instant, fat not added in cooking	1000_Grains	66	61	5	1	3.5	C
56203106	Oatmeal, instant, plain, made with non-dairy milk, no added fat	1000_Grains	65	73	-8	4	3.5	B
57231200	Cereal (Post Great Grains Raisins, Dates, and Pecans)	1000_Grains	65	73	-8	4	4	C
57308150	Mueslix cereal, NFS	1000_Grains	65	73	-8	4	4	C
57308190	Cereal, muesli	1000_Grains	65	73	-8	4	4	C
57411000	Cereal (General Mills Chex Wheat)	1000_Grains	65	73	-8	4	4	C
57301505	Cereal (Kashi Autumn Wheat)	1000_Grains	65	72	-7	4	5	A
57103050	Amaranth Flakes	1000_Grains	65	71	-6	4	5	A
56203055	Oatmeal, regular or quick, made with water, NS as to fat	1000_Grains	65	63	2	3	4	B
56203057	Oatmeal, regular or quick, made with water, fat added	1000_Grains	65	63	2	3	4	B
53710810	Cereal or granola bar (KIND Fruit and Nut Bar)	1000_Grains	64	73	-9	4	4	D
53713010	Cereal or granola bar, fruit and nut	1000_Grains	64	73	-9	4	4	D
56203220	Oatmeal, NS as to regular, quick, or instant, made with milk, fat added in cooking	1000_Grains	64	65	-1	3	3.5	C
56203210	Oatmeal, NS as to regular, quick, or instant, made with milk, fat not added in cooking	1000_Grains	64	63	1	3	3.5	B
56203230	Oatmeal, NS as to regular, quick, or instant, made with milk, NS as to fat added in cooking	1000_Grains	64	63	1	3	3.5	B
56202960	Oatmeal, NS as to regular, quick, or instant, NS as to fat	1000_Grains	64	62	2	3	4	B
56203040	Oatmeal, NS as to regular, quick, or instant, fat added	1000_Grains	64	62	2	3	4	B
51201020	Bread, whole wheat, 100%, toasted	1000_Grains	63	69	-6	4	4.5	A
56203520	Oatmeal, reduced sugar, plain, fat added	1000_Grains	63	63	0	3	3.5	C
56203560	Oatmeal, reduced sugar, flavored, fat added	1000_Grains	63	63	0	3	3.5	C
56203500	Oatmeal, reduced sugar, plain, NS as to fat	1000_Grains	63	62	1	3	3.5	C
56203550	Oatmeal, reduced sugar, flavored, NS as to fat	1000_Grains	63	62	1	3	3.5	C
56203065	Oatmeal, regular or quick, made with milk, NS as to fat	1000_Grains	63	61	2	3	3.5	C
56203067	Oatmeal, regular or quick, made with milk, fat added	1000_Grains	63	61	2	3	3.5	C

56208500	Oat bran cereal, cooked, no added fat	1000_Grains	63	52	11	1	4	A
57406105	Total Cranberry Crunch	1000_Grains	62	75	-13	4	3	D
57332050	Cereal (General Mills Total Raisin Bran)	1000_Grains	62	74	-12	4	3	D
57330000	Cereal (Kellogg's Raisin Bran)	1000_Grains	62	70	-8	4	3.5	D
51201120	Bread, whole wheat, 100%, with raisins, toasted	1000_Grains	62	67	-5	4	4.5	C
56207200	Whole wheat cereal, cooked, no added fat	1000_Grains	62	67	-5	4	3.5	A
56205350	Rice, brown and wild, cooked, NS as to fat	1000_Grains	62	59	3	1	3	C
58174000	Upma, Indian breakfast dish	1000_Grains	62	59	3	1	3.5	A
57143500	Cereal (Post Great Grains, Cranberry Almond Crunch)	1000_Grains	61	73	-12	4	4	C
57160000	Curves Fruit and Nut Crunch Cereal	1000_Grains	61	70	-9	4	3	D
57209000	Cereal (Post Bran Flakes)	1000_Grains	61	68	-7	4	3.5	C
71945020	Yam buns; Puerto Rican style	1000_Grains	61	59	2	2	4	C
56208530	Oat bran cereal, cooked, made with milk, fat not added in cooking	1000_Grains	61	56	5	1	3.5	C
57125010	Cereal (General Mills 25% Less Sugar Cinnamon Toast Crunch)	1000_Grains	60	76	-16	4	3	D
57206705	Cereal (General Mills Fiber One Caramel Delight)	1000_Grains	60	73	-13	4	3.5	D
57241200	Cereal (Post Shredded Wheat Honey Nut)	1000_Grains	60	71	-11	4	4	C
57228000	Granola, homemade	1000_Grains	60	70	-10	4	3	C
57418000	Cereal (General Mills Wheaties)	1000_Grains	60	70	-10	4	3	D
57321800	Optimum Slim, Nature's Path	1000_Grains	60	68	-8	4	3.5	C
57207000	Cereal, bran flakes	1000_Grains	60	67	-7	4	3.5	C
51202000	Muffin, English, whole wheat, 100%	1000_Grains	60	66	-6	4	4	B
51201110	Bread, whole wheat, 100%, with raisins	1000_Grains	60	65	-5	4	4	C
57410000	Cereal (Weetabix Whole Grain)	1000_Grains	60	65	-5	4	4.5	A
56205014	Rice, brown, cooked, made with butter	1000_Grains	60	56	4	1	3.5	C
57307500	Cereal, millet, puffed	1000_Grains	59	67	-8	4	4	A
55101020	Pancakes, plain, fat free	1000_Grains	59	64	-5	4	4.5	A
57101000	Cereal (Kellogg's All-Bran)	1000_Grains	59	62	-3	4	5	C
57103500	Apple Cinnamon Squares Mini-Wheats, Kellogg's (formerly Apple Cinnamon Squares)	1000_Grains	58	74	-16	4	4	C
57316385	Cereal (General Mills Cheerios Protein)	1000_Grains	58	74	-16	4	2.5	D
57241000	Cereal (General Mills Cheerios Honey Nut)	1000_Grains	58	73	-15	4	2.5	D
56203107	Oatmeal, instant, plain, made with non-dairy milk, fat added	1000_Grains	58	67	-9	4	3.5	B
57308900	Natural Muesli, Jenny's Cuisine	1000_Grains	58	65	-7	4	4.5	A
91781010	Snickers Marathon Protein bar	1000_Grains	58	65	-7	4	2.5	D
51201010	Bread, whole wheat, 100%	1000_Grains	58	64	-6	4	4	B
57102000	Cereal (Alpen)	1000_Grains	58	64	-6	4	4.5	A
57318000	100% Bran	1000_Grains	58	63	-5	4	4.5	C
41435300	Balance Original Bar	1000_Grains	57	75	-18	4	2.5	D
57308400	Cereal (General Mills Cheerios Multigrain)	1000_Grains	57	71	-14	4	3	C

57327500	Cereal (Quaker Oatmeal Squares)	1000_Grains	57	71	-14	4	4	C
57346500	Oatmeal Honey Nut Heaven, Quaker (formerly Toasted Oatmeal, Honey Nut)	1000_Grains	57	71	-14	4	2.5	D
57215000	Frosty O's	1000_Grains	57	70	-13	4	2.5	D
53544450	PowerBar (fortified high energy bar)	1000_Grains	57	69	-12	4	2.5	D
57304100	Cereal (Quaker Life)	1000_Grains	57	69	-12	4	3	D
57100100	Cereal, ready-to-eat, NFS	1000_Grains	57	67	-10	4	2.5	D
57206710	Cereal (General Mills Fiber One Honey Clusters)	1000_Grains	57	67	-10	4	4	C
57404100	Malt-O-Meal Toasty O's	1000_Grains	57	67	-10	4	3	D
57413000	Wheat germ, with sugar and honey	1000_Grains	57	67	-10	4	5	C
57206800	Cereal (Healt Valley Fiber 7 Flakes)	1000_Grains	57	66	-9	4	4.5	C
57231000	Cereal (Post Grape-Nuts Flakes)	1000_Grains	57	65	-8	4	4	C
51300110	Bread, whole wheat	1000_Grains	57	63	-6	4	4	B
56203096	Oatmeal, instant, plain, made with milk, no added fat	1000_Grains	57	63	-6	4	4	B
51122300	Bread, white, special formula, added fiber	1000_Grains	57	62	-5	4	4.5	A
51300050	Bread, whole grain white	1000_Grains	57	62	-5	4	4.5	A
51300120	Bread, whole wheat, toasted	1000_Grains	57	62	-5	4	4	B
56202970	Oatmeal, cooked, quick (1 or 3 minutes), NS as to fat added in cooking	1000_Grains	57	62	-5	4	3.5	B
56203020	Oatmeal, cooked, quick (1 or 3 minutes), fat not added in cooking	1000_Grains	57	62	-5	4	3.5	B
56207300	Whole wheat cereal, wheat and barley, cooked, fat not added in cooking	1000_Grains	57	62	-5	4	3.5	B
56207340	Whole wheat cereal, wheat and barley, cooked, NS as to fat added in cooking	1000_Grains	57	62	-5	4	3.5	B
51220000	Roll, whole wheat, 100%	1000_Grains	57	61	-4	4	4	C
56208510	Oat bran cereal, cooked, fat added	1000_Grains	57	49	8	1	4	A
56208520	Oat bran cereal, cooked, NS as to fat	1000_Grains	57	49	8	1	4	A
57305500	Cereal (Malt-O-Meal Honey Nut Toasty O's)	1000_Grains	56	73	-17	4	2.5	D
57321700	Optimum, Nature's Path	1000_Grains	56	72	-16	4	3.5	D
57206715	Cereal (General Mills Fiber One Raisin Bran Clusters)	1000_Grains	56	66	-10	4	4	C
56203030	Oatmeal, cooked, instant, fat not added in cooking	1000_Grains	56	64	-8	4	4	B
56203080	Oatmeal, cooked, instant, NS as to fat added in cooking	1000_Grains	56	64	-8	4	4	B
56207330	Whole wheat cereal, wheat and barley, cooked, fat added in cooking	1000_Grains	56	64	-8	4	3.5	B
51320550	Roll, whole wheat, hot dog bun	1000_Grains	56	62	-6	4	4	B
51320560	Roll, whole wheat, hamburger bun	1000_Grains	56	62	-6	4	4	B
51300060	Bread, whole grain white, toasted	1000_Grains	56	61	-5	4	4.5	B
51303100	Muffin, English, whole grain white	1000_Grains	56	61	-5	4	4	B
57213850	Cereal (General Mills Cheerios Frosted)	1000_Grains	55	70	-15	4	2.5	D
57221810	Cereal (General Mills Cheerios Fruity)	1000_Grains	55	70	-15	4	2.5	D
57128005	Cereal (General Mills 25% Less Sugar Cocoa Puffs)	1000_Grains	55	68	-13	4	2.5	D
57332100	Cereal (General Mills Raisin Nut Bran)	1000_Grains	55	67	-12	4	3	D

53720510	Snickers Marathon Energy bar	1000_Grains	55	66	-11	4	2	D
53720300	Nutrition bar (PowerBar)	1000_Grains	55	65	-10	4	2.5	D
57214100	Frosted Wheat Bites	1000_Grains	55	64	-9	4	4	B
57301530	Cereal (Kashi Heart to Heart Honey Toasted Oat)	1000_Grains	55	64	-9	4	4.5	C
57331000	Cereal (Post Raisin Bran)	1000_Grains	55	62	-7	4	3	D
51201150	Bread, pita, whole wheat, 100%	1000_Grains	55	60	-5	4	4.5	A
51601020	Bread, multigrain	1000_Grains	55	60	-5	4	4.5	A
52215260	Tortilla, whole wheat	1000_Grains	55	60	-5	4	3	C
57219000	Cereal, fruit and fiber	1000_Grains	55	60	-5	4	4	C
57221000	Cereal, fiber and fruit	1000_Grains	55	60	-5	4	4	C
53540500	Breakfast bar, date, with yogurt coating	1000_Grains	55	59	-4	4	4.5	C
56207360	Wheat cereal, chocolate flavored, cooked, fat not added in cooking	1000_Grains	55	59	-4	4	3.5	C
56207370	Wheat cereal, chocolate flavored, cooked	1000_Grains	55	59	-4	4	3.5	C
51320040	Roll, wheat or cracked wheat, made from home recipe or purchased at bakery	1000_Grains	55	51	4	1	3.5	D
55702100	Dosa (Indian), plain	1000_Grains	55	51	4	1	3.5	C
57103100	Cereal (General Mills Cheerios Apple Cinnamon)	1000_Grains	54	69	-15	4	2.5	D
57237200	Cereal (Post Honey Bunches of Oats with Vanilla Bunches)	1000_Grains	54	68	-14	4	4	C
53720100	Nutrition bar (Balance Original Bar)	1000_Grains	54	66	-12	4	2	D
57319000	100% Natural Cereal, plain, Quaker	1000_Grains	54	64	-10	4	4	C
56203105	Oatmeal, instant, plain, made with non-dairy milk, NS as to fat	1000_Grains	54	62	-8	4	3.5	B
57206000	Cereal (Famila)	1000_Grains	54	61	-7	4	4	B
56203087	Oatmeal, instant, plain, made with water, fat added	1000_Grains	54	60	-6	4	3.5	B
51201160	Bread, pita, whole wheat, 100%, toasted	1000_Grains	54	59	-5	4	4	A
51301600	Bread, pita, whole wheat	1000_Grains	54	59	-5	4	4	B
51301620	Bread, pita, wheat or cracked wheat	1000_Grains	54	59	-5	4	4	B
51601010	Bread, multigrain, toasted	1000_Grains	54	59	-5	4	4.5	A
57106260	Cereal (General Mills Cheerios Berry Burst)	1000_Grains	53	68	-15	4	2.5	D
53720510	Snickers Marathon Energy bar	1000_Grains	53	67	-14	4	2	D
57316380	Cereal (General Mills Cheerios Oat Cluster Crunch)	1000_Grains	53	67	-14	4	2.5	D
56203175	Oatmeal, instant, other flavors, no added fat	1000_Grains	53	63	-10	4	3.5	C
56203070	Oatmeal, cooked, instant, fat added in cooking	1000_Grains	53	60	-7	4	3.5	C
57138000	Total Corn Flakes	1000_Grains	53	60	-7	4	2.5	D
51301610	Bread, pita, whole wheat, toasted	1000_Grains	53	59	-6	4	4	A
51301630	Bread, pita, wheat or cracked wheat, toasted	1000_Grains	53	59	-6	4	4	A
53540802	Kashi TLC Chewy Granola Bar	1000_Grains	53	58	-5	4	3.5	C
51601210	Bread, multigrain, with raisins	1000_Grains	53	57	-4	4	4	B
56103020	Macaroni, cooked, spinach, fat added in cooking	1000_Grains	53	48	5	1	3.5	B
55301025	French toast, gluten free	1000_Grains	53	47	6	3	3	D

57232100	Healthy Choice Almond Crunch with raisins, Kellogg's	1000_Grains	52	70	-18	4	3	D
57404200	Malt-O-Meal Apple and Cinnamon Toasty O's	1000_Grains	52	65	-13	4	2	E
53720500	Nutrition bar (Snickers Marathon Protein Bar)	1000_Grains	52	64	-12	4	2.5	D
56203130	Oatmeal, instant, maple flavored, no added fat	1000_Grains	52	62	-10	4	3.5	C
56203155	Oatmeal, instant, fruit flavored, no added fat	1000_Grains	52	61	-9	4	3.5	C
57110000	Cereal (Kellogg's All-Bran Bran Buds)	1000_Grains	52	60	-8	4	3.5	D
56203060	Oatmeal, cooked, quick (1 or 3 minutes), fat added in cooking	1000_Grains	52	58	-6	4	3.5	B
57231100	Grape-Nuts Trail Mix Crunch	1000_Grains	52	58	-6	4	4	C
51601220	Bread, multigrain, with raisins, toasted	1000_Grains	52	57	-5	4	4	A
53710802	Cereal or granola bar (Kashi Crunchy)	1000_Grains	52	57	-5	4	3.5	C
57137000	Cereal, corn puffs	1000_Grains	52	56	-4	4	3.5	C
56203610	Oatmeal, multigrain, no added fat	1000_Grains	52	54	-2	4	4	A
55205000	Waffle, whole grain	1000_Grains	52	52	0	3	2	E
56114020	Noodles, cooked, spinach, fat added in cooking	1000_Grains	52	46	6	1	4	A
57335550	Cereal (General Mills Reese's Puffs)	1000_Grains	51	69	-18	4	1.5	E
53540404	Kellogg's Nutri-Grain Fruit and Nut Bar	1000_Grains	51	59	-8	4	2.5	C
57152000	Crispy Wheats'n Raisins	1000_Grains	51	58	-7	4	2.5	D
56203095	Oatmeal, instant, plain, made with milk, NS as to fat	1000_Grains	51	56	-5	4	3.5	C
56203097	Oatmeal, instant, plain, made with milk, fat added	1000_Grains	51	56	-5	4	3.5	C
57230000	Cereal (Post Grape-Nuts)	1000_Grains	51	54	-3	4	4.5	C
57316200	Cereal, nutty nuggets	1000_Grains	51	54	-3	4	4.5	C
55105200	Pancakes, whole grain	1000_Grains	51	48	3	3	2	D
56116990	Long rice noodles, made from mung beans, cooked	1000_Grains	51	45	6	1	4	A
56117000	Long rice noodles (made from mung beans), cooked, fat not added in cooking	1000_Grains	51	45	6	1	4	A
57124100	Cereal (General Mills Cheerios Chocolate)	1000_Grains	50	68	-18	4	2	D
53720700	Nutrition bar (Tiger's Milk)	1000_Grains	50	66	-16	4	2	D
41435110	High protein bar, candy-like, soy and milk base	1000_Grains	50	61	-11	4	2	D
57103000	Cereal (Post Alpha-Bits)	1000_Grains	50	61	-11	4	2.5	D
53720610	Nutrition bar (South Beach Living High Protein Bar)	1000_Grains	50	60	-10	4	2.5	D
53710504	Cereal or granola bar (Kellogg's Nutri-Grain Fruit and Nut Bar)	1000_Grains	50	59	-9	4	2.5	D
57301511	Cereal (Kashi GOLEAN Crunch)	1000_Grains	50	59	-9	4	4.5	C
57303100	Cereal (General Mills Kix)	1000_Grains	50	56	-6	4	3.5	C
51208100	Bagel, whole wheat, 100%, with raisins	1000_Grains	50	53	-3	4	3.5	C
57301510	Cereal (Kashi GOLEAN)	1000_Grains	50	50	0	4	5	A
56207086	Wheat, cream of, cooked, regular, made with milk, fat added in cooking	1000_Grains	50	45	5	1	3.5	C
56201700	Cornstarch with milk, eaten as a cereal (2 tbsp cornstarch in 2-1/2 cups milk)	1000_Grains	50	44	6	1	3.5	B
56207087	Wheat, cream of, cooked, regular, made with milk, fat not added in cooking	1000_Grains	50	44	6	1	3.5	C

55301015	French toast, whole grain	1000_Grains	50	43	7	3	3	D
55301020	French toast, whole grain, reduced fat	1000_Grains	50	43	7	3	3.5	C
55301055	French toast sticks, whole grain	1000_Grains	50	43	7	3	3	D
56104020	Macaroni, cooked, vegetable, fat added in cooking	1000_Grains	50	43	7	1	4	A
57125000	Cereal (General Mills Cinnamon Toast Crunch)	1000_Grains	49	64	-15	4	2.5	D
53540806	Kashi TLC Crunchy Granola Bar	1000_Grains	49	61	-12	4	3	C
57301512	Cereal (Kashi GOLEAN Crunch Honey Almond Flax)	1000_Grains	49	61	-12	4	4.5	C
57344025	Cereal (Kellogg's Special K Cinnamon Pecan)	1000_Grains	49	59	-10	4	3	D
41435120	Zone Perfect Classic Crunch nutrition bar	1000_Grains	49	58	-9	4	1.5	E
57128880	Complete Oat Bran Flakes, Kellogg's (formerly Common Sense Oat Bran, plain)	1000_Grains	49	55	-6	4	3	D
56207212	Whole wheat cereal, cooked, made with milk	1000_Grains	49	54	-5	4	3.5	B
51208000	Bagel, whole wheat, 100%	1000_Grains	49	53	-4	4	4	C
51208010	Bagel, whole wheat, 100%, toasted	1000_Grains	49	53	-4	4	3.5	C
51208110	Bagel, whole wheat, 100%, with raisins, toasted	1000_Grains	49	53	-4	4	3.5	C
51300100	Bagel, whole grain white	1000_Grains	49	53	-4	4	4	C
56207190	Whole wheat cereal, cooked, NS as to fat	1000_Grains	49	53	-4	4	3.5	B
56207210	Whole wheat cereal, cooked, fat added	1000_Grains	49	53	-4	4	3.5	B
56207026	Cream of wheat, regular or quick, made with non-dairy milk, no added fat	1000_Grains	49	46	3	3	3.5	B
55105205	Pancakes, whole grain, reduced fat	1000_Grains	49	45	4	3	2.5	D
57124300	Cereal (General Mills Lucky Charms Chocolate)	1000_Grains	48	66	-18	4	2	D
53540806	Kashi TLC Crunchy Granola Bar	1000_Grains	48	60	-12	4	3	C
57344007	Cereal (Kellogg's Special K Low Fat Granola)	1000_Grains	48	58	-10	4	3	D
53729000	Nutrition bar or meal replacement bar, NFS	1000_Grains	48	57	-9	4	1.5	E
53720800	Nutrition bar (Zone Perfect Classic Crunch)	1000_Grains	48	56	-8	4	1.5	E
55200060	Waffle, whole grain, from frozen	1000_Grains	48	56	-8	4	3.5	C
55200080	Waffle, whole grain, fruit, from frozen	1000_Grains	48	56	-8	4	3.5	C
57227000	Cereal, granola	1000_Grains	48	56	-8	4	4	C
57309100	Cereal (Nature Valley Granola)	1000_Grains	48	56	-8	4	4	C
56203222	Oatmeal, cooked, quick (1 or 3 minutes), made with milk, fat added in cooking	1000_Grains	48	54	-6	4	3.5	C
56203223	Oatmeal, cooked, instant, made with milk, fat added in cooking	1000_Grains	48	54	-6	4	3.5	C
56203212	Oatmeal, cooked, quick (1 or 3 minutes), made with milk, fat not added in cooking	1000_Grains	48	52	-4	4	3.5	B
56203232	Oatmeal, cooked, quick (1 or 3 minutes), made with milk, NS as to fat added in cooking	1000_Grains	48	52	-4	4	3.5	B
56112030	Noodles, cooked, fat added in cooking	1000_Grains	48	41	7	1	3.5	B
56207040	Wheat, cream of, cooked, made with milk	1000_Grains	48	41	7	1	3.5	C
57106060	Cereal (General Mills Cheerios Banana Nut)	1000_Grains	47	60	-13	4	2.5	D
57307010	Cereal (Post Maple Pecan Crunch)	1000_Grains	47	59	-12	4	3.5	C
57229500	Cereal (Kellogg's Low Fat Granola with Raisins)	1000_Grains	47	57	-10	4	3	D

57237300	Cereal (Post Honey Bunches of Oats with Almonds)	1000_Grains	47	57	-10	4	2.5	D
57301540	Cereal (Kashi Honey Sunshine Squares)	1000_Grains	47	53	-6	4	4	C
57329000	Cereal, raisin bran	1000_Grains	47	53	-6	4	3	D
56203213	Oatmeal, cooked, instant, made with milk, fat not added in cooking	1000_Grains	47	51	-4	4	3.5	C
56203233	Oatmeal, cooked, instant, made with milk, NS as to fat added in cooking	1000_Grains	47	51	-4	4	3.5	C
51300210	Bread, whole wheat, with raisins	1000_Grains	47	50	-3	4	3	D
56203600	Oatmeal, multigrain, NS as to fat	1000_Grains	47	49	-2	4	4	B
56203620	Oatmeal, multigrain, fat added	1000_Grains	47	49	-2	4	4	B
57130000	Cereal (General Mills Cookie Crisp)	1000_Grains	46	60	-14	4	2	D
57201900	Cereal (General Mills Dora The Explorer)	1000_Grains	46	57	-11	4	3	D
57229000	Cereal (Kellogg's Low Fat Granola)	1000_Grains	46	56	-10	4	4	C
57320500	Cereal (Quaker Granola with Oats, Honey, and Raisins)	1000_Grains	46	56	-10	4	4	C
51300180	Bread, puri, wheat	1000_Grains	46	55	-9	4	3.5	C
55200130	Waffle, whole grain, from fast food / restaurant	1000_Grains	46	55	-9	4	1.5	E
55200200	Waffle, from school, NFS	1000_Grains	46	54	-8	4	3.5	C
51300220	Bread, whole wheat, with raisins, toasted	1000_Grains	46	50	-4	4	2.5	D
56101030	Macaroni, cooked, fat added in cooking	1000_Grains	46	40	6	1	3.5	B
56131000	Spaghetti, cooked, fat added in cooking	1000_Grains	46	40	6	1	3.5	B
56113990	Noodles, vegetable, cooked	1000_Grains	46	36	10	1	4	B
57324000	Peanut Butter Toast Crunch, General Mills	1000_Grains	45	63	-18	4	1.5	E
57128000	Cereal (General Mills Cocoa Puffs)	1000_Grains	45	62	-17	4	2	E
53541300	Slim Fast Original Meal Bar	1000_Grains	45	57	-12	4	2	D
57327450	Cereal (Quaker Toasted Oat Bran)	1000_Grains	45	56	-11	4	4	C
55200070	Waffle, whole grain, reduced fat, from frozen	1000_Grains	45	53	-8	4	3.5	C
51320720	Roll, whole grain white, hamburger bun	1000_Grains	45	51	-6	4	3	D
57319500	Sun Country 100% Natural Granola, with Almonds	1000_Grains	45	51	-6	4	3.5	D
51320700	Roll, whole grain white	1000_Grains	45	50	-5	4	3	D
51320710	Roll, whole grain white, hot dog bun	1000_Grains	45	50	-5	4	3	D
51806010	Bread, rice	1000_Grains	45	49	-4	4	3.5	C
51806020	Bread, rice, toasted	1000_Grains	45	49	-4	4	3	C
51808000	Bread, gluten free	1000_Grains	45	49	-4	4	3.5	C
51808010	Bread, gluten free, toasted	1000_Grains	45	49	-4	4	3	C
56207080	Wheat, cream of, cooked, NS as to regular, quick, or instant, fat added in cooking	1000_Grains	45	44	1	3	3.5	B
55202000	Waffle, wheat, bran, or multigrain	1000_Grains	45	41	4	1	2.5	D
56207180	Couscous, plain, cooked, fat added in cooking	1000_Grains	45	40	5	1	3.5	B
56201072	Grits, cooked, corn or hominy, with cheese, regular, fat added in cooking	1000_Grains	45	39	6	3	3	C
57107000	Cereal (General Mills Boo Berry)	1000_Grains	44	59	-15	4	2.5	D
57211000	Cereal (General Mills Frankenberry)	1000_Grains	44	58	-14	4	2.5	D

57124050	Cereal (General Mills Chex Cinnamon)	1000_Grains	44	56	-12	4	2	E
57316450	Cereal (General Mills Oatmeal Crisp with Almonds)	1000_Grains	44	56	-12	4	4	C
53720600	Nutrition bar (South Beach Living Meal Bar)	1000_Grains	44	55	-11	4	2	D
57321905	Organic Flax Plus, Pumpkin Granola, Nature's Path	1000_Grains	44	55	-11	4	4	C
57303105	Cereal (General Mills Honey Kix)	1000_Grains	44	54	-10	4	3	D
56207027	Cream of wheat, regular or quick, made with non-dairy milk, fat added	1000_Grains	44	41	3	3	3.5	B
56201066	Grits, regular or quick, made with non-dairy milk, no added fat	1000_Grains	44	40	4	3	3.5	B
56207025	Cream of wheat, regular or quick, made with non-dairy milk, NS as to fat	1000_Grains	44	40	4	3	3.5	B
56207220	Wheat, cream of, cooked, regular, fat added in cooking	1000_Grains	44	40	4	2	3.5	B
56207022	Cream of wheat, regular or quick, made with milk, no added fat	1000_Grains	44	39	5	3	3.5	B
75217500	Hominy, cooked, fat not added in cooking	1000_Grains	44	36	8	3	4	B
57106250	Cereal (General Mills Kix Berry Berry)	1000_Grains	43	56	-13	4	2.5	D
53720400	Nutrition bar (Slim Fast Original Meal Bar)	1000_Grains	43	55	-12	4	2	D
41435710	South Beach Living Meal Replacement Bar	1000_Grains	43	53	-10	4	1.5	E
55100065	Pancakes, whole grain, from fast food / restaurant	1000_Grains	43	51	-8	4	2	D
56207021	Cream of wheat, regular or quick, made with milk, NS as to fat	1000_Grains	43	39	4	3	3.5	B
56207023	Cream of wheat, regular or quick, made with milk, fat added	1000_Grains	43	38	5	3	3.5	B
56201070	Grits, cooked, corn or hominy, with cheese, regular, NS as to fat added in cooking	1000_Grains	43	36	7	3	3	C
56201071	Grits, cooked, corn or hominy, with cheese, regular, fat not added in cooking	1000_Grains	43	36	7	3	3	C
56201320	Grits, cooked, corn or hominy, regular, made with milk, fat added in cooking	1000_Grains	43	36	7	1	3.5	C
56201324	Grits, cooked, corn or hominy, regular, made with milk, NS as to fat added in cooking	1000_Grains	43	36	7	1	3.5	C
56201322	Grits, cooked, corn or hominy, regular, made with milk, fat not added in cooking	1000_Grains	43	35	8	1	3.5	C
57305165	Cereal (Malt-O-Meal Cinnamon Toasters)	1000_Grains	42	60	-18	4	2	D
57419000	Cereal (General Mills Cheerios Yogurt Burst)	1000_Grains	42	59	-17	4	2.5	D
57139000	Cereal (General Mills Count Chocula)	1000_Grains	42	58	-16	4	2	D
57305100	Cereal (General Mills Lucky Charms)	1000_Grains	42	58	-16	4	2	E
41435700	South Beach Living High Protein Cereal Bar	1000_Grains	42	53	-11	4	2.5	D
57341200	Cereal (Kellogg's Smart Start Strong)	1000_Grains	42	52	-10	4	2.5	D
53540300	Fiber One Chewy Bar	1000_Grains	42	50	-8	4	3.5	D
53541200	Meal replacement bar	1000_Grains	42	50	-8	4	1.5	E
56203125	Oatmeal, instant, maple flavored, NS as to fat	1000_Grains	42	50	-8	4	3.5	C
56203135	Oatmeal, instant, maple flavored, fat added	1000_Grains	42	50	-8	4	3.5	C
56203180	Oatmeal, instant, other flavors, fat added	1000_Grains	42	50	-8	4	3.5	C
57321900	Cereal (Nature's Path Organic Flax Plus)	1000_Grains	42	50	-8	4	4.5	C
56203150	Oatmeal, instant, fruit flavored, NS as to fat	1000_Grains	42	49	-7	4	3.5	C
56203160	Oatmeal, instant, fruit flavored, fat added	1000_Grains	42	49	-7	4	3.5	C

56203170	Oatmeal, instant, other flavors, NS as to fat	1000_Grains	42	49	-7	4	3.5	C
55100040	Pancakes, gluten free, from frozen	1000_Grains	42	48	-6	4	3.5	C
55106000	Pancakes, gluten free	1000_Grains	42	48	-6	4	3.5	C
57306130	Cereal (Malt-O-Meal Raisin Bran)	1000_Grains	42	48	-6	4	2.5	D
56207350	Wheat cereal, chocolate flavored, cooked, made with milk	1000_Grains	42	45	-3	4	3	C
56201062	Grits, cooked, corn or hominy, with cheese, NS as to regular, quick, or instant, fat added in cooking	1000_Grains	42	39	3	3	3	C
56112000	Noodles, cooked	1000_Grains	42	33	9	1	3.5	B
56112010	Noodles, cooked, fat not added in cooking	1000_Grains	42	33	9	1	3.5	B
56103000	Macaroni, cooked, spinach, NS as to fat added in cooking	1000_Grains	42	32	10	1	4	A
56103010	Macaroni, cooked, spinach, fat not added in cooking	1000_Grains	42	32	10	1	4	A
56114000	Noodles, cooked, spinach, fat not added in cooking	1000_Grains	42	32	10	1	4	B
57237100	Cereal (Post Honey Bunches of Oats Honey Roasted)	1000_Grains	41	53	-12	4	2.5	D
57237900	Cereal (Post Honey Bunches of Oats Just Bunches)	1000_Grains	41	53	-12	4	2.5	D
57344015	Cereal (Kellogg's Special K Fruit & Yogurt)	1000_Grains	41	52	-11	4	2.5	D
57321500	100 % Natural Wholegrain Cereal with raisins, lowfat, Quaker	1000_Grains	41	50	-9	4	4	C
52303010	Muffin, whole wheat	1000_Grains	41	49	-8	4	2	D
52406010	Bread, whole wheat, with nuts	1000_Grains	41	49	-8	4	2.5	D
53544300	Granola bar, high fiber, coated with non-chocolate yogurt coating	1000_Grains	41	46	-5	4	4	C
55100030	Pancakes, whole grain, from frozen	1000_Grains	41	46	-5	4	3	C
55100035	Pancakes, whole grain, reduced fat, from frozen	1000_Grains	41	46	-5	4	3	C
53714300	Cereal or granola bar, high fiber, coated with non-chocolate yogurt coating	1000_Grains	41	45	-4	4	3.5	D
51300175	Bread, chappatti or roti, wheat	1000_Grains	41	42	-1	4	4	B
53430000	Crepe, NS as to filling	1000_Grains	41	36	5	2	3	C
56201055	Grits, regular or quick, made with milk, NS as to fat	1000_Grains	41	36	5	3	3.5	C
53430200	Crepe, fruit filled	1000_Grains	41	35	6	2	3	C
55501000	Chinese pancake	1000_Grains	41	34	7	1	3.5	B
57212100	French Toast Crunch, General Mills	1000_Grains	40	53	-13	4	1.5	E
53542200	Granola bar, lowfat, NFS	1000_Grains	40	47	-7	4	2.5	D
51123010	Bread, high protein	1000_Grains	40	43	-3	4	4	C
51301540	Bread, French or Vienna, whole wheat	1000_Grains	40	42	-2	4	4	B
56201065	Grits, regular or quick, made with non-dairy milk, NS as to fat	1000_Grains	40	36	4	3	3.5	C
56201067	Grits, regular or quick, made with non-dairy milk, fat added	1000_Grains	40	36	4	3	3.5	C
58117110	Cornmeal fritter, Puerto Rican style	1000_Grains	40	36	4	2	2	D
56201020	Grits, cooked, corn or hominy, regular, fat added in cooking	1000_Grains	40	35	5	2	3.5	B
56201057	Grits, regular or quick, made with milk, fat added	1000_Grains	40	35	5	3	3.5	C
56201056	Grits, regular or quick, made with milk, no added fat	1000_Grains	40	34	6	3	3.5	C
51301040	Bread, wheat or cracked wheat, made from home recipe or purchased at bakery	1000_Grains	40	33	7	1	3.5	C
56205094	Rice, cream of, cooked, made with milk	1000_Grains	40	33	7	1	3.5	C

56205440	Rice, white, cooked, converted, fat added in cooking	1000_Grains	40	33	7	1	3	C
71930200	Casabe, cassava bread	1000_Grains	40	30	10	1	3	D
56104000	Pasta, vegetable, cooked	1000_Grains	40	28	12	1	4	A
56104010	Macaroni, cooked, vegetable, fat not added in cooking	1000_Grains	40	28	12	1	4	A
57124030	Cereal (General Mills Chex Chocolate)	1000_Grains	39	51	-12	4	1.5	E
57237310	Cereal (Post Honey Bunches of Oats wth Pecan Bunches)	1000_Grains	39	51	-12	4	2.5	D
53712210	Cereal or granola bar, nonfat	1000_Grains	39	49	-10	4	2.5	D
57103020	Alpha-bits with marshmallows	1000_Grains	39	49	-10	4	2	D
57305150	Cereal, frosted oat cereal with marshmallows	1000_Grains	39	49	-10	4	2	D
53720200	Nutrition bar (Clif Bar)	1000_Grains	39	48	-9	4	3	D
53543100	Granola bar, peanuts, oats, sugar, wheat germ	1000_Grains	39	47	-8	4	2	D
57143000	Cereal (Kellogg's Cracklin' Oat Bran)	1000_Grains	39	47	-8	4	2.5	D
53710400	Cereal or granola bar (General Mills Fiber One Chewy Bar)	1000_Grains	39	45	-6	4	3.5	D
57132000	Cereal (General Mills Chex Corn)	1000_Grains	39	45	-6	4	2.5	D
57111000	Bran Chex	1000_Grains	39	44	-5	4	3	D
57308300	Multi Bran Chex	1000_Grains	39	44	-5	4	3	D
51123020	Bread, high protein, toasted	1000_Grains	39	42	-3	4	4	B
51301550	Bread, French or Vienna, whole wheat, toasted	1000_Grains	39	41	-2	4	4	C
56203540	Oatmeal, made with milk and sugar, Puerto Rican style	1000_Grains	39	37	2	1	3	C
56201298	Grits, cooked, corn or hominy, NS as to regular, quick, or instant, made with milk, fat not added in cooking	1000_Grains	39	34	5	3	3.5	C
56201300	Grits, cooked, corn or hominy, NS as to regular, quick, or instant, made with milk, NS as to fat added in cooking	1000_Grains	39	34	5	3	3.5	C
58117210	Cornmeal stick, Puerto Rican style	1000_Grains	39	34	5	2	2.5	C
55204000	Waffle, cornmeal	1000_Grains	39	33	6	2	3	D
51301050	Bread, wheat or cracked wheat, made from home recipe or purchased at bakery, toasted	1000_Grains	39	32	7	1	3.5	D
57106100	Cereal (General Mills Basic 4)	1000_Grains	38	50	-12	4	3	D
57344020	Cereal (Kellogg's Special K Vanilla Almond)	1000_Grains	38	49	-11	4	2.5	D
55200090	Waffle, gluten free, from frozen	1000_Grains	38	47	-9	4	3	C
55208000	Waffle, gluten free	1000_Grains	38	47	-9	4	3	C
57301535	Cereal (Kashi Heart to Heart Oat Flakes and Blueberry Clusters)	1000_Grains	38	46	-8	4	4	C
57124000	Chex cereal, NFS	1000_Grains	38	43	-5	4	2.5	D
57301520	Cereal (Kashi Good Friends)	1000_Grains	38	40	-2	4	5	B
55300055	French toast, whole grain, from fast food / restaurant	1000_Grains	38	39	-1	4	2.5	D
55103000	Pancakes, with fruit	1000_Grains	38	35	3	3	3	C
56205410	Rice, white, cooked with fat, Puerto Rican style	1000_Grains	38	32	6	1	3	C
56140100	Pasta, gluten free	1000_Grains	38	28	10	1	3.5	B
55400010	Crepe, NFS	1000_Grains	38	27	11	2	3	C
56200390	Barley, NS as to fat	1000_Grains	38	27	11	1	4	B
56200400	Barley, no added fat	1000_Grains	38	27	11	1	4	B

53710500	Cereal or granola bar (Kellogg's Nutri-Grain Cereal Bar)	1000_Grains	37	50	-13	4	2	D
41435500	Clif Bar	1000_Grains	37	48	-11	4	3	D
53540402	Kellogg's Nutri-Grain Yogurt Bar	1000_Grains	37	48	-11	4	2	D
57316300	Cereal (Health Valley Oat Bran Flakes)	1000_Grains	37	47	-10	4	3	C
57244000	Just Right	1000_Grains	37	43	-6	4	2.5	D
51102010	Bread, white with whole wheat swirl	1000_Grains	37	41	-4	4	4	C
51401060	Bread, rye, reduced calorie and/or high fiber	1000_Grains	37	36	1	4	5	A
51501060	Bread, oat bran, reduced calorie and/or high fiber	1000_Grains	37	36	1	4	5	A
56205002	Rice, white, cooked, made with oil	1000_Grains	37	30	7	1	3.5	B
55401000	Crepe, plain	1000_Grains	37	27	10	2	3	C
53540902	Nature Valley Chewy Granola Bar with Yogurt Coating	1000_Grains	36	48	-12	4	2	D
57316410	Oatmeal Crisp, Apple Cinnamon (formerly Oatmeal Crisp with Apples)	1000_Grains	36	48	-12	4	2.5	D
53540804	Kashi GOLEAN Crunchy Bars	1000_Grains	36	47	-11	4	2	E
57344001	Cereal (Kellogg's Special K Blueberry)	1000_Grains	36	45	-9	4	3	D
57336000	Cereal (General Mills Chex Rice)	1000_Grains	36	43	-7	4	2.5	D
57301100	Kaboom	1000_Grains	36	42	-6	4	2	D
51401070	Bread, rye, reduced calorie and/or high fiber, toasted	1000_Grains	36	35	1	4	5	A
51501070	Bread, oat bran, reduced calorie and/or high fiber, toasted	1000_Grains	36	35	1	4	5	A
55103020	Pancakes, pumpkin	1000_Grains	36	33	3	3	3	C
55203700	Waffle, cinnamon	1000_Grains	36	33	3	3	2	D
56201060	Grits, cooked, corn or hominy, with cheese, NS as to regular, quick, or instant, NS as to fat added in cooking	1000_Grains	36	30	6	3	3.5	C
75217490	Hominy, cooked, NS as to fat added in cooking	1000_Grains	36	28	8	3	3.5	C
75217520	Hominy, cooked	1000_Grains	36	28	8	3	3.5	C
53710502	Cereal or granola bar (Kellogg's Nutri-Grain Yogurt Bar)	1000_Grains	35	50	-15	4	2	D
53540400	Kellogg's Nutri-Grain Cereal Bar	1000_Grains	35	48	-13	4	2	D
53542210	Granola bar, nonfat	1000_Grains	35	47	-12	4	2.5	D
57407110	Cereal (General Mills 25% Less Sugar Trix)	1000_Grains	35	47	-12	4	2.5	D
57316500	Cereal (General Mills Oatmeal Crisp with Raisins)	1000_Grains	35	45	-10	4	3.5	C
57330010	Cereal (Kellogg's Raisin Bran Crunch)	1000_Grains	35	45	-10	4	2.5	D
53540804	Kashi GOLEAN Crunchy Bars	1000_Grains	35	42	-7	4	2	E
53713100	Cereal or granola bar, peanuts , oats, sugar, wheat germ	1000_Grains	35	41	-6	4	2	D
55200120	Waffle, fruit, from fast food / restaurant	1000_Grains	35	41	-6	4	2	D
53710906	Cereal or granola bar (General Mills Nature Valley Crunchy Granola Bar)	1000_Grains	35	40	-5	4	2	D
53712100	Cereal or Granola bar, NFS	1000_Grains	35	40	-5	4	2	D
51320530	Roll, whole wheat, NS as to 100%, made from home recipe or purchased at bakery	1000_Grains	35	39	-4	4	3.5	D
55212000	Waffle, whole grain, reduced fat	1000_Grains	35	38	-3	4	2	E
56201061	Grits, cooked, corn or hominy, with cheese, NS as to regular, quick, or instant, fat not added in cooking	1000_Grains	35	29	6	3	3.5	C

56201090	Grits, with cheese, NS as to fat	1000_Grains	35	29	6	2	3.5	C
56201092	Grits, with cheese, fat added	1000_Grains	35	29	6	2	3.5	C
53400300	Blintz, fruit-filled	1000_Grains	35	28	7	2	3	C
56201091	Grits, with cheese, no added fat	1000_Grains	35	27	8	2	3.5	C
56201530	Cornmeal mush, made with milk	1000_Grains	35	27	8	1	3.5	C
56205320	Rice, white and wild, cooked, fat added	1000_Grains	35	27	8	1	3	C
56205330	Rice, white and wild, cooked, NS as to fat	1000_Grains	35	27	8	1	3	C
56101000	Macaroni, cooked, NS as to fat added in cooking	1000_Grains	35	25	10	1	3.5	B
56101010	Macaroni, cooked, fat not added in cooking	1000_Grains	35	25	10	1	3.5	B
56130000	Pasta, cooked	1000_Grains	35	25	10	1	3.5	B
56130010	Spaghetti, cooked, fat not added in cooking	1000_Grains	35	25	10	1	3.5	B
56132000	Spaghetti, cooked, high protein type (assume no fat added)	1000_Grains	35	25	10	1	3.5	B
56200410	Barley, fat added	1000_Grains	35	25	10	1	3.5	C
57305600	Cereal (Malt-O-Meal Marshmallow Mateys)	1000_Grains	34	49	-15	4	1.5	E
53710902	Cereal or granola bar, with yogurt coating (General Mills Nature Valley Chewy Granola Bar)	1000_Grains	34	48	-14	4	2	D
53710900	Cereal or granola bar (General Mills Nature Valley Chewy Trail Mix)	1000_Grains	34	46	-12	4	2	E
57224000	Cereal (General Mills Golden Grahams)	1000_Grains	34	46	-12	4	2	E
57305400	Cereal (Malt-O-Meal Honey Graham Squares)	1000_Grains	34	46	-12	4	2	E
57221800	Cereal, fruit whirls	1000_Grains	34	44	-10	4	2.5	D
57240100	Cereal (General Mills Chex Honey Nut)	1000_Grains	34	43	-9	4	2	D
57307600	Mini-Swirlz Cinnamon Bun Cereal, Kellogg's	1000_Grains	34	40	-6	4	3	D
51102020	Bread, white with whole wheat swirl, toasted	1000_Grains	34	38	-4	4	4	C
51404010	Bread, pumpernickel	1000_Grains	34	37	-3	4	4	C
51404550	Muffin, English, pumpernickel	1000_Grains	34	37	-3	4	4	C
51407010	Bread, black	1000_Grains	34	37	-3	4	4	C
51404020	Bread, pumpernickel, toasted	1000_Grains	34	36	-2	4	4	C
51407020	Bread, black, toasted	1000_Grains	34	36	-2	4	4	C
51808100	Roll, gluten free	1000_Grains	34	35	-1	4	3.5	C
53400200	Blintz, cheese-filled	1000_Grains	34	26	8	2	3	D
55300010	French toast, NFS	1000_Grains	34	25	9	3	3	D
55301000	French toast, plain	1000_Grains	34	24	10	3	3	D
56209000	Cream of rye	1000_Grains	34	22	12	1	3.5	A
57407100	Cereal (General Mills Trix)	1000_Grains	33	47	-14	4	2	D
57148600	Harmony cereal, General Mills	1000_Grains	33	44	-11	4	2	D
57344010	Cereal (Kellogg's Special K Red Berries)	1000_Grains	33	42	-9	4	2.5	D
56207102	Cream of wheat, instant, made with non-dairy milk, no added fat	1000_Grains	33	40	-7	4	3.5	C
55200100	Waffle, plain, from fast food / restaurant	1000_Grains	33	39	-6	4	2	E
53540906	Nature Valley Crunchy Granola Bar	1000_Grains	33	38	-5	4	2	D

56207092	Wheat, cream of, cooked, quick, made with milk, fat not added in cooking	1000_Grains	33	36	-3	4	3.5	C
56207342	Whole wheat cereal, wheat and barley, cooked, made with milk	1000_Grains	33	36	-3	4	4	A
52202060	Cornbread, made from home recipe	1000_Grains	33	26	7	1	3	D
52206060	Cornbread muffin, stick, round, made from home recipe	1000_Grains	33	26	7	1	3	D
56205400	Rice, cooked, NS as to type, fat added in cooking	1000_Grains	33	25	8	1	3	C
56201550	Cornmeal dumpling	1000_Grains	33	20	13	1	3.5	C
57409100	Cereal (Post Waffle Crisp)	1000_Grains	32	45	-13	4	2	E
57124200	Cereal, chocolate flavored, frosted, puffed corn	1000_Grains	32	42	-10	4	1.5	E
57303200	Cereal (Kellogg's Krave)	1000_Grains	32	39	-7	4	2	E
55100080	Pancakes, from school, NFS	1000_Grains	32	34	-2	4	3.5	C
51300185	Bread, paratha, wheat	1000_Grains	32	32	0	4	3	C
55100005	Pancakes, NFS	1000_Grains	32	28	4	3	3	D
55101000	Pancakes, plain	1000_Grains	32	28	4	3	3	D
56205006	Rice, white, cooked, made with margarine	1000_Grains	32	24	8	1	3.5	C
57245000	Just Right Fruit and Nut (formerly Just Right with raisins, dates, and nuts)	1000_Grains	31	41	-10	4	2.5	D
53540900	Nature Valley Chewy Trail Mix Granola Bar	1000_Grains	31	39	-8	4	1.5	E
52304200	Muffin, oat bran with fruit and/or nuts	1000_Grains	31	37	-6	4	3.5	C
55100055	Pancakes, with fruit, from fast food / restaurant	1000_Grains	31	36	-5	4	3	D
51122050	Bread, reduced calorie and/or high fiber, Italian	1000_Grains	31	31	0	4	4.5	A
56205007	Rice, white, cooked, fat added, NS as to fat type	1000_Grains	31	22	9	1	3.5	C
57104000	Cereal (Kellogg's Apple Jacks)	1000_Grains	30	48	-18	4	2.5	D
57344005	Cereal (Kellogg's Special K Chocolatey Delight)	1000_Grains	30	40	-10	4	2	E
57125900	Cereal (General Mills Honey Nut Clusters)	1000_Grains	30	39	-9	4	2.5	D
53544400	Granola bar, with rice cereal	1000_Grains	30	37	-7	4	2	E
56207230	Wheat, cream of, cooked, quick, fat added in cooking	1000_Grains	30	34	-4	4	3.5	B
51108100	Naan, Indian flatbread	1000_Grains	30	32	-2	4	3.5	D
55200020	Waffle, plain, from frozen	1000_Grains	30	32	-2	4	3	D
56207050	Wheat, cream of, cooked, made with milk and sugar, Puerto Rican style	1000_Grains	30	24	6	1	3	C
55101015	Pancakes, plain, reduced fat	1000_Grains	30	23	7	3	3	D
56207015	Cream of wheat, regular or quick, made with water, NS as to fat	1000_Grains	30	23	7	3	3.5	B
56207017	Cream of wheat, regular or quick, made with water, fat added	1000_Grains	30	23	7	3	3.5	B
56207016	Cream of wheat, regular or quick, made with water, no added fat	1000_Grains	30	22	8	3	3.5	A
56117110	Chow fun rice noodles, cooked, fat added in cooking	1000_Grains	30	21	9	1	3.5	C
56201515	Cornmeal mush, NS as to fat	1000_Grains	30	21	9	1	3.5	B
56205420	Rice, white, cooked, regular, fat added in cooking	1000_Grains	30	21	9	1	3	C
56205430	Rice, white, cooked, instant, fat added in cooking	1000_Grains	30	21	9	1	3	C
56201517	Cornmeal mush, fat added	1000_Grains	30	20	10	1	3.5	B
56204980	Rice, white, cooked, converted, NS as to fat added in cooking	1000_Grains	30	20	10	1	3	C

56205040	Rice, white, cooked, converted, fat not added in cooking	1000_Grains	30	20	10	1	3	C
56201516	Cornmeal mush, no added fat	1000_Grains	30	19	11	1	3.5	A
56207010	Wheat, cream of, cooked, regular, fat not added in cooking	1000_Grains	30	19	11	1	3.5	B
57213000	Cereal (Kellogg's Froot Loops)	1000_Grains	29	46	-17	4	2.5	D
57221650	Fruit Harvest cereal, Kellogg's	1000_Grains	29	44	-15	4	2.5	D
57106530	Cereal (Post Selects Blueberry Morning)	1000_Grains	29	40	-11	4	2.5	D
53710904	Cereal or granola bar (General Mills Nature Valley Sweet and Salty Granola Bar)	1000_Grains	29	37	-8	4	1	E
53712200	Cereal or granola bar, lowfat, NFS	1000_Grains	29	35	-6	4	2	D
57243870	Jenny O's	1000_Grains	29	32	-3	4	4	B
56201355	Grits, instant, made with non-dairy milk, no added fat	1000_Grains	29	31	-2	4	3.5	C
55200010	Waffle, NFS	1000_Grains	29	30	-1	4	3	D
55200040	Waffle, fruit, from frozen	1000_Grains	29	30	-1	4	3	D
55105400	Pancakes, rye	1000_Grains	29	23	6	2	3	D
56206990	Cream of wheat, NS as to regular, quick, or instant, NS as to fat	1000_Grains	29	23	6	3	3.5	B
56207005	Cream of wheat, NS as to regular, quick, or instant, fat added	1000_Grains	29	23	6	3	3.5	B
56201051	Grits, regular or quick, made with water, no added fat	1000_Grains	29	20	9	3	3.5	A
56205060	Rice, cooked, with milk	1000_Grains	29	19	10	1	3.5	C
56201010	Grits, cooked, corn or hominy, regular, fat not added in cooking	1000_Grains	29	17	12	1	3.5	B
56201030	Grits, cooked, corn or hominy, regular, NS as to fat added in cooking	1000_Grains	29	17	12	1	3.5	B
57124900	Cereal (Kellogg's Cinnabon)	1000_Grains	28	42	-14	4	1.5	E
57341300	Cereal (Kellogg's Smorz)	1000_Grains	28	42	-14	4	1.5	E
57355000	Cereal (Post Golden Crisp)	1000_Grains	28	42	-14	4	2	D
56201082	Grits, cooked, corn or hominy, with cheese, quick, fat added in cooking	1000_Grains	28	28	0	4	3	C
56201050	Grits, regular or quick, made with water, NS as to fat	1000_Grains	28	21	7	3	3.5	B
56201052	Grits, regular or quick, made with water, fat added	1000_Grains	28	21	7	3	3.5	B
56207000	Cream of wheat, NS as to regular, quick, or instant, no added fat	1000_Grains	28	21	7	3	3.5	A
51000250	Roll, made from home recipe or purchased at a bakery, NS as to major flour	1000_Grains	28	18	10	2	3	D
51151060	Roll, white, soft, made from home recipe or purchased at a bakery	1000_Grains	28	18	10	2	3	D
56205300	Rice, white and wild, cooked, no added fat	1000_Grains	28	16	12	1	3	C
56207150	Couscous, plain, cooked, fat not added in cooking	1000_Grains	28	16	12	1	3.5	B
56207160	Couscous, plain, cooked	1000_Grains	28	16	12	1	3.5	B
56201600	Masa harina, cooked	1000_Grains	28	15	13	1	3.5	C
57213010	Cereal (Kellogg's Froot Loops Marshmallow)	1000_Grains	27	45	-18	4	2	D
53540904	Nature Valley Sweet and Salty Nut Granola Bar	1000_Grains	27	36	-9	4	1	E
53714230	Cereal or granola bar, oats, nuts, coated with non-chocolate coating	1000_Grains	27	35	-8	4	1.5	E
53544230	Granola bar, oats, nuts, coated with non-chocolate coating	1000_Grains	27	34	-7	4	1.5	E
53542000	Snack bar, oatmeal	1000_Grains	27	33	-6	4	2	D
53542000	Snack bar, oatmeal	1000_Grains	27	32	-5	4	2	D

55100050	Pancakes, plain, from fast food / restaurant	1000_Grains	27	32	-5	4	2.5	D
55200110	Waffle, chocolate, from fast food / restaurant	1000_Grains	27	31	-4	4	1	E
57131000	Cereal (Quaker Corn Bran Crunch)	1000_Grains	27	30	-3	4	3	D
51302520	Muffin, English, wheat bran, with raisins	1000_Grains	27	28	-1	4	4	B
51501010	Bread, oatmeal	1000_Grains	27	28	-1	4	3.5	C
51501020	Bread, oatmeal, toasted	1000_Grains	27	28	-1	4	3.5	C
51501040	Bread, oat bran	1000_Grains	27	28	-1	4	3.5	C
51501050	Bread, oat bran, toasted	1000_Grains	27	28	-1	4	3.5	C
51502010	Roll, oatmeal	1000_Grains	27	28	-1	4	3.5	C
51503000	Muffin, English, oat bran	1000_Grains	27	28	-1	4	3.5	C
51303050	Muffin, English, wheat or cracked wheat, with raisins	1000_Grains	27	27	0	4	4	C
51303070	Muffin, English, whole wheat, with raisins	1000_Grains	27	27	0	4	4	C
52307120	Muffin, multigrain, with fruit	1000_Grains	27	25	2	4	3.5	C
53430100	Crepe, chocolate filled	1000_Grains	27	20	7	3	3	C
56200990	Grits, NS as to regular, quick, or instant, NS as to fat	1000_Grains	27	20	7	3	3.5	B
56201040	Grits, NS as to regular, quick, or instant, fat added	1000_Grains	27	20	7	3	3.5	B
56201000	Grits, NS as to regular, quick, or instant, no added fat	1000_Grains	27	19	8	3	3.5	A
53430250	Crepe suzette	1000_Grains	27	16	11	2	2.5	D
57323000	Cereal (Quaker Sweet Crunch)	1000_Grains	26	37	-11	4	0.5	E
53540800	Kashi GOLEAN Chewy Bars	1000_Grains	26	34	-8	4	2	D
56207101	Cream of wheat, instant, made with non-dairy milk, NS as to fat	1000_Grains	26	32	-6	4	3	C
56207103	Cream of wheat, instant, made with non-dairy milk, fat added	1000_Grains	26	32	-6	4	3	C
53720210	Nutrition bar (Clif Kids Organic Zbar)	1000_Grains	26	30	-4	4	2.5	D
52306300	Muffin, cheese	1000_Grains	26	29	-3	4	2	D
55203000	Waffle, fruit	1000_Grains	26	28	-2	4	2.5	D
51320010	Roll, wheat or cracked wheat	1000_Grains	26	27	-1	4	3.5	C
51320500	Roll, whole wheat	1000_Grains	26	27	-1	4	3.5	C
51320510	Roll, whole wheat, NS as to 100%, toasted	1000_Grains	26	26	0	4	3.5	C
56201081	Grits, cooked, corn or hominy, with cheese, quick, fat not added in cooking	1000_Grains	26	25	1	4	3	C
51301510	Bread, wheat or cracked wheat, reduced calorie and/or high fiber	1000_Grains	26	22	4	4	4.5	A
51602010	Bread, multigrain, reduced calorie and/or high fiber	1000_Grains	26	22	4	4	4.5	A
55301030	French toast sticks, NFS	1000_Grains	26	22	4	3	2.5	D
56205090	Rice, cream of, cooked, fat added	1000_Grains	26	17	9	1	3.5	B
56205092	Rice, cream of, cooked, NS as to fat	1000_Grains	26	17	9	1	3.5	B
55105100	Pancakes, cornmeal	1000_Grains	26	16	10	2	2.5	D
57416010	Cereal, puffed wheat, sweetened	1000_Grains	25	37	-12	4	2	D
53710800	Cereal or granola bar (Kashi Chewy)	1000_Grains	25	34	-9	4	2	D
53540000	Breakfast bar, NFS	1000_Grains	25	34	-9	4	2	E

53540200	Breakfast bar, cereal crust with fruit filling, lowfat	1000_Grains	25	34	-9	4	2	D
51184260	Breadsticks, soft, stuffed with melted cheese	1000_Grains	25	30	-5	4	2	D
51207010	Bread, sprouted wheat	1000_Grains	25	27	-2	4	3.5	C
51207020	Bread, sprouted wheat, toasted	1000_Grains	25	27	-2	4	3.5	C
51301010	Bread, wheat or cracked wheat	1000_Grains	25	27	-2	4	3.5	C
51801010	Bread, barley	1000_Grains	25	27	-2	4	3.5	C
52401000	Bread, Boston Brown	1000_Grains	25	27	-2	4	3.5	C
55200050	Waffle, chocolate, from frozen	1000_Grains	25	27	-2	4	3	D
56201340	Grits, instant, made with milk, fat added	1000_Grains	25	27	-2	4	3	C
56201344	Grits, instant, made with milk, NS as to fat	1000_Grains	25	27	-2	4	3	C
56207094	Cream of wheat, instant, made with milk, fat added	1000_Grains	25	27	-2	4	3.5	C
51320020	Roll, wheat or cracked wheat, toasted	1000_Grains	25	26	-1	4	3.5	C
51320070	Roll, wheat or cracked wheat, hamburger bun	1000_Grains	25	26	-1	4	4	C
56201342	Grits, instant, made with milk, no added fat	1000_Grains	25	26	-1	4	3.5	C
51320060	Roll, wheat or cracked wheat, hot dog bun	1000_Grains	25	25	0	4	4	C
51301520	Bread, wheat or cracked wheat, reduced calorie and/or high fiber, toasted	1000_Grains	25	21	4	4	4.5	A
51602020	Bread, multigrain, reduced calorie and/or high fiber, toasted	1000_Grains	25	21	4	4	4.5	A
55103100	Pancakes, with chocolate	1000_Grains	25	20	5	3	2	E
55610300	Dumpling, plain	1000_Grains	25	16	9	2	3	C
56205050	Rice, cream of, cooked, no added fat	1000_Grains	25	14	11	1	3.5	A
57342010	Smorz, Kellogg's	1000_Grains	24	39	-15	4	1.5	E
57120000	Cereal (Quaker Cap'n Crunch's Peanut Butter Crunch)	1000_Grains	24	38	-14	4	1	E
53540200	Breakfast bar, cereal crust with fruit filling, lowfat	1000_Grains	24	31	-7	4	2	D
51183990	Breadsticks, NFS	1000_Grains	24	29	-5	4	3	D
51184200	Breadsticks, soft, NFS	1000_Grains	24	29	-5	4	3	D
51184210	Breadsticks, soft, from fast food / restaurant	1000_Grains	24	29	-5	4	3	D
52303500	Muffin, wheat	1000_Grains	24	29	-5	4	2	D
52304000	Muffin, whole grain	1000_Grains	24	29	-5	4	2	D
53544250	Granola bar, coated with non-chocolate coating	1000_Grains	24	29	-5	4	0.5	E
53713000	Cereal or granola bar, reduced sugar, NFS	1000_Grains	24	29	-5	4	2	D
53714250	Cereal or granola bar, coated with non-chocolate coating	1000_Grains	24	29	-5	4	0.5	E
51184250	Breadsticks, soft, topped with melted cheese	1000_Grains	24	28	-4	4	2	D
52304150	Muffin, oat bran	1000_Grains	24	27	-3	4	3.5	C
56207096	Cream of wheat, instant, made with milk, NS as to fat	1000_Grains	24	27	-3	4	3.5	C
57349020	Cereal (Kellogg's Frosted Flakes, Reduced Sugar)	1000_Grains	24	27	-3	4	3	D
51301020	Bread, wheat or cracked wheat, toasted	1000_Grains	24	26	-2	4	4	C
51801020	Bread, barley, toasted	1000_Grains	24	26	-2	4	4	C
56207095	Cream of wheat, instant, made with milk, no added fat	1000_Grains	24	26	-2	4	3.5	C

57325000	Cereal (Kellogg's Product 19)	1000_Grains	24	26	-2	4	2	D
51303020	Muffin, English, wheat or cracked wheat, toasted	1000_Grains	24	24	0	4	4	B
51303040	Muffin, English, whole wheat, NS as to 100%, toasted	1000_Grains	24	24	0	4	4	B
52215100	Tortilla, corn	1000_Grains	24	21	3	4	4.5	A
57305170	Cereal (Malt-O-Meal Coco-Roos)	1000_Grains	23	39	-16	4	1.5	E
57243000	Cereal (Kellogg's Honey Smacks)	1000_Grains	23	37	-14	4	2	D
57306500	Cereal (Malt-O-Meal Golden Puffs)	1000_Grains	23	37	-14	4	2	D
53711002	Cereal or granola bar (Quaker Chewy 90 Calorie Granola Bar)	1000_Grains	23	35	-12	4	2	D
57302100	Cereal (Quaker King Vitaman)	1000_Grains	23	31	-8	4	2	E
57239000	Honeycomb, strawberry	1000_Grains	23	29	-6	4	2	E
51184030	Bread stick, soft, prepared with garlic and parmesan cheese	1000_Grains	23	28	-5	4	3.5	D
51184230	Breadsticks, soft, with parmesan cheese, from fast food / restaurant	1000_Grains	23	28	-5	4	2	D
53543000	Granola bar, reduced sugar, NFS	1000_Grains	23	28	-5	4	2	D
55203500	Waffle, nut and honey	1000_Grains	23	27	-4	4	2	D
56201350	Grits, instant, made with non-dairy milk, NS as to fat	1000_Grains	23	26	-3	4	3	C
56201360	Grits, instant, made with non-dairy milk, fat added	1000_Grains	23	26	-3	4	3	C
51301820	Bagel, wheat, with fruit and nuts	1000_Grains	23	25	-2	4	3.5	C
56201330	Grits, cooked, corn or hominy, quick, made with milk, fat added in cooking	1000_Grains	23	24	-1	4	3.5	C
56201332	Grits, cooked, corn or hominy, quick, made with milk, fat not added in cooking	1000_Grains	23	24	-1	4	3.5	C
51302500	Muffin, English, wheat bran	1000_Grains	23	23	0	4	4	B
51303010	Muffin, English, wheat or cracked wheat	1000_Grains	23	23	0	4	4	B
51303030	Muffin, English, whole wheat	1000_Grains	23	23	0	4	4	B
51630200	Muffin, English, multigrain	1000_Grains	23	23	0	4	4	B
55100060	Pancakes, with chocolate, from fast food / restaurant	1000_Grains	23	23	0	4	1.5	E
51122000	Bread, reduced calorie and/or high fiber, white or NFS	1000_Grains	23	19	4	4	4.5	A
56205000	Rice, cooked, NFS	1000_Grains	23	11	12	1	3.5	C
56205001	Rice, white, cooked, NS as to fat	1000_Grains	23	11	12	1	3.5	C
56205008	Rice, white, cooked, no added fat	1000_Grains	23	11	12	1	3.5	C
57322500	Oreo O's cereal, Post	1000_Grains	22	38	-16	4	1.5	E
53541000	Quaker Chewy Granola Bar	1000_Grains	22	36	-14	4	2	E
53541002	Quaker Chewy 90 Calorie Granola Bar	1000_Grains	22	34	-12	4	2	D
53542100	Granola bar, NFS	1000_Grains	22	27	-5	4	1	E
56201120	Grits, cooked, corn or hominy, quick, fat added in cooking	1000_Grains	22	24	-2	4	3	C
55206000	Waffle, oat bran	1000_Grains	22	23	-1	4	3	D
57306100	Malt-O-Meal Puffed Rice	1000_Grains	22	23	-1	4	3.5	B
57340000	Cereal, puffed rice	1000_Grains	22	23	-1	4	3.5	B
51302050	Bread, wheat bran, with raisins	1000_Grains	22	22	0	4	3.5	D
51421000	Roll, pumpernickel	1000_Grains	22	22	0	4	4	B

56201240	Grits, cooked, flavored, corn or hominy, instant, fat not added in cooking	1000_Grains	22	22	0	4	3.5	C
51122010	Bread, reduced calorie and/or high fiber, white or NFS, toasted	1000_Grains	22	18	4	4	4.5	B
51000180	Bread, made from home recipe or purchased at a bakery, NS as to major flour	1000_Grains	22	10	12	1	3	D
51101050	Bread, white, made from home recipe or purchased at a bakery	1000_Grains	22	10	12	1	3	D
56204990	Rice, white, cooked, regular, NS as to fat added in cooking	1000_Grains	22	10	12	1	3	C
56205010	Rice, white, cooked, regular, fat not added in cooking	1000_Grains	22	10	12	1	3	C
56205020	Rice, white, cooked, instant, NS as to fat added in cooking	1000_Grains	22	10	12	1	3	C
56205030	Rice, white, cooked, instant, fat not added in cooking	1000_Grains	22	10	12	1	3	C
57307150	Marshmallow Safari, Quaker	1000_Grains	21	37	-16	4	1.5	E
57328000	Cereal (Quaker Quisp)	1000_Grains	21	37	-16	4	0.5	E
57126500	Cocoa Blasts, Quaker	1000_Grains	21	30	-9	4	1.5	E
52405010	Bread, fruit	1000_Grains	21	28	-7	4	2	D
52304100	Muffin, oatmeal	1000_Grains	21	25	-4	4	2	D
51121065	Garlic bread, with melted cheese, from fast food / restaurant	1000_Grains	21	24	-3	4	2	D
55100020	Pancakes, with fruit, from frozen	1000_Grains	21	23	-2	4	3	C
51804010	Bread, soy	1000_Grains	21	22	-1	4	3.5	C
51805010	Bread, sunflower meal	1000_Grains	21	22	-1	4	3.5	C
55100010	Pancakes, plain, from frozen	1000_Grains	21	22	-1	4	3	C
51301805	Bagel, whole wheat, with raisins	1000_Grains	21	21	0	4	3.5	C
51302060	Bread, wheat bran, with raisins, toasted	1000_Grains	21	21	0	4	3	C
51421100	Roll, pumpernickel, toasted	1000_Grains	21	21	0	4	4	B
55301040	French toast sticks, plain, from fast food / restaurant	1000_Grains	21	21	0	4	2	D
57134000	Cereal, corn flakes	1000_Grains	21	21	0	4	2.5	D
57135000	Cereal (Kellogg's Corn Flakes)	1000_Grains	21	21	0	4	2.5	D
51301800	Bagel, wheat, with raisins	1000_Grains	21	20	1	4	3.5	C
51630100	Bagel, multigrain, with raisins	1000_Grains	21	20	1	4	3.5	C
51630110	Bagel, multigrain, with raisins, toasted	1000_Grains	21	20	1	4	3.5	C
51122100	Bread, reduced calorie and/or high fiber, white or NFS, with fruit and/or nuts	1000_Grains	21	19	2	4	4	C
51000190	Bread, made from home recipe or purchased at a bakery, toasted, NS as to major flour	1000_Grains	21	9	12	1	3	D
51101060	Bread, white, made from home recipe or purchased at a bakery, toasted	1000_Grains	21	9	12	1	3	D
56205101	Congee	1000_Grains	21	8	13	1	3.5	C
56201510	Cornmeal mush, made with water	1000_Grains	21	7	14	1	3.5	C
53711000	Cereal or granola bar (Quaker Chewy Granola Bar)	1000_Grains	20	33	-13	4	1.5	E
53540700	Kellogg's Special K bar	1000_Grains	20	29	-9	4	1.5	E
52403000	Bread, nut	1000_Grains	20	26	-6	4	2	D
56202910	Oatmeal, from fast food, fruit flavored	1000_Grains	20	25	-5	4	3	C
51108010	Focaccia, Italian flatbread, plain	1000_Grains	20	22	-2	4	3	C

51121055	Garlic bread, with parmesan cheese, from frozen	1000_Grains	20	22	-2	4	2	D
51121075	Garlic bread, with melted cheese, from frozen	1000_Grains	20	22	-2	4	2	D
51184010	Bread stick, soft	1000_Grains	20	22	-2	4	2.5	D
55201000	Waffle, plain	1000_Grains	20	22	-2	4	2	D
55207000	Waffle, multi-bran	1000_Grains	20	22	-2	4	3	D
51804020	Bread, soy, toasted	1000_Grains	20	21	-1	4	4	D
51805020	Bread, sunflower meal, toasted	1000_Grains	20	21	-1	4	3.5	D
55300050	French toast, plain, from fast food / restaurant	1000_Grains	20	20	0	4	2	D
55301050	French toast sticks, plain	1000_Grains	20	20	0	4	2	D
57326000	Cereal (Barbara's Puffins)	1000_Grains	20	20	0	4	3.5	D
51122110	Bread, reduced calorie and/or high fiber, white or NFS, with fruit and/or nuts, toasted	1000_Grains	20	18	2	4	4	C
51502100	Roll, oat bran	1000_Grains	20	18	2	4	4	C
52308020	Matzo ball	1000_Grains	20	15	5	4	3.5	A
56205080	Rice, creamed, made with milk and sugar, Puerto Rican style	1000_Grains	20	14	6	1	3.5	C
56205004	Rice, white, cooked, made with butter	1000_Grains	20	9	11	1	3	C
57124500	Cinnamon Grahams, General Mills	1000_Grains	19	31	-12	4	1.5	E
57238000	Cereal (Post Honeycomb)	1000_Grains	19	29	-10	4	2	E
53540000	Breakfast bar, NFS	1000_Grains	19	25	-6	4	1.5	E
56202905	Oatmeal, from fast food, maple flavored	1000_Grains	19	25	-6	4	3.5	C
56202920	Oatmeal, from fast food, other flavors	1000_Grains	19	25	-6	4	3.5	C
51111010	Bread, cheese	1000_Grains	19	24	-5	4	1.5	E
51111040	Bread, cheese, toasted	1000_Grains	19	23	-4	4	1.5	E
53544220	Granola bar with nuts, chocolate-coated	1000_Grains	19	23	-4	4	1	E
51121015	Garlic bread, NFS	1000_Grains	19	22	-3	4	2	D
56207060	Cream of wheat, instant, made with water, fat added	1000_Grains	19	22	-3	4	3.5	C
56207070	Cream of wheat, instant, made with water, NS as to fat	1000_Grains	19	22	-3	4	3.5	C
51121025	Garlic bread, from fast food / restaurant	1000_Grains	19	21	-2	4	2	D
52208760	Gordita/sope shell, plain, no filling	1000_Grains	19	21	-2	4	3	C
51302010	Bread, wheat bran	1000_Grains	19	20	-1	4	3.5	C
51301700	Bagel, wheat	1000_Grains	19	18	1	4	4	C
51301710	Bagel, wheat, toasted	1000_Grains	19	18	1	4	3.5	C
51301750	Bagel, whole wheat	1000_Grains	19	18	1	4	4	C
51301760	Bagel, whole wheat, NS as to 100%, toasted	1000_Grains	19	18	1	4	3.5	C
51301900	Bagel, wheat bran	1000_Grains	19	18	1	4	4	C
51404500	Bagel, pumpernickel	1000_Grains	19	18	1	4	4	C
51501080	Bagel, oat bran	1000_Grains	19	18	1	4	4	C
51630000	Bagel, multigrain	1000_Grains	19	18	1	4	4	C
55310100	Fried bread, Puerto Rican style	1000_Grains	19	17	2	2	2	D

56201520	Cornmeal mush, fried	1000_Grains	19	6	13	1	3.5	C
57221700	Cereal, fruit rings	1000_Grains	18	27	-9	4	1.5	E
52306500	Muffin, pumpkin	1000_Grains	18	23	-5	4	2	D
52404060	Bread, pumpkin	1000_Grains	18	23	-5	4	2	D
51184220	Breadsticks, soft, from frozen	1000_Grains	18	22	-4	4	3.5	D
51184240	Breadsticks, soft, with parmesan cheese, from frozen	1000_Grains	18	22	-4	4	3.5	D
52306700	Muffin, carrot	1000_Grains	18	22	-4	4	2	D
53714220	Cereal or granola bar with nuts, chocolate coated	1000_Grains	18	22	-4	4	1	E
51302020	Bread, wheat bran, toasted	1000_Grains	18	19	-1	4	3.5	C
55100025	Pancakes, with chocolate, from frozen	1000_Grains	18	19	-1	4	2.5	D
51301830	Bagel, wheat, with fruit and nuts, toasted	1000_Grains	18	18	0	4	3.5	C
56201540	Cornmeal, Puerto Rican Style	1000_Grains	18	13	5	1	3	C
53711004	Cereal or granola bar (Quaker Chewy 25% Less Sugar Granola Bar)	1000_Grains	17	29	-12	4	2	D
57100500	Character cereals, TV or movie, Kellogg's	1000_Grains	17	26	-9	4	1.5	E
57347000	Cereal (Kellogg's Corn Pops)	1000_Grains	17	26	-9	4	3	D
53710700	Cereal or granola bar (Kellogg's Special K bar)	1000_Grains	17	23	-6	4	2	E
52302600	Muffin, chocolate	1000_Grains	17	21	-4	4	1.5	E
51121035	Garlic bread, from frozen	1000_Grains	17	20	-3	4	2	D
56207030	Cream of wheat, instant, made with water, no added fat	1000_Grains	17	20	-3	4	3.5	B
57344000	Cereal (Kellogg's Special K)	1000_Grains	17	20	-3	4	2	D
51121045	Garlic bread, with parmesan cheese, from fast food / restaurant	1000_Grains	17	19	-2	4	2	D
57403100	Toasties, Post	1000_Grains	17	19	-2	4	3	D
56205170	Yellow rice, cooked, fat added	1000_Grains	17	18	-1	4	3	C
51620020	Roll, multigrain, hot dog bun	1000_Grains	17	16	1	4	3.5	C
56206970	Wheat, cream of, cooked, quick, NS as to fat added in cooking	1000_Grains	17	16	1	4	3.5	B
56207020	Wheat, cream of, cooked, quick, fat not added in cooking	1000_Grains	17	16	1	4	3.5	B
53541004	Quaker Chewy 25% Less Sugar Granola Bar	1000_Grains	16	28	-12	4	2	D
57126000	Cereal (Kellogg's Cocoa Krispies)	1000_Grains	16	28	-12	4	1.5	E
53711100	Cereal or granola bar (Quaker Granola Bites)	1000_Grains	16	23	-7	4	1.5	E
53714200	Cereal or granola bar, chocolate coated, NFS	1000_Grains	16	22	-6	4	1	E
52306550	Muffin, zucchini	1000_Grains	16	20	-4	4	2	D
52407000	Bread, zucchini	1000_Grains	16	20	-4	4	2	D
51121040	Bread, garlic, toasted	1000_Grains	16	19	-3	4	2	D
51121010	Bread, garlic	1000_Grains	16	18	-2	4	2	D
55211050	Waffle, plain, reduced fat	1000_Grains	16	18	-2	4	2	D
56201220	Grits, instant, made with water, fat added	1000_Grains	16	17	-1	4	3	C
56201230	Grits, instant, made with water, NS as to fat	1000_Grains	16	17	-1	4	3	C
55203600	Waffle, chocolate	1000_Grains	16	16	0	4	1.5	E
51000400	Roll, bran, NS as to type of bran	1000_Grains	16	15	1	4	3.5	C

51301120	Bread, wheat or cracked wheat, with raisins	1000_Grains	16	15	1	4	2.5	D
51301130	Bread, wheat or cracked wheat, with raisins, toasted	1000_Grains	16	15	1	4	2.5	D
51620000	Roll, multigrain	1000_Grains	16	15	1	4	3.5	C
51620030	Roll, multigrain, hamburger bun	1000_Grains	16	15	1	4	3.5	C
51401010	Bread, rye	1000_Grains	16	13	3	4	4	C
51401030	Bread, marble rye and pumpernickel	1000_Grains	16	13	3	4	4	C
51420000	Roll, rye	1000_Grains	16	13	3	4	4	C
52104010	Biscuit, home recipe	1000_Grains	16	8	8	1	1.5	E
56205070	Rice, sweet, cooked with honey	1000_Grains	16	3	13	1	3	C
57305174	Cereal (Malt-O-Meal Colossal Crunch)	1000_Grains	15	30	-15	4	1	E
53544410	Quaker Granola Bites	1000_Grains	15	22	-7	4	1.5	E
57323050	Sweet Puffs, Quaker	1000_Grains	15	22	-7	4	2	D
52304040	Muffin, bran with fruit, lowfat	1000_Grains	15	16	-1	4	3	D
51129010	Bread, raisin	1000_Grains	15	15	0	4	3.5	D
52209010	Hush puppy	1000_Grains	15	15	0	4	2.5	D
57148000	Cereal (Kellogg's Crispix)	1000_Grains	15	14	1	4	2	D
51401020	Bread, rye, toasted	1000_Grains	15	12	3	4	4	C
51401040	Bread, marble rye and pumpernickel, toasted	1000_Grains	15	12	3	4	4	C
51152000	Roll, white, soft, reduced calorie and/or high fiber	1000_Grains	15	10	5	4	4	B
56205190	Rice, white, cooked, glutinous	1000_Grains	15	2	13	1	3.5	C
57316750	Oh's, Fruitangy, Quaker	1000_Grains	14	29	-15	4	1.5	E
57306800	Cereal (Malt-O-Meal Tootie Fruities)	1000_Grains	14	27	-13	4	1.5	E
51184020	Bread stick, NS as to hard or soft	1000_Grains	14	16	-2	4	2.5	D
52320110	Toaster muffin, fruit, toasted	1000_Grains	14	16	-2	4	2	D
52304060	Muffin, bran with fruit, no fat, no cholesterol	1000_Grains	14	15	-1	4	4	C
51129020	Bread, raisin, toasted	1000_Grains	14	14	0	4	3.5	D
57148500	Cereal, crispy brown rice	1000_Grains	14	14	0	4	2.5	D
55100015	Pancakes, plain, reduced fat, from fozen	1000_Grains	14	13	1	4	3	D
56201210	Grits, instant, made with water, no added fat	1000_Grains	14	13	1	4	3.5	B
57151000	Cereal, crispy rice	1000_Grains	14	13	1	4	2.5	D
57305200	Cereal (Malt-O-Meal Crispy Rice)	1000_Grains	14	13	1	4	2.5	D
57337000	Cereal, rice flakes	1000_Grains	14	13	1	4	2.5	D
57339000	Cereal (Kellogg's Rice Krispies)	1000_Grains	14	13	1	4	2.5	D
51127010	Bread, potato	1000_Grains	14	12	2	4	4	A
51127020	Bread, potato, toasted	1000_Grains	14	11	3	4	4	C
52208750	Gordita/sope shell, plain, no filling, grilled, no fat added	1000_Grains	14	11	3	4	4	B
51207010	Bread, sprouted wheat	1000_Grains	14	9	5	4	4	C
52301000	Muffin, NFS	1000_Grains	13	20	-7	4	2	E
52302010	Muffin, fruit	1000_Grains	13	20	-7	4	2	E

52306010	Muffin, plain	1000_Grains	13	17	-4	4	2	D
53544200	Granola bar, chocolate-coated, NFS	1000_Grains	13	17	-4	4	0.5	E
53714210	Cereal or granola bar, with coconut, chocolate coated	1000_Grains	13	17	-4	4	0.5	E
52405100	Bread, fruit and nut	1000_Grains	13	16	-3	4	2	D
53544210	Granola bar, with coconut, chocolate-coated	1000_Grains	13	16	-3	4	0.5	E
55301031	French toast sticks, plain, from frozen	1000_Grains	13	14	-1	4	2.5	D
51000300	Roll, hard, NS as to major flour	1000_Grains	13	13	0	4	3.5	C
51153000	Roll, white, hard	1000_Grains	13	13	0	4	3.5	C
51156500	Roll, garlic	1000_Grains	13	13	0	4	3.5	C
51153010	Roll, white, hard, toasted	1000_Grains	13	12	1	4	3.5	D
55200030	Waffle, plain, reduced fat, from frozen	1000_Grains	13	12	1	4	3	D
51207020	Bread, sprouted wheat, toasted	1000_Grains	13	9	4	4	4.5	C
57305175	Cereal (Malt-O-Meal Cocoa Dyno-Bites)	1000_Grains	12	22	-10	4	1.5	E
57213900	Frosted Chex	1000_Grains	12	18	-6	4	1.5	E
57349000	Cereal (Kellogg's Frosted Flakes)	1000_Grains	12	17	-5	4	2	E
51154600	Roll, cheese	1000_Grains	12	14	-2	4	3	D
51155000	Roll, French or Vienna	1000_Grains	12	11	1	4	3.5	C
51119010	Bread, egg, Challah	1000_Grains	12	10	2	4	3.5	C
51119100	Bread, lowfat, 98% fat free	1000_Grains	12	10	2	4	3.5	C
51154550	Roll, egg bread	1000_Grains	12	10	2	4	3.5	C
52311010	Popover	1000_Grains	12	7	5	4	3	D
56117090	Rice noodles, cooked	1000_Grains	12	1	11	1	3.5	C
56117100	Chow fun rice noodles, cooked, fat not added in cooking	1000_Grains	12	1	11	1	3.5	C
57127000	Cereal (Post Cocoa Pebbles)	1000_Grains	11	24	-13	4	1.5	E
57239100	Cereal (Kellogg's Honey Crunch Corn Flakes)	1000_Grains	11	17	-6	4	1.5	E
52302500	Muffin, chocolate chip	1000_Grains	11	16	-5	4	1.5	E
53540500	Breakfast bar, date, with yogurt coating	1000_Grains	11	16	-5	4	2	C
51000100	Bread, NS as to major flour	1000_Grains	11	12	-1	4	3.5	C
51101000	Bread, white	1000_Grains	11	12	-1	4	3.5	C
51158100	Roll, Mexican, bolillo	1000_Grains	11	12	-1	4	3.5	D
51133010	Bread, sour dough	1000_Grains	11	11	0	4	3.5	D
51159000	Roll, sour dough	1000_Grains	11	11	0	4	3.5	D
52302610	Muffin, chocolate, lowfat	1000_Grains	11	10	1	4	2.5	D
51119040	Bread, egg, Challah, toasted	1000_Grains	11	9	2	4	3.5	C
55105300	Pancakes, sour dough	1000_Grains	11	9	2	4	3	C
55300020	French toast, plain, from frozen	1000_Grains	11	9	2	4	3.5	C
57117000	Cereal (Quaker Cap'n Crunch)	1000_Grains	10	24	-14	4	1	E
57117500	Cereal (Quaker Christmas Crunch)	1000_Grains	10	24	-14	4	1	E
57119000	Cereal (Quaker Cap'n Crunch's Crunchberries)	1000_Grains	10	24	-14	4	1	E

57144000	Crisp Crunch	1000_Grains	10	24	-14	4	1	E
51113010	Bread, cinnamon	1000_Grains	10	12	-2	4	3	C
51000110	Bread, NS as to major flour, toasted	1000_Grains	10	11	-1	4	3.5	C
51101010	Bread, white, toasted	1000_Grains	10	11	-1	4	3.5	C
51113100	Bread, cinnamon, toasted	1000_Grains	10	11	-1	4	3.5	C
51133020	Bread, sour dough, toasted	1000_Grains	10	10	0	4	3.5	D
51186120	Muffin, English, with raisins, toasted	1000_Grains	10	10	0	4	3.5	C
51000200	Roll, NS as to major flour	1000_Grains	10	9	1	4	3.5	D
51000230	Roll, NS as to major flour, toasted	1000_Grains	10	9	1	4	3.5	D
51150000	Roll, white, soft	1000_Grains	10	9	1	4	3.5	D
51150100	Roll, white, soft, toasted	1000_Grains	10	9	1	4	3.5	D
51154010	Roll, white, hot dog bun	1000_Grains	10	9	1	4	3.5	D
51154100	Roll, white, hamburger bun	1000_Grains	10	9	1	4	3.5	D
51157000	Roll, white, hoagie, submarine	1000_Grains	10	9	1	4	3.5	D
51186100	Muffin, English, with raisins	1000_Grains	10	8	2	4	3.5	C
51186160	Muffin, English, with fruit other than raisins	1000_Grains	10	8	2	4	4	C
57134090	Corn flakes, low sodium	1000_Grains	10	8	2	4	3.5	B
57305210	Cereal (Malt-O-Meal Frosted Flakes)	1000_Grains	9	15	-6	4	1.5	E
57348000	Cereal, frosted corn flakes	1000_Grains	9	15	-6	4	1.5	E
53714400	Cereal or granola bar, with rice cereal	1000_Grains	9	12	-3	4	2	D
52215350	Taco shell, flour	1000_Grains	9	9	0	4	1.5	E
52215000	Tortilla, NFS	1000_Grains	9	6	3	4	3.5	C
53541006	Quaker Chewy Dipps Granola Bar	1000_Grains	8	20	-12	4	1	E
57305180	Cereal (Malt-O-Meal Corn Bursts)	1000_Grains	8	15	-7	4	1	E
52215300	Taco shell, corn	1000_Grains	8	7	1	4	2.5	D
51154510	Roll, diet	1000_Grains	8	6	2	4	3.5	D
52101100	Biscuit, baking powder or buttermilk type, made from mix	1000_Grains	8	6	2	4	1.5	E
52220110	Arepa Dominicana	1000_Grains	8	6	2	4	2	D
51186010	Muffin, English	1000_Grains	8	5	3	4	4	C
51204010	Bread, wheat germ	1000_Grains	8	5	3	4	3.5	C
52101040	Crumpet	1000_Grains	8	5	3	4	4	C
53711006	Cereal or granola bar (Quaker Chewy Dipps Granola Bar)	1000_Grains	7	22	-15	4	1	E
57216000	Cereal, frosted rice	1000_Grains	7	13	-6	4	1.5	E
57218000	Cereal (Kellogg's Frosted Krispies)	1000_Grains	7	13	-6	4	1.5	D
52206010	Cornbread muffin, stick, round	1000_Grains	7	7	0	4	3	D
52304010	Muffin, wheat bran	1000_Grains	7	7	0	4	3	C
56205130	Yellow rice, cooked, NS as to fat	1000_Grains	7	6	1	4	3	C
56205150	Yellow rice, cooked, no added fat	1000_Grains	7	6	1	4	3	C
51106010	Bread, native, water, Puerto Rican style	1000_Grains	7	4	3	4	3	C

51109010	Bread, Italian, Grecian, Armenian	1000_Grains	7	4	3	4	3.5	D
51109150	Bread, pita with fruit	1000_Grains	7	4	3	4	3	D
51186020	Muffin, English, toasted	1000_Grains	7	4	3	4	3.5	C
56201110	Grits, cooked, corn or hominy, quick, fat not added in cooking	1000_Grains	7	4	3	4	3.5	B
56201130	Grits, cooked, corn or hominy, quick, NS as to fat added in cooking	1000_Grains	7	3	4	4	3.5	B
51140100	Bread, dough, fried	1000_Grains	6	6	0	4	3	D
51105010	Bread, Cuban	1000_Grains	6	5	1	4	3.5	D
51107010	Bread, French or Vienna	1000_Grains	6	5	1	4	3.5	D
51110010	Bread, batter	1000_Grains	6	5	1	4	3	C
52308010	Matzo, fritters	1000_Grains	6	5	1	4	1.5	D
51155010	Roll, French or Vienna, toasted	1000_Grains	6	3	3	4	3.5	C
57305300	Cereal (Malt-O-Meal Fruity Dyno-Bites)	1000_Grains	5	15	-10	4	1.5	E
53540600	Milk 'n Cereal bar	1000_Grains	5	12	-7	4	0.5	E
57339500	Cereal (Kellogg's Rice Krispies Treats Cereal)	1000_Grains	5	7	-2	4	1.5	E
51105040	Bread, Cuban, toasted	1000_Grains	5	4	1	4	3.5	D
51107040	Bread, French or Vienna, toasted	1000_Grains	5	4	1	4	3.5	D
52102040	Biscuit, from refrigerated dough	1000_Grains	5	4	1	4	1.5	E
52215200	Tortilla, flour	1000_Grains	5	4	1	4	2.5	D
52408000	Bread, Irish soda	1000_Grains	5	3	2	4	2	E
51109040	Bread, Italian, Grecian, Armenian, toasted	1000_Grains	5	2	3	4	3	D
55101010	Pancakes, reduced calorie, high fiber	1000_Grains	5	2	3	4	3	C
51180090	Bagel, with fruit other than raisins, toasted	1000_Grains	5	1	4	4	3.5	C
52207010	Corn flour patty or tart, fried	1000_Grains	5	1	4	4	3	C
57223000	Cereal (Post Fruity Pebbles)	1000_Grains	4	16	-12	4	1	E
53540600	Milk 'n Cereal bar	1000_Grains	4	11	-7	4	0.5	E
57213005	Froot Loops Cereal Straws	1000_Grains	4	9	-5	4	1.5	E
51161260	Roll, sweet, crumb topping, Mexican (Pan Dulce)	1000_Grains	4	6	-2	4	2	D
51109100	Bread, pita	1000_Grains	4	1	3	4	3.5	C
51134000	Bread, sweet potato	1000_Grains	4	1	3	4	3.5	C
51135000	Bread, vegetable	1000_Grains	4	1	3	4	3	D
51180030	Bagel, with raisins	1000_Grains	4	1	3	4	3	D
51180040	Bagel, with raisins, toasted	1000_Grains	4	1	3	4	3.5	C
52208010	Corn pone, baked	1000_Grains	4	1	3	4	3	C
52104040	Biscuit, wheat	1000_Grains	3	4	-1	4	1	E
52104100	Biscuit, cheese	1000_Grains	3	4	-1	4	1	E
51109110	Bread, pita, toasted	1000_Grains	3	1	2	4	3.5	C
51121110	Bread, onion	1000_Grains	3	1	2	4	3	C
51135010	Bread, vegetable, toasted	1000_Grains	3	1	2	4	3	D
51180080	Bagel, with fruit other than raisins	1000_Grains	3	1	2	4	3.5	C

52101150	Biscuit, baking powder or buttermilk type, made from refrigerated dough, lowfat	1000_Grains	3	1	2	4	2	E
57339100	Rice Krispies with Real Strawberries, Kellogg's	1000_Grains	2	11	-9	4	1.5	E
52302020	Muffin, fruit, low fat	1000_Grains	2	4	-2	4	2.5	D
52104200	Biscuit with fruit	1000_Grains	2	3	-1	4	1	E
51180010	Bagel	1000_Grains	2	1	1	4	3.5	C
57316710	Cereal (Quaker Honey Graham Oh's)	1000_Grains	1	12	-11	4	0.5	E
57100400	Character cereals, TV or movie, General Mills	1000_Grains	1	11	-10	4	1.5	E
57201800	Disney cereals, Kellogg's	1000_Grains	1	11	-10	4	1.5	E
51115010	Bread, cornmeal and molasses	1000_Grains	1	1	0	4	3	D
51115020	Bread, cornmeal and molasses, toasted	1000_Grains	1	1	0	4	3	D
51126010	Bread, milk and honey	1000_Grains	1	1	0	4	3	C
51126020	Bread, milk and honey, toasted	1000_Grains	1	1	0	4	3.5	C
51180020	Bagel, toasted	1000_Grains	1	1	0	4	3.5	C
52101000	Biscuit, NFS	1000_Grains	1	1	0	4	1	E
52101030	Biscuit dough, fried	1000_Grains	1	1	0	4	1.5	E
52103000	Biscuit, from fast food / restaurant	1000_Grains	1	1	0	4	1	E
52105100	Scone	1000_Grains	1	1	0	4	1.5	E
52105200	Scone, with fruit	1000_Grains	1	1	0	4	1.5	E
52201000	Cornbread, prepared from mix	1000_Grains	1	1	0	4	2	D
52208020	Corn pone, fried	1000_Grains	1	1	0	4	3	C
52302100	Muffin, fruit, fat free, cholesterol free	1000_Grains	1	1	0	4	3	D
55211000	Waffle, plain, fat free	1000_Grains	1	1	0	4	3	D
75132100	Celery juice	10000_Beverages	100	100	0	1	4	B
78101125	Fruit and vegetable smoothie, no dairy	10000_Beverages	98	100	-2	1	5	D
78101130	Vegetable smoothie	10000_Beverages	97	98	-1	1	4.5	C
75132000	Mixed vegetable juice	10000_Beverages	95	100	-5	1	3	B
78101100	Fruit and vegetable smoothie, with dairy	10000_Beverages	95	99	-4	1	5	C
78101118	Fruit and vegetable smoothie, non-dairy, added protein	10000_Beverages	92	95	-3	2	5	C
78101115	Fruit and vegetable smoothie, non-dairy	10000_Beverages	91	96	-5	1	5	C
64134015	Fruit smoothie, with whole fruit, no dairy	10000_Beverages	90	93	-3	1	5	D
92201010	Coffee substitute	10000_Beverages	90	85	5	1	4	B
74301150	Tomato juice, 100%, low sodium	10000_Beverages	89	97	-8	1	4	B
74303100	Tomato and vegetable juice, 100%, low sodium	10000_Beverages	89	97	-8	1	4	B
78101110	Fruit and vegetable smoothie, added protein	10000_Beverages	89	91	-2	2	5	B
64134100	Fruit smoothie, light	10000_Beverages	87	86	1	1	4.5	C
92203000	Cereal beverage	10000_Beverages	87	82	5	1	4	B
64134030	Fruit smoothie juice drink, no dairy	10000_Beverages	84	86	-2	1	5	D
73105010	Carrot juice, 100%	10000_Beverages	82	86	-4	1	4	B

61210820	Orange juice, 100%, with calcium added, frozen, reconstituted	10000_Beverages	81	92	-11	1	3	D
64104610	Blueberry juice	10000_Beverages	81	91	-10	1	2	C
73105000	Beet juice	10000_Beverages	81	85	-4	1	2.5	C
64104600	Blackberry juice, 100%	10000_Beverages	80	96	-16	1	3	D
64132500	Strawberry juice, 100%	10000_Beverages	79	95	-16	1	3	D
74301100	Tomato juice, 100%	10000_Beverages	77	90	-13	3	4	B
64133100	Watermelon juice, 100%	10000_Beverages	77	87	-10	1	4	C
61210250	Orange juice, 100%, with calcium added, canned, bottled or in a carton	10000_Beverages	77	85	-8	1	3	D
61219000	Orange and banana juice	10000_Beverages	77	83	-6	1	1	E
61213900	Fruit juice blend, citrus, 100% juice, with calcium added	10000_Beverages	76	85	-9	1	3	C
95120010	Nutritional drink or shake, high protein, ready-to-drink, NFS	10000_Beverages	74	87	-13	4	5	B
74304000	Tomato juice with clam or beef juice	10000_Beverages	74	86	-12	3	4	C
61210620	Orange juice, 100%, frozen, reconstituted	10000_Beverages	74	83	-9	1	3	C
11553110	Fruit smoothie, with whole fruit and dairy	10000_Beverages	74	72	2	1	4.5	D
61210720	Orange juice, 100%, frozen, not reconstituted	10000_Beverages	73	83	-10	1	1	E
74302000	Tomato juice cocktail	10000_Beverages	73	83	-10	3	4	B
74303000	Tomato and vegetable juice, 100%	10000_Beverages	73	83	-10	3	4	B
61210000	Orange juice, 100%, NFS	10000_Beverages	73	81	-8	1	3	D
64132020	Prune juice, unsweetened	10000_Beverages	73	76	-3	1	0.5	E
64134025	Fruit smoothie, with whole fruit, non-dairy	10000_Beverages	73	75	-2	3	4.5	C
95102010	Carnation Instant Breakfast, nutritional drink, sugar free, ready-to-drink	10000_Beverages	72	83	-11	4	5	B
64134020	Fruit smoothie, with whole fruit, no dairy, added protein	10000_Beverages	72	82	-10	4	5	C
61210010	Orange juice, 100%, freshly squeezed	10000_Beverages	72	80	-8	1	3	D
11553120	Fruit smoothie, with whole fruit and dairy, added protein	10000_Beverages	72	71	1	2	5	C
11553100	Fruit smoothie, NFS	10000_Beverages	72	69	3	1	4.5	C
95106010	Nutritional drink or shake, ready-to-drink, light (Muscle Milk)	10000_Beverages	71	84	-13	4	5	B
61219100	Pineapple-orange-banana juice	10000_Beverages	71	78	-7	1	1	D
92102501	Coffee, Iced Latte, nonfat	10000_Beverages	71	67	4	1	4.5	B
92161001	Coffee, Cappuccino, nonfat	10000_Beverages	71	67	4	1	4.5	B
92162001	Coffee, Cappuccino, decaffeinated, nonfat	10000_Beverages	71	67	4	1	4.5	B
95110020	Nutritional drink or shake, high protein, ready-to-drink (Slim Fast)	10000_Beverages	70	88	-18	4	5	B
61201020	Grapefruit juice, 100%, NS as to form	10000_Beverages	70	81	-11	1	3	D
61201220	Grapefruit juice, 100%, canned, bottled or in a carton	10000_Beverages	70	81	-11	1	3	D
61225000	Pineapple-orange juice, NFS	10000_Beverages	70	78	-8	1	1.5	D
61225200	Pineapple-orange juice, canned, NS as to sweetened or unsweetened; sweetened, NS as to type of sweetener	10000_Beverages	70	78	-8	1	1.5	D
61225220	Pineapple-orange juice, canned, bottled or in a carton	10000_Beverages	70	78	-8	1	1.5	D
61210220	Orange juice, 100%, canned, bottled or in a carton	10000_Beverages	70	77	-7	1	3	D
61225600	Pineapple-orange juice, frozen (reconstituted with water)	10000_Beverages	70	77	-7	1	1	D

64121000	Passion fruit juice, 100%	10000_Beverages	70	77	-7	1	2	E
11613000	Instant breakfast, powder, sweetened with low calorie sweetener, milk added	10000_Beverages	70	70	0	2	4	C
95110010	Nutritional drink or shake, ready-to-drink, sugar free (Slim Fast)	10000_Beverages	69	83	-14	4	4.5	B
61201225	Grapefruit juice, 100%, with calcium added	10000_Beverages	69	80	-11	1	3	D
64124060	Pineapple juice, unsweetened, with added Vitamin C	10000_Beverages	69	74	-5	1	0.5	E
92610010	Horchata beverage, made with almonds or other nuts and seeds	10000_Beverages	69	74	-5	1	0.5	E
64132010	Prune juice, 100%	10000_Beverages	69	71	-2	1	1	E
92101911	Coffee, Latte, decaffeinated, nonfat	10000_Beverages	69	64	5	1	4.5	B
61213220	Tangerine juice, 100%	10000_Beverages	68	76	-8	1	3	D
61213620	Tangerine juice, frozen (reconstituted with water)	10000_Beverages	68	76	-8	1	1.5	D
61216220	Grapefruit and orange juice, canned	10000_Beverages	68	76	-8	1	1.5	E
61222000	Pineapple-grapefruit juice, NFS	10000_Beverages	68	76	-8	1	1.5	D
64124010	Pineapple juice, NS as to sweetened or unsweetened	10000_Beverages	68	74	-6	1	0.5	E
64124200	Pineapple-apple-guava juice, with added vitamin C	10000_Beverages	68	74	-6	1	1	D
92101901	Coffee, Latte, nonfat	10000_Beverages	68	64	4	1	4.5	B
64125000	Pineapple juice-non-citrus juice blend, unsweetened, with added vitamin C	10000_Beverages	67	71	-4	1	0.5	E
11552200	Orange Julius	10000_Beverages	67	67	0	1	0.5	D
92102502	Coffee, Iced Latte, with non-dairy milk	10000_Beverages	66	78	-12	3	4	C
61201000	Grapefruit juice, NFS	10000_Beverages	66	75	-9	1	2	D
61201010	Grapefruit juice, 100%, freshly squeezed	10000_Beverages	66	75	-9	1	3	D
61216010	Grapefruit and orange juice, fresh	10000_Beverages	66	74	-8	1	2	C
61219150	Orange-white grape-peach juice	10000_Beverages	66	73	-7	1	1.5	E
95104000	Nutritional drink or shake, ready-to-drink, sugar free (Glucerna)	10000_Beverages	65	76	-11	4	4	B
61201620	Grapefruit juice,100%, frozen, reconstituted	10000_Beverages	65	73	-8	1	3	D
64105400	Cranberry juice, 100%, not a blend	10000_Beverages	65	73	-8	1	2.5	E
64104150	Apple-cherry juice	10000_Beverages	65	71	-6	1	1	D
61213800	Fruit juice blend, citrus, 100% juice	10000_Beverages	65	70	-5	1	2.5	E
95106000	Nutritional drink or shake, ready-to-drink (Muscle Milk)	10000_Beverages	64	77	-13	4	5	B
41436000	Nutritional supplement for people with diabetes, liquid	10000_Beverages	64	70	-6	4	4	B
92161002	Coffee, Cappuccino, with non-dairy milk	10000_Beverages	63	75	-12	3	4	C
61226000	Strawberry-banana-orange juice	10000_Beverages	63	71	-8	1	2.5	E
64124020	Pineapple juice, 100%	10000_Beverages	63	68	-5	1	2.5	E
64134000	Fruit smoothie drink, made with fruit or fruit juice only (no dairy products)	10000_Beverages	63	67	-4	2	0.5	D
95110000	Nutritional drink or shake, ready-to-drink (Slim Fast)	10000_Beverages	62	79	-17	4	4.5	B
92101903	Coffee, Latte, with non-dairy milk	10000_Beverages	62	74	-12	3	4	C
64104030	Apple juice, 100%, with calcium added	10000_Beverages	61	67	-6	1	2.5	D
92101810	Coffee, macchiato	10000_Beverages	61	57	4	1	4	C

95105000	Nutritional drink or shake, ready-to-drink (Kellogg's Special K Protein)	10000_Beverages	60	78	-18	4	4.5	B
64116020	Grape juice, 100%	10000_Beverages	60	63	-3	1	1.5	E
64116050	Grape juice, NS as to sweetened or unsweetened, with added vitamin C	10000_Beverages	60	63	-3	1	0.5	E
64116060	Grape juice, 100%, with calcium added	10000_Beverages	60	63	-3	1	1.5	E
64116100	Grape juice, unsweetened, with added vitamin C	10000_Beverages	60	63	-3	1	0.5	E
92102500	Coffee, Iced Latte	10000_Beverages	60	56	4	1	4.5	B
92161000	Coffee, Cappuccino	10000_Beverages	60	56	4	1	4.5	B
92162000	Coffee, Cappuccino, decaffeinated	10000_Beverages	60	56	4	1	4.5	B
78101120	Fruit and vegetable smoothie, bottled	10000_Beverages	59	75	-16	4	3.5	D
92552100	Orange-cranberry juice drink, low calorie, with vitamin C added	10000_Beverages	59	68	-9	4	2.5	B
64104010	Apple juice, 100%	10000_Beverages	59	65	-6	1	2.5	D
64100220	Cranberry juice blend, 100% juice, with calcium added	10000_Beverages	58	63	-5	1	2.5	D
64100100	Fruit juice, NFS	10000_Beverages	58	62	-4	1	1	E
92101610	Coffee, espresso	10000_Beverages	58	53	5	1	4	B
92101630	Coffee, espresso, decaffeinated	10000_Beverages	58	53	5	1	4	B
92101900	Coffee, Latte	10000_Beverages	58	53	5	1	4.5	B
92101910	Coffee, Latte, decaffeinated	10000_Beverages	58	53	5	1	4.5	B
95120050	Nutritional drink or shake, liquid, soy-based	10000_Beverages	56	66	-10	4	4.5	E
64104090	Apple juice with added vitamin C and calcium	10000_Beverages	56	61	-5	1	1	E
64104200	Apple-pear juice	10000_Beverages	56	61	-5	1	1	D
64100110	Fruit juice blend, 100% juice	10000_Beverages	56	60	-4	1	2.5	E
64126000	Pomegranate juice, 100%	10000_Beverages	56	60	-4	1	2	E
92306090	Tea, hibiscus	10000_Beverages	56	51	5	1	1	D
64101010	Apple cider	10000_Beverages	55	60	-5	1	1.5	D
64104050	Apple juice, with added vitamin C	10000_Beverages	55	60	-5	1	1	D
64100200	Cranberry juice blend, 100% juice	10000_Beverages	55	58	-3	1	2.5	E
64116010	Grape juice, NFS	10000_Beverages	55	58	-3	1	0.5	E
61210230	Orange juice, canned, bottled or in a carton, with sugar	10000_Beverages	54	65	-11	3	1	E
92101906	Coffee, Latte, with non-dairy milk, flavored	10000_Beverages	54	64	-10	3	3.5	D
64104450	Apple-raspberry juice	10000_Beverages	54	60	-6	1	1.5	D
64105500	Cranberry-white grape juice mixture, unsweetened	10000_Beverages	54	56	-2	1	0.5	E
61213000	Tangerine juice, NFS	10000_Beverages	53	62	-9	3	1	E
95120020	Nutritional drink or shake, high protein, light, ready-to-drink, NFS	10000_Beverages	53	62	-9	4	4.5	E
11561000	Cafe con leche	10000_Beverages	53	49	4	1	4.5	B
64104500	Apple-grape juice	10000_Beverages	52	56	-4	1	0.5	E
92101918	Coffee, Latte, decaffeinated, nonfat, flavored	10000_Beverages	52	52	0	1	4	C
95101000	Nutritional drink or shake, ready-to-drink (Boost)	10000_Beverages	51	65	-14	4	4	E
41440020	Ensure with fiber, liquid	10000_Beverages	51	63	-12	4	4.5	C

92102505	Coffee, Iced Latte, with non-dairy milk, flavored	10000_Beverages	51	62	-11	3	3.5	C
64120010	Papaya juice, 100%	10000_Beverages	51	52	-1	1	1.5	E
11611000	Instant breakfast, fluid, canned	10000_Beverages	51	51	0	3	3	E
11612000	Instant breakfast, powder, milk added	10000_Beverages	51	51	0	3	3	E
11551050	Licuado or Batido	10000_Beverages	51	50	1	1	3.5	E
92520810	Grape drink, low calorie	10000_Beverages	50	57	-7	4	2.5	B
92550210	Cranberry-apple juice drink, low calorie, with vitamin C added	10000_Beverages	50	57	-7	4	2.5	B
92101905	Coffee, Latte, nonfat, flavored	10000_Beverages	50	49	1	1	4	C
64134200	Fruit smoothie, bottled	10000_Beverages	49	63	-14	4	3.5	E
95101010	Nutritional drink or shake, ready-to-drink (Boost Plus)	10000_Beverages	49	62	-13	4	4.5	E
92520410	Fruit drink, low calorie	10000_Beverages	49	56	-7	4	2.5	B
92801000	Wine, nonalcoholic	10000_Beverages	49	53	-4	4	3	B
92802000	Wine, light, nonalcoholic	10000_Beverages	49	53	-4	4	3	B
92432000	Fruit juice drink, citrus, carbonated	10000_Beverages	47	64	-17	4	2	D
92550350	Orange juice beverage, 40-50% juice, light	10000_Beverages	47	64	-17	4	2.5	B
11553130	Fruit smoothie juice drink, with dairy	10000_Beverages	47	51	-4	4	3	E
41440010	Ensure liquid nutrition	10000_Beverages	46	56	-10	4	4	D
92101850	Coffee, cafe con leche	10000_Beverages	46	43	3	1	4.5	C
92550300	Grapefruit juice drink, low calorie, with vitamin C added	10000_Beverages	45	52	-7	4	2.5	B
92101851	Coffee, cafe con leche, decaffeinated	10000_Beverages	45	43	2	1	4.5	C
41430200	Meal replacement or supplement, soy- and milk-base, powder, reconstituted with water	10000_Beverages	44	51	-7	4	4.5	C
92101917	Coffee, Latte, decaffeinated, flavored	10000_Beverages	44	41	3	1	4	C
92101904	Coffee, Latte, flavored	10000_Beverages	42	39	3	1	4	C
95120000	Nutritional drink or shake, ready-to-drink, NFS	10000_Beverages	41	48	-7	4	3.5	E
78101000	Vegetable and fruit juice, 100% juice, with high vitamin C	10000_Beverages	41	40	1	1	2.5	E
92102503	Coffee, Iced Latte, flavored	10000_Beverages	41	40	1	1	4	C
41440100	Meal replacement or supplement, liquid, soy-based	10000_Beverages	40	48	-8	4	4.5	B
92551700	Juice drink, low calorie	10000_Beverages	40	47	-7	4	2	B
92510650	Tamarind drink	10000_Beverages	40	42	-2	1	0.5	E
92162005	Cappuccino, decaffeinated, sweetened	10000_Beverages	40	37	3	2	4	C
42403010	Coconut water, unsweetened	10000_Beverages	40	31	9	1	2	C
92101820	Coffee, macchiato, sweetened	10000_Beverages	39	40	-1	1	3.5	D
92431000	Carbonated juice drink, NS as to type of juice	10000_Beverages	38	51	-13	4	2	C
92101955	Coffee, Cafe Mocha, nonfat	10000_Beverages	38	39	-1	2	3.5	D
92101970	Coffee, Cafe Mocha, decaffeinated, nonfat	10000_Beverages	38	39	-1	2	3.5	D
95103000	Nutritional drink or shake, ready-to-drink (Ensure)	10000_Beverages	37	48	-11	4	3.5	E
11623000	Meal supplement or replacement, commercially prepared, ready-to-drink	10000_Beverages	37	47	-10	4	3.5	E
92511250	Fruit juice beverage, 40-50% juice, citrus	10000_Beverages	37	46	-9	4	0.5	E

92510720	Fruit punch, made with fruit juice and soda	10000_Beverages	36	42	-6	2	0.5	E
11553000	Fruit smoothie drink, made with fruit or fruit juice and dairy products	10000_Beverages	36	39	-3	3	0.5	E
92161005	Cappuccino, sweetened	10000_Beverages	36	32	4	2	4	C
95342000	Fruit juice, acai blend	10000_Beverages	35	50	-15	4	0.5	E
92101960	Coffee, Cafe Mocha, with non-dairy milk	10000_Beverages	35	49	-14	4	3.5	E
92433000	Fruit juice drink, noncitrus, carbonated	10000_Beverages	35	46	-11	4	1.5	D
92550380	Pomegranate juice beverage, 40-50% juice, light	10000_Beverages	35	43	-8	4	0.5	E
92511290	Papaya juice drink	10000_Beverages	35	42	-7	4	0.5	E
92306800	Tea, hot, chai, with milk	10000_Beverages	35	37	-2	1	0.5	D
64122030	Peach juice, with sugar	10000_Beverages	34	48	-14	3	0.5	E
64116030	Grape juice, with sugar	10000_Beverages	33	46	-13	3	0.5	E
92101975	Coffee, Cafe Mocha, decaffeinated, with non-dairy milk	10000_Beverages	33	46	-13	4	3.5	E
11641000	Meal supplement or replacement, milk-based, high protein, liquid	10000_Beverages	33	42	-9	4	3.5	E
11641020	Meal replacement or supplement, milk based, ready-to-drink	10000_Beverages	33	42	-9	4	3.5	E
92550200	Grape juice drink, light	10000_Beverages	32	42	-10	4	2	B
92101950	Coffee, Cafe Mocha	10000_Beverages	32	31	1	2	3.5	D
92101965	Coffee, Cafe Mocha, decaffeinated	10000_Beverages	32	31	1	2	3.5	D
92610110	Coconut beverage, Puerto Rican	10000_Beverages	32	31	1	1	0.5	E
95103010	Nutritional drink or shake, ready-to-drink (Ensure Plus)	10000_Beverages	31	40	-9	4	4	E
92550050	Apple-white grape juice drink, low calorie, with vitamin C added	10000_Beverages	31	39	-8	4	2	B
95102000	Nutritional drink or shake, ready-to-drink (Carnation Instant Breakfast)	10000_Beverages	31	39	-8	4	3	E
92307510	Iced Tea / Lemonade juice drink, light	10000_Beverages	30	50	-20	4	2.5	B
92610030	Horchata beverage, made with milk	10000_Beverages	30	27	3	1	2.5	E
28400000	Gelatin drink, powder, unflavored, unsweetened, reconstituted	10000_Beverages	30	19	11	2	3	B
28401200	Gelatin drink, powder, flavored, with low-calorie sweetener, reconstituted	10000_Beverages	30	19	11	2	3	B
92102602	Coffee, Iced Cafe Mocha, with non-dairy milk	10000_Beverages	29	43	-14	4	3.5	D
92511280	Orange-grape-banana juice drink	10000_Beverages	29	42	-13	4	1	D
92610020	Horchata beverage, made with water	10000_Beverages	29	26	3	1	3	E
41440050	Ensure Plus liquid nutrition	10000_Beverages	28	39	-11	4	2.5	E
95312900	Energy drink (XS)	10000_Beverages	28	29	-1	4	3.5	B
95312905	Energy drink (XS Gold Plus)	10000_Beverages	28	29	-1	4	3.5	B
92101800	Coffee, Cuban	10000_Beverages	27	33	-6	2	3.5	D
95312600	Energy drink, sugar-free (Red Bull)	10000_Beverages	27	32	-5	4	3.5	B
92121030	Coffee, mocha, instant, pre-lightened and pre-sweetened with low calorie sweetener, reconstituted	10000_Beverages	27	30	-3	4	4	B
92121050	Coffee, mocha, instant, decaffeinated, pre-lightened and pre-sweetened with low calorie sweetener, reconstituted	10000_Beverages	27	30	-3	4	4	B
92102600	Coffee, Iced Cafe Mocha	10000_Beverages	27	27	0	3	3.5	D
92611600	Horchata beverage, NFS	10000_Beverages	26	29	-3	3	0.5	E

92650005	Red Bull Energy Drink, sugar-free	10000_Beverages	26	29	-3	4	3.5	B
92130030	Coffee, pre-sweetened with low calorie sweetener	10000_Beverages	26	27	-1	4	4	B
92130031	Coffee, decaffeinated, pre-sweetened with low calorie sweetener	10000_Beverages	26	27	-1	4	4	B
92550360	Apple juice beverage, 40-50% juice, light	10000_Beverages	25	36	-11	4	2	B
11631000	High calorie beverage, canned or powdered, reconstituted	10000_Beverages	25	33	-8	4	3	E
92511340	Pineapple-orange juice drink	10000_Beverages	24	35	-11	4	0.5	E
92101923	Frozen coffee drink, with non-dairy milk	10000_Beverages	24	29	-5	2	2.5	E
92101921	Frozen coffee drink, nonfat	10000_Beverages	24	27	-3	2	2.5	E
92611100	Oatmeal beverage with milk	10000_Beverages	24	26	-2	2	2.5	E
92552000	Fruit flavored drink, made from powdered mix, low calorie, with high vitamin C	10000_Beverages	24	25	-1	4	3.5	B
95312560	Energy drink (Ocean Spray Cran-Energy Juice Drink)	10000_Beverages	24	25	-1	4	2	B
11561010	Cafe con leche prepared with sugar	10000_Beverages	24	23	1	1	3.5	D
64201500	Banana nectar	10000_Beverages	23	36	-13	4	0.5	E
92510730	Fruit punch, made with soda, fruit juice, and sherbet or ice cream	10000_Beverages	23	32	-9	3	0.5	E
92171000	Coffee, bottled/canned	10000_Beverages	23	31	-8	4	3.5	E
92510200	Apple-orange-pineapple juice drink	10000_Beverages	23	30	-7	4	0.5	E
92101931	Frozen coffee drink, decaffeinated, nonfat	10000_Beverages	23	27	-4	2	2.5	E
92512090	Pina Colada, nonalcoholic	10000_Beverages	23	26	-3	2	0.5	E
92102010	Frozen mocha coffee drink, nonfat	10000_Beverages	22	26	-4	3	3	E
92613510	Cornmeal beverage with chocolate milk	10000_Beverages	22	23	-1	2	1.5	E
92511270	Orange-peach juice drink	10000_Beverages	21	37	-16	4	0.5	E
92550030	Fruit juice drink, with high vitamin C, light	10000_Beverages	21	36	-15	4	2	B
92550110	Cranberry juice drink, with high vitamin C, light	10000_Beverages	21	36	-15	4	2	B
64203020	Guava nectar	10000_Beverages	21	34	-13	4	0.5	E
94210300	Water, bottled, flavored (SoBe Life Water)	10000_Beverages	21	23	-2	4	4.5	B
92510950	Guava juice drink	10000_Beverages	20	35	-15	4	0.5	E
92530840	Guava juice drink with vitamin C added	10000_Beverages	20	35	-15	4	0.5	E
92102020	Frozen mocha coffee drink, with non-dairy milk	10000_Beverages	20	28	-8	3	3	E
92306100	Corn beverage	10000_Beverages	20	5	15	1	3.5	C
92611010	Oatmeal beverage with water	10000_Beverages	19	22	-3	2	3	E
92101926	Frozen coffee drink, nonfat, with whipped cream	10000_Beverages	19	20	-1	2	3	E
92101936	Frozen coffee drink, decaffeinated, nonfat, with whipped cream	10000_Beverages	19	20	-1	2	3	E
92613010	Cornmeal beverage	10000_Beverages	19	19	0	2	2.5	E
92101920	Frozen coffee drink	10000_Beverages	18	20	-2	2	2.5	E
92101925	Frozen coffee drink, with whipped cream	10000_Beverages	18	19	-1	2	2.5	E
92101930	Frozen coffee drink, decaffeinated	10000_Beverages	18	19	-1	2	2.5	E
92101935	Frozen coffee drink, decaffeinated, with whipped cream	10000_Beverages	18	19	-1	2	2.5	E
92102030	Frozen mocha coffee drink, with whipped cream	10000_Beverages	18	18	0	3	2.5	E

92102090	Frozen mocha coffee drink, decaffeinated, with whipped cream	10000_Beverages	18	18	0	3	2.5	E
92101600	Coffee, Turkish	10000_Beverages	17	23	-6	2	3.5	D
92102000	Frozen mocha coffee drink	10000_Beverages	17	18	-1	2	3	E
92102040	Frozen mocha coffee drink, nonfat, with whipped cream	10000_Beverages	17	18	-1	3	3	E
92307500	Iced Tea / Lemonade juice drink	10000_Beverages	16	36	-20	4	1.5	D
92531020	Orange breakfast drink, made from frozen concentrate	10000_Beverages	15	30	-15	4	0.5	E
92130011	Coffee, decaffeinated, pre-lightened	10000_Beverages	15	16	-1	4	4	B
64204010	Mango nectar	10000_Beverages	14	27	-13	4	0.5	E
92510820	Grape juice drink	10000_Beverages	14	25	-11	4	0.5	E
92308530	Tea, iced, brewed, green, decaffeinated, pre-sweetened with sugar	10000_Beverages	14	21	-7	3	1	D
92102050	Frozen mocha coffee drink, with non-dairy milk and whipped cream	10000_Beverages	14	20	-6	3	2.5	E
92510170	Apple-cranberry-grape juice drink	10000_Beverages	14	19	-5	4	0.5	D
92511260	Orange-cranberry juice drink	10000_Beverages	14	19	-5	4	1	D
42404010	Coconut water, sweetened	10000_Beverages	14	15	-1	3	0.5	C
92121041	Coffee, instant, decaffeinated, pre-lightened and pre-sweetened with low calorie sweetener, reconstituted	10000_Beverages	14	15	-1	4	4	B
92130010	Coffee, pre-lightened	10000_Beverages	14	15	-1	4	4	B
92309010	Tea, iced, bottled, black, decaffeinated	10000_Beverages	14	15	-1	1	0.5	D
92611510	Horchata beverage, made with rice	10000_Beverages	14	15	-1	2	0.5	E
92121040	Coffee, instant, pre-lightened and pre-sweetened with low calorie sweetener, reconstituted	10000_Beverages	13	14	-1	4	4	B
92130005	Coffee, pre-lightened and pre-sweetened with low calorie sweetener	10000_Beverages	13	14	-1	4	4	B
92130006	Coffee, decaffeinated, pre-lightened and pre-sweetened with low calorie sweetener	10000_Beverages	13	14	-1	4	4	B
64200100	Fruit nectar, NFS	10000_Beverages	12	27	-15	4	0.5	E
92308000	Tea, iced, brewed, black, pre-sweetened with sugar	10000_Beverages	12	19	-7	3	1	D
92308030	Tea, iced, brewed, black, decaffeinated, pre-sweetened with sugar	10000_Beverages	12	19	-7	3	1	D
92530950	Vegetable and fruit juice drink, with high vitamin C	10000_Beverages	12	14	-2	4	2	C
64213010	Passion fruit nectar	10000_Beverages	11	27	-16	4	0.5	E
64201010	Apricot nectar	10000_Beverages	11	25	-14	4	0.5	E
92171010	Coffee, bottled/canned, light	10000_Beverages	11	18	-7	4	3.5	C
92308500	Tea, iced, brewed, green, pre-sweetened with sugar	10000_Beverages	10	17	-7	3	1	D
92204000	Mate, sweetened beverage made from dried green leaves	10000_Beverages	10	15	-5	2	2	C
64210010	Papaya nectar	10000_Beverages	9	24	-15	4	0.5	E
92121020	Coffee, mocha, instant, pre-lightened and pre-sweetened with sugar, reconstituted	10000_Beverages	9	17	-8	4	4	C
95312410	Energy drink, sugar free (Monster)	10000_Beverages	8	11	-3	4	3	B
64215010	Pear nectar	10000_Beverages	7	21	-14	4	0.5	E
92511200	Orange-mango juice drink	10000_Beverages	7	21	-14	4	0.5	E
92530610	Fruit juice drink, with high vitamin C	10000_Beverages	7	21	-14	4	0.5	E
64202010	Cantaloupe nectar	10000_Beverages	6	21	-15	4	0.5	E

92582110	Fruit juice drink, added calcium (Sunny D)	10000_Beverages	5	20	-15	4	0.5	E
64221010	Soursop, nectar	10000_Beverages	5	15	-10	4	0.5	D
94210100	Water, bottled, flavored (Propel Water)	10000_Beverages	5	7	-2	4	4.5	B
94220100	Propel Zero Water	10000_Beverages	5	7	-2	4	4.5	B
92530310	Cherry drink with vitamin C added	10000_Beverages	4	17	-13	4	0.5	E
92530810	Grapefruit juice drink with vitamin C added	10000_Beverages	4	16	-12	4	0.5	E
92510910	Grapefruit juice drink	10000_Beverages	3	15	-12	4	0.5	E
92511220	Orange drink	10000_Beverages	3	7	-4	4	1	C
92553000	Fruit-flavored thirst quencher beverage, low calorie	10000_Beverages	3	1	2	4	3	B
92542000	Fruit flavored drink, with high vitamin C, powdered, reconstituted	10000_Beverages	2	16	-14	4	0.5	E
92531030	Fruit juice drink (Sunny D)	10000_Beverages	1	14	-13	4	0.5	E
92305000	Tea, made from powdered instant, presweetened, NS as to sweetener	10000_Beverages	1	13	-12	4	1	D
92305040	Tea, iced, instant, black, pre-sweetened with sugar	10000_Beverages	1	13	-12	4	1	D
92510150	Apple juice drink	10000_Beverages	1	12	-11	4	0.5	E
92510410	Black cherry drink	10000_Beverages	1	12	-11	4	0.5	E
92301190	Tea, NS as to type, decaffeinated, presweetened, NS as to sweetener	10000_Beverages	1	11	-10	4	2	C
92302800	Tea, leaf, decaffeinated, presweetened, NS as to sweetener	10000_Beverages	1	11	-10	4	2	C
92511000	Lemonade, frozen concentrate, not reconstituted	10000_Beverages	1	11	-10	4	0.5	E
92302600	Tea, leaf, decaffeinated, presweetened with sugar	10000_Beverages	1	10	-9	4	2	C
94100300	Water, bottled, flavored (Capri Sun Roarin' Waters)	10000_Beverages	1	10	-9	4	2	B
64205010	Peach nectar	10000_Beverages	1	9	-8	4	0.5	E
92530510	Cranberry juice drink, with high vitamin C	10000_Beverages	1	9	-8	4	0.5	E
92301130	Tea, NS as to type, presweetened, NS as to sweetener	10000_Beverages	1	8	-7	4	1.5	D
92510630	Fruit juice drink, NFS	10000_Beverages	1	8	-7	4	0.5	E
92530910	Lemonade with vitamin C added	10000_Beverages	1	8	-7	4	0.5	E
94210200	Water, bottled, flavored (Glaceau Vitamin Water)	10000_Beverages	1	8	-7	4	1.5	C
92130021	Coffee, decaffeinated, pre-sweetened with sugar	10000_Beverages	1	7	-6	4	4	C
92301060	Tea, NS as to type, presweetened with sugar	10000_Beverages	1	7	-6	4	1	D
92301160	Tea, NS as to type, decaffeinated, presweetened with sugar	10000_Beverages	1	7	-6	4	1	D
92302200	Tea, leaf, presweetened with sugar	10000_Beverages	1	7	-6	4	1	D
92302400	Tea, leaf, presweetened, NS as to sweetener	10000_Beverages	1	7	-6	4	1	D
92305800	Tea, made from powdered instant, decaffeinated, presweetened, NS as to sweetener	10000_Beverages	1	6	-5	4	2.5	C
92531010	Orange drink and orangeade with vitamin C added	10000_Beverages	1	5	-4	4	0.5	E
95310700	Energy drink (Rockstar)	10000_Beverages	1	5	-4	4	0.5	E
92510810	Grapeade and grape drink	10000_Beverages	1	4	-3	4	1	C
95310550	Energy drink (No Fear)	10000_Beverages	1	4	-3	4	0.5	E
95310555	Energy drink (No Fear Motherload)	10000_Beverages	1	4	-3	4	0.5	E
92121010	Coffee, instant, pre-sweetened with sugar, reconstituted	10000_Beverages	1	3	-2	4	4	C

92130020	Coffee, pre-sweetened with sugar	10000_Beverages	1	3	-2	4	4	C
92550370	Lemonade, fruit juice drink, light	10000_Beverages	1	3	-2	4	2	B
95312400	Energy drink, low calorie (Monster)	10000_Beverages	1	3	-2	4	3	B
92102450	Iced Coffee, pre-lightened and pre-sweetened	10000_Beverages	1	2	-1	4	4	C
92305050	Tea, iced, instant, black, decaffeinated, pre-sweetened with sugar	10000_Beverages	1	2	-1	4	1	D
92306020	Tea, herbal, presweetened with sugar	10000_Beverages	1	2	-1	4	2	C
92306040	Tea, herbal, presweetened, NS as to sweetener	10000_Beverages	1	2	-1	4	2	C
92309000	Tea, iced, bottled, black	10000_Beverages	1	2	-1	4	0.5	E
92511110	Limeade	10000_Beverages	1	2	-1	4	0.5	E
92511240	Orange-lemon drink	10000_Beverages	1	2	-1	4	0.5	E
92530520	Cranberry-apple juice drink with vitamin C added	10000_Beverages	1	2	-1	4	0.5	E
92552030	Fruit juice drink (Capri Sun)	10000_Beverages	1	2	-1	4	0.5	E
75200700	Aloe vera juice drink	10000_Beverages	1	1	0	1	2	B
92121000	Coffee, instant, pre-lightened and pre-sweetened with sugar, reconstituted	10000_Beverages	1	1	0	4	4	C
92121001	Coffee, instant, decaffeinated, pre-lightened and pre-sweetened with sugar, reconsitituted	10000_Beverages	1	1	0	4	4	C
92130000	Coffee, pre-lightened and pre-sweetened with sugar	10000_Beverages	1	1	0	4	4	C
92130001	Coffee, decaffeinated, pre-lightened and pre-sweetened with sugar	10000_Beverages	1	1	0	4	4	C
92305910	Tea, iced, instant, green, pre-sweetened with sugar	10000_Beverages	1	1	0	4	1.5	C
92309500	Tea, iced, bottled, green	10000_Beverages	1	1	0	4	1.5	B
92400000	Soft drink, NFS	10000_Beverages	1	1	0	4	0.5	E
92410110	Carbonated water, sweetened	10000_Beverages	1	1	0	4	1	D
92410310	Soft drink, cola	10000_Beverages	1	1	0	4	0.5	E
92410315	Soft drink, cola, reduced sugar	10000_Beverages	1	1	0	4	2	B
92410330	Soft drink, cola-type, with higher caffeine	10000_Beverages	1	1	0	4	0.5	E
92410340	Soft drink, cola, decaffeinated	10000_Beverages	1	1	0	4	0.5	E
92410360	Soft drink, pepper type	10000_Beverages	1	1	0	4	0.5	E
92410390	Soft drink, pepper type, decaffeinated	10000_Beverages	1	1	0	4	0.5	E
92410410	Soft drink, cream soda	10000_Beverages	1	1	0	4	0.5	E
92410510	Soft drink, fruit flavored, caffeine free	10000_Beverages	1	1	0	4	0.5	E
92410550	Soft drink, fruit flavored, caffeine containing	10000_Beverages	1	1	0	4	0.5	E
92410610	Soft drink, ginger ale	10000_Beverages	1	1	0	4	1	D
92410710	Soft drink, root beer	10000_Beverages	1	1	0	4	0.5	E
92410810	Soft drink, chocolate flavored	10000_Beverages	1	1	0	4	0.5	E
92411510	Soft drink, cola, fruit or vanilla flavored	10000_Beverages	1	1	0	4	0.5	E
92411520	Soft drink, cola, chocolate flavored	10000_Beverages	1	1	0	4	0.5	E
92416010	Mavi drink	10000_Beverages	1	1	0	4	0.5	E
92417010	Soft drink, ale type	10000_Beverages	1	1	0	4	0.5	D
92510120	Apple-cherry drink	10000_Beverages	1	1	0	4	0.5	E

92510610	Fruit juice drink	10000_Beverages	1	1	0	4	2.5	C
92510955	Lemonade, fruit juice drink	10000_Beverages	1	1	0	4	0.5	E
92510960	Lemonade, fruit flavored drink	10000_Beverages	1	1	0	4	1.5	D
92511010	Fruit flavored drink (formerly lemonade)	10000_Beverages	1	1	0	4	0.5	E
92511015	Fruit flavored drink	10000_Beverages	1	1	0	4	0.5	E
92511400	Raspberry-flavored drink	10000_Beverages	1	1	0	4	0.5	E
92511510	Strawberry-flavored drink	10000_Beverages	1	1	0	4	2	E
92512040	Frozen daiquiri mix, frozen concentrate, not reconstituted	10000_Beverages	1	1	0	4	0.5	E
92512050	Frozen daiquiri mix, from frozen concentrate, reconstituted	10000_Beverages	1	1	0	4	0.5	D
92512110	Margarita mix, nonalcoholic	10000_Beverages	1	1	0	4	0.5	E
92513000	Slush frozen drink	10000_Beverages	1	1	0	4	1	D
92513010	Slush frozen drink, no sugar added	10000_Beverages	1	1	0	4	2.5	B
92530410	Fruit flavored drink, with high vitamin C	10000_Beverages	1	1	0	4	1.5	C
92530710	Grape drink with vitamin C added	10000_Beverages	1	1	0	4	0.5	E
92541010	Fruit flavored drink, powdered, reconstituted	10000_Beverages	1	1	0	4	1.5	D
92541020	Lemonade-flavored drink, made from powdered mix, with sugar	10000_Beverages	1	1	0	4	0.5	E
92541100	Apple cider-flavored drink, made from powdered mix, with sugar and vitamin C added	10000_Beverages	1	1	0	4	1	D
92544000	Fruit-flavored drink, made from unsweetened powdered mix (fortified with vitamin C), with sugar added in preparation	10000_Beverages	1	1	0	4	0.5	E
92550035	Fruit juice drink, light	10000_Beverages	1	1	0	4	0.5	E
92550610	Fruit flavored drink, low calorie, with high vitamin C	10000_Beverages	1	1	0	4	0.5	D
92560000	Fruit-flavored thirst quencher beverage	10000_Beverages	1	1	0	4	1.5	C
92560100	Gatorade Thirst Quencher sports drink	10000_Beverages	1	1	0	4	1.5	C
92560200	Powerade sports drink	10000_Beverages	1	1	0	4	1	D
92565100	Gatorade G2 thirst quencher sports drink, low calorie	10000_Beverages	1	1	0	4	3	B
92570100	Fluid replacement, electrolyte solution	10000_Beverages	1	1	0	4	2.5	C
92570500	Fluid replacement, 5% glucose in water	10000_Beverages	1	1	0	4	2	C
92582100	Fruit juice drink, with high vitamin C, plus added calcium	10000_Beverages	1	1	0	4	0.5	E
92582120	Fruit flavored drink, reduced sugar, with high vitamin C, plus added calcium	10000_Beverages	1	1	0	4	1.5	C
92612010	Sugar cane beverage	10000_Beverages	1	1	0	4	0.5	E
92650000	Red Bull Energy Drink	10000_Beverages	1	1	0	4	0.5	E
92650100	Full Throttle Energy Drink	10000_Beverages	1	1	0	4	0.5	E
92650200	Monster Energy Drink	10000_Beverages	1	1	0	4	0.5	E
92650205	Mountain Dew AMP Energy Drink	10000_Beverages	1	1	0	4	0.5	E
92650700	Rockstar Energy Drink	10000_Beverages	1	1	0	4	0.5	E
92650800	Vault Energy Drink	10000_Beverages	1	1	0	4	0.5	E
92651000	Energy drink	10000_Beverages	1	1	0	4	0.5	E
92731000	Fruit-flavored drink, non-carbonated, made from powdered mix, with sugar	10000_Beverages	1	1	0	4	1	D

92803000	Nonalcoholic malt beverage	10000_Beverages	1	1	0	4	0.5	E
92804000	Shirley Temple	10000_Beverages	1	1	0	4	0.5	E
95310200	Energy drink (Full Throttle)	10000_Beverages	1	1	0	4	0.5	E
95310400	Energy drink (Monster)	10000_Beverages	1	1	0	4	0.5	E
95310500	Energy drink (Mountain Dew AMP)	10000_Beverages	1	1	0	4	0.5	E
95310560	Energy drink (NOS)	10000_Beverages	1	1	0	4	0.5	E
95310600	Energy drink (Red Bull)	10000_Beverages	1	1	0	4	0.5	E
95310750	Energy drink (SoBe Energize Energy Juice Drink)	10000_Beverages	1	1	0	4	0.5	E
95310800	Energy drink (Vault)	10000_Beverages	1	1	0	4	0.5	E
92651000	Energy Drink	10000_Beverages	1	1	0	4	0.5	E
95320200	Sports drink (Gatorade G)	10000_Beverages	1	1	0	4	1.5	C
95320500	Sports drink (Powerade)	10000_Beverages	1	1	0	4	1	D
95321000	Sports drink, NFS	10000_Beverages	1	1	0	4	1.5	C
95322200	Sports drink, low calorie (Gatorade G2)	10000_Beverages	1	1	0	4	3	B
95323000	Sports drink, low calorie	10000_Beverages	1	1	0	4	3	B
92570100	Fluid replacement, electrolyte solution	10000_Beverages	1	1	0	4	2.5	C
92570500	Fluid replacement, 5% glucose in water	10000_Beverages	1	1	0	4	2	C
61104010	Grapefruit and orange sections, raw	2000_Fruit	100	100	0	1	5	A
61113010	Lemon, raw	2000_Fruit	100	100	0	1	5	A
61116010	Lime, raw	2000_Fruit	100	100	0	1	5	A
61119010	Orange, raw	2000_Fruit	100	100	0	1	5	A
61125000	Tangelo, raw	2000_Fruit	100	100	0	1	5	A
63103010	Apricot, raw	2000_Fruit	100	100	0	1	5	A
63103120	Apricot, cooked or canned, unsweetened, water pack	2000_Fruit	100	100	0	1	5	A
63109010	Cantaloupe, raw	2000_Fruit	100	100	0	1	5	A
63109700	Starfruit, raw	2000_Fruit	100	100	0	1	5	A
63110010	Cassaba melon, raw	2000_Fruit	100	100	0	1	5	A
63125010	Guava, raw	2000_Fruit	100	100	0	1	5	A
63131010	Nectarine, raw	2000_Fruit	100	100	0	1	5	A
63133010	Papaya, raw	2000_Fruit	100	100	0	1	5	A
63135120	Peach, cooked or canned, unsweetened, water pack	2000_Fruit	100	100	0	1	5	A
63147010	Rhubarb, raw	2000_Fruit	100	100	0	1	5	A
63147120	Rhubarb, cooked or canned, unsweetened	2000_Fruit	100	100	0	1	5	A
63200100	Berries, NFS	2000_Fruit	100	100	0	1	4.5	A
63201010	Blackberries, raw	2000_Fruit	100	100	0	1	5	A
63201600	Blackberries, frozen	2000_Fruit	100	100	0	1	5	A
63205010	Boysenberries, raw	2000_Fruit	100	100	0	1	5	A
63205600	Boysenberries, frozen	2000_Fruit	100	100	0	1	5	A
63207010	Cranberries, raw	2000_Fruit	100	100	0	1	5	A

63215010	Loganberries, raw	2000_Fruit	100	100	0	1	5	A
63217010	Mulberries, raw	2000_Fruit	100	100	0	1	5	A
63219000	Raspberries, raw	2000_Fruit	100	100	0	1	5	A
63219020	Raspberries, red, raw	2000_Fruit	100	100	0	1	5	A
63219120	Raspberries, cooked or canned, unsweetened, water pack	2000_Fruit	100	100	0	1	5	A
63219610	Raspberries, frozen	2000_Fruit	100	100	0	1	5	A
63223020	Strawberries, raw	2000_Fruit	100	100	0	1	5	A
63223120	Strawberries, cooked or canned, unsweetened, water pack	2000_Fruit	100	100	0	1	5	A
63223610	Strawberries, frozen	2000_Fruit	100	100	0	1	4.5	A
63311180	Fruit mixture, frozen	2000_Fruit	100	100	0	1	4	A
63126500	Kiwi fruit, raw	2000_Fruit	100	99	1	1	5	A
63137050	Pear, Asian, raw	2000_Fruit	100	96	4	1	5	A
61122350	Orange, mandarin, canned or frozen, drained	2000_Fruit	99	100	-1	3	4	A
61101010	Grapefruit, raw	2000_Fruit	99	99	0	1	5	A
63149010	Watermelon, raw	2000_Fruit	98	100	-2	1	5	A
63311050	Fruit salad, fresh or raw, including citrus fruits, no dressing	2000_Fruit	98	98	0	1	5	A
61110010	Kumquat, raw	2000_Fruit	98	92	6	1	5	A
63200200	Berries, frozen, NFS	2000_Fruit	97	98	-1	1	4.5	A
63203570	Blueberries, frozen, NS as to sweetened or unsweetened	2000_Fruit	97	98	-1	1	4.5	A
63203600	Blueberries, frozen	2000_Fruit	97	98	-1	1	4.5	A
63135620	Peach, frozen	2000_Fruit	97	97	0	1	4.5	A
63135010	Peach, raw	2000_Fruit	97	96	1	1	5	A
63311080	Fruit cocktail or mix, frozen	2000_Fruit	97	96	1	1	4.5	A
63134010	Passion fruit, raw	2000_Fruit	97	89	8	1	5	A
62114000	Lychee, dried	2000_Fruit	96	98	-2	1	3	D
63203010	Blueberries, raw	2000_Fruit	96	96	0	1	5	A
63214000	Huckleberries, raw	2000_Fruit	96	96	0	1	5	A
63311000	Fruit salad, fresh or raw, excluding citrus fruits, no dressing	2000_Fruit	96	96	0	1	5	A
63101000	Apple, raw	2000_Fruit	95	94	1	1	5	A
62104100	Apricot, dried	2000_Fruit	94	94	0	1	3.5	D
63143010	Plum, raw	2000_Fruit	94	94	0	1	5	A
61125010	Tangerine, raw	2000_Fruit	94	93	1	1	5	A
63100100	Fruit, NFS	2000_Fruit	94	93	1	1	4.5	A
62116100	Peach, dried	2000_Fruit	93	92	1	1	4	C
62116220	Peach, dried, cooked, unsweetened	2000_Fruit	93	91	2	1	3.5	C
63101320	Apple, baked, unsweetened	2000_Fruit	93	91	2	1	5	A
63129050	Mango, frozen	2000_Fruit	92	93	-1	1	4	A
63141200	Pineapple, frozen	2000_Fruit	92	92	0	1	4	A
63127010	Honeydew melon, raw	2000_Fruit	91	93	-2	1	5	A

63127610	Honeydew, frozen	2000_Fruit	91	93	-2	1	4	A
63141010	Pineapple, raw	2000_Fruit	91	91	0	1	5	A
63129010	Mango, raw	2000_Fruit	90	91	-1	1	5	A
63141120	Pineapple, cooked or canned, unsweetened, waterpack	2000_Fruit	90	90	0	1	5	A
62104220	Apricot, dried, cooked, unsweetened	2000_Fruit	90	88	2	1	3.5	C
63133050	Papaya, green, cooked	2000_Fruit	89	88	1	1	3.5	A
63139010	Persimmon, raw	2000_Fruit	89	86	3	1	5	A
63137010	Pear, raw	2000_Fruit	89	84	5	1	5	A
63123020	Grapes, American type, slip skin, raw	2000_Fruit	88	89	-1	1	5	A
63113110	Cherries, sour, red, cooked, unsweetened	2000_Fruit	88	86	2	1	5	A
61100600	Clementine, raw	2000_Fruit	87	85	2	1	5	A
63115010	Cherries, raw	2000_Fruit	87	85	2	1	5	A
63115200	Cherries, frozen	2000_Fruit	87	85	2	1	4	A
63203120	Blueberries, cooked or canned, unsweetened, water pack	2000_Fruit	87	85	2	1	5	A
62113100	Fig, dried	2000_Fruit	87	84	3	1	4	C
63137120	Pear, cooked or canned, unsweetened, water pack	2000_Fruit	86	81	5	1	5	A
61122320	Orange, canned, juice pack	2000_Fruit	85	92	-7	3	3.5	A
63126510	Lychee	2000_Fruit	85	85	0	1	4	A
62101100	Apple, dried	2000_Fruit	85	82	3	1	3.5	D
62113220	Fig, dried, cooked, unsweetened	2000_Fruit	85	81	4	1	3.5	A
61119020	Orange, sections, canned, juice pack	2000_Fruit	84	88	-4	3	3.5	A
63123000	Grapes, raw	2000_Fruit	84	84	0	1	5	A
63123010	Grapes, European type, adherent skin, raw	2000_Fruit	84	84	0	1	5	A
62122100	Prune, dried	2000_Fruit	84	82	2	1	4	C
63107010	Banana, raw	2000_Fruit	83	81	2	1	5	A
63107050	Banana, white, Guineo blanco maduro	2000_Fruit	83	81	2	1	4.5	A
63107070	Banana, Chinese, raw	2000_Fruit	83	81	2	1	5	A
62101220	Apple, dried, cooked, unsweetened	2000_Fruit	83	80	3	1	4	B
63145010	Pomegranate, raw	2000_Fruit	83	79	4	1	5	A
63403030	Fruit salad, including citrus fruits, with nondairy whipped topping	2000_Fruit	82	90	-8	2	4	C
61101220	Grapefruit, canned or frozen, unsweetened, water pack	2000_Fruit	82	82	0	1	5	A
63126700	Longans, raw	2000_Fruit	82	81	1	1	5	A
63115120	Cherries, sweet, cooked, unsweetened, water pack	2000_Fruit	82	80	2	1	5	B
62122220	Prune, dried, cooked, unsweetened	2000_Fruit	82	79	3	1	3.5	C
63223030	Strawberries, raw, with sugar	2000_Fruit	81	87	-6	1	4.5	B
63320100	Fruit salad, Puerto Rican style	2000_Fruit	81	81	0	2	4	A
62119100	Pear, dried	2000_Fruit	81	78	3	1	3.5	D
63107310	Banana, ripe, boiled	2000_Fruit	81	78	3	1	4.5	B
63403020	Fruit salad, including citrus fruit, with whipped cream	2000_Fruit	80	85	-5	2	4	B

63101120	Applesauce, unsweetened	2000_Fruit	80	77	3	1	3.5	B
63119010	Fig, raw	2000_Fruit	80	75	5	1	5	A
63402970	Fruit salad, excluding citrus fruits, with nondairy whipped topping	2000_Fruit	79	86	-7	2	4	B
63103170	Apricot, cooked or canned, juice pack	2000_Fruit	79	82	-3	3	3.5	A
62108100	Currants, dried	2000_Fruit	79	77	2	1	3	D
63401030	Apple and fruit salad with dressing	2000_Fruit	79	76	3	1	4.5	A
63402960	Fruit salad, excluding citrus fruits, with whipped cream	2000_Fruit	77	82	-5	2	4	B
61101200	Grapefruit, canned	2000_Fruit	77	79	-2	3	3.5	A
62110100	Date	2000_Fruit	77	73	4	1	3.5	C
63135170	Peach, canned, juice pack	2000_Fruit	76	80	-4	3	4	A
63401010	Apple salad with dressing	2000_Fruit	76	80	-4	2	4	B
63402990	Fruit salad, including citrus fruits, with pudding	2000_Fruit	76	80	-4	2	4	A
63403010	Fruit salad, including citrus fruits, with salad dressing or mayonnaise	2000_Fruit	75	81	-6	3	4	B
62101300	Apple chips	2000_Fruit	75	80	-5	3	3	D
63413010	Pineapple salad with dressing	2000_Fruit	75	80	-5	3	4	B
63141150	Pineapple, cooked or canned, drained solids	2000_Fruit	75	76	-1	3	4	B
63403000	Fruit salad, excluding citrus fruits, with pudding	2000_Fruit	74	77	-3	2	4	A
61122300	Orange, canned, NFS	2000_Fruit	74	76	-2	3	3.5	A
63115170	Cherries, sweet, cooked or canned, juice pack	2000_Fruit	74	76	-2	3	3.5	A
63107210	Banana, ripe, fried	2000_Fruit	74	75	-1	1	4	B
63402045	Fried dwarf banana, Puerto Rican style	2000_Fruit	74	75	-1	2	4	A
62114110	Papaya, dried	2000_Fruit	74	71	3	1	2.5	D
63402950	Fruit salad, excluding citrus fruits, with salad dressing or mayonnaise	2000_Fruit	73	78	-5	3	3.5	B
63141170	Pineapple, canned, juice pack	2000_Fruit	73	77	-4	3	3.5	B
62125100	Raisins	2000_Fruit	73	70	3	1	3	D
62120100	Pineapple, dried	2000_Fruit	72	69	3	1	2.5	D
63301010	Ambrosia	2000_Fruit	72	69	3	1	4	A
63129030	Mango, canned	2000_Fruit	70	67	3	1	3.5	A
63412010	Pear salad with dressing	2000_Fruit	69	73	-4	3	4	B
63148750	Tamarind	2000_Fruit	69	65	4	1	4	A
62105000	Blueberries, dried	2000_Fruit	68	76	-8	3	3.5	C
63403040	Fruit salad, including citrus fruits, with marshmallows	2000_Fruit	68	74	-6	3	3.5	C
63201800	Blackberries, frozen, sweetened, NS as to type of sweetener	2000_Fruit	68	73	-5	3	4	C
63141110	Pineapple, canned, NFS	2000_Fruit	68	71	-3	3	3.5	C
63137170	Pear, canned, juice pack	2000_Fruit	68	70	-2	3	3.5	C
63143170	Plum, cooked or canned, juice pack	2000_Fruit	68	70	-2	3	3.5	B
62101050	Fruit mixture, dried	2000_Fruit	68	67	1	3	3	D
62120000	Persimmon, dried	2000_Fruit	68	67	1	3	2.5	C

63311120	Fruit cocktail, cooked or canned, unsweetened, water pack	2000_Fruit	67	73	-6	4	5	A
62104200	Apricot, dried, cooked, NS as to sweetened or unsweetened; sweetened, NS as to type of sweetener	2000_Fruit	67	66	1	1	3.5	C
63402980	Fruit salad, excluding citrus fruits, with marshmallows	2000_Fruit	66	72	-6	3	3.5	C
63115150	Cherries, sweet, cooked or canned, drained solids	2000_Fruit	66	67	-1	3	3.5	A
63107110	Banana, baked	2000_Fruit	66	65	1	1	4	C
62119200	Pear, dried, cooked, NS as to sweetened or unsweetened; sweetened, NS as to type of sweetener	2000_Fruit	65	62	3	1	3.5	C
63133100	Papaya, canned	2000_Fruit	64	70	-6	3	4	A
62116230	Peach, dried, cooked, with sugar	2000_Fruit	64	66	-2	1	3.5	C
62122200	Prune, dried, cooked, NS as to sweetened or unsweetened; sweetened, NS as to type of sweetener	2000_Fruit	64	62	2	1	3	C
61122330	Orange, canned, in syrup	2000_Fruit	63	65	-2	3	3	A
62119230	Pear, dried, cooked, with sugar	2000_Fruit	63	61	2	1	3.5	C
63119110	Fig, canned	2000_Fruit	63	60	3	3	3.5	C
63401015	Apple and grape salad with yogurt and walnuts	2000_Fruit	62	74	-12	4	4.5	A
62104230	Apricot, dried, cooked, with sugar	2000_Fruit	62	64	-2	1	3.5	C
62126000	Tamarind, dried	2000_Fruit	62	60	2	3	3.5	C
62122230	Prune, dried, cooked, with sugar	2000_Fruit	60	60	0	1	3	C
63219600	Raspberries, frozen, NS as to added sweetener	2000_Fruit	59	67	-8	3	4	A
63219620	Raspberries, frozen, with sugar	2000_Fruit	59	67	-8	3	4	A
63101140	Applesauce, stewed apples, sweetened with low calorie sweetener	2000_Fruit	59	65	-6	4	3.5	A
63103150	Apricot, cooked or canned, drained solids	2000_Fruit	59	64	-5	3	3.5	B
63115140	Cherries, sweet, cooked or canned, in light syrup	2000_Fruit	59	63	-4	3	3.5	A
63143110	Plum, canned	2000_Fruit	59	59	0	3	3.5	C
63101330	Apple, baked, with sugar	2000_Fruit	58	61	-3	1	4	B
63125100	Guava shell, canned in heavy syrup	2000_Fruit	57	62	-5	3	3.5	C
63203110	Bluberries, canned	2000_Fruit	57	61	-4	3	3	C
63119140	Figs, cooked or canned, in light syrup	2000_Fruit	57	57	0	3	3.5	A
62121100	Plum, rock salt, dried	2000_Fruit	57	53	4	3	0.5	E
63103110	Apricot, canned	2000_Fruit	56	64	-8	3	3.5	A
63203550	Blueberries, frozen, sweetened	2000_Fruit	56	64	-8	3	4	C
63135110	Peach, canned, NFS	2000_Fruit	56	62	-6	3	3.5	B
63115130	Cherries, sweet, cooked or canned, in heavy syrup	2000_Fruit	56	57	-1	3	3	C
63201110	Blackberries, cooked or canned, NS as to sweetened or unsweetened; sweetened, NS as to type of sweetener	2000_Fruit	55	62	-7	3	3.5	C
63131110	Nectarine, cooked	2000_Fruit	55	60	-5	2	4	B
63401020	Apple and cabbage salad with dressing	2000_Fruit	54	60	-6	3	4.5	A
62101200	Apple, dried, cooked, NS as to sweetened or unsweetened; sweetened, NS as to type of sweetener	2000_Fruit	54	54	0	1	3.5	C
63311170	Fruit cocktail, canned, juice pack	2000_Fruit	53	62	-9	4	3.5	A
63115110	Cherries, canned	2000_Fruit	53	57	-4	3	3	C

63135150	Peach, cooked or canned, drained solids	2000_Fruit	53	56	-3	3	3.5	A
63137150	Pear, cooked or canned, drained solids	2000_Fruit	53	54	-1	3	4	C
63147140	Rhubarb, cooked or canned, in light syrup	2000_Fruit	52	62	-10	3	3.5	B
62101000	Fruit, dried, NFS, uncooked	2000_Fruit	52	58	-6	1	2.5	C
63101110	Applesauce, regular	2000_Fruit	52	54	-2	3	3.5	C
63101130	Applesauce, stewed apples, with sugar	2000_Fruit	52	54	-2	3	3.5	C
63101410	Apple rings, fried	2000_Fruit	52	51	1	2	4	A
63135140	Peach, canned, in syrup	2000_Fruit	51	58	-7	3	3.5	A
63219110	Raspberries, cooked or canned, NS as to sweetened or unsweetened; sweetened, NS as to type of sweetener	2000_Fruit	51	58	-7	3	3.5	B
63219130	Raspberries, cooked or canned, in heavy syrup	2000_Fruit	51	58	-7	3	3.5	B
61101230	Grapefruit, canned or frozen, in light syrup	2000_Fruit	51	56	-5	3	3	A
63137110	Pear, canned, NFS	2000_Fruit	51	54	-3	3	3.5	A
63103140	Apricot, cooked or canned, in light syrup	2000_Fruit	50	58	-8	3	3.5	B
63141140	Pineapple, canned, in syrup	2000_Fruit	50	55	-5	3	3	A
62101230	Apple, dried, cooked, with sugar	2000_Fruit	50	52	-2	1	3.5	B
63101310	Apple, baked	2000_Fruit	50	48	2	2	4	B
62109100	Cranberries, dried	2000_Fruit	49	58	-9	3	2.5	C
63135610	Peach, frozen, NS as to added sweetener	2000_Fruit	49	56	-7	3	3.5	C
63135630	Peach, frozen, with sugar	2000_Fruit	49	56	-7	3	3.5	C
63119130	Fig, cooked or canned, in heavy syrup	2000_Fruit	49	52	-3	3	3	B
62125110	Raisins, cooked	2000_Fruit	49	50	-1	1	3	D
63223600	Strawberries, frozen, NS as to added sweetener	2000_Fruit	48	58	-10	3	4	C
63223620	Strawberries, frozen, with sugar	2000_Fruit	48	58	-10	3	4	C
63126600	Lychee, cooked or canned, in sugar or syrup	2000_Fruit	48	51	-3	3	3	C
63147110	Rhubarb	2000_Fruit	47	56	-9	3	3.5	A
62114050	Mango, dried	2000_Fruit	47	51	-4	3	2.5	D
63137140	Pear, canned, in syrup	2000_Fruit	46	50	-4	3	3.5	A
63401990	Banana, chocolate-covered with nuts	2000_Fruit	46	45	1	3	3	C
63223110	Strawberries, canned	2000_Fruit	45	55	-10	3	4	C
63223130	Strawberries, cooked or canned, in syrup	2000_Fruit	45	55	-10	3	4	B
63141130	Pineapple, cooked or canned, in heavy syrup	2000_Fruit	45	50	-5	3	3	A
63203130	Blueberries, cooked or canned, in heavy syrup	2000_Fruit	44	51	-7	3	3	C
63401070	Fruit, chocolate covered	2000_Fruit	44	49	-5	3	3.5	C
63143140	Plum, cooked or canned, in light syrup	2000_Fruit	44	45	-1	3	3	B
63103130	Apricot, cooked or canned, in heavy syrup	2000_Fruit	43	51	-8	3	3	B
63107410	Banana, fried	2000_Fruit	43	39	4	1	4	C
63411010	Cranberry salad, congealed	2000_Fruit	42	59	-17	4	3.5	C
63123110	Grapes, seedless, cooked or canned, NS as to sweetened or unsweetened; sweetened, NS as to type of sweetener	2000_Fruit	42	46	-4	3	3	A

63135660	Peach, spiced	2000_Fruit	41	49	-8	3	3	A
63137130	Pear, cooked or canned, in heavy syrup	2000_Fruit	41	44	-3	3	3.5	A
62106000	Cherries, dried	2000_Fruit	40	48	-8	3	2.5	D
63143150	Plum, cooked or canned, drained solids	2000_Fruit	40	44	-4	3	3.5	B
63147600	Rhubarb, frozen, NS as to sweetened or unsweetened; sweetened, NS as to type of sweetener	2000_Fruit	38	48	-10	3	3	A
63147620	Rhubarb, frozen, with sugar	2000_Fruit	38	48	-10	3	3	C
63129020	Mango, pickled	2000_Fruit	38	46	-8	3	3	C
63135130	Peach, cooked or canned, in heavy syrup	2000_Fruit	38	46	-8	3	3	A
63100110	Fruit, pickled	2000_Fruit	38	37	1	3	2.5	D
63101420	Apple, pickled	2000_Fruit	37	44	-7	3	3.5	C
63101210	Apple pie filling	2000_Fruit	37	31	6	3	3.5	A
63147130	Rhubarb, cooked or canned, in heavy syrup	2000_Fruit	36	45	-9	3	3	C
63101150	Applesauce, flavored	2000_Fruit	36	44	-8	4	3.5	B
63311150	Fruit cocktail, cooked or canned, drained solids	2000_Fruit	35	47	-12	4	3.5	A
63311110	Fruit cocktail, canned, NFS	2000_Fruit	35	45	-10	4	3	A
63401060	Apple, candied	2000_Fruit	35	37	-2	3	4	C
63101500	Apple, fried	2000_Fruit	35	33	2	2	4	C
63143650	Plum, pickled	2000_Fruit	34	42	-8	3	3	C
62107200	Banana chips	2000_Fruit	33	39	-6	4	1.5	D
63311140	Fruit cocktail, canned, in syrup	2000_Fruit	32	42	-10	4	3	A
63143130	Plum, cooked or canned, in heavy syrup	2000_Fruit	32	37	-5	3	3	C
63311145	Tropical fruit cocktail, cooked or canned, in light syrup	2000_Fruit	30	39	-9	4	3.5	A
63307010	Cranberry-orange relish, uncooked	2000_Fruit	28	43	-15	4	3	C
63311130	Fruit cocktail, cooked or canned, in heavy syrup	2000_Fruit	25	37	-12	4	3	A
63403100	Fruit dessert with cream and/or pudding and nuts	2000_Fruit	19	25	-6	3	2	D
63307100	Cranberry-raspberry Sauce	2000_Fruit	17	30	-13	4	3	C
63111010	Cherries, maraschino	2000_Fruit	14	25	-11	4	3	C
63207000	Cranberries, NS as to raw, cooked, or canned	2000_Fruit	14	25	-11	4	3.5	C
63207110	Cranberry sauce	2000_Fruit	14	25	-11	4	3	C
72101100	Beet greens, raw	3000_Vegetables	100	100	0	1	5	A
72101200	Beet greens, cooked, NS as to fat added in cooking	3000_Vegetables	100	100	0	2	4	B
72101210	Beet greens, cooked, fat not added in cooking	3000_Vegetables	100	100	0	1	4	C
72101220	Beet greens, cooked	3000_Vegetables	100	100	0	2	4	B
72101230	Beet greens, cooked, made with oil	3000_Vegetables	100	100	0	2	4	A
72101250	Beet greens, cooked, made with margarine	3000_Vegetables	100	100	0	2	4	C
72103000	Broccoli raab, raw	3000_Vegetables	100	100	0	1	5	A
72103020	Broccoli raab, cooked, fat not added in cooking	3000_Vegetables	100	100	0	1	4	A
72103030	Broccoli raab, cooked	3000_Vegetables	100	100	0	2	4.5	A

72103040	Broccoli raab, cooked, made with oil	3000_Vegetables	100	100	0	2	4	A
72103060	Broccoli raab, cooked, made with margarine	3000_Vegetables	100	100	0	2	4	A
72104100	Chard, raw	3000_Vegetables	100	100	0	1	5	A
72104200	Chard, cooked, NS as to fat added in cooking	3000_Vegetables	100	100	0	2	4	A
72104210	Chard, cooked, fat not added in cooking	3000_Vegetables	100	100	0	1	4	B
72104220	Chard, cooked	3000_Vegetables	100	100	0	2	4	B
72104230	Chard, cooked, made with oil	3000_Vegetables	100	100	0	2	4	B
72104250	Chard, cooked, made with margarine	3000_Vegetables	100	100	0	2	4	C
72107100	Collards, raw	3000_Vegetables	100	100	0	1	5	A
72107210	Collards, cooked, NS as to form, fat not added in cooking	3000_Vegetables	100	100	0	1	5	A
72107211	Collards, fresh, cooked, no added fat	3000_Vegetables	100	100	0	1	5	A
72107212	Collards, frozen, cooked, no added fat	3000_Vegetables	100	100	0	1	4	A
72107213	Collards, canned, cooked, no added fat	3000_Vegetables	100	100	0	1	4	A
72107220	Collards, NS as to form, cooked	3000_Vegetables	100	100	0	2	5	A
72107221	Collards, fresh, cooked, fat added, NS as to fat type	3000_Vegetables	100	100	0	2	5	A
72107222	Collards, frozen, cooked, fat added, NS as to fat type	3000_Vegetables	100	100	0	2	4	A
72107224	Collards, cooked, NS as to form, made with oil	3000_Vegetables	100	100	0	1	5	A
72107226	Collards, cooked, NS as to form, made with margarine	3000_Vegetables	100	100	0	2	5	A
72107227	Collards, fresh, cooked with oil	3000_Vegetables	100	100	0	2	5	A
72107229	Collards, cooked, from fresh, made with margarine	3000_Vegetables	100	100	0	2	5	A
72107230	Collards, frozen, cooked with oil	3000_Vegetables	100	100	0	2	4	A
72107232	Collards, cooked, from frozen, made with margarine	3000_Vegetables	100	100	0	2	4	A
72107233	Collards, canned, cooked with oil	3000_Vegetables	100	100	0	2	4	A
72107235	Collards, cooked, from canned, made with margarine	3000_Vegetables	100	100	0	2	4	A
72110100	Cress, raw	3000_Vegetables	100	100	0	1	5	A
72110210	Cress, cooked, NS as to form, fat not added in cooking	3000_Vegetables	100	100	0	1	4	A
72110211	Cress, cooked, from fresh, fat not added in cooking	3000_Vegetables	100	100	0	1	4	A
72110213	Cress, cooked, from canned, fat not added in cooking	3000_Vegetables	100	100	0	1	4	A
72110221	Cress, cooked	3000_Vegetables	100	100	0	2	4.5	A
72110230	Cress, cooked, NS as to form, made with oil	3000_Vegetables	100	100	0	2	4	A
72110232	Cress, cooked, NS as to form, made with margarine	3000_Vegetables	100	100	0	2	4	A
72110233	Cress, cooked, from fresh, made with oil	3000_Vegetables	100	100	0	2	4	A
72110236	Cress, cooked, from canned, made with oil	3000_Vegetables	100	100	0	2	4	A
72110238	Cress, cooked, from canned, made with margarine	3000_Vegetables	100	100	0	2	4	A
72113100	Dandelion greens, raw	3000_Vegetables	100	100	0	1	5	A
72113210	Dandelion greens, cooked, fat not added in cooking	3000_Vegetables	100	100	0	1	4.5	A
72113220	Dandelion greens, cooked	3000_Vegetables	100	100	0	1	4.5	A
72113230	Dandelion greens, cooked, made with oil	3000_Vegetables	100	100	0	2	4.5	A
72113250	Dandelion greens, cooked, made with margarine	3000_Vegetables	100	100	0	2	4.5	A

72116000	Romaine lettuce, raw	3000_Vegetables	100	100	0	1	5	A
72116200	Escarole, cooked, NS as to fat added in cooking	3000_Vegetables	100	100	0	2	4.5	A
72116210	Escarole, cooked, fat not added in cooking	3000_Vegetables	100	100	0	1	4.5	A
72116220	Escarole, cooked	3000_Vegetables	100	100	0	2	4.5	A
72118210	Greens, cooked, NS as to form, fat not added in cooking	3000_Vegetables	100	100	0	1	4	A
72118211	Greens, fresh, cooked, no added fat	3000_Vegetables	100	100	0	1	4.5	A
72118212	Greens, frozen, cooked, no added fat	3000_Vegetables	100	100	0	1	4.5	A
72118213	Greens, canned, cooked, no added fat	3000_Vegetables	100	100	0	1	4	A
72118220	Greens, NS as to form, cooked	3000_Vegetables	100	100	0	2	4.5	A
72118221	Greens, fresh, cooked, fat added	3000_Vegetables	100	100	0	2	4.5	A
72118222	Greens, frozen, cooked, fat added	3000_Vegetables	100	100	0	2	4.5	A
72118223	Greens, canned, cooked, fat added	3000_Vegetables	100	100	0	2	4	B
72118224	Greens, cooked, NS as to form, made with oil	3000_Vegetables	100	100	0	2	4.5	A
72118226	Greens, cooked, NS as to form, made with margarine	3000_Vegetables	100	100	0	2	4	A
72118227	Greens, cooked, from fresh, made with oil	3000_Vegetables	100	100	0	2	4.5	A
72118229	Greens, cooked, from fresh, made with margarine	3000_Vegetables	100	100	0	2	4	A
72118230	Greens, cooked, from frozen, made with oil	3000_Vegetables	100	100	0	2	4	A
72118232	Greens, cooked, from frozen, made with margarine	3000_Vegetables	100	100	0	2	4.5	A
72118233	Greens, cooked, from canned, made with oil	3000_Vegetables	100	100	0	2	4.5	A
72118235	Greens, cooked, from canned, made with margarine	3000_Vegetables	100	100	0	2	4	A
72118300	Chamnamul, cooked, NS as to fat added in cooking	3000_Vegetables	100	100	0	2	4	B
72118305	Chamnamul, cooked, fat not added in cooking	3000_Vegetables	100	100	0	1	4	A
72119190	Kale, raw	3000_Vegetables	100	100	0	1	5	A
72119210	Kale, cooked, NS as to form, fat not added in cooking	3000_Vegetables	100	100	0	1	4.5	B
72119211	Kale, fresh, cooked, no added fat	3000_Vegetables	100	100	0	1	5	A
72119212	Kale, frozen, cooked, no added fat	3000_Vegetables	100	100	0	1	4	A
72119213	Kale, canned, cooked, no added fat	3000_Vegetables	100	100	0	1	4	B
72119220	Kale, NS as to form, cooked	3000_Vegetables	100	100	0	2	5	A
72119221	Kale, fresh, cooked, fat added	3000_Vegetables	100	100	0	2	5	A
72119222	Kale, frozen, cooked, fat added	3000_Vegetables	100	100	0	2	4	A
72119223	Kale, canned, cooked, fat added	3000_Vegetables	100	100	0	2	4	A
72119224	Kale, cooked, NS as to form, made with oil	3000_Vegetables	100	100	0	2	4.5	A
72119226	Kale, cooked, NS as to form, made with margarine	3000_Vegetables	100	100	0	2	4.5	A
72119227	Kale, cooked, from fresh, made with oil	3000_Vegetables	100	100	0	2	4.5	A
72119230	Kale, cooked, from frozen, made with oil	3000_Vegetables	100	100	0	2	4	A
72119233	Kale, cooked, from canned, made with oil	3000_Vegetables	100	100	0	2	4	A
72120210	Lambsquarter, cooked, fat not added in cooking	3000_Vegetables	100	100	0	1	4	A
72120220	Lambsquarter, cooked	3000_Vegetables	100	100	0	2	4	A
72120230	Lambsquarter, cooked, made with oil	3000_Vegetables	100	100	0	1	4	A

72121210	Mustard cabbage, cooked, fat not added in cooking	3000_Vegetables	100	100	0	1	4	A
72121211	Mustard cabbage, cooked	3000_Vegetables	100	100	0	2	4	A
72122100	Mustard greens, raw	3000_Vegetables	100	100	0	1	5	A
72122210	Mustard greens, cooked, NS as to form, fat not added in cooking	3000_Vegetables	100	100	0	1	4	A
72122211	Mustard greens, fresh, cooked, no added fat	3000_Vegetables	100	100	0	1	4.5	A
72122212	Mustard greens, frozen, cooked, no added fat	3000_Vegetables	100	100	0	1	4.5	A
72122213	Mustard greens, canned, cooked, no added fat	3000_Vegetables	100	100	0	1	4	A
72122220	Mustard greens, NS as to form, cooked	3000_Vegetables	100	100	0	2	4.5	A
72122221	Mustard greens, fresh, cooked, fat added	3000_Vegetables	100	100	0	2	4.5	A
72122222	Mustard greens, frozen, cooked, fat added	3000_Vegetables	100	100	0	2	4.5	A
72122223	Mustard greens, canned, cooked, fat added	3000_Vegetables	100	100	0	2	4	A
72122224	Mustard greens, cooked, NS as to form, made with oil	3000_Vegetables	100	100	0	2	4	A
72122226	Mustard greens, cooked, NS as to form, made with margarine	3000_Vegetables	100	100	0	2	4	A
72122227	Mustard greens, cooked, from fresh, made with oil	3000_Vegetables	100	100	0	2	4	A
72122229	Mustard greens, cooked, from fresh, made with margarine	3000_Vegetables	100	100	0	2	4	A
72122230	Mustard greens, cooked, from frozen, made with oil	3000_Vegetables	100	100	0	2	4	A
72122232	Mustard greens, cooked, from frozen, made with margarine	3000_Vegetables	100	100	0	2	4	B
72122233	Mustard greens, cooked, from canned, made with oil	3000_Vegetables	100	100	0	2	4	A
72122235	Mustard greens, cooked, from canned, made with margarine	3000_Vegetables	100	100	0	2	4	A
72123010	Poke greens, cooked, fat not added in cooking	3000_Vegetables	100	100	0	1	4	A
72123020	Poke greens, cooked	3000_Vegetables	100	100	0	2	4	A
72123030	Poke greens, cooked, made with oil	3000_Vegetables	100	100	0	2	4	A
72123050	Poke greens, cooked, made with margarine	3000_Vegetables	100	100	0	2	4	B
72124100	Radicchio, raw	3000_Vegetables	100	100	0	1	5	A
72125100	Spinach, raw	3000_Vegetables	100	100	0	1	5	A
72125200	Spinach, cooked, NS as to form, NS as to fat added in cooking	3000_Vegetables	100	100	0	2	4	A
72125201	Spinach, cooked, from fresh, NS as to fat added in cooking	3000_Vegetables	100	100	0	2	4	A
72125202	Spinach, cooked, from frozen, NS as to fat added in cooking	3000_Vegetables	100	100	0	2	4	A
72125203	Spinach, cooked, from canned, NS as to fat added in cooking	3000_Vegetables	100	100	0	3	4	B
72125211	Spinach, fresh, cooked, no added fat	3000_Vegetables	100	100	0	1	4.5	A
72125212	Spinach, frozen, cooked, no added fat	3000_Vegetables	100	100	0	1	4.5	A
72125213	Spinach, canned, cooked, no added fat	3000_Vegetables	100	100	0	3	4	A
72125216	Spinach, cooked, NS as to form, made with margarine	3000_Vegetables	100	100	0	2	4.5	A
72125217	Spinach, fresh, cooked with oil	3000_Vegetables	100	100	0	2	4.5	A
72125218	Spinach, fresh, cooked with butter or margarine	3000_Vegetables	100	100	0	2	4	A
72125219	Spinach, cooked, from fresh, made with margarine	3000_Vegetables	100	100	0	2	4	A
72125220	Spinach, NS as to form, cooked	3000_Vegetables	100	100	0	2	4.5	A
72125221	Spinach, fresh, cooked, fat added, NS as to fat type	3000_Vegetables	100	100	0	2	4.5	A
72125222	Spinach, frozen, cooked, fat added, NS as to fat type	3000_Vegetables	100	100	0	2	4.5	A

72125223	Spinach, canned, cooked, fat added, NS as to fat type	3000_Vegetables	100	100	0	3	4	A
72125224	Spinach, frozen, cooked with oil	3000_Vegetables	100	100	0	2	4.5	A
72125227	Spinach, canned, cooked with oil	3000_Vegetables	100	100	0	3	4	A
72126000	Taro leaves, cooked, fat not added in cooking	3000_Vegetables	100	100	0	1	4.5	A
72126001	Taro leaves, cooked	3000_Vegetables	100	100	0	2	5	A
72127000	Thistle leaves, cooked, fat not added in cooking	3000_Vegetables	100	100	0	1	4	A
72127001	Thistle leaves, cooked	3000_Vegetables	100	100	0	2	4	A
72128210	Turnip greens, cooked, NS as to form, fat not added in cooking	3000_Vegetables	100	100	0	1	4.5	A
72128211	Turnip greens, fresh, cooked, no added fat	3000_Vegetables	100	100	0	1	4.5	A
72128212	Turnip greens, frozen, cooked, no added fat	3000_Vegetables	100	100	0	1	4.5	A
72128213	Turnip greens, canned, cooked, no added fat	3000_Vegetables	100	100	0	1	4	A
72128220	Turrnip greens, NS as to form, cooked	3000_Vegetables	100	100	0	2	4.5	A
72128221	Turnip greens, fresh, cooked, fat added	3000_Vegetables	100	100	0	2	4.5	A
72128222	Turnip greens, frozen, cooked, fat added	3000_Vegetables	100	100	0	2	4.5	A
72128226	Turnip greens, cooked, NS as to form, made with margarine	3000_Vegetables	100	100	0	2	4.5	A
72128227	Turnip greens, cooked, from fresh, made with oil	3000_Vegetables	100	100	0	2	4	A
72128229	Turnip greens, cooked, from fresh, made with margarine	3000_Vegetables	100	100	0	2	4	A
72128230	Turnip greens, cooked, from frozen, made with oil	3000_Vegetables	100	100	0	2	4.5	A
72128232	Turnip greens, cooked, from frozen, made with margarine	3000_Vegetables	100	100	0	2	4.5	A
72128233	Turnip greens, cooked, from canned, made with oil	3000_Vegetables	100	100	0	2	4	B
72128235	Turnip greens, cooked, from canned, made with margarine	3000_Vegetables	100	100	0	2	4	A
72128236	Turnip greens, canned, reduced sodium, cooked, NS as to fat added in cooking	3000_Vegetables	100	100	0	2	4	A
72128237	Turnip greens, canned, reduced sodium, cooked, no added fat	3000_Vegetables	100	100	0	1	4	A
72128238	Turnip greens, canned, reduced sodium, cooked, fat added	3000_Vegetables	100	100	0	2	4	A
72128410	Turnip greens with roots, cooked, NS as to form, fat not added in cooking	3000_Vegetables	100	100	0	1	4.5	A
72128411	Turnip greens with roots, cooked, from fresh, fat not added in cooking	3000_Vegetables	100	100	0	1	4	A
72128412	Turnip greens with roots, cooked, from frozen, fat not added in cooking	3000_Vegetables	100	100	0	1	4.5	A
72128413	Turnip greens with roots, cooked, from canned, fat not added in cooking	3000_Vegetables	100	100	0	1	4	A
72128500	Turnip greens, canned, low sodium, cooked, NS as to fat added in cooking	3000_Vegetables	100	100	0	2	4	A
72128510	Turnip greens, canned, low sodium, cooked, fat not added in cooking	3000_Vegetables	100	100	0	1	4	A
72128520	Turnip greens, canned, low sodium, cooked, fat added in cooking	3000_Vegetables	100	100	0	2	4	A
72130100	Watercress, raw	3000_Vegetables	100	100	0	1	5	A
72130200	Watercress, cooked, fat not added in cooking	3000_Vegetables	100	100	0	1	4	A
72130201	Watercress, cooked	3000_Vegetables	100	100	0	2	4.5	A
72132200	Bitter melon leaves, horseradish leaves, jute leaves, or radish leaves, cooked, fat not added in cooking	3000_Vegetables	100	100	0	1	4	B

72132201	Bitter melon, horseradish, jute, or radish leaves, cooked	3000_Vegetables	100	100	0	2	4.5	A
72133200	Sweet potato leaves, squash leaves, pumpkin leaves, chrysanthemum leaves, bean leaves, or swamp cabbage, cooked, fat not added in cooking	3000_Vegetables	100	100	0	1	4	C
72133201	Sweet potato, squash, pumpkin, chrysanthemum, or bean leaves, cooked	3000_Vegetables	100	100	0	2	4.5	A
72201100	Broccoli, raw	3000_Vegetables	100	100	0	1	5	A
72201210	Broccoli, cooked, NS as to form, fat not added in cooking	3000_Vegetables	100	100	0	1	4.5	A
72201211	Broccoli, fresh, cooked, no added fat	3000_Vegetables	100	100	0	1	4.5	A
72201212	Broccoli, frozen, cooked, no added fat	3000_Vegetables	100	100	0	1	4.5	A
72203000	Broccoli, chinese, raw	3000_Vegetables	100	100	0	1	5	A
72203040	Broccoli, chinese, cooked, NS as to form, fat not added in cooking	3000_Vegetables	100	100	0	1	4	A
72203050	Broccoli, chinese cooked, from fresh, fat not added in cooking	3000_Vegetables	100	100	0	1	4	A
72203060	Broccoli, chinese, cooked, from frozen, fat not added in cooking	3000_Vegetables	100	100	0	1	4	A
72203070	Broccoli, Chinese, cooked	3000_Vegetables	100	100	0	2	4.5	A
73103010	Carrots, canned, reduced sodium, cooked, no added fat	3000_Vegetables	100	100	0	1	4	A
73103021	Carrots, canned, reduced sodium, cooked with oil	3000_Vegetables	100	100	0	2	4	A
73111250	Peas and carrots, canned, low sodium, NS as to fat added in cooking	3000_Vegetables	100	100	0	2	5	A
73111260	Peas and carrots, canned, low sodium, fat added in cooking	3000_Vegetables	100	100	0	2	5	A
73111270	Peas and carrots, canned, low sodium, fat not added in cooking	3000_Vegetables	100	100	0	1	5	A
73302010	Winter squash, raw	3000_Vegetables	100	100	0	1	5	A
74101000	Tomatoes, raw	3000_Vegetables	100	100	0	1	5	A
74102000	Tomatoes, green, raw	3000_Vegetables	100	100	0	1	5	A
74202010	Tomatoes, NS as to form, broiled	3000_Vegetables	100	100	0	1	3.5	B
74202011	Tomatoes, from fresh, broiled	3000_Vegetables	100	100	0	1	3.5	B
74206000	Sun-dried tomatoes	3000_Vegetables	100	100	0	1	5	C
74403110	Tomato paste	3000_Vegetables	100	100	0	1	5	A
74403120	Tomato puree	3000_Vegetables	100	100	0	1	4.5	A
74504010	Tomato and okra, cooked, fat not added in cooking	3000_Vegetables	100	100	0	2	4	B
74504021	Tomato and okra, cooked, made with oil	3000_Vegetables	100	100	0	2	4	B
74504150	Tomato and celery, cooked, fat not added in cooking	3000_Vegetables	100	100	0	1	3.5	A
75100250	Raw vegetable, NFS	3000_Vegetables	100	100	0	1	4.5	A
75100300	Sprouts, NFS	3000_Vegetables	100	100	0	1	4.5	A
75100500	Alfalfa sprouts, raw	3000_Vegetables	100	100	0	1	5	A
75100800	Asparagus, raw	3000_Vegetables	100	100	0	1	5	A
75101000	Bean sprouts, raw	3000_Vegetables	100	100	0	1	5	A
75101800	Green beans, raw	3000_Vegetables	100	100	0	1	5	A
75102600	Broccoflower, raw	3000_Vegetables	100	100	0	1	5	A
75102750	Brussels sprouts, raw	3000_Vegetables	100	100	0	1	5	A
75103000	Cabbage, green, raw	3000_Vegetables	100	100	0	1	5	A

75104000	Cabbage, Chinese, raw	3000_Vegetables	100	100	0	1	5	A
75105000	Cabbage, red, raw	3000_Vegetables	100	100	0	1	5	A
75105500	Cactus, raw	3000_Vegetables	100	100	0	1	5	A
75107000	Cauliflower, raw	3000_Vegetables	100	100	0	1	5	A
75109000	Celery, raw	3000_Vegetables	100	100	0	1	5	A
75109010	Fennel bulb, raw	3000_Vegetables	100	100	0	1	5	A
75111000	Cucumber, raw	3000_Vegetables	100	100	0	1	5	A
75111200	Eggplant, raw	3000_Vegetables	100	100	0	1	5	A
75112000	Kohlrabi, raw	3000_Vegetables	100	100	0	1	5	A
75113000	Lettuce, raw	3000_Vegetables	100	100	0	1	5	A
75113060	Lettuce, Boston, raw	3000_Vegetables	100	100	0	1	5	A
75113070	Lettuce, manoa	3000_Vegetables	100	100	0	1	4.5	A
75113080	Lettuce, arugula, raw	3000_Vegetables	100	100	0	1	5	A
75114000	Mixed salad greens, raw	3000_Vegetables	100	100	0	1	5	A
75115000	Mushrooms, raw	3000_Vegetables	100	100	0	1	5	A
75117010	Onions, green, raw	3000_Vegetables	100	100	0	1	5	A
75120000	Green peas, raw	3000_Vegetables	100	100	0	1	5	A
75121400	Pepper, poblano, raw	3000_Vegetables	100	100	0	1	5	A
75121500	Pepper, Serrano, raw	3000_Vegetables	100	100	0	1	5	A
75122000	Pepper, raw, NFS	3000_Vegetables	100	100	0	1	5	A
75122100	Pepper, sweet, green, raw	3000_Vegetables	100	100	0	1	5	A
75122200	Pepper, sweet, red, raw	3000_Vegetables	100	100	0	1	5	A
75124000	Pepper, banana, raw	3000_Vegetables	100	100	0	1	5	A
75125000	Radish, raw	3000_Vegetables	100	100	0	1	5	A
75127000	Rutabaga, raw	3000_Vegetables	100	100	0	1	5	A
75127750	Snowpeas, raw	3000_Vegetables	100	100	0	1	5	A
75128000	Summer squash, yellow, raw	3000_Vegetables	100	100	0	1	5	A
75128010	Summer squash, green, raw	3000_Vegetables	100	100	0	1	5	A
75143000	Lettuce, salad with assorted vegetables including tomatoes and/or carrots, no dressing	3000_Vegetables	100	100	0	1	4.5	A
75143050	Lettuce, salad with assorted vegetables excluding tomatoes and carrots, no dressing	3000_Vegetables	100	100	0	1	4.5	A
75143100	Lettuce, salad with avocado, tomato, and/or carrots, with or without other vegetables, no dressing	3000_Vegetables	100	100	0	1	4.5	A
75202010	Asparagus, cooked, NS as to form, fat not added in cooking	3000_Vegetables	100	100	0	1	4	A
75202011	Asparagus, fresh, cooked, no added fat	3000_Vegetables	100	100	0	1	4	A
75202012	Asparagus, frozen, cooked, no added fat	3000_Vegetables	100	100	0	1	4	A
75202013	Asparagus, canned, cooked, no added fat	3000_Vegetables	100	100	0	3	4	B
75202021	Asparagus, fresh, cooked, fat added, NS as to fat type	3000_Vegetables	100	100	0	2	4	A
75202022	Asparagus, frozen, cooked, fat added, NS as to fat type	3000_Vegetables	100	100	0	2	4	A
75202024	Asparagus, cooked, NS as to form, made with oil	3000_Vegetables	100	100	0	2	4	A

75202027	Asparagus, fresh, cooked with oil	3000_Vegetables	100	100	0	2	4	A
75205021	Green beans, fresh, cooked, no added fat	3000_Vegetables	100	100	0	1	4.5	A
75205120	Green beans, canned, reduced sodium, cooked, no added fat	3000_Vegetables	100	100	0	1	4.5	A
75208300	Bitter melon, cooked, fat not added in cooking	3000_Vegetables	100	100	0	1	4	A
75208710	Broccoflower, cooked, fat not added in cooking	3000_Vegetables	100	100	0	1	4.5	A
75209010	Brussels sprouts, cooked, NS as to form, fat not added in cooking	3000_Vegetables	100	100	0	1	4.5	A
75209011	Brussels sprouts, fresh, cooked, no added fat	3000_Vegetables	100	100	0	1	4.5	A
75209012	Brussels sprouts, frozen, cooked, no added fat	3000_Vegetables	100	100	0	1	4.5	A
75210000	Cabbage, Chinese, cooked, NS as to fat added in cooking	3000_Vegetables	100	100	0	2	4	A
75210010	Cabbage, Chinese, cooked, no added fat	3000_Vegetables	100	100	0	1	4	A
75210021	Cabbage, Chinese, cooked, made with oil	3000_Vegetables	100	100	0	2	4	A
75213010	Cabbage, savoy, cooked, fat not added in cooking	3000_Vegetables	100	100	0	1	4.5	A
75213110	Cactus, cooked, no added fat	3000_Vegetables	100	100	0	1	4	A
75214010	Cauliflower, cooked, NS as to form, fat not added in cooking	3000_Vegetables	100	100	0	1	4.5	A
75214012	Cauliflower, frozen, cooked, no added fat	3000_Vegetables	100	100	0	1	4.5	A
75214013	Cauliflower, cooked, from canned, fat not added in cooking	3000_Vegetables	100	100	0	1	4	A
75214030	Cauliflower, frozen, cooked with oil	3000_Vegetables	100	100	0	2	4.5	A
75215010	Celery, cooked, fat not added in cooking	3000_Vegetables	100	100	0	1	4	A
75217010	Eggplant, cooked, no added fat	3000_Vegetables	100	100	0	1	4.5	A
75217300	Flowers or blossoms of sesbania, squash, or lily, fat not added in cooking	3000_Vegetables	100	100	0	1	4	A
75219010	Mushrooms, cooked, NS as to form, fat not added in cooking	3000_Vegetables	100	100	0	1	4	B
75219011	Mushrooms, fresh, cooked, no added fat	3000_Vegetables	100	100	0	1	4.5	A
75219012	Mushrooms, cooked, from frozen, fat not added in cooking	3000_Vegetables	100	100	0	1	4	B
75219033	Mushrooms, fresh, cooked with oil	3000_Vegetables	100	100	0	2	4.5	A
75220010	Okra, cooked, NS as to form, fat not added in cooking	3000_Vegetables	100	100	0	1	4	B
75220011	Okra, fresh, cooked, no added fat	3000_Vegetables	100	100	0	1	4.5	A
75220012	Okra, frozen, cooked, no added fat	3000_Vegetables	100	100	0	1	4	A
75220013	Okra, cooked, from canned, fat not added in cooking	3000_Vegetables	100	100	0	3	4	B
75220020	Okra, NS as to form, cooked	3000_Vegetables	100	100	0	2	4	A
75220021	Okra, fresh, cooked, fat added	3000_Vegetables	100	100	0	2	4.5	A
75220027	Okra, cooked, from fresh, made with oil	3000_Vegetables	100	100	0	2	4	B
75220050	Lettuce, cooked, fat not added in cooking	3000_Vegetables	100	100	0	1	4.5	A
75220100	Luffa, cooked, fat not added in cooking	3000_Vegetables	100	100	0	1	4	A
75220101	Luffa, cooked	3000_Vegetables	100	100	0	2	4.5	A
75227100	Radish, daikon, cooked, fat not added in cooking	3000_Vegetables	100	100	0	1	4	B
75230010	Sauerkraut, cooked, fat not added in cooking	3000_Vegetables	100	100	0	3	3.5	C
75230100	Sauerkraut, canned, low sodium	3000_Vegetables	100	100	0	3	4	A
75232100	Seaweed, cooked, NS as to fat added in cooking	3000_Vegetables	100	100	0	2	4.5	A

75232110	Seaweed, cooked, no added fat	3000_Vegetables	100	100	0	1	4	A
75232121	Seaweed, cooked, made with oil	3000_Vegetables	100	100	0	2	4.5	A
75233010	Squash, summer, yellow or green, cooked, NS as to form, fat not added in cooking	3000_Vegetables	100	100	0	1	4	A
75233011	Summer squash, yellow or green, fresh, cooked, no added fat	3000_Vegetables	100	100	0	1	4	A
75233012	Summer squash, yellow or green, frozen, cooked, no added fat	3000_Vegetables	100	100	0	1	4	A
75233013	Summer squash, yellow or green, canned, cooked, no added fat	3000_Vegetables	100	100	0	1	3.5	A
75316030	Squash, summer, yellow or green, with tomato sauce, cooked, fat not added in cooking	3000_Vegetables	100	100	0	2	4	A
75330050	Broccoli and cauliflower, cooked, no added fat	3000_Vegetables	100	100	0	1	4.5	A
75330080	Broccoli, cauliflower and carrots, cooked, no added fat	3000_Vegetables	100	100	0	1	4.5	B
75340140	Vegetable combination (green beans, broccoli, onions, mushrooms), cooked, fat not added in cooking	3000_Vegetables	100	100	0	1	4	A
75502520	Kimchi	3000_Vegetables	100	100	0	3	3.5	B
89902010	Cucumber, for use on a sandwich	3000_Vegetables	100	100	0	1	4.5	A
89902020	Lettuce, for use on a sandwich	3000_Vegetables	100	100	0	1	4.5	A
89902030	Mushrooms, for use on a sandwich	3000_Vegetables	100	100	0	1	4.5	A
89902050	Pepper, for use on a sandwich	3000_Vegetables	100	100	0	1	4.5	A
89902060	Spinach, for use on a sandwich	3000_Vegetables	100	100	0	1	5	A
89902070	Tomatoes, for use on a sandwich	3000_Vegetables	100	100	0	1	4.5	A
75143300	Lettuce, salad with egg, tomato, and/or carrots, with or without other vegetables, no dressing	3000_Vegetables	100	99	1	1	4.5	A
75203000	Bamboo shoots, cooked, fat not added in cooking	3000_Vegetables	100	99	1	1	4.5	A
75204993	Beans, string, cooked, from canned, NS as to color, fat not added in cooking	3000_Vegetables	100	99	1	1	4	A
75205022	Green beans, frozen, cooked, no added fat	3000_Vegetables	100	99	1	1	4.5	A
75205023	Green beans, canned, cooked, no added fat	3000_Vegetables	100	99	1	1	4	A
75330110	Vegetable combination, including carrots, broccoli, and/or dark-green leafy; cooked, no sauce, fat not added in cooking	3000_Vegetables	100	99	1	1	4	A
75212010	Cabbage, red, cooked, fat not added in cooking	3000_Vegetables	100	98	2	1	4	A
75100750	Artichoke, raw	3000_Vegetables	100	97	3	1	5	A
75206011	Beans, string, yellow, cooked, from fresh, fat not added in cooking	3000_Vegetables	100	97	3	1	4.5	A
75231012	Snowpea, frozen, cooked, no added fat	3000_Vegetables	100	97	3	1	4.5	A
73101010	Carrots, raw	3000_Vegetables	100	96	4	1	5	A
73112000	Carrot chips, dried	3000_Vegetables	100	96	4	1	5	D
75204990	Beans, string, cooked, NS as to form, NS as to color, fat not added in cooking	3000_Vegetables	100	96	4	1	4.5	A
75205020	Beans, string, green, cooked, NS as to form, fat not added in cooking	3000_Vegetables	100	96	4	1	4.5	A
75221051	Onions, green, cooked, from fresh, fat not added in cooking	3000_Vegetables	100	95	5	1	3.5	C
75127500	Seaweed, raw	3000_Vegetables	100	94	6	1	5	A
75211020	Cabbage, green, cooked, no added fat	3000_Vegetables	100	93	7	1	4	A
72125210	Spinach, cooked, NS as to form, fat not added in cooking	3000_Vegetables	100	100	0	1	4.5	A
72125214	Spinach, cooked, NS as to form, made with oil	3000_Vegetables	100	100	0	2	4	A

72125226	Spinach, cooked, from frozen, made with margarine	3000_Vegetables	100	100	0	2	4	A
72125229	Spinach, cooked, from canned, made with margarine	3000_Vegetables	100	100	0	3	4	C
72107223	Collards, canned, cooked, fat added, NS as to fat type	3000_Vegetables	99	100	-1	2	4	A
72116221	Escarole, cooked, made with oil	3000_Vegetables	99	100	-1	2	4.5	A
72118310	Chamnamul, cooked, fat added in cooking	3000_Vegetables	99	100	-1	2	4	C
72119232	Kale, cooked, from frozen, made with margarine	3000_Vegetables	99	100	-1	2	4	A
72120250	Lambsquarter, cooked, made with margarine	3000_Vegetables	99	100	-1	2	4	A
72201213	Broccoli, cooked, NS as to form, made with oil	3000_Vegetables	99	100	-1	2	4.5	A
72201226	Broccoli, frozen, cooked with oil	3000_Vegetables	99	100	-1	2	4.5	A
74204500	Tomatoes, canned, reduced sodium, cooked	3000_Vegetables	99	100	-1	2	4	A
75202026	Asparagus, cooked, NS as to form, made with margarine	3000_Vegetables	99	100	-1	2	4	A
75202029	Asparagus, cooked, from fresh, made with margarine	3000_Vegetables	99	100	-1	2	4	A
75213121	Cactus, cooked, made with oil	3000_Vegetables	99	100	-1	2	4	A
73201010	Pumpkin, cooked, NS as to form, fat not added in cooking	3000_Vegetables	99	97	2	1	3.5	A
73201011	Pumpkin, cooked, from fresh, fat not added in cooking	3000_Vegetables	99	97	2	1	3.5	A
73201012	Pumpkin, cooked, from frozen, fat not added in cooking	3000_Vegetables	99	97	2	1	3.5	A
75302010	Beans, string, green, with tomatoes, cooked, fat not added in cooking	3000_Vegetables	99	97	2	2	4	A
75231011	Snowpea, fresh, cooked, no added fat	3000_Vegetables	99	96	3	1	4	A
75206012	Beans, string, yellow, cooked, from frozen, fat not added in cooking	3000_Vegetables	99	94	5	1	4	A
75204992	Beans, string, cooked, from frozen, NS as to color, fat not added in cooking	3000_Vegetables	99	93	6	1	4	A
72103010	Broccoli raab, cooked, NS as to fat added in cooking	3000_Vegetables	98	100	-2	2	4	B
72116223	Escarole, cooked, made with margarine	3000_Vegetables	98	100	-2	2	4.5	A
72125225	Spinach, frozen, cooked with butter or margarine	3000_Vegetables	98	100	-2	2	4	A
72125228	Spinach, canned, cooked with butter or margarine	3000_Vegetables	98	100	-2	3	4	A
72203080	Broccoli, chinese, cooked, NS as to form, made with oil	3000_Vegetables	98	100	-2	2	4	A
72203160	Broccoli, chinese, cooked, from frozen, made with oil	3000_Vegetables	98	100	-2	2	4	A
73103023	Carrots, canned, low sodium, made with margarine	3000_Vegetables	98	100	-2	2	4.5	A
75205131	Green beans, canned, reduced sodium, cooked with oil	3000_Vegetables	98	100	-2	2	4.5	A
75210020	Cabbage, Chinese, cooked, fat added	3000_Vegetables	98	100	-2	2	4	A
75214027	Cauliflower, fresh, cooked with oil	3000_Vegetables	98	100	-2	2	4.5	A
75219030	Mushrooms, cooked, NS as to form, made with oil	3000_Vegetables	98	100	-2	2	4	B
75219036	Mushrooms, cooked, from frozen, made with oil	3000_Vegetables	98	100	-2	2	4	B
75511100	Pickles, NFS	3000_Vegetables	98	100	-2	3	3.5	C
72107228	Collards, fresh, cooked with butter or margarine	3000_Vegetables	98	99	-1	2	5	A
72128224	Turnip greens, cooked, NS as to form, made with oil	3000_Vegetables	98	99	-1	2	4.5	A
75302040	Beans, string, green, with almonds, cooked, fat not added in cooking	3000_Vegetables	98	98	0	1	4.5	A
75340010	Asian stir fry vegetables, cooked, no added fat	3000_Vegetables	98	95	3	1	5	A
75214011	Cauliflower, fresh, cooked, no added fat	3000_Vegetables	98	93	5	1	4.5	A

72119229	Kale, cooked, from fresh, made with margarine	3000_Vegetables	97	100	-3	2	4.5	A
72119235	Kale, cooked, from canned, made with margarine	3000_Vegetables	97	100	-3	2	4	A
75112500	Leek, raw	3000_Vegetables	97	100	-3	1	5	A
75211031	Cabbage, green, cooked with oil	3000_Vegetables	97	99	-2	2	4	A
75214024	Cauliflower, cooked, NS as to form, made with oil	3000_Vegetables	97	99	-2	2	4.5	A
74504023	Tomato and okra, cooked, made with margarine	3000_Vegetables	97	98	-1	3	4	B
75232120	Seaweed, cooked, fat added	3000_Vegetables	97	94	3	2	4	A
72110235	Cress, cooked, from fresh, made with margarine	3000_Vegetables	96	100	-4	2	4	A
72128223	Turnip greens, canned, cooked, fat added	3000_Vegetables	96	100	-4	2	4	B
72201223	Broccoli, fresh, cooked with oil	3000_Vegetables	96	100	-4	2	4.5	A
72203140	Broccoli, chinese, cooked, from fresh, made with margarine	3000_Vegetables	96	100	-4	3	4	A
75202020	Asparagus, NS as to form, cooked	3000_Vegetables	96	99	-3	2	4	A
75203020	Bamboo shoots, cooked, fat added in cooking	3000_Vegetables	96	99	-3	2	4	A
72125215	Spinach, cooked, NS as to form, made with butter	3000_Vegetables	96	98	-2	1	4	A
75209030	Brussels sprouts, cooked, NS as to form, made with oil	3000_Vegetables	96	98	-2	1	4.5	A
75209050	Brussels sprouts, cooked, from frozen, made with oil	3000_Vegetables	96	98	-2	1	4.5	A
75330060	Broccoli and cauliflower, cooked, fat added	3000_Vegetables	96	98	-2	2	4.5	A
72118200	Greens, cooked, NS as to form, NS as to fat added in cooking	3000_Vegetables	96	97	-1	2	4	A
72118201	Greens, cooked, from fresh, NS as to fat added in cooking	3000_Vegetables	96	97	-1	2	4	A
72125999	Taro leaves, cooked, NS as to fat added in cooking	3000_Vegetables	96	97	-1	2	4.5	A
72128202	Turnip greens, cooked, from frozen, NS as to fat added in cooking	3000_Vegetables	96	97	-1	2	4.5	A
63105010	Avocado, raw	3000_Vegetables	96	96	0	1	5	A
72101240	Beet greens, cooked, made with butter	3000_Vegetables	96	96	0	1	4	B
72122202	Mustard greens, cooked, from frozen, NS as to fat added in cooking	3000_Vegetables	96	96	0	2	4	A
89902000	Avocado, for use on a sandwich	3000_Vegetables	96	95	1	1	4.5	A
75302020	Beans, string, green, with onions, cooked, fat not added in cooking	3000_Vegetables	96	91	5	1	4	A
72130199	Watercress, cooked, NS as to fat added in cooking	3000_Vegetables	95	100	-5	2	4	A
72203100	Broccoli, chinese, cooked, NS as to form, made with margarine	3000_Vegetables	95	100	-5	3	4	A
72203120	Broccoli, chinese, cooked, from fresh, made with oil	3000_Vegetables	95	100	-5	2	4	A
75202023	Asparagus, canned, cooked, fat added, NS as to fat type	3000_Vegetables	95	100	-5	3	4	B
75205133	Beans, string, green, canned, low sodium, made with margarine	3000_Vegetables	95	100	-5	2	4.5	A
75330121	Vegetable combination, including carrots, broccoli, and/or dark-green leafy; cooked, no sauce, made with oil	3000_Vegetables	95	100	-5	2	4	B
75209040	Brussels sprouts, cooked, from fresh, made with oil	3000_Vegetables	95	99	-4	2	4	A
75330123	Vegetable combination, including carrots, broccoli, and/or dark-green leafy; cooked, no sauce, made with margarine	3000_Vegetables	95	98	-3	2	4	A
72128200	Turnip greens, cooked, NS as to form, NS as to fat added in cooking	3000_Vegetables	95	97	-2	2	4.5	A
72118203	Greens, cooked, from canned, NS as to fat added in cooking	3000_Vegetables	95	96	-1	2	4	A
73201013	Pumpkin, canned, cooked	3000_Vegetables	95	96	-1	2	4	A
75209021	Brussels sprouts, fresh, cooked, fat added	3000_Vegetables	95	96	-1	1	4.5	A

75214032	Cauliflower, cooked, from frozen, made with margarine	3000_Vegetables	95	96	-1	2	4.5	A
72104240	Chard, cooked, made with butter	3000_Vegetables	95	95	0	1	4	C
72128225	Turnip greens, cooked, NS as to form, made with butter	3000_Vegetables	95	95	0	1	4.5	A
73303010	Winter squash, cooked, no added fat	3000_Vegetables	95	93	2	1	4	A
75204991	Beans, string, cooked, from fresh, NS as to color, fat not added in cooking	3000_Vegetables	95	91	4	1	4.5	A
75224120	Green peas, canned, reduced sodium, cooked, no added fat	3000_Vegetables	95	91	4	1	5	A
73102212	Carrots, frozen, cooked, no added fat	3000_Vegetables	95	90	5	1	4.5	A
73111211	Peas and carrots, fresh, cooked, no added fat	3000_Vegetables	95	90	5	1	5	A
72121209	Mustard Cabbage, cooked, NS as to fat added in cooking	3000_Vegetables	94	100	-6	2	4	A
75205044	Green beans, fresh, cooked with oil	3000_Vegetables	94	98	-4	2	4.5	A
75220031	Okra, cooked, from frozen, made with oil	3000_Vegetables	94	97	-3	2	4	B
72118202	Greens, cooked, from frozen, NS as to fat added in cooking	3000_Vegetables	94	96	-2	2	4	A
72201225	Broccoli, cooked, from fresh, made with margarine	3000_Vegetables	94	96	-2	2	4	A
75208720	Broccoflower, cooked	3000_Vegetables	94	96	-2	2	4.5	A
75219038	Mushrooms, cooked, from frozen, made with margarine	3000_Vegetables	94	96	-2	2	4	B
75220033	Okra, cooked, from frozen, made with margarine	3000_Vegetables	94	96	-2	2	4	A
72120200	Lambsquarter, cooked, NS as to fat added in cooking	3000_Vegetables	94	95	-1	2	4	A
72128231	Turnip greens, cooked, from frozen, made with butter	3000_Vegetables	94	95	-1	1	4.5	A
75202001	Asparagus, cooked, from fresh, NS as to fat added in cooking	3000_Vegetables	94	95	-1	2	4	A
75202028	Asparagus, fresh, cooked with butter or margarine	3000_Vegetables	94	95	-1	2	4	A
75219035	Mushrooms, cooked, from fresh, made with margarine	3000_Vegetables	94	95	-1	2	4	B
72128201	Turnip greens, cooked, from fresh, NS as to fat added in cooking	3000_Vegetables	94	94	0	2	4	A
73201033	Pumpkin, cooked, from canned, made with oil	3000_Vegetables	94	94	0	1	4	A
72107200	Collards, cooked, NS as to form, NS as to fat added in cooking	3000_Vegetables	94	93	1	2	5	A
72107201	Collards, cooked, from fresh, NS as to fat added in cooking	3000_Vegetables	94	93	1	2	5	A
72201215	Broccoli, cooked, NS as to form, made with margarine	3000_Vegetables	94	93	1	2	4.5	A
72201228	Broccoli, cooked, from frozen, made with margarine	3000_Vegetables	94	93	1	2	4.5	A
75234012	Turnip, cooked, from frozen, fat not added in cooking	3000_Vegetables	94	92	2	1	4	A
75129000	Turnip, raw	3000_Vegetables	94	91	3	1	5	A
75219013	Mushrooms, cooked, from canned, fat not added in cooking	3000_Vegetables	94	89	5	3	3.5	C
75224021	Green peas, fresh, cooked, no added fat	3000_Vegetables	94	89	5	1	5	A
75201011	Artichoke, fresh, cooked, no added fat	3000_Vegetables	94	87	7	1	4.5	A
75201012	Artichoke, frozen, cooked, no added fat	3000_Vegetables	94	87	7	1	4.5	A
75201013	Artichoke, canned, cooked, no added fat	3000_Vegetables	94	87	7	1	4.5	A
72203180	Broccoli, chinese, cooked, from frozen, made with margarine	3000_Vegetables	93	99	-6	3	4	A
75208290	Bitter melon, cooked, NS as to fat added in cooking	3000_Vegetables	93	98	-5	2	4	A
73103020	Carrots, canned, reduced sodium, cooked, fat added, NS as to fat type	3000_Vegetables	93	97	-4	2	4	A
73201024	Pumpkin, cooked, NS as to form, made with oil	3000_Vegetables	93	97	-4	2	3.5	A

73201030	Pumpkin, cooked, from frozen, made with oil	3000_Vegetables	93	97	-4	2	3.5	A
72107231	Collards, frozen, cooked with butter or margarine	3000_Vegetables	93	95	-2	2	4	A
75213120	Cactus, cooked, fat added	3000_Vegetables	93	95	-2	2	4	A
75202000	Asparagus, cooked, NS as to form, NS as to fat added in cooking	3000_Vegetables	93	94	-1	2	4	A
75209022	Brussels sprouts, frozen, cooked, fat added	3000_Vegetables	93	94	-1	2	4.5	A
72201222	Broccoli, frozen, cooked, fat added, NS as to fat type	3000_Vegetables	93	93	0	2	4.5	A
75311110	Classic mixed vegetables, canned, reduced sodium, cooked, no added fat	3000_Vegetables	93	92	1	2	4.5	A
75316000	Squash, summer, yellow or green, and onions, cooked, fat not added in cooking	3000_Vegetables	93	92	1	1	3.5	A
75117020	Onions, raw	3000_Vegetables	93	91	2	1	5	A
89902040	Onions, for use on a sandwich	3000_Vegetables	93	91	2	1	4	A
75202036	Asparagus, cooked, from canned, made with margarine	3000_Vegetables	92	97	-5	3	3.5	A
75233024	Squash, summer, yellow or green, cooked, NS as to form, made with oil	3000_Vegetables	92	97	-5	2	4	A
73201027	Pumpkin, cooked, from fresh, made with oil	3000_Vegetables	92	96	-4	2	3.5	A
75208310	Bitter melon, cooked	3000_Vegetables	92	94	-2	2	4.5	A
75340021	Vegetable combinations, Asian style, broccoli, green pepper, water chestnut, etc., cooked, made with oil	3000_Vegetables	92	94	-2	1	4	A
75206013	Beans, string, yellow, cooked, from canned, fat not added in cooking	3000_Vegetables	92	93	-1	3	4	A
75209032	Brussels sprouts, cooked, NS as to form, made with margarine	3000_Vegetables	92	93	-1	2	4.5	A
75231001	Snowpea, cooked, from fresh, NS as to fat added in cooking	3000_Vegetables	92	93	-1	1	4	A
75330090	Broccoli, cauliflower and carrots, cooked, fat added	3000_Vegetables	92	93	-1	2	4.5	B
75224043	Green peas, fresh, cooked with oil	3000_Vegetables	92	90	2	1	5	A
73301010	Squash, winter type, mashed, no fat or sugar added in cooking	3000_Vegetables	92	89	3	1	4	B
73102210	Carrots, cooked, NS as to form, fat not added in cooking	3000_Vegetables	92	87	5	1	4	A
75231010	Snowpea, cooked, NS as to form, fat not added in cooking	3000_Vegetables	92	87	5	1	4.5	A
75201010	Artichoke, cooked, NS as to form, fat not added in cooking	3000_Vegetables	92	84	8	1	4	A
75220051	Lettuce, cooked	3000_Vegetables	91	98	-7	2	4.5	A
75233027	Summer squash, yellow or green, fresh, cooked with oil	3000_Vegetables	91	97	-6	2	4	A
75217301	Flowers or blossoms of sesbania, squash, or lily, cooked	3000_Vegetables	91	96	-5	2	4.5	A
75227099	Radish, daikon, cooked, NS as to fat added in cooking	3000_Vegetables	91	96	-5	2	4	B
75215030	Celery, cooked, made with oil	3000_Vegetables	91	95	-4	2	4	A
75219021	Mushrooms, fresh, cooked, fat added, NS as to fat type	3000_Vegetables	91	94	-3	2	4.5	A
72107202	Collards, cooked, from frozen, NS as to fat added in cooking	3000_Vegetables	91	93	-2	2	4	A
72123000	Poke greens, cooked, NS as to fat added in cooking	3000_Vegetables	91	93	-2	2	4	A
75202032	Asparagus, frozen, cooked with butter or margarine	3000_Vegetables	91	93	-2	2	4	A
75207010	Bean sprouts, cooked, NS as to form, fat not added in cooking	3000_Vegetables	91	93	-2	1	4	A
75207011	Bean sprouts, cooked, from fresh, fat not added in cooking	3000_Vegetables	91	93	-2	1	4	A
75212021	Cabbage, red, cooked, made with oil	3000_Vegetables	91	93	-2	2	4	A
75220022	Okra, frozen, cooked, fat added	3000_Vegetables	91	93	-2	2	4	A

75340130	Vegetable combination (green beans, broccoli, onions, mushrooms), cooked, NS as to fat added in cooking	3000_Vegetables	91	93	-2	2	4	A
75340150	Vegetable combination (green beans, broccoli, onions, mushrooms), cooked, fat added in cooking	3000_Vegetables	91	93	-2	2	4	A
72120240	Lambsquarter, cooked, made with butter	3000_Vegetables	91	92	-1	1	4	A
72122200	Mustard greens, cooked, NS as to form, NS as to fat added in cooking	3000_Vegetables	91	92	-1	2	4	A
72128400	Turnip greens with roots, cooked, NS as to form, NS as to fat added in cooking	3000_Vegetables	91	92	-1	2	4	A
72128402	Turnip greens with roots, cooked, from frozen, NS as to fat added in cooking	3000_Vegetables	91	92	-1	2	4	A
72128420	Turnip greens with roots, cooked, NS as to form, fat added in cooking	3000_Vegetables	91	92	-1	2	4	A
72128422	Turnip greens with roots, cooked, from frozen, fat added in cooking	3000_Vegetables	91	92	-1	2	4	A
73102224	Carrots, frozen, cooked with oil	3000_Vegetables	91	92	-1	2	4.5	A
75231026	Snowpea, cooked, from fresh, made with oil	3000_Vegetables	91	92	-1	1	4	A
75221061	Onions, green, cooked	3000_Vegetables	91	91	0	2	4	A
73201035	Pumpkin, cooked, from canned, made with margarine	3000_Vegetables	91	90	1	2	4	B
75209052	Brussels sprouts, cooked, from frozen, made with margarine	3000_Vegetables	91	90	1	2	4.5	A
75215120	Fennel bulb, cooked	3000_Vegetables	91	90	1	2	4.5	A
72128228	Turnip greens, cooked, from fresh, made with butter	3000_Vegetables	91	89	2	2	4	A
74504110	Tomato and onion, cooked, fat not added in cooking	3000_Vegetables	91	89	2	1	4	A
72107203	Collards, cooked, from canned, NS as to fat added in cooking	3000_Vegetables	91	88	3	2	4	A
75102500	Beets, raw	3000_Vegetables	91	86	5	1	5	A
75340110	Vegetable combinations (broccoli, carrots, corn, cauliflower, etc.), cooked, fat not added in cooking	3000_Vegetables	91	86	5	1	4.5	A
73102211	Carrots, fresh, cooked, no added fat	3000_Vegetables	91	85	6	1	4	A
72107234	Collards, canned, cooked with butter or margarine	3000_Vegetables	90	95	-5	2	3.5	A
75233029	Squash, summer, yellow or green, cooked, from fresh, made with margarine	3000_Vegetables	90	95	-5	2	4	A
72122201	Mustard greens, cooked, from fresh, NS as to fat added in cooking	3000_Vegetables	90	92	-2	2	4	A
72122203	Mustard greens, cooked, from canned, NS as to fat added in cooking	3000_Vegetables	90	92	-2	2	4	A
72201221	Broccoli, fresh, cooked, fat added, NS as to fat type	3000_Vegetables	90	92	-2	2	4.5	A
73201026	Pumpkin, cooked, NS as to form, made with margarine	3000_Vegetables	90	92	-2	2	3.5	B
73201029	Pumpkin, cooked, from fresh, made with margarine	3000_Vegetables	90	92	-2	2	3.5	B
73201032	Pumpkin, cooked, from frozen, made with margarine	3000_Vegetables	90	92	-2	2	3.5	A
73303021	Squash, winter type, baked, no sugar added in cooking, made with oil	3000_Vegetables	90	92	-2	1	3.5	B
75205047	Green beans, frozen, cooked with oil	3000_Vegetables	90	92	-2	2	4.5	A
75214021	Cauliflower, fresh, cooked, fat added, NS as to fat type	3000_Vegetables	90	92	-2	2	4.5	A
75214022	Cauliflower, frozen, cooked, fat added, NS as to fat type	3000_Vegetables	90	92	-2	2	4.5	A
72103050	Broccoli raab, cooked, made with butter	3000_Vegetables	90	91	-1	1	4	A
72128401	Turnip greens with roots, cooked, from fresh, NS as to fat added in cooking	3000_Vegetables	90	91	-1	2	4	A

72128421	Turnip greens with roots, cooked, from fresh, fat added in cooking	3000_Vegetables	90	91	-1	2	4	A
75209020	Brussels sprouts, NS as to form, cooked	3000_Vegetables	90	90	0	2	4.5	A
72118225	Greens, cooked, NS as to form, made with butter	3000_Vegetables	90	89	1	1	4	A
73102213	Carrots, canned, cooked, no added fat	3000_Vegetables	90	89	1	3	4	A
75220001	Okra, cooked, from fresh, NS as to fat added in cooking	3000_Vegetables	90	89	1	2	4	C
75102000	Beans, lima, raw	3000_Vegetables	90	88	2	1	5	A
75201040	Artichoke, cooked, from frozen, made with oil	3000_Vegetables	90	88	2	1	4	A
27420080	Greens with ham or pork	3000_Vegetables	90	87	3	2	4	A
72122231	Mustard greens, cooked, from frozen, made with butter	3000_Vegetables	90	87	3	2	4	B
75234011	Turnip, cooked, from fresh, fat not added in cooking	3000_Vegetables	90	83	7	1	4	A
75111800	Jicama, raw	3000_Vegetables	90	82	8	1	5	A
75215510	Christophine, cooked, fat not added in cooking	3000_Vegetables	90	82	8	1	4	A
72126999	Thistle leaves, cooked, NS as to fat added in cooking	3000_Vegetables	89	94	-5	2	4	A
75205130	Green beans, canned, reduced sodium, cooked, fat added, NS as to fat type	3000_Vegetables	89	93	-4	2	4.5	A
75233030	Summer squash, yellow or green, frozen, cooked with oil	3000_Vegetables	89	93	-4	2	4	A
74201003	Tomatoes, canned, cooked	3000_Vegetables	89	92	-3	3	4	A
75207021	Bean sprouts, cooked	3000_Vegetables	89	91	-2	1	5	A
75210022	Cabbage, Chinese, cooked, made with butter	3000_Vegetables	89	91	-2	2	3.5	A
75226060	Peppers, red, cooked	3000_Vegetables	89	91	-2	2	4.5	A
73102217	Carrots, fresh, cooked with oil	3000_Vegetables	89	90	-1	1	4	A
75215121	Fennel bulb, cooked, made with oil	3000_Vegetables	89	90	-1	2	4.5	A
75217020	Eggplant, cooked, fat added	3000_Vegetables	89	89	0	2	4.5	A
75231000	Snowpea, cooked, NS as to form, NS as to fat added in cooking	3000_Vegetables	89	89	0	1	4.5	A
72118234	Greens, cooked, from canned, made with butter	3000_Vegetables	89	88	1	1	4	A
75218011	Kohlrabi, cooked	3000_Vegetables	89	87	2	2	4.5	A
75302060	Beans, string, green, with pinto beans, cooked, fat not added in cooking	3000_Vegetables	89	87	2	3	5	A
72107225	Collards, cooked, NS as to form, made with butter	3000_Vegetables	89	86	3	1	4.5	A
75201034	Artichoke, cooked, from fresh, made with oil	3000_Vegetables	89	86	3	1	4.5	A
75226010	Peppers, green, cooked, fat not added in cooking	3000_Vegetables	89	86	3	1	4	A
75226050	Peppers, red, cooked, fat not added in cooking	3000_Vegetables	89	86	3	1	4	A
75306998	Peppers and onions, cooked, no added fat	3000_Vegetables	89	85	4	1	4.5	A
73102227	Carrots, canned, cooked with oil	3000_Vegetables	88	94	-6	3	4	A
75226061	Peppers, red, cooked, made with oil	3000_Vegetables	88	92	-4	2	4	A
75205031	Green beans, fresh, cooked, fat added, NS as to fat type	3000_Vegetables	88	90	-2	2	4.5	A
72113200	Dandelion greens, cooked, NS as to fat added in cooking	3000_Vegetables	88	89	-1	2	4	B
73102214	Carrots, cooked, NS as to form, made with oil	3000_Vegetables	88	89	-1	2	4	A
75224131	Green peas, canned, reduced sodium, cooked with oil	3000_Vegetables	88	89	-1	1	5	A
75220023	Okra, cooked, from canned, fat added in cooking, NS as to type of fat	3000_Vegetables	88	88	0	2	4	C

75231022	Snowpea, frozen, cooked, fat added	3000_Vegetables	88	88	0	1	4.5	A
75231029	Snowpea, cooked, from frozen, made with oil	3000_Vegetables	88	88	0	1	4.5	A
75340020	Asian stir fry vegetables, cooked, fat added	3000_Vegetables	88	88	0	2	5	A
75340100	Vegetable combinations (broccoli, carrots, corn, cauliflower, etc.), cooked, NS as to fat added in cooking	3000_Vegetables	88	88	0	2	4	A
75340120	Vegetable combinations (broccoli, carrots, corn, cauliflower, etc.), cooked, fat added in cooking	3000_Vegetables	88	88	0	2	4	A
75218010	Kohlrabi, cooked, fat not added in cooking	3000_Vegetables	88	86	2	1	4	B
74505010	Tomato with corn and okra, cooked, fat not added in cooking	3000_Vegetables	88	85	3	3	4	B
75228010	Rutabaga, cooked, fat not added in cooking	3000_Vegetables	88	85	3	1	4	B
72128203	Turnip greens, cooked, from canned, NS as to fat added in cooking	3000_Vegetables	87	92	-5	2	4	A
72119200	Kale, cooked, NS as to form, NS as to fat added in cooking	3000_Vegetables	87	91	-4	2	4	C
72119201	Kale, cooked, from fresh, NS as to fat added in cooking	3000_Vegetables	87	91	-4	2	4	C
75226021	Peppers, green, cooked, made with oil	3000_Vegetables	87	91	-4	2	4	A
72119202	Kale, cooked, from frozen, NS as to fat added in cooking	3000_Vegetables	87	90	-3	2	4	B
75203028	Bamboo shoots, cooked	3000_Vegetables	87	90	-3	2	4.5	A
75211033	Cabbage, green, cooked, made with margarine	3000_Vegetables	87	89	-2	2	4	A
75212020	Cabbage, red, cooked	3000_Vegetables	87	89	-2	2	4	A
75215020	Celery, cooked	3000_Vegetables	87	89	-2	2	4	A
75219034	Mushrooms, fresh, cooked with butter or margarine	3000_Vegetables	87	89	-2	2	4	B
75226020	Peppers, green, cooked	3000_Vegetables	87	89	-2	2	4	A
73103000	Carrots, canned, low sodium, NS as to fat added in cooking	3000_Vegetables	87	87	0	2	4	A
73111224	Peas and carrots, cooked, NS as to form, made with oil	3000_Vegetables	87	87	0	1	4.5	A
73111227	Peas and carrots, cooked, from fresh, made with oil	3000_Vegetables	87	87	0	1	4.5	A
73111230	Peas and carrots, cooked, from frozen, made with oil	3000_Vegetables	87	87	0	1	4.5	A
75302045	Beans, string, green, with almonds, cooked, fat added in cooking, NS as to type of fat	3000_Vegetables	87	87	0	1	4.5	A
72118228	Greens, cooked, from fresh, made with butter	3000_Vegetables	87	86	1	1	4	A
74504000	Tomato and okra, cooked, NS as to fat added in cooking	3000_Vegetables	87	86	1	2	4	B
74504020	Tomato and okra, cooked, fat added in cooking, NS as to type of fat	3000_Vegetables	87	86	1	2	4	B
75230020	Sauerkraut, cooked, fat added in cooking	3000_Vegetables	87	86	1	3	3.5	C
73111220	Peas and carrots, cooked, NS as to form	3000_Vegetables	87	85	2	1	5	A
73111221	Peas and carrots, fresh, cooked, fat added	3000_Vegetables	87	85	2	1	5	A
73111212	Peas and carrots, frozen, cooked, no added fat	3000_Vegetables	87	81	6	1	4.5	A
75205052	Beans, string, green, cooked, from canned, made with margarine	3000_Vegetables	86	90	-4	2	4	A
72119203	Kale, cooked, from canned, NS as to fat added in cooking	3000_Vegetables	86	89	-3	2	4	C
75340300	Pinacbet	3000_Vegetables	86	89	-3	2	3.5	A
75231021	Snowpea, fresh, cooked, fat added	3000_Vegetables	86	86	0	2	4	A
72123040	Poke greens, cooked, made with butter	3000_Vegetables	86	85	1	2	4	B
73102216	Carrots, cooked, NS as to form, made with margarine	3000_Vegetables	86	85	1	2	4	A
73102226	Carrots, cooked, from frozen, made with margarine	3000_Vegetables	86	85	1	2	4	B

75511040	Pepper, hot, pickled	3000_Vegetables	85	90	-5	4	2.5	D
75214029	Cauliflower, cooked, from fresh, made with margarine	3000_Vegetables	85	88	-3	2	4.5	B
72128234	Turnip greens, cooked, from canned, made with butter	3000_Vegetables	85	87	-2	2	4	B
74201001	Tomatoes, fresh, cooked	3000_Vegetables	85	87	-2	2	4	A
74504121	Tomato and onion, cooked, made with oil	3000_Vegetables	85	87	-2	2	4	A
75204960	Beans, string, cooked, NS as to form, NS as to color, made with oil	3000_Vegetables	85	86	-1	2	4.5	A
75204963	Beans, string, cooked, from fresh, NS as to color, made with oil	3000_Vegetables	85	86	-1	2	4.5	A
75204966	Beans, string, cooked, from frozen, NS as to color, made with oil	3000_Vegetables	85	86	-1	2	4	A
75205041	Beans, string, green, cooked, NS as to form, made with oil	3000_Vegetables	85	86	-1	2	4.5	A
75206030	Beans, string, yellow, cooked, NS as to form, made with oil	3000_Vegetables	85	86	-1	2	4.5	A
72118231	Greens, cooked, from frozen, made with butter	3000_Vegetables	85	85	0	1	4	B
72201220	Broccoli, NS as to form, cooked	3000_Vegetables	85	85	0	2	4.5	A
73103022	Carrots, canned, reduced sodium, cooked with butter or margarine	3000_Vegetables	85	85	0	2	4	A
73303023	Squash, winter type, baked, no sugar added in cooking, made with margarine	3000_Vegetables	85	85	0	2	3.5	A
72122234	Mustard greens, cooked, from canned, made with butter	3000_Vegetables	85	84	1	1	4	A
75231025	Snowpea, cooked, NS as to form, made with margarine	3000_Vegetables	85	84	1	2	4.5	A
75202025	Asparagus, cooked, NS as to form, made with butter	3000_Vegetables	85	83	2	2	4	A
75231031	Snowpea, cooked, from frozen, made with margarine	3000_Vegetables	85	83	2	2	4.5	A
72201200	Broccoli, cooked, NS as to form, NS as to fat added in cooking	3000_Vegetables	85	82	3	2	4.5	A
75216710	Cucumber, cooked, fat not added in cooking	3000_Vegetables	85	82	3	1	3.5	A
75224031	Green peas, fresh, cooked, fat added, NS as to fat type	3000_Vegetables	85	82	3	1	5	A
75204110	Beans, lima, immature, canned, low sodium, fat not added in cooking	3000_Vegetables	85	80	5	1	5	A
73111210	Peas and carrots, cooked, NS as to form, fat not added in cooking	3000_Vegetables	85	79	6	1	4.5	A
72110200	Cress, cooked, NS as to form, NS as to fat added in cooking	3000_Vegetables	84	89	-5	2	4	A
72110201	Cress, cooked, from fresh, NS as to fat added in cooking	3000_Vegetables	84	89	-5	2	4	A
72110203	Cress, cooked, from canned, NS as to fat added in cooking	3000_Vegetables	84	89	-5	2	4	A
72110220	Cress, cooked, NS as to form, fat added in cooking, NS as to type of fat	3000_Vegetables	84	89	-5	2	4	A
72110223	Cress, cooked, from canned, fat added in cooking, NS as to type of fat	3000_Vegetables	84	89	-5	2	4	A
75233021	Summer squash, yellow or green, fresh, cooked, fat added, NS as to fat type	3000_Vegetables	84	89	-5	2	4	A
73210010	Calabaza, cooked	3000_Vegetables	84	88	-4	2	4	A
72110237	Cress, cooked, from canned, made with butter	3000_Vegetables	84	87	-3	2	4	A
75202035	Asparagus, canned, cooked with butter or margarine	3000_Vegetables	84	86	-2	3	3.5	A
75219020	Mushrooms, NS as to form, cooked	3000_Vegetables	84	86	-2	2	4.5	A
75316031	Squash, summer, yellow or green, with tomato sauce, cooked, fat added in cooking	3000_Vegetables	84	86	-2	2	4	A
73303020	Winter squash, cooked, fat added	3000_Vegetables	84	85	-1	2	4	A
73102222	Carrots, frozen, cooked, fat added, NS as to fat type	3000_Vegetables	84	84	0	2	4.5	A

75204965	Beans, string, cooked, from fresh, NS as to color, made with margarine	3000_Vegetables	84	84	0	2	4.5	A
75205046	Beans, string, green, cooked, from fresh, made with margarine	3000_Vegetables	84	84	0	2	4.5	A
72113240	Dandelion greens, cooked, made with butter	3000_Vegetables	84	83	1	1	4	B
73102219	Carrots, cooked, from fresh, made with margarine	3000_Vegetables	84	83	1	2	4	A
75204962	Beans, string, cooked, NS as to form, NS as to color, made with margarine	3000_Vegetables	84	83	1	2	4.5	A
72201202	Broccoli, cooked, from frozen, NS as to fat added in cooking	3000_Vegetables	84	82	2	2	4.5	A
72201227	Broccoli, frozen, cooked with butter or margarine	3000_Vegetables	84	82	2	2	4.5	A
75209000	Brussels sprouts, cooked, NS as to form, NS as to fat added in cooking	3000_Vegetables	84	82	2	2	4.5	A
75201020	Artichokes, NS as to form, cooked	3000_Vegetables	84	80	4	1	4.5	A
75201021	Artichoke, fresh, cooked, fat added	3000_Vegetables	84	80	4	1	4.5	A
75201023	Artichoke, canned, cooked, fat added	3000_Vegetables	84	80	4	1	4.5	A
75503010	Pickles, dill	3000_Vegetables	83	91	-8	4	3.5	C
72110231	Cress, cooked, NS as to form, made with butter	3000_Vegetables	83	86	-3	2	4	A
72110234	Cress, cooked, from fresh, made with butter	3000_Vegetables	83	86	-3	2	4	A
72132199	Bitter melon leaves, horseradish leaves, jute leaves, or radish leaves, cooked, NS as to fat added in cooking	3000_Vegetables	83	86	-3	2	4	C
75209001	Brussels sprouts, cooked, from fresh, NS as to fat added in cooking	3000_Vegetables	83	86	-3	2	4	A
75316022	Squash, summer, yellow or green, and onions, cooked, made with oil	3000_Vegetables	83	86	-3	2	3.5	A
72128403	Turnip greens with roots, cooked, from canned, NS as to fat added in cooking	3000_Vegetables	83	85	-2	2	3.5	A
72128423	Turnip greens with roots, cooked, from canned, fat added in cooking	3000_Vegetables	83	85	-2	2	3.5	A
72201190	Broccoli, cooked, from restaurant	3000_Vegetables	83	85	-2	2	4	A
75215511	Christophine, cooked	3000_Vegetables	83	85	-2	2	4	A
75340200	Jai, Monk's Food	3000_Vegetables	83	85	-2	2	4	A
73403021	Sweet potato, baked, peel not eaten, made with oil	3000_Vegetables	83	84	-1	1	4	B
75214020	Cauliflower, NS as to form, cooked	3000_Vegetables	83	84	-1	2	4.5	A
72201201	Broccoli, cooked, from fresh, NS as to fat added in cooking	3000_Vegetables	83	83	0	2	4	A
75205032	Green beans, frozen, cooked, fat added, NS as to fat type	3000_Vegetables	83	83	0	2	4.5	A
75205043	Beans, string, green, cooked, NS as to form, made with margarine	3000_Vegetables	83	83	0	2	4.5	A
75211030	Cabbage, green, cooked, fat added, NS as to fat type	3000_Vegetables	83	83	0	2	4	A
73111226	Peas and carrots, cooked, NS as to form, made with margarine	3000_Vegetables	83	82	1	2	4.5	A
73111229	Peas and carrots, cooked, from fresh, made with margarine	3000_Vegetables	83	82	1	2	4.5	A
73111232	Peas and carrots, cooked, from frozen, made with margarine	3000_Vegetables	83	82	1	2	4.5	A
75205049	Beans, string, green, cooked, from frozen, made with margarine	3000_Vegetables	83	82	1	2	4	A
75214028	Cauliflower, fresh, cooked with butter or margarine	3000_Vegetables	83	82	1	2	4.5	A
75224133	Peas, green, canned, low sodium, made with margarine	3000_Vegetables	83	82	1	3	5	A
75302047	Beans, string, green, with almonds, cooked, made with butter	3000_Vegetables	83	82	1	1	4	A
75209002	Brussels sprouts, cooked, from frozen, NS as to fat added in cooking	3000_Vegetables	83	81	2	2	4.5	A
75219000	Mushrooms, cooked, NS as to form, NS as to fat added in cooking	3000_Vegetables	83	81	2	2	4	B

75219001	Mushrooms, cooked, from fresh, NS as to fat added in cooking	3000_Vegetables	83	81	2	2	4	B
75219002	Mushrooms, cooked, from frozen, NS as to fat added in cooking	3000_Vegetables	83	81	2	2	4	B
75219022	Mushrooms, cooked, from frozen, fat added in cooking, NS as to type of fat	3000_Vegetables	83	81	2	2	4	B
75220000	Okra, cooked, NS as to form, NS as to fat added in cooking	3000_Vegetables	83	81	2	2	4	B
75311027	Classic mixed vegetables, frozen, cooked with oil	3000_Vegetables	83	81	2	1	4.5	A
75315216	Peas and mushrooms, cooked, made with oil	3000_Vegetables	83	81	2	1	4.5	B
75143350	Lettuce, salad with egg, cheese, tomato, and/or carrots, with or without other vegetables, no dressing	3000_Vegetables	83	80	3	2	4	A
75220028	Okra, cooked, from fresh, made with butter	3000_Vegetables	83	79	4	1	4	C
72203030	Broccoli, chinese, cooked, from frozen, NS as to fat added in cooking	3000_Vegetables	82	87	-5	2	4	B
72203110	Broccoli, chinese, cooked, from fresh, fat added in cooking, NS as to type of fat	3000_Vegetables	82	87	-5	2	4	B
73201020	Pumpkin, cooked	3000_Vegetables	82	86	-4	2	3.5	A
75207000	Bean sprouts, cooked, NS as to form, NS as to fat added in cooking	3000_Vegetables	82	86	-4	2	4	A
75207001	Bean sprouts, cooked, from fresh, NS as to fat added in cooking	3000_Vegetables	82	86	-4	2	4	A
75207020	Bean sprouts, cooked, NS as to form, fat added in cooking	3000_Vegetables	82	86	-4	2	4	A
75204971	Beans, string, cooked, from canned, NS as to color, made with margarine	3000_Vegetables	82	85	-3	2	4	A
75204969	Beans, string, cooked, from canned, NS as to color, made with oil	3000_Vegetables	82	84	-2	2	4	A
75205050	Green beans, canned, cooked with oil	3000_Vegetables	82	84	-2	2	4	A
75219003	Mushrooms, cooked, from canned, NS as to fat added in cooking	3000_Vegetables	82	84	-2	3	3.5	C
73402021	Sweet potato, baked, peel eaten, made with oil	3000_Vegetables	82	83	-1	1	4	B
75234027	Turnip, cooked, from fresh, made with oil	3000_Vegetables	82	83	-1	2	4	A
72201224	Broccoli, fresh, cooked with butter or margarine	3000_Vegetables	82	82	0	2	4	A
75206020	Yellow string beans, cooked	3000_Vegetables	82	82	0	2	4.5	A
75302009	Beans, string, green, with tomatoes, cooked, NS as to fat added in cooking	3000_Vegetables	82	82	0	2	4	A
75302011	Beans, string, green, with tomatoes, cooked, fat added in cooking	3000_Vegetables	82	82	0	2	4	A
75214000	Cauliflower, cooked, NS as to form, NS as to fat added in cooking	3000_Vegetables	82	81	1	2	4.5	B
75220002	Okra, cooked, from frozen, NS as to fat added in cooking	3000_Vegetables	82	81	1	2	4	B
75231020	Snowpea, NS as to form, cooked	3000_Vegetables	82	81	1	2	4	A
73102221	Carrots, fresh, cooked, fat added, NS as to fat type	3000_Vegetables	82	80	2	2	4	A
73201003	Pumpkin, cooked, from canned, NS as to fat added in cooking	3000_Vegetables	82	78	4	1	4	B
73201023	Pumpkin, cooked, from canned, fat added in cooking, NS as to type of fat	3000_Vegetables	82	78	4	1	4	B
73402010	Sweet potato, baked, peel eaten, no added fat	3000_Vegetables	82	78	4	1	4	B
75302020	Beans, string, green, with onions, cooked, fat not added in cooking	3000_Vegetables	82	77	5	1	4	A
75315210	Peas and mushrooms, cooked, fat not added in cooking	3000_Vegetables	82	75	7	1	4.5	B
72203150	Broccoli, chinese, cooked, from frozen, fat added in cooking, NS as to type of fat	3000_Vegetables	81	85	-4	2	4	B
75205110	Beans, string, green, canned, low sodium, NS as to fat added in cooking	3000_Vegetables	81	84	-3	2	4	A

75226023	Peppers, green, cooked, made with margarine	3000_Vegetables	81	84	-3	2	4	B
75227110	Daikon radish, cooked	3000_Vegetables	81	83	-2	2	4	A
74504123	Tomato and onion, cooked, made with margarine	3000_Vegetables	81	82	-1	2	3.5	A
74505021	Tomato with corn and okra, cooked, made with oil	3000_Vegetables	81	82	-1	2	4	B
74504148	Tomato and celery, cooked, NS as to fat added in cooking	3000_Vegetables	81	81	0	3	3.5	B
75214031	Cauliflower, frozen, cooked with butter or margarine	3000_Vegetables	81	81	0	2	4.5	A
75307000	Peppers and onions, cooked, fat added	3000_Vegetables	81	81	0	2	4.5	A
75205045	Green beans, fresh, cooked with butter or margarine	3000_Vegetables	81	80	1	2	4.5	A
75214002	Cauliflower, cooked, from frozen, NS as to fat added in cooking	3000_Vegetables	81	80	1	2	4.5	B
75330120	Vegetable combination, including carrots, broccoli, and/or dark-green leafy; cooked, no sauce, fat added in cooking, NS as to type of fat	3000_Vegetables	81	80	1	2	4	B
73111222	Peas and carrots, frozen, cooked, fat added	3000_Vegetables	81	78	3	1	4.5	A
75200121	Vegetables, NS as to type, cooked, made with oil	3000_Vegetables	81	78	3	1	4.5	A
75211032	Cabbage, green, cooked with butter or margarine	3000_Vegetables	81	78	3	2	4	A
75224046	Green peas, frozen, cooked with oil	3000_Vegetables	81	78	3	1	5	A
72201214	Broccoli, cooked, NS as to form, made with butter	3000_Vegetables	81	77	4	1	4	A
73403010	Sweet potato, baked, peel not eaten, no added fat	3000_Vegetables	81	77	4	1	4	B
75311024	Mixed vegetables, cooked, NS as to form, made with oil	3000_Vegetables	81	77	4	1	4.5	A
75311012	Classic mixed vegetables, frozen, cooked, no added fat	3000_Vegetables	81	74	7	1	4.5	A
75503110	Cucumber pickles, dill, reduced salt	3000_Vegetables	80	87	-7	4	4	A
72203010	Broccoli, chinese, cooked, NS as to form, NS as to fat added in cooking	3000_Vegetables	80	84	-4	2	4	B
72203020	Broccoli, chinese, cooked, from fresh, NS as to fat added in cooking	3000_Vegetables	80	84	-4	2	4	B
73102223	Carrots, canned, cooked, fat added, NS as to fat type	3000_Vegetables	80	83	-3	3	4	A
75205132	Green beans, canned, reduced sodium, cooked with butter or margarine	3000_Vegetables	80	83	-3	2	4	A
75228021	Rutabaga, cooked, made with oil	3000_Vegetables	80	82	-2	2	4	B
72119228	Kale, cooked, from fresh, made with butter	3000_Vegetables	80	81	-1	1	4	A
75311120	Classic mixed vegetables, canned, reduced sodium, cooked, fat added, NS as to fat type	3000_Vegetables	80	81	-1	2	4.5	A
75230000	Sauerkraut	3000_Vegetables	80	80	0	3	3.5	C
75302211	Beans, string, green, with onions, cooked, made with oil	3000_Vegetables	80	80	0	2	4	A
75330100	Vegetable combination, including carrots, broccoli, and/or dark-green leafy; cooked, no sauce, NS as to fat added in cooking	3000_Vegetables	80	80	0	2	4	A
72122225	Mustard greens, cooked, NS as to form, made with butter	3000_Vegetables	80	79	1	1	4	A
72122228	Mustard greens, cooked, from fresh, made with butter	3000_Vegetables	80	79	1	1	4	B
73402023	Sweet potato, baked, peel eaten, made with margarine	3000_Vegetables	80	79	1	1	4	B
75311100	Mixed vegetables, canned, low sodium, NS as to fat added in cooking	3000_Vegetables	80	79	1	3	4.5	A
75213100	Cactus, cooked, NS as to fat added in cooking	3000_Vegetables	80	78	2	2	4	A
75224130	Green peas, canned, reduced sodium, cooked, fat added, NS as to fat type	3000_Vegetables	80	78	2	1	5	A

71001000	White potato, raw, with or without peel (assume peel not eaten)	3000_Vegetables	80	77	3	1	5	A
75204120	Beans, lima, immature, canned, low sodium, fat added in cooking	3000_Vegetables	80	77	3	1	5	A
72116222	Escarole, cooked, made with butter	3000_Vegetables	80	76	4	2	4	A
75219100	Mushroom, Asian, cooked, from dried	3000_Vegetables	80	75	5	1	4.5	B
75208011	Beets, fresh, cooked, no added fat	3000_Vegetables	80	72	8	1	4	A
72116150	Caesar salad, with romaine, no dressing	3000_Vegetables	79	82	-3	3	4.5	A
75205005	Green beans, cooked, from restaurant	3000_Vegetables	79	81	-2	2	4.5	A
75233022	Summer squash, yellow or green, frozen, cooked, fat added, NS as to fat type	3000_Vegetables	79	81	-2	2	4	A
72119231	Kale, cooked, from frozen, made with butter	3000_Vegetables	79	80	-1	1	4	A
75316024	Squash, summer, yellow or green, and onions, cooked, made with margarine	3000_Vegetables	79	80	-1	2	3.5	A
75316032	Squash, summer, yellow or green, with tomato sauce, cooked, NS as to fat added in cooking	3000_Vegetables	79	80	-1	2	4	A
73403023	Sweet potato, baked, peel not eaten, made with margarine	3000_Vegetables	79	78	1	2	4	B
75221040	Onions, green, cooked, NS as to form, NS as to fat added in cooking	3000_Vegetables	79	77	2	2	3.5	B
75221060	Onions, green, cooked, NS as to form, fat added in cooking	3000_Vegetables	79	77	2	2	3.5	B
75223024	Peas, cowpeas, field peas, or blackeye peas, not dried, cooked, NS as to form, made with oil	3000_Vegetables	79	76	3	1	5	A
75223030	Peas, cowpeas, field peas, or blackeye peas, not dried, cooked, from frozen, made with oil	3000_Vegetables	79	76	3	1	5	A
75224000	Green peas, cooked, from restaurant	3000_Vegetables	79	76	3	1	5	A
75223012	Peas, cowpeas, field peas, or blackeye peas, not dried, cooked, from frozen, fat not added in cooking	3000_Vegetables	79	74	5	1	5	A
75224044	Green peas, fresh, cooked with butter or margarine	3000_Vegetables	79	74	5	1	5	A
75223010	Peas, cowpeas, field peas, or blackeye peas, not dried, cooked, NS as to form, fat not added in cooking	3000_Vegetables	79	73	6	1	5	A
75221012	Onions, cooked, from frozen, fat not added in cooking	3000_Vegetables	79	72	7	1	3.5	A
75224022	Green peas, frozen, cooked, no added fat	3000_Vegetables	79	72	7	1	5	A
72133199	Sweet potato leaves, squash leaves, pumpkin leaves, chrysanthemum leaves, bean leaves, or swamp cabbage, cooked, NS as to fat added in cooking	3000_Vegetables	78	80	-2	2	3.5	C
73111233	Peas and carrots, cooked, from canned, made with oil	3000_Vegetables	78	79	-1	3	4	A
72119225	Kale, cooked, NS as to form, made with butter	3000_Vegetables	78	78	0	1	4	A
73201000	Pumpkin, cooked, NS as to form, NS as to fat added in cooking	3000_Vegetables	78	78	0	2	3.5	B
74504149	Tomato and celery, cooked, fat added in cooking	3000_Vegetables	78	78	0	2	3.5	A
75317011	Vegetables, stew type, cooked, made with oil	3000_Vegetables	78	78	0	1	4	A
73201001	Pumpkin, cooked, from fresh, NS as to fat added in cooking	3000_Vegetables	78	77	1	2	3.5	B
73201002	Pumpkin, cooked, from frozen, NS as to fat added in cooking	3000_Vegetables	78	77	1	2	3.5	B
73201021	Pumpkin, cooked, from fresh, fat added in cooking, NS as to type of fat	3000_Vegetables	78	77	1	2	3.5	B
73201022	Pumpkin, cooked, from frozen, fat added in cooking, NS as to type of fat	3000_Vegetables	78	77	1	2	3.5	B
75212000	Cabbage, red, cooked, NS as to fat added in cooking	3000_Vegetables	78	76	2	2	4	A

75340000	Vegetable combinations, Asian style, broccoli, green pepper, water chestnut, etc., cooked, NS as to fat added in cooking	3000_Vegetables	78	76	2	2	4	A
73111213	Peas and carrots, canned, cooked, no added fat	3000_Vegetables	78	75	3	3	4.5	A
75224040	Peas, green, cooked, NS as to form, made with oil	3000_Vegetables	78	75	3	1	4.5	B
75204027	Beans, lima, immature, cooked, from fresh, made with oil	3000_Vegetables	78	74	4	1	5	A
71103210	White potato, boiled, without peel, canned, low sodium, fat not added in cooking	3000_Vegetables	78	73	5	1	4.5	A
74502010	Tomato and lima beans, cooked, fat not added cooking	3000_Vegetables	78	73	5	1	4	A
75311026	Mixed vegetables, cooked, NS as to form, made with margarine	3000_Vegetables	78	73	5	2	4.5	A
75311029	Mixed vegetables, cooked, from frozen, made with margarine	3000_Vegetables	78	73	5	2	4.5	A
75200110	Vegetables, NS as to type, cooked, fat not added in cooking	3000_Vegetables	78	70	8	1	4.5	A
75311010	Mixed vegetables, cooked, NS as to form, fat not added in cooking	3000_Vegetables	78	70	8	1	4.5	A
75217021	Eggplant, cooked, made with oil	3000_Vegetables	77	79	-2	2	4	A
73410340	Sweet potato fries, from fresh, baked	3000_Vegetables	77	78	-1	2	3.5	C
74201000	Tomatoes, NS as to form, cooked	3000_Vegetables	77	78	-1	2	4	A
72119234	Kale, cooked, from canned, made with butter	3000_Vegetables	77	77	0	1	4	A
73102190	Carrots, cooked, from restaurant	3000_Vegetables	77	77	0	2	4	A
73405021	Sweet potato, boiled, made with oil	3000_Vegetables	77	77	0	1	3.5	B
74505023	Tomato with corn and okra, cooked, made with margarine	3000_Vegetables	77	77	0	3	4	B
75215000	Celery, cooked, NS as to fat added in cooking	3000_Vegetables	77	77	0	2	4	A
75228020	Rutabaga, cooked	3000_Vegetables	77	77	0	2	4	A
75221160	Palm hearts, cooked	3000_Vegetables	77	76	1	1	4	C
73102220	Carrots, NS as to form, cooked	3000_Vegetables	77	75	2	2	4	A
73301000	Squash, winter type, mashed, NS as to fat or sugar added in cooking	3000_Vegetables	77	75	2	1	3.5	A
73301020	Squash, winter type, mashed, fat added in cooking, no sugar added in cooking	3000_Vegetables	77	75	2	1	3.5	A
73303000	Squash, winter type, baked, NS as to fat or sugar added in cooking	3000_Vegetables	77	75	2	1	3.5	B
75221014	Onions, cooked, NS as to form, made with oil	3000_Vegetables	77	75	2	1	3.5	A
73102225	Carrots, frozen, cooked with butter or margarine	3000_Vegetables	77	74	3	2	4	A
75220032	Okra, cooked, from frozen, made with butter	3000_Vegetables	77	74	3	1	4	A
75223027	Peas, cowpeas, field peas, or blackeye peas, not dried, cooked, from fresh, made with oil	3000_Vegetables	77	74	3	1	4.5	A
74504022	Tomato and okra, cooked, made with butter	3000_Vegetables	77	73	4	2	3.5	B
75209031	Brussels sprouts, cooked, NS as to form, made with butter	3000_Vegetables	77	73	4	1	4.5	A
75209051	Brussels sprouts, cooked, from frozen, made with butter	3000_Vegetables	77	73	4	1	4.5	A
71980100	Poi	3000_Vegetables	77	72	5	1	4	A
75235000	Water Chesnut	3000_Vegetables	77	71	6	1	5	A
73102229	Carrots, cooked, from canned, made with margarine	3000_Vegetables	76	82	-6	3	4	B
75233020	Summer squash, yellow or green, NS as to form, cooked	3000_Vegetables	76	79	-3	2	4	A
75205030	Green beans, NS as to form, cooked	3000_Vegetables	76	77	-1	2	4.5	A
75209041	Brussels sprouts, cooked, from fresh, made with butter	3000_Vegetables	76	76	0	1	4	B

75219023	Mushrooms, canned, cooked	3000_Vegetables	76	76	0	3	4	C
75307001	Green peppers and onions, cooked, made with oil	3000_Vegetables	76	76	0	1	4	B
75208124	Beets, cooked, from fresh, made with oil	3000_Vegetables	76	75	1	1	3.5	A
75221023	Onions, cooked, from frozen, made with oil	3000_Vegetables	76	75	1	1	3.5	A
75440400	Pakora	3000_Vegetables	76	74	2	1	4	B
73407010	Sweet potato, canned without syrup	3000_Vegetables	76	73	3	1	4	A
75219031	Mushrooms, cooked, NS as to form, made with butter	3000_Vegetables	76	73	3	1	4	C
75223022	Blackeyed peas, from frozen	3000_Vegetables	76	73	3	1	5	A
73102218	Carrots, fresh, cooked with butter or margarine	3000_Vegetables	76	72	4	2	4	A
75205048	Green beans, frozen, cooked with butter or margarine	3000_Vegetables	76	72	4	2	4.5	A
75214025	Cauliflower, cooked, NS as to form, made with butter	3000_Vegetables	76	72	4	1	4	A
75215100	Fennel bulb, cooked, NS as to fat added in cooking	3000_Vegetables	76	72	4	2	4	A
75311020	Classic mixed vegetables, NS as to form, cooked	3000_Vegetables	76	72	4	1	4.5	A
75311022	Classic mixed vegetables, frozen, cooked, fat added, NS as to fat type	3000_Vegetables	76	72	4	1	4.5	A
75317020	Vegetables, stew type, cooked, no added fat	3000_Vegetables	76	72	4	1	4	B
73111201	Peas and carrots, cooked, from fresh, NS as to fat added in cooking	3000_Vegetables	76	71	5	1	4	A
75224048	Peas, green, cooked, from frozen, made with margarine	3000_Vegetables	76	71	5	2	4.5	B
75223011	Peas, cowpeas, field peas, or blackeye peas, not dried, cooked, from fresh, fat not added in cooking	3000_Vegetables	76	69	7	1	4.5	A
75315110	Peas and onions, cooked, fat not added in cooking	3000_Vegetables	76	69	7	1	4	B
75224020	Peas, green, cooked, NS as to form, fat not added in cooking	3000_Vegetables	76	68	8	1	5	A
75534500	Tsukemono, Japanese pickles	3000_Vegetables	75	78	-3	4	3.5	B
75233001	Squash, summer, yellow or green, cooked, from fresh, NS as to fat added in cooking	3000_Vegetables	75	77	-2	2	4	A
75311032	Mixed vegetables, cooked, from canned, made with margarine	3000_Vegetables	75	77	-2	3	4	A
75205033	Green beans, canned, cooked, fat added, NS as to fat type	3000_Vegetables	75	76	-1	2	4	A
75218400	Leek, cooked	3000_Vegetables	75	76	-1	1	4	B
75311030	Classic mixed vegetables, canned, cooked with oil	3000_Vegetables	75	76	-1	3	4.5	A
73402000	Sweet potato, baked, peel eaten, NS as to fat	3000_Vegetables	75	74	1	1	3.5	B
73402020	Sweet potato, baked, peel eaten, fat added, NS as to fat type	3000_Vegetables	75	74	1	1	3.5	B
73403000	Sweet potato, baked, peel not eaten, NS as to fat	3000_Vegetables	75	74	1	1	3.5	B
73403020	Sweet potato, baked, peel not eaten, fat added, NS as to fat type	3000_Vegetables	75	74	1	1	3.5	B
75310990	Classic mixed vegetables, cooked, from restaurant	3000_Vegetables	75	74	1	2	4	B
75218499	Lotus root, cooked, NS as to fat added in cooking	3000_Vegetables	75	73	2	1	4.5	C
75330151	Vegetable combination, excluding carrots, broccoli, and dark-green leafy; cooked, no sauce, made with oil	3000_Vegetables	75	73	2	1	4	A
75223033	Peas, cowpeas, field peas, or blackeye peas, not dried, cooked, from canned, made with oil	3000_Vegetables	75	71	4	1	5	A
75231027	Snowpea, cooked, from fresh, made with butter	3000_Vegetables	75	71	4	1	4	A
73201034	Pumpkin, cooked, from canned, made with butter	3000_Vegetables	75	70	5	1	3.5	B
75201000	Artichoke, cooked, NS as to form, NS as to fat added in cooking	3000_Vegetables	75	69	6	1	4	A

75204011	Beans, lima, immature, cooked, from fresh, fat not added in cooking	3000_Vegetables	75	69	6	1	5	A
75225010	Pigeon peas, cooked, NS as to form, fat not added in cooking	3000_Vegetables	75	69	6	1	5	A
75225011	Pigeon peas, cooked, from fresh, fat not added in cooking	3000_Vegetables	75	69	6	1	5	A
75225013	Pigeon peas, cooked, from canned, fat not added in cooking	3000_Vegetables	75	69	6	1	5	A
75221010	Onions, cooked, NS as to form, fat not added in cooking	3000_Vegetables	75	67	8	1	3.5	A
73410330	Sweet potato fries, from fresh, fried	3000_Vegetables	74	77	-3	2	3.5	C
75216721	Cucumber, cooked, made with oil	3000_Vegetables	74	77	-3	2	3.5	A
75233028	Summer squash, yellow or green, fresh, cooked with butter or margarine	3000_Vegetables	74	76	-2	2	4	A
72203090	Broccoli, chinese, cooked, NS as to form, made with butter	3000_Vegetables	74	75	-1	2	4	B
72203130	Broccoli, chinese, cooked, from fresh, made with butter	3000_Vegetables	74	75	-1	2	4	B
72203170	Broccoli, chinese, cooked, from frozen, made with butter	3000_Vegetables	74	75	-1	2	4	B
75233023	Summer squash, yellow or green, canned, cooked, fat added, NS as to fat type	3000_Vegetables	74	75	-1	2	3.5	A
75208110	Beets, canned, reduced sodium, cooked	3000_Vegetables	74	74	0	2	4	A
73111223	Peas and carrots, canned, cooked, fat added	3000_Vegetables	74	73	1	3	4.5	A
74504100	Tomato and onion, cooked, NS as to fat added in cooking	3000_Vegetables	74	73	1	2	3.5	A
74504120	Tomato and onion, cooked, fat added in cooking, NS as to type of fat	3000_Vegetables	74	73	1	2	3.5	A
75221017	Onions, cooked, from fresh, made with oil	3000_Vegetables	74	73	1	1	3.5	A
75233000	Squash, summer, yellow or green, cooked, NS as to form, NS as to fat added in cooking	3000_Vegetables	74	73	1	2	4	A
75233002	Squash, summer, yellow or green, cooked, from frozen, NS as to fat added in cooking	3000_Vegetables	74	73	1	2	4	A
75206021	Beans, string, yellow, cooked, from fresh, fat added in cooking, NS as to type of fat	3000_Vegetables	74	72	2	2	4.5	C
75311013	Classic mixed vegetables, canned, cooked, no added fat	3000_Vegetables	74	71	3	3	4.5	A
75330122	Vegetable combination, including carrots, broccoli, and/or dark-green leafy; cooked, no sauce, made with butter	3000_Vegetables	74	71	3	1	4	A
73102200	Carrots, cooked, NS as to form, NS as to fat added in cooking	3000_Vegetables	74	70	4	2	4	A
73102201	Carrots, cooked, from fresh, NS as to fat added in cooking	3000_Vegetables	74	70	4	2	4	A
73102202	Carrots, cooked, from frozen, NS as to fat added in cooking	3000_Vegetables	74	70	4	2	4	A
75224032	Green peas, frozen, cooked, fat added, NS as to fat type	3000_Vegetables	74	70	4	1	5	A
75221011	Onions, cooked, no added fat	3000_Vegetables	74	68	6	1	4	A
75224011	Peas, green, cooked, from fresh, NS as to fat added in cooking	3000_Vegetables	74	68	6	1	4.5	B
75221032	Onions, pearl, cooked, from frozen	3000_Vegetables	74	67	7	1	4	A
75330140	Vegetable combination, excluding carrots, broccoli, and dark-green leafy; cooked, no sauce, fat not added in cooking	3000_Vegetables	74	67	7	1	4	A
75214001	Cauliflower, cooked, from fresh, NS as to fat added in cooking	3000_Vegetables	73	75	-2	2	4	C
75234021	Turnip, cooked	3000_Vegetables	73	72	1	2	4	A
73405100	Sweet potato, boiled with peel, peel not eaten, NS as to fat added in cooking	3000_Vegetables	73	71	2	2	3.5	C
73405120	Sweet potato, boiled with peel, peel not eaten, fat added in cooking	3000_Vegetables	73	71	2	2	3.5	C
75302511	Beans, string, green, and potatoes, cooked, made with oil	3000_Vegetables	73	71	2	1	4	A

73111200	Peas and carrots, cooked, NS as to form, NS as to fat added in cooking	3000_Vegetables	73	69	4	1	4.5	A
73111202	Peas and carrots, cooked, from frozen, NS as to fat added in cooking	3000_Vegetables	73	69	4	1	4.5	A
75208013	Beets, canned, cooked, no added fat	3000_Vegetables	73	69	4	3	3.5	A
75224132	Green peas, canned, reduced sodium, cooked with butter or margarine	3000_Vegetables	73	69	4	1	5	B
75223013	Peas, cowpeas, field peas, or blackeye peas, not dried, cooked, from canned, fat not added in cooking	3000_Vegetables	73	66	7	1	5	A
75315300	Peas and potatoes, cooked, fat not added in cooking	3000_Vegetables	73	66	7	1	4.5	C
73111235	Peas and carrots, cooked, from canned, made with margarine	3000_Vegetables	72	73	-1	3	4	A
75143200	Lettuce, salad with cheese, tomato and/or carrots, with or without other vegetables, no dressing	3000_Vegetables	72	73	-1	3	4	A
73102228	Carrots, canned, cooked with butter or margarine	3000_Vegetables	72	72	0	3	4	B
75233031	Summer squash, yellow or green, frozen, cooked with butter or margarine	3000_Vegetables	72	72	0	2	4	A
75316020	Squash, summer, yellow or green, and onions, cooked, fat added in cooking	3000_Vegetables	72	72	0	2	3.5	A
73405023	Sweet potato, boiled, made with margarine	3000_Vegetables	72	71	1	2	3.5	B
75307003	Green peppers and onions, cooked, made with margarine	3000_Vegetables	72	71	1	2	4	B
75211010	Cabbage, green, cooked, NS as to fat added in cooking	3000_Vegetables	72	70	2	2	4	A
73201025	Pumpkin, cooked, NS as to form, made with butter	3000_Vegetables	72	69	3	1	3.5	B
73201028	Pumpkin, cooked, from fresh, made with butter	3000_Vegetables	72	69	3	1	3.5	B
73201031	Pumpkin, cooked, from frozen, made with butter	3000_Vegetables	72	69	3	1	3.5	B
74503010	Tomato and corn, cooked, fat not added in cooking	3000_Vegetables	72	69	3	3	4	B
75204980	Beans, string, cooked, NS as to form, NS as to color, fat added in cooking, NS as to type of fat	3000_Vegetables	72	69	3	2	4	A
75205000	Beans, string, cooked, NS as to form, NS as to color, NS as to fat added in cooking	3000_Vegetables	72	69	3	2	4	A
75205010	Beans, string, green, cooked, NS as to form, NS as to fat added in cooking	3000_Vegetables	72	69	3	2	4	A
75330153	Vegetable combination, excluding carrots, broccoli, and dark-green leafy; cooked, no sauce, made with margarine	3000_Vegetables	72	69	3	2	4	A
71104010	White potato, roasted, fat not added in cooking	3000_Vegetables	72	68	4	1	4.5	B
75109600	Corn, raw	3000_Vegetables	72	68	4	1	5	B
75208021	Beets, fresh, cooked, fat added	3000_Vegetables	72	68	4	1	4	B
73405010	Sweet potato, boiled, no added fat	3000_Vegetables	72	66	6	1	3.5	B
75208010	Beets, cooked, NS as to form, fat not added in cooking	3000_Vegetables	72	65	7	1	4	A
75503030	Cucumber pickles, sour	3000_Vegetables	71	76	-5	4	2.5	D
71104070	Potato, roasted, from fresh, peel eaten, made with oil	3000_Vegetables	71	70	1	1	4.5	B
71704000	Stewed potatoes with tomatoes	3000_Vegetables	71	70	1	1	4.5	B
73410110	Sweet potato, fried	3000_Vegetables	71	70	1	1	3.5	C
71961010	Celeriac, cooked	3000_Vegetables	71	69	2	2	4	B
74505000	Tomato with corn and okra, cooked, NS as to fat added in cooking	3000_Vegetables	71	68	3	2	4	B
74505020	Tomato with corn and okra, cooked, fat added in cooking, NS as to type of fat	3000_Vegetables	71	68	3	2	4	B

75218501	Lotus root, cooked	3000_Vegetables	71	68	3	1	4.5	B
73303022	Squash, winter type, baked, no sugar added in cooking, made with butter	3000_Vegetables	71	67	4	1	3.5	B
75204982	Beans, string, cooked, from frozen, NS as to color, fat added in cooking, NS as to type of fat	3000_Vegetables	71	67	4	2	4	A
75205012	Beans, string, green, cooked, from frozen, NS as to fat added in cooking	3000_Vegetables	71	67	4	2	4	A
75206000	Beans, string, yellow, cooked, NS as to form, NS as to fat added in cooking	3000_Vegetables	71	67	4	2	4	A
75206010	Beans, string, yellow, cooked, NS as to form, fat not added in cooking	3000_Vegetables	71	67	4	2	4	A
75206022	Beans, string, yellow, cooked, from frozen, fat added in cooking, NS as to type of fat	3000_Vegetables	71	67	4	2	4	B
75204030	Beans, lima, immature, cooked, from frozen, made with oil	3000_Vegetables	71	66	5	1	4.5	A
75215509	Christophine, cooked, NS as to fat added in cooking	3000_Vegetables	71	66	5	2	4	A
75223023	Blackeyed peas, from canned	3000_Vegetables	71	66	5	1	5	A
75224030	Green peas, NS as to form, cooked	3000_Vegetables	71	66	5	1	5	A
75315023	Peas and corn, cooked, made with margarine	3000_Vegetables	71	66	5	1	4.5	C
73421000	Sweet potato, yellow, Puerto Rican, cooked	3000_Vegetables	71	65	6	1	4	B
75221031	Onions, pearl, cooked, from fresh	3000_Vegetables	71	65	6	1	4	B
75221033	Onions, pearl, cooked, from canned	3000_Vegetables	71	65	6	1	4	B
75302050	Beans, string, green, and potatoes, cooked, fat not added in cooking	3000_Vegetables	71	64	7	1	4.5	A
75222010	Parsnips, cooked, fat not added in cooking	3000_Vegetables	71	63	8	1	4.5	B
75507000	Okra, pickled	3000_Vegetables	70	73	-3	3	3	C
73102203	Carrots, cooked, from canned, NS as to fat added in cooking	3000_Vegetables	70	71	-1	3	4	A
75214023	Cauliflower, cooked, from canned, fat added in cooking, NS as to type of fat	3000_Vegetables	70	71	-1	2	4	A
71104000	White potato, roasted, NS as to fat added in cooking	3000_Vegetables	70	70	0	1	4.5	C
71104020	White potato, roasted, fat added in cooking	3000_Vegetables	70	70	0	1	4.5	C
75221019	Onions, cooked, from fresh, made with margarine	3000_Vegetables	70	68	2	2	3.5	B
75317010	Vegetables, stew type, cooked, fat added	3000_Vegetables	70	68	2	1	4	B
71905000	Ripe plantain, raw	3000_Vegetables	70	67	3	1	5	C
75204981	Beans, string, cooked, from fresh, NS as to color, fat added in cooking, NS as to type of fat	3000_Vegetables	70	67	3	2	4.5	A
75205001	Beans, string, cooked, from fresh, NS as to color, NS as to fat added in cooking	3000_Vegetables	70	67	3	2	4.5	A
75205011	Beans, string, green, cooked, from fresh, NS as to fat added in cooking	3000_Vegetables	70	67	3	2	4.5	A
75302200	Beans, string, green, with onions, NS as to fat added in cooking	3000_Vegetables	70	67	3	2	4	A
75302210	Beans, string, green, with onions, cooked, fat added in cooking, NS as to type of fat	3000_Vegetables	70	67	3	2	4	A
75222020	Parsnips, cooked	3000_Vegetables	70	65	5	1	4.5	A
75223000	Peas, cowpeas, field peas, or blackeye peas, not dried, cooked, NS as to form, NS as to fat added in cooking	3000_Vegetables	70	64	6	1	5	A
75223001	Peas, cowpeas, field peas, or blackeye peas, not dried, cooked, from fresh, NS as to fat added in cooking	3000_Vegetables	70	64	6	1	5	A

75223002	Peas, cowpeas, field peas, or blackeye peas, not dried, cooked, from frozen, NS as to fat added in cooking	3000_Vegetables	70	64	6	1	5	A
75223020	Peas, cowpeas, field peas, or blackeye peas, not dried, cooked, NS as to form, fat added in cooking, NS as to type of fat	3000_Vegetables	70	64	6	1	5	A
75224023	Green peas, canned, cooked, no added fat	3000_Vegetables	70	64	6	3	5	A
75218500	Lotus root, cooked, fat not added in cooking	3000_Vegetables	70	63	7	1	4.5	B
75204012	Lima beans, from frozen, no added fat	3000_Vegetables	70	62	8	1	5	A
75216720	Cucumber, cooked	3000_Vegetables	69	71	-2	2	4	A
71104130	Potato, roasted, from fresh, peel not eaten, made with oil	3000_Vegetables	69	69	0	1	4.5	B
75235750	Winter melon, cooked	3000_Vegetables	69	69	0	2	4	A
71103135	Potato, boiled, from fresh, peel eaten, made with oil	3000_Vegetables	69	68	1	1	4.5	B
75226040	Peppers, red, cooked, NS as to fat added in cooking	3000_Vegetables	69	68	1	2	4	A
75311023	Classic mixed vegetables, canned, cooked, fat added, NS as to fat type	3000_Vegetables	69	68	1	3	4.5	A
73405000	Sweet potato, boiled, NS as to fat	3000_Vegetables	69	67	2	1	3.5	C
73405020	Sweet potato, boiled, fat added, NS as to fat type	3000_Vegetables	69	67	2	1	3.5	C
75221030	Onions, pearl, cooked	3000_Vegetables	69	67	2	2	4	A
75315200	Peas and mushrooms, cooked, NS as to fat added in cooking	3000_Vegetables	69	64	5	1	4	C
75315215	Peas and mushrooms, cooked, fat added in cooking, NS as to type of fat	3000_Vegetables	69	64	5	1	4	C
73405110	Sweet potato, boiled with peel, peel not eaten, fat not added in cooking	3000_Vegetables	69	63	6	1	3.5	B
75302050	Beans, string, green, and potatoes, cooked, fat not added in cooking	3000_Vegetables	69	63	6	1	4.5	A
75311028	Classic mixed vegetables, frozen, cooked with butter or margarine	3000_Vegetables	69	62	7	1	4.5	A
75315010	Peas and corn, cooked, no added fat	3000_Vegetables	69	62	7	1	4.5	A
75220049	Lettuce, cooked, NS as to fat added in cooking	3000_Vegetables	68	69	-1	2	4	A
71103030	Potato, boiled, from fresh, peel not eaten, made with oil	3000_Vegetables	68	68	0	1	4.5	B
75204983	Beans, string, cooked, from canned, NS as to color, fat added in cooking, NS as to type of fat	3000_Vegetables	68	68	0	2	4	A
75205003	Beans, string, cooked, from canned, NS as to color, NS as to fat added in cooking	3000_Vegetables	68	68	0	2	4	A
75205013	Beans, string, green, cooked, from canned, NS as to fat added in cooking	3000_Vegetables	68	68	0	2	4	A
75315999	Squash, summer, yellow or green, and onions, cooked, NS as to fat added in cooking	3000_Vegetables	68	67	1	2	3.5	A
75510030	Olives, stuffed	3000_Vegetables	68	67	1	3	2.5	D
71703990	Stewed potatoes	3000_Vegetables	68	66	2	1	4.5	B
75221021	Onions, cooked, fat added	3000_Vegetables	68	66	2	2	3.5	A
75302513	Beans, string, green, and potatoes, cooked, made with margarine	3000_Vegetables	68	66	2	2	4	A
75208120	Beets, canned, low sodium, fat added in cooking, NS as to type of fat	3000_Vegetables	68	65	3	2	4	A
71901010	Green plantains, boiled	3000_Vegetables	68	64	4	1	4	C
73407050	Sweet potato, canned, no added fat	3000_Vegetables	68	63	5	1	3.5	B
75204032	Beans, lima, immature, cooked, from frozen, made with margarine	3000_Vegetables	68	62	6	1	4.5	A

75224047	Green peas, frozen, cooked with butter or margarine	3000_Vegetables	68	62	6	1	4.5	B
75200100	Vegetables, NS as to type, cooked, NS as to fat added in cooking	3000_Vegetables	68	61	7	1	4.5	A
75200120	Vegetables, NS as to type, cooked, fat added in cooking, NS as to type of fat	3000_Vegetables	68	61	7	1	4.5	A
75311000	Mixed vegetables, cooked, NS as to form, NS as to fat added in cooking	3000_Vegetables	68	61	7	1	4.5	A
75311002	Mixed vegetables, cooked, from frozen, NS as to fat added in cooking	3000_Vegetables	68	61	7	1	4.5	A
75204010	Beans, lima, immature, cooked, NS as to form, fat not added in cooking	3000_Vegetables	68	59	9	1	4.5	A
75204013	Beans, lima, immature, cooked, from canned, fat not added in cooking	3000_Vegetables	68	59	9	1	4.5	A
75205051	Green beans, canned, cooked with butter or margarine	3000_Vegetables	67	68	-1	2	4	A
75233220	Spaghetti squash, cooked	3000_Vegetables	67	68	-1	2	4	A
75233223	Squash, spaghetti, cooked, made with margarine	3000_Vegetables	67	68	-1	2	4	A
71910310	Pickled green bananas, Puerto Rican style	3000_Vegetables	67	67	0	2	3	C
75226000	Peppers, green, cooked, NS as to fat added in cooking	3000_Vegetables	67	66	1	2	4	A
75228000	Rutabaga, cooked, NS as to fat added in cooking	3000_Vegetables	67	65	2	2	4	B
75311003	Mixed vegetables, cooked, from canned, NS as to fat added in cooking	3000_Vegetables	67	65	2	3	4	B
73111225	Peas and carrots, cooked, NS as to form, made with butter	3000_Vegetables	67	62	5	1	4.5	A
73111228	Peas and carrots, cooked, from fresh, made with butter	3000_Vegetables	67	62	5	1	4.5	A
73111231	Peas and carrots, cooked, from frozen, made with butter	3000_Vegetables	67	62	5	1	4.5	A
75215040	Celery, cooked, made with butter	3000_Vegetables	67	62	5	2	4	A
75225015	Pigeon peas, cooked, NS as to form, fat added in cooking	3000_Vegetables	67	62	5	1	5	B
73102215	Carrots, cooked, NS as to form, made with butter	3000_Vegetables	67	61	6	1	4	A
75225014	Pigeon peas, cooked, NS as to form, NS as to fat added in cooking	3000_Vegetables	67	61	6	1	5	B
72125230	Spinach, creamed	3000_Vegetables	66	68	-2	3	4	C
71905110	Fried ripe plantain, Puerto Rican style	3000_Vegetables	66	67	-1	2	3.5	C
71900200	Plantain, cooked with oil	3000_Vegetables	66	64	2	1	3.5	C
73407060	Sweet potato, canned, fat added	3000_Vegetables	66	64	2	1	3.5	A
75234000	Turnip, cooked, NS as to form, NS as to fat added in cooking	3000_Vegetables	66	64	2	2	4	B
75234003	Turnip, cooked, from canned, NS as to fat added in cooking	3000_Vegetables	66	64	2	2	4	B
75234022	Turnip, cooked, from frozen, fat added in cooking, NS as to type of fat	3000_Vegetables	66	64	2	2	4	A
75513010	Seaweed, pickled	3000_Vegetables	66	64	2	3	3	D
75204033	Beans, lima, immature, cooked, from canned, made with oil	3000_Vegetables	66	63	3	3	4.5	A
75216134	Corn, fresh, cooked with oil	3000_Vegetables	66	63	3	1	4	B
75216137	Corn, frozen, cooked with oil	3000_Vegetables	66	63	3	1	4.5	B
75303021	Corn with peppers, red or green, cooked, made with oil	3000_Vegetables	66	63	3	1	4	B
75315020	Peas and corn, cooked, fat added	3000_Vegetables	66	61	5	1	4.5	B
71103125	Potato, boiled, from fresh, peel eaten, no addded fat	3000_Vegetables	66	60	6	1	4.5	B
71104050	Potato, roasted, from fresh, peel eaten, no added fat	3000_Vegetables	66	60	6	1	4.5	B

75204001	Beans, lima, immature, cooked, from fresh, NS as to fat added in cooking	3000_Vegetables	66	60	6	1	5	B
75204021	Beans, lima, immature, cooked, from fresh, fat added in cooking, NS as to type of fat	3000_Vegetables	66	60	6	1	5	B
75204022	Lima beans, from frozen, fat added	3000_Vegetables	66	60	6	1	5	A
75224010	Peas, green, cooked, NS as to form, NS as to fat added in cooking	3000_Vegetables	66	59	7	1	4.5	C
75224012	Peas, green, cooked, from frozen, NS as to fat added in cooking	3000_Vegetables	66	59	7	1	4.5	C
75301110	Lima beans and corn, cooked, no added fat	3000_Vegetables	66	58	8	1	4.5	A
74204010	Tomatoes, NS as to form, stewed	3000_Vegetables	65	70	-5	4	4	B
74204013	Tomatoes, from canned, stewed	3000_Vegetables	65	70	-5	4	4	A
75208023	Beets, canned, cooked, fat added	3000_Vegetables	65	64	1	3	3.5	B
75510010	Olives, green	3000_Vegetables	65	64	1	3	2.5	D
71962040	Taro, cooked	3000_Vegetables	65	61	4	1	4.5	A
73111203	Peas and carrots, cooked, from canned, NS as to fat added in cooking	3000_Vegetables	65	61	4	3	4	A
75224033	Green peas, canned, cooked, fat added, NS as to fat type	3000_Vegetables	65	61	4	3	4.5	B
75233025	Squash, summer, yellow or green, cooked, NS as to form, made with butter	3000_Vegetables	65	61	4	1	3.5	A
75317000	Vegetables, stew type, cooked, NS as to fat added in cooking	3000_Vegetables	65	61	4	1	4	A
71950010	Tannier, cooked	3000_Vegetables	65	60	5	1	4.5	A
71962020	Dasheen, cooked	3000_Vegetables	65	60	5	1	4.5	A
75301121	Beans, lima and corn, cooked, made with oil	3000_Vegetables	65	60	5	1	4.5	B
71508001	Potato, baked, peel eaten	3000_Vegetables	65	59	6	1	4.5	B
75223003	Peas, cowpeas, field peas, or blackeye peas, not dried, cooked, from canned, NS as to fat added in cooking	3000_Vegetables	65	59	6	1	5	B
75223021	Peas, cowpeas, field peas, or blackeye peas, not dried, cooked, from fresh, fat added in cooking, NS as to type of fat	3000_Vegetables	65	59	6	1	4.5	B
75303010	Corn with peppers, red or green, cooked, fat not added in cooking	3000_Vegetables	65	59	6	1	4	C
71101150	White potato skins, with adhering flesh, baked	3000_Vegetables	65	58	7	1	4.5	B
71941120	Sweet potatoes, white, Puerto Rican, boiled	3000_Vegetables	65	58	7	1	4.5	B
71945010	Yam, cooked, Puerto Rican	3000_Vegetables	65	58	7	1	4.5	B
75223031	Peas, cowpeas, field peas, or blackeye peas, not dried, cooked, from frozen, made with butter	3000_Vegetables	65	58	7	1	5	A
71401010	Potato, french fries, from fresh, fried	3000_Vegetables	64	65	-1	2	4	C
75206003	Beans, string, yellow, cooked, from canned, NS as to fat added in cooking	3000_Vegetables	64	64	0	3	4	A
75206023	Beans, string, yellow, cooked, from canned, fat added in cooking, NS as to type of fat	3000_Vegetables	64	64	0	3	4	A
75510000	Olives, NFS	3000_Vegetables	64	63	1	3	3	C
75510020	Olives, black	3000_Vegetables	64	63	1	3	3	C
71103150	Potato, boiled, from fresh, peel eaten, made with margarine	3000_Vegetables	64	61	3	2	4.5	C
71104090	Potato, roasted, from fresh, peel eaten, made with margarine	3000_Vegetables	64	61	3	2	4.5	C
73401000	Sweet potato, NFS	3000_Vegetables	64	61	3	1	3.5	C
75216310	Corn, canned, reduced sodium, cooked, no added fat	3000_Vegetables	64	61	3	3	4.5	B

75224051	Peas, green, cooked, from canned, made with margarine	3000_Vegetables	64	61	3	3	4.5	B
75216024	Corn, cooked, NS as to form, NS as to color, made with oil	3000_Vegetables	64	60	4	1	4	B
75216027	Corn, cooked, from fresh, NS as to color, made with oil	3000_Vegetables	64	60	4	1	4.5	C
75216131	Corn, yellow, cooked, NS as to form, made with oil	3000_Vegetables	64	60	4	1	4	B
75216177	Corn, yellow and white, cooked, from fresh, made with oil	3000_Vegetables	64	60	4	1	4	C
75234001	Turnip, cooked, from fresh, NS as to fat added in cooking	3000_Vegetables	64	60	4	2	4	B
75216111	Corn, fresh, cooked, no added fat	3000_Vegetables	64	57	7	1	4.5	B
71103320	Potato, canned, no added fat	3000_Vegetables	64	56	8	1	4.5	B
73410300	Sweet potato fries, NS as to fresh or frozen	3000_Vegetables	63	68	-5	3	4	C
73410310	Sweet potato fries, frozen, fried	3000_Vegetables	63	68	-5	3	4	B
71603010	Potato salad, made with mayonnaise	3000_Vegetables	63	66	-3	3	4	C
71401015	Potato, french fries, from fresh, baked	3000_Vegetables	63	63	0	2	4	C
71905120	Plantain, ripe, rolled in flour, fried	3000_Vegetables	63	63	0	1	4	C
71104150	Potato, roasted, from fresh, peel not eaten, made with margarine	3000_Vegetables	63	61	2	2	4.5	C
75204970	Beans, string, cooked, from canned, NS as to color, made with butter	3000_Vegetables	63	61	2	2	4	A
75226062	Peppers, red, cooked, made with butter	3000_Vegetables	63	60	3	1	4	A
71701000	Potato pancake	3000_Vegetables	63	59	4	1	4	C
73402022	Sweet potato, baked, peel eaten, made with butter	3000_Vegetables	63	59	4	1	3.5	C
75216227	Corn, white, cooked, from fresh, made with oil	3000_Vegetables	63	59	4	1	4	C
71103010	Potato, boiled, from fresh, peel not eaten, no added fat	3000_Vegetables	63	58	5	1	4.5	B
71941130	Sweet potatoes, white, Puerto Rican, roasted or baked	3000_Vegetables	63	58	5	2	4	B
73403022	Sweet potato, baked, peel not eaten, made with butter	3000_Vegetables	63	58	5	1	3.5	C
75301120	Lima beans and corn, cooked, fat added	3000_Vegetables	63	58	5	1	4.5	A
75315100	Peas and onions, cooked, NS as to fat added in cooking	3000_Vegetables	63	58	5	1	4	B
75315120	Peas and onions, cooked, fat added in cooking, NS as to type of fat	3000_Vegetables	63	58	5	1	4	B
71101110	White potato, baked, peel eaten, fat not added in cooking	3000_Vegetables	63	57	6	1	4.5	C
71104120	Potato, roasted, from fresh, peel not eaten, no added fat	3000_Vegetables	63	57	6	1	4.5	B
75204023	Lima beans, from canned	3000_Vegetables	63	57	6	1	4.5	B
75204964	Beans, string, cooked, from fresh, NS as to color, made with butter	3000_Vegetables	63	57	6	1	4.5	A
75205042	Beans, string, green, cooked, NS as to form, made with butter	3000_Vegetables	63	57	6	1	4	A
71103110	White potato, boiled with peel, peel not eaten, fat not added in cooking	3000_Vegetables	63	56	7	1	4.5	C
75204967	Beans, string, cooked, from frozen, NS as to color, made with butter	3000_Vegetables	63	56	7	1	4	A
75206037	Beans, string, yellow, cooked, from frozen, made with butter	3000_Vegetables	63	56	7	1	4	A
71603030	Potato salad, made with creamy dressing	3000_Vegetables	62	66	-4	3	4	C
71403500	Potato, home fries, with vegetables	3000_Vegetables	62	63	-1	2	4	C
73407000	Sweet potato, canned, NS as to fat	3000_Vegetables	62	62	0	3	3.5	C
71403000	White potato, home fries	3000_Vegetables	62	61	1	2	4	C
71103050	Potato, boiled, from fresh, peel not eaten, made with margarine	3000_Vegetables	62	60	2	2	4.5	C

71103105	Potato, boiled, from fresh, peel eaten, NS as to fat	3000_Vegetables	62	59	3	1	4.5	C
71104040	Potato, roasted, from fresh, peel eaten, NS as to fat	3000_Vegetables	62	59	3	1	4.5	C
71104060	Potato, roasted, from fresh, peel eaten, fat added, NS as to fat type	3000_Vegetables	62	59	3	1	4.5	C
71603045	Potato salad, made with light Italian dressing	3000_Vegetables	62	59	3	2	4	C
74504122	Tomato and onion, cooked, made with butter	3000_Vegetables	62	59	3	1	3.5	B
75311031	Classic mixed vegetables, canned, cooked with butter or margarine	3000_Vegetables	62	59	3	3	4	A
71103115	Potato, boiled, from fresh, peel eaten, fat added, NS as to fat type	3000_Vegetables	62	58	4	1	4.5	C
74505022	Tomato with corn and okra, cooked, made with butter	3000_Vegetables	62	58	4	2	4	C
71101000	Potato, baked, peel not eaten	3000_Vegetables	62	57	5	1	4.5	C
75216224	Corn, white, cooked, NS as to form, made with oil	3000_Vegetables	62	57	5	1	4	B
75216232	Corn, white, cooked, from frozen, made with oil	3000_Vegetables	62	57	5	1	4	B
71100100	Potato, baked, NFS	3000_Vegetables	62	56	6	1	4.5	C
75221000	Onions, cooked, NS as to form, NS as to fat added in cooking	3000_Vegetables	62	56	6	2	3.5	A
75221002	Onions, cooked, from frozen, NS as to fat added in cooking	3000_Vegetables	62	56	6	2	3.5	A
75221020	Onions, cooked, NS as to form, fat added in cooking, NS as to type of fat	3000_Vegetables	62	56	6	2	3.5	A
75221022	Onions, cooked, from frozen, fat added in cooking, NS as to type of fat	3000_Vegetables	62	56	6	2	3.5	A
75301123	Beans, lima and corn, cooked, made with margarine	3000_Vegetables	62	56	6	1	4.5	B
75315305	Peas and potatoes, cooked, NS as to fat added in cooking	3000_Vegetables	62	56	6	1	4.5	C
75315310	Peas and potatoes, cooked, fat added in cooking, NS as to type of fat	3000_Vegetables	62	56	6	1	4.5	C
75330130	Vegetable combination, excluding carrots, broccoli, and dark-green leafy; cooked, no sauce, NS as to fat added in cooking	3000_Vegetables	62	56	6	1	4	A
75330150	Vegetable combination, excluding carrots, broccoli, and dark-green leafy; cooked, no sauce, fat added in cooking, NS as to type of fat	3000_Vegetables	62	56	6	1	4	A
75216011	Corn, cooked, from fresh, NS as to color, fat not added in cooking	3000_Vegetables	62	55	7	1	4.5	C
75216211	Corn, white, cooked, from fresh, fat not added in cooking	3000_Vegetables	62	55	7	1	4	C
75315000	Peas and corn, cooked, NS as to fat added in cooking	3000_Vegetables	62	55	7	1	4.5	C
71962010	Dasheen, boiled	3000_Vegetables	62	54	8	1	4.5	B
75216112	Corn, frozen, cooked, no added fat	3000_Vegetables	62	54	8	1	4.5	B
75311025	Mixed vegetables, cooked, NS as to form, made with butter	3000_Vegetables	62	54	8	1	4	A
75208500	Breadfruit, cooked, fat not added in cooking	3000_Vegetables	62	53	9	1	4	C
73410200	Sweet potato fries, NFS	3000_Vegetables	61	66	-5	3	4	C
71602950	Potato salad, from restaurant	3000_Vegetables	61	64	-3	3	4	C
73303040	Squash, winter type, baked, no fat added in cooking, sugar added in cooking	3000_Vegetables	61	62	-1	1	3.5	B
71403040	Potato, home fries, from fresh	3000_Vegetables	61	61	0	2	4	C
71405010	Potato, hash brown, from fresh	3000_Vegetables	61	61	0	2	4	C
71601030	Potato salad with egg, made with creamy dressing	3000_Vegetables	61	61	0	2	3.5	C
74205011	Tomatoes, green, cooked, from fresh	3000_Vegetables	61	61	0	2	3.5	C
71603035	Potato salad, made with light creamy dressing	3000_Vegetables	61	60	1	2	4	C

71410000	Potato skins without topping	3000_Vegetables	61	59	2	2	4	C
75217000	Eggplant, cooked, NS as to fat added in cooking	3000_Vegetables	61	57	4	2	4	B
75316023	Squash, summer, yellow or green, and onions, cooked, made with butter	3000_Vegetables	61	57	4	1	3.5	A
71103200	White potato, boiled, without peel, canned, low sodium, NS as to fat added in cooking	3000_Vegetables	61	56	5	1	4.5	A
71103220	White potato, boiled, without peel, canned, low sodium, fat added in cooking	3000_Vegetables	61	56	5	1	4.5	A
71900100	Plantain, cooked, no added fat	3000_Vegetables	61	55	6	1	4	C
75208520	Breadfruit, fried	3000_Vegetables	61	55	6	1	4	C
75216179	Corn, yellow and white, cooked, from fresh, made with margarine	3000_Vegetables	61	55	6	1	4	C
75216171	Corn, yellow and white, cooked, from fresh, fat not added in cooking	3000_Vegetables	61	54	7	1	4	C
75224041	Peas, green, cooked, NS as to form, made with butter	3000_Vegetables	61	53	8	1	4.5	C
71403020	Potato, home fries, NFS	3000_Vegetables	60	61	-1	2	4	C
71603025	Potato salad, made with light mayonnaise-type salad dressing	3000_Vegetables	60	61	-1	2	4	C
71600950	Potato salad with egg, from restaurant	3000_Vegetables	60	60	0	3	3.5	C
75440200	Vegetable tempura	3000_Vegetables	60	59	1	2	3.5	A
71102990	Potato, boiled, ready-to-heat	3000_Vegetables	60	57	3	1	4.5	C
71103000	Potato, boiled, from fresh, peel not eaten, NS as to fat	3000_Vegetables	60	57	3	1	4.5	C
71103020	Potato, boiled, from fresh, peel not eaten, fat added, NS as to fat type	3000_Vegetables	60	57	3	1	4.5	C
71104030	Potato, roasted, NFS	3000_Vegetables	60	57	3	1	4.5	C
71104100	Potato, roasted, from fresh, peel not eaten, NS as to fat	3000_Vegetables	60	57	3	1	4.5	C
71104110	Potato, roasted, from fresh, peel not eaten, fat added, NS as to fat type	3000_Vegetables	60	57	3	1	4.5	C
71104200	Potato, roasted, ready-to-heat	3000_Vegetables	60	57	3	1	4.5	C
71910210	Green banana, fried	3000_Vegetables	60	57	3	1	4	C
71103300	Potato, canned, NS as to fat	3000_Vegetables	60	56	4	2	4	C
71103310	Potato, canned, fat added, NS as to fat type	3000_Vegetables	60	56	4	2	4	C
71703040	Stewed potatoes with tomatoes, Mexican style (Papas guisadas con tomate)	3000_Vegetables	60	56	4	2	4	C
75216121	Corn, fresh, cooked, fat added, NS as to fat type	3000_Vegetables	60	56	4	1	4	C
71106000	Stewed potatoes, Puerto Rican style	3000_Vegetables	60	55	5	1	4	C
71910110	Green banana, cooked in salt water	3000_Vegetables	60	55	5	1	4	C
75216026	Corn, cooked, NS as to form, NS as to color, made with margarine	3000_Vegetables	60	55	5	1	4	C
75216033	Corn, cooked, from frozen, NS as to color, made with margarine	3000_Vegetables	60	55	5	1	4	C
75216122	Corn, frozen, cooked, fat added, NS as to fat type	3000_Vegetables	60	55	5	1	4.5	B
75216133	Corn, yellow, cooked, NS as to form, made with margarine	3000_Vegetables	60	55	5	1	4	C
75216136	Corn, yellow, cooked, from fresh, made with margarine	3000_Vegetables	60	55	5	1	4.5	C
75216139	Corn, yellow, cooked, from frozen, made with margarine	3000_Vegetables	60	55	5	1	4	C
75216229	Corn, white, cooked, from fresh, made with margarine	3000_Vegetables	60	55	5	1	4	C
75221001	Onions, cooked, from fresh, NS as to fat added in cooking	3000_Vegetables	60	55	5	1	3.5	A

75228022	Rutabaga, cooked, made with butter	3000_Vegetables	60	55	5	1	3.5	C
75208000	Beets, cooked, NS as to form, NS as to fat added in cooking	3000_Vegetables	60	54	6	1	3.5	A
75208001	Beets, cooked, from fresh, NS as to fat added in cooking	3000_Vegetables	60	54	6	1	3.5	A
75234028	Turnip, cooked, from fresh, made with butter	3000_Vegetables	60	54	6	1	4	A
75222000	Parsnips, cooked, NS as to fat added in cooking	3000_Vegetables	60	53	7	1	4.5	C
75223028	Peas, cowpeas, field peas, or blackeye peas, not dried, cooked, from fresh, made with butter	3000_Vegetables	60	53	7	1	4.5	B
75223034	Peas, cowpeas, field peas, or blackeye peas, not dried, cooked, from canned, made with butter	3000_Vegetables	60	53	7	1	5	B
75505000	Mushrooms, pickled	3000_Vegetables	59	66	-7	3	3.5	C
71603015	Potato salad, made with light mayonnaise	3000_Vegetables	59	60	-1	2	4	C
75208020	Beets, NS as to form, cooked	3000_Vegetables	59	57	2	3	3.5	A
75216320	Corn, canned, reduced sodium, cooked, fat added, NS as to fat type	3000_Vegetables	59	57	2	3	4.5	B
73111234	Peas and carrots, cooked, from canned, made with butter	3000_Vegetables	59	55	4	3	4	B
74503009	Tomato and corn, cooked, NS as to fat added in cooking	3000_Vegetables	59	55	4	3	4	B
74503011	Tomato and corn, cooked, fat added in cooking	3000_Vegetables	59	55	4	3	4	B
75306999	Green peppers and onions, cooked, NS as to fat added in cooking	3000_Vegetables	59	55	4	2	4	B
77121010	Fried stuffed potatoes, Puerto Rican style	3000_Vegetables	59	55	4	2	4	C
71905008	Plantain, cooked, fat added, NS as to fat type	3000_Vegetables	59	54	5	1	4	C
75224050	Green peas, canned, cooked with butter or margarine	3000_Vegetables	59	54	5	3	4.5	B
75302500	Beans, string, green, and potatoes, cooked, NS as to fat added in cooking	3000_Vegetables	59	54	5	1	4	A
75302510	Beans, string, green, and potatoes, cooked, fat added in cooking, NS as to type of fat	3000_Vegetables	59	54	5	1	4	A
71106010	Potato only from Puerto Rican mixed dishes, gravy and other components reported separately	3000_Vegetables	59	53	6	1	4.5	C
71501080	White potato, from fresh, mashed, not made with milk or fat	3000_Vegetables	59	53	6	1	4	C
71601035	Potato salad with egg, made with light creamy dressing	3000_Vegetables	59	53	6	2	4	B
75302212	Beans, string, green, with onions, cooked, made with butter	3000_Vegetables	59	53	6	1	4	A
71501090	White potato, from dry, mashed, made with milk, no fat	3000_Vegetables	59	52	7	1	4	B
75204000	Beans, lima, immature, cooked, NS as to form, NS as to fat added in cooking	3000_Vegetables	59	51	8	1	4.5	B
75204002	Beans, lima, immature, cooked, from frozen, NS as to fat added in cooking	3000_Vegetables	59	51	8	1	4.5	B
75204020	Beans, lima, immature, cooked, NS as to form, fat added in cooking, NS as to type of fat	3000_Vegetables	59	51	8	1	4.5	B
75502010	Cauliflower, pickled	3000_Vegetables	58	62	-4	3	3	C
71603040	Potato salad, made with Italian dressing	3000_Vegetables	58	58	0	2	4	C
75216323	Corn, yellow, canned, low sodium, made with margarine	3000_Vegetables	58	58	0	3	4.5	B
71603020	Potato salad, made with mayonnaise-type salad dressing	3000_Vegetables	58	57	1	2	4	C
74202051	Tomatoes, red, from fresh, fried	3000_Vegetables	58	57	1	2	3.5	C
71601015	Potato salad with egg, made with light mayonnaise	3000_Vegetables	58	55	3	2	4	B
71601025	Potato salad with egg, made with light mayonnaise-type salad dressing	3000_Vegetables	58	54	4	2	4	B

75215990	Corn, cooked, from restaurant	3000_Vegetables	58	54	4	1	4	C
71508040	White potato, stuffed, baked, peel eaten, stuffed with broccoli and cheese sauce	3000_Vegetables	58	53	5	2	4.5	C
75224013	Peas, green, cooked, from canned, NS as to fat added in cooking	3000_Vegetables	58	53	5	3	4.5	C
75226022	Peppers, green, cooked, made with butter	3000_Vegetables	58	53	5	1	4	B
75317012	Vegetables, stew type, cooked, made with butter	3000_Vegetables	58	53	5	1	4	B
75216226	Corn, white, cooked, NS as to form, made with margarine	3000_Vegetables	58	52	6	1	4	C
75315022	Peas and corn, cooked, made with butter	3000_Vegetables	58	50	8	1	4.5	C
75216010	Corn, cooked, NS as to form, NS as to color, fat not added in cooking	3000_Vegetables	58	49	9	1	4	B
75216012	Corn, cooked, from frozen, NS as to color, fat not added in cooking	3000_Vegetables	58	49	9	1	4	B
75216110	Corn, yellow, cooked, NS as to form, fat not added in cooking	3000_Vegetables	58	49	9	1	4	B
75515100	Vegetables, pickled	3000_Vegetables	57	60	-3	3	3	D
75216141	Corn, canned, cooked with oil	3000_Vegetables	57	55	2	3	4	C
75233210	Squash, spaghetti, cooked, fat added in cooking, NS as to type of fat	3000_Vegetables	57	54	3	2	4	A
71601020	Potato salad with egg, made with mayonnaise-type salad dressing	3000_Vegetables	57	53	4	2	4	B
71601040	Potato salad with egg, made with Italian dressing	3000_Vegetables	57	53	4	2	4	B
71305011	White potato, scalloped, fat not added in cooking	3000_Vegetables	57	51	6	1	4	B
71970200	Fufu	3000_Vegetables	57	51	6	1	4.5	B
73420000	Sweet potato tots, NFS	3000_Vegetables	56	60	-4	4	4	B
75510050	Olive tapenade	3000_Vegetables	56	55	1	3	2	D
75216700	Cucumber, cooked, NS as to fat added in cooking	3000_Vegetables	56	54	2	2	3.5	A
71401031	Potato, french fries, restaurant	3000_Vegetables	56	53	3	1	4	C
71000100	Potato, NFS	3000_Vegetables	56	52	4	1	4.5	C
71102980	Potato, boiled, NFS	3000_Vegetables	56	52	4	1	4.5	C
71507040	White potato, stuffed, baked, peel not eaten, stuffed with broccoli and cheese sauce	3000_Vegetables	56	52	4	2	4	C
71941110	Sweet potatoes, white, Puerto Rican, fried	3000_Vegetables	56	52	4	1	4.5	C
75208003	Beets, cooked, from canned, NS as to fat added in cooking	3000_Vegetables	56	52	4	3	3.5	B
71601045	Potato salad with egg, made with light Italian dressing	3000_Vegetables	56	50	6	1	4	B
73405022	Sweet potato, boiled, made with butter	3000_Vegetables	56	50	6	1	3.5	C
75216135	Corn, fresh, cooked with butter or margarine	3000_Vegetables	56	50	6	1	4	C
71905010	Ripe plantain, boiled	3000_Vegetables	56	49	7	1	4	C
75330152	Vegetable combination, excluding carrots, broccoli, and dark-green leafy; cooked, no sauce, made with butter	3000_Vegetables	56	49	7	1	4	A
71403010	White potato, home fries, fat not added in cooking	3000_Vegetables	56	48	8	1	4	C
75216170	Corn, yellow and white, cooked, NS as to form, fat not added in cooking	3000_Vegetables	56	48	8	1	4.5	B
75216172	Corn, yellow and white, cooked, from frozen, fat not added in cooking	3000_Vegetables	56	48	8	1	4.5	B
75221015	Onions, cooked, NS as to form, made with butter	3000_Vegetables	56	48	8	1	3.5	A
73410320	Sweet potato fries, frozen, baked	3000_Vegetables	55	61	-6	4	4	C
73410500	Sweet potato fries, school	3000_Vegetables	55	61	-6	4	4	B

73420200	Sweet potato tots, school	3000_Vegetables	55	61	-6	4	4	B
75502510	Cabbage, red, pickled	3000_Vegetables	55	59	-4	3	3.5	C
71405019	Potato, hash brown, from fresh, with cheese	3000_Vegetables	55	54	1	2	3.5	C
74204011	Tomatoes, from fresh, stewed	3000_Vegetables	55	54	1	3	4	B
71930190	Yuca fries	3000_Vegetables	55	53	2	2	4	C
77201210	Green plantain with cracklings, Puerto Rican style	3000_Vegetables	55	53	2	2	3	D
71405100	White potato, hash brown, with cheese	3000_Vegetables	55	51	4	1	3.5	D
71305010	White potato, scalloped	3000_Vegetables	55	50	5	2	3.5	C
71101100	White potato, baked, peel eaten, NS as to fat added in cooking	3000_Vegetables	55	49	6	1	4.5	B
71101120	White potato, baked, peel eaten, fat added in cooking	3000_Vegetables	55	49	6	1	4.5	B
75221018	Onions, cooked, from fresh, made with butter	3000_Vegetables	55	49	6	1	3.5	A
75303000	Corn with peppers, red or green, cooked, NS as to fat added in cooking	3000_Vegetables	55	49	6	1	4	C
75303020	Corn with peppers, red or green, cooked, fat added in cooking, NS as to type of fat	3000_Vegetables	55	49	6	1	4	C
75307002	Green peppers and onions, cooked, made with butter	3000_Vegetables	55	49	6	1	3.5	B
71970110	Starchy vegetables, NFS, Puerto Rican style	3000_Vegetables	55	47	8	1	4.5	C
71970120	Starchy vegetables, including tannier, white sweet potato and yam, with green or ripe plantains, Puerto Rican style	3000_Vegetables	55	47	8	1	4.5	C
71970130	Starchy vegetables, including tannier, white sweet potato and yam, no plantain, Puerto Rican style	3000_Vegetables	55	47	8	1	4.5	C
75222022	Parsnips, cooked, made with butter	3000_Vegetables	55	47	8	1	4.5	C
75216210	Corn, white, cooked, NS as to form, fat not added in cooking	3000_Vegetables	55	46	9	1	4.5	B
75216212	Corn, white, cooked, from frozen, fat not added in cooking	3000_Vegetables	55	46	9	1	4.5	B
75500110	Green beans, pickled	3000_Vegetables	54	56	-2	3	3	D
77205110	Ripe plantain fritters, Puerto Rican style	3000_Vegetables	54	50	4	2	3.5	D
71103100	White potato, boiled with peel, peel not eaten, NS as to fat added in cooking	3000_Vegetables	54	48	6	1	4	C
71103120	White potato, boiled with peel, peel not eaten, fat added in cooking	3000_Vegetables	54	48	6	1	4	C
75204003	Beans, lima, immature, cooked, from canned, NS as to fat added in cooking	3000_Vegetables	54	48	6	3	4.5	B
71602010	Potato salad, German style	3000_Vegetables	54	47	7	1	4.5	C
75216138	Corn, frozen, cooked with butter or margarine	3000_Vegetables	54	47	7	1	4	B
75204031	Beans, lima, immature, cooked, from frozen, made with butter	3000_Vegetables	54	45	9	1	4.5	B
73420020	Sweet potato tots, from frozen, baked	3000_Vegetables	53	58	-5	4	4	B
75503080	Eggplant, pickled	3000_Vegetables	53	54	-1	3	3.5	D
73301030	Squash, winter type, mashed, fat and sugar added in cooking	3000_Vegetables	53	53	0	2	3.5	C
71703000	Stewed potatoes, Mexican style (Papas guisadas)	3000_Vegetables	53	49	4	2	4	C
75503085	Ginger root, pickled	3000_Vegetables	53	49	4	4	2.5	C
75216113	Corn, canned, cooked, no added fat	3000_Vegetables	53	48	5	3	4.5	C
71106070	Potato from Puerto Rican chicken fricassee, with sauce	3000_Vegetables	53	47	6	2	4	B
71930130	Cassava, cooked, made with oil	3000_Vegetables	53	47	6	1	4	C

73407030	Sweet potato, canned in syrup, with fat added in cooking	3000_Vegetables	52	58	-6	3	3.5	C
73407020	Sweet potato, canned in syrup	3000_Vegetables	52	56	-4	3	3.5	C
73303030	Squash, winter type, baked, fat and sugar added in cooking	3000_Vegetables	52	53	-1	2	3.5	B
75205200	Fried green beans	3000_Vegetables	52	50	2	2	3.5	A
71508060	White potato, stuffed, baked, peel eaten, stuffed with bacon and cheese	3000_Vegetables	52	49	3	3	4	C
77121110	Potato and ham fritters, Puerto Rican style	3000_Vegetables	52	49	3	2	3.5	C
71501050	White potato, from fresh, mashed, made with milk, fat and cheese	3000_Vegetables	52	48	4	2	3.5	C
75216300	Corn, yellow, canned, low sodium, NS as to fat added in cooking	3000_Vegetables	52	48	4	3	4	B
75216322	Corn, canned, reduced sodium, cooked with butter or margarine	3000_Vegetables	52	48	4	3	4	C
71301021	White potato, cooked, with cheese, fat not added in cooking	3000_Vegetables	52	46	6	2	3.5	C
75302512	Beans, string, green, and potatoes, cooked, made with butter	3000_Vegetables	52	46	6	1	4	A
71905100	Plantain, cooked with butter or margarine	3000_Vegetables	52	45	7	1	4	C
75216000	Corn, cooked, NS as to form, NS as to color, NS as to fat added in cooking	3000_Vegetables	52	45	7	1	4	C
75216001	Corn, cooked, from fresh, NS as to color, NS as to fat added in cooking	3000_Vegetables	52	45	7	1	4	C
75216002	Corn, cooked, from frozen, NS as to color, NS as to fat added in cooking	3000_Vegetables	52	45	7	1	4	C
75216020	Corn, cooked, NS as to form, NS as to color, fat added in cooking, NS as to type of fat	3000_Vegetables	52	45	7	1	4	C
75216021	Corn, cooked, from fresh, NS as to color, fat added in cooking, NS as to type of fat	3000_Vegetables	52	45	7	1	4	C
75216022	Corn, cooked, from frozen, NS as to color, fat added in cooking, NS as to type of fat	3000_Vegetables	52	45	7	1	4	C
75216100	Corn, yellow, cooked, NS as to form, NS as to fat added in cooking	3000_Vegetables	52	45	7	1	4	C
75216101	Corn, yellow, cooked, from fresh, NS as to fat added in cooking	3000_Vegetables	52	45	7	1	4	C
75216102	Corn, yellow, cooked, from frozen, NS as to fat added in cooking	3000_Vegetables	52	45	7	1	4	C
75216161	Corn, yellow and white, cooked, from fresh, NS as to fat added in cooking	3000_Vegetables	52	45	7	1	4	C
75216181	Corn, yellow and white, cooked, from fresh, fat added in cooking, NS as to type of fat	3000_Vegetables	52	45	7	1	4	C
75216201	Corn, white, cooked, from fresh, NS as to fat added in cooking	3000_Vegetables	52	45	7	1	4	C
75216221	Corn, white, cooked, from fresh, fat added in cooking, NS as to type of fat	3000_Vegetables	52	45	7	1	4	C
71601050	Potato salad with egg, made with any type of fat free dressing	3000_Vegetables	52	41	11	1	4	B
75535000	Zucchini, pickled	3000_Vegetables	51	60	-9	3	3	D
75502500	Cabbage, fresh, pickled, Japanese style	3000_Vegetables	51	52	-1	3	3.5	C
72202030	Fried broccoli	3000_Vegetables	51	49	2	2	3.5	B
75216143	Corn, yellow, cooked, from canned, made with margarine	3000_Vegetables	51	49	2	3	4	C
71410500	Potato skins, with cheese	3000_Vegetables	51	48	3	2	3.5	D
75216123	Corn, canned, cooked, fat added, NS as to fat type	3000_Vegetables	51	48	3	3	4	C
71301020	White potato, cooked, with cheese	3000_Vegetables	51	46	5	2	3.5	C
71301000	White potato, cooked, with sauce, NS as to sauce	3000_Vegetables	51	45	6	2	3.5	C

75216013	Corn, cooked, from canned, NS as to color, fat not added in cooking	3000_Vegetables	51	45	6	3	4	C
71508050	White potato, stuffed, baked, peel eaten, stuffed with meat in cream sauce	3000_Vegetables	51	44	7	1	4	C
75216213	Corn, white, cooked, from canned, fat not added in cooking	3000_Vegetables	51	44	7	3	4	C
75303022	Corn with peppers, red or green, cooked, made with butter	3000_Vegetables	51	44	7	1	4	C
73409000	Sweet potato, casserole or mashed	3000_Vegetables	51	43	8	1	3.5	A
71400990	Potato, french fries, NFS	3000_Vegetables	50	53	-3	3	4	C
71401000	Potato, french fries, NS as to fresh or frozen	3000_Vegetables	50	53	-3	3	4	C
75414030	Fried mushrooms	3000_Vegetables	50	48	2	2	4	C
71901110	Fried green plantain, Puerto Rican style	3000_Vegetables	50	47	3	2	2	E
75216036	Corn, cooked, from canned, NS as to color, made with margarine	3000_Vegetables	50	47	3	3	4	C
75412010	Fried eggplant	3000_Vegetables	50	47	3	2	3.5	A
71508035	Potato, baked, peel eaten, with vegetables	3000_Vegetables	50	45	5	2	4	C
75216722	Cucumber, cooked, made with butter	3000_Vegetables	50	45	5	2	3.5	A
71501020	White potato, from fresh, mashed, made with milk and fat	3000_Vegetables	50	44	6	1	4	C
71501030	White potato, from fresh, mashed, made with fat	3000_Vegetables	50	44	6	1	4	C
71501310	White potato, from fresh, mashed, NS as to milk or fat	3000_Vegetables	50	44	6	1	4	C
75216173	Corn, yellow and white, cooked, from canned, fat not added in cooking	3000_Vegetables	50	44	6	3	4	C
71603050	Potato salad, made with any type of fat free dressing	3000_Vegetables	50	43	7	2	4	C
75216162	Corn, yellow and white, cooked, from frozen, NS as to fat added in cooking	3000_Vegetables	50	43	7	1	4	C
75216180	Corn, yellow and white, cooked, NS as to form, fat added in cooking, NS as to type of fat	3000_Vegetables	50	43	7	1	4	C
75216182	Corn, yellow and white, cooked, from frozen, fat added in cooking, NS as to type of fat	3000_Vegetables	50	43	7	1	4	C
75216160	Corn, yellow and white, cooked, NS as to form, NS as to fat added in cooking	3000_Vegetables	50	42	8	1	4	C
75511020	Peppers, pickled	3000_Vegetables	49	53	-4	3	3	C
71508070	White potato, stuffed, baked, peel not eaten, stuffed with bacon and cheese	3000_Vegetables	49	45	4	3	3.5	D
71507035	Potato, baked, peel not eaten, with vegetables	3000_Vegetables	49	44	5	2	4	C
75233222	Squash, spaghetti, cooked, made with butter	3000_Vegetables	49	44	5	1	4	A
71930120	Cassava, cooked	3000_Vegetables	49	43	6	1	4	C
71103140	Potato, boiled, from fresh, peel eaten, made with butter	3000_Vegetables	49	42	7	1	4	C
71104080	Potato, roasted, from fresh, peel eaten, made with butter	3000_Vegetables	49	42	7	1	4	C
71508005	Potato, baked, peel eaten, with butter	3000_Vegetables	49	42	7	1	4	C
71930150	Cassava, cooked, made with margarine	3000_Vegetables	49	42	7	1	4	C
71702000	Potato pudding	3000_Vegetables	49	41	8	2	3	C
75216178	Corn, yellow and white, cooked, from fresh, made with butter	3000_Vegetables	49	41	8	1	4	C
75216200	Corn, white, cooked, NS as to form, NS as to fat added in cooking	3000_Vegetables	49	41	8	1	4	C
75216202	Corn, white, cooked, from frozen, NS as to fat added in cooking	3000_Vegetables	49	41	8	1	4	C

75216220	Corn, white, cooked, NS as to form, fat added in cooking, NS as to type of fat	3000_Vegetables	49	41	8	1	4	C
75216222	Corn, white, cooked, from frozen, fat added in cooking, NS as to type of fat	3000_Vegetables	49	41	8	1	4	C
75216228	Corn, white, cooked, from fresh, made with butter	3000_Vegetables	49	41	8	1	4	C
75500510	Celery, pickled	3000_Vegetables	48	53	-5	3	3	D
73406010	Sweet potato with fruit	3000_Vegetables	48	49	-1	3	3.5	B
73102240	Carrots, cooked, NS as to form, glazed	3000_Vegetables	48	45	3	2	4	B
71501011	Potato, mashed, from fresh, made with milk, with cheese	3000_Vegetables	48	43	5	2	3.5	C
71508030	Potato, baked, peel eaten, with chili	3000_Vegetables	48	43	5	2	4	B
75216120	Corn, NS as to form, cooked	3000_Vegetables	48	43	5	3	4	C
71501000	Potato, mashed, NFS	3000_Vegetables	48	42	6	1	4	C
71501010	Potato, mashed, from fresh, made with milk	3000_Vegetables	48	42	6	1	4	C
71501013	Potato, mashed, from fresh, NFS	3000_Vegetables	48	42	6	1	4	C
71508020	Potato, baked, peel eaten, with cheese	3000_Vegetables	48	42	6	2	4	C
71507050	White potato, stuffed, baked, peel not eaten, stuffed with meat in cream sauce	3000_Vegetables	48	41	7	2	4	C
71106020	Potato from Puerto Rican style stuffed pot roast, with gravy	3000_Vegetables	48	40	8	2	4	B
71106050	Potato from Puerto Rican beef stew, with gravy	3000_Vegetables	48	40	8	2	4	B
75216028	Corn, cooked, from fresh, NS as to color, made with butter	3000_Vegetables	48	40	8	1	4	C
75217022	Eggplant, cooked, made with butter	3000_Vegetables	48	40	8	2	4	B
75216025	Corn, cooked, NS as to form, NS as to color, made with butter	3000_Vegetables	48	39	9	1	4	C
75216132	Corn, yellow, cooked, NS as to form, made with butter	3000_Vegetables	48	39	9	1	4	C
71411000	Potato skins, with cheese and bacon	3000_Vegetables	47	44	3	2	3.5	D
75414500	Fried okra	3000_Vegetables	47	44	3	2	3	C
71507030	Potato, baked, peel not eaten, with chili	3000_Vegetables	47	42	5	2	4	C
71103040	Potato, boiled, from fresh, peel not eaten, made with butter	3000_Vegetables	47	41	6	1	4	C
71104140	Potato, roasted, from fresh, peel not eaten, made with butter	3000_Vegetables	47	41	6	1	4	C
71507005	Potato, baked, peel not eaten, with butter	3000_Vegetables	47	40	7	1	4	C
71508000	White potato, stuffed, baked, peel eaten, NS as to topping	3000_Vegetables	47	40	7	2	4	C
71508010	Potato, baked, peel eaten, with sour cream	3000_Vegetables	47	40	7	1	4	C
71501070	White potato, from dry, mashed, made with milk, fat, egg and cheese	3000_Vegetables	47	39	8	2	2	D
71508120	White potato, stuffed with ham, broccoli and cheese sauce, baked, peel eaten	3000_Vegetables	47	39	8	2	4	A
73102241	Carrots, glazed, cooked	3000_Vegetables	46	45	1	2	4	B
75409020	Fried cauliflower	3000_Vegetables	46	43	3	2	3.5	C
71305015	Potato, scalloped, NFS	3000_Vegetables	46	41	5	2	3	D
71503010	Potato patty	3000_Vegetables	46	41	5	3	3	C
71507020	Potato, baked, peel not eaten, with cheese	3000_Vegetables	46	41	5	2	4	C
71508025	Potato, baked, peel eaten, with meat	3000_Vegetables	46	40	6	2	4	C
71501060	White potato, from dry, mashed, made with milk, fat and egg	3000_Vegetables	46	37	9	2	3.5	D

74205020	Tomato, green, pickled	3000_Vegetables	45	45	0	2	2.5	C
74205010	Fried green tomatoes	3000_Vegetables	45	42	3	2	3.5	C
75418010	Fried summer squash, yellow or green	3000_Vegetables	45	42	3	2	3	C
71305030	Potato, scalloped, from fresh	3000_Vegetables	45	41	4	2	3	D
75415021	Onion rings, from fresh, batter-dipped, baked or fried	3000_Vegetables	45	41	4	2	3	C
71501045	Potato, mashed, from dry mix, made with milk, with cheese	3000_Vegetables	45	40	5	2	3	C
75216142	Corn, canned, cooked with butter or margarine	3000_Vegetables	45	40	5	3	4	C
71501012	Potato, mashed, from fresh, made with milk, with gravy	3000_Vegetables	45	39	6	2	4	C
71507010	Potato, baked, peel not eaten, with sour cream	3000_Vegetables	45	39	6	1	4	C
75216233	Corn, white, cooked, from frozen, made with butter	3000_Vegetables	45	35	10	1	4	C
71401033	Potato, french fries, school	3000_Vegetables	44	51	-7	4	4	C
71505020	Potato tots, school	3000_Vegetables	44	51	-7	4	4	C
71404050	Potato, hash brown, from school lunch	3000_Vegetables	44	49	-5	4	4.5	B
75216070	Corn, dried, cooked	3000_Vegetables	44	43	1	3	4	C
71305050	Potato, scalloped, from dry mix	3000_Vegetables	44	40	4	2	3	D
71305070	Potato, scalloped, ready-to-heat	3000_Vegetables	44	39	5	2	3	D
71501016	Potato, mashed, from restaurant	3000_Vegetables	44	39	5	2	3.5	C
71501300	White potato, from dry, mashed, NS as to milk or fat	3000_Vegetables	44	39	5	2	3	C
71507025	Potato, baked, peel not eaten, with meat	3000_Vegetables	44	39	5	2	4	C
75216003	Corn, cooked, from canned, NS as to color, NS as to fat added in cooking	3000_Vegetables	44	38	6	3	4	C
75216023	Corn, cooked, from canned, NS as to color, fat added in cooking, NS as to type of fat	3000_Vegetables	44	38	6	3	4	C
75216103	Corn, yellow, cooked, from canned, NS as to fat added in cooking	3000_Vegetables	44	38	6	3	4	C
71930100	Cassava, cooked, fat not added in cooking	3000_Vegetables	44	36	8	1	4	C
73102242	Carrots, cooked, from frozen, glazed	3000_Vegetables	43	42	1	2	3.5	C
71305020	Potato, scalloped, from fast food or restaurant	3000_Vegetables	43	38	5	2	2	D
71305040	Potato, scalloped, from fresh, with meat	3000_Vegetables	43	38	5	2	3	D
71501035	Potato, mashed, from dry mix, NFS	3000_Vegetables	43	38	5	2	3.5	C
75216163	Corn, yellow and white, cooked, from canned, NS as to fat added in cooking	3000_Vegetables	43	38	5	3	4	C
75216183	Corn, yellow and white, cooked, from canned, fat added in cooking, NS as to type of fat	3000_Vegetables	43	38	5	3	4	C
75216223	Corn, white, cooked, from canned, fat added in cooking, NS as to type of fat	3000_Vegetables	43	37	6	3	4	C
71507000	White potato, stuffed, baked, peel not eaten, NS as to topping	3000_Vegetables	43	36	7	2	3.5	C
71401041	Potato, french fries, with cheese, school	3000_Vegetables	42	49	-7	4	4	C
73410400	Sweet potato fries, fast food / restaurant	3000_Vegetables	42	47	-5	4	3	C
71501040	Potato, mashed, from dry mix, made with milk	3000_Vegetables	42	37	5	2	3.5	C
71301120	White potato, cooked, with ham and cheese	3000_Vegetables	42	35	7	2	3.5	C
71305110	White potato, scalloped, with ham	3000_Vegetables	42	34	8	2	3.5	C
71403050	Potato, home fries, ready-to-heat	3000_Vegetables	41	49	-8	4	4	C

71405030	Potato, hash brown, from dry mix	3000_Vegetables	41	46	-5	4	4	C
71405040	Potato, hash brown, ready-to-heat	3000_Vegetables	41	46	-5	4	4	C
75515010	Vegetable relish	3000_Vegetables	41	46	-5	3	3.5	B
71305080	Potato, scalloped, ready-to-heat, with meat	3000_Vegetables	41	36	5	2	2.5	D
71501017	Potato, mashed, from restaurant, with gravy	3000_Vegetables	41	36	5	2	3	C
71501025	White potato, from fresh, mashed, made with milk, and sour cream and/or cream cheese and fat	3000_Vegetables	41	36	5	2	3	C
71305060	Potato, scalloped, from dry mix, with meat	3000_Vegetables	41	35	6	2	3	D
71501055	White potato, from fresh, mashed, made with sour cream and/or cream cheese and fat	3000_Vegetables	41	35	6	2	3	C
71501015	White potato, from fresh, mashed, made with milk, and sour cream and/or cream cheese	3000_Vegetables	41	34	7	2	3.5	C
71404000	Potato, hash brown, NFS	3000_Vegetables	40	46	-6	4	4	C
71402500	Potato, french fries, with cheese	3000_Vegetables	40	42	-2	4	3	C
71501200	White potato, from complete dry mix, mashed, made with water	3000_Vegetables	40	42	-2	4	4	B
75534030	Turnip, pickled	3000_Vegetables	40	41	-1	3	2.5	D
75511300	Pickles, fried	3000_Vegetables	40	36	4	2	2.5	C
71501054	Potato, mashed, from dry mix, made with milk, with gravy	3000_Vegetables	40	34	6	2	3	C
75216035	Corn, cooked, from canned, NS as to color, made with butter	3000_Vegetables	40	33	7	3	4	C
71930090	Cassava, cooked, NS as to fat added in cooking	3000_Vegetables	40	32	8	1	4	C
71401032	Potato, french fries, from frozen, fried	3000_Vegetables	39	46	-7	4	4	C
71501018	Potato, mashed, from school lunch	3000_Vegetables	39	42	-3	4	4	B
75216188	Corn, yellow and white, cooked, from canned, made with butter	3000_Vegetables	39	33	6	3	4	C
71401030	Potato, french fries, fast food	3000_Vegetables	38	42	-4	4	4.5	C
71402510	Potato, french fries, with chili and cheese	3000_Vegetables	38	40	-2	4	3	C
71404040	Potato, hash brown, from restaurant, with cheese	3000_Vegetables	37	39	-2	4	3	D
71505000	Potato tots, NFS	3000_Vegetables	37	38	-1	4	3.5	C
75500210	Beets, pickled	3000_Vegetables	37	34	3	3	3.5	A
71405000	White potato, hash brown, NS as to from fresh, frozen, or dry mix	3000_Vegetables	36	41	-5	4	3.5	D
71405020	White potato, hash brown, from frozen	3000_Vegetables	36	41	-5	4	3.5	D
71405050	Potato, hash brown, ready-to-heat, with cheese	3000_Vegetables	36	41	-5	4	3.5	D
71404010	Potato, hash brown, from fast food	3000_Vegetables	36	40	-4	4	3.5	D
71404030	Potato, hash brown, from restaurant	3000_Vegetables	36	40	-4	4	3.5	D
71402520	Potato, french fries, with chili	3000_Vegetables	36	39	-3	4	3.5	C
73406000	Sweet potato, candied	3000_Vegetables	36	39	-3	2	2.5	C
71505030	Potato tots, from fresh, fried or baked	3000_Vegetables	36	38	-2	4	3.5	C
71930140	Cassava, cooked, made with butter	3000_Vegetables	36	27	9	1	4	C
71401020	Potato, french fries, from frozen, baked	3000_Vegetables	35	39	-4	4	4.5	C
71401035	White potato, french fries, from frozen, NS as to deep fried or oven baked	3000_Vegetables	35	39	-4	4	4.5	C
71401045	Potato, french fries, with chili, fast food / restaurant	3000_Vegetables	35	38	-3	4	3.5	C

71601010	Potato salad with egg, made with mayonnaise	3000_Vegetables	34	41	-7	4	3.5	C
71905210	Candied ripe plantain, Puerto Rican style	3000_Vegetables	34	36	-2	2	2	D
71401039	Potato, french fries, with cheese, fast food / restaurant	3000_Vegetables	33	37	-4	4	3	C
71404020	Potato, hash brown, from fast food, with cheese	3000_Vegetables	33	37	-4	4	3	D
71401050	Potato, french fries, with chili and cheese, fast food / restaurant	3000_Vegetables	33	36	-3	4	3	C
73102243	Carrots, cooked, from canned, glazed	3000_Vegetables	33	34	-1	2	3	C
71701500	Lefse (Norwegian)	3000_Vegetables	33	25	8	1	4	C
71501005	Potato, mashed, from fast food	3000_Vegetables	32	35	-3	4	4	C
71402505	White potato, french fries, with cheese and bacon	3000_Vegetables	32	34	-2	4	1.5	E
71403030	Potato, home fries, from restaurant / fast food	3000_Vegetables	31	34	-3	4	3.5	C
73304010	Squash fritter or cake	3000_Vegetables	31	23	8	2	3	D
74202050	Tomatoes, red, NS as to form, fried	3000_Vegetables	31	22	9	3	3	C
71501071	Potato, mashed, ready-to-heat, with cheese	3000_Vegetables	30	32	-2	4	3	C
71505050	Potato tots, frozen, fried	3000_Vegetables	29	33	-4	4	3.5	C
71505060	Potato tots, frozen, NS as to fried or baked	3000_Vegetables	29	33	-4	4	3.5	C
75216153	Corn, creamed	3000_Vegetables	29	28	1	4	4	C
71501006	Potato, mashed, from fast food, with gravy	3000_Vegetables	28	30	-2	4	3.5	C
75216193	Corn, yellow, from canned, cream style, fat added in cooking	3000_Vegetables	28	27	1	4	3.5	C
75216053	Corn, from canned, NS as to color, cream style	3000_Vegetables	27	27	0	4	3.5	C
75216190	Corn, yellow, NS as to form, cream style, fat added in cooking	3000_Vegetables	27	25	2	4	3.5	C
75411020	Corn fritter	3000_Vegetables	27	18	9	3	2.5	D
75512010	Radishes, pickled, Hawaiian style	3000_Vegetables	25	35	-10	4	3	C
71501007	Potato, mashed, ready-to-heat	3000_Vegetables	25	26	-1	4	3.5	C
71501061	Potato, mashed, ready-to-heat, NFS	3000_Vegetables	25	26	-1	4	3.5	C
71505040	Potato tots, frozen, baked	3000_Vegetables	25	26	-1	4	4	C
75216050	Corn, NS as to form, NS as to color, cream style	3000_Vegetables	25	24	1	4	3.5	C
75216150	Corn, yellow, NS as to form, cream style	3000_Vegetables	25	24	1	4	3.5	C
75216253	Corn, white, from canned, cream style	3000_Vegetables	24	23	1	4	3.5	C
71505010	Potato tots, fast food / restaurant	3000_Vegetables	23	25	-2	4	3.5	D
71501075	Potato, mashed, ready-to-heat, with gravy	3000_Vegetables	23	23	0	4	3.5	C
75216250	Corn, white, NS as to form, cream style	3000_Vegetables	23	21	2	4	3.5	C
75415022	Fried onion rings	3000_Vegetables	21	25	-4	4	2.5	D
71402040	White potato, french fries, breaded or battered	3000_Vegetables	21	21	0	4	3	D
75503100	Mustard pickles	3000_Vegetables	20	25	-5	3	2.5	C
75501010	Relish, corn	3000_Vegetables	20	21	-1	3	3	C
75415020	Onion rings, NS as to form, batter-dipped, baked or fried	3000_Vegetables	18	24	-6	4	3	C
91408100	Chinese preserved sweet vegetable	3000_Vegetables	15	20	-5	2	0.5	D
75503140	Cucumber pickles, sweet, reduced salt	3000_Vegetables	9	21	-12	4	3	C
75503040	Pickles, sweet	3000_Vegetables	1	10	-9	4	3	C

75503050	Cucumber pickles, fresh	3000_Vegetables	1	10	-9	4	3	C
75503020	Relish, pickle	3000_Vegetables	1	2	-1	4	2	D
75511200	Pickles, mixed	3000_Vegetables	1	2	-1	4	2	D
41107000	Soybeans, dry, cooked, fat not added in cooking	4000_LegNut	100	100	0	1	5	A
41420020	Edamame, cooked	4000_LegNut	100	100	0	1	5	A
41422010	Soybean meal	4000_LegNut	100	100	0	1	5	A
41440000	Textured vegetable protein, dry	4000_LegNut	100	100	0	1	5	A
42100100	Almonds, NFS	4000_LegNut	100	100	0	1	5	A
42101000	Almonds, unroasted	4000_LegNut	100	100	0	1	5	A
42101210	Almonds, dry roasted, without salt	4000_LegNut	100	100	0	1	5	A
42111040	Peanuts, unroasted	4000_LegNut	100	100	0	1	5	A
43101000	Pumpkin and/or squash seeds, hulled, unroasted	4000_LegNut	100	100	0	1	5	A
43102000	Sunflower seeds, plain, unsalted	4000_LegNut	100	100	0	1	5	A
43102100	Sunflower seeds, plain, salted	4000_LegNut	100	100	0	1	4.5	B
43102110	Sunflower seeds, hulled, roasted, without salt	4000_LegNut	100	100	0	3	5	A
43102400	Sunflower seeds, NFS	4000_LegNut	100	100	0	1	5	A
43103050	Sesame seeds, whole seed	4000_LegNut	100	100	0	1	5	A
43104000	Flax seeds	4000_LegNut	100	100	0	1	5	A
43107000	Mixed seeds	4000_LegNut	100	100	0	1	5	A
43108010	Chia seeds	4000_LegNut	100	100	0	1	5	A
41301020	Cowpeas, dry, cooked, fat not added in cooking	4000_LegNut	100	99	1	1	5	A
41103020	Lima beans, dry, cooked, fat not added in cooking	4000_LegNut	100	98	2	1	5	A
41305000	Lentils, from dried, no added fat	4000_LegNut	100	98	2	1	5	A
42110050	Mixed nuts, unroasted	4000_LegNut	99	100	-1	1	5	A
43101050	Pumpkin seeds, NFS	4000_LegNut	99	100	-1	1	5	A
43101150	Pumpkin seeds, unsalted	4000_LegNut	99	100	-1	1	5	A
41304991	Lentils, dry, cooked, made with oil	4000_LegNut	98	100	-2	1	5	A
42110500	Mixed nuts, in shell	4000_LegNut	98	100	-2	1	4.5	A
43102300	Sunflower seeds, flavored	4000_LegNut	98	100	-2	1	4.5	C
42114130	Pistachio nuts, NFS	4000_LegNut	98	99	-1	1	5	A
41102020	Black beans, from dried, no added fat	4000_LegNut	98	95	3	1	5	A
42202200	Peanut butter, vitamin and mineral fortified	4000_LegNut	97	100	-3	3	3.5	C
42107000	Hazelnuts	4000_LegNut	97	99	-2	1	5	A
41304980	Lentils, dry, cooked, NS as to fat added in cooking	4000_LegNut	97	98	-1	1	5	A
41108000	Mung beans, dry, cooked, fat not added in cooking	4000_LegNut	97	91	6	1	5	A
42114145	Pistachio nuts, unsalted	4000_LegNut	96	98	-2	1	5	A
41106020	Kidney beans, from dried, no added fat	4000_LegNut	96	94	2	1	5	A
43102005	Sunflower seeds, hulled, unroasted, salted	4000_LegNut	95	100	-5	3	4.5	B
42113000	Pine nuts	4000_LegNut	95	98	-3	1	4	B

43103200	Sesame paste (sesame butter made from whole seeds)	4000_LegNut	95	97	-2	1	4.5	A
43103300	Tahini	4000_LegNut	95	97	-2	1	4.5	A
42110020	Mixed nuts, without salt	4000_LegNut	95	96	-1	1	4.5	A
41304993	Lentils, dry, cooked, made with margarine	4000_LegNut	95	95	0	2	5	A
41109000	Mungo beans, cooked, fat not added in cooking	4000_LegNut	95	94	1	1	5	A
41102120	Black, brown, or Bayo beans, canned, drained, low sodium, fat not added in cooking	4000_LegNut	95	93	2	3	5	A
41104020	Pinto beans, from dried, no added fat	4000_LegNut	95	92	3	1	5	A
42101130	Almonds, unsalted	4000_LegNut	94	99	-5	3	5	A
43102200	Sunflower seeds, hulled, dry roasted	4000_LegNut	94	98	-4	3	4.5	C
42114140	Pistachio nuts, salted	4000_LegNut	94	96	-2	1	5	C
43103000	Sesame seeds	4000_LegNut	94	96	-2	1	5	A
41304990	Lentils, from dried, fat added	4000_LegNut	94	95	-1	1	5	A
41106520	Yellow, canary, or Peruvian beans, dry, cooked, fat not added in cooking	4000_LegNut	94	90	4	1	5	A
41202505	Beans and tomatoes, no added fat	4000_LegNut	94	90	4	1	5	A
42110160	Mixed nuts, without peanuts, unsalted	4000_LegNut	93	100	-7	3	4.5	A
42101120	Almonds, lightly salted	4000_LegNut	93	98	-5	3	5	A
41106000	Red kidney beans, dry, cooked, NS as to fat added in cooking	4000_LegNut	93	95	-2	1	5	A
41301000	Cowpeas, dry, cooked, NS as to fat added in cooking	4000_LegNut	93	95	-2	1	5	A
41301010	Blackeyed peas, from dried	4000_LegNut	93	95	-2	1	5	A
42500100	Trail mix with nuts	4000_LegNut	93	95	-2	1	4.5	B
41102000	Black, brown, or Bayo beans, dry, cooked, NS as to fat added in cooking	4000_LegNut	93	94	-1	1	5	A
41102010	Black beans, from dried, fat added	4000_LegNut	93	94	-1	1	5	A
41305020	Lentils, from canned	4000_LegNut	93	94	-1	1	5	A
42102000	Brazil nuts	4000_LegNut	93	94	-1	1	4	B
41101020	Beans, from dried, NS as to type, no added fat	4000_LegNut	93	89	4	1	5	A
41303000	Split peas, from dried, no added fat	4000_LegNut	93	87	6	1	5	A
42116000	Walnuts, excluding honey roasted	4000_LegNut	92	99	-7	1	4.5	A
42116050	Walnuts	4000_LegNut	92	98	-6	1	4.5	A
42116055	Walnuts, roasted, without salt	4000_LegNut	92	98	-6	1	4.5	A
41103000	Lima beans, dry, cooked, NS as to fat added in cooking	4000_LegNut	92	93	-1	1	5	A
41103011	Lima beans, dry, cooked, made with oil	4000_LegNut	92	93	-1	1	5	A
42105000	Chestnuts	4000_LegNut	92	93	-1	1	4.5	B
42111210	Peanuts, dry roasted, unsalted	4000_LegNut	92	93	-1	1	5	A
41101120	White beans, from dried, no added fat	4000_LegNut	92	89	3	1	5	A
41102220	Fava beans, dry, cooked, fat not added in cooking	4000_LegNut	92	88	4	1	5	A
42110120	Mixed nuts, with peanuts, unsalted	4000_LegNut	91	97	-6	3	5	A
41107010	Soybeans, cooked	4000_LegNut	91	95	-4	1	5	A
41107020	Soybeans, dry, cooked, NS as to fat added in cooking	4000_LegNut	91	95	-4	1	5	A

42112000	Pecans, NFS	4000_LegNut	91	94	-3	1	4.5	A
41103010	Lima beans, from dried	4000_LegNut	91	93	-2	1	5	A
41106011	Red kidney beans, dry, cooked, made with oil	4000_LegNut	91	93	-2	1	5	A
41101000	Beans, dry, cooked, NS as to type and as to fat added in cooking	4000_LegNut	91	92	-1	1	5	A
41102011	Black, brown, or Bayo beans, dry, cooked, made with oil	4000_LegNut	91	92	-1	1	5	A
41102110	Black beans, from canned, reduced sodium	4000_LegNut	91	92	-1	3	5	A
41104000	Pinto, calico, or red Mexican beans, dry, cooked, NS as to fat added in cooking	4000_LegNut	91	92	-1	1	5	A
41104010	Pinto beans, from dried, fat added	4000_LegNut	91	92	-1	1	5	A
41104011	Pinto, calico, or red Mexican beans, dry, cooked, made with oil	4000_LegNut	91	92	-1	1	5	A
41106010	Kidney beans, from dried, fat added	4000_LegNut	91	92	-1	1	5	A
41202500	Beans and tomatoes, NS as to fat added in cooking	4000_LegNut	91	92	-1	1	5	A
43101100	Pumpkin seeds, salted	4000_LegNut	91	92	-1	1	4.5	C
41302020	Chickpeas, from dried, no added fat	4000_LegNut	91	88	3	1	5	A
42112100	Pecans, unroasted	4000_LegNut	90	93	-3	1	4.5	A
41102100	Black, brown, or Bayo beans, canned, drained, low sodium, NS as to fat added in cooking	4000_LegNut	90	92	-2	3	5	A
42100050	Nuts, NFS	4000_LegNut	90	92	-2	1	4.5	B
42110000	Mixed nuts, NFS	4000_LegNut	90	92	-2	1	4.5	B
42110100	Mixed nuts, with peanuts, salted	4000_LegNut	90	92	-2	1	4.5	B
41103013	Lima beans, dry, cooked, made with margarine	4000_LegNut	90	90	0	2	5	A
41108020	Mung beans, dry, cooked, NS as to fat added in cooking	4000_LegNut	90	90	0	1	5	A
41103060	Pink beans, dry, cooked, fat not added in cooking	4000_LegNut	90	89	1	1	5	A
41310310	Stewed blackeye peas or cowpeas, Puerto Rican style	4000_LegNut	90	89	1	1	5	A
41106120	Red kidney beans, canned, drained, low sodium, fat not added in cooking	4000_LegNut	90	88	2	3	5	A
42200510	Almond butter, lower sodium	4000_LegNut	89	94	-5	3	4.5	A
42111110	Peanuts, roasted, unsalted	4000_LegNut	89	92	-3	3	5	A
42104000	Cashews, NFS	4000_LegNut	89	91	-2	1	4	B
42104205	Cashew nuts, dry roasted, without salt	4000_LegNut	89	91	-2	1	4	B
41101110	White beans, from dried, fat added	4000_LegNut	89	90	-1	1	5	A
41106013	Red kidney beans, dry, cooked, made with margarine	4000_LegNut	89	90	-1	2	5	A
41106500	Yellow, canary, or Peruvian beans, dry, cooked, NS as to fat added in cooking	4000_LegNut	89	90	-1	1	5	A
41202510	Beans and tomatoes, fat added	4000_LegNut	89	90	-1	1	4.5	A
41106511	Yellow, canary, or Peruvian beans, dry, cooked, made with oil	4000_LegNut	89	89	0	1	5	A
41108010	Mung beans, cooked	4000_LegNut	89	89	0	1	5	A
41303011	Green or yellow split peas, dry, cooked, made with oil	4000_LegNut	89	89	0	1	5	A
41102013	Black, brown, or Bayo beans, dry, cooked, made with margarine	4000_LegNut	89	88	1	2	5	A
41104013	Pinto, calico, or red Mexican beans, dry, cooked, made with margarine	4000_LegNut	89	88	1	2	5	A

41303020	Green or yellow split peas, dry, cooked, NS as to fat added in cooking	4000_LegNut	89	88	1	1	5	A
41102080	Black beans, from canned, no added fat	4000_LegNut	89	86	3	3	5	A
41303500	Stewed green peas, Puerto Rican style	4000_LegNut	89	85	4	1	5	A
42101200	Almonds, dry roasted, salted	4000_LegNut	88	91	-3	3	5	C
42104050	Cashews, unroasted	4000_LegNut	88	91	-3	1	4	B
42104110	Cashews, unsalted	4000_LegNut	88	91	-3	1	4	B
41101100	White beans, dry, cooked, NS as to fat added in cooking	4000_LegNut	88	90	-2	1	5	A
41101111	White beans, dry, cooked, made with oil	4000_LegNut	88	90	-2	1	5	A
41103050	Pink beans, dry, cooked, NS as to fat added in cooking	4000_LegNut	88	90	-2	1	5	A
41103070	Pink beans, cooked	4000_LegNut	88	89	-1	1	5	A
41106510	Peruvian beans, from dried	4000_LegNut	88	87	1	1	5	A
41303010	Split peas, from dried, fat added	4000_LegNut	88	87	1	1	5	A
41303013	Green or yellow split peas, dry, cooked, made with margarine	4000_LegNut	88	86	2	2	5	A
42200500	Almond butter	4000_LegNut	87	92	-5	3	4.5	A
41106100	Red kidney beans, canned, drained, low sodium, NS as to fat added in cooking	4000_LegNut	87	90	-3	3	5	A
42101100	Almonds, roasted	4000_LegNut	87	90	-3	3	5	A
42101110	Almonds, salted	4000_LegNut	87	90	-3	3	5	A
41106110	Kidney beans, from canned, reduced sodium	4000_LegNut	87	89	-2	3	5	A
41102030	Black, brown, or Bayo beans, canned, drained, NS as to fat added in cooking	4000_LegNut	87	88	-1	3	5	A
41102040	Black beans, from canned, fat added	4000_LegNut	87	88	-1	3	5	A
41102050	Black, brown, or Bayo beans, canned, drained, made with oil	4000_LegNut	87	88	-1	3	5	A
42202010	Peanut butter, lower sodium	4000_LegNut	87	88	-1	1	3.5	C
41101113	White beans, dry, cooked, made with margarine	4000_LegNut	87	87	0	2	5	A
41108030	Mung beans, canned, drained, NS as to fat added in cooking	4000_LegNut	87	87	0	1	5	A
41304970	Lentils, NFS	4000_LegNut	87	87	0	1	5	A
41106540	Yellow, canary, or Peruvian beans, canned, drained, fat added in cooking, NS as to type of fat	4000_LegNut	87	86	1	1	5	A
41420010	Soybean curd	4000_LegNut	86	94	-8	3	4	A
42110150	Mixed nuts, without peanuts, salted	4000_LegNut	86	92	-6	3	4	C
42110110	Mixed nuts, with peanuts, lightly salted	4000_LegNut	86	91	-5	3	4.5	A
42114142	Pistachio nuts, lightly salted	4000_LegNut	86	90	-4	3	5	A
42203200	Soy nut butter	4000_LegNut	86	89	-3	3	3	C
41106150	Kidney beans, from fast food / restaurant	4000_LegNut	86	88	-2	1	5	A
41102150	Black beans, from fast food / restaurant	4000_LegNut	86	87	-1	1	5	A
41102210	Fava beans, cooked	4000_LegNut	86	87	-1	1	5	A
41104120	Pinto, calico, or red Mexican beans, canned, drained, low sodium, fat not added in cooking	4000_LegNut	86	86	0	3	5	A
41104200	Pinto beans, from fast food / restaurant	4000_LegNut	86	86	0	1	5	A
41300990	Blackeyed peas, NFS	4000_LegNut	86	86	0	1	5	A

41106513	Yellow, canary, or Peruvian beans, dry, cooked, made with margarine	4000_LegNut	86	85	1	2	5	A
41302120	Chickpeas, canned, drained, low sodium, fat not added in cooking	4000_LegNut	86	85	1	3	5	A
41103100	Pink beans, canned, drained, fat not added in cooking	4000_LegNut	86	83	3	1	5	A
42112210	Pecans, unsalted	4000_LegNut	85	90	-5	3	4.5	A
41410010	Soy nuts	4000_LegNut	85	89	-4	3	5	C
41102200	Fava beans, dry, cooked, NS as to fat added in cooking	4000_LegNut	85	87	-2	1	5	A
41103090	Pink beans, canned, drained, fat added in cooking	4000_LegNut	85	86	-1	1	5	A
41101990	Black beans, NFS	4000_LegNut	85	85	0	1	5	A
41102990	Lima beans, NFS	4000_LegNut	85	85	0	1	5	A
41302000	Chickpeas, dry, cooked, NS as to fat added in cooking	4000_LegNut	85	85	0	1	5	A
41302010	Chickpeas, from dried, fat added	4000_LegNut	85	85	0	1	5	A
41302011	Chickpeas, dry, cooked, made with oil	4000_LegNut	85	85	0	1	5	A
41103990	Pinto beans, NFS	4000_LegNut	85	84	1	1	5	A
42110015	Mixed nuts, salted	4000_LegNut	84	89	-5	3	4	C
41104100	Pinto, calico, or red Mexican beans, canned, drained, low sodium, NS as to fat added in cooking	4000_LegNut	84	87	-3	3	5	A
41104110	Pinto beans, from canned, reduced sodium	4000_LegNut	84	87	-3	3	5	A
42110200	Mixed nuts, dry roasted	4000_LegNut	84	87	-3	3	4.5	C
41102260	Fava beans, canned, drained, fat added in cooking	4000_LegNut	84	85	-1	1	5	A
41101090	White beans, NFS	4000_LegNut	84	84	0	1	5	A
59003000	Meat substitute, cereal- and vegetable protein-based, fried	4000_LegNut	84	83	1	1	4.5	A
41101070	Beans, from canned, NS as to type, no added fat	4000_LegNut	84	82	2	3	5	A
41106080	Kidney beans, from canned, no added fat	4000_LegNut	84	82	2	3	5	A
41221010	Baked beans, low sodium	4000_LegNut	83	86	-3	3	4.5	A
41302100	Chickpeas, canned, drained, low sodium, NS as to fat added in cooking	4000_LegNut	83	85	-2	3	5	A
41302110	Chickpeas, from canned, reduced sodium	4000_LegNut	83	85	-2	3	5	A
41101080	Beans, from fast food / restaurant, NS as to type	4000_LegNut	83	84	-1	1	5	A
41105990	Kidney beans, NFS	4000_LegNut	83	83	0	1	5	A
41100990	Beans, NFS	4000_LegNut	83	82	1	1	5	A
41101010	Beans, from dried, NS as to type, fat added	4000_LegNut	83	82	1	1	5	A
41221000	Baked beans, reduced sodium	4000_LegNut	83	82	1	3	5	A
41304992	Lentils, dry, cooked, made with animal fat or meat drippings	4000_LegNut	83	78	5	1	5	A
41101050	Beans, canned, drained, NS as to type and as to fat added in cooking	4000_LegNut	82	84	-2	3	5	A
41104030	Pinto, calico, or red Mexican beans, canned, drained, NS as to fat added in cooking	4000_LegNut	82	84	-2	3	5	A
41104050	Pinto, calico, or red Mexican beans, canned, drained, made with oil	4000_LegNut	82	84	-2	3	5	A
41106050	Red kidney beans, canned, drained, made with oil	4000_LegNut	82	84	-2	3	5	A
42111010	Peanuts, in shell, NFS (shell not eaten)	4000_LegNut	82	84	-2	3	4.5	C
42111100	Peanuts, roasted, salted	4000_LegNut	82	84	-2	3	4.5	C

42111205	Peanuts, dry roasted, lightly salted	4000_LegNut	82	84	-2	3	5	B
41106040	Kidney beans, from canned, fat added	4000_LegNut	82	83	-1	3	5	A
42202100	Peanut butter, lower sodium and lower sugar	4000_LegNut	82	83	-1	2	4	C
41104080	Pinto beans, from canned, no added fat	4000_LegNut	82	81	1	3	5	A
41302013	Chickpeas, dry, cooked, made with margarine	4000_LegNut	82	81	1	2	5	A
41302080	Chickpeas, from canned, no added fat	4000_LegNut	82	81	1	3	5	A
41104040	Pinto beans, from canned, fat added	4000_LegNut	81	84	-3	3	5	A
42502000	Nut mixture with seeds	4000_LegNut	81	82	-1	3	5	A
41210200	Black beans, Cuban style	4000_LegNut	81	80	1	1	4.5	A
41205015	Refried beans, fat not added in cooking	4000_LegNut	81	79	2	3	5	A
41102070	Black, brown, or Bayo beans, canned, drained, made with margarine	4000_LegNut	80	82	-2	3	5	A
41302030	Chickpeas, canned, drained, NS as to fat added in cooking	4000_LegNut	80	82	-2	3	5	A
41302050	Chickpeas, canned, drained, made with oil	4000_LegNut	80	82	-2	3	5	A
42104100	Cashews, salted	4000_LegNut	80	82	-2	1	3.5	C
41101200	White beans, canned, drained, low sodium, NS as to fat added in cooking	4000_LegNut	80	81	-1	3	5	A
41302040	Chickpeas, from canned, fat added	4000_LegNut	80	81	-1	3	5	A
41311000	Papad, grilled or broiled	4000_LegNut	80	81	-1	3	4.5	E
42500000	Trail mix, NFS	4000_LegNut	80	80	0	1	4	C
42501000	Trail mix with nuts and fruit	4000_LegNut	80	80	0	1	4	C
41104250	Pinto beans with meat	4000_LegNut	80	78	2	1	5	A
41301990	Chickpeas, NFS	4000_LegNut	80	78	2	1	5	A
41101210	White beans, from canned, reduced sodium	4000_LegNut	79	81	-2	3	5	A
41101150	White beans, canned, drained, made with oil	4000_LegNut	79	80	-1	3	5	A
41205040	Refried beans, from canned, reduced sodium	4000_LegNut	79	80	-1	3	5	A
42111000	Peanuts, NFS	4000_LegNut	79	80	-1	3	4.5	C
42111200	Peanuts, dry roasted, salted	4000_LegNut	79	80	-1	3	4.5	C
41101220	White beans, canned, drained, low sodium, fat not added in cooking	4000_LegNut	79	77	2	3	5	A
41102170	Black beans with meat	4000_LegNut	79	77	2	1	5	A
41106170	Kidney beans with meat	4000_LegNut	79	77	2	1	5	A
41421010	Soybean curd, deep fried	4000_LegNut	78	85	-7	3	4.5	A
41203020	Kidney bean salad	4000_LegNut	78	81	-3	2	5	A
41101140	White beans, from canned, fat added	4000_LegNut	78	80	-2	3	5	A
41205011	Refried beans, made with oil	4000_LegNut	78	78	0	2	5	A
41205016	Refried beans, NS as to fat added in cooking	4000_LegNut	78	78	0	2	5	A
42111030	Peanuts, boiled	4000_LegNut	78	78	0	3	4.5	C
42104105	Cashews, lightly salted	4000_LegNut	77	81	-4	3	3.5	C
42202130	Peanut butter, lower sugar	4000_LegNut	77	80	-3	3	4	C
41101060	Beans, from canned, NS as to type, fat added	4000_LegNut	77	77	0	3	5	A

41205013	Refried beans, made with margarine	4000_LegNut	77	76	1	2	5	A
41101180	White beans, from canned, no added fat	4000_LegNut	77	75	2	3	5	A
41207030	Beans, dry, cooked with ground beef	4000_LegNut	77	75	2	1	4.5	A
41811200	Fish stick, meatless	4000_LegNut	76	89	-13	4	4	C
41210110	Stewed dry lima beans, Puerto Rican style	4000_LegNut	76	72	4	2	4	B
41102012	Black, brown, or Bayo beans, dry, cooked, made with animal fat or meat drippings	4000_LegNut	76	71	5	1	5	A
41103012	Lima beans, dry, cooked, made with animal fat or meat drippings	4000_LegNut	76	71	5	1	5	A
41104012	Pinto, calico, or red Mexican beans, dry, cooked, made with animal fat or meat drippings	4000_LegNut	76	71	5	1	5	A
41812000	Sandwich spread, meat substitute type	4000_LegNut	75	89	-14	4	3.5	C
41104070	Pinto, calico, or red Mexican beans, canned, drained, made with margarine	4000_LegNut	75	78	-3	3	5	A
42202150	Peanut butter, reduced fat	4000_LegNut	75	78	-3	3	4	C
41210150	Stewed pink beans with white potatoes and ham, Puerto Rican style	4000_LegNut	75	72	3	1	4	B
41106012	Red kidney beans, dry, cooked, made with animal fat or meat drippings	4000_LegNut	75	71	4	1	5	A
41303012	Green or yellow split peas, dry, cooked, made with animal fat or meat drippings	4000_LegNut	75	68	7	1	5	A
42104200	Cashew nuts, dry roasted, salted	4000_LegNut	74	78	-4	3	3	D
42101350	Almonds, honey roasted	4000_LegNut	74	76	-2	3	2.5	C
42110300	Mixed nuts, honey roasted	4000_LegNut	74	76	-2	3	2.5	C
41205017	Refried beans, from fast food / restaurant	4000_LegNut	74	72	2	1	4.5	A
41101112	White beans, dry, cooked, made with animal fat or meat drippings	4000_LegNut	74	70	4	1	5	A
41208100	Beans with meat, NS as to type	4000_LegNut	74	70	4	1	5	A
41106512	Yellow, canary, or Peruvian beans, dry, cooked, made with animal fat or meat drippings	4000_LegNut	74	69	5	1	5	A
41205010	Refried beans	4000_LegNut	73	72	1	3	4.5	A
42111500	Peanuts, honey roasted	4000_LegNut	73	72	1	3	4	C
41210100	Stewed red beans, Puerto Rican style	4000_LegNut	73	70	3	2	4	B
42200600	Almond paste	4000_LegNut	72	78	-6	3	2.5	C
42201000	Cashew butter	4000_LegNut	72	76	-4	3	2.5	C
42202000	Peanut butter	4000_LegNut	72	75	-3	3	3	D
42502100	Trail mix with pretzels, cereal, or granola	4000_LegNut	72	73	-1	2	4	C
41102060	Black, brown, or Bayo beans, canned, drained, made with animal fat or meat drippings	4000_LegNut	72	68	4	3	5	A
41812600	Vegetarian, fillet	4000_LegNut	71	84	-13	4	4	C
42101300	Almonds, flavored	4000_LegNut	71	79	-8	4	5	C
42112200	Pecans, salted	4000_LegNut	71	73	-2	3	4	B
41310160	Stewed chickpeas, with potatoes, Puerto Rican style	4000_LegNut	71	66	5	1	5	B
41302012	Chickpeas, dry, cooked, made with animal fat or meat drippings	4000_LegNut	71	65	6	1	5	A
42501500	Trail mix with chocolate	4000_LegNut	70	72	-2	2	3.5	D
41205020	Refried beans with cheese	4000_LegNut	70	69	1	3	4.5	A

41812900	Vegetarian meat loaf	4000_LegNut	68	78	-10	4	4	A
42109105	Macadamia nuts, roasted, without salt	4000_LegNut	68	68	0	3	3.5	C
41104060	Pinto, calico, or red Mexican beans, canned, drained, made with animal fat or meat drippings	4000_LegNut	68	65	3	3	5	A
41205012	Refried beans, made with animal fat or meat drippings	4000_LegNut	68	64	4	2	4.5	A
41310150	Stewed chickpeas, Puerto Rican style	4000_LegNut	68	63	5	1	3.5	A
42116100	Walnuts, honey roasted	4000_LegNut	67	71	-4	3	2	C
42109000	Macadamia nuts, unroasted	4000_LegNut	67	68	-1	3	3.5	C
42109100	Macadamia nuts	4000_LegNut	67	68	-1	3	3.5	C
41306000	Loaf, lentil	4000_LegNut	67	67	0	1	3.5	A
41310100	Stewed pigeon peas, Puerto Rican style (Gandules guisados, Gandur, Gandules)	4000_LegNut	67	62	5	1	4	C
42112300	Pecans, honey roasted	4000_LegNut	66	67	-1	3	2.5	C
41304130	Cowpeas, dry, cooked with pork	4000_LegNut	66	64	2	2	4.5	A
42104500	Cashews, honey roasted	4000_LegNut	65	67	-2	3	1.5	D
41304030	Peas, dry, cooked with pork	4000_LegNut	65	61	4	2	4.5	A
41811900	Soyburger, meatless, no bun	4000_LegNut	64	73	-9	4	4	A
41811910	Vegetable burger or patty, meatless, no bun	4000_LegNut	64	73	-9	4	4	A
41201020	Baked beans, vegetarian	4000_LegNut	63	63	0	3	4	A
41310220	Fried chickpeas with bacon, Puerto Rican style	4000_LegNut	63	60	3	2	4.5	C
41205030	Refried beans with meat	4000_LegNut	62	58	4	3	4.5	A
41310200	Chickpeas stewed with pig's feet, Puerto Rican style	4000_LegNut	62	56	6	1	4	A
41810610	Chicken, meatless, breaded, fried	4000_LegNut	61	70	-9	4	4.5	A
41811400	Frankfurter or hot dog, meatless	4000_LegNut	60	69	-9	4	4	A
41810400	Breakfast link, pattie, or slice, meatless	4000_LegNut	59	71	-12	4	2	D
41201050	Baked beans from fast food / restaurant	4000_LegNut	59	62	-3	4	4	C
41310210	Stewed chickpeas with Spanish sausages, Puerto Rican style	4000_LegNut	59	56	3	2	3.5	B
41210180	Stewed white beans with pig's feet, Puerto Rican style	4000_LegNut	59	52	7	1	4	B
41811890	Vegetarian burger or patty, meatless, no bun	4000_LegNut	58	63	-5	4	4	B
41210160	Stewed pink beans with pig's feet, Puerto Rican style	4000_LegNut	58	51	7	1	4	B
41210170	Stewed red beans with pig's feet, Puerto Rican style	4000_LegNut	58	51	7	1	4	B
41303550	Stewed green peas with pig's feet and potatoes, Puerto Rican style	4000_LegNut	58	50	8	1	4	A
41420050	Soybean curd cheese	4000_LegNut	57	68	-11	4	4	A
41811800	Meatball, meatless	4000_LegNut	56	65	-9	4	4	A
42203000	Peanut butter and jelly	4000_LegNut	56	58	-2	3	2	D
41210190	Stewed red beans with pig's feet and potatoes, Puerto Rican style	4000_LegNut	56	49	7	1	4	A
41811600	Luncheon slice, meatless-beef, chicken, salami or turkey	4000_LegNut	55	65	-10	4	3.5	D
41210090	Stewed beans with pork, tomatoes, and chili peppers, Mexican style	4000_LegNut	54	48	6	1	3.5	C
42106000	Coconut, fresh	4000_LegNut	53	47	6	1	2	C
41210120	Stewed white beans, Puerto Rican style	4000_LegNut	53	46	7	1	3	C

41810600	Chicken, meatless, NFS	4000_LegNut	49	55	-6	4	4	C
41810200	Bacon strip, meatless	4000_LegNut	47	57	-10	4	1	E
42203100	Peanut butter and chocolate spread	4000_LegNut	46	49	-3	3	1	D
41420380	Yogurt, soy	4000_LegNut	39	46	-7	4	3.5	A
41425010	Vermicelli, made from soybeans	4000_LegNut	39	42	-3	4	3.5	B
41421020	Soybean curd, breaded, fried	4000_LegNut	38	40	-2	3	3.5	B
41901020	Soyburger, meatless, with cheese on bun	4000_LegNut	36	39	-3	4	3.5	D
41304000	Wasabi peas	4000_LegNut	34	37	-3	4	4	D
41204020	Boston baked beans	4000_LegNut	33	37	-4	4	4	A
41208030	Pork and beans	4000_LegNut	33	37	-4	4	4	A
41201010	Baked beans	4000_LegNut	33	36	-3	4	4	B
41202020	Chili beans, barbecue beans, ranch style beans or Mexican- style beans	4000_LegNut	32	35	-3	4	4	A
43105200	Psyllium seed, husks	4000_LegNut	32	24	8	4	5	B
42106020	Coconut, packaged	4000_LegNut	26	22	4	3	0.5	E
41206030	Beans and franks	4000_LegNut	17	15	2	4	3.5	C
41480020	Frozen dessert, non-dairy	4000_LegNut	1	6	-5	4	2	D
25110420	Chicken liver, braised	5000_MPE	81	74	7	1	4	C
32105020	Egg omelet or scrambled egg, with fish	5000_MPE	77	65	12	2	3.5	C
32105130	Egg omelet or scrambled egg, Spanish omelet, made with onions, peppers, tomatoes, and mushrooms	5000_MPE	76	68	8	2	4	B
25120000	Heart, cooked	5000_MPE	75	66	9	1	4	C
25110120	Beef liver, braised	5000_MPE	74	66	8	1	4	C
32130440	Egg omelet or scrambled egg, with dark-green vegetables, no added fat	5000_MPE	74	63	11	1	3.5	B
32130470	Egg omelet or scrambled egg, with tomatoes and dark-green vegetables, no fat added	5000_MPE	74	62	12	1	3.5	B
23333100	Squirrel, cooked	5000_MPE	74	58	16	1	4.5	C
24402100	Dove, cooked, NS as to cooking method	5000_MPE	73	66	7	1	4	A
25130000	Kidney, cooked	5000_MPE	73	64	9	1	4	C
32105040	Egg omelet or scrambled egg, with dark-green vegetables	5000_MPE	73	64	9	2	3.5	C
31101010	Egg, whole, raw	5000_MPE	73	61	12	1	4	A
31103010	Egg, whole, boiled or poached	5000_MPE	72	60	12	1	4	A
32130500	Egg omelet or scrambled egg, with vegetables other than dark green and/or tomatoes, no added fat	5000_MPE	72	59	13	1	3.5	B
33401220	Egg substitute, omelet, scrambled, or fried, with vegetables, NS as to fat added in cooking	5000_MPE	71	75	-4	3	3.5	C
25110450	Chicken liver, fried	5000_MPE	71	63	8	1	3.5	C
31110010	Egg, yolk only, raw	5000_MPE	71	59	12	1	2	D
32105013	Egg omelet or scrambled egg, with seafood	5000_MPE	71	57	14	2	3.5	C
32130430	Egg omelet or scrambled egg, with dark-green vegetables, fat added	5000_MPE	70	61	9	1	3.5	C
32130450	Egg omelet or scrambled egg, with dark-green vegetables, NS as to fat	5000_MPE	70	61	9	1	3.5	C

25110140	Beef liver, fried	5000_MPE	70	60	10	1	4	C
25150000	Brains, cooked	5000_MPE	70	54	16	1	3.5	C
32400320	Egg white, omelet, scrambled, or fried, with vegetables, NS as to fat added in cooking	5000_MPE	69	71	-2	2	3.5	B
24198720	Chicken, ground	5000_MPE	69	61	8	1	4	B
24207000	Turkey, ground	5000_MPE	69	61	8	1	4	B
31102000	Egg, whole, cooked, NS as to cooking method	5000_MPE	69	59	10	1	3.5	C
32130640	Egg omelet or scrambled egg, with cheese and dark-green vegetables, no added fat	5000_MPE	69	59	10	2	3.5	C
32130410	Egg omelet or scrambled egg, with tomatoes, no added fat	5000_MPE	69	56	13	1	3.5	B
32131210	Egg omelet or scrambled egg, with potatoes and/or onions, no added fat	5000_MPE	69	56	13	1	3.5	B
24403100	Quail, cooked	5000_MPE	68	63	5	1	4	A
24123301	Chicken breast, grilled without sauce, skin not eaten	5000_MPE	68	61	7	1	4	A
24124201	Chicken breast, sauteed, skin not eaten	5000_MPE	68	61	7	1	4	A
24127501	Chicken breast, baked, coated, skin / coating not eaten	5000_MPE	68	61	7	1	4	A
24122161	Chicken breast, baked, broiled, or roasted with marinade, skin not eaten, from raw	5000_MPE	68	60	8	1	4	A
24202500	Turkey, thigh, cooked, skin not eaten	5000_MPE	68	59	9	1	4	B
32105048	Egg omelet or scrambled egg, with mushrooms	5000_MPE	68	59	9	1	3.5	B
32130010	Egg omelet or scrambled egg, made with oil	5000_MPE	68	59	9	1	3.5	C
32130080	Egg omelet or scrambled egg, from fast food / restaurant	5000_MPE	68	59	9	1	3.5	C
24201220	Turkey, dark meat, roasted, skin not eaten	5000_MPE	68	58	10	1	4	B
32105170	Egg omelet or scrambled egg, with chicken or turkey	5000_MPE	68	58	10	1	4	A
32130460	Egg omelet or scrambled egg, with tomatoes and dark-green vegetables, fat added	5000_MPE	68	58	10	1	3.5	C
32130480	Egg omelet or scrambled egg, with tomatoes and dark-green vegetables, NS as to fat	5000_MPE	68	58	10	1	3.5	C
32130060	Egg omelet or scrambled egg, made with cooking spray	5000_MPE	68	56	12	1	3.5	B
31106010	Egg, whole, baked, no added fat	5000_MPE	68	54	14	1	3.5	B
32130070	Egg omelet or scrambled egg, no added fat	5000_MPE	68	54	14	1	3.5	B
33401210	Egg substitute, omelet, scrambled, or fried, with vegetables, fat not added in cooking	5000_MPE	67	66	1	3	4	A
23332100	Opossum, cooked	5000_MPE	67	59	8	1	4.5	C
23350100	Ostrich, cooked	5000_MPE	67	59	8	1	4	B
24103050	Chicken, NS as to part, grilled without sauce, NS as to skin eaten	5000_MPE	67	59	8	1	4	A
24103060	Chicken, NS as to part, grilled without sauce, skin not eaten	5000_MPE	67	59	8	1	4	A
24104051	Chicken, NS as to part, sauteed, skin not eaten	5000_MPE	67	59	8	1	4	A
32110150	Shrimp-egg patty	5000_MPE	67	59	8	2	1	E
24201320	Turkey, light and dark meat, roasted, skin not eaten	5000_MPE	67	58	9	1	4	A
24202010	Turkey, drumstick, cooked, skin not eaten	5000_MPE	67	58	9	1	4	A
24202060	Turkey, drumstick, roasted, skin not eaten	5000_MPE	67	58	9	1	4	A
32104950	Egg omelet or scrambled egg, fat not added in cooking	5000_MPE	67	54	13	1	3.5	C

23150270	Goat head, cooked	5000_MPE	67	49	18	1	4	C
33401200	Egg substitute, omelet, scrambled, or fried, with vegetables	5000_MPE	66	70	-4	3	3.5	B
24201310	Turkey, light and dark meat, roasted, NS as to skin eaten	5000_MPE	66	58	8	1	4	B
24201330	Turkey, light and dark meat, roasted, skin eaten	5000_MPE	66	58	8	1	4	B
32130630	Egg omelet or scrambled egg, with cheese and dark-green vegetables, fat added	5000_MPE	66	58	8	2	3	D
32130650	Egg omelet or scrambled egg, with cheese and dark-green vegetables, NS as to fat	5000_MPE	66	58	8	2	3	D
24142501	Chicken drumstick, grilled without sauce, skin not eaten	5000_MPE	66	57	9	1	4	B
24201000	Turkey, NFS	5000_MPE	66	57	9	1	4	A
24201010	Turkey, light meat, cooked, NS as to skin eaten	5000_MPE	66	57	9	1	4	A
24201020	Turkey, light meat, skin not eaten	5000_MPE	66	57	9	1	4	A
24201120	Turkey, light meat, roasted, skin not eaten	5000_MPE	66	57	9	1	4	A
24201410	Turkey, light or dark meat, stewed, skin not eaten	5000_MPE	66	57	9	1	4	B
24203010	Turkey, wing, cooked, skin not eaten	5000_MPE	66	57	9	1	4	A
24144301	Chicken drumstick, sauteed, skin not eaten	5000_MPE	66	56	10	1	4	B
24147401	Chicken drumstick, baked, coated, skin / coating not eaten	5000_MPE	66	56	10	1	4	B
32130490	Egg omelet or scrambled egg, with vegetables other than dark green and/or tomatoes, fat added	5000_MPE	66	56	10	1	3.5	C
32130510	Egg omelet or scrambled egg, with vegetables other than dark green and/or tomatoes, NS as to fat	5000_MPE	66	56	10	1	3.5	C
32130670	Egg omelet or scrambled egg, with cheese, tomatoes, and dark-green vegetables, no added fat	5000_MPE	66	56	10	2	3.5	C
24400020	Cornish game hen, cooked, skin not eaten	5000_MPE	66	54	12	1	4	A
24401020	Cornish game hen, roasted, skin not eaten	5000_MPE	66	54	12	1	4	A
31104000	Egg, whole, poached	5000_MPE	66	53	13	1	3.5	B
33001100	Egg substitute, cheese flavored, omelet, scrambled, or fried, fat added in cooking	5000_MPE	65	68	-3	3	3.5	C
33001120	Egg substitute, cheese flavored, omelet, scrambled, or fried, NS as to fat added in cooking	5000_MPE	65	68	-3	3	3.5	C
33001200	Egg substitute, vegetable flavored, omelet, scrambled, or fried, fat added in cooking	5000_MPE	65	68	-3	3	3.5	C
33001220	Egg substitute, vegetable flavored, omelet, scrambled, or fried, NS as to fat added in cooking	5000_MPE	65	68	-3	3	3.5	C
32400010	Egg white omelet or scrambled egg, NS as to fat added in cooking	5000_MPE	65	65	0	2	4	A
32400012	Egg white omelet or scrambled egg, fat added in cooking	5000_MPE	65	65	0	2	4	A
31201000	Duck egg, cooked	5000_MPE	65	59	6	1	3.5	C
32400310	Egg white, omelet, scrambled, or fried, with vegetables, fat not added in cooking	5000_MPE	65	58	7	1	4	A
24201030	Turkey, light meat, skin eaten	5000_MPE	65	57	8	1	4	B
24201110	Turkey, light meat, roasted, NS as to skin eaten	5000_MPE	65	57	8	1	4	B
24201130	Turkey, light meat, roasted, skin eaten	5000_MPE	65	57	8	1	4	B
24202460	Turkey, thigh, cooked, skin eaten	5000_MPE	65	57	8	1	4	B
24402110	Dove, fried	5000_MPE	65	57	8	1	4	A

24201210	Turkey, dark meat, roasted, NS as to skin eaten	5000_MPE	65	55	10	1	4	B
24201230	Turkey, dark meat, roasted, skin eaten	5000_MPE	65	55	10	1	4	B
32105100	Egg omelet or scrambled egg, with potatoes and/or onions (Tortilla Espanola, traditional style Spanish omelet)	5000_MPE	65	55	10	1	3.5	C
32130000	Egg omelet or scrambled egg, made with margarine	5000_MPE	65	55	10	2	3.5	C
24142301	Chicken drumstick, baked, broiled, or roasted, skin not eaten, from raw	5000_MPE	65	54	11	1	4	A
32130400	Egg omelet or scrambled egg, with tomatoes, fat added	5000_MPE	65	54	11	1	3.5	C
32130420	Egg omelet or scrambled egg, with tomatoes, NS as to fat	5000_MPE	65	54	11	1	3.5	C
32130610	Egg omelet or scrambled egg, with cheese and tomatoes, no added fat	5000_MPE	65	54	11	2	3.5	C
32130700	Egg omelet or scrambled egg, with cheese and vegetables other than dark green and/or tomatoes, no added fat	5000_MPE	65	54	11	2	3.5	C
31111010	Egg, yolk only, cooked, no added fat	5000_MPE	65	52	13	1	2	D
33401420	Egg substitute, omelet, scrambled, or fried, with cheese and vegetables, NS as to fat added in cooking	5000_MPE	64	67	-3	3	3.5	C
31202000	Goose egg, cooked	5000_MPE	64	60	4	1	3.5	C
24198683	Chicken fillet, grilled	5000_MPE	64	58	6	1	4	A
24124200	Chicken breast, sauteed, skin eaten	5000_MPE	64	57	7	1	4	B
24123300	Chicken breast, grilled without sauce, skin eaten	5000_MPE	64	56	8	1	4	B
32105045	Egg omelet or scrambled egg, with cheese and dark-green vegetables	5000_MPE	64	56	8	2	3	C
32130110	Egg omelet or scrambled egg, with cheese, made with oil	5000_MPE	64	56	8	2	3	D
24134101	Chicken leg, drumstick and thigh, grilled without sauce, skin not eaten	5000_MPE	64	55	9	1	4	B
24134301	Chicken leg, drumstick and thigh, sauteed, skin not eaten	5000_MPE	64	55	9	1	4	B
24137311	Chicken leg, drumstick and thigh, baked, coated, skin / coating not eaten	5000_MPE	64	55	9	1	4	B
32105180	Huevos rancheros	5000_MPE	64	55	9	2	3.5	C
32130660	Egg omelet or scrambled egg, with cheese, tomatoes, and dark-green vegetables, fat added	5000_MPE	64	55	9	2	3	D
32130680	Egg omelet or scrambled egg, with cheese, tomatoes, and dark-green vegetables, NS as to fat	5000_MPE	64	55	9	2	3	D
24102000	Chicken, NS as to part, baked, broiled, or roasted, NS as to skin eaten	5000_MPE	64	54	10	1	4	A
24102020	Chicken, NS as to part, baked, broiled, or roasted, skin not eaten	5000_MPE	64	54	10	1	4	A
24132231	Chicken leg, drumstick and thigh, baked or broiled, skin not eaten	5000_MPE	64	54	10	1	4	A
24140200	Chicken, drumstick, NS as to cooking method, NS as to skin eaten	5000_MPE	64	54	10	1	4	A
24142200	Chicken, drumstick, roasted, broiled, or baked, NS as to skin eaten	5000_MPE	64	54	10	1	4	A
24142220	Chicken, drumstick, roasted, broiled, or baked, skin not eaten	5000_MPE	64	54	10	1	4	A
32131200	Egg omelet or scrambled egg, with potatoes and/or onions, fat added	5000_MPE	64	54	10	1	3.5	C
32131220	Egg omelet or scrambled egg, with potatoes and/or onions, NS as to fat	5000_MPE	64	54	10	1	3.5	C
24103000	Chicken, NS as to part, stewed, NS as to skin eaten	5000_MPE	64	53	11	1	4	A
24103020	Chicken, NS as to part, stewed, skin not eaten	5000_MPE	64	53	11	1	4	A

24123120	Chicken breast, stewed, skin not eaten	5000_MPE	64	53	11	1	4.5	A
24152231	Chicken thigh, baked, broiled, or roasted, skin not eaten, from raw	5000_MPE	64	53	11	1	4	B
32104900	Egg omelet or scrambled egg, NS as to fat added in cooking	5000_MPE	64	53	11	2	3.5	C
24160120	Chicken, wing, NS as to cooking method, skin not eaten	5000_MPE	64	51	13	1	4	B
24162120	Chicken, wing, roasted, broiled, or baked, skin not eaten	5000_MPE	64	51	13	1	4	B
32110100	Eggs a la Malaguena, Puerto Rican style	5000_MPE	64	51	13	2	3.5	C
32400065	Egg white omelet, scrambled, or fried, made with oil	5000_MPE	63	63	0	2	4	A
32105310	Ripe plantain omelet, Puerto Rican style	5000_MPE	63	56	7	1	3	D
24127201	Chicken breast, fried, coated, skin / coating not eaten, from raw	5000_MPE	63	55	8	1	4	A
24154011	Chicken thigh, grilled without sauce, skin not eaten	5000_MPE	63	55	8	1	4	B
24154301	Chicken thigh, sauteed, skin not eaten	5000_MPE	63	54	9	1	4	B
24157401	Chicken thigh, baked, coated, skin / coating not eaten	5000_MPE	63	54	9	1	4	B
24201360	Turkey, light or dark meat, fried, coated, skin not eaten	5000_MPE	63	54	9	1	4	A
24122130	Chicken breast, baked, broiled, or roasted, skin eaten, from raw	5000_MPE	63	53	10	1	4	B
24122131	Chicken breast, baked, broiled, or roasted, skin not eaten, from raw	5000_MPE	63	53	10	1	4.5	A
24122171	Chicken breast, rotisserie, skin not eaten	5000_MPE	63	53	10	1	4.5	A
31105030	Egg, whole, fried with oil	5000_MPE	63	53	10	1	3.5	C
31105090	Egg, whole, fried, from fast food / restaurant	5000_MPE	63	53	10	1	3.5	C
31106000	Egg, whole, baked, NS as to fat	5000_MPE	63	53	10	1	3.5	C
31106020	Egg, whole, baked, fat added	5000_MPE	63	53	10	1	3.5	C
32129990	Egg omelet or scrambled egg, NS as to fat	5000_MPE	63	53	10	1	3.5	C
32130065	Egg omelet or scrambled egg, NS as to fat type	5000_MPE	63	53	10	1	3.5	C
32130690	Egg omelet or scrambled egg, with cheese and vegetables other than dark green and/or tomatoes, fat added	5000_MPE	63	53	10	2	3	D
32130710	Egg omelet or scrambled egg, with cheese and vegetables other than dark green and/or tomatoes, NS as to fat	5000_MPE	63	53	10	2	3	D
24130200	Chicken, leg (drumstick and thigh), NS as to cooking method, NS as to skin eaten	5000_MPE	63	52	11	1	4	B
24132200	Chicken, leg (drumstick and thigh), roasted, broiled, or baked, NS as to skin eaten	5000_MPE	63	52	11	1	4	B
24132220	Chicken, leg (drumstick and thigh), roasted, broiled, or baked, skin not eaten	5000_MPE	63	52	11	1	4	B
24140220	Chicken drumstick, NS as to cooking method, skin not eaten	5000_MPE	63	52	11	1	4	B
24142401	Chicken drumstick, rotisserie, skin not eaten	5000_MPE	63	52	11	1	4	B
24202000	Turkey, drumstick, cooked, NS as to skin eaten	5000_MPE	63	52	11	1	4	C
24202050	Turkey, drumstick, roasted, NS as to skin eaten	5000_MPE	63	52	11	1	4	C
24202070	Turkey, drumstick, roasted, skin eaten	5000_MPE	63	52	11	1	4	C
24202600	Turkey, neck	5000_MPE	63	52	11	1	3.5	D
32130160	Egg omelet or scrambled egg, with cheese, made with cooking spray	5000_MPE	63	52	11	2	3.5	C
32130170	Egg omelet or scrambled egg, with cheese, no added fat	5000_MPE	63	52	11	2	3	C
24164120	Chicken, wing, fried, no coating, skin not eaten, NS as to type of fat added in cooking	5000_MPE	63	51	12	1	4	B

24164123	Chicken, wing, fried, no coating, skin not eaten, made with oil	5000_MPE	63	51	12	1	4	B
24167120	Chicken, wing, coated, baked or fried, prepared with skin, skin/coating not eaten, NS as to type of fat added in cooking	5000_MPE	63	51	12	1	4	B
24167123	Chicken, wing, coated, baked or fried, prepared with skin, skin/coating not eaten, made with oil	5000_MPE	63	51	12	1	4	B
31103000	Egg, whole, boiled	5000_MPE	63	51	12	1	3.5	B
31111000	Egg, yolk only, cooked, NS as to fat	5000_MPE	63	51	12	1	2	D
31111020	Egg, yolk only, cooked, fat added	5000_MPE	63	51	12	1	2	D
32130840	Egg omelet or scrambled egg, with meat and dark-green vegetables, no added fat	5000_MPE	63	50	13	1	3.5	B
24104000	Chicken, NS as to part, fried, no coating, NS as to skin eaten, fat added in cooking	5000_MPE	62	54	8	1	4	B
24104020	Chicken, NS as to part, fried, no coating, skin not eaten, fat added in cooking	5000_MPE	62	54	8	1	4	B
24107020	Chicken, NS as to part, coated, baked or fried, prepared with skin, skin/coating not eaten, fat added in cooking	5000_MPE	62	54	8	1	4	B
24107060	Chicken, NS as to part, coated, baked or fried, prepared skinless, coating not eaten, fat added in cooking	5000_MPE	62	54	8	1	4	B
24122160	Chicken breast, baked, broiled, or roasted with marinade, skin eaten, from raw	5000_MPE	62	54	8	1	4	B
24124100	Chicken, breast, fried, no coating, NS as to skin eaten, fat added in cooking	5000_MPE	62	54	8	1	4	B
24124120	Chicken, breast, fried, no coating, skin not eaten, NS as to type of fat added in cooking	5000_MPE	62	54	8	1	4	B
24124123	Chicken, breast, fried, no coating, skin not eaten, made with oil	5000_MPE	62	54	8	1	4	B
24127120	Chicken, breast, coated, baked or fried, prepared with skin, skin/coating not eaten, NS as to type of fat added in cooking	5000_MPE	62	54	8	1	4	B
24127160	Chicken, breast, coated, baked or fried, prepared skinless, coating not eaten, NS as to type of fat added in cooking	5000_MPE	62	54	8	1	4	B
24127163	Chicken, breast, coated, baked or fried, prepared skinless, coating not eaten, made with oil	5000_MPE	62	54	8	1	4	B
32102000	Egg, deviled	5000_MPE	62	54	8	2	3	C
24203020	Turkey, wing, cooked, skin eaten	5000_MPE	62	53	9	1	4	B
32130620	Egg omelet or scrambled egg, with cheese and tomatoes, NS as to fat	5000_MPE	62	53	9	2	3	D
24102050	Chicken, NS as to part, rotisserie, NS as to skin eaten	5000_MPE	62	52	10	1	4	A
24102070	Chicken, NS as to part, rotisserie, skin not eaten	5000_MPE	62	52	10	1	4	A
24120120	Chicken breast, NS as to cooking method, skin not eaten	5000_MPE	62	52	10	1	4.5	A
24123110	Chicken breast, stewed, skin eaten	5000_MPE	62	52	10	1	4	A
24147301	Chicken drumstick, fried, coated, skin / coating not eaten, from raw	5000_MPE	62	52	10	1	4	A
24150200	Chicken, thigh, NS as to cooking method, NS as to skin eaten	5000_MPE	62	52	10	1	4	B
24152200	Chicken, thigh, roasted, broiled, or baked, NS as to skin eaten	5000_MPE	62	52	10	1	4	B
24152220	Chicken, thigh, roasted, broiled, or baked, skin not eaten	5000_MPE	62	52	10	1	4	B
32130100	Egg omelet or scrambled egg, with cheese, made with margarine	5000_MPE	62	52	10	2	3	D
32130600	Egg omelet or scrambled egg, with cheese and tomatoes, fat added	5000_MPE	62	52	10	2	3	D
89901006	Chicken, for use with vegetables	5000_MPE	62	52	10	1	4	A
24100000	Chicken, NS as to part and cooking method, NS as to skin eaten	5000_MPE	62	51	11	1	4	A

24100020	Chicken, NS as to part and cooking method, skin not eaten	5000_MPE	62	51	11	1	4	A
24120100	Chicken, breast, NS as to cooking method, NS as to skin eaten	5000_MPE	62	51	11	1	4	B
24122100	Chicken, breast, roasted, broiled, or baked, NS as to skin eaten	5000_MPE	62	51	11	1	4	B
24122110	Chicken, breast, roasted, broiled, or baked, skin eaten	5000_MPE	62	51	11	1	4	B
24122120	Chicken, breast, roasted, broiled, or baked, skin not eaten	5000_MPE	62	51	11	1	4	B
24143220	Chicken drumstick, stewed, skin not eaten	5000_MPE	62	51	11	1	4	A
25170420	Gizzard, cooked	5000_MPE	62	51	11	1	4.5	C
32105050	Egg omelet or scrambled egg, with vegetables other than dark-green	5000_MPE	62	51	11	2	3.5	C
23335100	Raccoon, cooked	5000_MPE	62	50	12	1	4	C
31105010	Egg, whole, fried no added fat	5000_MPE	62	48	14	1	3.5	B
31105080	Egg, whole, fried with cooking spray	5000_MPE	62	48	14	1	3.5	B
23324100	Caribou, cooked	5000_MPE	62	47	15	1	4.5	C
24127202	Chicken breast, fried, coated, prepared skinless, coating eaten, from raw	5000_MPE	61	56	5	1	4	A
24201350	Turkey, light or dark meat, battered, fried, NS as to skin eaten	5000_MPE	61	53	8	1	4	A
24201370	Turkey, light or dark meat, fried, coated, skin eaten	5000_MPE	61	53	8	1	4	A
32103020	Egg salad, made with mayonnaise-type salad dressing	5000_MPE	61	53	8	2	3	C
24142500	Chicken drumstick, grilled without sauce, skin eaten	5000_MPE	61	52	9	1	4	B
24154223	Chicken, thigh, fried, no coating, skin not eaten, made with oil	5000_MPE	61	52	9	1	4	B
24157223	Chicken, thigh, coated, baked or fried, prepared with skin, skin/coating not eaten, made with oil	5000_MPE	61	52	9	1	4	B
32105150	Egg omelet or scrambled egg, with cheese, beans, tomatoes, and chili sauce	5000_MPE	61	52	9	2	3	C
24133220	Chicken leg, drumstick and thigh, stewed, skin not eaten	5000_MPE	61	51	10	1	4	B
24154200	Chicken, thigh, fried, no coating, NS as to skin eaten, fat added in cooking	5000_MPE	61	51	10	1	4	B
24154220	Chicken, thigh, fried, no coating, skin not eaten, NS as to type of fat added in cooking	5000_MPE	61	51	10	1	4	B
24157220	Chicken, thigh, coated, baked or fried, prepared with skin, skin/coating not eaten, NS as to type of fat added in cooking	5000_MPE	61	51	10	1	4	B
24157260	Chicken, thigh, coated, baked or fried, prepared skinless, coating not eaten, NS as to type of fat added in cooking	5000_MPE	61	51	10	1	4	B
24157263	Chicken, thigh, coated, baked or fried, prepared skinless, coating not eaten, made with oil	5000_MPE	61	51	10	1	4	B
24132241	Chicken leg, drumstick and thigh, rotisserie, skin not eaten	5000_MPE	61	50	11	1	4	B
24153220	Chicken thigh, stewed, skin not eaten	5000_MPE	61	50	11	1	4	B
24202020	Turkey, drumstick, cooked, skin eaten	5000_MPE	61	50	11	1	4	B
24202450	Turkey, thigh, cooked, NS as to skin eaten	5000_MPE	61	50	11	1	4	B
32105000	Egg omelet or scrambled egg, fat added in cooking	5000_MPE	61	50	11	2	3.5	C
32130830	Egg omelet or scrambled egg, with meat and dark-green vegetables, fat added	5000_MPE	61	50	11	2	3	C
32130850	Egg omelet or scrambled egg, with meat and dark-green vegetables, NS as to fat	5000_MPE	61	50	11	2	3	C
24143200	Chicken, drumstick, stewed, NS as to skin eaten	5000_MPE	61	49	12	1	4	B

32400300	Egg white, omelet, scrambled, or fried, with vegetables	5000_MPE	60	58	2	2	3.5	B
32103000	Egg salad, made with mayonnaise	5000_MPE	60	56	4	3	3	D
33201500	Scrambled egg, made from cholesterol-free frozen mixture with vegetables	5000_MPE	60	56	4	2	4	B
24127500	Chicken breast, baked, coated, skin / coating eaten	5000_MPE	60	52	8	1	4	A
24300120	Duck, cooked, skin not eaten	5000_MPE	60	52	8	1	4	A
24301000	Duck, roasted, NS as to skin eaten	5000_MPE	60	52	8	1	4	A
24301020	Duck, roasted, skin not eaten	5000_MPE	60	52	8	1	4	A
24404100	Pheasant, cooked	5000_MPE	60	52	8	1	4	A
24134200	Chicken, leg (drumstick and thigh), fried, no coating, NS as to skin eaten, fat added in cooking	5000_MPE	60	51	9	1	4	B
24134220	Chicken, leg (drumstick and thigh), fried, no coating, skin not eaten, fat added in cooking	5000_MPE	60	51	9	1	4	B
24137220	Chicken, leg (drumstick and thigh), coated, baked or fried, prepared with skin, skin/coating not eaten, fat added in cooking	5000_MPE	60	51	9	1	4	B
24137260	Chicken, leg (drumstick and thigh), coated, baked or fried, prepared skinless, coating not eaten, fat added in cooking	5000_MPE	60	51	9	1	4	B
24144200	Chicken, drumstick, fried, no coating, NS as to skin eaten, fat added in cooking	5000_MPE	60	51	9	1	4	B
24144220	Chicken, drumstick, fried, no coating, skin not eaten, NS as to type of fat added in cooking	5000_MPE	60	51	9	1	4	B
24144223	Chicken, drumstick, fried, no coating, skin not eaten, made with oil	5000_MPE	60	51	9	1	4	B
24147220	Chicken, drumstick, coated, baked or fried, prepared with skin, skin/coating not eaten, NS as to type of fat added in cooking	5000_MPE	60	51	9	1	4	B
24147223	Chicken, drumstick, coated, baked or fried, prepared with skin, skin/coating not eaten, made with oil	5000_MPE	60	51	9	1	4	B
24147260	Chicken, drumstick, coated, baked or fried, prepared skinless, coating not eaten, NS as to type of fat added in cooking	5000_MPE	60	51	9	1	4	B
24147263	Chicken, drumstick, coated, baked or fried, prepared skinless, coating not eaten, made with oil	5000_MPE	60	51	9	1	4	B
24201400	Turkey, light or dark meat, stewed, NS as to skin eaten	5000_MPE	60	51	9	1	4	C
24201420	Turkey light or dark meat, stewed, skin eaten	5000_MPE	60	51	9	1	4	C
32103045	Egg salad, made with light Italian dressing	5000_MPE	60	51	9	2	3	C
23323500	Bear, cooked	5000_MPE	60	50	10	1	4	C
24102010	Chicken, NS as to part, baked, broiled, or roasted, skin eaten	5000_MPE	60	50	10	1	4	B
24137301	Chicken leg, drumstick and thigh, fried, coated, skin / coating not eaten	5000_MPE	60	50	10	1	4	B
24123100	Chicken, breast, stewed, NS as to skin eaten	5000_MPE	60	49	11	1	4	B
24130220	Chicken leg, drumstick and thigh, NS as to cooking method, skin not eaten	5000_MPE	60	49	11	1	4	B
24142300	Chicken drumstick, baked, broiled, or roasted, skin eaten, from raw	5000_MPE	60	49	11	1	4	B
24198670	Chicken, chicken roll, roasted	5000_MPE	60	49	11	1	4	B
32105048	Egg omelet or scrambled egg, with mushrooms	5000_MPE	60	49	11	2	3.5	B
24133200	Chicken, leg (drumstick and thigh), stewed, NS as to skin eaten	5000_MPE	60	48	12	1	4	B
31105020	Egg, whole, fried with margarine	5000_MPE	60	48	12	2	3.5	C
32130900	Egg omelet or scrambled egg, with meat and vegetables other than dark-green and/or tomatoes, no added fat	5000_MPE	60	46	14	1	3.5	B

24164125	Chicken, wing, fried, no coating, skin not eaten, made without fat	5000_MPE	60	45	15	1	4	B
24167119	Chicken, wing, coated, baked or fried, prepared with skin, skin/coating not eaten, made without fat	5000_MPE	60	45	15	1	4	B
23321250	Venison/deer steak, breaded or floured, cooked, NS as to cooking method	5000_MPE	59	55	4	2	4	C
32103030	Egg salad, made with creamy dressing	5000_MPE	59	55	4	2	3	D
24107050	Chicken, NS as to part, coated, baked or fried, prepared skinless, coating eaten, fat added in cooking	5000_MPE	59	53	6	1	4	B
24127140	Chicken, breast, coated, baked or fried, prepared skinless, NS as to coating eaten, fat added in cooking	5000_MPE	59	53	6	1	4	B
24127150	Chicken, breast, coated, baked or fried, prepared skinless, coating eaten, NS as to type of fat added in cooking	5000_MPE	59	53	6	1	4	B
24127153	Chicken, breast, coated, baked or fried, prepared skinless, coating eaten, made with oil	5000_MPE	59	53	6	1	4	B
24201060	Turkey, light meat, breaded, baked or fried, skin not eaten	5000_MPE	59	53	6	2	4.5	A
24123311	Chicken breast, grilled with sauce, skin not eaten	5000_MPE	59	52	7	2	4	B
24137250	Chicken, leg (drumstick and thigh), coated, baked or fried, prepared skinless, coating eaten, fat added in cooking	5000_MPE	59	52	7	1	4	A
32103025	Egg salad, made with light mayonnaise-type salad dressing	5000_MPE	59	52	7	2	3	C
24144300	Chicken drumstick, sauteed, skin eaten	5000_MPE	59	50	9	1	4	B
24103010	Chicken, NS as to part, stewed, skin eaten	5000_MPE	59	49	10	1	4	B
24120110	Chicken breast, NS as to cooking method, skin eaten	5000_MPE	59	49	10	1	4	A
24122170	Chicken breast, rotisserie, skin eaten	5000_MPE	59	49	10	1	4	A
24124124	Chicken, breast, fried, no coating, skin not eaten, made with cooking spray	5000_MPE	59	49	10	1	4	B
24142210	Chicken, drumstick, roasted, broiled, or baked, skin eaten	5000_MPE	59	49	10	1	3.5	B
24157301	Chicken thigh, fried, coated, skin / coating not eaten, from raw	5000_MPE	59	49	10	1	4	B
32104100	Egg, scrambled, made from dry eggs	5000_MPE	59	49	10	2	3	C
89901004	Beef, for use with vegetables	5000_MPE	59	49	10	1	4.5	B
24154224	Chicken, thigh, fried, no coating, skin not eaten, made with cooking spray	5000_MPE	59	48	11	1	4	B
32103050	Egg Salad, made with any type of fat free dressing	5000_MPE	59	47	12	1	3.5	C
24153200	Chicken, thigh, stewed, NS as to skin eaten	5000_MPE	59	46	13	1	4	B
24163120	Chicken, wing, stewed, skin not eaten	5000_MPE	59	46	13	1	4	A
24198570	Chicken, canned, meat only	5000_MPE	59	46	13	3	4	C
33401410	Egg substitute, omelet, scrambled, or fried, with cheese and vegetables, fat not added in cooking	5000_MPE	58	57	1	3	3.5	C
24107040	Chicken, NS as to part, coated, baked or fried, prepared skinless, NS as to coating eaten, fat added in cooking	5000_MPE	58	52	6	1	4	C
24201070	Turkey, light meat, breaded, baked or fried, skin eaten	5000_MPE	58	52	6	2	4	A
32103015	Egg salad, made with light mayonnaise	5000_MPE	58	51	7	2	3	C
24134100	Chicken leg, drumstick and thigh, grilled without sauce, skin eaten	5000_MPE	58	49	9	1	3.5	C
24134300	Chicken leg, drumstick and thigh, sauteed, skin eaten	5000_MPE	58	49	9	1	3.5	C
32103035	Egg salad, made with light creamy dressing	5000_MPE	58	49	9	2	3.5	C
32105049	Egg omelet or scrambled egg, with cheese and mushrooms	5000_MPE	58	49	9	2	3	D

24100010	Chicken, NS as to part and cooking method, skin eaten	5000_MPE	58	48	10	1	4	B
24102060	Chicken, NS as to part, rotisserie, skin eaten	5000_MPE	58	48	10	1	4	B
24124110	Chicken, breast, fried, no coating, skin eaten, NS as to type of fat added in cooking	5000_MPE	58	48	10	1	4	C
24124113	Chicken, breast, fried, no coating, skin eaten, made with oil	5000_MPE	58	48	10	1	4	C
24132230	Chicken leg, drumstick and thigh, baked or broiled, skin eaten	5000_MPE	58	48	10	1	4	B
24144210	Chicken, drumstick, fried, no coating, skin eaten, NS as to type of fat added in cooking	5000_MPE	58	48	10	1	3.5	D
24144213	Chicken, drumstick, fried, no coating, skin eaten, made with oil	5000_MPE	58	48	10	1	3.5	D
24152301	Chicken thigh, rotisserie, skin not eaten	5000_MPE	58	48	10	1	4	B
24203000	Turkey, wing, cooked, NS as to skin eaten	5000_MPE	58	48	10	1	4	B
24140210	Chicken drumstick, NS as to cooking method, skin eaten	5000_MPE	58	47	11	1	4	B
24142400	Chicken drumstick, rotisserie, skin eaten	5000_MPE	58	47	11	1	4	B
24143210	Chicken drumstick, stewed, skin eaten	5000_MPE	58	47	11	1	4	B
24150220	Chicken thigh, NS as to cooking method, skin not eaten	5000_MPE	58	47	11	1	4	B
24154225	Chicken, thigh, fried, no coating, skin not eaten, made without fat	5000_MPE	58	47	11	1	4	B
31105005	Egg, whole, fried, NS as to fat	5000_MPE	58	47	11	1	3.5	C
32105110	Egg omelet or scrambled egg, with beef	5000_MPE	58	47	11	1	3.5	C
32131070	Egg omelet or scrambled egg, with cheese, meat, tomatoes, and dark-green vegetables, no added fat	5000_MPE	58	47	11	2	3.5	C
24144225	Chicken, drumstick, fried, no coating, skin not eaten, made without fat	5000_MPE	58	46	12	1	4	B
24147265	Chicken, drumstick, coated, baked or fried, prepared skinless, coating not eaten, made without fat	5000_MPE	58	46	12	1	4	B
31105085	Egg, whole, fried, NS as to fat type	5000_MPE	58	46	12	1	3.5	C
32131040	Egg omelet or scrambled egg, with cheese, meat, and dark-green vegetables, no added fat	5000_MPE	58	46	12	2	3	C
32130810	Egg omelet or scrambled egg, with meat and tomatoes, no added fat	5000_MPE	58	44	14	1	3.5	B
24201050	Turkey, light meat, breaded, baked or fried, NS as to skin eaten	5000_MPE	57	51	6	2	4	A
23334100	Beaver, cooked	5000_MPE	57	48	9	1	4	C
32130310	Egg omelet or scrambled egg, with cheese and meat, made with oil	5000_MPE	57	48	9	2	3	D
24132210	Chicken, leg (drumstick and thigh), roasted, broiled, or baked, skin eaten	5000_MPE	57	47	10	1	3.5	C
32105055	Egg omelet or scrambled egg, with cheese and vegetables other than dark-green	5000_MPE	57	47	10	2	3	C
32131060	Egg omelet or scrambled egg, with cheese, meat, tomatoes, and dark-green vegetables, fat added	5000_MPE	57	47	10	2	3	D
32131080	Egg omelet or scrambled egg, with cheese, meat, tomatoes, and dark-green vegetables, NS as to fat	5000_MPE	57	47	10	2	3	D
32130890	Egg omelet or scrambled egg, with meat and vegetables other than dark-green and/or tomatoes, fat added	5000_MPE	57	46	11	2	3	C
32130910	Egg omelet or scrambled egg, with meat and vegetables other than dark-green and/or tomatoes, NS as to fat	5000_MPE	57	46	11	2	3	C
24127165	Chicken, breast, coated, baked or fried, prepared skinless, coating not eaten, made without fat	5000_MPE	57	45	12	1	4	B

32130040	Egg omelet or scrambled egg, made with animal fat or meat drippings	5000_MPE	57	44	13	1	3	C
32400520	Egg white, omelet, scrambled, or fried, with cheese and vegetables, NS as to fat added in cooking	5000_MPE	56	56	0	2	3.5	C
31109000	Egg, white only, cooked, NS as to fat added in cooking	5000_MPE	56	53	3	2	4	B
31109020	Egg, white only, cooked, fat added in cooking	5000_MPE	56	53	3	2	4	B
25112200	Liver paste or pate, chicken	5000_MPE	56	50	6	3	3.5	C
24103070	Chicken, NS as to part, grilled with sauce, NS as to skin eaten	5000_MPE	56	48	8	2	3.5	C
24103080	Chicken, NS as to part, grilled with sauce, skin not eaten	5000_MPE	56	48	8	2	3.5	C
31203000	Quail egg, canned	5000_MPE	56	48	8	3	3.5	B
24124121	Chicken, breast, fried, no coating, skin not eaten, made with shortening	5000_MPE	56	47	9	1	4	B
24154010	Chicken thigh, grilled without sauce, skin eaten	5000_MPE	56	47	9	1	3.5	D
24154300	Chicken thigh, sauteed, skin eaten	5000_MPE	56	47	9	1	3.5	D
24152230	Chicken thigh, baked, broiled, or roasted, skin eaten, from raw	5000_MPE	56	46	10	1	3.5	C
31107000	Egg, whole, pickled	5000_MPE	56	46	10	3	3.5	C
32105116	Egg omelet or scrambled egg, with sausage and dark-green vegetables	5000_MPE	56	46	10	2	3.5	C
32131030	Egg omelet or scrambled egg, with cheese, meat, and dark-green vegetables, fat added	5000_MPE	56	46	10	2	3	D
32131050	Egg omelet or scrambled egg, with cheese, meat, and dark-green vegetables, NS as to fat	5000_MPE	56	46	10	2	3	D
24104010	Chicken, NS as to part, fried, no coating, skin eaten, fat added in cooking	5000_MPE	56	45	11	1	3.5	D
24124125	Chicken, breast, fried, no coating, skin not eaten, made without fat	5000_MPE	56	45	11	1	4	B
32130210	Egg omelet or scrambled egg, with meat, made with oil	5000_MPE	56	45	11	2	3.5	C
32130800	Egg omelet or scrambled egg, with meat and tomatoes, fat added	5000_MPE	56	45	11	2	3	C
32130820	Egg omelet or scrambled egg, with meat and tomatoes, NS as to fat	5000_MPE	56	45	11	2	3	C
32131020	Egg omelet or scrambled egg, with cheese, meat, and tomatoes, NS as to fat	5000_MPE	56	45	11	2	3	D
24124115	Chicken, breast, fried, no coating, skin eaten, made without fat	5000_MPE	56	44	12	1	4	C
24127115	Chicken, breast, coated, baked or fried, prepared with skin, skin/coating eaten, made without fat	5000_MPE	56	44	12	1	4	A
24147255	Chicken, drumstick, coated, baked or fried, prepared skinless, coating eaten, made without fat	5000_MPE	56	44	12	1	4	A
32130140	Egg omelet or scrambled egg, with cheese, made with animal fat or meat drippings	5000_MPE	56	44	12	2	3	D
32131010	Egg omelet or scrambled egg, with cheese, meat, and tomatoes, no added fat	5000_MPE	56	44	12	2	3	C
32131100	Egg omelet or scrambled egg, with cheese, meat, and vegetables other than dark-green and/or tomatoes, no added fat	5000_MPE	56	44	12	2	3	C
24144215	Chicken, drumstick, fried, no coating, skin eaten, made without fat	5000_MPE	56	43	13	1	4	C
31105000	Egg, whole, fried	5000_MPE	56	43	13	2	3	C
31108100	Egg, white, cooked, NS as to fat	5000_MPE	55	53	2	2	3.5	B
31108120	Egg, white, cooked, fat added	5000_MPE	55	53	2	2	3.5	B
32400060	Egg white omelet, scrambled, or fried, made with margarine	5000_MPE	55	52	3	2	4	A

32400510	Egg white, omelet, scrambled, or fried, with cheese and vegetables, fat not added in cooking	5000_MPE	55	50	5	2	3.5	C
24137240	Chicken, leg (drumstick and thigh), coated, baked or fried, prepared skinless, NS as to coating eaten, fat added in cooking	5000_MPE	55	48	7	1	3.5	C
24147240	Chicken, drumstick, coated, baked or fried, prepared skinless, NS as to coating eaten, fat added in cooking	5000_MPE	55	48	7	1	3.5	C
24147250	Chicken, drumstick, coated, baked or fried, prepared skinless, coating eaten, NS as to type of fat added in cooking	5000_MPE	55	48	7	1	3.5	C
24147253	Chicken, drumstick, coated, baked or fried, prepared skinless, coating eaten, made with oil	5000_MPE	55	48	7	1	3.5	C
24157240	Chicken, thigh, coated, baked or fried, prepared skinless, NS as to coating eaten, fat added in cooking	5000_MPE	55	48	7	1	3.5	C
24157250	Chicken, thigh, coated, baked or fried, prepared skinless, coating eaten, NS as to type of fat added in cooking	5000_MPE	55	48	7	1	3.5	C
24157253	Chicken, thigh, coated, baked or fried, prepared skinless, coating eaten, made with oil	5000_MPE	55	48	7	1	3.5	C
32103040	Egg salad, made with Italian dressing	5000_MPE	55	48	7	2	3	C
23322300	Deer chop, cooked	5000_MPE	55	47	8	2	4	C
24147302	Chicken drumstick, fried, coated, prepared skinless, coating eaten, from raw	5000_MPE	55	47	8	1	3.5	B
24311010	Goose, wild, roasted	5000_MPE	55	47	8	1	3.5	D
24147400	Chicken drumstick, baked, coated, skin / coating eaten	5000_MPE	55	46	9	1	4	B
24133210	Chicken leg, drumstick and thigh, stewed, skin eaten	5000_MPE	55	45	10	1	3.5	B
24152210	Chicken, thigh, roasted, broiled, or baked, skin eaten	5000_MPE	55	45	10	1	3.5	D
32105059	Egg omelet or scrambled egg, with ham or bacon, and dark-green vegetables	5000_MPE	55	45	10	2	3.5	C
32131090	Egg omelet or scrambled egg, with cheese, meat, and vegetables other than dark-green and/or tomatoes, fat added	5000_MPE	55	45	10	2	3	D
32131110	Egg omelet or scrambled egg, with cheese, meat, and vegetables other than dark-green and/or tomatoes, NS as to fat	5000_MPE	55	45	10	2	3	D
24127154	Chicken, breast, coated, baked or fried, prepared skinless, coating eaten, made with cooking spray	5000_MPE	55	44	11	1	4	A
24130210	Chicken leg, drumstick and thigh, NS as to cooking method, skin eaten	5000_MPE	55	44	11	1	4	B
24132240	Chicken leg, drumstick and thigh, rotisserie, skin eaten	5000_MPE	55	44	11	1	4	B
24144221	Chicken, drumstick, fried, no coating, skin not eaten, made with shortening	5000_MPE	55	44	11	1	3.5	C
24154221	Chicken, thigh, fried, no coating, skin not eaten, made with shortening	5000_MPE	55	44	11	1	4	C
32130300	Egg omelet or scrambled egg, with cheese and meat, made with margarine	5000_MPE	55	44	11	2	3	D
32131000	Egg omelet or scrambled egg, with cheese, meat, and tomatoes, fat added	5000_MPE	55	44	11	2	3	D
32130360	Egg omelet or scrambled egg, with cheese and meat, made with cooking spray	5000_MPE	55	43	12	2	3.5	D
32130370	Egg omelet or scrambled egg, with cheese and meat, no added fat	5000_MPE	55	43	12	2	3.5	D
32130880	Egg omelet or scrambled egg, with meat, tomatoes, and dark-green vegetables, NS as to fat	5000_MPE	55	43	12	2	3	C
32130020	Egg omelet or scrambled egg, made with butter	5000_MPE	55	42	13	1	3	D

32130870	Egg omelet or scrambled egg, with meat, tomatoes, and dark-green vegetables, no added fat	5000_MPE	55	41	14	2	3.5	C
33401400	Egg substitute, omelet, scrambled, or fried, with cheese and vegetables	5000_MPE	54	56	-2	3	3.5	C
33001010	Egg substitute, omelet, scrambled, or fried, fat added	5000_MPE	54	55	-1	3	3.5	B
32400500	Egg white, omelet, scrambled, or fried, with cheese and vegetables	5000_MPE	54	53	1	2	3.5	C
24127200	Chicken breast, fried, coated, skin / coating eaten, from raw	5000_MPE	54	48	6	1	4	B
27146010	Chicken or turkey with barbecue sauce, skin not eaten	5000_MPE	54	47	7	2	4	B
24142511	Chicken drumstick, grilled with sauce, skin not eaten	5000_MPE	54	45	9	2	3.5	D
24164000	Chicken wing, grilled without sauce	5000_MPE	54	45	9	1	3.5	D
32105117	Egg omelet or scrambled egg, with sausage, cheese, and dark-green vegetables	5000_MPE	54	45	9	2	3	D
22701020	Pork, spareribs, cooked, lean only eaten	5000_MPE	54	44	10	1	4	C
24153210	Chicken thigh, stewed, skin eaten	5000_MPE	54	44	10	1	3.5	C
32105010	Egg omelet or scrambled egg, with cheese	5000_MPE	54	44	10	2	2	D
32130290	Egg omelet or scrambled egg, with cheese and meat, NS as to fat	5000_MPE	54	44	10	2	3	D
32130365	Egg omelet or scrambled egg, with cheese and meat, NS as to fat type	5000_MPE	54	44	10	2	3	D
24127155	Chicken, breast, coated, baked or fried, prepared skinless, coating eaten, made without fat	5000_MPE	54	43	11	1	4	A
24150210	Chicken thigh, NS as to cooking method, skin eaten	5000_MPE	54	43	11	1	4	B
24152300	Chicken thigh, rotisserie, skin eaten	5000_MPE	54	43	11	1	4	B
32130120	Egg omelet or scrambled egg, with cheese, made with butter	5000_MPE	54	43	11	2	2	D
32130860	Egg omelet or scrambled egg, with meat, tomatoes, and dark-green vegetables, fat added	5000_MPE	54	43	11	2	3	C
24164122	Chicken, wing, fried, no coating, skin not eaten, made with butter	5000_MPE	54	39	15	1	4	C
32400075	Egg white omelet, scrambled, or fried, made with cooking spray	5000_MPE	53	47	6	1	4	A
24127100	Chicken, breast, coated, baked or fried, prepared with skin, NS as to skin/coating eaten, fat added in cooking	5000_MPE	53	45	8	1	3.5	D
24127110	Chicken, breast, coated, baked or fried, prepared with skin, skin/coating eaten, NS as to type of fat added in cooking	5000_MPE	53	45	8	1	3.5	D
24127113	Chicken, breast, coated, baked or fried, prepared with skin, skin/coating eaten, made with oil	5000_MPE	53	45	8	1	3.5	D
24157302	Chicken thigh, fried, coated, prepared skinless, coating eaten, from raw	5000_MPE	53	45	8	1	4	B
23310000	Rabbit, NS as to domestic or wild, cooked	5000_MPE	53	44	9	1	4	C
23311100	Rabbit, domestic, NS as to cooking method	5000_MPE	53	44	9	1	4	C
24123310	Chicken breast, grilled with sauce, skin eaten	5000_MPE	53	44	9	2	3.5	C
24137310	Chicken leg, drumstick and thigh, baked, coated, skin / coating eaten	5000_MPE	53	44	9	1	3.5	C
24157400	Chicken thigh, baked, coated, skin / coating eaten	5000_MPE	53	44	9	1	3.5	C
24164200	Chicken wing, sauteed	5000_MPE	53	44	9	1	3.5	D
32105081	Egg omelet or scrambled egg, with ham or bacon, cheese, and dark-green vegetables	5000_MPE	53	44	9	2	3	D
22201020	Pork steak or cutlet, NS as to cooking method, lean only eaten	5000_MPE	53	42	11	1	4	C

22201120	Pork steak or cutlet, broiled or baked, lean only eaten	5000_MPE	53	42	11	1	4	C
23150100	Goat, boiled	5000_MPE	53	42	11	1	4	C
23150250	Goat, baked	5000_MPE	53	42	11	1	4	C
23150300	Goat ribs, cooked	5000_MPE	53	42	11	1	4	C
24127141	Chicken, breast, coated, baked or fried, prepared skinless, NS as to coating eaten, fat not added in cooking	5000_MPE	53	42	11	1	4	A
24162130	Chicken wing, baked, broiled, or roasted, from raw	5000_MPE	53	42	11	1	3.5	D
23311200	Rabbit, wild, cooked	5000_MPE	53	41	12	1	4.5	C
24122151	Chicken breast, baked or broiled, skin not eaten, from fast food / restaurant	5000_MPE	52	53	-1	4	4	B
33401020	Egg substitute, omelet, scrambled, or fried, with cheese, NS as to fat added in cooking	5000_MPE	52	52	0	3	3	D
24134151	Chicken leg, drumstick and thigh, grilled with sauce, skin not eaten	5000_MPE	52	44	8	2	3.5	D
24147200	Chicken, drumstick, coated, baked or fried, prepared with skin, NS as to skin/coating eaten, fat added in cooking	5000_MPE	52	44	8	1	3.5	D
24147210	Chicken, drumstick, coated, baked or fried, prepared with skin, skin/coating eaten, NS as to type of fat added in cooking	5000_MPE	52	44	8	1	3.5	D
24147213	Chicken, drumstick, coated, baked or fried, prepared with skin, skin/coating eaten, made with oil	5000_MPE	52	44	8	1	3.5	D
24205000	Turkey, tail	5000_MPE	52	44	8	1	2	D
24154021	Chicken thigh, grilled with sauce, skin not eaten	5000_MPE	52	43	9	2	3.5	D
23150200	Goat, fried	5000_MPE	52	42	10	1	4	C
24134210	Chicken, leg (drumstick and thigh), fried, no coating, skin eaten, fat added in cooking	5000_MPE	52	42	10	1	3.5	D
24160100	Chicken, wing, NS as to cooking method, NS as to skin eaten	5000_MPE	52	42	10	1	3.5	D
24162100	Chicken, wing, roasted, broiled, or baked, NS as to skin eaten	5000_MPE	52	42	10	1	3.5	D
24162110	Chicken, wing, roasted, broiled, or baked, skin eaten	5000_MPE	52	42	10	1	3.5	D
22000100	Pork, NS as to cut, cooked, NS as to fat eaten	5000_MPE	52	41	11	1	3.5	D
22000110	Pork, NS as to cut, cooked, lean and fat eaten	5000_MPE	52	41	11	1	3.5	D
22101000	Pork chop, NS as to cooking method, NS as to fat eaten	5000_MPE	52	41	11	1	3.5	D
22101010	Pork chop, NS as to cooking method, lean and fat eaten	5000_MPE	52	41	11	1	3.5	D
22101100	Pork chop, broiled or baked, NS as to fat eaten	5000_MPE	52	41	11	1	3.5	D
22101110	Pork chop, broiled or baked, lean and fat eaten	5000_MPE	52	41	11	1	3.5	D
23326100	Bison, cooked	5000_MPE	52	41	11	1	4.5	B
24170200	Chicken, back	5000_MPE	52	41	11	1	3.5	D
24137251	Chicken, leg (drumstick and thigh), coated, baked or fried, prepared skinless, coating eaten, fat not added in cooking	5000_MPE	52	40	12	1	4	B
24157255	Chicken, thigh, coated, baked or fried, prepared skinless, coating eaten, made without fat	5000_MPE	52	40	12	1	4	B
24400000	Cornish game hen, cooked, NS as to skin eaten	5000_MPE	52	40	12	1	3.5	D
24400010	Cornish game hen, cooked, skin eaten	5000_MPE	52	40	12	1	3.5	D
24401000	Cornish game hen, roasted, NS as to skin eaten	5000_MPE	52	40	12	1	3.5	D
24401010	Cornish game hen, roasted, skin eaten	5000_MPE	52	40	12	1	3.5	D
32130190	Egg omelet or scrambled egg, with meat, NS as to fat	5000_MPE	52	40	12	2	3	C

32130200	Egg omelet or scrambled egg, with meat, made with margarine	5000_MPE	52	40	12	2	3.5	C
32130265	Egg omelet or scrambled egg, with meat, NS as to fat type	5000_MPE	52	40	12	2	3	C
24154222	Chicken, thigh, fried, no coating, skin not eaten, made with butter	5000_MPE	52	39	13	1	3.5	C
32130260	Egg omelet or scrambled egg, with meat, made with cooking spray	5000_MPE	52	39	13	2	3.5	C
31105060	Egg, whole, fried with animal fat or meat drippings	5000_MPE	52	38	14	1	3	C
32130270	Egg omelet or scrambled egg, with meat, no added fat	5000_MPE	52	37	15	2	3.5	C
33000990	Egg substitute, omelet, scrambled, or fried, NS as to fat added in cooking	5000_MPE	51	52	-1	3	3.5	C
33001035	Egg substitute, omelet, scrambled, or fried, NS as to type of fat	5000_MPE	51	52	-1	3	3.5	C
32400050	Egg white omelet or scrambled egg, with cheese	5000_MPE	51	49	2	2	3.5	C
24302010	Duck, pressed, Chinese	5000_MPE	51	47	4	1	3	C
24208000	Turkey, nuggets	5000_MPE	51	46	5	3	4	B
21401400	Beef, roast, canned	5000_MPE	51	45	6	3	4	C
24107000	Chicken, NS as to part, coated, baked or fried, prepared with skin, NS as to skin/coating eaten, fat added in cooking	5000_MPE	51	43	8	1	3.5	D
24107010	Chicken, NS as to part, coated, baked or fried, prepared with skin, skin/coating eaten, fat added in cooking	5000_MPE	51	43	8	1	3.5	D
24147300	Chicken drumstick, fried, coated, skin / coating eaten, from raw	5000_MPE	51	43	8	1	3.5	B
23331100	Ground hog, cooked	5000_MPE	51	41	10	1	4	C
24162200	Chicken wing, rotisserie	5000_MPE	51	41	10	1	2	D
21000100	Beef, NS as to cut, cooked, NS as to fat eaten	5000_MPE	51	40	11	1	4	C
21000120	Beef, NS as to cut, cooked, lean only eaten	5000_MPE	51	40	11	1	4	C
21001000	Steak, NS as to type of meat, cooked, NS as to fat eaten	5000_MPE	51	40	11	1	4	C
21001020	Steak, NS as to type of meat, cooked, lean only eaten	5000_MPE	51	40	11	1	4	C
21101000	Beef steak, NS as to cooking method, NS as to fat eaten	5000_MPE	51	40	11	1	4	C
21101020	Beef steak, NS as to cooking method, lean only eaten	5000_MPE	51	40	11	1	4	C
21101110	Beef steak, broiled or baked, NS as to fat eaten	5000_MPE	51	40	11	1	4	C
21101130	Beef steak, broiled or baked, lean only eaten	5000_MPE	51	40	11	1	4	C
21401110	Beef, roast, roasted, lean and fat eaten	5000_MPE	51	40	11	1	4	C
22210300	Pork, tenderloin, cooked, NS as to cooking method	5000_MPE	51	40	11	1	4	C
22210400	Pork, tenderloin, baked	5000_MPE	51	40	11	1	4	C
22301120	Ham, fresh, cooked, lean only eaten	5000_MPE	51	40	11	1	4	C
24160110	Chicken wing, NS as to cooking method	5000_MPE	51	40	11	1	2	D
23321000	Venison/deer, NFS	5000_MPE	51	39	12	1	4.5	C
23321100	Venison/deer, roasted	5000_MPE	51	39	12	1	4.5	C
23322350	Venison/deer ribs, cooked	5000_MPE	51	39	12	1	4.5	C
23322400	Venison/deer, stewed	5000_MPE	51	39	12	1	4.5	C
23323100	Moose, cooked	5000_MPE	51	39	12	1	4.5	B
32101000	Egg, creamed	5000_MPE	51	39	12	1	3	C
24198500	Chicken feet	5000_MPE	51	38	13	1	4	A

32130340	Egg omelet or scrambled egg, with cheese and meat, made with animal fat or meat drippings	5000_MPE	51	38	13	2	3	D
24144222	Chicken, drumstick, fried, no coating, skin not eaten, made with butter	5000_MPE	51	37	14	1	3.5	D
32400055	Egg white omelet, scrambled, or fried, NS as to fat	5000_MPE	50	47	3	2	3.5	B
32400078	Egg white omelet, scrambled, or fried, NS as to fat type	5000_MPE	50	47	3	2	3.5	B
21500000	Ground beef, raw	5000_MPE	50	41	9	1	3.5	C
24103075	Chicken, NS as to part, grilled with sauce, skin eaten	5000_MPE	50	41	9	2	3.5	D
24167300	Chicken wing, baked, coated	5000_MPE	50	41	9	1	3.5	D
24301210	Duck, coated, fried	5000_MPE	50	40	10	1	4	B
20000000	Meat, NFS	5000_MPE	50	39	11	1	4	C
22400120	Pork roast, NS as to cut, cooked, lean only eaten	5000_MPE	50	39	11	1	4	C
22401020	Pork roast, loin, cooked, lean only eaten	5000_MPE	50	39	11	1	4	C
24164100	Chicken, wing, fried, no coating, NS as to skin eaten, fat added in cooking	5000_MPE	50	39	11	1	3.5	D
24164110	Chicken, wing, fried, no coating, skin eaten, NS as to type of fat added in cooking	5000_MPE	50	39	11	1	3.5	D
24164113	Chicken, wing, fried, no coating, skin eaten, made with oil	5000_MPE	50	39	11	1	3.5	D
32105118	Egg omelet or scrambled egg, with sausage and vegetables other than dark-green	5000_MPE	50	39	11	2	3.5	C
22000120	Pork, NS as to cut, cooked, lean only eaten	5000_MPE	50	38	12	1	4	C
22101020	Pork chop, NS as to cooking method, lean only eaten	5000_MPE	50	38	12	1	4	C
22101120	Pork chop, broiled or baked, lean only eaten	5000_MPE	50	38	12	1	4	C
23204030	Veal cutlet or steak, NS as to cooking method, lean only eaten	5000_MPE	50	38	12	1	4	C
23204220	Veal cutlet or steak, broiled, lean only eaten	5000_MPE	50	38	12	1	4	C
24124122	Chicken, breast, fried, no coating, skin not eaten, made with butter	5000_MPE	50	38	12	1	4	C
24163110	Chicken wing, stewed	5000_MPE	50	38	12	1	3.5	D
24144212	Chicken, drumstick, fried, no coating, skin eaten, made with butter	5000_MPE	50	37	13	1	3.5	D
24163100	Chicken, wing, stewed, NS as to skin eaten	5000_MPE	50	37	13	1	3.5	D
31105040	Egg, whole, fried with butter	5000_MPE	50	36	14	1	3	D
41810250	bacon bits, meatless	5000_MPE	49	56	-7	4	1.5	E
32400120	Egg white, omelet, scrambled, or fried, with cheese, NS as to fat added in cooking	5000_MPE	49	47	2	2	3	D
22210310	Pork, tenderloin, breaded, fried	5000_MPE	49	46	3	2	3.5	D
24127151	Chicken, breast, coated, baked or fried, prepared skinless, coating eaten, made with shortening	5000_MPE	49	41	8	2	4	C
24137300	Chicken leg, drumstick and thigh, fried, coated, skin / coating eaten	5000_MPE	49	41	8	1	3.5	C
24157300	Chicken thigh, fried, coated, skin / coating eaten, from raw	5000_MPE	49	41	8	1	3.5	D
31108010	Egg, white only, raw	5000_MPE	49	41	8	1	4	A
24137200	Chicken, leg (drumstick and thigh), coated, baked or fried, prepared with skin, NS as to skin/coating eaten, fat added in cooking	5000_MPE	49	40	9	1	3.5	D
24137210	Chicken, leg (drumstick and thigh), coated, baked or fried, prepared with skin, skin/coating eaten, fat added in cooking	5000_MPE	49	40	9	1	3.5	D
24142510	Chicken drumstick, grilled with sauce, skin eaten	5000_MPE	49	40	9	2	3.5	D

22201320	Pork steak or cutlet, breaded or floured, broiled or baked, lean only eaten	5000_MPE	49	39	10	1	3.5	C
23108020	Lamb, shoulder, cooked, lean only eaten	5000_MPE	49	39	10	1	4	C
24134150	Chicken leg, drumstick and thigh, grilled with sauce, skin eaten	5000_MPE	49	39	10	2	3	D
21401000	Beef, roast, roasted, NS as to fat eaten	5000_MPE	49	38	11	1	4	C
22210350	Pork, tenderloin, braised	5000_MPE	49	37	12	1	4	C
23200120	Veal, NS as to cut, cooked, lean only eaten	5000_MPE	49	37	12	1	4	C
24107001	Chicken, NS as to part, coated, baked or fried, prepared with skin, NS as to skin/coating eaten, fat not added in cooking	5000_MPE	49	37	12	1	3.5	D
24147215	Chicken, drumstick, coated, baked or fried, prepared with skin, skin/coating eaten, made without fat	5000_MPE	49	37	12	1	4	C
24164115	Chicken, wing, fried, no coating, skin eaten, made without fat	5000_MPE	49	37	12	1	3.5	D
24180200	Chicken, neck or ribs	5000_MPE	49	37	12	1	3.5	D
25170110	Tripe, cooked	5000_MPE	49	35	14	1	3.5	C
24198695	Chicken patty, fillet, or tenders, breaded, cooked, from school lunch	5000_MPE	48	52	-4	4	3.5	C
22101130	Pork chop, breaded or floured, broiled or baked, NS as to fat eaten	5000_MPE	48	41	7	2	3.5	D
22101140	Pork chop, breaded or floured, broiled or baked, lean and fat eaten	5000_MPE	48	41	7	2	3.5	D
22101150	Pork chop, breaded or floured, broiled or baked, lean only eaten	5000_MPE	48	40	8	2	4	C
24167113	Chicken, wing, coated, baked or fried, prepared with skin, skin/coating eaten, made with oil	5000_MPE	48	40	8	1	3.5	D
24167100	Chicken, wing, coated, baked or fried, prepared with skin, NS as to skin/coating eaten, fat added in cooking	5000_MPE	48	39	9	1	3.5	D
24167110	Chicken, wing, coated, baked or fried, prepared with skin, skin/coating eaten, NS as to type of fat added in cooking	5000_MPE	48	39	9	1	3.5	D
22201000	Pork steak or cutlet, NS as to cooking method, NS as to fat eaten	5000_MPE	48	38	10	1	3.5	D
22201010	Pork steak or cutlet, NS as to cooking method, lean and fat eaten	5000_MPE	48	38	10	1	3.5	D
22201100	Pork steak or cutlet, broiled or baked, NS as to fat eaten	5000_MPE	48	38	10	1	3.5	D
22201110	Pork steak or cutlet, broiled or baked, lean and fat eaten	5000_MPE	48	38	10	1	3.5	D
22400100	Pork roast, NS as to cut, cooked, NS as to fat eaten	5000_MPE	48	38	10	1	3.5	D
22400110	Pork roast, NS as to cut, cooked, lean and fat eaten	5000_MPE	48	38	10	1	3.5	D
22401000	Pork roast, loin, cooked, NS as to fat eaten	5000_MPE	48	38	10	1	3.5	D
22401010	Pork roast, loin, cooked, lean and fat eaten	5000_MPE	48	38	10	1	3.5	D
22705010	Pork ears, tail, head, snout, miscellaneous parts, cooked	5000_MPE	48	38	10	1	3.5	C
24154020	Chicken thigh, grilled with sauce, skin eaten	5000_MPE	48	38	10	1	3	D
32105119	Egg omelet or scrambled egg, with sausage, cheese, and vegetables other than dark-green	5000_MPE	48	38	10	2	3	D
21000110	Beef, NS as to cut, cooked, lean and fat eaten	5000_MPE	48	37	11	1	3.5	D
21101010	Beef steak, NS as to cooking method, lean and fat eaten	5000_MPE	48	37	11	1	3.5	D
21101120	Beef steak, broiled or baked, lean and fat eaten	5000_MPE	48	37	11	1	3.5	D
22411020	Pork roast, shoulder, cooked, lean only eaten	5000_MPE	48	37	11	1	3.5	C
32105082	Egg omelet or scrambled egg, with ham or bacon, cheese, and vegetables other than dark-green	5000_MPE	48	37	11	2	3	D
22201220	Pork steak or cutlet, fried, lean only eaten	5000_MPE	48	36	12	1	4	C

23101020	Lamb chop, NS as to cut, cooked, lean only eaten	5000_MPE	48	36	12	1	4	C
23104020	Lamb, loin chop, cooked, lean only eaten	5000_MPE	48	36	12	1	4	C
23321200	Venison/deer steak, cooked, NS as to cooking method	5000_MPE	48	36	12	1	4	C
23340100	Armadillo, cooked	5000_MPE	48	36	12	1	4.5	B
32105060	Egg omelet or scrambled egg, with ham or bacon and vegetables other than dark-green	5000_MPE	48	36	12	2	3.5	C
32130320	Egg omelet or scrambled egg, with cheese and meat, made with butter	5000_MPE	48	36	12	2	2	D
21401120	Beef, roast, roasted, lean only eaten	5000_MPE	48	35	13	1	4	C
33401000	Egg substitute, omelet, scrambled, or fried, with cheese	5000_MPE	47	48	-1	3	3.5	C
33401500	Egg substitute, omelet, scrambled, or fried, with meat and vegetables	5000_MPE	47	46	1	3	3.5	C
24300100	Duck, cooked, NS as to skin eaten	5000_MPE	47	39	8	1	2	D
24300110	Duck, cooked, skin eaten	5000_MPE	47	39	8	1	2	D
24301010	Duck, roasted, skin eaten	5000_MPE	47	39	8	1	2	D
21540100	Ground beef with textured vegetable protein, cooked	5000_MPE	47	38	9	1	3.5	D
22708010	Pork, pig's hocks, cooked	5000_MPE	47	37	10	1	3.5	D
24154210	Chicken, thigh, fried, no coating, skin eaten, NS as to type of fat added in cooking	5000_MPE	47	37	10	1	2	D
24154213	Chicken, thigh, fried, no coating, skin eaten, made with oil	5000_MPE	47	37	10	1	2	D
21001010	Steak, NS as to type of meat, cooked, lean and fat eaten	5000_MPE	47	36	11	1	3.5	D
22101500	Pork chop, stewed, NS as to fat eaten	5000_MPE	47	36	11	1	3.5	D
22101510	Pork chop, stewed, lean and fat eaten	5000_MPE	47	36	11	1	3.5	D
24154214	Chicken, thigh, fried, no coating, skin eaten, made with cooking spray	5000_MPE	47	36	11	1	2	D
25160000	Tongue, cooked	5000_MPE	47	36	11	1	2	D
32105123	Egg omelet or scrambled egg, with sausage, cheese, and mushrooms	5000_MPE	47	36	11	2	3	D
23204010	Veal cutlet or steak, NS as to cooking method, NS as to fat eaten	5000_MPE	47	35	12	1	4	C
32105085	Egg omelet or scrambled egg, with ham or bacon, cheese, and tomatoes	5000_MPE	47	35	12	2	2	D
23204020	Veal cutlet or steak, NS as to cooking method, lean and fat eaten	5000_MPE	47	34	13	1	4	C
33301010	Scrambled egg, made from packaged liquid mixture	5000_MPE	46	48	-2	4	3.5	B
32400100	Egg white, omelet, scrambled, or fried, with cheese	5000_MPE	46	43	3	2	3.5	C
32400600	Egg white, omelet, scrambled, or fried, with meat and vegetables	5000_MPE	46	41	5	2	3.5	B
21420100	Beef, sandwich steak, flaked, formed, thinly sliced	5000_MPE	46	38	8	1	2	D
22201300	Pork steak or cutlet, breaded or floured, broiled or baked, NS as to fat eaten	5000_MPE	46	37	9	1	2	D
22201310	Pork steak or cutlet, breaded or floured, broiled or baked, lean and fat eaten	5000_MPE	46	37	9	1	2	D
24164010	Chicken wing, grilled with sauce	5000_MPE	46	37	9	1	2	D
22301000	Ham, fresh, cooked, NS as to fat eaten	5000_MPE	46	36	10	1	2	D
22301110	Ham, fresh, cooked, lean and fat eaten	5000_MPE	46	36	10	1	2	D
23110000	Lamb, ribs, cooked, lean only eaten	5000_MPE	46	36	10	1	3.5	D

32105161	Egg omelet or scrambled egg, with chorizo and cheese	5000_MPE	46	36	10	2	1.5	D
21410110	Beef, stew meat, cooked, lean and fat eaten	5000_MPE	46	35	11	1	4	C
21417100	Beef brisket, cooked, NS as to fat eaten	5000_MPE	46	35	11	1	4	C
21417120	Beef brisket, cooked, lean only eaten	5000_MPE	46	35	11	1	4	C
22000200	Pork, NS as to cut, fried, NS as to fat eaten	5000_MPE	46	35	11	1	3.5	D
22000210	Pork, NS as to cut, fried, lean and fat eaten	5000_MPE	46	35	11	1	3.5	D
22101200	Pork chop, fried, NS as to fat eaten	5000_MPE	46	35	11	1	3.5	D
22101210	Pork chop, fried, lean and fat eaten	5000_MPE	46	35	11	1	3.5	D
23120120	Lamb, roast, cooked, lean only eaten	5000_MPE	46	35	11	1	4	C
24154215	Chicken, thigh, fried, no coating, skin eaten, made without fat	5000_MPE	46	35	11	1	2	D
24137211	Chicken, leg (drumstick and thigh), coated, baked or fried, prepared with skin, skin/coating eaten, fat not added in cooking	5000_MPE	46	34	12	1	3.5	D
24164111	Chicken, wing, fried, no coating, skin eaten, made with shortening	5000_MPE	46	34	12	1	3.5	D
32130240	Egg omelet or scrambled egg, with meat, made with animal fat or meat drippings	5000_MPE	46	32	14	2	3	D
33000100	Egg substitute, NS as to powdered, frozen, or liquid	5000_MPE	45	50	-5	4	4	A
33201010	Scrambled egg, made from cholesterol-free frozen mixture	5000_MPE	45	48	-3	4	3.5	B
24142321	Chicken drumstick, baked or broiled, skin not eaten, from fast food / restaurant	5000_MPE	45	44	1	4	4	B
21500200	Ground beef or patty, breaded, cooked	5000_MPE	45	41	4	2	2	D
22002100	Pork, ground or patty, breaded, cooked	5000_MPE	45	40	5	2	2	D
22000300	Pork, NS as to cut, breaded or floured, fried, NS as to fat eaten	5000_MPE	45	39	6	2	3.5	D
22000320	Pork, NS as to cut, breaded or floured, fried, lean only eaten	5000_MPE	45	39	6	2	3.5	D
22101320	Pork chop, breaded or floured, fried, lean only eaten	5000_MPE	45	39	6	2	3.5	D
22210450	Pork, tenderloin, battered, fried	5000_MPE	45	37	8	2	4	C
24167200	Chicken wing, fried, coated, from raw	5000_MPE	45	37	8	1	3.5	D
27146000	Chicken or turkey with barbecue sauce, skin eaten	5000_MPE	45	37	8	2	3.5	D
24147251	Chicken, drumstick, coated, baked or fried, prepared skinless, coating eaten, made with shortening	5000_MPE	45	36	9	2	3.5	D
24157200	Chicken, thigh, coated, baked or fried, prepared with skin, NS as to skin/coating eaten, fat added in cooking	5000_MPE	45	36	9	1	2	D
24157210	Chicken, thigh, coated, baked or fried, prepared with skin, skin/coating eaten, NS as to type of fat added in cooking	5000_MPE	45	36	9	1	2	D
24157213	Chicken, thigh, coated, baked or fried, prepared with skin, skin/coating eaten, made with oil	5000_MPE	45	36	9	1	2	D
23108000	Lamb, shoulder, cooked, NS as to fat eaten	5000_MPE	45	35	10	1	2	D
23108010	Lamb, shoulder, cooked, lean and fat eaten	5000_MPE	45	35	10	1	2	D
23107020	Lamb, shoulder chop, cooked, lean only eaten	5000_MPE	45	34	11	1	3.5	C
24154211	Chicken, thigh, fried, no coating, skin eaten, made with shortening	5000_MPE	45	34	11	1	2	D
21003000	Beef, NS as to cut, fried, NS to fat eaten	5000_MPE	45	33	12	1	4	C
21102110	Beef steak, fried, NS as to fat eaten	5000_MPE	45	33	12	1	4	C
21102130	Beef steak, fried, lean only eaten	5000_MPE	45	33	12	1	4	C
21105110	Beef steak, braised, NS as to fat eaten	5000_MPE	45	33	12	1	3.5	D

21105120	Beef steak, braised, lean and fat eaten	5000_MPE	45	33	12	1	3.5	D
21301000	Beef, oxtails, cooked	5000_MPE	45	33	12	1	3.5	D
21407000	Beef, pot roast, braised or boiled, NS as to fat eaten	5000_MPE	45	33	12	1	3.5	D
21407120	Beef, pot roast, braised or boiled, lean only eaten	5000_MPE	45	33	12	1	3.5	D
21410000	Beef, stew meat, cooked, NS as to fat eaten	5000_MPE	45	33	12	1	3.5	D
21410120	Beef, stew meat, cooked, lean only eaten	5000_MPE	45	33	12	1	3.5	D
21501300	Ground beef, 85% - 89% lean, cooked (formerly extra lean)	5000_MPE	45	33	12	1	3.5	D
21501350	Ground beef, 90% - 94% lean, cooked	5000_MPE	45	33	12	1	3.5	C
23200100	Veal, NS as to cut, cooked, NS as to fat eaten	5000_MPE	45	33	12	1	4	D
23200110	Veal, NS as to cut, cooked, lean and fat eaten	5000_MPE	45	33	12	1	4	D
23210010	Veal, roasted, NS as to fat eaten	5000_MPE	45	33	12	1	4	D
23210020	Veal, roasted, lean and fat eaten	5000_MPE	45	33	12	1	4	D
21501360	Ground beef, 95% or more lean, cooked	5000_MPE	45	32	13	1	4	C
22101520	Pork chop, stewed, lean only eaten	5000_MPE	45	32	13	1	4	C
23205030	Veal cutlet or steak, fried, lean only eaten	5000_MPE	45	32	13	1	4	C
24167115	Chicken, wing, coated, baked or fried, prepared with skin, skin/coating eaten, made without fat	5000_MPE	45	32	13	1	3.5	D
24164112	Chicken, wing, fried, no coating, skin eaten, made with butter	5000_MPE	45	31	14	1	2	D
24168022	Chicken "wings", plain, from other sources	5000_MPE	44	39	5	3	2	D
22201420	Pork steak or cutlet, breaded or floured, fried, lean only eaten	5000_MPE	44	38	6	2	3.5	D
32105126	Egg omelet or scrambled egg, with hot dog and cheese	5000_MPE	44	34	10	2	2	D
21304000	Beef, shortribs, cooked, NS as to fat eaten	5000_MPE	44	33	11	1	2	D
21304120	Beef, shortribs, cooked, lean only eaten	5000_MPE	44	33	11	1	2	D
21305000	Beef, cow head, cooked	5000_MPE	44	33	11	1	2	D
21500310	Ground beef patty, cooked	5000_MPE	44	33	11	1	2	D
22002000	Pork, ground or patty, cooked	5000_MPE	44	33	11	1	2	D
22411000	Pork roast, shoulder, cooked, NS as to fat eaten	5000_MPE	44	33	11	1	2	D
22411010	Pork roast, shoulder, cooked, lean and fat eaten	5000_MPE	44	33	11	1	2	D
24127152	Chicken, breast, coated, baked or fried, prepared skinless, coating eaten, made with butter	5000_MPE	44	33	11	1	3.5	D
21501200	Ground beef, 80% - 84% lean, cooked (formerly lean)	5000_MPE	44	32	12	1	3.5	D
24157215	Chicken, thigh, coated, baked or fried, prepared with skin, skin/coating eaten, made without fat	5000_MPE	44	32	12	1	3.5	D
32105120	Egg omelet or scrambled egg, with sausage and mushrooms	5000_MPE	44	32	12	2	3	C
32105122	Egg omelet or scrambled egg, with sausage	5000_MPE	44	32	12	2	3	C
22000220	Pork, NS as to cut, fried, lean only eaten	5000_MPE	44	31	13	1	4	C
22101220	Pork chop, fried, lean only eaten	5000_MPE	44	31	13	1	4	C
23201030	Veal chop, NS as to cooking method, lean only eaten	5000_MPE	44	31	13	1	4	C
23203120	Veal chop, broiled, lean only eaten	5000_MPE	44	31	13	1	4	C
23210030	Veal, roasted, lean only eaten	5000_MPE	44	31	13	1	4	C
23220010	Veal, ground or patty, cooked	5000_MPE	44	31	13	1	4	C

23204200	Veal cutlet or steak, broiled, NS as to fat eaten	5000_MPE	44	30	14	1	4	C
23204210	Veal cutlet or steak, broiled, lean and fat eaten	5000_MPE	44	30	14	1	4	C
32130220	Egg omelet or scrambled egg, with meat, made with butter	5000_MPE	44	30	14	2	3	D
24157331	Chicken thigh, fried, coated, skin / coating not eaten, from restaurant	5000_MPE	43	39	4	3	3.5	D
24204000	Turkey, rolled roast, light or dark meat, cooked	5000_MPE	43	39	4	4	3.5	C
32400700	Egg white, omelet, scrambled, or fried, with cheese, meat, and vegetables	5000_MPE	43	39	4	2	3.5	C
22000310	Pork, NS as to cut, breaded or floured, fried, lean and fat eaten	5000_MPE	43	37	6	2	3.5	D
22101300	Pork chop, breaded or floured, fried, NS as to fat eaten	5000_MPE	43	37	6	2	3.5	D
22101310	Pork chop, breaded or floured, fried, lean and fat eaten	5000_MPE	43	37	6	2	3.5	D
21103110	Beef steak, breaded or floured, baked or fried, NS as to fat eaten	5000_MPE	43	36	7	2	3.5	D
21103130	Beef steak, breaded or floured, baked or fried, lean only eaten	5000_MPE	43	36	7	2	3.5	D
14630200	Cheese souffle	5000_MPE	43	33	10	2	2	D
22701000	Pork, spareribs, cooked, NS as to fat eaten	5000_MPE	43	33	10	1	1.5	E
22701010	Pork, spareribs, cooked, lean and fat eaten	5000_MPE	43	33	10	1	1.5	E
23132000	Lamb, ground or patty, cooked	5000_MPE	43	33	10	1	2	D
31108110	Egg, white, cooked, no added fat	5000_MPE	43	33	10	1	4	A
20000200	Ground meat, NFS	5000_MPE	43	32	11	1	2	D
21302000	Beef, neck bones, cooked	5000_MPE	43	32	11	1	2	D
21500100	Ground beef, cooked	5000_MPE	43	32	11	1	2	D
21500110	Ground beef, meatballs, meat only, cooked, NS as to percent lean (formerly NS as to regular, lean, or extra lean)	5000_MPE	43	32	11	1	2	D
23111010	Lamb hocks, cooked	5000_MPE	43	32	11	1	3.5	D
23203030	Veal chop, fried, lean only eaten	5000_MPE	43	32	11	1	4	C
32105121	Egg omelet or scrambled egg, with sausage and cheese	5000_MPE	43	32	11	3	2	D
21102120	Beef steak, fried, lean and fat eaten	5000_MPE	43	31	12	1	3.5	D
21501000	Ground beef, less than 80% lean, cooked (formerly regular)	5000_MPE	43	31	12	1	2	D
22201200	Pork steak or cutlet, fried, NS as to fat eaten	5000_MPE	43	31	12	1	3.5	D
22201210	Pork steak or cutlet, fried, lean and fat eaten	5000_MPE	43	31	12	1	3.5	D
24154212	Chicken, thigh, fried, no coating, skin eaten, made with butter	5000_MPE	43	31	12	1	2	D
32105080	Egg omelet or scrambled egg, with ham or bacon and cheese	5000_MPE	43	31	12	2	2	D
21105130	Beef steak, braised, lean only eaten	5000_MPE	43	30	13	1	4	C
21407110	Beef, pot roast, braised or boiled, lean and fat eaten	5000_MPE	43	30	13	1	4	C
24122150	Chicken breast, baked or broiled, skin eaten, from fast food / restaurant	5000_MPE	42	42	0	4	4	B
24127211	Chicken breast, fried, coated, skin / coating not eaten, from pre-cooked	5000_MPE	42	42	0	4	4	A
33401600	Egg substitute, omelet, scrambled, or fried, with cheese, meat, and vegetables	5000_MPE	42	41	1	3	3.5	C
23220030	Veal patty, breaded, cooked	5000_MPE	42	35	7	2	3.5	D
23000100	Lamb, NS as to cut, cooked	5000_MPE	42	32	10	1	2	D

23120100	Lamb, roast, cooked, NS as to fat eaten	5000_MPE	42	32	10	1	2	D
23120110	Lamb, roast, cooked, lean and fat eaten	5000_MPE	42	32	10	1	2	D
31109010	Egg, white only, cooked, fat not added in cooking	5000_MPE	42	32	10	1	4	A
32400011	Egg white omelet or scrambled egg, fat not added in cooking	5000_MPE	42	32	10	1	4	A
21417110	Beef brisket, cooked, lean and fat eaten	5000_MPE	42	31	11	1	2	D
24127112	Chicken, breast, coated, baked or fried, prepared with skin, skin/coating eaten, made with butter	5000_MPE	42	31	11	1	2	D
24198440	Chicken skin	5000_MPE	42	31	11	1	1.5	E
24147252	Chicken, drumstick, coated, baked or fried, prepared skinless, coating eaten, made with butter	5000_MPE	42	30	12	1	3.5	D
25170310	Hog maws, cooked	5000_MPE	42	28	14	1	2	D
33201110	Scrambled egg, made from cholesterol-free frozen mixture with cheese	5000_MPE	41	44	-3	4	3.5	C
24122141	Chicken breast, baked or broiled, skin not eaten, from pre-cooked	5000_MPE	41	40	1	4	4.5	A
24152251	Chicken thigh, baked or broiled, skin not eaten, from fast food / restaurant	5000_MPE	41	40	1	4	4	C
24142311	Chicken drumstick, baked or broiled, skin not eaten, from pre-cooked	5000_MPE	41	39	2	4	4	B
21103120	Beef steak, breaded or floured, baked or fried, lean and fat eaten	5000_MPE	41	34	7	2	3.5	D
22201410	Pork steak or cutlet, breaded or floured, fried, lean and fat eaten	5000_MPE	41	34	7	2	2	D
22704010	Pork, cracklings, cooked	5000_MPE	41	34	7	3	0.5	E
22201070	Pork steak or cutlet, battered, fried, lean only eaten	5000_MPE	41	33	8	2	3.5	C
23101000	Lamb chop, NS as to cut, cooked, NS as to fat eaten	5000_MPE	41	32	9	1	1.5	D
23101010	Lamb chop, NS as to cut, cooked, lean and fat eaten	5000_MPE	41	32	9	1	1.5	D
23104000	Lamb, loin chop, cooked, NS as to fat eaten	5000_MPE	41	32	9	1	1.5	D
23104010	Lamb, loin chop, cooked, lean and fat eaten	5000_MPE	41	32	9	1	1.5	D
23107000	Lamb, shoulder chop, cooked, NS as to fat eaten	5000_MPE	41	31	10	1	2	D
23107010	Lamb, shoulder chop, cooked, lean and fat eaten	5000_MPE	41	31	10	1	2	D
23203010	Veal chop, fried, NS as to fat eaten	5000_MPE	41	30	11	1	3.5	C
23203020	Veal chop, fried, lean and fat eaten	5000_MPE	41	30	11	1	3.5	C
23205010	Veal cutlet or steak, fried, NS as to fat eaten	5000_MPE	41	30	11	1	3.5	C
23205020	Veal cutlet or steak, fried, lean and fat eaten	5000_MPE	41	30	11	1	3.5	C
23201010	Veal chop, NS as to cooking method, NS as to fat eaten	5000_MPE	41	29	12	1	3.5	D
23201020	Veal chop, NS as to cooking method, lean and fat eaten	5000_MPE	41	29	12	1	3.5	D
23203100	Veal chop, broiled, NS as to fat eaten	5000_MPE	41	29	12	1	3.5	D
23203110	Veal chop, broiled, lean and fat eaten	5000_MPE	41	29	12	1	3.5	D
25160110	Tongue, smoked, cured, or pickled, cooked	5000_MPE	41	29	12	1	1	E
33001000	Egg substitute, omelet, scrambled, or fried, made with margarine	5000_MPE	40	45	-5	4	3.5	C
24198735	Chicken nuggets, from school lunch	5000_MPE	40	43	-3	4	3.5	D
24198745	Chicken tenders or strips, breaded, from school lunch	5000_MPE	40	43	-3	4	3.5	D
24157235	Chicken, thigh, from fast food, coated, baked or broiled, prepared with skin, skin/coating not eaten	5000_MPE	40	38	2	4	3.5	D

24147311	Chicken drumstick, fried, coated, skin / coating not eaten, from pre-cooked	5000_MPE	40	37	3	4	4	B
24168021	Chicken "wings" with other sauces or seasoning, from other sources	5000_MPE	40	33	7	2	1.5	D
22701050	Pork, spareribs, barbecued, with sauce, lean only eaten	5000_MPE	40	32	8	2	3.5	D
23311120	Rabbit, NS as to domestic or wild, breaded, fried	5000_MPE	40	31	9	2	4	C
32105330	Scrambled eggs with jerked beef, Puerto Rican style	5000_MPE	40	30	10	3	1	E
21104110	Beef steak, battered, fried, NS as to fat eaten	5000_MPE	40	28	12	2	4	C
24147212	Chicken, drumstick, coated, baked or fried, prepared with skin, skin/coating eaten, made with butter	5000_MPE	40	28	12	1	2	D
24142320	Chicken drumstick, baked or broiled, skin eaten, from fast food / restaurant	5000_MPE	39	38	1	4	3.5	C
24122140	Chicken breast, baked or broiled, skin eaten, from pre-cooked	5000_MPE	39	37	2	4	4	A
32400400	Egg white, omelet, scrambled, or fried, with cheese and meat	5000_MPE	39	34	5	2	3.5	C
22101420	Pork chop, battered, fried, lean only eaten	5000_MPE	39	31	8	2	3.5	C
22201400	Pork steak or cutlet, breaded or floured, fried, NS as to fat eaten	5000_MPE	39	31	8	2	2	D
23110010	Lamb, ribs, cooked, NS as to fat eaten	5000_MPE	39	30	9	1	1.5	D
23110050	Lamb, ribs, cooked, lean and fat eaten	5000_MPE	39	30	9	1	1.5	D
23220020	Mock chicken legs, cooked	5000_MPE	39	29	10	2	3.5	D
24127221	Chicken breast, fried, coated, skin / coating not eaten, from fast food / restaurant	5000_MPE	38	40	-2	4	4	B
24147235	Chicken, drumstick, from fast food, coated, baked or fried, prepared with skin, skin/coating not eaten	5000_MPE	38	36	2	4	4	C
22101400	Pork chop, battered, fried, NS as to fat eaten	5000_MPE	38	30	8	2	3.5	D
22101410	Pork chop, battered, fried, lean and fat eaten	5000_MPE	38	30	8	2	3.5	D
22201050	Pork steak or cutlet, battered, fried, NS as to fat eaten	5000_MPE	38	30	8	1	3	D
22201060	Pork steak or cutlet, battered, fried, lean and fat eaten	5000_MPE	38	30	8	1	3	D
21304220	Beef, shortribs, barbecued, with sauce, lean only eaten	5000_MPE	38	28	10	2	2	D
22701030	Pork, spareribs, barbecued, with sauce, NS as to fat eaten	5000_MPE	38	28	10	1	1.5	E
22701040	Pork, spareribs, barbecued, with sauce, lean and fat eaten	5000_MPE	38	28	10	1	1.5	E
24198840	Fried chicken chunks, Puerto Rican style	5000_MPE	38	27	11	1	2	D
32400080	Egg white omelet, scrambled, or fried, no added fat	5000_MPE	38	27	11	1	4	A
21104130	Beef steak, battered, fried, lean only eaten	5000_MPE	38	26	12	2	3.5	C
24198710	Chicken patty with cheese, breaded, cooked	5000_MPE	37	39	-2	4	2	D
24157311	Chicken thigh, fried, coated, skin / coating not eaten, from pre-cooked	5000_MPE	37	36	1	4	4	B
33401300	Egg substitute, omelet, scrambled, or fried, with cheese and meat	5000_MPE	37	36	1	3	3	C
24107071	Chicken, NS as to part, fried, coated, skin / coating not eaten	5000_MPE	37	35	2	4	4	B
24127135	Chicken, breast, from fast food, coated, baked or fried, prepared with skin, skin/coating not eaten	5000_MPE	37	35	2	4	4	B
24152241	Chicken thigh, baked or broiled, skin not eaten, from pre-cooked	5000_MPE	37	35	2	4	4	B
24142310	Chicken drumstick, baked or broiled, skin eaten, from pre-cooked	5000_MPE	37	34	3	4	4	B
24168020	Chicken "wings" with hot sauce, from other sources	5000_MPE	37	30	7	2	1.5	E
21304110	Beef, shortribs, cooked, lean and fat eaten	5000_MPE	37	27	10	1	1	E

21104120	Beef steak, battered, fried, lean and fat eaten	5000_MPE	37	25	12	2	3.5	D
32105030	Egg omelet or scrambled egg, with ham or bacon	5000_MPE	37	23	14	2	3	D
24198677	Chicken fillet, breaded	5000_MPE	36	39	-3	4	3.5	C
24198700	Chicken patty, fillet, or tenders, breaded, cooked	5000_MPE	36	39	-3	4	3.5	C
24198746	Chicken tenders or strips, breaded, from frozen	5000_MPE	36	39	-3	4	3.5	C
24198742	Chicken tenders or strips, breaded, from restaurant	5000_MPE	36	37	-1	4	2	D
33001050	Egg substitute, omelet, scrambled, or fried, no added fat	5000_MPE	36	37	-1	4	4	A
33102010	Scrambled egg, made from powdered mixture	5000_MPE	36	37	-1	4	3.5	C
33202010	Scrambled egg, made from frozen mixture	5000_MPE	36	33	3	4	3.5	B
33401010	Egg substitute, omelet, scrambled, or fried, with cheese, fat not added in cooking	5000_MPE	36	33	3	3	3	D
32400110	Egg white, omelet, scrambled, or fried, with cheese, fat not added in cooking	5000_MPE	36	28	8	2	3.5	C
24198747	Chicken tenders or strips, breaded, from other sources	5000_MPE	35	38	-3	4	3.5	D
24198671	Chicken patty, breaded	5000_MPE	35	37	-2	4	3	D
24198690	Chicken patty, fillet, or tenders, breaded, cooked, from fast food / restaurant	5000_MPE	35	37	-2	4	2	D
24198739	Chicken tenders or strips, NFS	5000_MPE	35	37	-2	4	2	D
24198741	Chicken tenders or strips, breaded, from fast food	5000_MPE	35	37	-2	4	2	D
33001040	Egg substitute, omelet, scrambled, or fried, made with cooking spray	5000_MPE	35	36	-1	4	3.5	B
33001110	Egg substitute, cheese flavored, omelet, scrambled, or fried, fat not added in cooking	5000_MPE	35	36	-1	4	3.5	B
33001210	Egg substitute, vegetable flavored, omelet, scrambled, or fried, fat not added in cooking	5000_MPE	35	36	-1	4	3.5	B
33401520	Egg substitute, omelet, scrambled, or fried, with meat and vegetables, NS as to fat added in cooking	5000_MPE	35	32	3	3	3	D
32400620	Egg white, omelet, scrambled, or fried, with meat and vegetables, NS as to fat added in cooking	5000_MPE	35	29	6	3	3	D
21304200	Beef, shortribs, barbecued, with sauce, NS as to fat eaten	5000_MPE	35	25	10	1	1	E
21304210	Beef, shortribs, barbecued, with sauce, lean and fat eaten	5000_MPE	35	25	10	1	1	E
22706010	Pork, neck bones, cooked	5000_MPE	35	23	12	2	3.5	D
24127210	Chicken breast, fried, coated, skin / coating eaten, from pre-cooked	5000_MPE	34	35	-1	4	4	B
24147321	Chicken drumstick, fried, coated, skin / coating not eaten, from fast food / restaurant	5000_MPE	34	34	0	4	3.5	C
24152250	Chicken thigh, baked or broiled, skin eaten, from fast food / restaurant	5000_MPE	34	33	1	4	3.5	D
24167135	Chicken, wing, from fast food, coated, baked or fried, prepared with skin, skin/coating not eaten	5000_MPE	34	32	2	4	3.5	D
33401100	Egg substitute, omelet, scrambled, or fried, with meat	5000_MPE	34	31	3	3	3.5	C
33401620	Egg substitute, omelet, scrambled, or fried, with cheese, meat, and vegetables, NS as to fat added in cooking	5000_MPE	34	31	3	3	3	D
22709110	Pork skin, boiled	5000_MPE	34	24	10	1	0.5	E
32105160	Egg omelet or scrambled egg, with chorizo	5000_MPE	34	22	12	3	1.5	D
25170210	Chitterlings, cooked	5000_MPE	34	18	16	1	2	D

24198729	Chicken nuggets, NFS	5000_MPE	33	35	-2	4	3	D
24198730	Chicken nuggets, from fast food / restaurant	5000_MPE	33	35	-2	4	3	D
24198731	Chicken nuggets, from fast food	5000_MPE	33	35	-2	4	3	D
24198732	Chicken nuggets, from restaurant	5000_MPE	33	35	-2	4	3	D
24152240	Chicken thigh, baked or broiled, skin eaten, from pre-cooked	5000_MPE	33	31	2	4	4	B
32400720	Egg white, omelet, scrambled, or fried, with cheese, meat, and vegetables, NS as to fat added in cooking	5000_MPE	33	27	6	3	3	D
32400200	Egg white, omelet, scrambled, or fried, with meat	5000_MPE	33	26	7	2	3.5	C
24107080	Chicken, NS as to part, baked, coated, skin / coating eaten	5000_MPE	32	32	0	4	2	D
24157321	Chicken thigh, fried, coated, skin / coating not eaten, from fast food	5000_MPE	32	32	0	4	3.5	D
24162150	Chicken wing, baked or broiled, from fast food / restaurant	5000_MPE	32	30	2	4	2	D
33001020	Egg substitute, omelet, scrambled, or fried, made with butter	5000_MPE	32	26	6	3	3	D
24198737	Chicken nuggets, from other sources	5000_MPE	31	33	-2	4	3	D
32105190	Egg casserole with bread, cheese, milk and meat	5000_MPE	31	21	10	3	3	C
24147310	Chicken drumstick, fried, coated, skin / coating eaten, from pre-cooked	5000_MPE	30	31	-1	4	3.5	D
24162140	Chicken wing, baked or broiled, from pre-cooked	5000_MPE	30	27	3	4	2	D
24157330	Chicken thigh, fried, coated, skin / coating eaten, from restaurant	5000_MPE	30	26	4	3	2	D
22709010	Pork skin rinds	5000_MPE	30	21	9	3	0.5	E
22501010	Canadian bacon, cooked	5000_MPE	30	18	12	3	2	D
23345100	Wild pig, smoked	5000_MPE	30	15	15	1	3.5	D
24168012	Chicken "wings", plain, from precooked	5000_MPE	29	30	-1	4	3	D
24147225	Chicken, drumstick, from fast food, coated, baked or fried, prepared with skin, NS as to skin/coating eaten	5000_MPE	29	28	1	4	2	D
24147230	Chicken, drumstick, from fast food, coated, baked or fried, prepared with skin, skin/coating eaten	5000_MPE	29	28	1	4	2	D
24167230	Chicken wing, fried, coated, from restaurant	5000_MPE	29	26	3	4	1.5	D
32400070	Egg white omelet, scrambled, or fried, made with butter	5000_MPE	29	20	9	1	3.5	C
21601010	Beef, bacon, reduced sodium, cooked	5000_MPE	29	19	10	3	0.5	E
24208500	Turkey bacon, cooked	5000_MPE	29	19	10	3	0.5	E
32101500	Egg, Benedict	5000_MPE	29	18	11	2	1.5	E
32105125	Egg omelet or scrambled egg, with hot dogs	5000_MPE	29	16	13	3	2	D
22707010	Pork, pig's feet, cooked	5000_MPE	29	14	15	1	3.5	C
24127220	Chicken breast, fried, coated, skin / coating eaten, from fast food / restaurant	5000_MPE	28	29	-1	4	3	D
24157310	Chicken thigh, fried, coated, skin / coating eaten, from pre-cooked	5000_MPE	28	28	0	4	3	D
24127125	Chicken, breast, from fast food, coated, baked or fried, prepared with skin, NS as to skin/coating eaten	5000_MPE	28	26	2	4	3.5	D
24127130	Chicken, breast, from fast food, coated, baked or fried, prepared with skin, skin/coating eaten	5000_MPE	28	26	2	4	3.5	D
27146050	Chicken wing with hot pepper sauce	5000_MPE	28	26	2	4	1.5	E
32400420	Egg white, omelet, scrambled, or fried, with cheese and meat, NS as to fat added in cooking	5000_MPE	28	20	8	3	2	D

22300140	Ham, fried, lean only eaten	5000_MPE	28	19	9	3	1.5	E
22600210	Pork bacon, NS as to fresh, smoked or cured, reduced sodium, cooked	5000_MPE	28	19	9	3	0.5	E
22602010	Pork bacon, smoked or cured, reduced sodium, cooked	5000_MPE	28	19	9	3	0.5	E
32400220	Egg white, omelet, scrambled, or fried, with meat, NS as to fat added in cooking	5000_MPE	28	19	9	3	2	D
22311210	Ham, smoked or cured, low sodium, cooked, lean and fat eaten	5000_MPE	28	18	10	3	2	D
23321050	Venison/deer, cured	5000_MPE	28	11	17	1	1.5	D
24168031	Chicken "wings", boneless, with hot sauce, from other sources	5000_MPE	27	27	0	4	2	D
24107070	Chicken, NS as to part, fried, coated, skin / coating eaten	5000_MPE	27	25	2	4	2	D
22300120	Ham, fried, NS as to fat eaten	5000_MPE	27	18	9	3	1.5	D
22300130	Ham, fried, lean and fat eaten	5000_MPE	27	18	9	3	1.5	D
22311200	Ham, smoked or cured, low sodium, cooked, NS as to fat eaten	5000_MPE	27	17	10	3	2	D
32400710	Egg white, omelet, scrambled, or fried, with cheese, meat, and vegetables, fat not added in cooking	5000_MPE	27	17	10	3	3.5	D
22107020	Pork chop, smoked or cured, cooked, lean only eaten	5000_MPE	27	16	11	3	1.5	E
24201520	Turkey, light or dark meat, smoked, skin not eaten	5000_MPE	27	12	15	3	2	D
24202120	Turkey, drumstick, smoked, skin eaten	5000_MPE	27	12	15	3	1.5	D
24168002	Chicken "wings", plain, from fast food / restaurant	5000_MPE	26	27	-1	4	1.5	D
24198736	Chicken nuggets, from frozen	5000_MPE	26	27	-1	4	3	D
24198740	Chicken nuggets	5000_MPE	26	27	-1	4	3	D
24147320	Chicken drumstick, fried, coated, skin / coating eaten, from fast food / restaurant	5000_MPE	26	26	0	4	2	D
22300170	Ham, breaded or floured, fried, lean only eaten	5000_MPE	26	18	8	3	1.5	D
22600100	Bacon, NS as to type of meat, cooked	5000_MPE	26	16	10	3	0.5	E
22600200	Pork bacon, NS as to fresh, smoked or cured, cooked	5000_MPE	26	16	10	3	0.5	E
22601000	Pork bacon, smoked or cured, cooked	5000_MPE	26	16	10	3	0.5	E
22601040	Bacon or side pork, fresh, cooked	5000_MPE	26	16	10	3	0.5	E
89901000	Bacon, for use with vegetables	5000_MPE	26	16	10	3	0.5	E
89902100	Bacon, for use on a sandwich	5000_MPE	26	16	10	3	0.5	E
22421000	Pork roast, smoked or cured, cooked, NS as to fat eaten	5000_MPE	26	15	11	3	1.5	E
22421010	Pork roast, smoked or cured, cooked, lean and fat eaten	5000_MPE	26	15	11	3	1.5	E
22601020	Pork bacon, smoked or cured, cooked, lean only eaten	5000_MPE	26	15	11	3	1	E
32400610	Egg white, omelet, scrambled, or fried, with meat and vegetables, fat not added in cooking	5000_MPE	26	15	11	3	3.5	C
22311450	Ham, prosciutto	5000_MPE	26	14	12	3	0.5	E
22421020	Pork roast, smoked or cured, cooked, lean only eaten	5000_MPE	26	14	12	3	1.5	D
24201500	Turkey, light or dark meat, smoked, cooked, NS as to skin eaten	5000_MPE	26	12	14	3	2	D
24201510	Turkey, light or dark meat, smoked, skin eaten	5000_MPE	26	12	14	3	2	D
25240110	Chicken salad spread	5000_MPE	25	24	1	4	3	D
24168011	Chicken "wings" with other sauces or seasoning, from precooked	5000_MPE	25	23	2	4	2	D

33401510	Egg substitute, omelet, scrambled, or fried, with meat and vegetables, fat not added in cooking	5000_MPE	25	21	4	4	3.5	C
33401320	Egg substitute, omelet, scrambled, or fried, with cheese and meat, NS as to fat added in cooking	5000_MPE	25	20	5	3	2	D
22300150	Ham, breaded or floured, fried, NS as to fat eaten	5000_MPE	25	17	8	3	2	D
22300160	Ham, breaded or floured, fried, lean and fat eaten	5000_MPE	25	17	8	3	2	D
22311220	Ham, smoked or cured, low sodium, cooked, lean only eaten	5000_MPE	25	13	12	3	3.5	D
24203120	Turkey, wing, smoked, skin eaten	5000_MPE	25	11	14	3	1.5	D
24167210	Chicken wing, fried, coated, from pre-cooked	5000_MPE	24	24	0	4	3	D
33401120	Egg substitute, omelet, scrambled, or fried, with meat, NS as to fat added in cooking	5000_MPE	24	19	5	3	2	D
24168030	Chicken "wings", boneless, with hot sauce, from fast food / restaurant	5000_MPE	23	24	-1	4	1.5	D
24168001	Chicken "wings" with other sauces or seasoning, from fast food / restaurant	5000_MPE	23	23	0	4	1.5	D
25221855	Turkey or chicken sausage, reduced sodium	5000_MPE	23	21	2	4	4	B
25221875	Turkey or chicken, pork, and beef sausage, reduced sodium	5000_MPE	23	21	2	4	3.5	C
25220108	Beef sausage, reduced sodium	5000_MPE	22	21	1	4	3.5	C
25221408	Pork sausage, reduced sodium	5000_MPE	22	21	1	4	3.5	C
33401610	Egg substitute, omelet, scrambled, or fried, with cheese, meat, and vegetables, fat not added in cooking	5000_MPE	22	17	5	4	3	D
22311000	Ham, smoked or cured, cooked, NS as to fat eaten	5000_MPE	22	13	9	3	1.5	E
22311010	Ham, smoked or cured, cooked, lean and fat eaten	5000_MPE	22	13	9	3	1.5	E
22605010	Pork bacon, formed, lean meat added, cooked	5000_MPE	22	13	9	3	0.5	E
32400410	Egg white, omelet, scrambled, or fried, with cheese and meat, fat not added in cooking	5000_MPE	22	13	9	3	2	D
22107000	Pork chop, smoked or cured, cooked, NS as to fat eaten	5000_MPE	22	12	10	3	1	E
22107010	Pork chop, smoked or cured, cooked, lean and fat eaten	5000_MPE	22	12	10	3	1	E
22311520	Ham, smoked or cured, canned, lean only eaten	5000_MPE	22	12	10	3	2	D
21601000	Beef, bacon, cooked	5000_MPE	22	11	11	3	0.5	E
21601250	Beef, bacon, cooked, lean only eaten	5000_MPE	22	9	13	3	1.5	E
24157320	Chicken thigh, fried, coated, skin / coating eaten, from fast food	5000_MPE	21	21	0	4	2	D
24167220	Chicken wing, fried, coated, from fast food	5000_MPE	21	21	0	4	1.5	D
24157225	Chicken, thigh, from fast food, coated, baked or fried, prepared with skin, NS as to skin/coating eaten	5000_MPE	21	19	2	4	2	D
24157230	Chicken, thigh, from fast food, coated, baked or fried, prepared with skin, skin/coating eaten	5000_MPE	21	19	2	4	2	D
24167125	Chicken, wing, from fast food, coated, baked or fried, prepared with skin, NS as to skin/coating eaten	5000_MPE	21	19	2	4	1.5	D
24167130	Chicken, wing, from fast food, coated, baked or fried, prepared with skin, skin/coating eaten	5000_MPE	21	19	2	4	1.5	D
22311020	Ham, smoked or cured, cooked, lean only eaten	5000_MPE	21	11	10	3	1.5	D
89901002	Ham, for use with vegetables	5000_MPE	21	9	12	3	2	D
21500300	Ground beef patty, cooked (for fast food sandwiches)	5000_MPE	20	17	3	4	2	D
22311500	Ham, smoked or cured, canned, NS as to fat eaten	5000_MPE	20	10	10	3	2	D

22311510	Ham, smoked or cured, canned, lean and fat eaten	5000_MPE	20	10	10	3	2	D
32400210	Egg white, omelet, scrambled, or fried, with meat, fat not added in cooking	5000_MPE	20	10	10	3	2	D
24168010	Chicken "wings" with hot sauce, from precooked	5000_MPE	19	19	0	4	1.5	D
21601500	Beef, bacon, formed, lean meat added, cooked	5000_MPE	19	8	11	3	0.5	E
21002000	Beef, pickled	5000_MPE	19	6	13	3	1.5	E
21416000	Corned beef, cooked, NS as to fat eaten	5000_MPE	19	6	13	3	1.5	E
21416110	Corned beef, cooked, lean and fat eaten	5000_MPE	19	6	13	3	1.5	E
24168000	Chicken "wings" with hot sauce, from fast food / restaurant	5000_MPE	18	18	0	4	1.5	E
25230560	Liverwurst	5000_MPE	18	14	4	4	1	E
33401310	Egg substitute, omelet, scrambled, or fried, with cheese and meat, fat not added in cooking	5000_MPE	18	12	6	4	2	D
24208510	Turkey bacon, reduced sodium, cooked	5000_MPE	18	9	9	3	1.5	E
22621000	Salt pork, cooked	5000_MPE	17	7	10	3	0.5	E
21602010	Beef, dried, chipped, cooked in fat	5000_MPE	17	4	13	3	0.5	E
25230430	Ham and cheese loaf	5000_MPE	15	13	2	4	1.5	E
25221830	Turkey or chicken sausage	5000_MPE	15	10	5	4	3.5	D
21416120	Corned beef, cooked, lean only eaten	5000_MPE	15	3	12	3	1.5	E
21416150	Corned beef, canned, ready-to-eat	5000_MPE	15	3	12	3	1.5	E
23321900	Venison/deer jerky	5000_MPE	15	1	14	1	0.5	E
25221860	Turkey or chicken sausage, reduced fat	5000_MPE	14	9	5	4	3.5	D
25220010	Cold cut, NFS	5000_MPE	14	8	6	4	2	D
25220510	Capicola	5000_MPE	14	8	6	4	2	D
25230230	Ham, sliced, extra lean, prepackaged or deli, luncheon meat	5000_MPE	14	8	6	4	2	D
25230520	Ham, luncheon meat, chopped, minced, pressed, spiced, lowfat, not canned	5000_MPE	14	8	6	4	2	D
25231120	Beef, prepackaged or deli, luncheon meat, reduced sodium	5000_MPE	14	8	6	4	3.5	C
21603000	Beef, pastrami (beef, smoked, spiced)	5000_MPE	14	1	13	3	2	D
25220106	Beef sausage, reduced fat	5000_MPE	13	8	5	4	1.5	D
33401110	Egg substitute, omelet, scrambled, or fried, with meat, fat not added in cooking	5000_MPE	13	8	5	4	3	D
25230220	Ham, prepackaged or deli, luncheon meat, reduced sodium	5000_MPE	13	7	6	4	3.5	D
25221220	Pastrami, made from any kind of meat, reduced fat	5000_MPE	13	6	7	4	3.5	D
25231110	Beef, prepackaged or deli, luncheon meat	5000_MPE	13	6	7	4	3.5	D
25221406	Pork sausage, reduced fat	5000_MPE	12	8	4	4	1.5	D
25221450	Pork sausage rice links	5000_MPE	12	8	4	4	1.5	D
25221505	Salami, made from any type of meat, reduced fat	5000_MPE	12	6	6	4	1.5	D
25230110	Luncheon meat, NFS	5000_MPE	12	6	6	4	2	D
25230210	Ham, prepackaged or deli, luncheon meat	5000_MPE	12	6	6	4	2	D
21602000	Beef, dried, chipped, uncooked	5000_MPE	12	1	11	3	0.5	E
25221405	Pork sausage	5000_MPE	11	7	4	4	1	E

25230310	Chicken or turkey loaf, prepackaged or deli, luncheon meat	5000_MPE	11	5	6	4	3	D
25230900	Turkey or chicken breast, prepackaged or deli, luncheon meat	5000_MPE	11	5	6	4	3	D
25220710	Chorizo	5000_MPE	10	5	5	4	1	E
25230840	Turkey salami	5000_MPE	10	4	6	4	1.5	D
25230785	Turkey, prepackaged or deli, luncheon meat, reduced sodium	5000_MPE	10	3	7	4	3.5	C
25240310	Roast beef spread	5000_MPE	9	7	2	4	1.5	E
25220360	Bratwurst, with cheese	5000_MPE	9	5	4	4	1	E
25221870	Turkey or chicken and pork sausage	5000_MPE	9	4	5	4	1.5	E
25221850	Turkey sausage, smoked	5000_MPE	9	2	7	4	2	D
22431000	Pork roll, cured, fried	5000_MPE	9	1	8	3	0.5	E
25230420	Ham luncheon meat, loaf type	5000_MPE	9	1	8	4	2	D
25230780	Turkey, prepackaged or deli, luncheon meat	5000_MPE	9	1	8	4	3.5	D
21602100	Beef jerky	5000_MPE	8	4	4	4	0.5	E
22002800	Pork jerky	5000_MPE	8	4	4	4	0.5	E
25220310	Bockwurst	5000_MPE	8	3	5	4	2	D
25220350	Bratwurst	5000_MPE	8	3	5	4	1	E
25230790	Turkey ham, sliced, extra lean, prepackaged or deli, luncheon meat	5000_MPE	8	1	7	4	2	D
25230800	Turkey ham, prepackaged or deli, luncheon meat	5000_MPE	8	1	7	4	2	D
25220120	Beef sausage, smoked, stick	5000_MPE	6	1	5	4	0.5	E
25221400	Sausage, NFS	5000_MPE	6	1	5	4	1	E
25221515	Salami, made from any type of meat, reduced sodium	5000_MPE	6	1	5	4	0.5	E
25221350	Italian sausage	5000_MPE	5	2	3	4	1	E
25240220	Ham salad spread	5000_MPE	5	2	3	4	1.5	E
22321110	Ham, smoked or cured, ground patty	5000_MPE	5	1	4	4	1	E
25221410	Pork sausage, fresh, bulk, patty or link, cooked	5000_MPE	5	1	4	4	1	E
25221430	Pork sausage, country style, fresh, cooked	5000_MPE	5	1	4	4	1	E
25221840	Turkey breakfast sausage, bulk, patty or link, cooked	5000_MPE	5	1	4	4	3	D
25230540	Ham, pork and chicken, canned luncheon meat, chopped, minced, pressed, spiced, reduced fat and reduced sodium	5000_MPE	5	1	4	4	1	E
25230550	Ham, pork, and chicken, canned luncheon meat, chopped, minced, pressed, spiced, reduced sodium	5000_MPE	5	1	4	4	1	E
25230610	Luncheon meat, loaf type	5000_MPE	5	1	4	4	1.5	E
25210170	Frankfurter or hot dog, chili-filled	5000_MPE	4	1	3	4	1	E
25220420	Bologna, Lebanon	5000_MPE	4	1	3	4	1.5	E
25220650	Turkey or chicken and beef sausage	5000_MPE	4	1	3	4	1.5	E
25221255	Pepperoni, reduced fat	5000_MPE	4	1	3	4	0.5	E
25221500	Salami, NFS	5000_MPE	4	1	3	4	0.5	E
25221520	Salami, dry or hard	5000_MPE	4	1	3	4	0.5	E
25221610	Scrapple, cooked	5000_MPE	4	1	3	4	3	D
25221920	Vienna sausage, chicken, canned	5000_MPE	4	1	3	4	2	D

25230340	Chicken, prepackaged or deli, luncheon meat, reduced sodium	5000_MPE	4	1	3	4	3.5	C
25210290	Frankfurter or hot dog, meat and poultry, reduced fat or light	5000_MPE	3	1	2	4	3	D
25210310	Frankfurter or hot dog, chicken	5000_MPE	3	1	2	4	1.5	D
25210410	Frankfurter or hot dog, turkey	5000_MPE	3	1	2	4	1.5	D
25210620	Frankfurter or hot dog, beef, reduced fat or light	5000_MPE	3	1	2	4	3	D
25210700	Frankfurter or hot dog, meat & poultry, lowfat	5000_MPE	3	1	2	4	3	D
25220460	Bologna, pork	5000_MPE	3	1	2	4	1.5	E
25221510	Salami, soft, cooked	5000_MPE	3	1	2	4	0.5	E
25221950	Pickled sausage	5000_MPE	3	1	2	4	1	E
25230320	Chicken, prepackaged or deli, luncheon meat	5000_MPE	3	1	2	4	3	D
25230510	Ham, luncheon meat, chopped, minced, pressed, spiced, not canned	5000_MPE	3	1	2	4	1.5	E
25230710	Sandwich loaf, luncheon meat	5000_MPE	3	1	2	4	2	D
25230820	Turkey pastrami	5000_MPE	3	1	2	4	2	D
25230905	Turkey or chicken breast, low salt, prepackaged or deli, luncheon meat	5000_MPE	3	1	2	4	3.5	D
22001000	Pork, pickled, NS as to cut	5000_MPE	2	1	1	3	2	D
22707020	Pork, pig's feet, pickled	5000_MPE	2	1	1	3	2	D
25210150	Frankfurter or hot dog, cheese-filled	5000_MPE	2	1	1	4	1	E
25210250	Frankfurter or hot dog, meat and poultry, fat free	5000_MPE	2	1	1	4	3	D
25220140	Beef sausage, fresh, bulk, patty or link, cooked	5000_MPE	2	1	1	4	1	E
25220490	Bologna, with cheese	5000_MPE	2	1	1	4	1	E
25221420	Pork sausage, brown and serve, cooked	5000_MPE	2	1	1	4	1	E
23322100	Deer sausage	5000_MPE	1	1	0	4	1	E
25210110	Frankfurter or hot dog, NFS	5000_MPE	1	1	0	4	1	E
25210210	Frankfurter or hot dog, beef	5000_MPE	1	1	0	4	1	E
25210220	Frankfurter or hot dog, beef and pork	5000_MPE	1	1	0	4	1.5	E
25210230	Frankfurter or hot dog, beef and pork, lowfat	5000_MPE	1	1	0	4	1.5	E
25210240	Frankfurter or hot dog, beef and pork, reduced fat or light	5000_MPE	1	1	0	4	1.5	E
25210280	Frankfurter or hot dog, meat and poultry	5000_MPE	1	1	0	4	1.5	E
25210510	Frankfurter or hot dog, low salt	5000_MPE	1	1	0	4	1.5	D
25210610	Frankfurter or hot dog, beef, lowfat	5000_MPE	1	1	0	4	1	E
25220100	Beef sausage, NFS	5000_MPE	1	1	0	4	1	E
25220105	Beef sausage	5000_MPE	1	1	0	4	1	E
25220110	Beef sausage, brown and serve, links, cooked	5000_MPE	1	1	0	4	0.5	E
25220130	Beef sausage, smoked	5000_MPE	1	1	0	4	1	E
25220150	Beef sausage with cheese	5000_MPE	1	1	0	4	0.5	E
25220210	Blood sausage	5000_MPE	1	1	0	4	1	E
25220390	Bologna, beef, lowfat	5000_MPE	1	1	0	4	1.5	D
25220400	Bologna, pork and beef	5000_MPE	1	1	0	4	1	E

25220410	Bologna, NFS	5000_MPE	1	1	0	4	0.5	E
25220425	Bologna, made from any kind of meat, reduced fat	5000_MPE	1	1	0	4	1.5	D
25220430	Bologna, beef	5000_MPE	1	1	0	4	1	E
25220435	Bologna, made from any kind of meat, reduced sodium	5000_MPE	1	1	0	4	1	E
25220440	Bologna, turkey	5000_MPE	1	1	0	4	1.5	E
25220445	Bologna, made from any kind of meat, reduced fat and reduced sodium	5000_MPE	1	1	0	4	1.5	D
25220450	Bologna ring, smoked	5000_MPE	1	1	0	4	1	E
25220470	Bologna, beef, lower sodium	5000_MPE	1	1	0	4	1	E
25220480	Bologna, chicken, beef, and pork	5000_MPE	1	1	0	4	1	E
25220500	Bologna, beef and pork, lowfat	5000_MPE	1	1	0	4	1	E
25220610	Cervelat, soft	5000_MPE	1	1	0	4	0.5	E
25220910	Head cheese	5000_MPE	1	1	0	4	2	D
25221110	Knockwurst	5000_MPE	1	1	0	4	1	E
25221210	Mortadella	5000_MPE	1	1	0	4	0.5	E
25221215	Pastrami, NFS	5000_MPE	1	1	0	4	2	D
25221250	Pepperoni, NFS	5000_MPE	1	1	0	4	0.5	E
25221260	Pepperoni, reduced sodium	5000_MPE	1	1	0	4	0.5	E
25221310	Polish sausage	5000_MPE	1	1	0	4	1	E
25221460	Pork and beef sausage	5000_MPE	1	1	0	4	1	E
25221470	Pork and beef sausage, brown and serve, cooked	5000_MPE	1	1	0	4	1	E
25221480	Mettwurst	5000_MPE	1	1	0	4	1	E
25221530	Salami, beef	5000_MPE	1	1	0	4	1	E
25221650	Smoked link sausage, pork	5000_MPE	1	1	0	4	1	E
25221660	Smoked link sausage, pork and beef	5000_MPE	1	1	0	4	1	E
25221680	Smoked sausage, pork	5000_MPE	1	1	0	4	1	E
25221710	Souse	5000_MPE	1	1	0	4	3	D
25221810	Thuringer	5000_MPE	1	1	0	4	0.5	E
25221880	Turkey or chicken, pork, and beef sausage, reduced fat	5000_MPE	1	1	0	4	1.5	E
25221890	Turkey, pork, and beef sausage, lowfat, smoked	5000_MPE	1	1	0	4	3	C
25221910	Vienna sausage, canned	5000_MPE	1	1	0	4	1.5	E
25230235	Ham, sliced, extra lean, lower sodium, prepackaged or deli, luncheon meat	5000_MPE	1	1	0	4	2	D
25230410	Ham loaf, luncheon meat	5000_MPE	1	1	0	4	0.5	E
25230450	Honey loaf	5000_MPE	1	1	0	4	3.5	D
25230530	Ham and pork, canned luncheon meat, chopped, minced, pressed, spiced	5000_MPE	1	1	0	4	0.5	E
25230810	Veal loaf	5000_MPE	1	1	0	4	0.5	E
25231150	Corned beef, pressed	5000_MPE	1	1	0	4	1.5	E
25240000	Meat spread or potted meat, NFS	5000_MPE	1	1	0	4	1.5	E
25240210	Ham, deviled or potted	5000_MPE	1	1	0	4	0.5	E

25240320	Corned beef spread	5000_MPE	1	1	0	4	1.5	E
26118020	Halibut, baked or broiled, made with oil	5800_Seafood	100	100	0	1	4	A
26153100	Tuna, fresh, raw	5800_Seafood	100	100	0	1	4.5	A
26321110	Snails, cooked, NS as to cooking method	5800_Seafood	100	100	0	1	4	A
26137100	Salmon, raw	5800_Seafood	100	97	3	1	4.5	A
26100100	Fish, NS as to type, raw	5800_Seafood	100	96	4	1	4.5	A
26117110	Haddock, cooked, NS as to cooking method	5800_Seafood	100	96	4	1	4	A
26117120	Haddock, baked or broiled, fat added	5800_Seafood	100	96	4	1	4	A
26309160	Crayfish, boiled or steamed	5800_Seafood	100	96	4	1	4	A
26315100	Oysters, raw	5800_Seafood	100	96	4	1	4	A
26153110	Tuna, fresh, cooked, NS as to cooking method	5800_Seafood	100	95	5	1	4	A
26153120	Tuna, fresh, baked or broiled, fat added	5800_Seafood	100	95	5	1	4	A
26315180	Oysters, canned	5800_Seafood	100	95	5	3	4	A
26118024	Halibut, baked or broiled, made with cooking spray	5800_Seafood	100	94	6	1	4	A
27150230	Shrimp scampi	5800_Seafood	100	93	7	1	4	A
26305120	Crab, baked or broiled, fat added	5800_Seafood	100	92	8	1	3.5	C
26118023	Halibut, baked or broiled, no added fat	5800_Seafood	100	91	9	1	4	A
26118050	Halibut, steamed or poached	5800_Seafood	100	91	9	1	4	A
26213100	Squid, raw	5800_Seafood	100	90	10	1	4.5	A
26137110	Salmon, cooked, NS as to cooking method	5800_Seafood	99	92	7	1	4	B
26137120	Salmon, baked or broiled, made with oil	5800_Seafood	99	92	7	1	4	B
26319120	Shrimp, baked or broiled, made with oil	5800_Seafood	99	90	9	1	4	A
26137170	Salmon, dried	5800_Seafood	98	93	5	3	4.5	B
26109120	Cod, baked or broiled, made with oil	5800_Seafood	98	91	7	1	4	A
26121100	Mackerel, raw	5800_Seafood	98	91	7	1	4	A
26129120	Pike, baked or broiled, fat added	5800_Seafood	98	91	7	1	4	A
26109110	Cod, cooked, NS as to cooking method	5800_Seafood	98	90	8	1	4	A
26311120	Lobster, baked or broiled, fat added	5800_Seafood	98	90	8	1	3.5	D
26127120	Perch, baked or broiled, made with oil	5800_Seafood	98	89	9	1	4	A
26207110	Roe, shad, cooked	5800_Seafood	98	88	10	1	4	B
26100120	Fish, NS as to type, baked or broiled, made with oil	5800_Seafood	97	91	6	1	4	B
26100110	Fish, NS as to type, cooked, NS as to cooking method	5800_Seafood	97	90	7	1	4	B
26319124	Shrimp, baked or broiled, made with cooking spray	5800_Seafood	97	87	10	1	4	A
26215120	Turtle, cooked, NS as to cooking method	5800_Seafood	96	91	5	1	4	A
26147110	Sturgeon, cooked, NS as to cooking method	5800_Seafood	96	90	6	1	4	A
26153170	Tuna, fresh, dried	5800_Seafood	96	90	6	3	4.5	A
26119100	Herring, raw	5800_Seafood	96	88	8	1	4	A
26137124	Salmon, baked or broiled, made with cooking spray	5800_Seafood	96	88	8	1	4	B
26319122	Shrimp, baked or broiled, made with margarine	5800_Seafood	96	86	10	2	4	A

26139180	Sardines, canned in oil	5800_Seafood	95	91	4	3	4	A
26139110	Sardines, cooked	5800_Seafood	95	90	5	3	4	A
26115120	Flounder, baked or broiled, made with oil	5800_Seafood	95	88	7	1	4	B
26137122	Salmon, baked or broiled, made with margarine	5800_Seafood	95	87	8	1	4	B
26141110	Sea bass, cooked, NS as to cooking method	5800_Seafood	95	87	8	1	4	A
26141120	Sea bass, baked or broiled, fat added	5800_Seafood	95	87	8	1	4	A
26100160	Fish, NS as to type, steamed	5800_Seafood	95	86	9	1	4	A
26137123	Salmon, baked or broiled, no added fat	5800_Seafood	95	86	9	1	4	A
26137160	Salmon, steamed or poached	5800_Seafood	95	86	9	1	4	A
26157120	Whiting, baked or broiled, made with oil	5800_Seafood	95	86	9	1	4	A
26213120	Squid, baked or broiled, fat added	5800_Seafood	95	86	9	1	4	A
26313100	Mussels, raw	5800_Seafood	95	86	9	1	4	A
26100123	Fish, NS as to type, baked or broiled, no added fat	5800_Seafood	95	85	10	1	4	A
26115124	Flounder, baked or broiled, made with cooking spray	5800_Seafood	95	85	10	1	4	A
26319123	Shrimp, baked or broiled, no added fat	5800_Seafood	95	83	12	1	4	A
26319130	Shrimp, steamed or boiled	5800_Seafood	95	83	12	1	4	A
26149110	Swordfish, cooked, NS as to cooking method	5800_Seafood	94	87	7	1	4	B
26149120	Swordfish, baked or broiled, fat added	5800_Seafood	94	87	7	1	4	B
26158000	Tilapia, cooked, NS as to cooking method	5800_Seafood	94	87	7	1	4	A
26158010	Tilapia, baked or broiled, made with oil	5800_Seafood	94	87	7	1	4	A
26100122	Fish, NS as to type, baked or broiled, made with margarine	5800_Seafood	94	86	8	1	4	A
26125110	Ocean perch, cooked, NS as to cooking method	5800_Seafood	94	86	8	1	4	A
26125120	Ocean perch, baked or broiled, fat added	5800_Seafood	94	86	8	1	4	A
26133120	Porgy, baked or broiled, fat added	5800_Seafood	94	86	8	1	4	A
26133110	Porgy, cooked, NS as to cooking method	5800_Seafood	94	85	9	1	4	A
26115123	Flounder, baked or broiled, no added fat	5800_Seafood	94	83	11	1	4	A
26157124	Whiting, baked or broiled, made with cooking spray	5800_Seafood	94	83	11	1	4	A
26109122	Cod, baked or broiled, made with margarine	5800_Seafood	93	85	8	2	4	A
26151110	Trout, cooked, NS as to cooking method	5800_Seafood	93	85	8	1	4	B
26151120	Trout, baked or broiled, made with oil	5800_Seafood	93	85	8	1	4	B
26111110	Croaker, cooked, NS as to cooking method	5800_Seafood	93	84	9	1	4	A
26111120	Croaker, baked or broiled, fat added	5800_Seafood	93	84	9	1	4	A
26149121	Swordfish, baked or broiled, no added fat	5800_Seafood	93	83	10	1	4	B
26315120	Oysters, baked or broiled, fat added	5800_Seafood	92	86	6	1	3.5	C
26137180	Salmon, canned	5800_Seafood	92	85	7	3	4	A
26115122	Flounder, baked or broiled, made with margarine	5800_Seafood	92	83	9	2	4	B
26149160	Swordfish, steamed or poached	5800_Seafood	92	82	10	1	4	B
26151123	Trout, baked or broiled, no added fat	5800_Seafood	92	82	10	1	4	A
26315121	Oysters, baked or broiled, no added fat	5800_Seafood	92	82	10	1	3.5	C

26117121	Haddock, baked or broiled, no added fat	5800_Seafood	92	81	11	1	4	A
26133121	Porgy, baked or broiled, no added fat	5800_Seafood	92	81	11	1	4	A
26115160	Flounder, steamed or poached	5800_Seafood	92	80	12	1	4	A
26305110	Crab, cooked, NS as to cooking method	5800_Seafood	92	79	13	1	3.5	B
26305160	Crab, hard shell, steamed	5800_Seafood	92	79	13	1	3.5	B
26105120	Carp, baked or broiled, fat added	5800_Seafood	91	84	7	1	4	A
26109180	Cod, dried, salted, salt removed in water	5800_Seafood	91	83	8	3	4.5	A
26121110	Mackerel, cooked, NS as to cooking method	5800_Seafood	91	83	8	1	3.5	D
26121120	Mackerel, baked or broiled, fat added	5800_Seafood	91	83	8	1	3.5	D
26151122	Trout, baked or broiled, made with margarine	5800_Seafood	91	82	9	1	4	B
26158012	Tilapia, baked or broiled, made with margarine	5800_Seafood	91	82	9	1	4	A
26141121	Sea bass, baked or broiled, no added fat	5800_Seafood	91	81	10	1	4	A
26153122	Tuna, fresh, baked or broiled, no added fat	5800_Seafood	91	81	10	1	4.5	A
26157122	Whiting, baked or broiled, made with margarine	5800_Seafood	91	81	10	1	4	A
26158014	Tilapia, baked or broiled, made with cooking spray	5800_Seafood	91	81	10	1	4	A
26115190	Flounder, smoked	5800_Seafood	91	80	11	1	4.5	A
26157123	Whiting, baked or broiled, no added fat	5800_Seafood	91	80	11	1	4	A
26117160	Haddock, steamed or poached	5800_Seafood	91	79	12	1	4	A
26125121	Ocean perch, baked or broiled, no added fat	5800_Seafood	91	79	12	1	4	A
26157160	Whiting, steamed or poached	5800_Seafood	91	79	12	1	4	A
26139170	Sardines, dried	5800_Seafood	90	84	6	3	4	D
26105160	Carp, steamed or poached	5800_Seafood	90	81	9	1	4	A
26121121	Mackerel, baked or broiled, no added fat	5800_Seafood	90	81	9	1	4	C
26131100	Pompano, raw	5800_Seafood	90	81	9	1	4	A
26141160	Sea bass, steamed or poached	5800_Seafood	90	80	10	1	4	A
26151160	Trout, steamed or poached	5800_Seafood	90	80	10	1	4	A
26153160	Tuna, fresh, steamed or poached	5800_Seafood	90	80	10	1	4.5	A
26125160	Ocean perch, steamed or poached	5800_Seafood	90	79	11	1	4	A
26133160	Porgy, steamed or poached	5800_Seafood	90	79	11	1	4	A
26158013	Tilapia, baked or broiled, no added fat	5800_Seafood	90	79	11	1	4	A
26315130	Oysters, steamed	5800_Seafood	90	79	11	1	3.5	C
26111121	Croaker, baked or broiled, no added fat	5800_Seafood	90	78	12	1	4	A
26100190	Fish, NS as to type, smoked	5800_Seafood	89	84	5	3	3.5	B
26137190	Salmon, smoked	5800_Seafood	89	84	5	3	3.5	B
26123120	Mullet, baked or broiled, fat added	5800_Seafood	89	80	9	1	4	A
26113190	Eel, smoked	5800_Seafood	89	79	10	1	3.5	D
26127123	Perch, baked or broiled, no added fat	5800_Seafood	89	76	13	1	4	A
26305121	Crab, baked or broiled, no added fat	5800_Seafood	89	76	13	1	3.5	C
26100180	Fish, NS as to type, canned	5800_Seafood	88	80	8	3	4	A

26155110	Tuna, canned, NS as to oil or water pack	5800_Seafood	88	80	8	3	4	A
26155190	Tuna, canned, water pack	5800_Seafood	88	80	8	3	4	A
26317110	Scallops, cooked, NS as to cooking method	5800_Seafood	88	80	8	1	4	B
26317120	Scallops, baked or broiled, fat added	5800_Seafood	88	80	8	1	4	B
26113160	Eel, steamed or poached	5800_Seafood	88	79	9	1	4	B
26119120	Herring, baked or broiled, fat added	5800_Seafood	88	79	9	1	4	C
26135120	Ray, baked or broiled, fat added	5800_Seafood	88	79	9	1	4	B
26143110	Shark, cooked, NS as to cooking method	5800_Seafood	88	79	9	1	4	B
26143120	Shark, baked or broiled, fat added	5800_Seafood	88	79	9	1	4	B
26119121	Herring, baked or broiled, no added fat	5800_Seafood	88	78	10	1	4	B
26158050	Tilapia, steamed or poached	5800_Seafood	88	77	11	1	4	A
26111160	Croaker, steamed or poached	5800_Seafood	88	76	12	1	4	A
26127160	Perch, steamed or poached	5800_Seafood	88	74	14	1	4	A
26155180	Tuna, canned, oil pack	5800_Seafood	87	81	6	3	4	B
26315190	Oysters, smoked	5800_Seafood	87	80	7	3	3.5	C
26213190	Squid, canned	5800_Seafood	87	78	9	3	4	A
26305180	Crab, canned	5800_Seafood	87	78	9	3	4	B
26303100	Clams, raw	5800_Seafood	87	76	11	1	4.5	A
26205160	Octopus, steamed	5800_Seafood	87	75	12	1	4	C
26213160	Squid, steamed or boiled	5800_Seafood	87	73	14	1	4	A
26319180	Shrimp, canned	5800_Seafood	86	77	9	3	3.5	D
26137121	Salmon, baked or broiled, made with butter	5800_Seafood	86	76	10	1	4	B
26123121	Mullet, baked or broiled, no added fat	5800_Seafood	86	74	12	1	4	A
26311110	Lobster, cooked, NS as to cooking method	5800_Seafood	86	73	13	1	3.5	C
26121180	Mackerel, canned	5800_Seafood	85	77	8	3	4	A
26301110	Abalone, cooked, NS as to cooking method	5800_Seafood	85	77	8	1	4	B
26143160	Shark, steamed or poached	5800_Seafood	85	74	11	1	4	B
26313110	Mussels, cooked, NS as to cooking method	5800_Seafood	85	74	11	1	3.5	D
26313160	Mussels, steamed or poached	5800_Seafood	85	74	11	1	3.5	D
26109123	Cod, baked or broiled, no added fat	5800_Seafood	85	72	13	1	4	A
26109124	Cod, baked or broiled, made with cooking spray	5800_Seafood	85	72	13	1	4	A
26151190	Trout, smoked	5800_Seafood	84	76	8	3	4	D
26131110	Pompano, cooked, NS as to cooking method	5800_Seafood	84	75	9	1	3.5	C
26131120	Pompano, baked or broiled, fat added	5800_Seafood	84	75	9	1	3.5	C
26303110	Clams, cooked, NS as to cooking method	5800_Seafood	84	75	9	1	4	A
26303120	Clams, baked or broiled, fat added	5800_Seafood	84	75	9	1	4	A
26319170	Shrimp, dried	5800_Seafood	84	74	10	3	0.5	E
26123160	Mullet, steamed or poached	5800_Seafood	84	73	11	1	4	A
26311121	Lobster, baked or broiled, no added fat	5800_Seafood	84	71	13	1	3.5	D

26109160	Cod, steamed or poached	5800_Seafood	84	70	14	1	4	A
26315110	Oysters, cooked, NS as to cooking method	5800_Seafood	83	80	3	2	3	C
26119110	Herring, cooked, NS as to cooking method	5800_Seafood	83	77	6	3	4	C
26139190	Sardines, skinless, boneless, packed in water	5800_Seafood	83	77	6	3	4	B
26100121	Fish, NS as to type, baked or broiled, made with butter	5800_Seafood	83	73	10	1	4	B
26213170	Squid, dried	5800_Seafood	83	73	10	3	2	D
26107124	Catfish, baked or broiled, made with cooking spray	5800_Seafood	83	72	11	1	4	A
26107123	Catfish, baked or broiled, no added fat	5800_Seafood	83	71	12	1	4	A
26315160	Oysters, coated, baked or broiled, fat added	5800_Seafood	82	78	4	2	3	C
26137130	Salmon, coated, baked or broiled, made with oil	5800_Seafood	82	76	6	2	4	A
26118030	Halibut, coated, baked or broiled, made with oil	5800_Seafood	82	75	7	2	4	A
26151121	Trout, baked or broiled, made with butter	5800_Seafood	82	72	10	1	4	B
26131121	Pompano, baked or broiled, no added fat	5800_Seafood	82	71	11	1	3.5	D
26107160	Catfish, steamed or poached	5800_Seafood	82	70	12	1	4	A
26311160	Lobster, steamed or boiled	5800_Seafood	82	69	13	1	3.5	B
26153130	Tuna, fresh, coated, baked or broiled, fat added	5800_Seafood	81	77	4	2	4	A
26205110	Octopus, cooked, NS as to cooking method	5800_Seafood	81	76	5	2	3.5	C
26107120	Catfish, baked or broiled, made with oil	5800_Seafood	81	73	8	1	3.5	B
26305130	Crab, coated, baked or broiled, fat added	5800_Seafood	81	73	8	2	3.5	D
26211100	Roe, sturgeon	5800_Seafood	81	71	10	3	1	E
26131190	Pompano, smoked	5800_Seafood	81	70	11	1	3.5	D
27150110	Shrimp cocktail	5800_Seafood	81	69	12	2	4	B
26121160	Mackerel, pickled	5800_Seafood	80	74	6	3	3	D
26149130	Swordfish, coated, baked or broiled, fat added	5800_Seafood	80	73	7	2	3.5	B
26131160	Pompano, steamed or poached	5800_Seafood	80	69	11	1	3.5	C
26317121	Scallops, baked or broiled, no added fat	5800_Seafood	80	68	12	1	4	B
27150140	Seafood sauce	5800_Seafood	79	76	3	2	3	C
26113110	Eel, cooked, NS as to cooking method	5800_Seafood	79	73	6	1	3.5	B
26137134	Salmon, coated, baked or broiled, made with cooking spray	5800_Seafood	79	71	8	1	4	A
26137133	Salmon, coated, baked or broiled, no added fat	5800_Seafood	79	70	9	1	4	A
26151150	Trout, battered, fried	5800_Seafood	79	69	10	1	4	A
26303121	Clams, baked or broiled, no added fat	5800_Seafood	79	66	13	1	4	A
26315140	Oysters, coated, fried	5800_Seafood	78	73	5	2	3	C
26105110	Carp, cooked, NS as to cooking method	5800_Seafood	78	72	6	2	3.5	B
26137140	Salmon, coated, fried, made with oil	5800_Seafood	78	72	6	2	3.5	B
26100130	Fish, NS as to type, coated, baked or broiled, made with oil	5800_Seafood	78	71	7	2	4	A
26117130	Haddock, coated, baked or broiled, fat added	5800_Seafood	78	71	7	2	4	A
26151130	Trout, coated, baked or broiled, made with oil	5800_Seafood	78	71	7	2	4	B
26303180	Clams, canned	5800_Seafood	78	69	9	3	4.5	A

26137150	Salmon, battered, fried	5800_Seafood	78	68	10	1	4	A
26319121	Shrimp, baked or broiled, made with butter	5800_Seafood	78	65	13	1	3.5	B
26157110	Whiting, cooked, NS as to cooking method	5800_Seafood	77	70	7	2	4	A
26309140	Crayfish, coated, fried	5800_Seafood	77	70	7	2	4	A
26119190	Herring, smoked, kippered	5800_Seafood	77	69	8	3	2	D
26127110	Perch, cooked, NS as to cooking method	5800_Seafood	77	69	8	2	4	A
26153131	Tuna, fresh, coated, baked or broiled, no added fat	5800_Seafood	77	68	9	2	4	A
26100150	Fish, NS as to type, battered, fried	5800_Seafood	77	67	10	1	4	A
26157150	Whiting, battered, fried	5800_Seafood	77	66	11	1	4	A
26115121	Flounder, baked or broiled, made with butter	5800_Seafood	77	65	12	1	3.5	B
26158011	Tilapia, baked or broiled, made with butter	5800_Seafood	77	65	12	1	4	B
26127121	Perch, baked or broiled, made with butter	5800_Seafood	77	63	14	1	4	B
26153140	Tuna, fresh, coated, fried	5800_Seafood	76	73	3	2	4	A
26133130	Porgy, coated, baked or broiled, fat added	5800_Seafood	76	68	8	2	4	A
26307140	Crab, soft shell, coated, fried	5800_Seafood	76	68	8	2	3.5	D
26127130	Perch, coated, baked or broiled, made with oil	5800_Seafood	76	67	9	2	4	A
26115110	Flounder, cooked, NS as to cooking method	5800_Seafood	75	69	6	2	3.5	C
26121140	Mackerel, coated, fried	5800_Seafood	75	69	6	2	3.5	D
26109130	Cod, coated, baked or broiled, made with oil	5800_Seafood	75	68	7	2	3.5	B
26119130	Herring, coated, baked or broiled, fat added	5800_Seafood	75	68	7	2	3.5	B
26149140	Swordfish, coated, fried	5800_Seafood	75	68	7	2	3.5	C
26111130	Croaker, coated, baked or broiled, fat added	5800_Seafood	75	67	8	2	4	B
26157130	Whiting, coated, baked or broiled, made with oil	5800_Seafood	75	67	8	2	4	A
26319110	Shrimp, cooked, NS as to cooking method	5800_Seafood	75	67	8	2	3.5	B
26213140	Squid, coated, fried	5800_Seafood	75	66	9	2	3.5	B
26319160	Shrimp, coated, baked or broiled, made with oil	5800_Seafood	75	66	9	2	3.5	B
26100133	Fish, NS as to type, coated, baked or broiled, no added fat	5800_Seafood	75	65	10	2	4	A
26101110	Anchovy, cooked, NS as to cooking method	5800_Seafood	75	65	10	3	0.5	E
26101180	Anchovy, canned	5800_Seafood	75	65	10	3	0.5	E
26151133	Trout, coated, baked or broiled, no added fat	5800_Seafood	75	65	10	1	4	A
26109150	Cod, battered, fried	5800_Seafood	75	64	11	1	4	B
26157121	Whiting, baked or broiled, made with butter	5800_Seafood	75	61	14	1	4	B
26137142	Salmon, coated, fried, made with margarine	5800_Seafood	74	67	7	2	3.5	B
26141130	Sea bass, coated, baked or broiled, fat added	5800_Seafood	74	67	7	2	4	A
26125130	Ocean perch, coated, baked or broiled, fat added	5800_Seafood	74	66	8	2	3.5	B
26151140	Trout, coated, fried, made with oil	5800_Seafood	74	66	8	2	3.5	B
26137143	Salmon, coated, fried, no added fat	5800_Seafood	74	64	10	2	4	B
26317130	Scallops, steamed or boiled	5800_Seafood	74	60	14	1	4	B
26100140	Fish, NS as to type, coated, fried, made with oil	5800_Seafood	73	66	7	2	3.5	B

26115130	Flounder, coated, baked or broiled, made with oil	5800_Seafood	73	66	7	2	3.5	B
26117140	Haddock, coated, fried	5800_Seafood	73	66	7	2	4	B
26119131	Herring, coated, baked or broiled, no added fat	5800_Seafood	73	63	10	1	3.5	B
26115150	Flounder, battered, fried	5800_Seafood	73	62	11	1	3.5	B
26127150	Perch, battered, fried	5800_Seafood	73	61	12	1	4	A
26109121	Cod, baked or broiled, made with butter	5800_Seafood	73	60	13	1	3.5	B
26303160	Clams, steamed or boiled	5800_Seafood	73	59	14	1	4	A
26158020	Tilapia, coated, baked or broiled, made with oil	5800_Seafood	72	67	5	2	4	A
26105140	Carp, coated, fried	5800_Seafood	72	65	7	2	3.5	B
26111140	Croaker, coated, fried	5800_Seafood	72	64	8	2	3.5	B
26133140	Porgy, coated, fried	5800_Seafood	72	64	8	2	3.5	B
26311140	Lobster, coated, fried	5800_Seafood	72	64	8	2	3.5	D
26317160	Scallops, coated, baked or broiled, fat added	5800_Seafood	71	65	6	2	3.5	C
26109140	Cod, coated, fried, made with oil	5800_Seafood	71	64	7	2	3.5	B
26157140	Whiting, coated, fried, made with oil	5800_Seafood	71	64	7	2	4	A
26119140	Herring, coated, fried	5800_Seafood	71	63	8	2	3.5	D
26127140	Perch, coated, fried, made with oil	5800_Seafood	71	63	8	2	4	A
26157132	Whiting, coated, baked or broiled, made with margarine	5800_Seafood	71	62	9	2	4	B
26158024	Tilapia, coated, baked or broiled, made with cooking spray	5800_Seafood	71	61	10	2	4	A
26107121	Catfish, baked or broiled, made with butter	5800_Seafood	71	60	11	1	3.5	C
26117131	Haddock, coated, baked or broiled, no added fat	5800_Seafood	71	60	11	2	4	A
26131131	Pompano, coated, baked or broiled, no added fat	5800_Seafood	71	60	11	1	3.5	B
26131150	Pompano, battered, fried	5800_Seafood	71	60	11	1	3.5	B
26117150	Haddock, battered, fried	5800_Seafood	71	59	12	1	4	A
26127133	Perch, coated, baked or broiled, no added fat	5800_Seafood	71	58	13	2	4	A
26319164	Shrimp, coated, baked or broiled, made with cooking spray	5800_Seafood	71	58	13	2	4	A
26125140	Ocean perch, coated, fried	5800_Seafood	70	63	7	2	3.5	B
26100142	Fish, NS as to type, coated, fried, made with margarine	5800_Seafood	70	62	8	2	3.5	B
26141140	Sea bass, coated, fried	5800_Seafood	70	62	8	2	3.5	B
26151142	Trout, coated, fried, made with margarine	5800_Seafood	70	62	8	2	4	B
26137131	Salmon, coated, baked or broiled, made with butter	5800_Seafood	70	61	9	2	3.5	C
26319140	Shrimp, coated, fried, made with oil	5800_Seafood	70	61	9	2	3.5	B
26100143	Fish, NS as to type, coated, fried, no added fat	5800_Seafood	70	59	11	2	4	B
26151143	Trout, coated, fried, no added fat	5800_Seafood	70	59	11	2	4	B
26157133	Whiting, coated, baked or broiled, no added fat	5800_Seafood	70	59	11	2	4	A
26100170	Fish, NS as to type, dried	5800_Seafood	70	58	12	3	0.5	E
26109170	Cod, dried, salted	5800_Seafood	70	58	12	3	0.5	E
26115140	Flounder, coated, fried, made with oil	5800_Seafood	69	62	7	2	3.5	C
26115132	Flounder, coated, baked or broiled, made with margarine	5800_Seafood	69	61	8	2	3.5	C

26123140	Mullet, coated, fried	5800_Seafood	69	61	8	2	3.5	B
26131140	Pompano, coated, fried	5800_Seafood	69	61	8	2	3.5	D
26158023	Tilapia, coated, baked or broiled, no added fat	5800_Seafood	69	58	11	2	4	A
26319163	Shrimp, coated, baked or broiled, no added fat	5800_Seafood	69	55	14	2	4	A
26203110	Frog legs, NS as to cooking method	5800_Seafood	68	67	1	2	4	A
26129140	Pike, coated, fried	5800_Seafood	68	63	5	2	4	A
26158030	Tilapia, coated, fried, made with oil	5800_Seafood	68	63	5	2	4	B
26158040	Tilapia, battered, fried	5800_Seafood	68	59	9	1	4	A
26115133	Flounder, coated, baked or broiled, no added fat	5800_Seafood	68	57	11	2	4	A
26127143	Perch, coated, fried, no added fat	5800_Seafood	68	53	15	2	4	A
26109133	Cod, coated, baked or broiled, no added fat	5800_Seafood	67	56	11	2	4	A
26109134	Cod, coated, baked or broiled, made with cooking spray	5800_Seafood	67	56	11	2	4	A
27250510	Fish cake (Kamaboko) tempura	5800_Seafood	67	55	12	1	3	D
27150070	Lobster with butter sauce	5800_Seafood	67	53	14	1	3.5	D
26107150	Catfish, battered, fried	5800_Seafood	66	58	8	1	4	A
26319142	Shrimp, coated, fried, made with margarine	5800_Seafood	66	55	11	3	3.5	C
26109144	Cod, coated, fried, made with cooking spray	5800_Seafood	66	53	13	2	4	A
26158034	Tilapia, coated, fried, made with cooking spray	5800_Seafood	66	53	13	2	4	B
26107110	Catfish, cooked, NS as to cooking method	5800_Seafood	65	61	4	2	3.5	C
26317140	Scallops, coated, fried	5800_Seafood	65	60	5	2	3.5	C
26301140	Abalone, floured or breaded, fried	5800_Seafood	65	59	6	1	3.5	D
26125150	Ocean perch, battered, fried	5800_Seafood	65	52	13	1	3.5	B
26158033	Tilapia, coated, fried, no added fat	5800_Seafood	65	52	13	2	4	B
26107130	Catfish, coated, baked or broiled, made with oil	5800_Seafood	64	58	6	2	3.5	B
26303140	Clams, coated, fried	5800_Seafood	64	58	6	2	3.5	B
26158032	Tilapia, coated, fried, made with margarine	5800_Seafood	64	57	7	3	4	B
26315150	Oysters, battered, fried	5800_Seafood	64	54	10	2	3	C
26319143	Shrimp, coated, fried, no added fat	5800_Seafood	64	51	13	2	4	B
26109143	Cod, coated, fried, no added fat	5800_Seafood	64	50	14	2	4	A
26137141	Salmon, coated, fried, made with butter	5800_Seafood	63	53	10	2	3.5	D
26145140	Smelt, floured or breaded, fried	5800_Seafood	63	52	11	2	3.5	D
26107133	Catfish, coated, baked or broiled, no added fat	5800_Seafood	62	53	9	1	4	B
26119180	Herring, pickled	5800_Seafood	61	53	8	3	2	D
26107140	Catfish, coated, fried, made with oil	5800_Seafood	60	55	5	2	3.5	C
26107144	Catfish, coated, fried, made with cooking spray	5800_Seafood	59	49	10	2	3.5	B
26319161	Shrimp, coated, baked or broiled, made with butter	5800_Seafood	59	45	14	2	3.5	C
26133150	Porgy, battered, fried	5800_Seafood	58	52	6	1	4	A
26158021	Tilapia, coated, baked or broiled, made with butter	5800_Seafood	58	49	9	2	3.5	B
26107143	Catfish, coated, fried, no added fat	5800_Seafood	58	48	10	2	3.5	B

26119160	Herring, pickled, in cream sauce	5800_Seafood	57	47	10	3	2	D
26100200	Fish, NS as to type, from fast food	5800_Seafood	55	55	0	4	3.5	C
26317150	Scallops, battered, fried	5800_Seafood	54	43	11	2	3	D
26127141	Perch, coated, fried, made with butter	5800_Seafood	54	41	13	2	3.5	D
26107131	Catfish, coated, baked or broiled, made with butter	5800_Seafood	53	44	9	2	3.5	D
26109141	Cod, coated, fried, made with butter	5800_Seafood	53	41	12	2	3	D
26303150	Clams, battered, fried	5800_Seafood	52	41	11	2	2	D
26115141	Flounder, coated, fried, made with butter	5800_Seafood	52	40	12	2	3	D
26319141	Shrimp, coated, fried, made with butter	5800_Seafood	52	38	14	2	3	D
26158031	Tilapia, coated, fried, made with butter	5800_Seafood	51	42	9	2	3.5	D
26100260	Fish stick, patty or nugget from fast food	5800_Seafood	41	40	1	4	3	D
26100270	Fish stick, patty or nugget from restaurant, home, or other place	5800_Seafood	41	40	1	4	3	D
26100210	Fish stick, patty, or fillet, NS as to type, cooked, NS as to cooking method	5800_Seafood	41	38	3	4	3	D
26100220	Fish stick, patty, or fillet, NS as to type, baked or broiled	5800_Seafood	41	38	3	4	3	D
26100230	Fish stick, patty, or fillet, NS as to type, breaded or battered, baked	5800_Seafood	41	38	3	4	3	D
26100250	Fish stick, patty, or fillet, NS as to type, battered, fried	5800_Seafood	40	38	2	4	3	C
26100240	Fish stick, patty, or fillet, NS as to type, floured or breaded, fried	5800_Seafood	38	35	3	4	3	D
26319145	Shrimp, coated, fried, from fast food / restaurant	5800_Seafood	36	37	-1	4	1.5	E
27250520	Seafood restructured	5800_Seafood	30	30	0	4	3	C
11433500	Yogurt, fruit, nonfat milk, light	6000_Dairy	87	99	-12	4	4.5	B
11411300	Yogurt, nonfat milk, plain	6000_Dairy	86	86	0	1	5	A
11411420	Yogurt, Greek, nonfat milk, plain	6000_Dairy	86	85	1	1	5	A
11513854	Chocolate milk, made from sugar free syrup with fat free milk	6000_Dairy	86	85	1	1	5	B
11410000	Yogurt, NS as to type of milk or flavor	6000_Dairy	83	80	3	1	5	A
11400010	Yogurt, Greek, NS as to type of milk or flavor	6000_Dairy	82	77	5	1	5	A
11411390	Yogurt, Greek, NS as to type of milk, plain	6000_Dairy	82	77	5	1	5	A
11411410	Yogurt, Greek, low fat milk, plain	6000_Dairy	82	77	5	1	5	A
11513394	Chocolate milk, made from no sugar added dry mix with fat free milk (Nesquik)	6000_Dairy	81	81	0	2	4.5	B
11513370	Chocolate milk, made from reduced sugar mix with fat free milk	6000_Dairy	80	80	0	2	4.5	B
11400000	Yogurt, NFS	6000_Dairy	80	76	4	1	5	A
11411010	Yogurt, NS as to type of milk, plain	6000_Dairy	80	76	4	1	5	A
11411200	Yogurt, low fat milk, plain	6000_Dairy	80	76	4	1	5	A
11411100	Yogurt, whole milk, plain	6000_Dairy	76	72	4	1	4.5	B
11350030	Almond milk, unsweetened, chocolate	6000_Dairy	75	84	-9	4	4	B
11411400	Yogurt, Greek, whole milk, plain	6000_Dairy	74	71	3	1	5	B
11350020	Almond milk, unsweetened	6000_Dairy	73	83	-10	4	4	B
11513365	Chocolate milk, made from reduced sugar mix with low fat milk	6000_Dairy	73	69	4	2	4.5	B
11513393	Chocolate milk, made from no sugar added dry mix with low fat milk (Nesquik)	6000_Dairy	73	69	4	2	4.5	B

11514350	Hot chocolate / Cocoa, made with no sugar added dry mix and fat free milk	6000_Dairy	72	70	2	2	4	B
11513395	Chocolate milk, made from no sugar added dry mix with non-dairy milk (Nesquik)	6000_Dairy	71	79	-8	4	4	C
11513855	Chocolate milk, made from sugar free syrup with non-dairy milk	6000_Dairy	71	78	-7	4	4	B
11513853	Chocolate milk, made from sugar free syrup with low fat milk	6000_Dairy	71	64	7	1	5	B
11513360	Chocolate milk, made from reduced sugar mix with reduced fat milk	6000_Dairy	70	65	5	2	4	B
11513392	Chocolate milk, made from no sugar added dry mix with reduced fat milk (Nesquik)	6000_Dairy	70	65	5	2	4	B
11513350	Chocolate milk, made from reduced sugar mix, NS as to type of milk	6000_Dairy	69	64	5	2	4	B
11513852	Chocolate milk, made from sugar free syrup with reduced fat milk	6000_Dairy	69	62	7	1	4.5	B
11513850	Chocolate milk, made from sugar free syrup, NS as to type of milk	6000_Dairy	68	61	7	1	4.5	B
11115400	Kefir, NS as to fat content	6000_Dairy	68	60	8	1	4.5	B
11513390	Chocolate milk, made from no sugar added dry mix, NS as to type of milk (Nesquik)	6000_Dairy	67	64	3	2	4	B
11518000	Milk beverage with nonfat dry milk and low calorie sweetener, water added, chocolate	6000_Dairy	66	72	-6	4	4.5	B
11113000	Milk, fat free (skim)	6000_Dairy	66	61	5	1	4.5	B
11114320	Milk, lactose free, fat free (skim)	6000_Dairy	66	61	5	1	4.5	B
11513355	Chocolate milk, made from reduced sugar mix with whole milk	6000_Dairy	66	60	6	2	4	C
11513391	Chocolate milk, made from no sugar added dry mix with whole milk (Nesquik)	6000_Dairy	66	60	6	2	4	C
11516000	Cocoa, whey, and low-calorie sweetener mixture, lowfat milk added	6000_Dairy	66	60	6	2	4.5	B
11330000	Milk, soy, dry, reconstituted, not baby's	6000_Dairy	65	73	-8	4	5	B
11111170	Milk, calcium fortified, fat free (skim)	6000_Dairy	65	60	5	1	5	B
11114321	Milk, cow's, fluid, lactose reduced, nonfat, fortified with calcium	6000_Dairy	65	60	5	1	5	B
11810000	Milk, dry, not reconstituted, NS as to fat content	6000_Dairy	65	60	5	1	1	E
11813000	Milk, dry, not reconstituted, fat free (skim)	6000_Dairy	65	60	5	1	1	E
11120000	Milk, dry, reconstituted, NS as to fat content	6000_Dairy	65	59	6	1	5	B
11424000	Yogurt, vanilla, nonfat milk, light	6000_Dairy	64	73	-9	4	4.5	B
11514360	Hot chocolate / Cocoa, made with no sugar added dry mix and non-dairy milk	6000_Dairy	64	70	-6	4	3.5	C
11812000	Milk, dry, not reconstituted, low fat (1%)	6000_Dairy	64	59	5	1	1	E
11514340	Hot chocolate / Cocoa, made with no sugar added dry mix and low fat milk	6000_Dairy	64	58	6	2	4	C
11514330	Hot chocolate / Cocoa, made with no sugar added dry mix and reduced fat milk	6000_Dairy	64	57	7	2	3.5	D
11513851	Chocolate milk, made from sugar free syrup with whole milk	6000_Dairy	64	56	8	1	4.5	B
11320100	Soy milk, light	6000_Dairy	63	76	-13	4	4.5	B
11112000	Milk, cow's, fluid, other than whole, NS as to 2%, 1%, or skim (formerly milk, cow's, fluid, "lowfat", NS as to percent fat)	6000_Dairy	62	53	9	1	4.5	B
11428000	Yogurt, Greek, chocolate, nonfat	6000_Dairy	61	72	-11	4	5	A
11513375	Chocolate milk, made from reduced sugar mix with non-dairy milk	6000_Dairy	61	71	-10	4	4	C
11446000	Yogurt parfait, low fat, with fruit	6000_Dairy	61	70	-9	4	4.5	C

11514320	Hot chocolate / Cocoa, made with no sugar added dry mix and whole milk	6000_Dairy	61	55	6	2	3	D
11111160	Milk, calcium fortified, low fat (1%)	6000_Dairy	61	52	9	1	5	B
11112120	Milk, acidophilus, low fat (1%)	6000_Dairy	61	52	9	1	5	B
11112210	Milk, low fat (1%)	6000_Dairy	61	52	9	1	5	B
11114300	Milk, lactose free, low fat (1%)	6000_Dairy	61	52	9	1	5	B
11114310	Milk, cow's, fluid, lactose reduced, 1% fat, fortified with calcium	6000_Dairy	61	52	9	1	4.5	B
11121300	Milk, dry, reconstituted, fat free (skim)	6000_Dairy	60	54	6	1	5	B
14206010	Cheese, cottage, lowfat, low sodium	6000_Dairy	60	53	7	3	5	A
11121210	Milk, dry, reconstituted, low fat (1%)	6000_Dairy	60	52	8	1	4.5	B
14107040	Cheese, Mozzarella, reduced sodium	6000_Dairy	59	51	8	3	5	C
11112110	Milk, reduced fat (2%)	6000_Dairy	59	50	9	1	4.5	B
11112130	Milk, acidophilus, reduced fat (2%)	6000_Dairy	59	50	9	1	4.5	B
11114330	Milk, lactose free, reduced fat (2%)	6000_Dairy	59	50	9	1	4.5	B
11424520	Yogurt, Greek, vanilla, nonfat	6000_Dairy	58	69	-11	4	5	A
11435030	Yogurt, Greek, nonfat milk, flavors other than fruit	6000_Dairy	58	69	-11	4	5	A
11527000	Milk, malted, fortified, NS as to flavor, made with milk	6000_Dairy	58	56	2	2	3.5	E
11212100	Milk, evaporated, skim, undiluted	6000_Dairy	58	53	5	2	4.5	E
14109020	Cheese, Swiss, reduced sodium	6000_Dairy	58	52	6	3	5	C
11100000	Milk, NFS	6000_Dairy	58	49	9	1	4.5	B
11514300	Cocoa with nonfat dry milk and low calorie sweetener, mixture, water added	6000_Dairy	57	62	-5	4	4	B
11513300	Chocolate milk, made from dry mix with fat free milk	6000_Dairy	57	59	-2	2	4	C
11212000	Milk, evaporated, skim, NS as to dilution	6000_Dairy	57	52	5	2	4.5	B
11111100	Milk, low sodium, whole	6000_Dairy	57	49	8	1	3.5	C
11519040	Strawberry milk, NFS	6000_Dairy	57	49	8	1	4	B
11114200	Milk, cow's, fluid, filled with vegetable oil, lowfat	6000_Dairy	57	48	9	1	5	B
11122000	Buttermilk, dry, reconstituted	6000_Dairy	57	46	11	1	5	B
11212050	Milk, evaporated, fat free (skim)	6000_Dairy	56	52	4	2	4.5	E
11320000	Soy milk	6000_Dairy	55	69	-14	4	4.5	B
11514500	Cocoa, whey, and low calorie sweetener, mixture, fortified, water added	6000_Dairy	55	61	-6	4	4	B
14109030	Cheese, Swiss, reduced fat	6000_Dairy	55	45	10	3	5	A
11434020	Yogurt, Greek, nonfat milk, fruit	6000_Dairy	54	66	-12	4	5	B
11514310	Hot chocolate / Cocoa, made with no sugar added dry mix and water	6000_Dairy	54	59	-5	4	4	C
11111000	Milk, whole	6000_Dairy	54	45	9	1	4.5	B
11111150	Milk, calcium fortified, whole	6000_Dairy	54	45	9	1	4.5	B
11114350	Milk, lactose free, whole	6000_Dairy	54	45	9	1	4.5	B
11513384	Chocolate milk, made from dry mix with fat free milk (Nesquik)	6000_Dairy	53	60	-7	2	4	C
11211400	Milk, evaporated, reduced fat (2%)	6000_Dairy	53	45	8	2	3.5	E
11811000	Milk, dry, not reconstituted, whole	6000_Dairy	53	44	9	1	0.5	E

11121100	Milk, dry, reconstituted, whole	6000_Dairy	53	43	10	1	3.5	C
11434300	Yogurt, nonfat milk, flavors other than fruit	6000_Dairy	52	60	-8	4	4.5	B
14610520	Cheese ball	6000_Dairy	52	53	-1	3	5	D
11525000	Milk, malted, natural flavor, made with milk	6000_Dairy	52	44	8	2	3.5	C
14108050	Cheese, Parmesan, low sodium	6000_Dairy	52	44	8	3	5	C
14110010	Cheese, Cheddar, reduced sodium	6000_Dairy	52	44	8	3	5	C
11300100	Non-dairy milk, NFS	6000_Dairy	51	66	-15	4	4	C
11513382	Chocolate milk, made from dry mix with reduced fat milk (Nesquik)	6000_Dairy	51	46	5	2	3.5	D
11519210	Strawberry milk, reduced sugar	6000_Dairy	51	42	9	1	4.5	B
11116000	Goat's milk, whole	6000_Dairy	51	41	10	1	4	C
11526000	Milk, malted	6000_Dairy	50	44	6	2	4	C
14200100	Cheese, cottage, NFS	6000_Dairy	50	41	9	3	4.5	C
14204010	Cheese, cottage, low fat	6000_Dairy	50	41	9	3	4.5	C
14207010	Cheese, cottage, lowfat, lactose reduced	6000_Dairy	50	41	9	3	4.5	C
11115200	Buttermilk, reduced fat (2%)	6000_Dairy	50	40	10	1	3.5	C
11321100	Soy milk, light, chocolate	6000_Dairy	49	66	-17	4	4	C
11321200	Soy milk, nonfat, chocolate	6000_Dairy	49	66	-17	4	4	C
11433000	Yogurt, nonfat milk, fruit	6000_Dairy	49	59	-10	4	4.5	B
11513200	Chocolate milk, made from dry mix with low fat milk	6000_Dairy	49	46	3	2	4	D
11513150	Chocolate milk, made from dry mix with reduced fat milk	6000_Dairy	49	45	4	2	3.5	D
11513000	Chocolate milk, made from dry mix, NS as to type of milk	6000_Dairy	49	44	5	2	3.5	D
11513380	Chocolate milk, made from dry mix, NS as to type of milk (Nesquik)	6000_Dairy	49	44	5	2	3.5	D
14109040	Cheese, Swiss, nonfat or fat free	6000_Dairy	49	43	6	3	5	C
11115300	Buttermilk, whole	6000_Dairy	49	41	8	1	4	C
11114000	Milk, cow's, fluid, filled with vegetable oil, NS as to percent fat	6000_Dairy	49	40	9	1	3.5	C
11114100	Milk, cow's, fluid, filled with vegetable oil, whole	6000_Dairy	49	40	9	1	3.5	C
11115000	Buttermilk, fat free (skim)	6000_Dairy	49	38	11	1	4.5	B
11115100	Buttermilk, low fat (1%)	6000_Dairy	49	38	11	1	4.5	B
11422100	Yogurt, vanilla, low fat milk, light	6000_Dairy	48	49	-1	4	4.5	A
14107060	Cheese, Mozzarella, nonfat or fat free	6000_Dairy	48	41	7	3	5	C
11425000	Yogurt, chocolate, NS as to type of milk	6000_Dairy	47	56	-9	4	4	C
11435100	Yogurt, Greek, with oats	6000_Dairy	47	56	-9	4	5	B
11427000	Yogurt, chocolate, nonfat milk	6000_Dairy	47	55	-8	4	4	C
11434090	Yogurt, NS as to type of milk, flavors other than fruit	6000_Dairy	47	51	-4	4	4.5	A
11434200	Yogurt, low fat milk, flavors other than fruit	6000_Dairy	47	51	-4	4	4.5	A
11514140	Hot chocolate / Cocoa, made with dry mix and fat free milk	6000_Dairy	47	49	-2	3	2.5	E
11513100	Chocolate milk, made from dry mix with whole milk	6000_Dairy	47	41	6	2	3	E
14201500	Cheese, Ricotta	6000_Dairy	47	40	7	3	4	C
11210000	Milk, evaporated, NS as to fat content and dilution	6000_Dairy	47	39	8	2	3.5	C

14109010	Cheese, Swiss	6000_Dairy	47	37	10	3	5	C
11320200	Soy milk, nonfat	6000_Dairy	46	59	-13	4	4.5	B
11520000	Milk, malted, unfortified, NS as to flavor, made with milk	6000_Dairy	46	41	5	2	3.5	D
11211000	Milk, evaporated, whole, NS as to dilution	6000_Dairy	46	38	8	2	3.5	C
11211100	Milk, evaporated, whole, undiluted	6000_Dairy	46	37	9	2	2	E
11321000	Soy milk, chocolate	6000_Dairy	45	60	-15	4	4	D
11444000	Yogurt, fruit and nuts, NS as to type of milk	6000_Dairy	45	55	-10	4	3.5	C
11445000	Yogurt, fruit and nuts, lowfat milk	6000_Dairy	45	55	-10	4	3.5	C
14410130	Cheese, American, nonfat or fat free	6000_Dairy	45	47	-2	4	5	D
11513383	Chocolate milk, made from dry mix with low fat milk (Nesquik)	6000_Dairy	45	46	-1	2	4	D
11432500	Yogurt, fruit, low fat milk, light	6000_Dairy	45	45	0	4	5	A
14202020	Cheese, cottage, with vegetables	6000_Dairy	45	44	1	4	4.5	B
11513804	Chocolate milk, made from light syrup with fat free milk	6000_Dairy	45	43	2	2	4	B
14203010	Cheese, cottage, dry curd	6000_Dairy	45	39	6	3	5	A
11210050	Milk, evaporated, NS as to fat content	6000_Dairy	45	36	9	2	2	E
11211050	Milk, evaporated, whole	6000_Dairy	45	36	9	2	2	E
14107010	Cheese, Mozzarella, NFS	6000_Dairy	45	35	10	3	5	D
14107030	Cheese, Mozzarella, part skim	6000_Dairy	45	35	10	3	5	D
14108420	Cheese, provolone, reduced fat	6000_Dairy	45	35	10	3	5	D
11360000	Rice milk	6000_Dairy	44	49	-5	4	3.5	D
92205000	Rice beverage	6000_Dairy	44	49	-5	4	3.5	D
11433990	Yogurt, Greek, NS as to type of milk, fruit	6000_Dairy	44	48	-4	4	5	B
11434010	Yogurt, Greek, low fat milk, fruit	6000_Dairy	44	48	-4	4	5	B
11522000	Milk, malted, unfortified, natural flavor, made with milk	6000_Dairy	44	37	7	2	4	D
11211200	Milk, evaporated, whole, diluted	6000_Dairy	44	35	9	2	3.5	C
14107250	Cheese, Muenster, reduced fat	6000_Dairy	44	35	9	3	5	D
14104015	Cheese, natural, Cheddar or American type, reduced fat	6000_Dairy	44	34	10	3	5	D
14201010	Cheese, cottage, creamed, large or small curd	6000_Dairy	44	34	10	3	4.5	B
14104110	Cheese, Cheddar, reduced fat	6000_Dairy	44	33	11	3	5	D
11430000	Yogurt, NS as to type of milk, fruit	6000_Dairy	43	47	-4	4	4	C
11432000	Yogurt, low fat milk, fruit	6000_Dairy	43	47	-4	4	4	C
14502000	Imitation cheese	6000_Dairy	43	47	-4	4	5	C
14502040	Imitation cheese, American or cheddar type, low cholesterol	6000_Dairy	43	47	-4	4	5	C
14410210	Cheese, American, reduced sodium	6000_Dairy	43	42	1	4	5	C
14120020	Cheese, Mexican blend, reduced fat	6000_Dairy	43	33	10	3	5	D
14110030	Cheese, Cheddar or Colby, lowfat	6000_Dairy	43	32	11	3	5	C
11350000	Almond milk, sweetened	6000_Dairy	42	57	-15	4	4	C
11436000	Yogurt, liquid	6000_Dairy	42	47	-5	4	4.5	D
11513381	Chocolate milk, made from dry mix with whole milk (Nesquik)	6000_Dairy	42	41	1	2	3	E

11514120	Hot chocolate / Cocoa, made with dry mix and reduced fat milk	6000_Dairy	42	37	5	3	2	E
11513803	Chocolate milk, made from light syrup with low fat milk	6000_Dairy	42	35	7	2	4	C
14203020	Cheese, cottage, salted, dry curd	6000_Dairy	42	35	7	3	4	C
14108060	Cheese, Parmesan, dry grated, fat free	6000_Dairy	42	33	9	3	5	D
14104400	Cheese, Feta	6000_Dairy	42	32	10	3	4	E
14010000	Cheese, NFS	6000_Dairy	42	31	11	3	3.5	D
11513310	Chocolate milk, made from dry mix with non-dairy milk	6000_Dairy	41	55	-14	4	3.5	E
11513385	Chocolate milk, made from dry mix with non-dairy milk (Nesquik)	6000_Dairy	41	55	-14	4	3.5	E
11513805	Chocolate milk, made from light syrup with non-dairy milk	6000_Dairy	41	55	-14	4	3.5	D
11435000	Yogurt, Greek, NS as to type of milk, flavors other than fruit	6000_Dairy	41	47	-6	4	4.5	B
11431000	Yogurt, whole milk, fruit	6000_Dairy	41	43	-2	4	3.5	C
14202010	Cheese, cottage, with fruit	6000_Dairy	41	40	1	4	4.5	B
11514130	Hot chocolate / Cocoa, made with dry mix and low fat milk	6000_Dairy	41	37	4	3	2.5	E
42401010	Coconut milk, used in cooking	6000_Dairy	41	36	5	2	0.5	D
11513800	Chocolate milk, made from light syrup, NS as to type of milk	6000_Dairy	41	34	7	2	3.5	C
11513802	Chocolate milk, made from light syrup with reduced fat milk	6000_Dairy	41	34	7	2	4	C
14103010	Cheese, Camembert	6000_Dairy	41	31	10	3	4	D
14133000	Queso Fresco	6000_Dairy	41	30	11	3	5	D
14203510	Puerto Rican white cheese	6000_Dairy	41	30	11	3	0.5	D
11424510	Yogurt, Greek, vanilla, low fat	6000_Dairy	40	45	-5	4	4.5	B
11435020	Yogurt, Greek, low fat milk, flavors other than fruit	6000_Dairy	40	45	-5	4	4.5	B
14104115	Cheese, Cheddar, nonfat or fat free	6000_Dairy	40	33	7	3	5	C
14105200	Cheese, Gruyere	6000_Dairy	40	31	9	3	3.5	D
14108015	Cheese, Parmesan, dry grated, reduced fat	6000_Dairy	40	31	9	3	3.5	E
14010100	Cheese, Cheddar or American type, NS as to natural or processed	6000_Dairy	40	30	10	3	3	D
14108400	Cheese, Provolone	6000_Dairy	40	30	10	3	3.5	D
14134000	Queso cotija	6000_Dairy	40	30	10	3	1.5	E
14100100	Cheese, natural, NFS	6000_Dairy	40	29	11	3	4	D
14104700	Cheese, goat	6000_Dairy	40	29	11	3	4	D
11514110	Hot chocolate / Cocoa, made with dry mix and whole milk	6000_Dairy	39	33	6	3	2	E
14610250	Cheese, cottage cheese, with gelatin dessert and vegetables	6000_Dairy	39	32	7	3	4	B
11513801	Chocolate milk, made from light syrup with whole milk	6000_Dairy	39	31	8	2	3.5	D
14107020	Cheese, Mozzarella, whole milk	6000_Dairy	39	30	9	3	5	D
14108010	Cheese, Parmesan, dry grated	6000_Dairy	39	30	9	3	1	E
14108020	Cheese, Parmesan, hard	6000_Dairy	39	30	9	3	1	E
14102010	Cheese, Brick	6000_Dairy	39	29	10	3	4	D
14107200	Cheese, Muenster	6000_Dairy	39	29	10	3	3.5	D
14105010	Cheese, Gouda or Edam	6000_Dairy	39	28	11	3	3.5	D
14106500	Cheese, Monterey, reduced fat	6000_Dairy	39	28	11	3	4.5	D

14108200	Cheese, Port du Salut	6000_Dairy	39	28	11	3	3.5	D
14106010	Cheese, Limburger	6000_Dairy	38	28	10	3	4	D
14103020	Cheese, Brie	6000_Dairy	38	27	11	3	4	D
14201200	Cottage cheese, farmer's	6000_Dairy	38	27	11	3	3.5	C
14104100	Cheese, Cheddar	6000_Dairy	38	26	12	3	3	D
14504010	Imitation mozzarella cheese	6000_Dairy	37	41	-4	4	5	D
11426000	Yogurt, chocolate, whole milk	6000_Dairy	37	40	-3	4	3.5	C
14660200	Mozzarella sticks, breaded, baked, or fried	6000_Dairy	37	32	5	3	5	D
11519105	Strawberry milk, reduced fat	6000_Dairy	37	31	6	2	3	D
14101010	Cheese, Blue or Roquefort	6000_Dairy	37	27	10	3	2	E
14104200	Cheese, Colby	6000_Dairy	37	27	10	3	3.5	D
14104250	Cheese, Colby Jack	6000_Dairy	37	26	11	3	3.5	D
14120010	Cheese, Mexican blend	6000_Dairy	37	26	11	3	4	D
11420000	Yogurt, vanilla, NS as to type of milk	6000_Dairy	36	41	-5	4	4	B
11422000	Yogurt, vanilla, low fat milk	6000_Dairy	36	41	-5	4	4	B
11435010	Yogurt, Greek, whole milk, flavors other than fruit	6000_Dairy	36	40	-4	4	4.5	B
11511700	Chocolate milk, ready to drink, low fat, no sugar added (Nesquik)	6000_Dairy	36	35	1	4	4	C
14204020	Cheese, cottage, lowfat, with fruit	6000_Dairy	36	35	1	4	4.5	B
11541110	Milk shake, home recipe, chocolate	6000_Dairy	36	32	4	3	2	E
11541135	Milk shake, home recipe, flavors other than chocolate, light	6000_Dairy	36	31	5	3	3	E
11541120	Milk shake, home recipe, flavors other than chocolate	6000_Dairy	36	30	6	3	2	E
14104010	Cheese, natural, Cheddar or American type	6000_Dairy	36	26	10	3	2.5	D
14104020	Cheese, Cheddar or American type, dry, grated	6000_Dairy	36	26	10	3	0.5	E
14104600	Cheese, Fontina	6000_Dairy	36	26	10	3	3	D
14102110	Cheese, Brick, with salami	6000_Dairy	36	25	11	3	4	D
14106200	Cheese, Monterey	6000_Dairy	36	25	11	3	3.5	D
11434100	Yogurt, whole milk, flavors other than fruit	6000_Dairy	35	39	-4	4	4	B
11424500	Yogurt, Greek, vanilla, whole milk	6000_Dairy	35	38	-3	4	4	C
11519205	Strawberry milk, fat free	6000_Dairy	35	36	-1	2	3.5	D
14410350	Cheese, processed, American or Cheddar type, nonfat or fat free	6000_Dairy	35	36	-1	4	5	D
14131500	Queso Asadero	6000_Dairy	35	24	11	3	4	D
14132000	Queso Chihuahua	6000_Dairy	35	23	12	3	3.5	D
11350010	Almond milk, sweetened, chocolate	6000_Dairy	34	50	-16	4	3.5	D
11423000	Yogurt, vanilla, nonfat milk	6000_Dairy	34	44	-10	4	4.5	A
11519200	Strawberry milk, low fat	6000_Dairy	34	30	4	2	3.5	D
89901020	Cheese sauce, for use with vegetables	6000_Dairy	34	24	10	3	5	D
14131000	Queso Anejo, aged Mexican cheese	6000_Dairy	34	23	11	3	2	E
11541500	Milk shake, made with skim milk, chocolate	6000_Dairy	33	32	1	3	2.5	E
14410420	Cheese, processed, Swiss, lowfat	6000_Dairy	33	29	4	4	5	D

11541400	Milk shake with malt	6000_Dairy	33	28	5	3	2	E
11519215	Strawberry milk, non-dairy	6000_Dairy	32	46	-14	4	3	E
11421000	Yogurt, vanilla, whole milk	6000_Dairy	32	35	-3	4	4	C
11513700	Chocolate milk, made from syrup with fat free milk	6000_Dairy	32	33	-1	3	3.5	E
11541130	Milk shake, home recipe, chocolate, light	6000_Dairy	32	30	2	3	3	E
11541510	Milk shake, made with skim milk, flavors other than chocolate	6000_Dairy	32	30	2	3	2.5	E
11519050	Strawberry milk, whole	6000_Dairy	32	26	6	2	2.5	E
11434000	Yogurt, Greek, whole milk, fruit	6000_Dairy	31	38	-7	4	4.5	B
14610210	Cheese, cottage cheese, with gelatin dessert and fruit	6000_Dairy	31	27	4	3	4.5	B
11513400	Chocolate milk, made from syrup, NS as to type of milk	6000_Dairy	31	26	5	2	3.5	E
11531500	Eggnog, lowfat / light	6000_Dairy	31	25	6	4	4	C
11513750	Chocolate milk, made from syrup with non-dairy milk	6000_Dairy	30	44	-14	4	3	E
11511200	Chocolate milk, ready to drink, reduced fat	6000_Dairy	30	30	0	4	3	E
14410330	Cheese spread, American or Cheddar cheese base, reduced fat	6000_Dairy	30	28	2	4	5	D
11513550	Chocolate milk, made from syrup with reduced fat milk	6000_Dairy	30	26	4	2	3.5	E
11513600	Chocolate milk, made from syrup with low fat milk	6000_Dairy	30	26	4	2	3.5	E
11519000	Milk beverage, made with whole milk, flavors other than chocolate	6000_Dairy	30	25	5	2	2.5	E
14410300	Cheese, processed, American or Cheddar type, lowfat	6000_Dairy	30	24	6	4	5	D
11514150	Hot chocolate / Cocoa, made with dry mix and non-dairy milk	6000_Dairy	29	39	-10	4	2.5	E
11512000	Cocoa, hot chocolate, not from dry mix, made with whole milk	6000_Dairy	29	32	-3	4	3	E
14410120	Cheese, American, reduced fat	6000_Dairy	29	28	1	4	5	E
14410110	Cheese, American	6000_Dairy	29	27	2	4	3.5	E
14410500	Cheese, processed cheese food	6000_Dairy	29	27	2	4	3.5	E
14410620	Cheese, with wine	6000_Dairy	29	27	2	4	3.5	E
11541100	Milk shake, homemade or fountain-type, NS as to flavor	6000_Dairy	29	26	3	2	1	E
14410200	Cheese, processed, American or Cheddar type	6000_Dairy	29	26	3	4	3	E
11513500	Chocolate milk, made from syrup with whole milk	6000_Dairy	29	24	5	2	2.5	E
11521000	Milk, malted, unfortified, chocolate, made with milk	6000_Dairy	28	30	-2	4	3.5	D
11512510	Hot chocolate, Puerto Rican style, made with low fat milk	6000_Dairy	28	27	1	4	2.5	E
11541000	Milk shake, NS as to flavor or type	6000_Dairy	28	27	1	3	1	E
11511550	Chocolate milk, ready to drink, reduced sugar, NS as to milk	6000_Dairy	28	26	2	4	4.5	B
11511100	Chocolate milk, ready to drink, whole	6000_Dairy	27	28	-1	4	2	E
11511400	Chocolate milk, ready to drink, low fat	6000_Dairy	26	27	-1	4	4	C
11511600	Chocolate milk, ready to drink, low fat (Nesquik)	6000_Dairy	26	27	-1	4	4	C
14502010	Imitation cheese, American or cheddar type	6000_Dairy	25	27	-2	4	4.5	E
11511610	Chocolate milk, ready to drink, fat free (Nesquik)	6000_Dairy	24	27	-3	4	4.5	C
11542100	Milk shake, fast food, chocolate	6000_Dairy	24	24	0	4	1	E
11543000	Milk shake, bottled, chocolate	6000_Dairy	24	24	0	4	1	E
11512500	Hot chocolate, Puerto Rican style, made with whole milk	6000_Dairy	24	22	2	4	1.5	E

11531000	Eggnog	6000_Dairy	24	19	5	4	3	E
11512030	Hot chocolate / Cocoa, ready to drink, made with non-dairy milk	6000_Dairy	23	38	-15	4	2.5	E
11511000	Chocolate milk, NFS	6000_Dairy	23	26	-3	4	4.5	C
11511300	Chocolate milk, ready to drink, fat free	6000_Dairy	23	26	-3	4	4.5	C
14410400	Cheese, processed, Swiss	6000_Dairy	23	18	5	4	2	E
14410100	Cheese, American and Swiss blends	6000_Dairy	23	17	6	4	0.5	E
14420000	Cheese spread, NFS	6000_Dairy	21	18	3	4	2	E
14420100	Cheese spread, American or Cheddar cheese base	6000_Dairy	21	18	3	4	2	E
14420160	Cheese spread, Swiss cheese base	6000_Dairy	21	18	3	4	2	E
14420300	Cheese spread, pressurized can	6000_Dairy	21	18	3	4	2	E
11542000	Carry-out milk shake, NS as to flavor	6000_Dairy	20	22	-2	4	1.5	E
11542200	Milk shake, fast food, flavors other than chocolate	6000_Dairy	20	20	0	4	1.5	E
11543010	Milk shake, bottled, flavors other than chocolate	6000_Dairy	20	20	0	4	1.5	E
11310000	Milk, imitation, fluid, soy based	6000_Dairy	19	28	-9	4	4	D
11560000	Chocolate milk drink	6000_Dairy	18	27	-9	4	3	E
14610200	Cheese, cottage cheese, with gelatin dessert	6000_Dairy	18	13	5	3	4.5	B
11560020	Flavored milk drink, whey- and milk-based, flavors other than chocolate	6000_Dairy	17	26	-9	4	3	E
14410600	Cheese, processed, with vegetables	6000_Dairy	17	9	8	4	2.5	E
11220100	Milk, condensed, sweetened, undiluted	6000_Dairy	16	15	1	3	0.5	E
11220200	Milk, condensed, sweetened, diluted	6000_Dairy	16	15	1	3	0.5	E
11512120	Hot chocolate / Cocoa, ready to drink, made with non-dairy milk and whipped cream	6000_Dairy	15	27	-12	4	1.5	E
11340000	Imitation milk, non-soy, sweetened	6000_Dairy	15	22	-7	4	3.5	C
11220000	Milk, condensed, sweetened	6000_Dairy	15	13	2	3	0.5	E
11514100	Hot chocolate / Cocoa, made with dry mix and water	6000_Dairy	13	22	-9	4	3	E
11512020	Hot chocolate / Cocoa, ready to drink, made with nonfat milk	6000_Dairy	11	19	-8	4	3	E
42402010	Coconut cream, canned, sweetened	6000_Dairy	10	11	-1	3	0.5	E
11512010	Hot chocolate / Cocoa, ready to drink	6000_Dairy	9	11	-2	4	2.5	E
11512110	Hot chocolate / Cocoa, ready to drink, made with nonfat milk and whipped cream	6000_Dairy	8	11	-3	4	2	E
11512100	Hot chocolate / Cocoa, ready to drink, with whipped cream	6000_Dairy	8	10	-2	4	2	E
42401100	Yogurt, coconut milk	6000_Dairy	5	8	-3	4	3	C
13120810	Ice cream soda, chocolate	6000_Dairy	1	6	-5	4	1.5	E
13120800	Ice cream soda, flavors other than chocolate	6000_Dairy	1	4	-3	4	1.5	E
11370000	Coconut milk	6000_Dairy	1	2	-1	4	3	C
82105750	Canola and soybean oil	7000_Fats Oils	78	85	-7	2	3.5	B
82105800	Canola, soybean and sunflower oil	7000_Fats Oils	78	85	-7	2	3.5	C
82101000	Vegetable oil, NFS	7000_Fats Oils	78	84	-6	2	3	B
82105500	Canola oil	7000_Fats Oils	78	84	-6	2	4	B
82108250	Soybean and sunflower oil	7000_Fats Oils	78	83	-5	2	3.5	C

82101300	Almond oil	7000_Fats Oils	78	80	-2	2	4	B
82104000	Olive oil	7000_Fats Oils	78	80	-2	2	3	B
82106000	Safflower oil	7000_Fats Oils	78	80	-2	2	4	B
82108500	Sunflower oil	7000_Fats Oils	78	80	-2	2	3.5	C
82102500	Corn and canola oil	7000_Fats Oils	77	84	-7	2	4	B
82102000	Corn oil	7000_Fats Oils	77	80	-3	2	3.5	C
82103500	Flaxseed oil	7000_Fats Oils	76	83	-7	2	4	B
82108700	Walnut oil	7000_Fats Oils	76	83	-7	2	4	B
82108000	Soybean oil	7000_Fats Oils	76	82	-6	2	3	B
82107000	Sesame oil	7000_Fats Oils	76	78	-2	2	3	C
82105000	Peanut oil	7000_Fats Oils	73	74	-1	2	3	C
82109000	Wheat germ oil	7000_Fats Oils	72	77	-5	2	2.5	C
82103000	Cottonseed oil	7000_Fats Oils	65	64	1	2	1.5	C
83112980	Celery seed dressing	7000_Fats Oils	55	58	-3	2	1	E
81103100	Margarine-like spread, stick, unsalted	7000_Fats Oils	54	67	-13	4	4	C
81103120	Margarine-like spread, tub, unsalted	7000_Fats Oils	54	67	-13	4	4	C
81104070	Margarine-like spread, reduced calorie, about 20% fat, tub, unsalted	7000_Fats Oils	52	60	-8	4	5	B
83105100	Fruit dressing, made with honey, oil, and water	7000_Fats Oils	52	56	-4	2	1.5	D
81103060	Margarine, tub, unsalted	7000_Fats Oils	49	61	-12	4	3.5	C
81103070	Margarine, whipped, tub, unsalted	7000_Fats Oils	49	61	-12	4	3.5	C
83208500	Korean dressing or marinade	7000_Fats Oils	48	49	-1	2	0.5	E
81103030	Margarine, stick, unsalted	7000_Fats Oils	47	57	-10	4	3.5	C
83112600	Cream cheese dressing	7000_Fats Oils	43	44	-1	2	0.5	E
12310100	Sour cream, regular	7000_Fats Oils	40	27	13	2	1	D
83103500	Feta Cheese Dressing	7000_Fats Oils	39	45	-6	4	1	E
12100100	Cream, NS as to light, heavy, or half and half	7000_Fats Oils	39	28	11	2	2.5	D
12120100	Cream, half and half	7000_Fats Oils	39	28	11	2	2.5	D
83112960	Peppercorn Dressing	7000_Fats Oils	38	46	-8	4	0.5	E
83100100	Salad dressing, NFS, for salads	7000_Fats Oils	37	47	-10	4	1	E
83101600	Bacon and tomato dressing	7000_Fats Oils	37	47	-10	4	1.5	E
83104000	French or Catalina dressing	7000_Fats Oils	37	47	-10	4	1.5	E
83112500	Creamy dressing	7000_Fats Oils	37	47	-10	4	1	E
81102020	Margarine, tub	7000_Fats Oils	37	46	-9	4	3	D
81103035	Margarine-oil blend, NFS	7000_Fats Oils	37	46	-9	4	3	D
81103080	Margarine-oil blend, tub	7000_Fats Oils	37	46	-9	4	3	D
12110100	Cream, light	7000_Fats Oils	37	23	14	2	2.5	D
81103090	Butter replacement, liquid	7000_Fats Oils	36	45	-9	4	3	D
81103130	Margarine like spread, whipped, tub, salted	7000_Fats Oils	36	45	-9	4	3	D
83114000	Thousand Island dressing	7000_Fats Oils	36	45	-9	4	1	E

81102000	Margarine, NFS	7000_Fats Oils	36	44	-8	4	3	D
83112000	Avocado dressing	7000_Fats Oils	34	43	-9	4	1.5	E
81104011	Margarine like spread, reduced calorie, about 40% fat, made with yogurt, tub, salted	7000_Fats Oils	33	40	-7	4	4.5	D
81104010	Margarine-oil blend, tub, light	7000_Fats Oils	32	41	-9	4	4	D
81104020	Margarine-oil blend, stick, light	7000_Fats Oils	32	41	-9	4	4	D
83101000	Blue or roquefort cheese dressing	7000_Fats Oils	32	41	-9	4	1	E
83112990	Sesame dressing	7000_Fats Oils	32	41	-9	4	1	E
81105030	Butter-margarine blend, stick, unsalted	7000_Fats Oils	32	38	-6	4	2	D
83102000	Caesar dressing	7000_Fats Oils	32	37	-5	4	0.5	E
14410380	Cream cheese spread, fat free	7000_Fats Oils	32	33	-1	4	4	C
81104110	Margarine like spread, fat free, liquid, salted	7000_Fats Oils	32	33	-1	4	5	D
14303010	Cream cheese, light	7000_Fats Oils	32	22	10	3	0.5	D
81103020	Margarine, whipped, tub, salted	7000_Fats Oils	30	38	-8	4	2.5	D
81102030	Margarine, liquid, salted	7000_Fats Oils	30	36	-6	4	2.5	D
81102010	Margarine, stick	7000_Fats Oils	29	34	-5	4	2	D
81103040	Margarine-oil blend, stick	7000_Fats Oils	29	34	-5	4	2	D
81104050	Margarine like spread, reduced calorie, about 20% fat, tub, salted	7000_Fats Oils	29	32	-3	4	5	C
81100000	Table fat, NFS	7000_Fats Oils	29	22	7	3	0.5	E
81103041	Margarine-like spread, made with yogurt, stick, salted	7000_Fats Oils	28	33	-5	4	3	D
12120105	Cream, half and half, low fat	7000_Fats Oils	28	26	2	4	3.5	D
14301100	Cream cheese, regular, flavored	7000_Fats Oils	28	18	10	3	0.5	D
14301010	Cream cheese, regular, plain	7000_Fats Oils	27	13	14	3	0.5	D
81101100	Butter, stick, unsalted	7000_Fats Oils	27	7	20	2	1	E
81101110	Butter, whipped, tub, unsalted	7000_Fats Oils	27	7	20	2	1	E
83109000	Russian dressing	7000_Fats Oils	26	35	-9	4	1	E
81203000	Shortening, NS as to vegetable or animal	7000_Fats Oils	26	29	-3	4	1.5	C
22621100	Fat back, cooked	7000_Fats Oils	26	14	12	1	0.5	E
81204000	Ghee, clarified butter	7000_Fats Oils	26	7	19	2	1	E
81201000	Animal fat or drippings	7000_Fats Oils	24	13	11	2	0.5	E
83202000	French dressing, low-calorie	7000_Fats Oils	23	31	-8	4	2	D
83105500	Honey mustard dressing	7000_Fats Oils	23	28	-5	4	1.5	E
81104560	Vegetable oil-butter spread, reduced calorie, tub, salted	7000_Fats Oils	23	26	-3	4	3	D
83106000	Italian dressing, made with vinegar and oil	7000_Fats Oils	22	31	-9	4	1.5	E
83205000	Italian dressing, low calorie	7000_Fats Oils	22	30	-8	4	2.5	D
83200100	Salad dressing, light, NFS	7000_Fats Oils	22	28	-6	4	2	D
83210100	Creamy dressing, light	7000_Fats Oils	22	28	-6	4	2	D
81105020	Butter-margarine blend, tub, salted	7000_Fats Oils	22	27	-5	4	2.5	D
81105500	Butter-vegetable oil blend	7000_Fats Oils	22	27	-5	4	2.5	D

12120110	Cream, half and half, fat free	7000_Fats Oils	22	24	-2	4	3.5	D
83202020	French or Catalina dressing, light	7000_Fats Oils	21	30	-9	4	2	D
83201000	Blue or roquefort cheese dressing, light	7000_Fats Oils	21	27	-6	4	2	D
12310370	Sour cream, fat free	7000_Fats Oils	21	20	1	4	4.5	A
81100500	Butter, NFS	7000_Fats Oils	21	5	16	2	0.5	E
81101000	Butter, stick	7000_Fats Oils	21	5	16	2	0.5	E
81101010	Butter, tub	7000_Fats Oils	21	4	17	2	0.5	E
83112950	Poppy seed dressing	7000_Fats Oils	20	29	-9	4	0.5	E
83103000	Coleslaw dressing	7000_Fats Oils	20	26	-6	4	1	E
12120106	Cream, half and half, flavored	7000_Fats Oils	20	16	4	4	3	D
83105000	Fruit dressing, made with fruit juice and cream	7000_Fats Oils	20	15	5	3	2	D
12310300	Sour cream, reduced fat	7000_Fats Oils	19	17	2	4	2.5	D
12310350	Sour cream, light	7000_Fats Oils	19	17	2	4	3	D
12130100	Cream, heavy	7000_Fats Oils	19	13	6	4	1	D
12130200	Cream, heavy, whipped, unsweetened	7000_Fats Oils	19	13	6	4	0.5	D
12210305	Cream substitute, sugar free, liquid	7000_Fats Oils	18	17	1	4	3	C
83201050	Blue or roquefort cheese dressing, reduced calorie	7000_Fats Oils	18	17	1	4	2.5	D
12320200	Sour cream, filled, sour dressing, nonbutterfat	7000_Fats Oils	18	16	2	4	0.5	D
12210280	Coffee creamer, liquid, fat free, sugar free, flavored	7000_Fats Oils	17	17	0	4	3.5	B
12210310	Coffee creamer, liquid, sugar free, flavored	7000_Fats Oils	17	17	0	4	3.5	B
83201200	Blue or roquefort cheese dressing, reduced calorie, fat-free, cholesterol-free	7000_Fats Oils	17	15	2	4	2.5	D
83300100	Blue or roquefort cheese dressing, fat free	7000_Fats Oils	17	15	2	4	2.5	D
12310200	Sour cream, half and half	7000_Fats Oils	17	14	3	4	2.5	D
12110300	Cream, light, whipped, unsweetened	7000_Fats Oils	17	11	6	4	0.5	D
83204500	Honey mustard dressing, light	7000_Fats Oils	16	27	-11	4	2	D
81103140	Margarine-like spread, tub, sweetened	7000_Fats Oils	16	21	-5	4	3.5	D
14420210	Cheese spread, cream cheese, light	7000_Fats Oils	16	12	4	4	0.5	D
83207000	Thousand Island dressing, light	7000_Fats Oils	15	24	-9	4	2	D
12140100	Cream, whipped, pressurized container	7000_Fats Oils	15	11	4	4	0.5	D
12140105	Cream, whipped, pressurized container, light	7000_Fats Oils	15	11	4	4	0.5	D
83206500	Sesame dressing, light	7000_Fats Oils	14	24	-10	4	2	D
83210250	Creamy dressing, made with sour cream and/or buttermilk and oil, reduced calorie, cholesterol-free	7000_Fats Oils	14	16	-2	4	2	D
83112900	Milk, vinegar, and sugar dressing	7000_Fats Oils	14	14	0	2	3	C
83115000	Yogurt dressing	7000_Fats Oils	14	14	0	4	2	D
81101510	Light butter, stick, unsalted	7000_Fats Oils	14	10	4	4	1.5	E
83205450	Italian dressing, light	7000_Fats Oils	13	24	-11	4	2	D
82101500	Coconut oil	7000_Fats Oils	13	3	10	2	1	E
81105010	Butter-margarine blend, stick, salted	7000_Fats Oils	12	13	-1	4	0.5	E

12140000	Cream, whipped	7000_Fats Oils	12	6	6	4	0.5	D
83301000	Thousand Island dressing, fat free	7000_Fats Oils	11	14	-3	4	2.5	D
81104100	Margarine like spread, fat free, tub, salted	7000_Fats Oils	11	10	1	4	5	D
13251000	Chantilly Cream	7000_Fats Oils	11	4	7	4	0.5	D
83201400	Coleslaw dressing, light	7000_Fats Oils	10	19	-9	4	0.5	E
12140110	Whipped topping, dairy based, fat free, pressurized container	7000_Fats Oils	10	17	-7	4	3	D
83205500	Italian dressing, reduced calorie, fat-free	7000_Fats Oils	9	20	-11	4	2	D
83300600	Italian dressing, fat free	7000_Fats Oils	9	20	-11	4	2	D
12220280	Whipped topping, sugar free	7000_Fats Oils	9	5	4	4	0.5	E
14420200	Cheese spread, cream cheese, regular	7000_Fats Oils	9	2	7	4	0.5	D
12210210	Coffee creamer, liquid, flavored	7000_Fats Oils	8	18	-10	4	2	D
83207100	Thousand Island dressing, reduced calorie, fat-free, cholesterol-free	7000_Fats Oils	8	12	-4	4	2.5	D
12210500	Cream substitute, sugar free, powder	7000_Fats Oils	7	7	0	4	1	E
12210505	Coffee creamer,powder, sugar free, flavored	7000_Fats Oils	7	7	0	4	1	E
12320100	Sour cream, imitation	7000_Fats Oils	7	7	0	4	0.5	D
81322000	Honey butter	7000_Fats Oils	7	1	6	2	1	E
12200100	Coffee creamer, NFS	7000_Fats Oils	6	10	-4	4	3	C
12210100	Cream substitute, frozen	7000_Fats Oils	6	10	-4	4	3	C
12210200	Coffee creamer, liquid	7000_Fats Oils	6	10	-4	4	3	C
12210520	Coffee creamer, soy, liquid	7000_Fats Oils	6	10	-4	4	3	C
83203000	Caesar dressing, light	7000_Fats Oils	5	15	-10	4	1.5	E
83300300	Creamy dressing, fat free	7000_Fats Oils	4	3	1	4	2.5	D
83300900	Salad dressing, fat free, NFS	7000_Fats Oils	4	3	1	4	2.5	D
81101500	Light butter, stick, salted	7000_Fats Oils	4	1	3	4	1	E
81101520	Butter, light	7000_Fats Oils	4	1	3	4	1	E
81104550	Butter-oil blend, light	7000_Fats Oils	4	1	3	4	1	E
83206000	Russian dressing, light	7000_Fats Oils	3	14	-11	4	2	D
12210400	Coffee creamer, powder	7000_Fats Oils	3	4	-1	4	1	E
81104490	Butter-oil blend, NFS	7000_Fats Oils	3	1	2	4	0.5	E
81104510	Butter-oil blend, tub	7000_Fats Oils	3	1	2	4	0.5	E
83210200	Creamy dressing, made with sour cream and/or buttermilk and oil, reduced calorie, fat-free, cholesterol-free	7000_Fats Oils	2	3	-1	4	2.5	D
81104500	Butter-oil blend, stick	7000_Fats Oils	2	1	1	4	0.5	E
12210250	Cream substitute, light, liquid	7000_Fats Oils	1	8	-7	4	3.5	B
12210255	Cream substitute, light, flavored, liquid	7000_Fats Oils	1	8	-7	4	3.5	B
12210260	Coffee creamer, liquid, fat free	7000_Fats Oils	1	8	-7	4	3.5	B
12210410	Cream substitute, light, powdered	7000_Fats Oils	1	4	-3	4	0.5	E
12210430	Coffee creamer, powder, fat free	7000_Fats Oils	1	4	-3	4	0.5	E
12210440	Coffee creamer,powder, fat free, flavored	7000_Fats Oils	1	4	-3	4	0.5	E

12210270	Coffee creamer, liquid, fat free, flavored	7000_Fats Oils	1	2	-1	4	2.5	C
12210420	Coffee creamer, powder, flavored	7000_Fats Oils	1	1	0	4	0.5	E
12220000	Whipped topping, nondairy, NS as to canned, frozen, or made from powdered mix	7000_Fats Oils	1	1	0	4	0.5	E
12220100	Whipped topping, nondairy, pressurized can	7000_Fats Oils	1	1	0	4	0.5	E
12220200	Whipped topping	7000_Fats Oils	1	1	0	4	0.5	E
12220250	Whipped topping, nondairy, frozen, lowfat	7000_Fats Oils	1	1	0	4	0.5	E
12220270	Whipped topping, fat free	7000_Fats Oils	1	1	0	4	0.5	E
12220300	Whipped cream substitute, nondairy, made from powdered mix	7000_Fats Oils	1	1	0	4	0.5	D
12220400	Whipped cream substitute, nondairy, lowfat, low sugar, made from powdered mix	7000_Fats Oils	1	1	0	4	2.5	D
81106010	Butter replacement, powder	7000_Fats Oils	1	1	0	4	4	E
83101500	Bacon dressing (hot)	7000_Fats Oils	1	1	0	2	1	E
83113000	Sweet and sour dressing	7000_Fats Oils	1	1	0	4	3.5	C
83202010	French dressing, reduced calorie, fat-free, cholesterol-free	7000_Fats Oils	1	1	0	4	2.5	D
83208000	Vinegar, sugar, and water dressing	7000_Fats Oils	1	1	0	2	2	D
83210000	Creamy dressing, made with sour cream and/or buttermilk and oil, diet, NS as to low or reduced calorie	7000_Fats Oils	1	1	0	4	1	E
83210050	Creamy dressing made with sour cream and/or buttermilk and oil, low calorie	7000_Fats Oils	1	1	0	4	1	E
83220000	Salad dressing, low calorie, oil-free	7000_Fats Oils	1	1	0	4	1.5	E
83300200	Caesar dressing, fat free	7000_Fats Oils	1	1	0	4	1.5	D
83300400	French or Catalina dressing, fat free	7000_Fats Oils	1	1	0	4	2.5	D
83300500	Honey mustard dressing, fat free	7000_Fats Oils	1	1	0	4	1.5	E
27151030	Ceviche	8000_Mixed	100	100	0	1	4	A
27151070	Stewed codfish, no potatoes, Puerto Rican style	8000_Mixed	100	100	0	1	4.5	A
27351030	Stewed codfish, Puerto Rican style	8000_Mixed	100	100	0	1	4.5	A
27450180	Seafood garden salad with seafood, lettuce, vegetables excluding tomato and carrots, no dressing	8000_Mixed	100	100	0	2	4	A
27450190	Seafood garden salad with seafood, lettuce, tomato and/or carrots, other vegetables, no dressing	8000_Mixed	100	100	0	2	4.5	A
27450200	Seafood garden salad with seafood, lettuce, eggs, vegetables excluding tomato and carrots, no dressing	8000_Mixed	100	100	0	2	4	A
27450310	Lomi salmon	8000_Mixed	100	100	0	3	4	A
27450400	Shrimp and vegetables including carrots, broccoli, and/or dark-green leafy; no potatoes, no sauce	8000_Mixed	100	100	0	2	4	A
27450740	Fish and vegetables including carrots, broccoli, and/or dark-green leafy; no potatoes, soy-based sauce	8000_Mixed	100	100	0	2	4	A
63415100	Soup, fruit	8000_Mixed	100	100	0	2	4	A
72125500	Channa Saag	8000_Mixed	100	100	0	1	4.5	A
75146000	Greek Salad, no dressing	8000_Mixed	100	100	0	2	4.5	A
75316010	Zucchini with tomato sauce, cooked, fat not added in cooking	8000_Mixed	100	100	0	2	3.5	B
75440300	Vegetable combinations, including carrots, broccoli, and/or dark-green leafy; cooked, with tomato sauce	8000_Mixed	100	100	0	2	4	A

27450760	Fish shish kabob with vegetables, excluding potatoes	8000_Mixed	100	99	1	1	4	A
27450210	Seafood garden salad with seafood, lettuce, eggs, tomato and/or carrots, other vegetables, no dressing	8000_Mixed	100	98	2	2	4	A
27450410	Shrimp and vegetables including carrots, broccoli, and/or dark-green leafy; no potatoes, soy-based sauce	8000_Mixed	100	98	2	2	4	A
27450600	Shellfish mixture and vegetables including carrots, broccoli, and/or dark-green leafy; no potatoes, soy-based sauce	8000_Mixed	100	98	2	2	4	A
27450750	Fish and vegetables excluding carrots, broccoli, and dark-green leafy; no potatoes, soy-based sauce	8000_Mixed	100	98	2	2	4	A
27451060	Octopus salad, Puerto Rican style	8000_Mixed	100	98	2	2	4	A
27150100	Shrimp curry	8000_Mixed	100	97	3	1	4	A
27150330	Mussels with tomato-based sauce	8000_Mixed	100	96	4	1	4	B
28350120	Crab soup, tomato-base	8000_Mixed	100	96	4	1	3.5	B
27450405	Shrimp and vegetables excluding carrots, broccoli, and dark-green leafy; no potatoes, no sauce	8000_Mixed	100	95	5	2	4	A
27450110	Shrimp garden salad, shrimp, lettuce, eggs, tomato and/or carrots, other vegetables, no dressing	8000_Mixed	100	94	6	2	4	A
27450120	Shrimp garden salad, shrimp, lettuce, eggs, vegetables excluding tomato and carrots, no dressing	8000_Mixed	100	94	6	2	4	A
27450430	Shrimp shish kabob with vegetables, excluding potatoes	8000_Mixed	100	93	7	1	4	A
27450040	Shrimp chow mein or chop suey, no noodles	8000_Mixed	100	90	10	1	4	A
27450420	Shrimp and vegetables excluding carrots, broccoli, and dark-green leafy; no potatoes, soy-based sauce	8000_Mixed	99	93	6	2	4	B
28351110	Fish and vegetable soup, no potatoes, Mexican style	8000_Mixed	99	92	7	1	3.5	A
28355470	Seafood soup with vegetables including carrots, broccoli, and/or dark-green leafy; no potatoes	8000_Mixed	99	92	7	1	3.5	B
28355480	Seafood soup with vegetables excluding carrots, broccoli, and dark-green leafy; no potatoes	8000_Mixed	98	91	7	1	3.5	B
27151040	Crabs in tomato-based sauce, Puerto Rican style	8000_Mixed	98	90	8	2	3.5	C
28355450	Seafood soup with potatoes and vegetables including carrots, broccoli, and/or dark-green leafy	8000_Mixed	98	90	8	1	3.5	A
27350110	Bouillabaisse	8000_Mixed	98	89	9	1	4	A
27450450	Shrimp creole, no rice	8000_Mixed	98	89	9	2	4	A
41221020	Chili with beans, without meat	8000_Mixed	97	97	0	3	4	A
27450610	Shellfish mixture and vegetables excluding carrots, broccoli, and dark-green leafy; no potatoes, soy-based sauce	8000_Mixed	97	92	5	2	4	A
73111400	Carrots in tomato sauce	8000_Mixed	97	92	5	2	4	B
27450470	Kung Pao shrimp	8000_Mixed	97	90	7	2	4	A
27451030	Lobster with sauce, Puerto Rican style	8000_Mixed	97	89	8	2	3.5	C
28355460	Seafood soup with potatoes, and vegetables excluding carrots, broccoli, and dark-green leafy	8000_Mixed	97	89	8	1	3.5	A
75232050	Seaweed, prepared with soy sauce	8000_Mixed	96	90	6	1	3.5	C
72306000	Watercress broth with shrimp	8000_Mixed	96	87	9	1	3.5	B
72125253	Spinach, cooked, from canned, with cheese sauce	8000_Mixed	95	99	-4	2	3.5	B
27450700	Fish and vegetables including carrots, broccoli, and/or dark-green leafy; no potatoes, tomato-based sauce	8000_Mixed	95	96	-1	2	3.5	B

58146672	Pasta, whole grain, with tomato-based sauce, seafood, and added vegetables, home recipe	8000_Mixed	95	92	3	2	4	B
27250060	Gefilte fish	8000_Mixed	95	85	10	1	4	A
75316050	Ratatouille	8000_Mixed	94	96	-2	2	4.5	A
75306010	Eggplant in tomato sauce, cooked, fat not added in cooking	8000_Mixed	94	90	4	2	4.5	A
28351120	Fish soup with potatoes, Mexican style	8000_Mixed	94	87	7	1	3.5	A
75604600	Gazpacho	8000_Mixed	93	98	-5	2	4	A
72307000	Spinach soup	8000_Mixed	93	96	-3	2	3.5	A
27251010	Stewed salmon, Puerto Rican style	8000_Mixed	93	89	4	2	4	A
72125251	Spinach, cooked, from fresh, with cheese sauce	8000_Mixed	92	97	-5	3	3.5	B
41311020	Sambar, vegetable stew	8000_Mixed	92	92	0	1	4	A
41603010	Lentil soup, home recipe, canned, or ready-to-serve	8000_Mixed	91	88	3	1	4	A
58146662	Pasta, whole grain, with tomato-based sauce and seafood, home recipe	8000_Mixed	91	88	3	2	4	B
27446300	Chicken or turkey garden salad, chicken and/or turkey, tomato and/or carrots, other vegetables, no dressing	8000_Mixed	91	87	4	1	4.5	A
75147000	Spinach salad, no dressing	8000_Mixed	91	87	4	2	4.5	A
27350310	Seafood stew with potatoes and vegetables including carrots, broccoli, and/or dark-green leafy; tomato-based sauce	8000_Mixed	91	79	12	1	4	A
27250220	Oyster fritter	8000_Mixed	90	90	0	3	3	C
27446360	Chicken or turkey caesar garden salad, chicken and/or turkey, lettuce, tomato, cheese, no dressing	8000_Mixed	90	89	1	1	4.5	A
27446310	Chicken or turkey garden salad, chicken and/or turkey, other vegetables excluding tomato and carrots, no dressing	8000_Mixed	90	86	4	1	4.5	A
27350030	Seafood stew with potatoes and vegetables excluding carrots, broccoli, and dark-green leafy; tomato-based sauce	8000_Mixed	90	79	11	2	4	A
27446355	Asian chicken or turkey garden salad with crispy noodles, chicken and/or turkey, lettuce, fruit, nuts, crispy noodles, no dressing	8000_Mixed	89	88	1	1	4.5	A
27250040	Crab cake	8000_Mixed	89	85	4	2	3.5	C
27260550	Liver hash	8000_Mixed	89	83	6	1	4.5	A
27150310	Fish with tomato-based sauce	8000_Mixed	89	78	11	1	4	A
28355260	Lobster gumbo	8000_Mixed	89	78	11	1	3.5	A
72125231	Spinach, from fresh, creamed	8000_Mixed	88	90	-2	1	3.5	B
58146612	Pasta, whole grain, with tomato-based sauce and added vegetables, home recipe	8000_Mixed	88	89	-1	2	4	B
27445180	Moo Goo Gai Pan	8000_Mixed	88	87	1	1	4.5	A
27450710	Fish and vegetables excluding carrots, broccoli, and dark- green leafy; no potatoes, tomato-based sauce	8000_Mixed	88	87	1	2	3.5	B
28350310	Turtle and vegetable soup	8000_Mixed	88	83	5	1	3.5	B
72125310	Palak Paneer	8000_Mixed	87	93	-6	2	4	A
27250400	Shrimp cake or patty	8000_Mixed	87	81	6	2	4	A
27361010	Stewed variety meats, mostly liver, Puerto Rican style	8000_Mixed	87	80	7	1	4	A
72125252	Spinach, cooked, from frozen, with cheese sauce	8000_Mixed	86	91	-5	2	3.5	C
75403200	Green beans, cooked, Szechuan-style	8000_Mixed	86	90	-4	2	4.5	A

27141500	Chili con carne with chicken or turkey and beans	8000_Mixed	86	83	3	2	4	A
32101530	Egg curry	8000_Mixed	86	82	4	1	4	A
27460750	Liver, beef or calves, and onions	8000_Mixed	86	80	6	1	4	A
72125250	Spinach, cooked, NS as to form, with cheese sauce	8000_Mixed	85	90	-5	2	3.5	C
27450150	Fish, tofu, and vegetables, tempura	8000_Mixed	85	87	-2	2	4	B
58146602	Pasta, whole grain, with tomato-based sauce, home recipe	8000_Mixed	85	86	-1	2	4	B
75201030	Artichoke salad in oil	8000_Mixed	85	86	-1	3	5	A
58161321	Kidney beans and brown rice	8000_Mixed	85	85	0	1	4.5	A
58146652	Pasta, whole grain, with tomato-based sauce, poultry, and added vegetables, home recipe	8000_Mixed	85	84	1	1	4	B
58161322	Black beans and brown rice	8000_Mixed	85	84	1	1	4.5	A
58161323	Pinto beans and brown rice	8000_Mixed	85	84	1	1	4.5	A
58161325	Rice, brown, with beans and tomatoes	8000_Mixed	85	84	1	1	4	A
72116140	Caesar salad (with romaine)	8000_Mixed	85	81	4	2	4	A
75440100	Vegetable combination, including carrots, broccoli, and/or dark-green leafy; cooked, with soy-based sauce	8000_Mixed	84	89	-5	2	3.5	C
27150320	Fish curry	8000_Mixed	84	86	-2	2	3.5	B
58161320	Beans and brown rice	8000_Mixed	84	84	0	1	4	A
75450600	Vegetable combination, including carrots, broccoli, and/or dark-green leafy; cooked, with butter sauce	8000_Mixed	84	84	0	2	4	A
27250128	Shrimp and noodles with soy-based sauce	8000_Mixed	84	75	9	2	3.5	B
72125232	Spinach, from frozen, creamed	8000_Mixed	83	85	-2	1	4	B
58132350	Spaghetti with tomato sauce, meatless, whole wheat noodles	8000_Mixed	83	84	-1	2	3.5	C
58146310	Pasta, whole wheat, with tomato sauce, meatless	8000_Mixed	83	84	-1	2	3.5	C
75439500	Chow mein or chop suey, meatless, no noodles	8000_Mixed	83	84	-1	1	4	A
41601080	Pinto bean soup, home recipe, canned or ready-to-serve	8000_Mixed	83	79	4	1	5	A
27150350	Sardines with tomato-based sauce	8000_Mixed	83	77	6	3	4	B
27150370	Sardines with mustard sauce	8000_Mixed	83	77	6	3	4	B
41812500	Tofu and vegetables including carrots, broccoli, and/or dark-green leafy; no potatoes, with soy-based sauce	8000_Mixed	82	88	-6	2	3.5	B
27450010	Crab salad	8000_Mixed	82	84	-2	3	3	C
58175000	Vada, fried dumpling	8000_Mixed	82	83	-1	1	5	A
27450020	Lobster salad	8000_Mixed	82	82	0	3	3	C
27250070	Salmon cake or patty	8000_Mixed	82	80	2	3	3.5	D
27250160	Tuna cake or patty	8000_Mixed	82	79	3	3	3.5	B
75417030	Peas, cooked, NS as to form, with tomato sauce	8000_Mixed	82	76	6	1	4.5	A
75417032	Peas, cooked, from frozen, with tomato sauce	8000_Mixed	82	76	6	1	4.5	A
27250250	Flounder with crab stuffing	8000_Mixed	82	73	9	2	4	B
27451010	Fried fish with sauce, Puerto Rican style	8000_Mixed	82	72	10	2	3.5	C
27150030	Crab imperial	8000_Mixed	82	71	11	2	3.5	C
28160810	Livers, chicken, with vegetable (diet frozen meal)	8000_Mixed	81	86	-5	4	4.5	A

41601050	Soybean soup, made with milk	8000_Mixed	81	84	-3	1	3.5	B
27445125	Chicken or turkey and vegetables including carrots, broccoli, and/or dark-green leafy; no potatoes, tomato-based sauce	8000_Mixed	81	83	-2	2	3.5	B
58146642	Pasta, whole grain, with tomato-based sauce and poultry, home recipe	8000_Mixed	81	80	1	1	4	B
58161474	Rice, brown, with dark green vegetables, fat added	8000_Mixed	81	80	1	1	3.5	B
58161494	Rice, brown, with dark green vegetables and tomatoes and/or tomato-based sauce, fat added	8000_Mixed	81	80	1	1	3.5	B
27446350	Asian chicken or turkey garden salad, chicken and/or turkey, lettuce, fruit, nuts, no dressing	8000_Mixed	81	79	2	1	4.5	A
27351010	Codfish with starchy vegetables, Puerto Rican style	8000_Mixed	81	78	3	2	5	A
75440310	Vegetable combinations, excluding carrots, broccoli, and dark-green leafy; cooked, with tomato sauce	8000_Mixed	81	76	5	1	4	A
75148010	Cobb salad, no dressing	8000_Mixed	81	74	7	1	4	A
27351060	Shrimp and pasta garden salad (shrimp, pasta salad, tomato and/or carrots, other vegetables), no dressing	8000_Mixed	81	73	8	2	4	A
75647000	Seaweed soup	8000_Mixed	81	72	9	2	3	C
28355440	Shrimp gumbo	8000_Mixed	81	68	13	2	3.5	B
41812450	Vegetarian chili, made with meat substitute	8000_Mixed	80	89	-9	4	5	A
58161470	Rice, brown, with dark green vegetables, NS as to fat	8000_Mixed	80	80	0	1	3.5	B
27351040	Biscayne codfish, Puerto Rican style	8000_Mixed	80	77	3	2	4	A
27450650	Shellfish mixture and vegetables including carrots, broccoli, and/or dark-green leafy; no potatoes, mushroom sauce	8000_Mixed	80	77	3	3	3.5	C
27351050	Codfish salad, Puerto Rican style (Ensalada de bacalao)	8000_Mixed	80	76	4	2	4	B
28350040	Fish stock, home recipe	8000_Mixed	80	69	11	1	3.5	B
27150410	Shrimp teriyaki	8000_Mixed	80	68	12	1	4	B
41601070	Soybean soup, miso broth	8000_Mixed	79	80	-1	3	3	C
72125233	Spinach, from canned, creamed	8000_Mixed	79	80	-1	1	3.5	C
58161435	Rice, brown, with carrots, NS as to fat	8000_Mixed	79	78	1	1	3.5	C
58161439	Rice, brown, with carrots, fat added	8000_Mixed	79	78	1	1	3.5	C
58161490	Rice, brown, with dark green vegetables and tomatoes and/or tomato-based sauce, NS as to fat	8000_Mixed	79	78	1	1	3.5	B
27250210	Clam cake or patty	8000_Mixed	79	77	2	3	3.5	C
27450067	Tuna salad, made with light Italian dressing	8000_Mixed	79	77	2	3	3.5	B
41812510	Tofu and vegetables excluding carrots, broccoli, and dark-green leafy; no potatoes, with soy-based sauce	8000_Mixed	78	83	-5	2	3.5	B
75440110	Vegetable combination, excluding carrots, broccoli, and dark-green leafy; cooked, with soy-based sauce	8000_Mixed	78	83	-5	2	3.5	C
58146601	Pasta, whole grain, with tomato-based sauce, restaurant	8000_Mixed	78	81	-3	2	3.5	C
27420100	Pork, tofu, and vegetables including carrots, broccoli, and/or dark-green leafy; no potatoes, soy-base sauce	8000_Mixed	78	80	-2	2	4	B
41311030	Lentil curry	8000_Mixed	78	80	-2	2	4	A
75401011	Asparagus, from fresh, creamed or with cheese sauce	8000_Mixed	78	79	-1	2	3.5	B
58161464	Rice, brown, with tomatoes and/or tomato based sauce, fat added	8000_Mixed	78	78	0	1	3.5	C
58161510	Grape leaves stuffed with rice	8000_Mixed	78	78	0	2	4.5	A

58175110	Tabbouleh	8000_Mixed	78	78	0	2	3	C
75401010	Asparagus, NS as to form, creamed or with cheese sauce	8000_Mixed	78	78	0	2	3.5	B
58161460	Rice, brown, with tomatoes and/or tomato based sauce, NS as to fat	8000_Mixed	78	77	1	1	3.5	C
58161480	Rice, brown, with carrots and tomatoes and/or tomato-based sauce, NS as to fat	8000_Mixed	78	77	1	1	3.5	C
58161484	Rice, brown, with carrots and tomatoes and/or tomato-based sauce, fat added	8000_Mixed	78	77	1	1	3.5	C
58161500	Rice, brown, with carrots and dark green vegetables, NS as to fat	8000_Mixed	78	77	1	1	3.5	B
58161504	Rice, brown, with carrots and dark green vegetables, fat added	8000_Mixed	78	77	1	1	3.5	B
58161520	Rice, brown, with carrots, dark green vegetables, and tomatoes and/or tomato-based sauce, NS as to fat	8000_Mixed	78	77	1	1	3.5	C
58161524	Rice, brown, with carrots, dark green vegetables, and tomatoes and/or tomato-based sauce, fat added	8000_Mixed	78	77	1	1	3.5	C
58161472	Rice, brown, with dark green vegetables, no added fat	8000_Mixed	78	76	2	1	3.5	B
58146632	Pasta, whole grain, with tomato-based sauce, meat, and added vegetables, home recipe	8000_Mixed	78	75	3	1	4	B
75601200	Cabbage soup, home recipe, canned or ready-to-serve	8000_Mixed	78	75	3	2	3.5	B
28340150	Mexican style chicken broth soup stock	8000_Mixed	78	74	4	1	4	B
27450250	Oysters Rockefeller	8000_Mixed	78	71	7	2	3	C
28355350	Salmon soup, cream style	8000_Mixed	78	70	8	2	3.5	B
28350110	Crab soup, NS as to tomato-base or cream style	8000_Mixed	78	68	10	1	3.5	B
28355210	Crab soup, cream of, prepared with milk	8000_Mixed	78	68	10	1	3.5	B
75141025	Cabbage salad or coleslaw, made with light Italian dressing	8000_Mixed	77	80	-3	3	4	A
27415110	Beef and broccoli	8000_Mixed	77	79	-2	2	4	A
27450080	Seafood salad	8000_Mixed	77	77	0	4	3	D
75401012	Asparagus, from frozen, creamed or with cheese sauce	8000_Mixed	77	77	0	2	3.5	B
27450063	Tuna salad, made with light mayonnaise-type salad dressing	8000_Mixed	77	76	1	3	3.5	B
27341510	Chicken or turkey stew with potatoes and vegetables including carrots, broccoli, and/or dark-green leafy; tomato-based sauce	8000_Mixed	77	72	5	1	4	A
27441120	Chicken or turkey creole, without rice	8000_Mixed	77	71	6	1	4	A
27150250	Fish moochim	8000_Mixed	77	68	9	2	0.5	E
27250410	Shrimp with crab stuffing	8000_Mixed	77	66	11	2	3.5	C
28355250	Lobster bisque	8000_Mixed	77	66	11	1	3.5	B
32105200	Egg foo yung, NFS	8000_Mixed	77	64	13	1	4	B
32105230	Shrimp egg foo yung	8000_Mixed	77	64	13	1	4	B
58146641	Pasta, whole grain, with tomato-based sauce and poultry, restaurant	8000_Mixed	76	79	-3	2	3.5	C
27415120	Beef, tofu, and vegetables including carrots, broccoli, and/or dark-green leafy; no potatoes, soy-based sauce	8000_Mixed	76	77	-1	2	4	A
27420400	Pork and vegetables including carrots, broccoli, and/or dark-green leafy; no potatoes, tomato-based sauce	8000_Mixed	76	76	0	2	3.5	B
27450061	Tuna salad, made with light mayonnaise	8000_Mixed	76	76	0	3	3.5	B
27411100	Beef with vegetables including carrots, broccoli, and/or dark-green leafy; no potatoes, tomato-based sauce	8000_Mixed	76	75	1	2	4	B

27446330	Chicken or turkey garden salad with cheese, chicken and/or turkey, cheese, lettuce and/or greens, tomato and/or carrots, other vegetables, no dressing	8000_Mixed	76	75	1	2	4	A
58146622	Pasta, whole grain, with tomato-based sauce and meat, home recipe	8000_Mixed	76	75	1	1	4	B
58161437	Rice, brown, with carrots, no added fat	8000_Mixed	76	73	3	1	3.5	C
58161482	Rice, brown, with carrots and tomatoes and/or tomato-based sauce, no added fat	8000_Mixed	76	73	3	1	3.5	C
58161492	Rice, brown, with dark green vegetables and tomatoes and/or tomato-based sauce, no added fat	8000_Mixed	76	73	3	1	3.5	B
58161502	Rice, brown, with carrots and dark green vegetables, no added fat	8000_Mixed	76	73	3	1	3.5	B
75649110	Vegetable soup, home recipe	8000_Mixed	76	73	3	2	3.5	B
27241000	Chicken or turkey hash	8000_Mixed	76	72	4	1	4.5	A
77316600	Eggplant and meat casserole	8000_Mixed	76	70	6	1	4	A
75302080	Bean salad, yellow and/or green string beans	8000_Mixed	75	79	-4	2	4	B
27550200	Fish sandwich, from school cafeteria	8000_Mixed	75	76	-1	3	4	A
75141035	Cabbage salad or coleslaw, made with light creamy dressing	8000_Mixed	75	75	0	3	4	A
75401013	Asparagus, from canned, creamed or with cheese sauce	8000_Mixed	75	75	0	2	3.5	C
41601200	Liquid from stewed kidney beans, Puerto Rican style	8000_Mixed	75	74	1	1	3.5	B
58161440	Rice, brown, with peas and carrots, NS as to fat	8000_Mixed	75	74	1	1	3.5	B
58161444	Rice, brown, with peas and carrots, fat added	8000_Mixed	75	74	1	1	3.5	B
58161530	Rice, brown, with other vegetables, NS as to fat	8000_Mixed	75	73	2	1	3.5	B
58161534	Rice, brown, with other vegetables, fat added	8000_Mixed	75	73	2	1	3.5	B
58161462	Rice, brown, with tomatoes and/or tomato based sauce, no added fat	8000_Mixed	75	72	3	1	3.5	C
58161522	Rice, brown, with carrots, dark green vegetables, and tomatoes and/or tomato-based sauce, no added fat	8000_Mixed	75	72	3	1	3.5	B
28340580	Chicken or turkey soup with vegetables, broccoli, carrots, celery, potatoes and onions, Asian style	8000_Mixed	75	71	4	1	3.5	C
58161402	Rice, brown, with vegetables (including carrots, broccoli, and/or dark-green leafy), no sauce, fat not added in cooking	8000_Mixed	75	71	4	1	3.5	C
58403100	Noodle and potato soup, Puerto Rican style	8000_Mixed	75	70	5	1	3.5	B
27136100	Chili con carne with venison/deer and beans	8000_Mixed	75	69	6	2	4.5	A
27341520	Chicken or turkey stew with potatoes and vegetables excluding carrots, broccoli, and dark-green leafy; tomato- based sauce	8000_Mixed	75	69	6	1	4.5	A
27350040	Shad creole, with rice	8000_Mixed	75	65	10	1	3.5	C
27350050	Shrimp chow mein or chop suey with noodles	8000_Mixed	75	63	12	1	4	B
27450064	Tuna salad, made with creamy dressing	8000_Mixed	74	77	-3	4	3.5	C
75440600	Vegetable curry	8000_Mixed	74	77	-3	2	3.5	B
27450066	Tuna salad, made with Italian dressing	8000_Mixed	74	75	-1	3	3.5	C
27445130	Chicken or turkey and vegetables excluding carrots, broccoli, and dark-green leafy; no potatoes, tomato-based sauce	8000_Mixed	74	74	0	2	3.5	B
27450062	Tuna salad, made with mayonnaise-type salad dressing	8000_Mixed	74	74	0	4	3.5	C
58146300	Pasta, whole wheat, with meat sauce	8000_Mixed	74	73	1	1	3.5	B

58161400	Rice, brown, with vegetables (including carrots, broccoli, and/or dark-green leafy), no sauce, NS as to fat added in cooking	8000_Mixed	74	73	1	1	3.5	C
58161404	Rice, brown, with vegetables (including carrots, broccoli, and/or dark-green leafy), no sauce, fat added in cooking	8000_Mixed	74	73	1	1	3.5	C
75612010	Zucchini soup, cream of, prepared with milk	8000_Mixed	74	73	1	1	3.5	B
27120160	Pork curry	8000_Mixed	74	72	2	1	3.5	B
58160800	Rice, white, with lentils, NS as to fat	8000_Mixed	74	72	2	1	4	A
58160805	Rice, white, with lentils, fat added	8000_Mixed	74	72	2	1	4	A
58161310	Rice, brown, with tomato sauce	8000_Mixed	74	72	2	1	3	C
58161430	Rice, brown, with peas, NS as to fat	8000_Mixed	74	72	2	1	3.5	B
58161434	Rice, brown, with peas, fat added	8000_Mixed	74	72	2	1	3.5	B
27451070	Codfish salad, Puerto Rican style, Serenata	8000_Mixed	74	71	3	2	3	C
58161452	Rice, brown, with tomatoes, fat not added in cooking	8000_Mixed	74	70	4	1	3.5	C
28310160	Beef broth, with tomato, home recipe	8000_Mixed	73	77	-4	2	3.5	C
27540240	Chicken fillet, broiled, sandwich, on whole wheat roll, with lettuce, tomato and spread	8000_Mixed	73	75	-2	3	4	B
28150210	Haddock with chopped spinach, diet frozen meal	8000_Mixed	73	73	0	4	4	A
27445120	Chicken or turkey and vegetables excluding carrots, broccoli, and dark-green leafy; no potatoes, soy-based sauce	8000_Mixed	73	72	1	2	4	B
58132360	Spaghetti with tomato sauce and meatballs, whole wheat noodles or spaghetti with meat sauce, whole wheat noodles or spaghetti with meat sauce and meatballs, whole wheat noodles	8000_Mixed	73	72	1	2	3.5	C
27445110	Chicken or turkey and vegetables including carrots, broccoli, and/or dark-green leafy; no potatoes, soy-based sauce	8000_Mixed	73	71	2	1	3.5	B
58160150	Kidney beans and white rice	8000_Mixed	73	71	2	1	4	A
27450068	Tuna salad, made with any type of fat free dressing	8000_Mixed	73	70	3	3	3.5	B
58160156	Pinto beans and white rice	8000_Mixed	73	70	3	1	4	A
27450660	Shellfish mixture and vegetables excluding carrots, broccoli, and dark-green leafy; no potatoes, mushroom sauce	8000_Mixed	73	69	4	2	3.5	C
75601210	Cabbage with meat soup, home recipe, canned or ready-to-serve	8000_Mixed	73	69	4	1	3.5	B
75651000	Minestrone soup, home recipe	8000_Mixed	73	69	4	1	4	B
27151050	Shrimp in garlic sauce, Puerto Rican style	8000_Mixed	73	68	5	2	3	D
27150010	Fish with cream or white sauce, not tuna or lobster	8000_Mixed	73	61	12	1	4	A
58134310	Stuffed shells, with fish and/or shellfish, with tomato sauce	8000_Mixed	73	59	14	1	4	B
73101110	Carrots, raw, salad	8000_Mixed	72	77	-5	3	3.5	C
27450030	Salmon salad	8000_Mixed	72	76	-4	4	3	C
28340130	Chicken or turkey broth, with tomato, home recipe	8000_Mixed	72	76	-4	2	3.5	C
27420370	Pork, tofu, and vegetables, excluding carrots, broccoli, and dark-green leafy; no potatoes, soy-based sauce	8000_Mixed	72	74	-2	2	3.5	B
27341055	Chicken or turkey, potatoes, and vegetables including carrots, broccoli, and/or dark-green leafy; tomato-based sauce	8000_Mixed	72	72	0	2	4	B
27330170	Stuffed grape leaves with lamb and rice	8000_Mixed	72	71	1	1	4.5	A
28150220	Flounder with chopped broccoli, diet frozen meal	8000_Mixed	72	71	1	4	4	A
58161450	Rice, brown, with tomatoes, NS as to fat added in cooking	8000_Mixed	72	71	1	1	3.5	C

58161454	Rice, brown, with tomatoes, fat added in cooking	8000_Mixed	72	71	1	1	3.5	C
27420500	Pork and vegetables including carrots, broccoli, and/or dark-green leafy; no potatoes, soy-based sauce	8000_Mixed	72	70	2	1	4	B
58160154	Black beans and white rice	8000_Mixed	72	70	2	1	4.5	A
58161420	Rice, brown, with corn, NS as to fat	8000_Mixed	72	70	2	1	3.5	B
58161424	Rice, brown, with corn, fat added	8000_Mixed	72	70	2	1	3.5	B
58161442	Rice, brown, with peas and carrots, no added fat	8000_Mixed	72	68	4	1	3.5	B
58161532	Rice, brown, with other vegetables, no added fat	8000_Mixed	72	68	4	1	3.5	B
27420110	Pork and vegetables, Hawaiian style	8000_Mixed	72	67	5	1	4	A
73501000	Carrot soup, cream of, prepared with milk, home recipe, canned or ready-to-serve	8000_Mixed	72	67	5	1	3.5	B
27440130	Chicken or turkey shish kabob with vegetables, excluding potatoes	8000_Mixed	72	65	7	1	4	A
27150120	Tuna with cream or white sauce	8000_Mixed	72	63	9	3	4	A
28351170	Codfish soup with noodles, Puerto Rican style	8000_Mixed	72	63	9	2	3.5	B
58155320	Seafood paella, Puerto Rican style	8000_Mixed	72	62	10	1	3.5	A
27415220	Beef, tofu, and vegetables excluding carrots, broccoli, and dark-green leafy; no potatoes, soy-based sauce	8000_Mixed	71	73	-2	2	3.5	A
27450100	Tuna salad with egg	8000_Mixed	71	73	-2	4	3.5	C
72201241	Broccoli, cooked, from fresh, with mushroom sauce	8000_Mixed	71	72	-1	2	3.5	B
58160106	Pinto beans and rice, from fast food / restaurant	8000_Mixed	71	70	1	1	4	B
75141040	Cabbage salad or coleslaw, made with any type of fat free dressing	8000_Mixed	71	70	1	2	4.5	A
27130100	Lamb or mutton curry	8000_Mixed	71	69	2	1	3.5	C
27415100	Beef and vegetables including carrots, broccoli, and/or dark-green leafy; no potatoes, soy-based sauce	8000_Mixed	71	69	2	1	4	A
27440110	Chicken or turkey and vegetables including carrots, broccoli, and/or dark-green leafy; no potatoes, no sauce	8000_Mixed	71	69	2	1	3.5	B
27445250	Almond chicken	8000_Mixed	71	69	2	1	4	B
58160104	Black beans and rice, from fast food / restaurant	8000_Mixed	71	69	2	1	4	B
58161405	Rice, brown, with vegetables (excluding carrots, broccoli, and dark-green leafy), no sauce, NS as to fat added in cooking	8000_Mixed	71	69	2	1	3.5	B
58161409	Rice, brown, with vegetables (excluding carrots, broccoli, and dark-green leafy), no sauce, fat added in cooking	8000_Mixed	71	69	2	1	3.5	B
27420060	Pork and vegetables including carrots, broccoli, and/or dark-green leafy; no potatoes, no sauce	8000_Mixed	71	68	3	1	4	A
75148000	Cobb salad with dressing	8000_Mixed	71	68	3	2	4	B
58132810	Spaghetti with red clam sauce	8000_Mixed	71	67	4	2	4	B
58161407	Rice, brown, with vegetables (excluding carrots, broccoli, and dark-green leafy), no sauce, fat not added in cooking	8000_Mixed	71	67	4	1	3.5	B
72201250	Broccoli, cooked, NS as to form, with cream sauce	8000_Mixed	71	67	4	1	4	A
58150100	Bibimbap, Korean	8000_Mixed	71	66	5	1	4	A
27450065	Tuna salad, made with light creamy dressing	8000_Mixed	71	65	6	3	3.5	B
27460010	Chow mein or chop suey, NS as to type of meat, no noodles	8000_Mixed	71	65	6	1	4	A
58160810	Rice, white, with lentils, no added fat	8000_Mixed	71	64	7	1	4	A
27250132	Shrimp and noodles with tomato sauce	8000_Mixed	71	63	8	1	4	B

27250080	Salmon loaf	8000_Mixed	71	62	9	3	3	D
28351160	Codfish, rice, and vegetable soup, Puerto Rican style	8000_Mixed	71	62	9	2	3.5	B
58146215	Pasta with sauce, meatless, school lunch	8000_Mixed	70	79	-9	4	4	B
73101210	Carrots, raw, salad with apples	8000_Mixed	70	78	-8	3	4	B
75141020	Cabbage salad or coleslaw, made with Italian dressing	8000_Mixed	70	77	-7	3	4	A
27450060	Tuna salad, made with mayonnaise	8000_Mixed	70	74	-4	4	3.5	C
27146150	Chicken curry	8000_Mixed	70	71	-1	2	3.5	B
58160102	Kidney beans and rice, from fast food / restaurant	8000_Mixed	70	70	0	1	4	A
27320100	Pork, potatoes, and vegetables including carrots, broccoli, and/or dark-green leafy; tomato-based sauce	8000_Mixed	70	69	1	2	4	A
27420410	Pork and vegetables excluding carrots, broccoli, and dark-green leafy; no potatoes, tomato-based sauce	8000_Mixed	70	69	1	2	3.5	B
72201240	Broccoli, cooked, NS as to form, with mushroom sauce	8000_Mixed	70	69	1	2	4	B
72201242	Broccoli, cooked, from frozen, with mushroom sauce	8000_Mixed	70	69	1	2	4	B
27345310	Chicken or turkey, rice, and vegetables including carrots, broccoli, and/or dark-green leafy; soy-based sauce	8000_Mixed	70	68	2	1	4	B
27446315	Chicken or turkey garden salad with bacon and cheese, chicken and/or turkey, bacon, cheese, lettuce and/or greens, tomato and/or carrots, other vegetables, no dressing	8000_Mixed	70	68	2	2	4	A
58160110	Beans and white rice	8000_Mixed	70	68	2	1	4	A
41812800	Vegetarian stew	8000_Mixed	70	67	3	2	3.5	B
27410210	Beef and vegetables including carrots, broccoli, and/or dark-green leafy; no potatoes, no sauce	8000_Mixed	70	66	4	1	4	B
58161432	Rice, brown, with peas, no added fat	8000_Mixed	70	66	4	1	4	B
27250950	Shellfish and noodles with tomato-based sauce	8000_Mixed	70	65	5	2	4	B
27443150	Chicken or turkey divan	8000_Mixed	70	65	5	2	4	A
28340660	Chicken or turkey vegetable soup, home recipe	8000_Mixed	70	65	5	1	3.5	B
27415150	Beef chow mein or chop suey, no noodles	8000_Mixed	70	64	6	1	4	A
27446100	Chicken or turkey chow mein or chop suey, no noodles	8000_Mixed	70	64	6	1	4	A
27464000	Gumbo, no rice	8000_Mixed	70	64	6	1	3.5	C
28340670	Chicken or turkey vegetable soup with rice, home recipe, Mexican style	8000_Mixed	70	63	7	1	4	A
27250030	Codfish ball or cake	8000_Mixed	70	62	8	2	4	B
28311030	Menudo soup, canned, prepared with water or ready-to-serve	8000_Mixed	70	59	11	2	3.5	C
41209000	Falafel	8000_Mixed	69	73	-4	2	4	D
72125260	Spinach and cheese casserole	8000_Mixed	69	73	-4	3	3.5	C
27116100	Beef curry	8000_Mixed	69	69	0	2	4	B
72201231	Broccoli, cooked, from fresh, with cheese sauce	8000_Mixed	69	69	0	2	3.5	C
27341060	Chicken or turkey, potatoes, and vegetables excluding carrots, broccoli, and dark-green leafy; tomato-based sauce	8000_Mixed	69	68	1	2	4	B
27510511	Hamburger, 1 miniature patty, on miniature bun, from school	8000_Mixed	69	68	1	3	4	A
58160100	Beans and rice, from fast food / restaurant	8000_Mixed	69	68	1	1	4	B
75412070	Eggplant with cheese and tomato sauce	8000_Mixed	69	68	1	3	4.5	A

27315270	Stuffed grape leaves with beef and rice	8000_Mixed	69	67	2	1	4.5	A
27411200	Beef with vegetables excluding carrots, broccoli, and dark-green leafy; no potatoes, tomato-based sauce	8000_Mixed	69	67	2	2	3.5	B
27420510	Pork and vegetables excluding carrots, broccoli, and dark- green leafy; no potatoes, soy-based sauce	8000_Mixed	69	67	2	1	4	B
27445220	Kung pao chicken	8000_Mixed	69	67	2	1	3.5	C
27540250	Chicken fillet, broiled, sandwich with cheese, on whole wheat roll, with lettuce, tomato and non-mayonnaise type spread	8000_Mixed	69	67	2	3	3.5	C
27260510	Liver dumpling	8000_Mixed	69	66	3	2	2	D
27341010	Chicken or turkey, potatoes, and vegetables including carrots, broccoli, and/or dark-green leafy; no sauce	8000_Mixed	69	66	3	1	4	B
58160120	Beans and rice, with tomatoes	8000_Mixed	69	66	3	1	4	B
75450500	Vegetable combination, including carrots, broccoli, and/or dark-green leafy; cooked, with cream sauce	8000_Mixed	69	66	3	1	3.5	B
77250710	Tannier fritters, Puerto Rican style	8000_Mixed	69	66	3	2	4.5	A
28150240	Sole with vegetable (diet frozen meal)	8000_Mixed	69	65	4	4	3.5	C
58160130	Rice with beans and chicken	8000_Mixed	69	65	4	1	4	A
27341310	Chicken or turkey stew with potatoes and vegetables including carrots, broccoli, and/or dark-green leafy; gravy	8000_Mixed	69	64	5	1	4	A
58161422	Rice, brown, with corn, no added fat	8000_Mixed	69	64	5	1	3.5	B
27111400	Chili con carne, NS as to beans	8000_Mixed	69	63	6	2	4	B
27111406	Chili con carne with beans, home recipe	8000_Mixed	69	63	6	2	4	B
27336100	Venison or deer stew with potatoes and vegetables including carrots, broccoli, and/or dark-green leafy; tomato-based sauce	8000_Mixed	69	61	8	1	4	A
27440150	Chicken or turkey pate with vegetables, diet	8000_Mixed	69	59	10	1	4.5	A
27150510	Scallops with cheese sauce	8000_Mixed	69	58	11	2	3.5	C
58155910	Rice with squid, Puerto Rican style	8000_Mixed	69	57	12	1	3	C
27350060	Shrimp creole, with rice	8000_Mixed	69	56	13	2	3.5	B
14670000	Mozzarella cheese, tomato, and basil, with oil and vinegar dressing	8000_Mixed	68	70	-2	3	3.5	C
27446205	Chicken or turkey salad with nuts and/or fruits	8000_Mixed	68	70	-2	3	3.5	C
27510150	Cheeseburger, 1 miniature patty, on miniature bun, from school	8000_Mixed	68	69	-1	3	4	D
27311625	Beef, potatoes, and vegetables including carrots, broccoli, and/or dark-green leafy; tomato-based sauce	8000_Mixed	68	67	1	2	4	B
58163360	Flavored rice, brown and wild	8000_Mixed	68	66	2	1	3.5	C
72201230	Broccoli, cooked, NS as to form, with cheese sauce	8000_Mixed	68	66	2	3	4	B
72201232	Broccoli, cooked, from frozen, with cheese sauce	8000_Mixed	68	66	2	3	4	B
27415200	Beef and vegetables excluding carrots, broccoli, and dark-green leafy; no potatoes, soy-based sauce	8000_Mixed	68	65	3	1	4	A
27420160	Moo Shu pork, without Chinese pancake	8000_Mixed	68	65	3	2	3	D
58165430	Rice, brown, with vegetables and gravy, NS as to fat	8000_Mixed	68	65	3	1	3.5	C
27351020	Codfish salad, Puerto Rican style (Gazpacho de bacalao)	8000_Mixed	68	64	4	2	3.5	B
41601090	Bean soup, with macaroni, home recipe, canned, or ready-to-serve	8000_Mixed	68	64	4	1	4	B
41601170	Bean and rice soup	8000_Mixed	68	64	4	2	3.5	B
27250270	Clams Casino	8000_Mixed	68	62	6	3	4	B

28315140	Beef vegetable soup, home recipe, Mexican style	8000_Mixed	68	61	7	1	4	A
27336150	Venison or deer stew with potatoes and vegetables excluding carrots, broccoli, and dark-green leafy; tomato-based sauce	8000_Mixed	68	60	8	1	4	A
28345170	Duck soup	8000_Mixed	68	60	8	1	4	A
28350050	Fish chowder	8000_Mixed	68	58	10	2	3.5	B
58151420	Sushi, topped with salmon	8000_Mixed	68	57	11	1	3.5	B
75141030	Cabbage salad or coleslaw, made with creamy dressing	8000_Mixed	67	76	-9	3	4	B
41203030	Black bean salad	8000_Mixed	67	71	-4	3	4	B
74501010	Tomato aspic	8000_Mixed	67	70	-3	2	3.5	B
75440500	Vegetable combinations, including carrots, broccoli, and/or dark-green leafy; cooked, with cheese sauce	8000_Mixed	67	66	1	3	3.5	C
75651140	Vegetable soup with chicken broth, home recipe, Mexican style	8000_Mixed	67	66	1	1	3.5	A
27311645	Beef, potatoes, and vegetables including carrots, broccoli, and/or dark-green leafy; soy-based sauce	8000_Mixed	67	65	2	1	4	A
27440120	Chicken or turkey and vegetables excluding carrots, broccoli, and dark-green leafy; no potatoes, no sauce	8000_Mixed	67	65	2	2	3.5	C
58165450	Rice, brown, with vegetables and gravy, fat added	8000_Mixed	67	65	2	1	3.5	C
27345320	Chicken or turkey, rice, and vegetables excluding carrots, broccoli, and dark-green leafy; soy-based sauce	8000_Mixed	67	64	3	1	3.5	B
27416150	Pepper steak	8000_Mixed	67	64	3	1	4	B
58147110	Pasta with tomato-based sauce and beans or lentils	8000_Mixed	67	64	3	1	4	B
72201251	Broccoli, cooked, from fresh, with cream sauce	8000_Mixed	67	64	3	1	3.5	B
27211150	Beef goulash with potatoes	8000_Mixed	67	63	4	1	4	A
58160140	Rice with beans and pork	8000_Mixed	67	63	4	1	4	A
75417021	Peas, cooked, from fresh, with mushroom sauce	8000_Mixed	67	63	4	2	4	B
27341320	Chicken or turkey stew with potatoes and vegetables excluding carrots, broccoli, and dark-green leafy; gravy	8000_Mixed	67	61	6	1	4	A
75652010	Vegetable beef soup, home recipe	8000_Mixed	67	61	6	1	3.5	B
27111310	Beef stew, no potatoes, tomato-based sauce, with chili peppers, Mexican style	8000_Mixed	67	60	7	1	4	B
27121000	Pork with chili and tomatoes	8000_Mixed	67	60	7	1	4	A
58110200	Roll with meat and/or shrimp, vegetables and rice paper, not fried	8000_Mixed	67	59	8	1	4	B
75646010	Shav soup	8000_Mixed	67	56	11	1	3.5	B
58151150	Sushi, with seafood, no vegetables	8000_Mixed	67	55	12	1	3.5	B
27460100	Lau lau	8000_Mixed	67	52	15	1	4	A
75412060	Eggplant parmesan casserole, regular	8000_Mixed	66	66	0	2	4	B
27311650	Beef, potatoes, and vegetables excluding carrots, broccoli, and dark-green leafy; soy-based sauce	8000_Mixed	66	64	2	1	4	A
27320110	Pork, potatoes, and vegetables excluding carrots, broccoli, and dark-green leafy; tomato-based sauce	8000_Mixed	66	64	2	2	4	B
27420150	Kung Pao pork	8000_Mixed	66	64	2	1	4	A
27320320	Pork, rice, and vegetables including carrots, broccoli, and/or dark-green leafy; soy-based sauce	8000_Mixed	66	63	3	1	4	A
58165440	Rice, brown, with vegetables and gravy, no added fat	8000_Mixed	66	62	4	1	3.5	C

72201252	Broccoli, cooked, from frozen, with cream sauce	8000_Mixed	66	62	4	1	3.5	B
75414011	Mushrooms, from fresh, creamed	8000_Mixed	66	62	4	1	3.5	B
27250150	Tuna loaf	8000_Mixed	66	61	5	3	4	B
27121410	Chili con carne with beans, made with pork	8000_Mixed	66	60	6	2	4	B
28320300	Pork with vegetable excluding carrots, broccoli and/or dark-green leafy; soup, Asian Style	8000_Mixed	66	60	6	1	3.5	B
41601040	Lima bean soup, home recipe, canned or ready-to-serve	8000_Mixed	66	57	9	2	4	B
27150160	Shrimp with lobster sauce	8000_Mixed	66	54	12	2	2	D
27450090	Tuna salad with cheese	8000_Mixed	65	68	-3	4	3	D
75141100	Cabbage salad or coleslaw with apples and/or raisins, with dressing	8000_Mixed	65	68	-3	3	4	B
27311630	Beef, potatoes, and vegetables excluding carrots, broccoli, and dark-green leafy; tomato-based sauce	8000_Mixed	65	64	1	2	4	B
27442110	Chicken or turkey and vegetables including carrots, broccoli, and/or dark-green leafy; no potatoes, gravy	8000_Mixed	65	63	2	2	3.5	C
27450070	Shrimp salad	8000_Mixed	65	63	2	4	3	D
58146371	Pasta with tomato-based sauce, seafood, and added vegetables, restaurant	8000_Mixed	65	63	2	2	3.5	C
58165480	Rice, brown, with vegetables, soy-based sauce, fat added	8000_Mixed	65	63	2	1	3.5	C
75406011	Brussels sprouts, from fresh, creamed	8000_Mixed	65	63	2	1	3.5	B
27315510	Beef, rice, and vegetables including carrots, broccoli, and/or dark-green leafy; soy-based sauce	8000_Mixed	65	62	3	1	4	A
27415170	Kung Pao beef	8000_Mixed	65	62	3	1	4	A
27420170	Pork and onions with soy-based sauce	8000_Mixed	65	62	3	1	3.5	B
27320040	Pork, potatoes, and vegetables including carrots, broccoli, and/or dark-green leafy; no sauce	8000_Mixed	65	61	4	1	4	B
58132800	Spaghetti with clam sauce, NS as to red or white	8000_Mixed	65	61	4	2	3.5	C
58132820	Spaghetti with white clam sauce	8000_Mixed	65	61	4	2	3.5	C
58160132	Beans and rice, with meat	8000_Mixed	65	61	4	1	4	A
58160135	Rice with beans and beef	8000_Mixed	65	61	4	1	4	A
58406020	Turkey noodle soup, home recipe	8000_Mixed	65	61	4	2	3.5	A
75460700	Vegetable combinations, including carrots, broccoli, and/or dark-green leafy; cooked, with pasta	8000_Mixed	65	61	4	1	4	A
27415130	Szechuan beef	8000_Mixed	65	60	5	1	3.5	C
27415140	Hunan beef	8000_Mixed	65	60	5	1	3.5	C
28320160	Pork vegetable soup with potato, pasta, or rice, stew type, chunky style	8000_Mixed	65	60	5	1	3.5	B
75410530	Chiles rellenos, filled with meat and cheese	8000_Mixed	65	60	5	2	3.5	C
27246505	Meat loaf made with chicken or turkey, with tomato-based sauce	8000_Mixed	65	58	7	2	3.5	B
27363000	Gumbo with rice	8000_Mixed	65	58	7	1	3.5	B
28310220	Chili beef soup, chunky style	8000_Mixed	65	57	8	1	3.5	C
27250120	Shrimp and noodles, no sauce	8000_Mixed	65	55	10	1	4	A
75140990	Cabbage salad or coleslaw, from fast food / restaurant	8000_Mixed	64	65	-1	1	4	B
27341025	Chicken or turkey, potatoes, and vegetables including carrots, broccoli, and/or dark-green leafy; gravy	8000_Mixed	64	62	2	2	3.5	C

27550110	Crab cake sandwich	8000_Mixed	64	62	2	3	3.5	C
58146361	Pasta with tomato-based sauce and seafood, restaurant	8000_Mixed	64	62	2	2	3	C
58162140	Stuffed tomato, with rice, meatless	8000_Mixed	64	62	2	1	4	A
58164830	Rice, brown, with gravy, NS as to fat	8000_Mixed	64	62	2	2	3.5	C
58164850	Rice, brown, with gravy, fat added	8000_Mixed	64	62	2	2	3.5	C
58165460	Rice, brown, with vegetables, soy-based sauce, NS as to fat	8000_Mixed	64	62	2	1	3.5	C
75144100	Lettuce, wilted, with bacon dressing	8000_Mixed	64	62	2	2	4.5	A
27313150	Beef, noodles, and vegetables including carrots, broccoli, and/or dark-green leafy; soy-based sauce	8000_Mixed	64	61	3	1	4	A
27341020	Chicken or turkey, potatoes, and vegetables excluding carrots, broccoli, and dark-green leafy; no sauce	8000_Mixed	64	61	3	1	4	A
58101820	Mexican casserole made with ground beef, beans, tomato sauce, cheese, taco seasonings, and corn chips	8000_Mixed	64	61	3	2	4	C
27311110	Beef, potatoes, and vegetables including carrots, broccoli, and/or dark-green leafy; no sauce	8000_Mixed	64	60	4	1	4	B
75649100	Vegetable soup, cream of, made from dry mix, low sodium, prepared with water	8000_Mixed	64	60	4	2	4	A
27335500	Stewed rabbit, Puerto Rican style,	8000_Mixed	64	59	5	1	4	B
27450510	Tuna casserole with vegetables and mushroom sauce, no noodles	8000_Mixed	64	59	5	3	3.5	C
27111100	Beef goulash	8000_Mixed	64	57	7	1	4	A
27360100	Brunswick stew	8000_Mixed	64	57	7	1	4	A
27420520	Pork shish kabob with vegetables, excluding potatoes	8000_Mixed	64	57	7	1	4	A
27362000	Stewed tripe, with potatoes, Puerto Rican style	8000_Mixed	64	56	8	2	4	C
27463000	Stewed gizzards, Puerto Rican style	8000_Mixed	64	56	8	2	4	A
41601130	Bean soup, mixed beans, home recipe, canned or ready-to-serve	8000_Mixed	64	56	8	1	4	B
27142200	Turkey with gravy	8000_Mixed	64	55	9	1	4	A
28311020	Menudo soup, home recipe	8000_Mixed	64	55	9	1	3.5	B
58151410	Sushi, topped with eel	8000_Mixed	64	53	11	1	3.5	C
58409000	Noodle soup, with fish ball, shrimp, and dark green leafy vegetable	8000_Mixed	64	51	13	1	3.5	B
27261000	Breaded brains, Puerto Rican style	8000_Mixed	64	49	15	2	3	C
58146613	Pasta, whole grain, with tomato-based sauce and added vegetables, ready-to-heat	8000_Mixed	63	72	-9	4	3.5	C
75140510	Broccoli slaw salad	8000_Mixed	63	69	-6	3	4	B
27336200	Venison or deer, potatoes, and vegetables including carrots, broccoli, and/or dark-green leafy; gravy	8000_Mixed	63	61	2	2	3.5	B
28150230	Turbot with vegetable (diet frozen meal)	8000_Mixed	63	61	2	4	3.5	C
73102250	Carrots, cooked, NS as to form, with cheese sauce	8000_Mixed	63	61	2	2	3.5	C
73102251	Carrots, cooked, from fresh, with cheese sauce	8000_Mixed	63	61	2	2	3.5	C
73102252	Carrots, cooked, from frozen, with cheese sauce	8000_Mixed	63	61	2	2	3.5	C
27320330	Pork, rice, and vegetables excluding carrots, broccoli, and dark-green leafy; soy-based sauce	8000_Mixed	63	60	3	1	4	A
27545210	Turkey or chicken burger, with condiments, on wheat bun	8000_Mixed	63	60	3	3	4	B
27545220	Turkey or chicken burger, with condiments, on whole wheat bun	8000_Mixed	63	60	3	3	4	B

27250020	Clams, stuffed	8000_Mixed	63	59	4	3	3.5	C
27313160	Beef, noodles, and vegetables excluding carrots, broccoli, and dark-green leafy; soy-based sauce	8000_Mixed	63	59	4	1	4	A
27418210	Beef stew with vegetables excluding potatoes, Puerto Rican style	8000_Mixed	63	59	4	1	4	C
27420350	Pork and vegetables excluding carrots, broccoli, and dark-green leafy; no potatoes, no sauce	8000_Mixed	63	59	4	1	4	B
58164840	Rice, brown, with gravy, no added fat	8000_Mixed	63	59	4	1	3.5	C
72116230	Escarole, creamed	8000_Mixed	63	59	4	1	3.5	B
73111031	Peas and carrots, from fresh, creamed	8000_Mixed	63	58	5	1	3.5	C
75649150	Vegetable noodle soup, home recipe	8000_Mixed	63	58	5	1	3.5	C
77316510	Stuffed cabbage, with meat and rice, Syrian dish, Puerto Rican style	8000_Mixed	63	58	5	1	4.5	A
27111200	Beef burgundy	8000_Mixed	63	56	7	1	4	A
27150020	Crab, deviled	8000_Mixed	63	56	7	3	3	D
27420390	Pork chow mein or chop suey, no noodles	8000_Mixed	63	56	7	1	4	A
27410250	Beef shish kabob with vegetables, excluding potatoes	8000_Mixed	63	55	8	1	4	A
55702000	Cake or pancake made with rice flour and/or dried beans	8000_Mixed	63	54	9	1	4.5	A
28355310	Oyster stew	8000_Mixed	63	52	11	1	3	C
58136130	Lo mein, with shrimp	8000_Mixed	63	50	13	1	3.5	B
58146603	Pasta, whole grain, with tomato-based sauce, ready-to-heat	8000_Mixed	62	72	-10	4	3.5	C
28355140	Clam chowder, New England, reduced sodium, canned or ready-to-serve	8000_Mixed	62	64	-2	4	3.5	B
58146221	Pasta with tomato-based sauce, restaurant	8000_Mixed	62	63	-1	2	3	C
58162120	Stuffed pepper, with rice, meatless	8000_Mixed	62	63	-1	2	3.5	C
27341035	Chicken or turkey, potatoes, and vegetables including carrots, broccoli, and/or dark-green leafy; cream sauce, white sauce, or mushroom sauce	8000_Mixed	62	61	1	2	3.5	C
58100625	Enchilada with chicken and beans, green-chile or enchilada sauce	8000_Mixed	62	61	1	3	4	B
58146741	Pasta, whole grain, with cream sauce and seafood, restaurant	8000_Mixed	62	61	1	2	2.5	D
75403020	Green bean casserole	8000_Mixed	62	61	1	3	4	B
58104110	Nachos with beans, no cheese	8000_Mixed	62	60	2	3	4.5	A
58164860	Rice, brown, with soy-based sauce, NS as to fat	8000_Mixed	62	60	2	1	3	C
58164880	Rice, brown, with soy-based sauce, fat added	8000_Mixed	62	60	2	1	3	C
27315520	Beef, rice, and vegetables excluding carrots, broccoli, and dark-green leafy; soy-based sauce	8000_Mixed	62	59	3	1	4	A
27420120	Pork and watercress with soy-based sauce	8000_Mixed	62	59	3	1	3.5	B
27540205	Chicken fillet sandwich, grilled, on wheat bun	8000_Mixed	62	59	3	3	4	A
58165400	Rice, brown, with vegetables, cheese and/or cream based sauce, NS as to fat	8000_Mixed	62	59	3	2	3.5	C
58165420	Rice, brown, with vegetables, cheese and/or cream based sauce, fat added	8000_Mixed	62	59	3	2	3.5	C
58174100	Dosa (Indian), with filling	8000_Mixed	62	59	3	1	3.5	C
27343510	Chicken or turkey, noodles, and vegetables including carrots, broccoli, and/or dark-green leafy; tomato-based sauce	8000_Mixed	62	58	4	1	3.5	C
27545110	Turkey or chicken burger, on wheat bun	8000_Mixed	62	58	4	2	4	A

41601100	Portuguese bean soup, home recipe, canned or ready-to-serve	8000_Mixed	62	58	4	2	4.5	A
58101910	Taco or tostada salad with beef and cheese, corn chips	8000_Mixed	62	58	4	2	3.5	C
75403010	Beans, string, green, NS as to form, creamed or with cheese sauce	8000_Mixed	62	58	4	2	3.5	C
27320210	Pork, potatoes, and vegetables excluding carrots, broccoli, and dark-green leafy; no sauce	8000_Mixed	62	57	5	1	4	A
27336300	Venison or deer, noodles, and vegetables including carrots, broccoli, and/or dark-green leafy; tomato-based sauce	8000_Mixed	62	57	5	1	3.5	B
27410220	Beef and vegetables excluding carrots, broccoli, and dark-green leafy; no potatoes, no sauce	8000_Mixed	62	57	5	1	4	B
73102231	Carrots, cooked, from fresh, creamed	8000_Mixed	62	57	5	1	3.5	C
27211100	Beef stew with potatoes, tomato-based sauce	8000_Mixed	62	56	6	1	4	A
75417011	Peas, from fresh, creamed	8000_Mixed	62	56	6	1	3.5	B
27311410	Beef stew with potatoes and vegetables including carrots, broccoli, and/or dark-green leafy; gravy	8000_Mixed	62	55	7	1	4	A
41601140	Bean soup, home recipe	8000_Mixed	62	55	7	1	4	A
58115150	Tamal in a leaf, Puerto Rican style	8000_Mixed	62	55	7	1	4	B
58137240	Pad Thai with seafood	8000_Mixed	62	55	7	1	3.5	C
27111405	Chili con carne with beans, from restaurant	8000_Mixed	62	54	8	1	3.5	C
27430610	Lamb shish kabob with vegetables, excluding potatoes	8000_Mixed	62	54	8	1	4	A
41602020	Garbanzo bean or chickpea soup, home recipe, canned or ready-to-serve	8000_Mixed	62	54	8	1	4	B
32105210	Chicken egg foo yung	8000_Mixed	62	52	10	1	3.5	B
58146653	Pasta, whole grain, with tomato-based sauce, poultry, and added vegetables, ready-to-heat	8000_Mixed	61	69	-8	4	3.5	B
75141200	Cabbage salad or coleslaw with pineapple, with dressing	8000_Mixed	61	66	-5	3	4	B
75416500	Pea salad	8000_Mixed	61	66	-5	3	3.5	C
42301125	Peanut butter sandwich, with reduced fat peanut butter, on whole wheat bread	8000_Mixed	61	65	-4	3	3.5	C
74506000	Tomato and cucumber salad made with tomato, cucumber, oil, and vinegar	8000_Mixed	61	64	-3	2	3	C
58100720	Enchilada with beans, meatless, red-chile or enchilada sauce	8000_Mixed	61	61	0	3	4	B
27250450	Shrimp toast, fried	8000_Mixed	61	59	2	2	3	D
27341030	Chicken or turkey, potatoes, and vegetables excluding carrots, broccoli, and dark-green leafy; gravy	8000_Mixed	61	59	2	2	3.5	C
58100620	Enchilada with chicken and beans, red-chile or enchilada sauce	8000_Mixed	61	59	2	3	4	B
58132450	Spaghetti with tomato sauce, meatless, made with spinach noodles	8000_Mixed	61	59	2	2	3.5	C
77316010	Stuffed cabbage, with meat, Puerto Rican style	8000_Mixed	61	59	2	2	4	C
27311600	Beef, potatoes, and vegetables including carrots, broccoli, and/or dark-green leafy; gravy	8000_Mixed	61	58	3	2	3.5	B
27320140	Pork, potatoes, and vegetables including carrots, broccoli, and/or dark-green leafy; gravy	8000_Mixed	61	58	3	2	3.5	B
27336250	Venison or deer, potatoes, and vegetables excluding carrots, broccoli, and dark-green leafy; gravy	8000_Mixed	61	58	3	2	3.5	B
27550120	Salmon cake sandwich	8000_Mixed	61	58	3	3	3.5	C
75403011	Beans, string, green, from fresh, creamed or with cheese sauce	8000_Mixed	61	58	3	2	3.5	C

75403012	Beans, string, green, from frozen, creamed or with cheese sauce	8000_Mixed	61	58	3	2	3.5	C
75410500	Chiles rellenos, cheese-filled	8000_Mixed	61	58	3	2	3	C
27550510	Sardine sandwich	8000_Mixed	61	57	4	4	4	A
27311120	Beef, potatoes, and vegetables, excluding carrots, broccoli, and dark-green leafy; no sauce	8000_Mixed	61	56	5	1	4	A
73102230	Carrots, cooked, NS as to form, creamed	8000_Mixed	61	56	5	1	3.5	C
73102232	Carrots, cooked, from frozen, creamed	8000_Mixed	61	56	5	1	3.5	C
75409010	Cauliflower, NS as to form, creamed	8000_Mixed	61	56	5	1	3.5	B
75417022	Peas, cooked, from frozen, with mushroom sauce	8000_Mixed	61	56	5	2	4	B
27150170	Sweet and sour shrimp	8000_Mixed	61	55	6	2	3	C
27212150	Beef goulash with noodles	8000_Mixed	61	55	6	1	4	A
27246500	Meat loaf made with chicken or turkey	8000_Mixed	61	55	6	2	4	B
73111030	Peas and carrots, NS as to form, creamed	8000_Mixed	61	55	6	1	3.5	C
73111032	Peas and carrots, from frozen, creamed	8000_Mixed	61	55	6	1	3.5	C
75601100	Borscht	8000_Mixed	61	55	6	2	3.5	A
77563010	Puerto Rican stew	8000_Mixed	61	55	6	1	4	A
27211200	Beef stew with potatoes, gravy	8000_Mixed	61	54	7	1	4	A
27311320	Beef stew with potatoes and vegetables excluding carrots, broccoli, and dark-green leafy; tomato-based sauce	8000_Mixed	61	54	7	1	4	A
75652040	Vegetable beef soup with noodles or pasta, home recipe	8000_Mixed	61	54	7	1	3.5	B
77230510	Cassava fritter stuffed with crab meat, Puerto Rican style	8000_Mixed	61	54	7	1	3.5	C
27330210	Lamb or mutton stew with potatoes and vegetables including carrots, broccoli, and/or dark-green leafy; tomato-based sauce	8000_Mixed	61	53	8	1	4	A
27330220	Lamb or mutton stew with potatoes and vegetables excluding carrots, broccoli, and dark-green leafy; tomato-based sauce	8000_Mixed	61	53	8	1	4	A
58146372	Pasta with tomato-based sauce, seafood, and added vegetables, home recipe	8000_Mixed	61	52	9	2	4	B
73305020	Squash, winter, souffle	8000_Mixed	61	52	9	1	4	B
27416400	Stir fried beef and vegetables in soy sauce	8000_Mixed	61	50	11	1	4	A
42301020	Peanut butter sandwich, with regular peanut butter, on wheat bread	8000_Mixed	60	65	-5	3	2.5	D
42301025	Peanut butter sandwich, with regular peanut butter, on whole wheat bread	8000_Mixed	60	65	-5	3	2.5	D
42301120	Peanut butter sandwich, with reduced fat peanut butter, on wheat bread	8000_Mixed	60	65	-5	3	3.5	C
72308500	Dark-green leafy vegetable soup, meatless, Asian style	8000_Mixed	60	62	-2	3	3.5	B
58100525	Enchilada with meat and beans, green-chile or enchilada sauce	8000_Mixed	60	59	1	3	4	B
27311610	Beef, potatoes, and vegetables including carrots, broccoli, and/or dark-green leafy; cream sauce, white sauce, or mushroom sauce	8000_Mixed	60	58	2	2	3.5	B
77272010	Puerto Rican pasteles	8000_Mixed	60	58	2	2	3.5	C
27243600	Chicken or turkey and rice with soy-based sauce	8000_Mixed	60	57	3	1	3.5	C
27416450	Beef and vegetables including carrots, broccoli, and/or dark-green leafy; no potatoes, gravy	8000_Mixed	60	57	3	2	3.5	B
58147100	Pasta with pesto sauce	8000_Mixed	60	57	3	2	3	C
58421020	Sopa de Fideo Aguada, Mexican style noodle soup, home recipe	8000_Mixed	60	57	3	1	3.5	A

27120150	Pork or ham with soy-based sauce	8000_Mixed	60	56	4	1	3.5	B
27241010	Chicken or turkey and potatoes with gravy	8000_Mixed	60	56	4	1	3.5	C
27242500	Chicken or turkey and noodles with soy-based sauce	8000_Mixed	60	56	4	1	3.5	B
27336310	Venison or deer, noodles, and vegetables excluding carrots, broccoli, and dark-green leafy; tomato-based sauce	8000_Mixed	60	56	4	1	3.5	B
27343520	Chicken or turkey, noodles, and vegetables excluding carrots, broccoli, and dark-green leafy; tomato-based sauce	8000_Mixed	60	56	4	1	3.5	C
58146732	Pasta, whole grain, with cream sauce, poultry, and added vegetables, home recipe	8000_Mixed	60	56	4	2	3.5	C
58165410	Rice, brown, with vegetables, cheese and/or cream based sauce, no added fat	8000_Mixed	60	56	4	2	3.5	C
75409012	Cauliflower, from frozen, creamed	8000_Mixed	60	56	4	1	3.5	B
27146300	Chicken or turkey parmigiana	8000_Mixed	60	55	5	2	3.5	C
27232000	Lamb or mutton and potatoes with tomato-based sauce	8000_Mixed	60	55	5	1	4	B
27250810	Fish and rice with tomato-based sauce	8000_Mixed	60	55	5	1	3.5	B
75439010	Vegetable stew without meat	8000_Mixed	60	54	6	2	3.5	B
28310170	Beef broth, without tomato, home recipe	8000_Mixed	60	53	7	1	3.5	B
28340550	Sweet and sour soup	8000_Mixed	60	53	7	2	3.5	C
58403040	Chicken or turkey noodle soup, home recipe	8000_Mixed	60	53	7	1	3.5	B
27448030	Chicken or turkey fricassee, no sauce, no potatoes, Puerto Rican style	8000_Mixed	60	52	8	1	3.5	D
28310320	Beef noodle soup, Puerto Rican style	8000_Mixed	60	51	9	1	3.5	B
75142550	Cucumber salad, made with Italian dressing	8000_Mixed	59	62	-3	2	3.5	C
75651150	Vegetable noodle soup, reduced sodium, canned, prepared with water or ready-to-serve	8000_Mixed	59	62	-3	4	4	A
27446332	Chicken or turkey, breaded, fried, garden salad with cheese, chicken and/or turkey, cheese, lettuce and/or greens, tomato and/or carrots, other vegetables, no dressing	8000_Mixed	59	61	-2	3	4	A
58100725	Enchilada with beans, green-chile or enchilada sauce	8000_Mixed	59	58	1	3	4	A
27341040	Chicken or turkey, potatoes, and vegetables excluding carrots, broccoli, and dark-green leafy; cream sauce, white sauce, or mushroom sauce	8000_Mixed	59	57	2	2	3.5	C
27414100	Beef with vegetables including carrots, broccoli, and/or dark-green leafy; no potatoes, mushroom sauce	8000_Mixed	59	57	2	2	3.5	B
27348100	Chicken fricassee, Puerto Rican style	8000_Mixed	59	56	3	2	3.5	D
58100520	Enchilada with meat and beans, red-chile or enchilada sauce	8000_Mixed	59	56	3	3	4	B
58117510	Hayacas, Puerto Rican style	8000_Mixed	59	56	3	3	4	B
58124250	Spanakopitta	8000_Mixed	59	56	3	3	3	C
58164800	Rice, brown, with cheese and/or cream based sauce, NS as to fat	8000_Mixed	59	56	3	2	3.5	C
58164820	Rice, brown, with cheese and/or cream based sauce, fat added	8000_Mixed	59	56	3	2	3.5	C
58100630	Enchilada with chicken, red-chile or enchilada sauce	8000_Mixed	59	55	4	3	4	B
58101830	Mexican casserole made with ground beef, tomato sauce, cheese, taco seasonings, and corn chips	8000_Mixed	59	55	4	3	3	D
75407010	Cabbage, creamed	8000_Mixed	59	55	4	1	3.5	B

27320070	Ham or pork, noodles, and vegetables including carrots, broccoli, and/or dark-green leafy; tomato-based sauce	8000_Mixed	59	54	5	1	3.5	C
58165470	Rice, brown, with vegetables, soy-based sauce, no added fat	8000_Mixed	59	54	5	1	3.5	C
71931010	Cassava with creole sauce, Puerto Rican style	8000_Mixed	59	54	5	1	4	A
27250300	Mackerel cake or patty	8000_Mixed	59	52	7	3	3.5	D
27311420	Beef stew with potatoes and vegetables excluding carrots, broccoli, and dark-green leafy; gravy	8000_Mixed	59	52	7	1	4	A
58146362	Pasta with tomato-based sauce and seafood, home recipe	8000_Mixed	59	52	7	2	3.5	B
28340680	Chicken or turkey and corn hominy soup, home recipe, Mexican style	8000_Mixed	59	51	8	1	3.5	A
58117410	Codfish fritter, Puerto Rican style	8000_Mixed	59	51	8	1	4	A
72125240	Spinach souffle	8000_Mixed	59	51	8	1	3.5	C
27250122	Shrimp and noodles with gravy	8000_Mixed	59	50	9	2	3.5	B
27430510	Veal goulash with vegetables including carrots, broccoli, and/or dark-green leafy; no potatoes, tomato-based sauce	8000_Mixed	59	50	9	1	4	A
27462000	Stewed chitterlings, Puerto Rican style	8000_Mixed	59	50	9	1	3.5	C
28310420	Beef and rice soup, Puerto Rican style	8000_Mixed	59	50	9	1	3.5	B
28340800	Chicken or turkey soup with vegetables and fruit, Asian Style	8000_Mixed	59	50	9	2	3	C
27350410	Tuna noodle casserole with vegetables and mushroom sauce	8000_Mixed	59	49	10	2	3.5	B
32105220	Pork egg foo yung	8000_Mixed	59	49	10	1	3.5	B
32105240	Beef egg foo yung	8000_Mixed	59	49	10	1	3.5	B
58151440	Sushi, topped with tuna	8000_Mixed	59	49	10	1	4	A
27250260	Lobster with bread stuffing, baked	8000_Mixed	59	48	11	2	2	D
58151200	Sushi roll, salmon	8000_Mixed	59	47	12	1	3.5	C
75416600	Pea salad with cheese	8000_Mixed	58	64	-6	3	3.5	C
42302020	Peanut butter and jelly sandwich, with regular peanut butter, regular jelly, on wheat bread	8000_Mixed	58	62	-4	3	2.5	D
42302025	Peanut butter and jelly sandwich, with regular peanut butter, regular jelly, on whole wheat bread	8000_Mixed	58	62	-4	3	2.5	D
75650990	Minestrone soup, reduced sodium, canned or ready-to-serve	8000_Mixed	58	62	-4	4	4	A
58146301	Pasta with tomato-based sauce, and added vegetables, restaurant	8000_Mixed	58	59	-1	2	3.5	C
58146351	Pasta with tomato-based sauce, poultry, and added vegetables, restaurant	8000_Mixed	58	57	1	2	3.5	C
58162090	Stuffed pepper, with meat	8000_Mixed	58	57	1	2	3.5	C
27540206	Chicken fillet sandwich, grilled, on wheat bun, with cheese	8000_Mixed	58	56	2	3	4	C
72308000	Dark-green leafy vegetable soup with meat, Asian style	8000_Mixed	58	56	2	2	4	A
27246300	Chicken or turkey cake, patty, or croquette	8000_Mixed	58	55	3	2	3.5	D
27320150	Pork, potatoes, and vegetables excluding carrots, broccoli, and dark-green leafy; gravy	8000_Mixed	58	55	3	2	3.5	B
27442120	Chicken or turkey and vegetables excluding carrots, broccoli, and dark-green leafy; no potatoes, gravy	8000_Mixed	58	55	3	2	3.5	C
27446230	Chicken or turkey salad, made with mayonnaise-type salad dressing	8000_Mixed	58	55	3	2	3.5	C
41311040	Lentil curry with rice	8000_Mixed	58	55	3	1	3.5	C
27115000	Beef with soy-based sauce	8000_Mixed	58	54	4	1	3.5	B

27150325	Fish curry with rice	8000_Mixed	58	54	4	1	3.5	C
58162110	Stuffed pepper, with rice and meat	8000_Mixed	58	54	4	2	3.5	C
27242400	Chicken or turkey and noodles with tomato-based sauce	8000_Mixed	58	53	5	1	3.5	C
27313210	Beef, noodles, and vegetables including carrots, broccoli, and/or dark-green leafy; tomato-based sauce	8000_Mixed	58	53	5	1	3.5	C
27341045	Chicken or turkey, potatoes, and vegetables including carrots, broccoli, and/or dark-green leafy; cheese sauce	8000_Mixed	58	53	5	2	3.5	B
58162130	Stuffed tomato, with rice and meat	8000_Mixed	58	53	5	1	4	B
27111410	Chili con carne with beans	8000_Mixed	58	52	6	3	4	C
27211000	Beef and potatoes, no sauce	8000_Mixed	58	52	6	1	4	A
27311510	Shepherd's pie with beef	8000_Mixed	58	52	6	2	4	A
27418110	Seasoned shredded soup meat	8000_Mixed	58	52	6	2	4	A
77205610	Ripe plantain meat pie, Puerto Rican style	8000_Mixed	58	52	6	2	3.5	D
75417010	Peas, NS as to form, creamed	8000_Mixed	58	51	7	1	3.5	C
75417012	Peas, from frozen, creamed	8000_Mixed	58	51	7	1	3.5	C
75652050	Vegetable beef soup with rice, home recipe	8000_Mixed	58	51	7	1	3.5	B
27430500	Veal goulash with vegetables excluding carrots, broccoli, and dark-green leafy; no potatoes, tomato-based sauce	8000_Mixed	58	50	8	1	4	A
27460710	Livers, chicken, chopped, with eggs and onion	8000_Mixed	58	50	8	2	1.5	E
28320150	Pork, vegetable soup with potatoes, stew type	8000_Mixed	58	50	8	2	4	A
27250124	Shrimp and noodles with mushroom sauce	8000_Mixed	58	49	9	2	3.5	B
27350080	Tuna noodle casserole with vegetables, cream or white sauce	8000_Mixed	58	49	9	2	3.5	B
27150050	Fish timbale or mousse	8000_Mixed	58	45	13	2	3	C
27540139	Chicken fillet sandwich, from school cafeteria	8000_Mixed	57	64	-7	4	4	B
58146315	Pasta with sauce and meat, from school lunch	8000_Mixed	57	64	-7	4	4	A
75141000	Cabbage salad or coleslaw, made with coleslaw dressing	8000_Mixed	57	64	-7	3	4	B
41811950	Swiss steak, with gravy, meatless	8000_Mixed	57	60	-3	4	3.5	C
27446200	Chicken or turkey salad, made with mayonnaise	8000_Mixed	57	59	-2	3	3.5	C
42302165	Peanut butter and jelly sandwich, with reduced fat peanut butter, reduced sugar jelly, on whole wheat bread	8000_Mixed	57	59	-2	3	3.5	D
27416250	Beef salad	8000_Mixed	57	58	-1	3	3.5	C
27446220	Chicken or turkey salad with egg	8000_Mixed	57	58	-1	3	3.5	C
58146341	Pasta with tomato-based sauce and poultry, restaurant	8000_Mixed	57	57	0	2	3	C
58148130	Macaroni or pasta salad with tuna	8000_Mixed	57	57	0	2	3.5	C
58148140	Macaroni or pasta salad with crab meat	8000_Mixed	57	57	0	2	3.5	C
27311620	Beef, potatoes, and vegetables excluding carrots, broccoli, and dark-green leafy; cream sauce, white sauce, or mushroom sauce	8000_Mixed	57	55	2	2	3.5	B
27446235	Chicken or turkey salad, made with light mayonnaise-type salad dressing	8000_Mixed	57	55	2	2	3.5	C
27540260	Chicken fillet, broiled, sandwich, on oat bran bun, with lettuce, tomato, spread	8000_Mixed	57	55	2	3	4	B
58148160	Macaroni or pasta salad with tuna and egg	8000_Mixed	57	55	2	2	3.5	C
75403022	Beans, string, green, cooked, from frozen, with mushroom sauce	8000_Mixed	57	55	2	3	3.5	B

75440610	Vegetable curry with rice	8000_Mixed	57	55	2	1	3.5	C
27311605	Beef, potatoes, and vegetables excluding carrots, broccoli, and dark-green leafy; gravy	8000_Mixed	57	54	3	2	3.5	B
27345510	Chicken or turkey, rice, and vegetables including carrots, broccoli, and/or dark-green leafy; tomato-based sauce	8000_Mixed	57	54	3	1	3.5	C
27446225	Chicken or turkey salad, made with light mayonnaise	8000_Mixed	57	54	3	2	3.5	C
28150370	Flounder in cream sauce with potatoes, carrots (diet frozen meal)	8000_Mixed	57	54	3	4	3.5	C
58100710	Enchilada with beans, meatless	8000_Mixed	57	54	3	3	4.5	A
58132110	Spaghetti with tomato sauce, meatless	8000_Mixed	57	54	3	2	3.5	C
58132340	Spaghetti with tomato sauce and vegetables	8000_Mixed	57	54	3	2	3.5	B
58132910	Spaghetti with tomato sauce and poultry	8000_Mixed	57	54	3	2	3.5	B
58146100	Pasta with tomato sauce, meatless	8000_Mixed	57	54	3	2	3.5	C
58146692	Pasta, whole grain, with cream sauce, and added vegetables, home recipe	8000_Mixed	57	54	3	2	3.5	C
27213500	Beef and rice with soy-based sauce	8000_Mixed	57	53	4	1	3.5	B
27446400	Chicken or turkey and vegetables including carrots, broccoli, and/or dark-green leafy; no potatoes, cheese sauce	8000_Mixed	57	53	4	2	3.5	C
58146160	Pasta with vegetables, no sauce or dressing	8000_Mixed	57	53	4	1	4	B
58148150	Macaroni or pasta salad with shrimp	8000_Mixed	57	53	4	2	3.5	C
58164810	Rice, brown, with cheese and/or cream based sauce, no added fat	8000_Mixed	57	53	4	2	3.5	C
27218210	Beef stew with potatoes, Puerto Rican style	8000_Mixed	57	52	5	1	3.5	D
27446245	Chicken or turkey salad, made with light creamy dressing	8000_Mixed	57	52	5	2	3.5	C
75410010	Celery, creamed	8000_Mixed	57	52	5	1	3.5	C
27343010	Chicken or turkey, noodles, and vegetables including carrots, broccoli, and/or dark-green leafy; no sauce	8000_Mixed	57	51	6	1	3.5	B
75402020	Beans, lima, immature, cooked, NS as to form, with mushroom sauce	8000_Mixed	57	51	6	2	4	B
75450510	Vegetable combination, excluding carrots, broccoli, and dark-green leafy; cooked, with cream sauce	8000_Mixed	57	51	6	1	3.5	C
75460810	Vegetable combinations, excluding carrots, broccoli, and dark-green leafy; cooked, with butter sauce and pasta	8000_Mixed	57	51	6	1	4	A
27121010	Stewed pork, Puerto Rican style	8000_Mixed	57	50	7	1	3.5	D
27411120	Swiss steak	8000_Mixed	57	50	7	1	3.5	B
58402100	Beef noodle soup, home recipe	8000_Mixed	57	50	7	1	3.5	B
27330110	Lamb or mutton stew with potatoes and vegetables excluding carrots, broccoli, and dark-green leafy; gravy	8000_Mixed	57	49	8	1	4	A
27335100	Rabbit stew with potatoes and vegetables	8000_Mixed	57	49	8	1	3.5	B
27211110	Beef stew with potatoes, tomato-based sauce, Mexican style	8000_Mixed	57	48	9	1	4	B
27422010	Pork chop stewed with vegetables, Puerto Rican style	8000_Mixed	57	48	9	1	4	B
27430410	Lamb or mutton stew with vegetables excluding carrots, broccoli, and dark-green leafy; no potatoes, gravy	8000_Mixed	57	47	10	1	4	A
58134210	Stuffed shells, with chicken, with tomato sauce	8000_Mixed	57	47	10	1	4	A
27446362	Chicken or turkey, breaded, fried, caesar garden salad, chicken and/or turkey, lettuce, tomatoes, cheese, no dressing	8000_Mixed	56	59	-3	4	4	A

42302110	Peanut butter and jelly sandwich, with regular peanut butter, reduced sugar jelly, on wheat bread	8000_Mixed	56	58	-2	3	2.5	D
42302115	Peanut butter and jelly sandwich, with regular peanut butter, reduced sugar jelly, on whole wheat bread	8000_Mixed	56	58	-2	3	2.5	D
42302160	Peanut butter and jelly sandwich, with reduced fat peanut butter, reduced sugar jelly, on wheat bread	8000_Mixed	56	58	-2	3	3.5	D
27446240	Chicken or turkey salad, made with creamy dressing	8000_Mixed	56	57	-1	3	3	C
41601030	Black bean soup, home recipe, canned or ready-to-serve	8000_Mixed	56	57	-1	4	3.5	C
14620310	Topping from vegetable pizza	8000_Mixed	56	56	0	3	3	D
58146150	Pasta with tomato-based sauce and cheese	8000_Mixed	56	54	2	2	3.5	C
73102253	Carrots, cooked, from canned, with cheese sauce	8000_Mixed	56	54	2	3	3	C
74602300	Tomato soup, canned, reduced sodium, prepared with milk	8000_Mixed	56	54	2	3	3.5	A
75141300	Cabbage, Chinese, salad, with dressing	8000_Mixed	56	54	2	2	3	C
75403013	Beans, string, green, from canned, creamed or with cheese sauce	8000_Mixed	56	54	2	2	3.5	C
58100635	Enchilada with chicken, green-chile or enchilada sauce	8000_Mixed	56	53	3	2	4	B
27212500	Beef and noodles with soy-based sauce	8000_Mixed	56	52	4	1	3.5	B
27220510	Ham or pork and potatoes with gravy	8000_Mixed	56	52	4	2	3.5	B
75409011	Cauliflower, from fresh, creamed	8000_Mixed	56	52	4	1	3.5	C
27313220	Beef, noodles, and vegetables excluding carrots, broccoli, and dark-green leafy; tomato-based sauce	8000_Mixed	56	51	5	1	3.5	C
27330010	Shepherd's pie with lamb	8000_Mixed	56	51	5	2	4	A
58100610	Enchilada with chicken and beans, tomato-based sauce	8000_Mixed	56	51	5	3	4	A
58132460	Spaghetti with tomato sauce and meatballs made with spinach noodles, or spaghetti with meat sauce made with spinach noodles, or spaghetti with meat sauce and meatballs made with spinach noodles	8000_Mixed	56	51	5	2	3.5	C
73111033	Peas and carrots, from canned, creamed	8000_Mixed	56	51	5	2	3.5	C
75415011	Onions, from fresh, creamed	8000_Mixed	56	51	5	1	3.5	C
27313110	Beef chow mein or chop suey with noodles	8000_Mixed	56	50	6	1	4	A
58164870	Rice, brown, with soy-based sauce, no added fat	8000_Mixed	56	50	6	1	3	C
75402012	Beans, lima, immature, from frozen, creamed or with cheese sauce	8000_Mixed	56	50	6	2	4	A
27141050	Stewed chicken with tomato-based sauce, Mexican style	8000_Mixed	56	49	7	2	3.5	B
27331150	Veal fricassee, Puerto Rican style	8000_Mixed	56	49	7	2	3.5	C
27446255	Chicken or turkey salad, made with light Italian dressing	8000_Mixed	56	49	7	2	3.5	C
27448020	Chicken or turkey fricassee, with sauce, no potatoes, potatoes reported separately, Puerto Rican style	8000_Mixed	56	49	7	2	3.5	D
28310230	Meatball soup, home recipe, Mexican style	8000_Mixed	56	49	7	2	4	B
28340590	Chicken or turkey corn soup with noodles, home recipe	8000_Mixed	56	49	7	2	3.5	C
58115210	Taco with crab meat, Puerto Rican style	8000_Mixed	56	48	8	2	3	D
27221150	Pork stew, with potatoes, tomato-based sauce, Mexican style	8000_Mixed	56	47	9	1	3.5	B
27330030	Lamb or mutton stew with potatoes and vegetables including carrots, broccoli, and/or dark-green leafy; gravy	8000_Mixed	56	47	9	1	4	A
27416100	Beef and vegetables, Hawaiian style	8000_Mixed	56	47	9	1	3.5	C

27430400	Lamb or mutton stew with vegetables including carrots, broccoli, and/or dark-green leafy; no potatoes, gravy	8000_Mixed	56	47	9	1	4	B
58155310	Paella with meat, Valenciana style	8000_Mixed	56	47	9	1	2	D
27144000	Chicken or turkey with mushroom sauce	8000_Mixed	56	46	10	1	4	B
28340750	Hot and sour soup	8000_Mixed	56	46	10	1	3	C
58151190	Sushi roll, eel	8000_Mixed	56	46	10	1	3.5	C
27250550	Seafood souffle	8000_Mixed	56	41	15	2	3	D
58151430	Sushi, topped with shrimp	8000_Mixed	56	39	17	1	3.5	B
58302080	Noodles with vegetables in tomato-based sauce, diet frozen meal	8000_Mixed	55	60	-5	4	4	A
75609050	Pea soup, canned, low sodium, prepared with water	8000_Mixed	55	59	-4	4	4	A
41812850	Vegetarian stroganoff	8000_Mixed	55	54	1	3	3	C
72302000	Broccoli soup, prepared with milk, home recipe, canned or ready-to-serve	8000_Mixed	55	54	1	3	3	C
58137220	Pad Thai, meatless	8000_Mixed	55	53	2	2	3.5	C
72302100	Broccoli cheese soup, prepared with milk, home recipe, canned, or ready-to-serve	8000_Mixed	55	53	2	3	3	C
75418020	Squash, summer, casserole with tomato and cheese	8000_Mixed	55	53	2	3	4	A
27446250	Chicken or turkey salad, made with Italian dressing	8000_Mixed	55	52	3	2	3.5	C
58101710	Taco or tostada with beans, meatless, with lettuce, tomato and salsa	8000_Mixed	55	52	3	3	4	A
27320340	Pork, rice, and vegetables including carrots, broccoli, and/or dark-green leafy; tomato-based sauce	8000_Mixed	55	51	4	1	3.5	C
27345520	Chicken or turkey, rice, and vegetables excluding carrots, broccoli, and dark-green leafy; tomato-based sauce	8000_Mixed	55	51	4	1	3.5	C
28153010	Shrimp and clams in tomato-based sauce, with noodles, frozen meal	8000_Mixed	55	51	4	4	3.5	B
58100500	Enchilada, no sauce	8000_Mixed	55	51	4	2	4	B
58100530	Enchilada with meat, red-chile or enchilada sauce	8000_Mixed	55	51	4	2	4	B
58146222	Pasta with tomato-based sauce, home recipe	8000_Mixed	55	51	4	2	3.5	B
58146722	Pasta, whole grain, with cream sauce and poultry, home recipe	8000_Mixed	55	51	4	2	3.5	C
73102233	Carrots, cooked, from canned, creamed	8000_Mixed	55	51	4	1	3	C
75418051	Squash, summer, from fresh, creamed	8000_Mixed	55	51	4	1	3.5	B
27341050	Chicken or turkey, potatoes, and vegetables excluding carrots, broccoli, and dark-green leafy; cheese sauce	8000_Mixed	55	50	5	2	3.5	B
27416500	Beef and vegetables excluding carrots, broccoli, and dark-green leafy; no potatoes, gravy	8000_Mixed	55	50	5	2	3.5	B
28350210	Clam chowder, NS as to Manhattan or New England style	8000_Mixed	55	50	5	3	3.5	C
28355120	Clam chowder, New England, prepared with milk	8000_Mixed	55	50	5	3	3.5	C
58104150	Nachos with chicken and cheese	8000_Mixed	55	50	5	3	4	B
75418050	Squash, summer, NS as to form, creamed	8000_Mixed	55	50	5	1	3.5	B
27133010	Stewed goat, Puerto Rican style	8000_Mixed	55	49	6	2	2	D
27135110	Veal parmigiana	8000_Mixed	55	49	6	2	3.5	D
27343910	Chicken or turkey chow mein or chop suey with noodles	8000_Mixed	55	49	6	1	4	B
27360080	Chow mein or chop suey, NS as to type of meat, with noodles	8000_Mixed	55	49	6	1	4	B

27430580	Veal with vegetables including carrots, broccoli, and/or dark-green leafy; no potatoes, cream or white sauce	8000_Mixed	55	49	6	2	3.5	C
41610100	White bean soup, Puerto Rican style (Sopon de habichuelas blancas)	8000_Mixed	55	49	6	1	3	C
58146352	Pasta with tomato-based sauce, poultry, and added vegetables, home recipe	8000_Mixed	55	49	6	1	4	A
75415010	Onions, NS as to form, creamed	8000_Mixed	55	49	6	1	3.5	C
75418053	Squash, summer, from canned, creamed	8000_Mixed	55	49	6	1	3.5	B
27211300	Beef, roast, hash	8000_Mixed	55	48	7	2	3.5	C
27343020	Chicken or turkey, noodles, and vegetables excluding carrots, broccoli, and dark-green leafy; no sauce	8000_Mixed	55	48	7	1	3.5	B
27332100	Veal stew with potatoes and vegetables including carrots, broccoli, and/or dark-green leafy; tomato-based sauce	8000_Mixed	55	47	8	1	4	A
27111300	Beef stew, no potatoes, tomato-based sauce, Mexican style	8000_Mixed	55	46	9	1	4	B
27120130	Pork stew, no potatoes, tomato-based sauce, Mexican style	8000_Mixed	55	46	9	1	4	B
27315250	Stuffed cabbage rolls with beef and rice	8000_Mixed	55	46	9	1	4	B
27332110	Veal stew with potatoes and vegetables excluding carrots, broccoli, and/or dark-green leafy; tomato-based sauce	8000_Mixed	55	46	9	1	4	A
28310150	Oxtail soup	8000_Mixed	55	46	9	1	3.5	B
58117310	Kibby, Puerto Rican style	8000_Mixed	55	46	9	1	3.5	C
27250630	Tuna noodle casserole with mushroom sauce	8000_Mixed	55	45	10	2	3.5	C
27142000	Chicken with gravy	8000_Mixed	55	44	11	1	4	B
58146623	Pasta, whole grain, with tomato-based sauce and meat, ready-to-heat	8000_Mixed	54	60	-6	4	3.5	C
58146331	Pasta with tomato-based sauce, meat, and added vegetables, restaurant	8000_Mixed	54	53	1	2	3.5	C
75403023	Beans, string, green, cooked, from canned, with mushroom sauce	8000_Mixed	54	53	1	3	3.5	C
58146321	Pasta with tomato-based sauce and meat, restaurant	8000_Mixed	54	52	2	2	3	C
77250110	Stuffed tannier fritters, Puerto Rican style	8000_Mixed	54	52	2	2	2.5	E
27414200	Beef with vegetables excluding carrots, broccoli, and dark-green leafy; no potatoes, mushroom sauce	8000_Mixed	54	51	3	2	3.5	B
58101940	Taco or tostada salad, meatless	8000_Mixed	54	51	3	3	4	B
27315210	Beef, rice, and vegetables including carrots, broccoli, and/or dark-green leafy; tomato-based sauce	8000_Mixed	54	50	4	1	3.5	C
27510190	Cheeseburger, from school cafeteria	8000_Mixed	54	50	4	3	4	D
58100535	Enchilada with meat, green-chile or enchilada sauce	8000_Mixed	54	50	4	2	4	B
58161120	Brown rice casserole with cheese	8000_Mixed	54	50	4	2	3	D
75440510	Vegetable combinations, excluding carrots, broccoli, and dark-green leafy; cooked, with cheese sauce	8000_Mixed	54	50	4	2	3.5	C
77141010	Potato chicken pie, Puerto Rican style	8000_Mixed	54	50	4	2	2	D
27350200	Oyster pie	8000_Mixed	54	49	5	2	2.5	C
58134810	Cannelloni, cheese- and spinach-filled, no sauce	8000_Mixed	54	49	5	2	3	C
73502000	Squash, winter type, soup, home recipe, canned, or ready-to-serve	8000_Mixed	54	49	5	2	3	C
27311635	Beef, potatoes, and vegetables including carrots, broccoli, and/or dark-green leafy; cheese sauce	8000_Mixed	54	48	6	2	4	B
27360120	Chow mein or chop suey, various types of meat, with noodles	8000_Mixed	54	48	6	1	4	A

27430590	Veal with vegetables excluding carrots, broccoli, and dark-green leafy; and potatoes, cream or white sauce	8000_Mixed	54	48	6	1	3.5	B
27550420	Fish sandwich, grilled	8000_Mixed	54	48	6	3	4	B
58130014	Lasagna with meat, from restaurant	8000_Mixed	54	48	6	1	3	D
58137210	Pad Thai, NFS	8000_Mixed	54	48	6	1	3.5	C
58137230	Pad Thai with chicken	8000_Mixed	54	48	6	1	3.5	C
58156610	Pigeon pea asopao, Asopao de gandules	8000_Mixed	54	48	6	1	3.5	C
75417013	Peas, from canned, creamed	8000_Mixed	54	48	6	2	3.5	C
27250900	Fish and noodles with mushroom sauce	8000_Mixed	54	47	7	2	3.5	B
27320027	Ham or pork, noodles, and vegetables including carrots, broccoli, and/or dark-green leafy; no sauce	8000_Mixed	54	47	7	1	3.5	B
58404030	Chicken or turkey rice soup, home recipe	8000_Mixed	54	47	7	1	3.5	B
71802010	Macaroni and potato soup	8000_Mixed	54	47	7	1	3.5	B
27243100	Biryani with chicken	8000_Mixed	54	46	8	1	4	A
27145000	Chicken or turkey with teriyaki	8000_Mixed	54	45	9	1	3.5	D
27250610	Tuna noodle casserole with cream or white sauce	8000_Mixed	54	45	9	2	3.5	C
27446260	Chicken or turkey salad, made with any type of fat free dressing	8000_Mixed	54	45	9	2	3.5	C
27250130	Shrimp and noodles with cheese sauce	8000_Mixed	54	44	10	2	3.5	B
27250126	Shrimp and noodles with cream or white sauce	8000_Mixed	54	43	11	1	3.5	B
27116400	Steak tartare	8000_Mixed	54	42	12	1	3.5	B
75649030	Vegetable soup, canned, low sodium, prepared with water or ready-to-serve	8000_Mixed	53	58	-5	4	3.5	B
42302060	Peanut butter and jelly sandwich, with reduced fat peanut butter, regular jelly, on wheat bread	8000_Mixed	53	56	-3	3	2.5	D
42302065	Peanut butter and jelly sandwich, with reduced fat peanut butter, regular jelly, on whole wheat bread	8000_Mixed	53	56	-3	3	2.5	D
27550750	Tuna salad submarine sandwich, with lettuce and tomato	8000_Mixed	53	55	-2	4	3.5	C
27550751	Tuna salad submarine sandwich, with cheese, lettuce and tomato	8000_Mixed	53	55	-2	4	3.5	C
27446320	Chicken or turkey, breaded, fried, garden salad with bacon and cheese, chicken and/or turkey, bacon, cheese, lettuce and/or greens, tomato and/or carrots, other vegetables, no dressing	8000_Mixed	53	53	0	3	4	A
27550800	Seafood salad sandwich	8000_Mixed	53	53	0	4	3.5	C
58148116	Macaroni or pasta salad, made with creamy dressing	8000_Mixed	53	53	0	2	3.5	C
24209001	Turkey with barbecue sauce, skin not eaten	8000_Mixed	53	49	4	2	3.5	B
27243500	Chicken or turkey and rice with tomato-based sauce	8000_Mixed	53	49	4	1	3.5	C
27320450	Ham, potatoes, and vegetables including carrots, broccoli, and/or dark-green leafy; no sauce	8000_Mixed	53	49	4	2	3.5	C
58146342	Pasta with tomato-based sauce and poultry, home recipe	8000_Mixed	53	49	4	1	3.5	B
58146702	Pasta, whole grain, with cream sauce and meat, home recipe	8000_Mixed	53	49	4	2	3.5	C
71801100	Potato and cheese soup	8000_Mixed	53	49	4	2	3.5	B
27250050	Fish cake or patty, NS as to fish	8000_Mixed	53	48	5	3	3.5	C
27250310	Haddock cake or patty	8000_Mixed	53	48	5	3	3.5	C
27320350	Pork, rice, and vegetables excluding carrots, broccoli, and dark-green leafy; tomato-based sauce	8000_Mixed	53	48	5	1	3.5	C

27343410	Chicken or turkey, noodles, and vegetables including carrots, broccoli, and/or dark-green leafy; gravy	8000_Mixed	53	48	5	2	3.5	C
27343470	Chicken or turkey, noodles, and vegetables including carrots, broccoli, and/or dark-green leafy; cream sauce, white sauce, or mushroom sauce	8000_Mixed	53	48	5	2	3.5	C
27550410	Fish sandwich, fried, on wheat bun	8000_Mixed	53	48	5	3	4	B
58104190	Nachos with chicken, cheese, and sour cream	8000_Mixed	53	48	5	3	3.5	C
27118180	Beef stew, meat with gravy, no potatoes, Puerto Rican style	8000_Mixed	53	47	6	1	3.5	D
27320310	Pork chow mein or chop suey with noodles	8000_Mixed	53	47	6	1	4	B
58100600	Enchilada with chicken, tomato-based sauce	8000_Mixed	53	47	6	3	4	A
75414013	Mushrooms, from canned, creamed	8000_Mixed	53	47	6	1	3.5	C
75418101	Turnips, from fresh, creamed	8000_Mixed	53	47	6	1	3.5	C
73501010	Carrot with rice soup, cream of, prepared with milk, home recipe, canned or ready-to-serve	8000_Mixed	53	46	7	1	3.5	B
75460710	Vegetable combinations, excluding carrots, broccoli, and dark-green leafy; cooked, with pasta	8000_Mixed	53	46	7	1	4	A
27350090	Fish, noodles, and vegetables including carrots, broccoli, and/or dark green leafy; cheese sauce	8000_Mixed	53	45	8	2	3.5	C
28315150	Meat and corn hominy soup, home recipe, Mexican style	8000_Mixed	53	45	8	1	3.5	B
58163130	Dirty rice	8000_Mixed	53	45	8	1	3.5	B
27211550	Stewed, seasoned, ground beef with potatoes, Mexican style	8000_Mixed	53	44	9	1	4	B
28340220	Chicken soup with noodles and potatoes, Puerto Rican style	8000_Mixed	53	44	9	1	3.5	B
58120110	Crepe, filled with meat, poultry, or seafood, with sauce	8000_Mixed	53	44	9	2	3.5	C
75411010	Corn, scalloped or pudding	8000_Mixed	53	44	9	2	3.5	C
27120100	Ham or pork with tomato-based sauce	8000_Mixed	53	43	10	1	4	A
27261500	Stewed, seasoned, ground beef and pork with potatoes, Mexican style	8000_Mixed	53	43	10	1	4	A
28340210	Chicken rice soup, Puerto Rican style	8000_Mixed	53	43	10	1	3.5	B
58100900	Enchilada with seafood, tomato-based sauce	8000_Mixed	53	43	10	4	4	B
58131610	Ravioli, cheese and spinach filled, with tomato sauce	8000_Mixed	53	43	10	2	3.5	C
27136050	Venison or deer with tomato-based sauce	8000_Mixed	53	42	11	1	4	A
75140500	Broccoli salad with cauliflower, cheese, bacon bits, and dressing	8000_Mixed	52	60	-8	3	3	D
42303010	Peanut butter and banana sandwich	8000_Mixed	52	51	1	3	3.5	C
27146110	Sweet and sour chicken or turkey, without vegetables	8000_Mixed	52	48	4	1	3.5	C
27146155	Chicken curry with rice	8000_Mixed	52	48	4	1	3.5	C
58101935	Taco or tostada salad with chicken	8000_Mixed	52	48	4	3	3.5	C
58132310	Spaghetti with tomato sauce and meatballs or spaghetti with meat sauce or spaghetti with meat sauce and meatballs	8000_Mixed	52	48	4	2	3.5	C
58146302	Pasta with tomato-based sauce, and added vegetables, home recipe	8000_Mixed	52	48	4	1	3.5	B
58160550	Rice, white, with dark green vegetables, NS as to fat	8000_Mixed	52	48	4	1	3.5	B
27315220	Beef, rice, and vegetables excluding carrots, broccoli, and/or dark-green leafy; tomato-based sauce	8000_Mixed	52	47	5	1	3.5	B
27345010	Chicken or turkey, rice, and vegetables including carrots, broccoli, and/or dark-green leafy; no sauce	8000_Mixed	52	47	5	1	3.5	C

27445150	General Tso chicken	8000_Mixed	52	47	5	1	3	D
27510643	Hamburger, 1 medium patty, with condiments, on whole wheat bun	8000_Mixed	52	47	5	2	3.5	C
58104130	Nachos with meat and cheese	8000_Mixed	52	47	5	2	3.5	C
27220520	Ham or pork and potatoes with cheese sauce	8000_Mixed	52	46	6	2	3.5	B
27446410	Chicken or turkey and vegetables excluding carrots, broccoli, and dark-green leafy; no potatoes, cheese sauce	8000_Mixed	52	46	6	2	3.5	C
58104250	Nachos with chicken or turkey and cheese	8000_Mixed	52	46	6	3	3.5	D
58134710	Tortellini, spinach-filled, with tomato sauce	8000_Mixed	52	46	6	2	3	C
75414010	Mushrooms, NS as to form, creamed	8000_Mixed	52	46	6	1	3.5	C
27311640	Beef, potatoes, and vegetables excluding carrots, broccoli, and dark-green leafy; cheese sauce	8000_Mixed	52	45	7	2	4	B
27313010	Beef, noodles, and vegetables including carrots, broccoli, and/or dark-green leafy; no sauce	8000_Mixed	52	45	7	1	3.5	B
28331110	Lamb, pasta, and vegetable soup, Puerto Rican style	8000_Mixed	52	45	7	1	3	C
27320025	Ham or pork, noodles and vegetables excluding carrots, broccoli, and dark-green leafy; no sauce	8000_Mixed	52	44	8	1	3.5	B
27420010	Cabbage with ham hocks	8000_Mixed	52	44	8	3	4.5	A
27461010	Stewed seasoned ground beef, Puerto Rican style (Picadillo para relleno)	8000_Mixed	52	44	8	1	3.5	D
58131600	Ravioli, cheese and spinach-filled, with cream sauce	8000_Mixed	52	44	8	2	3	C
58160160	Hopping John	8000_Mixed	52	44	8	1	4	B
27146200	Chicken or turkey with cheese sauce	8000_Mixed	52	43	9	2	3.5	D
27350100	Fish, noodles, and vegetables excluding carrots, broccoli, and dark-green leafy; cheese sauce	8000_Mixed	52	43	9	2	3.5	C
27421010	Stuffed christophine, Puerto Rican style	8000_Mixed	52	43	9	1	4	C
27221110	Stuffed pork roast, Puerto Rican style	8000_Mixed	52	42	10	1	4	A
27146160	Chicken with mole sauce	8000_Mixed	52	41	11	1	3.5	B
27148010	Stuffed chicken, drumstick or breast, Puerto Rican style	8000_Mixed	52	41	11	2	2	D
58157310	Congee, with meat, poultry, and/or seafood, and vegetables	8000_Mixed	52	41	11	1	3.5	B
14640046	Cheese sandwich, reduced fat Cheddar cheese, on wheat bread, with mayonnaise	8000_Mixed	51	58	-7	4	2	D
27564570	Frankfurter or hot dog sandwich, meatless, on bread, with meatless chili	8000_Mixed	51	57	-6	4	4	B
58401200	Barley soup, sweet, with or without nuts, Asian Style	8000_Mixed	51	56	-5	2	3.5	B
75651090	Vegetable chicken soup, canned, low sodium, prepared with water	8000_Mixed	51	56	-5	4	4	A
75649070	Vegetable soup, made from dry mix, low sodium	8000_Mixed	51	55	-4	4	3.5	B
14620300	Topping from cheese pizza	8000_Mixed	51	51	0	3	1.5	E
58148170	Macaroni or pasta salad with chicken	8000_Mixed	51	51	0	2	3.5	B
27510206	Cheeseburger, 1 small patty, with condiments, on wheat bun	8000_Mixed	51	49	2	3	3.5	D
27510207	Cheeseburger, 1 small patty, with condiments, on whole wheat bun	8000_Mixed	51	49	2	3	3.5	D
27510565	Hamburger, from school cafeteria	8000_Mixed	51	49	2	3	4	B
27510252	Cheeseburger, 1 medium patty, with condiments, on wheat bun	8000_Mixed	51	48	3	3	3.5	D
27510253	Cheeseburger, 1 medium patty, with condiments, on whole wheat bun	8000_Mixed	51	48	3	3	3.5	D

58101955	Taco or tostada salad, meatless with sour cream	8000_Mixed	51	48	3	3	3.5	C
58130150	Lasagna, with chicken or turkey, and spinach	8000_Mixed	51	48	3	2	3	D
27116110	Beef curry with rice	8000_Mixed	51	47	4	1	3.5	C
27146100	Sweet and sour chicken or turkey	8000_Mixed	51	47	4	1	3.5	C
27510587	Hamburger, 1 small patty, with condiments, on whole wheat bun	8000_Mixed	51	47	4	2	3.5	C
58146682	Pasta, whole grain, with cream sauce, home recipe	8000_Mixed	51	47	4	2	3	C
58160570	Rice, white, with dark green vegetables, fat added	8000_Mixed	51	47	4	1	3.5	B
27250710	Tuna and rice with mushroom sauce	8000_Mixed	51	46	5	2	3.5	C
27343480	Chicken or turkey, noodles, and vegetables excluding carrots, broccoli, and/or dark-green leafy; cream sauce, white sauce, or mushroom sauce	8000_Mixed	51	46	5	2	3.5	C
27510642	Hamburger, 1 medium patty, with condiments, on wheat bun	8000_Mixed	51	46	5	2	3.5	C
58137250	Pad Thai with meat	8000_Mixed	51	46	5	1	3.5	C
72202010	Broccoli casserole with noodles	8000_Mixed	51	46	5	2	4	A
27242000	Chicken or turkey and noodles, no sauce	8000_Mixed	51	45	6	1	3.5	C
27343420	Chicken or turkey, noodles, and vegetables excluding carrots, broccoli, and dark-green leafy; gravy	8000_Mixed	51	45	6	2	3.5	C
58133140	Manicotti, vegetable- and cheese-filled, with tomato sauce, meatless	8000_Mixed	51	45	6	2	3	C
58450300	Noodle soup, made with milk	8000_Mixed	51	45	6	1	3.5	C
27135050	Veal Marsala	8000_Mixed	51	44	7	1	4	A
58160210	Rice with vegetables, no sauce	8000_Mixed	51	44	7	1	3.5	B
27313020	Beef, noodles, and vegetables excluding carrots, broccoli, and dark-green leafy; no sauce	8000_Mixed	51	43	8	1	3.5	B
28315160	Italian Wedding Soup	8000_Mixed	51	43	8	2	3.5	C
58149110	Noodle pudding	8000_Mixed	51	43	8	2	3.5	C
58134720	Tortellini, spinach-filled, no sauce	8000_Mixed	51	42	9	2	3.5	C
75656010	Vegetable soup, Spanish style, stew type	8000_Mixed	51	42	9	2	4	B
77513010	Spanish stew	8000_Mixed	51	42	9	2	4	B
27143000	Chicken or turkey with cream sauce	8000_Mixed	51	41	10	1	3.5	B
58151160	Sushi, with egg, no vegetables, no seafood (no fish or shellfish), rolled in seaweed	8000_Mixed	51	39	12	1	3.5	C
74602100	Tomato soup, canned, low sodium, ready-to-serve	8000_Mixed	50	54	-4	4	3.5	B
28340600	Chicken or turkey vegetable soup, canned, prepared with water or ready-to-serve	8000_Mixed	50	52	-2	4	3.5	B
58128210	Dressing with oysters	8000_Mixed	50	50	0	4	3	C
58145300	Macaroni or noodles with cheese, whole grain	8000_Mixed	50	48	2	2	3	D
58421080	Sopa de tortilla, Mexican style tortilla soup, home recipe	8000_Mixed	50	48	2	2	3	C
75141500	Celery, stuffed with cheese	8000_Mixed	50	48	2	3	3	C
27550415	Fish sandwich, fried, on wheat bun, with cheese	8000_Mixed	50	47	3	3	3	D
27416300	Beef taco filling: beef, cheese, tomato, taco sauce	8000_Mixed	50	46	4	2	3	D
27510243	Cheeseburger, 1 medium patty, plain, on whole wheat bun	8000_Mixed	50	46	4	2	2	D

58104090	Nachos with cheese and sour cream	8000_Mixed	50	46	4	3	3.5	C
58148117	Macaroni or pasta salad, made with light creamy dressing	8000_Mixed	50	46	4	2	3.5	B
58156510	Soupy rice from Puerto Rican style Asopao de Pollo (chicken parts reported separately)	8000_Mixed	50	46	4	2	3.5	B
24209000	Turkey with barbecue sauce, skin eaten	8000_Mixed	50	45	5	2	3.5	D
27135020	Veal scallopini	8000_Mixed	50	45	5	2	3.5	C
27510577	Hamburger, 1 small patty, plain, on whole wheat bun	8000_Mixed	50	45	5	2	3.5	D
27510633	Hamburger, 1 medium patty, plain, on whole wheat bun	8000_Mixed	50	45	5	2	3.5	D
58100510	Enchilada with beef and beans	8000_Mixed	50	45	5	3	4	B
58146110	Pasta with meat sauce	8000_Mixed	50	45	5	1	3.5	B
58146332	Pasta with tomato-based sauce, meat, and added vegetables, home recipe	8000_Mixed	50	45	5	1	4	B
58160670	Rice, white, with carrots, dark green vegetables, and tomatoes and/or tomato-based sauce, NS as to fat	8000_Mixed	50	45	5	1	3.5	C
75652030	Vegetable beef soup, canned, prepared with milk	8000_Mixed	50	45	5	3	3.5	C
27211500	Beef and potatoes with cheese sauce	8000_Mixed	50	44	6	2	3.5	B
27220110	Pork and rice with tomato-based sauce	8000_Mixed	50	44	6	1	3.5	C
27231000	Lamb or mutton and potatoes with gravy	8000_Mixed	50	44	6	2	3.5	B
27313310	Beef, noodles, and vegetables including carrots, broccoli, and/or dark-green leafy; mushroom sauce	8000_Mixed	50	44	6	2	3.5	C
58100210	Burrito with chicken and beans	8000_Mixed	50	44	6	3	4	B
58156710	Rice with stewed beans, Puerto Rican style	8000_Mixed	50	44	6	1	3	C
72305000	Escarole soup	8000_Mixed	50	44	6	1	3	C
75604010	Corn soup, cream of, prepared with milk	8000_Mixed	50	44	6	2	3.5	C
75411030	Corn, cooked, NS as to form, with cream sauce, made with milk	8000_Mixed	50	43	7	1	3.5	C
75411031	Corn, cooked, from fresh, with cream sauce, made with milk	8000_Mixed	50	43	7	1	3.5	C
75411032	Corn, cooked, from frozen, with cream sauce, made with milk	8000_Mixed	50	43	7	1	3.5	C
27250110	Scallops and noodles with cheese sauce	8000_Mixed	50	42	8	2	3.5	C
27350020	Paella with seafood	8000_Mixed	50	42	8	1	3.5	C
28311010	Pepperpot soup	8000_Mixed	50	42	8	2	3.5	B
28340620	Turkey noodle soup, chunky style	8000_Mixed	50	42	8	2	3.5	B
58134160	Stuffed shells, cheese- and spinach- filled, no sauce	8000_Mixed	50	42	8	2	3	C
27130050	Lamb or mutton goulash	8000_Mixed	50	41	9	1	4	A
27214110	Meat loaf made with beef, with tomato-based sauce	8000_Mixed	50	41	9	1	3.5	C
27260100	Meat loaf made with beef and pork, with tomato-based sauce	8000_Mixed	50	41	9	2	3.5	C
28320120	Pork vegetable soup with noodles, stew type, chunky style	8000_Mixed	50	40	10	2	3	C
27111000	Beef with tomato-based sauce	8000_Mixed	50	39	11	1	4	A
27460490	Julienne salad, meat, cheese, eggs, vegetables, no dressing	8000_Mixed	50	39	11	2	3.5	B
27136080	Venison or deer with gravy	8000_Mixed	50	38	12	1	4	A
32300100	Egg drop soup	8000_Mixed	50	37	13	1	3	C

75145000	Seven-layer salad, lettuce salad made with a combination of onion, celery, green pepper, peas, mayonnaise, cheese, eggs, and/or bacon	8000_Mixed	49	53	-4	3	3	C
75604610	Gazpacho, canned, undiluted	8000_Mixed	49	52	-3	4	3.5	B
74203011	Tomatoes, from fresh, scalloped	8000_Mixed	49	51	-2	3	3.5	C
28145110	Turkey with vegetable, stuffing, diet frozen meal	8000_Mixed	49	50	-1	4	4	A
75651070	Vegetable rice soup, canned, prepared with water or ready-to-serve	8000_Mixed	49	50	-1	4	3.5	B
75656040	Vegetable soup, with pasta, chunky style	8000_Mixed	49	50	-1	4	3.5	B
58148110	Macaroni or pasta salad, made with mayonnaise	8000_Mixed	49	49	0	2	3.5	C
58148120	Macaroni or pasta salad with egg	8000_Mixed	49	49	0	2	3.5	C
75418040	Squash, summer, casserole, with cheese sauce	8000_Mixed	49	49	0	3	4	B
58100800	Enchilada, just cheese, meatless, no beans, red-chile or enchilada sauce	8000_Mixed	49	47	2	3	3.5	C
58100805	Enchilada, just cheese, meatless, no beans, green-chile or enchilada sauce	8000_Mixed	49	47	2	3	3.5	C
58148114	Macaroni or pasta salad, made with Italian dressing	8000_Mixed	49	47	2	2	3.5	B
27510343	Bacon cheeseburger, 1 medium patty, with condiments, on whole wheat bun	8000_Mixed	49	46	3	3	2	D
27540310	Turkey sandwich, with spread	8000_Mixed	49	46	3	3	4	A
58130140	Lasagna with chicken or turkey	8000_Mixed	49	46	3	2	3	D
58130320	Lasagna, meatless, with vegetables	8000_Mixed	49	46	3	2	3	C
58146451	Pasta with cream sauce, seafood, and added vegetables, restaurant	8000_Mixed	49	46	3	2	3	D
75400500	Artichokes, stuffed	8000_Mixed	49	46	3	3	4	C
58101930	Taco or tostada salad with meat	8000_Mixed	49	45	4	3	3.5	C
58101950	Taco or tostada salad with chicken and sour cream	8000_Mixed	49	45	4	3	3.5	C
58130020	Lasagna with meat and spinach	8000_Mixed	49	45	4	2	3	D
58148115	Macaroni or pasta salad, made with light Italian dressing	8000_Mixed	49	45	4	1	3.5	B
75460900	Chow mein or chop suey, meatless, with noodles	8000_Mixed	49	45	4	1	4	B
27213100	Beef and rice with tomato-based sauce	8000_Mixed	49	44	5	1	3.5	C
27311310	Beef stew with potatoes and vegetables including carrots, broccoli, and/or dark-green leafy; tomato-based sauce	8000_Mixed	49	44	5	3	3.5	B
27345210	Chicken or turkey, rice, and vegetables including carrots, broccoli, and/or dark-green leafy; gravy	8000_Mixed	49	44	5	2	3.5	C
27345410	Chicken or turkey, rice, and vegetables including carrots, broccoli, and/or dark-green leafy; cream sauce, white sauce, or mushroom sauce	8000_Mixed	49	44	5	2	3.5	C
27360000	Stew, NFS	8000_Mixed	49	44	5	3	3.5	B
58104140	Nachos with beef and cheese	8000_Mixed	49	44	5	2	2	D
58160460	Rice, white, with carrots, NS as to fat	8000_Mixed	49	44	5	1	3.5	C
58160480	Rice, white, with carrots, fat added	8000_Mixed	49	44	5	1	3.5	C
58160610	Rice, white, with dark green vegetables and tomatoes and/or tomato-based sauce, NS as to fat	8000_Mixed	49	44	5	1	3.5	C
58160630	Rice, white, with dark green vegetables and tomatoes and/or tomato-based sauce, fat added	8000_Mixed	49	44	5	1	3.5	C

58160640	Rice, white, with carrots and dark green vegetables, NS as to fat	8000_Mixed	49	44	5	1	3.5	B
58160660	Rice, white, with carrots and dark green vegetables, fat added	8000_Mixed	49	44	5	1	3.5	B
27313410	Beef, noodles, and vegetables including carrots, broccoli, and/or dark-green leafy; gravy	8000_Mixed	49	43	6	1	3.5	C
27330060	Lamb or mutton, rice, and vegetables including carrots, broccoli, and/or dark-green leafy; tomato-based sauce	8000_Mixed	49	43	6	1	3.5	C
27345020	Chicken or turkey, rice, and vegetables excluding carrots, broccoli, and dark-green leafy; no sauce	8000_Mixed	49	43	6	1	3.5	C
58160520	Rice, white, with tomatoes and/or tomato-based sauce, NS as to fat	8000_Mixed	49	43	6	1	3.5	C
58160580	Rice, white, with carrots and tomatoes and/or tomato-based sauce, NS as to fat	8000_Mixed	49	43	6	1	3.5	C
58160600	Rice, white, with carrots and tomatoes and/or tomato-based sauce, fat added	8000_Mixed	49	43	6	1	3.5	C
27418410	Beef steak with onions, Puerto Rican style	8000_Mixed	49	42	7	2	1	E
28340120	Chicken or turkey broth, without tomato, home recipe	8000_Mixed	49	42	7	1	3.5	A
58115110	Tamale casserole, Puerto Rican style	8000_Mixed	49	42	7	1	3.5	C
58134130	Stuffed shells, cheese-filled, with meat sauce	8000_Mixed	49	42	7	2	3.5	C
27213010	Biryani with meat	8000_Mixed	49	41	8	1	4	B
27550150	Fried seafood sandwich	8000_Mixed	49	41	8	3	3.5	C
41601180	Bean and ham soup, home recipe	8000_Mixed	49	41	8	2	3.5	B
58155410	Soupy rice with chicken, Puerto Rican style	8000_Mixed	49	40	9	1	3.5	C
27120090	Ham or pork with mushroom sauce	8000_Mixed	49	39	10	1	4	B
58151220	Sushi roll tuna	8000_Mixed	49	39	10	1	3.5	B
58155210	Stuffed rice with chicken, Dominican style (Arroz relleno Dominicano)	8000_Mixed	49	39	10	1	2	D
27162010	Meat with tomato-based sauce	8000_Mixed	49	38	11	1	4	A
58128110	Chicken cornbread	8000_Mixed	49	38	11	2	3.5	C
58151450	Sushi, topped with egg	8000_Mixed	49	36	13	1	3.5	C
14640036	Cheese sandwich, Cheddar cheese, on whole wheat bread, with mayonnaise	8000_Mixed	48	54	-6	4	1.5	D
75141005	Cabbage salad or coleslaw, made with light coleslaw dressing	8000_Mixed	48	54	-6	3	3.5	C
27550740	Tuna salad sandwich, on bun	8000_Mixed	48	51	-3	4	3.5	C
41602070	Split pea soup, canned, reduced sodium, prepared with water or ready-to-serve	8000_Mixed	48	50	-2	4	4	A
14640024	Cheese sandwich, reduced fat Cheddar cheese, on whole wheat bread, no spread	8000_Mixed	48	49	-1	4	2.5	D
28143150	Chicken and vegetable entree with noodles, diet frozen meal	8000_Mixed	48	49	-1	4	4	A
28141610	Chicken and vegetables in cream or white sauce, diet frozen meal	8000_Mixed	48	48	0	4	4	A
75603010	Celery soup, cream of, prepared with milk, home recipe, canned or ready-to-serve	8000_Mixed	48	45	3	3	3	C
58128120	Cornmeal dressing with chicken or turkey and vegetables	8000_Mixed	48	44	4	2	3	D
58145111	Macaroni or noodles with cheese, from restaurant	8000_Mixed	48	44	4	1	3	C
58421010	Sopa Seca de Fideo, Mexican style, made with dry noodles, home recipe	8000_Mixed	48	44	4	2	3	C
72202020	Broccoli casserole with rice	8000_Mixed	48	44	4	2	3.5	B

27120060	Sweet and sour pork	8000_Mixed	48	43	5	2	3.5	C
27347240	Chicken or turkey, dumplings, and vegetables including carrots, broccoli, and/or dark green leafy; gravy	8000_Mixed	48	43	5	2	3.5	C
58104310	Chalupa with beans, chicken, cheese, lettuce and tomato	8000_Mixed	48	43	5	3	3.5	C
58130015	Lasagna with meat, home recipe	8000_Mixed	48	43	5	2	3	D
58160540	Rice, white, with tomatoes and/or tomato-based sauce, fat added	8000_Mixed	48	43	5	1	3.5	C
77230210	Cassava Pasteles, Puerto Rican style	8000_Mixed	48	43	5	2	2.5	D
27250830	Fish and rice with mushroom sauce	8000_Mixed	48	42	6	2	3.5	B
27443110	Chicken or turkey a la king with vegetables including carrots, broccoli, and/or dark-green leafy; no potatoes, cream, white, or soup-based sauce	8000_Mixed	48	42	6	2	3	D
58100400	Enchilada with beef, no beans	8000_Mixed	48	42	6	3	4	B
58146210	Pasta with sauce, NFS	8000_Mixed	48	42	6	1	3.5	B
58146322	Pasta with tomato-based sauce and meat, home recipe	8000_Mixed	48	42	6	1	3.5	B
58160220	Rice with vegetables, tomato-based sauce (mixture)	8000_Mixed	48	42	6	1	3.5	C
58160690	Rice, white, with carrots, dark green vegetables, and tomatoes and/or tomato-based sauce, fat added	8000_Mixed	48	42	6	1	3.5	C
27242200	Chicken or turkey and noodles with gravy	8000_Mixed	48	41	7	2	3.5	C
27313320	Beef, noodles, and vegetables excluding carrots, broccoli, and dark-green leafy; mushroom sauce	8000_Mixed	48	41	7	2	3.5	C
27313420	Beef, noodles, and vegetables excluding carrots, broccoli, and dark-green leafy; gravy	8000_Mixed	48	41	7	1	3.5	C
27343950	Chicken or turkey, noodles, and vegetables including carrots, broccoli, and/or dark-green leafy; cheese sauce	8000_Mixed	48	41	7	2	3.5	C
27540330	Turkey sandwich, with gravy	8000_Mixed	48	41	7	3	4	A
41601060	Bean soup, with macaroni and meat	8000_Mixed	48	41	7	2	3.5	C
58136150	Lo mein, with beef	8000_Mixed	48	41	7	1	4	B
71851010	Plantain soup, Puerto Rican style	8000_Mixed	48	41	7	2	3	C
58136160	Lo mein, with chicken	8000_Mixed	48	40	8	1	3.5	C
58146120	Pasta with tomato-based sauce, cheese and meat	8000_Mixed	48	40	8	2	3.5	C
27360010	Goulash, NFS	8000_Mixed	48	39	9	1	3.5	B
27120020	Ham or pork with gravy	8000_Mixed	48	38	10	1	4	A
58131590	Ravioli, cheese and spinach-filled, no sauce	8000_Mixed	48	38	10	1	3.5	C
58155510	Soupy rice mixture with chicken and potatoes, Puerto Rican style	8000_Mixed	48	38	10	1	3.5	B
28320110	Pork and rice soup, stew type, chunky style	8000_Mixed	48	37	11	1	3.5	B
58131320	Ravioli, meat-filled, with tomato sauce or meat sauce	8000_Mixed	48	37	11	1	3	C
27162500	Stewed, seasoned, ground beef and pork, Mexican style	8000_Mixed	48	36	12	1	4	B
27150130	Seafood newburg	8000_Mixed	48	34	14	2	1.5	D
14640040	Cheese sandwich, reduced fat American cheese,, on wheat bread, with mayonnaise	8000_Mixed	47	57	-10	4	2	D
27540230	Chicken patty sandwich with cheese, on wheat bun, with lettuce, tomato and spread	8000_Mixed	47	54	-7	4	2.5	D
27550720	Tuna salad sandwich, on bread	8000_Mixed	47	51	-4	4	3.5	C
27550745	Tuna salad sandwich, on bun, with cheese	8000_Mixed	47	50	-3	4	3	D

75649010	Vegetable soup, canned, prepared with water or ready-to-serve	8000_Mixed	47	50	-3	4	3.5	B
75656020	Vegetable soup, chunky style	8000_Mixed	47	50	-3	4	3.5	B
28355130	Clam chowder, New England, prepared with water	8000_Mixed	47	48	-1	4	3.5	C
28143180	Chicken in butter sauce with potatoes and vegetable, diet frozen meal	8000_Mixed	47	47	0	4	4.5	A
58148180	Macaroni or pasta salad with cheese	8000_Mixed	47	47	0	3	3	C
73305010	Squash, winter, baked with cheese	8000_Mixed	47	46	1	3	3.5	C
58105075	Fajita with vegetables	8000_Mixed	47	45	2	3	3	C
27146360	Sesame chicken	8000_Mixed	47	44	3	1	2	D
27420250	Ham and vegetables including carrots broccoli, and/or dark- green leafy; no potatoes, no sauce	8000_Mixed	47	44	3	2	3	D
27510342	Bacon cheeseburger, 1 medium patty, with condiments, on wheat bun	8000_Mixed	47	44	3	3	2	D
75611010	Vegetable soup, cream of, prepared with milk	8000_Mixed	47	44	3	2	3	C
27211190	Beef and potatoes with cream sauce, white sauce or mushroom sauce	8000_Mixed	47	42	5	2	3.5	C
27540195	Chicken fillet sandwich, grilled, on white bun	8000_Mixed	47	42	5	3	4	A
27242250	Chicken or turkey and noodles with mushroom sauce	8000_Mixed	47	41	6	2	3.5	C
27311210	Corned beef, potatoes, and vegetables including carrots, broccoli, and/or dark-green leafy; no sauce	8000_Mixed	47	41	6	2	4	A
27320410	Ham, potatoes, and vegetables excluding carrots, broccoli, and dark-green leafy; no sauce	8000_Mixed	47	41	6	2	4	A
27345220	Chicken or turkey, rice, and vegetables excluding carrots, broccoli, and dark-green leafy; gravy	8000_Mixed	47	41	6	2	3.5	C
58160490	Rice, white, with peas and carrots, NS as to fat	8000_Mixed	47	41	6	1	3.5	B
58160510	Rice, white, with peas and carrots, fat added	8000_Mixed	47	41	6	1	3.5	B
74604500	Tomato noodle soup, canned, prepared with water or ready-to-serve	8000_Mixed	47	41	6	2	3.5	B
75460800	Vegetable combinations, including carrots, broccoli, and/or dark-green leafy; cooked, with butter sauce and pasta	8000_Mixed	47	41	6	1	3	C
27315010	Beef, rice, and vegetables including carrots, broccoli, and/or dark-green leafy; no sauce	8000_Mixed	47	40	7	1	3.5	B
27343960	Chicken or turkey, noodles, and vegetables excluding carrots, broccoli, and dark-green leafy; cheese sauce	8000_Mixed	47	40	7	2	3.5	C
58160430	Rice, white, with peas, NS as to fat	8000_Mixed	47	40	7	1	3.5	B
58160450	Rice, white, with peas, fat added	8000_Mixed	47	40	7	1	3.5	B
27246200	Chicken or turkey with stuffing	8000_Mixed	47	39	8	2	4	B
27320030	Ham or pork, noodles and vegetables excluding carrots, broccoli, and dark-green leafy; cheese sauce	8000_Mixed	47	39	8	2	3.5	C
27161010	Meat loaf, Puerto Rican style	8000_Mixed	47	38	9	1	1.5	D
27214100	Meat loaf made with beef	8000_Mixed	47	37	10	1	3.5	C
27246100	Chicken or turkey with dumplings	8000_Mixed	47	37	10	1	3.5	B
27260080	Meat loaf made with beef and pork	8000_Mixed	47	37	10	1	3.5	C
27260090	Meat loaf made with beef, veal and pork	8000_Mixed	47	37	10	1	3.5	C
28315110	Beef noodle soup, chunky style	8000_Mixed	47	37	10	1	3.5	B
28360210	Spanish vegetable soup, Puerto Rican style	8000_Mixed	47	37	10	2	3.5	C

32301100	Garlic egg soup, Puerto Rican style	8000_Mixed	47	37	10	2	3	C
58100350	Burrito with eggs and cheese, no beans	8000_Mixed	47	37	10	3	3	D
58137300	Adobo, with noodles	8000_Mixed	47	36	11	1	3.5	C
58150530	Adobo, with rice	8000_Mixed	47	36	11	1	3.5	C
75418060	Squash, summer, souffle	8000_Mixed	47	36	11	1	3	C
58151210	Sushi roll, shrimp	8000_Mixed	47	30	17	1	3.5	C
74602200	Tomato soup, canned, reduced sodium, prepared with water, or ready-to-serve	8000_Mixed	46	54	-8	4	3.5	B
27564430	Frankfurter or hot dog sandwich, meatless, plain, on bread	8000_Mixed	46	53	-7	4	4	B
14640048	Cheese sandwich, reduced fat Cheddar cheese, on whole wheat bread, with mayonnaise	8000_Mixed	46	52	-6	4	2	D
74606020	Tomato vegetable soup with noodles, prepared with water	8000_Mixed	46	50	-4	4	3.5	C
27141000	Chicken or turkey cacciatore	8000_Mixed	46	47	-1	4	3.5	D
42301010	Peanut butter sandwich, NFS	8000_Mixed	46	47	-1	3	2.5	D
42301015	Peanut butter sandwich, with regular peanut butter, on white bread	8000_Mixed	46	47	-1	3	2.5	D
41210000	Bean cake	8000_Mixed	46	46	0	2	2.5	D
28150810	Scallops with potatoes, vegetable (frozen meal)	8000_Mixed	46	45	1	4	3.5	C
58104080	Nachos with beef, beans, cheese, and sour cream	8000_Mixed	46	44	2	3	3.5	D
27545010	Turkey or chicken burger, with condiments, on bun, from fast food / restaurant	8000_Mixed	46	43	3	3	3.5	C
27545200	Turkey or chicken burger, with condiments, on white bun	8000_Mixed	46	43	3	3	3.5	C
28152050	Shrimp with rice, vegetable (frozen meal)	8000_Mixed	46	43	3	4	3.5	C
27350070	Tuna pot pie	8000_Mixed	46	42	4	3	3.5	C
27540153	Chicken fillet sandwich, grilled, from fast food, with cheese	8000_Mixed	46	42	4	3	3.5	D
58101945	Taco or tostada salad with meat and sour cream	8000_Mixed	46	42	4	2	3.5	C
27345420	Chicken or turkey, rice, and vegetables excluding carrots, broccoli, and dark-green leafy; cream sauce, white sauce, or mushroom sauce	8000_Mixed	46	41	5	2	3.5	C
27540152	Chicken fillet sandwich, grilled, from fast food	8000_Mixed	46	41	5	3	4	B
27545100	Turkey or chicken burger, on white bun	8000_Mixed	46	41	5	2	4	A
28345160	Chicken or turkey mushroom soup, cream of, prepared with milk	8000_Mixed	46	41	5	3	3.5	C
58100180	Burrito with pork and beans	8000_Mixed	46	41	5	3	4	B
75600150	Soup, cream of, NFS	8000_Mixed	46	41	5	3	3.5	B
75602010	Cauliflower soup, cream of, prepared with milk	8000_Mixed	46	41	5	1	3.5	C
75607080	Mushroom with chicken soup, cream of, prepared with milk	8000_Mixed	46	41	5	3	3.5	C
27315310	Beef, rice, and vegetables including carrots, broccoli, and/or dark-green leafy; mushroom sauce	8000_Mixed	46	40	6	2	3.5	C
27315410	Beef, rice, and vegetables including carrots, broccoli, and/or dark-green leafy; gravy	8000_Mixed	46	40	6	2	3.5	C
27320500	Sweet and sour pork with rice	8000_Mixed	46	40	6	2	3.5	C
58163410	Spanish rice, fat added	8000_Mixed	46	40	6	1	3.5	C
27243300	Chicken or turkey and rice with cream sauce	8000_Mixed	46	39	7	2	3.5	C

27443120	Chicken or turkey a la king with vegetables excluding carrorts, broccoli, and dark-green leafy; no potatoes, cream, white, or soup-based sauce	8000_Mixed	46	39	7	2	3	D
27118120	Stewed seasoned ground beef, Puerto Rican style	8000_Mixed	46	38	8	2	2	D
27242350	Chicken or turkey tetrazzini	8000_Mixed	46	38	8	2	3.5	B
58101510	Taco or tostada with chicken or turkey, lettuce, tomato and salsa	8000_Mixed	46	38	8	3	4	A
58136140	Lo mein, with pork	8000_Mixed	46	38	8	1	3.5	C
58135110	Chow fun noodles with meat and vegetables	8000_Mixed	46	37	9	1	4	A
27260010	Meat loaf, NS as to type of meat	8000_Mixed	46	36	10	1	3.5	D
27411150	Beef rolls, stuffed with vegetables or meat mixture, tomato-based sauce	8000_Mixed	46	36	10	1	4	B
58131110	Ravioli, NS as to filling, with tomato sauce	8000_Mixed	46	36	10	2	3	C
58147340	Macaroni or noodles, creamed, with cheese and tuna	8000_Mixed	46	35	11	2	3.5	C
27150060	Lobster newburg	8000_Mixed	46	33	13	2	1.5	D
14640034	Cheese sandwich, Cheddar cheese, on wheat bread, with mayonnaise	8000_Mixed	45	51	-6	4	1.5	D
75651040	Vegetable noodle soup, canned, prepared with water, or ready-to-serve	8000_Mixed	45	49	-4	4	3.5	B
75654010	Vegetarian vegetable soup, prepared with water	8000_Mixed	45	49	-4	4	3.5	B
27550730	Tuna salad sandwich, on bread, with cheese	8000_Mixed	45	48	-3	4	3	D
14640070	Cheese sandwich, reduced fat Cheddar cheese, on wheat bread, with butter	8000_Mixed	45	47	-2	3	1.5	E
42301115	Peanut butter sandwich, with reduced fat peanut butter, on white bread	8000_Mixed	45	47	-2	3	3.5	D
58146373	Pasta with tomato-based sauce, seafood, and added vegetables, ready-to-heat	8000_Mixed	45	47	-2	4	3.5	B
58146363	Pasta with tomato-based sauce and seafood, ready-to-heat	8000_Mixed	45	46	-1	4	3.5	C
27347200	Chicken or turkey, stuffing, and vegetables including carrots, broccoli, and/or dark-green leafy; no sauce	8000_Mixed	45	45	0	3	3.5	C
51136000	Bruschetta	8000_Mixed	45	43	2	3	3.5	C
75607100	Mushroom soup, cream of, canned, reduced sodium, prepared with milk	8000_Mixed	45	43	2	3	3.5	C
27540196	Chicken fillet sandwich, grilled, on white bun, with cheese	8000_Mixed	45	42	3	3	3.5	C
27550710	Tuna salad sandwich, with lettuce	8000_Mixed	45	42	3	4	3.5	B
58146441	Pasta with cream sauce and seafood, restaurant	8000_Mixed	45	42	3	2	2.5	D
27116300	Beef with sweet and sour sauce	8000_Mixed	45	40	5	2	3.5	C
27347250	Chicken or turkey, dumplings, and vegetables excluding carrots, broccoli, and dark green leafy; gravy	8000_Mixed	45	40	5	2	3.5	C
27420270	Ham and vegetables excluding carrots, broccoli, and dark-green leafy; no potatoes, no sauce	8000_Mixed	45	40	5	2	3	D
27516010	Gyro sandwich (pita bread, beef, lamb, onion, condiments), with tomato and spread	8000_Mixed	45	40	5	3	4	B
74601010	Tomato soup, cream of, prepared with milk	8000_Mixed	45	40	5	3	3.5	B
27146250	Chicken or turkey cordon bleu	8000_Mixed	45	39	6	2	3.5	D
27212120	Chili con carne with beans and macaroni	8000_Mixed	45	39	6	3	3.5	C
27510710	Pizzaburger (hamburger, cheese, sauce) on 1/2 bun	8000_Mixed	45	39	6	2	3.5	C

27545000	Turkey or chicken burger, plain, on bun, from fast food / restaurant	8000_Mixed	45	39	6	2	4	B
58122210	Gnocchi, cheese	8000_Mixed	45	39	6	3	3	C
58145130	Macaroni or noodles with cheese and beef	8000_Mixed	45	39	6	3	3.5	C
58160700	Rice, white, with other vegetables, NS as to fat	8000_Mixed	45	39	6	1	3.5	C
58160720	Rice, white, with other vegetables, fat added	8000_Mixed	45	39	6	1	3.5	C
58163430	Spanish rice, NS as to fat	8000_Mixed	45	39	6	1	3.5	C
58408500	Noodle soup with vegetables, Asian style	8000_Mixed	45	39	6	2	3.5	B
27214300	Beef wellington	8000_Mixed	45	38	7	2	2	D
58133130	Manicotti, cheese-filled, with meat sauce	8000_Mixed	45	37	8	2	3	C
27111500	Beef sloppy joe, no bun	8000_Mixed	45	36	9	2	3	C
28320140	Ham, noodle, and vegetable soup, Puerto Rican style	8000_Mixed	45	36	9	2	3	C
58131120	Ravioli, NS as to filling, with cream sauce	8000_Mixed	45	36	9	2	3.5	C
58163450	Spanish rice with ground beef	8000_Mixed	45	36	9	1	3.5	C
75418000	Squash, summer, yellow or green, breaded or battered, baked	8000_Mixed	45	35	10	1	3.5	A
27114000	Beef with mushroom sauce	8000_Mixed	45	34	11	1	4	B
27135030	Veal with cream sauce	8000_Mixed	45	34	11	1	4	B
27235000	Meat loaf made with venison/deer	8000_Mixed	45	34	11	1	3.5	B
27135010	Veal with gravy	8000_Mixed	45	33	12	1	4	A
27460510	Antipasto with ham, fish, cheese, vegetables	8000_Mixed	45	33	12	3	2	D
58157300	Congee, with meat, poultry, and/or seafood	8000_Mixed	45	33	12	1	3.5	B
27450130	Crab salad made with imitation crab	8000_Mixed	44	53	-9	4	2.5	D
58109150	Pizza, cheese and vegetables, gluten-free thick crust	8000_Mixed	44	50	-6	4	3	C
27564560	Frankfurter or hot dog sandwich, meatless, on bun, with meatless chili	8000_Mixed	44	49	-5	4	4	B
58106570	Pizza with pepperoni, from school lunch, thin crust	8000_Mixed	44	49	-5	4	3.5	D
14640190	Grilled cheese sandwich, reduced fat Cheddar cheese, on wheat bread	8000_Mixed	44	46	-2	3	1.5	E
27550755	Tuna salad wrap sandwich	8000_Mixed	44	46	-2	4	2	D
14640195	Grilled cheese sandwich, reduced fat Cheddar cheese, on whole wheat bread	8000_Mixed	44	45	-1	3	1.5	E
27540320	Turkey salad or turkey spread sandwich	8000_Mixed	44	45	-1	3	4	B
28315050	Beef vegetable soup with potato, pasta, or rice, chunky style, canned, or ready-to-serve	8000_Mixed	44	45	-1	4	3.5	C
32203010	Egg salad sandwich	8000_Mixed	44	44	0	3	3	D
42302105	Peanut butter and jelly sandwich, with regular peanut butter, reduced sugar jelly, on white bread	8000_Mixed	44	43	1	3	2.5	D
58109050	Pizza, cheese and vegetables, whole wheat thin crust	8000_Mixed	44	42	2	3	3.5	D
28340650	Chicken vegetable soup with rice, stew type, chunky style, prepared with milk	8000_Mixed	44	41	3	3	3.5	C
58148500	Pasta or macaroni salad with oil and vinegar-type dressing	8000_Mixed	44	41	3	2	3	C
27540300	Chicken fillet wrap sandwich, grilled, from fast food	8000_Mixed	44	40	4	3	2	D
58100310	Burrito with beans, meatless	8000_Mixed	44	40	4	3	3.5	C

58101600	Soft taco with bean, cheese, and lettuce	8000_Mixed	44	40	4	3	3.5	C
58421000	Sopa seca, Mexican style, NFS	8000_Mixed	44	40	4	2	3	C
75608010	Onion soup, cream of, prepared with milk	8000_Mixed	44	40	4	3	3	C
27146350	Orange chicken	8000_Mixed	44	39	5	1	2	D
27520160	Bacon, chicken, and tomato club sandwich, on multigrain roll with lettuce and spread	8000_Mixed	44	39	5	3	3.5	D
27113100	Beef stroganoff	8000_Mixed	44	38	6	2	3	C
27315320	Beef, rice, and vegetables excluding carrots, broccoli, and dark-green leafy; mushroom sauce	8000_Mixed	44	38	6	2	3.5	C
27315420	Beef, rice, and vegetables excluding carrots, broccoli, and dark-green leafy; gravy	8000_Mixed	44	38	6	2	3.5	C
27317100	Beef, dumplings, and vegetables including carrots, broccoli, and/or dark-green leafy; gravy	8000_Mixed	44	38	6	2	3.5	C
58160400	Rice, white, with corn, NS as to fat	8000_Mixed	44	38	6	1	3.5	B
58160420	Rice, white, with corn, fat added	8000_Mixed	44	38	6	1	3.5	B
58200300	Wrap sandwich, filled with meat, poultry, or fish, vegetables, rice, and cheese	8000_Mixed	44	38	6	2	3.5	C
74604600	Tomato noodle soup, canned, prepared with milk	8000_Mixed	44	38	6	2	3.5	B
75410550	Stuffed jalapeno pepper	8000_Mixed	44	38	6	3	3	D
75411033	Corn, cooked, from canned, with cream sauce, made with milk	8000_Mixed	44	38	6	2	3.5	C
75418030	Squash, summer, casserole, with rice and tomato sauce	8000_Mixed	44	38	6	2	4	A
27212400	Beef and noodles with mushroom sauce	8000_Mixed	44	37	7	2	3.5	B
27236000	Venison or deer and noodles with cream or white sauce	8000_Mixed	44	37	7	1	3.5	C
27242310	Chicken or turkey and noodles with cheese sauce	8000_Mixed	44	37	7	2	3.5	C
27319010	Stuffed green pepper, Puerto Rican style	8000_Mixed	44	37	7	2	3.5	C
58116110	Meat turnover, Puerto Rican style	8000_Mixed	44	37	7	1	3.5	C
58133120	Manicotti, cheese-filled, with tomato sauce, meatless	8000_Mixed	44	37	7	2	3	C
58136110	Lo mein, NFS	8000_Mixed	44	37	7	1	3.5	C
58136120	Lo mein, meatless	8000_Mixed	44	37	7	1	3.5	C
58147350	Macaroni, creamed, with vegetables	8000_Mixed	44	37	7	1	3.5	C
58160304	Rice with peas, fat added in cooking	8000_Mixed	44	37	7	1	3.5	C
58200100	Wrap sandwich, filled with meat, poultry, or fish, vegetables, and rice	8000_Mixed	44	37	7	2	3.5	C
27118110	Meatballs, Puerto Rican style	8000_Mixed	44	36	8	2	2	D
27212200	Beef and noodles with gravy	8000_Mixed	44	36	8	1	3.5	B
27218110	Stuffed pot roast, with potatoes, Puerto Rican style	8000_Mixed	44	36	8	2	1.5	E
27315020	Beef, rice, and vegetables excluding carrots, broccoli, and dark-green leafy; no sauce	8000_Mixed	44	36	8	1	3.5	C
27363100	Jambalaya with meat and rice	8000_Mixed	44	36	8	2	3.5	C
58134120	Stuffed shells, cheese-filled, with tomato sauce, meatless	8000_Mixed	44	36	8	2	3	C
58146452	Pasta with cream sauce, seafood, and added vegetables, home recipe	8000_Mixed	44	35	9	2	3.5	C
27142100	Chicken or turkey fricassee	8000_Mixed	44	34	10	1	3.5	C

27212000	Beef and noodles, no sauce	8000_Mixed	44	34	10	1	3.5	C
27420200	Pork hash	8000_Mixed	44	34	10	1	3	D
27112000	Beef with gravy	8000_Mixed	44	33	11	1	4	A
27113000	Beef with cream or white sauce	8000_Mixed	44	33	11	1	4	B
27163010	Meat with gravy, NS as to type of meat,	8000_Mixed	44	33	11	1	4	A
58109100	Pizza, cheese, gluten-free thin crust	8000_Mixed	43	49	-6	4	3	D
58109140	Pizza, cheese and vegetables, gluten-free thin crust	8000_Mixed	43	49	-6	4	3	C
75340160	Vegetable and pasta combinations with cream or cheese sauce, broccoli, pasta, carrots, corn, zucchini, peppers, cauliflower, peas, etc., cooked	8000_Mixed	43	47	-4	4	3.5	C
14640012	Cheese sandwich, Cheddar cheese, on whole wheat bread, no spread	8000_Mixed	43	46	-3	3	2	D
28350220	Clam chowder, Manhattan	8000_Mixed	43	46	-3	4	3.5	C
14640022	Cheese sandwich, reduced fat Cheddar cheese, on wheat bread, no spread	8000_Mixed	43	44	-1	4	2.5	D
28113110	Salisbury steak, baked, with tomato sauce, vegetable, diet frozen meal	8000_Mixed	43	44	-1	4	4	B
28141050	Chicken patty parmigiana, breaded, with vegetable, diet frozen meal	8000_Mixed	43	44	-1	4	3.5	C
58403060	Chicken or turkey noodle soup, reduced sodium, canned or ready-to-serve	8000_Mixed	43	44	-1	4	3.5	B
28143030	Chicken and vegetable entree, oriental (diet frozen meal)	8000_Mixed	43	43	0	4	4	B
42302155	Peanut butter and jelly sandwich, with reduced fat peanut butter, reduced sugar jelly, on white bread	8000_Mixed	43	43	0	3	3.5	D
75142600	Cucumber salad made with cucumber and vinegar	8000_Mixed	43	43	0	2	3.5	B
27347210	Chicken or turkey,stuffing, and vegetables excluding carrots, broccoli, and dark green leafy; no sauce	8000_Mixed	43	42	1	3	3.5	C
75603000	Celery soup, cream of, NS as to made with milk or water	8000_Mixed	43	42	1	3	3	C
58156410	Rice with onions, Puerto Rican style	8000_Mixed	43	41	2	3	3	C
27260500	Vienna sausages stewed with potatoes, Puerto Rican style	8000_Mixed	43	40	3	3	3.5	C
58101540	Taco or tostada with fish	8000_Mixed	43	40	3	3	3.5	D
58146431	Pasta with cream sauce, poultry, and added vegetables, restaurant	8000_Mixed	43	39	4	2	3	D
58148111	Macaroni or pasta salad, made with light mayonnaise	8000_Mixed	43	39	4	2	3.5	B
75605010	Leek soup, cream of, prepared with milk	8000_Mixed	43	39	4	3	3	C
27243000	Chicken or turkey and rice, no sauce	8000_Mixed	43	37	6	1	3.5	C
58105000	Fajita with chicken and vegetables	8000_Mixed	43	37	6	3	3.5	C
58145190	Macaroni or noodles with cheese and chicken or turkey	8000_Mixed	43	37	6	3	3.5	B
58160200	Rice with vegetables (including carrots, broccoli, and/or dark-green leafy), no sauce, NS as to fat added in cooking	8000_Mixed	43	37	6	2	3	C
58160204	Rice with vegetables (including carrots, broccoli, and/or dark-green leafy), no sauce, fat added in cooking	8000_Mixed	43	37	6	1	3.5	C
58165030	Rice, white, with vegetables and gravy, NS as to fat	8000_Mixed	43	37	6	2	3.5	C
75142500	Cucumber salad, made with sour cream dressing	8000_Mixed	43	37	6	2	3.5	B
27146011	Chicken, shredded or pulled, with barbecue sauce	8000_Mixed	43	36	7	2	3.5	D
27212350	Beef stroganoff with noodles	8000_Mixed	43	36	7	2	3.5	C

58130010	Lasagna with meat and/or poultry	8000_Mixed	43	36	7	2	3.5	C
58160205	Rice with vegetables (excluding carrots, broccoli, and dark-green leafy), no sauce, NS as to fat added in cooking	8000_Mixed	43	36	7	1	3.5	C
58160209	Rice with vegetables (excluding carrots, broccoli, and dark-green leafy), no sauce, fat added in cooking	8000_Mixed	43	36	7	1	3.5	C
58403050	Chicken or turkey noodle soup, cream of, home recipe, canned, or ready-to-serve	8000_Mixed	43	36	7	2	3.5	C
27311220	Corned beef, potatoes, and vegetables excluding carrots, broccoli, and dark-green leafy; no sauce	8000_Mixed	43	35	8	2	4	A
58134610	Tortellini, meat-filled, with tomato sauce	8000_Mixed	43	35	8	2	3	C
58134640	Tortellini, cheese-filled, meatless, with vinaigrette dressing	8000_Mixed	43	35	8	2	2.5	D
58160560	Rice, white, with dark green vegetables, no added fat	8000_Mixed	43	35	8	1	3.5	B
58131530	Ravioli, cheese-filled, with meat sauce	8000_Mixed	43	34	9	2	3	C
27260050	Meatballs, with breading, NS as to type of meat, with gravy	8000_Mixed	43	33	10	2	3.5	C
27246400	Chicken or turkey souffle	8000_Mixed	43	31	12	1	3	D
58156110	Fried rice, Puerto Rican style	8000_Mixed	43	26	17	1	3	D
14640042	Cheese sandwich, reduced fat American cheese, on whole wheat bread, with mayonnaise	8000_Mixed	42	51	-9	4	2	D
58106236	Pizza, cheese, from school lunch, thick crust	8000_Mixed	42	48	-6	4	3.5	D
58106578	Pizza, with pepperoni, from school lunch, medium crust	8000_Mixed	42	47	-5	4	3.5	D
75651010	Minestrone soup, canned, prepared with water, or ready-to-serve	8000_Mixed	42	47	-5	4	3.5	C
14640072	Cheese sandwich, reduced fat Cheddar cheese, on whole wheat bread, with butter	8000_Mixed	42	44	-2	3	1.5	E
27540120	Chicken salad or chicken spread sandwich	8000_Mixed	42	42	0	3	3.5	C
58125120	Spinach quiche, meatless	8000_Mixed	42	41	1	3	1.5	D
28150910	Shrimp with potatoes, vegetable (frozen meal)	8000_Mixed	42	40	2	4	3.5	C
58109015	Pizza, cheese, whole wheat thin crust	8000_Mixed	42	40	2	3	2.5	D
27550300	Fish sandwich, NFS	8000_Mixed	42	39	3	3	2	D
41812400	Vegetarian pot pie	8000_Mixed	42	38	4	3	3.5	C
58104490	Chimichanga, NFS	8000_Mixed	42	38	4	3	3.5	C
58148112	Macaroni or pasta salad, made with mayonnaise-type salad dressing	8000_Mixed	42	38	4	2	3.5	B
75601000	Asparagus soup, cream of, NS as to made with milk or water	8000_Mixed	42	38	4	3	3.5	C
27213150	Chili con carne with beans and rice	8000_Mixed	42	37	5	3	3.5	C
27320120	Sausage, potatoes, and vegetables including carrots, broccoli, and/or dark-green leafy; gravy	8000_Mixed	42	37	5	3	3.5	C
27515050	Fajita-style beef sandwich with cheese, on pita bread, with lettuce and tomato	8000_Mixed	42	37	5	3	3.5	C
58146411	Pasta with cream sauce, meat, and added vegetables, restaurant	8000_Mixed	42	37	5	2	2.5	D
27243400	Chicken or turkey and rice with mushroom sauce	8000_Mixed	42	36	6	2	3.5	C
27317110	Beef, dumplings, and vegetables excluding carrots, broccoli, and dark-green leafy; gravy	8000_Mixed	42	36	6	2	3.5	C
28345020	Chicken or turkey soup, cream of, canned, reduced sodium, made with milk	8000_Mixed	42	36	6	3	3.5	B
58100005	Burrito, taco, or quesadilla with egg and potato	8000_Mixed	42	36	6	3	3	D

58105050	Fajita with meat and vegetables	8000_Mixed	42	36	6	3	3.5	C
58165020	Rice, white, with vegetables, cheese and/or cream based sauce, fat added	8000_Mixed	42	36	6	2	3.5	C
75604510	Cucumber soup, cream of, prepared with milk	8000_Mixed	42	36	6	1	3	C
27345440	Chicken or turkey, rice, and vegetables including carrots, broccoli, and/or dark-green leafy; cheese sauce	8000_Mixed	42	35	7	2	3.5	C
58165050	Rice, white, with vegetables and gravy, fat added	8000_Mixed	42	35	7	2	3.5	C
27235750	Veal and noodles with cream or white sauce	8000_Mixed	42	34	8	1	3.5	C
27242300	Chicken or turkey and noodles with cream or white sauce	8000_Mixed	42	34	8	1	3.5	C
27250820	Fish and rice with cream sauce	8000_Mixed	42	34	8	1	3.5	C
58131535	Ravioli, cheese-filled, with cream sauce	8000_Mixed	42	34	8	2	3	C
27115100	Steak teriyaki	8000_Mixed	42	32	10	1	3.5	D
58131100	Ravioli, NS as to filling, no sauce	8000_Mixed	42	30	12	1	3.5	C
58134650	Tortellini, meat-filled, no sauce	8000_Mixed	42	30	12	2	3.5	C
58151130	Sushi, with vegetables and seafood	8000_Mixed	42	29	13	1	3	C
14640028	Cheese sandwich, American cheese, on wheat bread, with mayonnaise	8000_Mixed	41	50	-9	4	1.5	E
58301110	Vegetable lasagna, frozen meal	8000_Mixed	41	46	-5	4	3.5	B
58146733	Pasta, whole grain, with cream sauce, poultry, and added vegetables, ready-to-heat	8000_Mixed	41	45	-4	4	3.5	C
28310120	Beef, broth, bouillon, or consomme, canned, low sodium	8000_Mixed	41	44	-3	4	3.5	A
28340170	Chicken broth, canned, low sodium	8000_Mixed	41	44	-3	4	3.5	A
28133210	Veal parmigiana with vegetable (diet frozen meal)	8000_Mixed	41	43	-2	4	4	A
14640010	Cheese sandwich, Cheddar cheese, on wheat bread, no spread	8000_Mixed	41	42	-1	3	2	D
58104270	Gordita, sope, or chalupa with beans and sour cream	8000_Mixed	41	39	2	3	3.5	C
27540110	Sliced chicken sandwich, with spread	8000_Mixed	41	38	3	3	4	B
58101240	Flauta with chicken	8000_Mixed	41	38	3	3	3.5	C
27345230	Chicken or turkey, rice, corn, and cheese, with gravy	8000_Mixed	41	37	4	3	3	C
27550425	Fish wrap sandwich	8000_Mixed	41	37	4	3	2	D
58100330	Burrito with beans, rice, and sour cream, meatless	8000_Mixed	41	37	4	3	3	D
58145120	Macaroni or noodles with cheese and tuna	8000_Mixed	41	37	4	3	3.5	C
75601010	Asparagus soup, cream of, prepared with milk	8000_Mixed	41	37	4	3	3.5	C
27515030	Steak and cheese sandwich, plain, on roll	8000_Mixed	41	36	5	3	3	D
58163610	Rice-vegetable medley	8000_Mixed	41	36	5	2	3	C
58421060	Sopa seca de arroz, home recipe, Mexican style	8000_Mixed	41	36	5	2	3	C
71801010	Potato soup, cream of, prepared with milk	8000_Mixed	41	36	5	3	3	C
75609010	Pea soup, prepared with milk	8000_Mixed	41	36	5	3	3.5	C
58108010	Calzone, with meat and cheese	8000_Mixed	41	35	6	2	2	D
58145135	Macaroni or noodles with cheese and meat	8000_Mixed	41	35	6	2	3.5	C
58160310	Rice with peas and carrots, NS as to fat added in cooking	8000_Mixed	41	35	6	1	3.5	C
58160314	Rice with peas and carrots, fat added in cooking	8000_Mixed	41	35	6	1	3.5	C

58165000	Rice, white, with vegetables, cheese and/or cream based sauce, NS as to fat	8000_Mixed	41	35	6	2	3.5	C
27315330	Beef, rice, and vegetables including carrots, broccoli, and/or dark-green leafy; cheese sauce	8000_Mixed	41	34	7	2	3.5	C
27330080	Lamb or mutton, rice, and vegetables including carrots, broccoli, and/or dark-green leafy; gravy	8000_Mixed	41	34	7	1	3.5	C
27540200	Fajita-style chicken sandwich with cheese, on pita bread, with lettuce and tomato	8000_Mixed	41	34	7	3	3.5	C
32204010	Scrambled egg sandwich	8000_Mixed	41	34	7	3	3.5	C
58101310	Taco or tostada with beef, lettuce, tomato and salsa	8000_Mixed	41	34	7	3	3.5	C
58135120	Chow fun noodles with vegetables, meatless	8000_Mixed	41	34	7	1	3.5	B
58163405	Spanish rice, from restaurant	8000_Mixed	41	34	7	1	3	C
71803010	Potato chowder	8000_Mixed	41	34	7	2	3	C
75440170	Vegetable sticks, breaded (including corn, carrots, and green beans)	8000_Mixed	41	34	7	2	3	C
27220310	Ham or pork and rice, no sauce	8000_Mixed	41	33	8	1	3.5	C
27345450	Chicken or turkey, rice, and vegetables excluding carrots, broccoli, and dark-green leafy; cheese sauce	8000_Mixed	41	33	8	2	3.5	C
41601120	Bean soup with vegetables, rice, and pork	8000_Mixed	41	33	8	2	3.5	B
58100000	Burrito, taco, or quesadilla with egg	8000_Mixed	41	33	8	3	3	D
58147310	Macaroni, creamed	8000_Mixed	41	33	8	1	3.5	B
58161300	White rice with tomato sauce	8000_Mixed	41	33	8	1	3	C
27360090	Paella, NFS	8000_Mixed	41	32	9	2	3	D
58404500	Matzo ball soup	8000_Mixed	41	32	9	3	3.5	C
27212050	Beef and macaroni with cheese sauce	8000_Mixed	41	31	10	1	3.5	C
27221100	Stewed pig's feet, Puerto Rican style	8000_Mixed	41	31	10	2	3.5	C
55703000	Cake made with glutinous rice and dried beans	8000_Mixed	41	31	10	1	3	D
58131330	Ravioli, meat-filled, with cream sauce	8000_Mixed	41	31	10	1	3	D
27116350	Stewed seasoned ground beef, Mexican style	8000_Mixed	41	30	11	1	3.5	C
27416200	Beef, ground, with egg and onion	8000_Mixed	41	28	13	1	2	D
14640030	Cheese sandwich, American cheese, on whole wheat bread, with mayonnaise	8000_Mixed	40	49	-9	4	1.5	E
27564420	Frankfurter or hot dog sandwich, meatless, plain, on bun	8000_Mixed	40	47	-7	4	4	B
58106234	Pizza, cheese, from school lunch, medium crust	8000_Mixed	40	46	-6	4	3.5	D
14640052	Cheese sandwich, American cheese, on wheat bread, with butter	8000_Mixed	40	45	-5	4	1.5	E
58106235	Pizza, cheese, from school lunch, thin crust	8000_Mixed	40	45	-5	4	3.5	D
14640064	Cheese sandwich, reduced fat American cheese, on wheat bread, with butter	8000_Mixed	40	44	-4	4	1.5	E
14640066	Cheese sandwich, reduced fat American cheese, on whole wheat bread, with butter	8000_Mixed	40	44	-4	4	1.5	E
14640165	Grilled cheese sandwich, reduced fat American cheese, on whole wheat bread	8000_Mixed	40	44	-4	4	1.5	E
58106580	Pizza with pepperoni, from school lunch, thick crust	8000_Mixed	40	44	-4	4	3.5	D
72302020	Broccoli soup, prepared with water, home recipe, canned, or ready-to-serve	8000_Mixed	40	44	-4	4	3	C

58110130	Egg roll, with beef and/or pork	8000_Mixed	40	42	-2	4	3	C
14640058	Cheese sandwich, Cheddar cheese, on wheat bread, with butter	8000_Mixed	40	41	-1	3	1.5	E
14640060	Cheese sandwich, Cheddar cheese, on whole wheat bread, with butter	8000_Mixed	40	41	-1	3	1.5	E
14640130	Grilled cheese sandwich, Cheddar cheese, on wheat bread	8000_Mixed	40	41	-1	3	1.5	E
14640135	Grilled cheese sandwich, Cheddar cheese, on whole wheat bread	8000_Mixed	40	41	-1	3	1.5	E
27347220	Chicken or turkey, stuffing, and vegetables including carrots, broccoli, and/or dark-green leafy; gravy	8000_Mixed	40	40	0	3	3.5	C
42302010	Peanut butter and jelly sandwich, NFS	8000_Mixed	40	40	0	3	2	D
42302015	Peanut butter and jelly sandwich, with regular peanut butter, regular jelly, on white bread	8000_Mixed	40	40	0	3	2	D
42303100	Peanut butter and jelly sandwich, frozen commercial product without crusts	8000_Mixed	40	40	0	3	2	D
58101200	Flauta, NFS	8000_Mixed	40	38	2	3	3.5	C
58109060	Pizza, cheese and vegetables, whole wheat thick crust	8000_Mixed	40	38	2	3	3.5	C
75607060	Mushroom soup, cream of, NS as to made with milk or water	8000_Mixed	40	38	2	3	3	C
58101730	Taco or tostada with meat and beans	8000_Mixed	40	37	3	3	3.5	C
58146391	Pasta with cream sauce and added vegetables, restaurant	8000_Mixed	40	37	3	2	2.5	D
27420450	Sausage and vegetables including carrots, broccoli, and/or dark-green leafy; no potatoes, tomato-based sauce	8000_Mixed	40	36	4	3	3	C
58100245	Burrito with chicken, beans, and sour cream	8000_Mixed	40	36	4	3	2	D
58101555	Soft taco with fish	8000_Mixed	40	36	4	3	3	D
58101745	Taco or tostada with meat, beans, and sour cream	8000_Mixed	40	36	4	3	3	C
58146421	Pasta with cream sauce and poultry, restaurant	8000_Mixed	40	36	4	2	2.5	D
14710100	Cheddar cheese soup, home recipe, canned or ready-to-serve	8000_Mixed	40	35	5	3	3	C
27510205	Cheeseburger, 1 small patty, with condiments, on white bun	8000_Mixed	40	35	5	3	3	D
27515070	Steak and cheese submarine sandwich, with fried peppers and onions, on roll	8000_Mixed	40	35	5	3	3.5	B
58100120	Burrito with meat and beans	8000_Mixed	40	35	5	3	3	D
58100140	Burrito with meat, beans, and sour cream	8000_Mixed	40	35	5	3	3	D
58100160	Burrito with meat, beans, and rice	8000_Mixed	40	35	5	3	3	D
58100260	Burrito with chicken, beans, rice, and sour cream	8000_Mixed	40	35	5	3	3	D
27500200	Wrap sandwich, filled with meat, poultry, or fish, vegetables, and cheese	8000_Mixed	40	34	6	3	3	D
27510251	Cheeseburger, 1 medium patty, with condiments, on white bun	8000_Mixed	40	34	6	3	3	D
27510720	Pizzaburger (hamburger, cheese, sauce) on whole bun	8000_Mixed	40	34	6	3	3.5	D
32202040	Egg, cheese, and beef on English Muffin	8000_Mixed	40	34	6	3	2	D
58100165	Burrito with meat, beans, rice, and sour cream	8000_Mixed	40	34	6	3	3	D
58101320	Taco or tostada with meat	8000_Mixed	40	34	6	3	2.5	D
58122310	Knish, potato	8000_Mixed	40	34	6	2	2.5	D
58200200	Wrap sandwich, filled with vegetables and rice	8000_Mixed	40	34	6	2	3.5	C
58122330	Knish, meat	8000_Mixed	40	33	7	2	2	D
58160320	Rice with tomatoes, NS as to fat added in cooking	8000_Mixed	40	33	7	2	3.5	C

58160324	Rice with tomatoes, fat added in cooking	8000_Mixed	40	33	7	2	3.5	C
58163400	Flavored rice and pasta mixture, reduced sodium	8000_Mixed	40	33	7	2	3.5	B
27330050	Lamb or mutton, rice, and vegetables excluding carrots, broccoli, and dark-green leafy; gravy	8000_Mixed	40	32	8	1	3.5	C
58100230	Burrito with chicken and cheese	8000_Mixed	40	32	8	3	2	D
58105100	Pupusa, cheese-filled	8000_Mixed	40	32	8	1	2.5	D
58120120	Crepe, filled with meat, poultry, or seafood, no sauce	8000_Mixed	40	32	8	2	3	D
58133110	Manicotti, cheese-filled, no sauce	8000_Mixed	40	32	8	2	3	D
58160300	Rice with peas, NS as to fat added in cooking	8000_Mixed	40	32	8	1	3.5	C
27213120	Porcupine balls with tomato-based sauce	8000_Mixed	40	31	9	1	3.5	C
27320090	Sausage, noodles, and vegetables including carrots, broccoli, and/or dark-green leafy; tomato-based sauce	8000_Mixed	40	31	9	2	3.5	C
58103110	Tamale with meat and/or poultry	8000_Mixed	40	31	9	1	3	D
58134110	Stuffed shells, cheese-filled, no sauce	8000_Mixed	40	31	9	2	3	D
58146442	Pasta with cream sauce and seafood, home recipe	8000_Mixed	40	31	9	2	3	C
58160620	Rice, white, with dark green vegetables and tomatoes and/or tomato-based sauce, no added fat	8000_Mixed	40	31	9	1	3.5	C
27130010	Lamb or mutton with gravy	8000_Mixed	40	30	10	1	3.5	D
27233000	Lamb or mutton and noodles with gravy	8000_Mixed	40	30	10	1	3.5	C
27243700	Chicken in cheese sauce with Spanish rice	8000_Mixed	40	30	10	1	3.5	C
58100240	Burrito with chicken, NFS	8000_Mixed	40	30	10	3	4	C
14640018	Cheese sandwich, reduced fat American cheese, on whole wheat bread, no spread	8000_Mixed	39	43	-4	4	2	D
14640160	Grilled cheese sandwich, reduced fat American cheese, on wheat bread	8000_Mixed	39	43	-4	4	1.5	E
58146693	Pasta, whole grain, with cream sauce, and added vegetables, ready-to-heat	8000_Mixed	39	43	-4	4	3	C
58146713	Pasta, whole grain, with cream sauce, meat, and added vegetables, ready-to-heat	8000_Mixed	39	43	-4	4	3.5	C
75654020	Vegetarian vegetable soup, undiluted	8000_Mixed	39	43	-4	4	3	C
14640054	Cheese sandwich, American cheese, on whole wheat bread, with butter	8000_Mixed	39	42	-3	4	1.5	E
58106634	Pizza, with meat other than pepperoni, from school lunch, medium crust	8000_Mixed	39	42	-3	4	3.5	D
58106635	Pizza, with meat other than pepperoni, from school lunch, thin crust	8000_Mixed	39	42	-3	4	3.5	D
58127110	Vegetables in pastry	8000_Mixed	39	42	-3	4	3.5	C
75649040	Vegetable soup, reduced sodium, canned, ready to serve	8000_Mixed	39	42	-3	4	3.5	A
28141650	Chicken and vegetables au gratin with rice, diet frozen entree	8000_Mixed	39	41	-2	4	3.5	C
58304220	Rigatoni with meat sauce and cheese, diet frozen meal	8000_Mixed	39	41	-2	4	3.5	B
28355110	Clam chowder, New England, NS as to prepared with water or milk	8000_Mixed	39	40	-1	4	3.5	C
42302055	Peanut butter and jelly sandwich, with reduced fat peanut butter, regular jelly, on white bread	8000_Mixed	39	40	-1	3	2.5	D
71801040	Potato soup, instant, made from dry mix	8000_Mixed	39	40	-1	4	3.5	A
28110270	Sirloin beef, with gravy, potatoes, vegetable, frozen meal	8000_Mixed	39	39	0	4	4	B

28141300	Chicken with rice and vegetable, reduced fat and sodium (diet frozen meal)	8000_Mixed	39	39	0	4	4	A
75652020	Vegetable beef soup, canned, undiluted	8000_Mixed	39	39	0	4	3	C
27540111	Sliced chicken sandwich, with cheese and spread	8000_Mixed	39	38	1	3	3.5	D
58104260	Gordita, sope, or chalupa with beans	8000_Mixed	39	38	1	3	3.5	C
58100300	Burrito with beans and rice, meatless	8000_Mixed	39	37	2	3	3	D
58101230	Flauta with beef	8000_Mixed	39	37	2	3	3.5	C
58148550	Macaroni or pasta salad with meat	8000_Mixed	39	37	2	2	3.5	C
75607010	Mushroom soup, cream of, prepared with milk	8000_Mixed	39	37	2	3	3	C
58101725	Taco or tostada with beans and sour cream	8000_Mixed	39	36	3	3	3.5	C
58100210	Burrito with chicken and beans	8000_Mixed	39	35	4	3	2.5	B
58100255	Burrito with chicken, beans, and rice	8000_Mixed	39	35	4	3	3.5	D
58101750	Taco or tostada with chicken, beans, and sour cream	8000_Mixed	39	35	4	3	3	C
58101520	Taco or tostada with chicken	8000_Mixed	39	34	5	3	3	D
58101525	Taco or tostada with chicken and sour cream	8000_Mixed	39	34	5	3	3	D
58101630	Soft taco with meat, beans, and sour cream	8000_Mixed	39	34	5	3	3	C
58108000	Calzone, with cheese, meatless	8000_Mixed	39	34	5	2	1.5	E
58146401	Pasta with cream sauce and meat, restaurant	8000_Mixed	39	34	5	2	2.5	D
27320130	Sausage, potatoes, and vegetables excluding carrots, broccoli, and dark-green leafy; gravy	8000_Mixed	39	33	6	3	3.5	C
28154010	Shrimp and vegetables in sauce with noodles, diet frozen meal	8000_Mixed	39	33	6	4	3.5	C
58100235	Burrito with chicken and sour cream	8000_Mixed	39	33	6	3	2	D
58101325	Taco or tostada with meat and sour cream	8000_Mixed	39	33	6	3	2	D
58165060	Rice, white, with vegetables, soy-based sauce, NS as to fat	8000_Mixed	39	33	6	1	3	C
58165080	Rice, white, with vegetables, soy-based sauce, fat added	8000_Mixed	39	33	6	1	3	C
27120030	Ham or pork with barbecue sauce	8000_Mixed	39	32	7	2	3.5	D
27213400	Beef and rice with mushroom sauce	8000_Mixed	39	32	7	2	3.5	C
27315340	Beef, rice, and vegetables excluding carrots, broccoli, and dark-green leafy; cheese sauce	8000_Mixed	39	32	7	2	3.5	C
27515010	Steak sandwich, plain, on roll	8000_Mixed	39	32	7	2	4	B
27135150	Veal cordon bleu	8000_Mixed	39	31	8	2	2	D
27213000	Beef and rice, no sauce	8000_Mixed	39	31	8	1	3.5	B
27218310	Stewed corned beef, Puerto Rican style	8000_Mixed	39	31	8	2	3.5	C
58104810	Taquitoes	8000_Mixed	39	31	8	2	3.5	D
58160290	Rice with corn, NS as to fat added in cooking	8000_Mixed	39	31	8	1	3	C
58160294	Rice with corn, fat added in cooking	8000_Mixed	39	31	8	1	3	C
58160470	Rice, white, with carrots, no added fat	8000_Mixed	39	31	8	1	3.5	C
58161710	Rice croquette	8000_Mixed	39	31	8	2	3	C
58404100	Rice and potato soup, Puerto Rican style	8000_Mixed	39	31	8	1	3	C
27212300	Beef and noodles with cream or white sauce	8000_Mixed	39	30	9	1	3.5	C
27230010	Lamb or mutton loaf	8000_Mixed	39	30	9	1	3	D

58160590	Rice, white, with carrots and tomatoes and/or tomato-based sauce, no added fat	8000_Mixed	39	30	9	1	3.5	C
58160650	Rice, white, with carrots and dark green vegetables, no added fat	8000_Mixed	39	30	9	1	3.5	B
58105110	Pupusa, meat-filled	8000_Mixed	39	29	10	1	3.5	C
58160302	Rice with peas, fat not added in cooking	8000_Mixed	39	29	10	1	3.5	C
27113300	Swedish meatballs with cream or white sauce	8000_Mixed	39	28	11	1	3	D
27118140	Stuffed pot roast, Puerto Rican style, NFS	8000_Mixed	39	28	11	2	1.5	D
27160100	Meatballs, NS as to type of meat, with sauce	8000_Mixed	39	26	13	1	2	D
58131310	Ravioli, meat-filled, no sauce	8000_Mixed	39	26	13	1	3	D
58150510	Rice, fried, with shrimp	8000_Mixed	39	25	14	1	3.5	C
14640016	Cheese sandwich, reduced fat American cheese, on wheat bread, no spread	8000_Mixed	38	42	-4	4	2	D
14640110	Grilled cheese sandwich, American cheese, on wheat bread	8000_Mixed	38	42	-4	4	1.5	E
58127150	Vegetables and cheese in pastry	8000_Mixed	38	42	-4	4	3	D
58110170	Egg roll, with chicken or turkey	8000_Mixed	38	41	-3	4	3.5	C
58301020	Lasagna with cheese and sauce, diet frozen meal	8000_Mixed	38	41	-3	4	3.5	C
58106636	Pizza, with meat other than pepperoni, from school lunch, thick crust	8000_Mixed	38	40	-2	4	3.5	D
27341000	Chicken or turkey, potatoes, corn, and cheese, with gravy	8000_Mixed	38	38	0	4	3.5	C
28143010	Chicken and vegetable entree with rice, Oriental (frozen meal)	8000_Mixed	38	38	0	4	4	B
58101720	Taco or tostada with beans	8000_Mixed	38	38	0	4	3.5	C
27111430	Chili con carne, NS as to beans, with cheese	8000_Mixed	38	37	1	4	3.5	C
27111440	Chili con carne with beans and cheese	8000_Mixed	38	37	1	4	3.5	C
27347230	Chicken or turkey, stuffing, and vegetables excluding carrots, broccoli, and dark-green leafy; gravy	8000_Mixed	38	37	1	3	3.5	C
58100310	Burrito with beans, meatless	8000_Mixed	38	37	1	3	3	C
28150510	Fish in lemon-butter sauce with starch item, vegetable, frozen meal	8000_Mixed	38	36	2	4	3	C
58101735	Taco or tostada with chicken and beans	8000_Mixed	38	36	2	3	3.5	C
58104160	Nachos with chili	8000_Mixed	38	36	2	3	2.5	D
58109020	Pizza, cheese, whole wheat thick crust	8000_Mixed	38	36	2	3	3.5	D
58128220	Dressing with chicken or turkey and vegetables	8000_Mixed	38	36	2	3	3	C
58304400	Linguini with vegetables and seafood in white wine sauce, diet frozen meal	8000_Mixed	38	36	2	4	3.5	C
58104290	Gordita, sope, or chalupa with meat	8000_Mixed	38	35	3	3	3	D
27515040	Steak and cheese submarine sandwich, plain, on roll	8000_Mixed	38	34	4	3	3.5	D
58101635	Soft taco with chicken, beans, and sour cream	8000_Mixed	38	34	4	3	3.5	C
58104280	Gordita, sope, or chalupa with meat and sour cream	8000_Mixed	38	34	4	3	3	D
58104320	Gordita, sope, or chalupa with chicken and sour cream	8000_Mixed	38	34	4	3	3	D
58146381	Pasta with cream sauce, restaurant	8000_Mixed	38	34	4	2	2	D
27510341	Bacon cheeseburger, 1 medium patty, with condiments, on white bun	8000_Mixed	38	33	5	3	2	D
27513050	Roast beef sandwich with cheese	8000_Mixed	38	33	5	2	1.5	D
27550405	Fish sandwich, fried, on white bun, with cheese	8000_Mixed	38	33	5	3	3	D

58104600	Chimichanga with beef and rice	8000_Mixed	38	33	5	3	3.5	C
27550400	Fish sandwich, fried, on white bun	8000_Mixed	38	32	6	3	3.5	C
58100135	Burrito with meat and sour cream	8000_Mixed	38	32	6	3	2	D
58100150	Burrito with beef and potato, no beans	8000_Mixed	38	32	6	2	3.5	C
58109210	Breakfast pizza with egg	8000_Mixed	38	32	6	3	2	D
27500300	Wrap sandwich, NFS	8000_Mixed	38	31	7	3	3.5	C
27510641	Hamburger, 1 medium patty, with condiments, on white bun	8000_Mixed	38	31	7	2	3.5	D
27550000	Fish sandwich, fried, from fast food	8000_Mixed	38	31	7	3	3.5	C
58122320	Knish, cheese	8000_Mixed	38	31	7	2	3	D
58145170	Macaroni or noodles with cheese and egg	8000_Mixed	38	31	7	3	3	C
27116200	Beef with barbecue sauce	8000_Mixed	38	30	8	2	3.5	C
27320080	Sausage, noodles, and vegetables excluding carrots, broccoli, and dark-green leafy; tomato-based sauce	8000_Mixed	38	30	8	2	3.5	C
58100020	Burrito, taco, or quesadilla with egg, beans, and breakfast meat	8000_Mixed	38	30	8	3	3	D
58150520	Dukboki or Tteokbokki, Korean	8000_Mixed	38	30	8	2	3.5	B
58165010	Rice, white, with vegetables, cheese and/or cream based sauce, no added fat	8000_Mixed	38	30	8	2	3.5	C
27213420	Porcupine balls with mushroom sauce	8000_Mixed	38	29	9	1	3	C
58146130	Pasta with carbonara sauce	8000_Mixed	38	29	9	2	3	C
58155110	Rice with chicken, Puerto Rican style	8000_Mixed	38	29	9	1	2	D
58160530	Rice, white, with tomatoes and/or tomato-based sauce, no added fat	8000_Mixed	38	29	9	1	3.5	C
58160680	Rice, white, with carrots, dark green vegetables, and tomatoes and/or tomato-based sauce, no added fat	8000_Mixed	38	29	9	1	3.5	C
58163420	Spanish rice, no added fat	8000_Mixed	38	29	9	1	3.5	C
58103310	Tamale casserole with meat	8000_Mixed	38	28	10	1	3.5	C
58160202	Rice with vegetables (including carrots, broccoli, and/or dark-green leafy), no sauce, fat not added in cooking	8000_Mixed	38	28	10	1	3.5	C
27540296	Buffalo chicken submarine sandwich with cheese	8000_Mixed	37	42	-5	4	3.5	C
14640115	Grilled cheese sandwich, American cheese, on whole wheat bread	8000_Mixed	37	41	-4	4	1.5	E
28145000	Turkey dinner, NFS, frozen meal	8000_Mixed	37	41	-4	4	3.5	C
28145210	Turkey with gravy, dressing, potatoes, vegetable, frozen meal	8000_Mixed	37	41	-4	4	3.5	C
58146303	Pasta with tomato-based sauce, and added vegetables, ready-to-heat	8000_Mixed	37	41	-4	4	3.5	C
58146723	Pasta, whole grain, with cream sauce and poultry, ready-to-heat	8000_Mixed	37	41	-4	4	3	C
14640004	Cheese sandwich, American cheese, on wheat bread, no spread	8000_Mixed	37	40	-3	4	2	D
14640006	Cheese sandwich, American cheese, on whole wheat bread, no spread	8000_Mixed	37	40	-3	4	2	D
41601010	Bean soup, NFS	8000_Mixed	37	40	-3	4	3.5	B
41601020	Bean with bacon or ham soup, canned or ready-to-serve	8000_Mixed	37	40	-3	4	3.5	B
58301050	Lasagna with cheese and meat sauce, diet frozen meal	8000_Mixed	37	40	-3	4	4	B
28140730	Chicken patty, breaded, with tomato sauce and cheese, fettuccine alfredo, vegetable, frozen meal	8000_Mixed	37	39	-2	4	4	B

58305250	Pasta with vegetable and cheese sauce, diet frozen meal	8000_Mixed	37	39	-2	4	3.5	B
28143110	Chicken cacciatore with noodles, diet frozen meal	8000_Mixed	37	38	-1	4	3.5	B
28110250	Sirloin tips, with gravy, potatoes, vegetable, frozen meal	8000_Mixed	37	37	0	4	4	B
28160710	Stuffed cabbage, with meat and tomato sauce, diet frozen meal	8000_Mixed	37	37	0	4	3.5	A
28340310	Chicken or turkey gumbo soup, home recipe, canned or ready-to-serve	8000_Mixed	37	36	1	4	3	C
58101610	Soft taco with beans	8000_Mixed	37	36	1	4	3.5	C
75405010	Beets with Harvard sauce	8000_Mixed	37	36	1	3	3.5	C
75651020	Vegetable beef soup, canned, prepared with water, or ready-to-serve	8000_Mixed	37	36	1	4	3.5	C
75651030	Vegetable beef noodle soup, prepared with water	8000_Mixed	37	36	1	4	3.5	C
75651080	Vegetable beef soup with rice, canned, prepared with water or ready-to-serve	8000_Mixed	37	36	1	4	3.5	C
58101615	Soft taco with beans and sour cream	8000_Mixed	37	35	2	3	3	C
58104340	Gordita, sope, or chalupa with chicken	8000_Mixed	37	35	2	3	3	D
75608100	Onion soup, French	8000_Mixed	37	35	2	3	3	C
58101620	Soft taco with meat and beans	8000_Mixed	37	34	3	3	3	D
58107212	White pizza, cheese, with vegetables, thin crust	8000_Mixed	37	34	3	3	3	D
27550100	Fish sandwich, fried, from fast food, with cheese	8000_Mixed	37	33	4	3	2	D
58100200	Burrito with chicken	8000_Mixed	37	33	4	3	2	D
58101345	Soft taco with meat	8000_Mixed	37	33	4	3	2.5	D
58107220	White pizza, thin crust	8000_Mixed	37	33	4	2	1.5	D
27510585	Hamburger, 1 small patty, with condiments, on white bun	8000_Mixed	37	32	5	3	3.5	D
27317010	Beef pot pie	8000_Mixed	37	31	6	2	3	C
58100100	Burrito with meat	8000_Mixed	37	31	6	3	2	D
58101742	Soft taco with egg, potato, cheese, tomato and/or salsa	8000_Mixed	37	31	6	3	3	C
58107030	Pizza, no cheese, NS as to type of crust	8000_Mixed	37	31	6	1	3.5	C
58107060	Pizza, no cheese, regular crust	8000_Mixed	37	31	6	1	3.5	C
27515000	Steak submarine sandwich with lettuce and tomato	8000_Mixed	37	30	7	3	4	A
58109000	Italian pie, meatless	8000_Mixed	37	30	7	2	2	D
58146432	Pasta with cream sauce, poultry, and added vegetables, home recipe	8000_Mixed	37	30	7	2	3.5	C
27213200	Beef and rice with gravy	8000_Mixed	37	29	8	2	3.5	C
58105105	Pupusa, bean-filled	8000_Mixed	37	29	8	2	3.5	C
58116130	Empanada, Mexican turnover, filled with chicken and vegetables	8000_Mixed	37	29	8	2	3	D
58116120	Empanada, Mexican turnover, filled with meat and vegetables	8000_Mixed	37	28	9	1	2.5	D
58160500	Rice, white, with peas and carrots, no added fat	8000_Mixed	37	28	9	1	3.5	B
27520140	Bacon and egg sandwich	8000_Mixed	37	27	10	3	3	D
58157320	Congee, with vegetables	8000_Mixed	37	27	10	1	3.5	B
58160207	Rice with vegetables (excluding carrots, broccoli, and dark-green leafy), no sauce, fat not added in cooking	8000_Mixed	37	27	10	1	3.5	C
58131510	Ravioli, cheese-filled, no sauce	8000_Mixed	37	26	11	2	2.5	D

58146223	Pasta with tomato-based sauce, ready-to-heat	8000_Mixed	36	41	-5	4	3.5	C
28160650	Stuffed green pepper, frozen meal	8000_Mixed	36	40	-4	4	3.5	C
58110110	Egg roll, meatless	8000_Mixed	36	40	-4	4	3	C
58110120	Egg roll, with shrimp	8000_Mixed	36	40	-4	4	3	C
58130310	Lasagna, meatless	8000_Mixed	36	40	-4	4	3.5	C
58146353	Pasta with tomato-based sauce, poultry, and added vegetables, ready-to-heat	8000_Mixed	36	40	-4	4	3.5	B
58106820	Pizza with beans and vegetables, thin crust	8000_Mixed	36	39	-3	4	3.5	C
58146343	Pasta with tomato-based sauce and poultry, ready-to-heat	8000_Mixed	36	39	-3	4	3.5	C
28110510	Beef, sliced, with gravy, potatoes, vegetable, frozen meal	8000_Mixed	36	38	-2	4	3.5	C
75414020	Mushrooms, stuffed	8000_Mixed	36	38	-2	4	4	C
28140720	Chicken patty, or nuggets, boneless, breaded, potatoes, vegetable, frozen meal	8000_Mixed	36	37	-1	4	3.5	B
58111120	Wonton, fried, meatless	8000_Mixed	36	37	-1	4	3	C
58307010	Beef and pork cannelloni (diet frozen meal)	8000_Mixed	36	37	-1	4	3.5	B
28110150	Beef with vegetable, diet frozen meal	8000_Mixed	36	36	0	4	3.5	B
27220050	Ham or pork with stuffing	8000_Mixed	36	35	1	3	3.5	C
28143050	Chicken chow mein with rice, reduced fat and sodium (diet frozen meal)	8000_Mixed	36	35	1	4	4	B
28160660	Stuffed green pepper (diet frozen meal)	8000_Mixed	36	35	1	4	3.5	C
27570310	Hors d'oeuvres, with spread	8000_Mixed	36	34	2	4	3.5	C
58101625	Soft taco with chicken and beans	8000_Mixed	36	34	2	3	3.5	C
74203010	Tomatoes, scalloped	8000_Mixed	36	33	3	3	4	A
58101450	Soft taco with chicken	8000_Mixed	36	32	4	3	3	D
27420460	Sausage and vegetables, excluding carrots, broccoli, and dark-green leafy; no potatoes, tomato-based sauce	8000_Mixed	36	31	5	3	3	C
58101350	Soft taco with meat and sour cream	8000_Mixed	36	31	5	3	3	D
58101460	Soft taco with chicken and sour cream	8000_Mixed	36	31	5	3	3	D
58145110	Macaroni or noodles with cheese	8000_Mixed	36	31	5	2	3	D
58106520	Pizza with meat, thin crust	8000_Mixed	36	30	6	2	2	D
27160010	Meat with barbecue sauce, NS as to type of meat	8000_Mixed	36	28	8	2	3.5	D
58146412	Pasta with cream sauce, meat, and added vegetables, home recipe	8000_Mixed	36	28	8	2	3.5	C
58164110	Rice with raisins	8000_Mixed	36	28	8	1	3	C
58151120	Sushi, with vegetables, no seafood (no fish or shellfish)	8000_Mixed	36	27	9	1	3.5	C
58151140	Sushi, with vegetables, rolled in seaweed	8000_Mixed	36	27	9	1	3.5	C
58126000	Turnover filled with ground beef and cabbage	8000_Mixed	36	26	10	1	3.5	C
58134660	Tortellini, cheese-filled, with cream sauce	8000_Mixed	36	26	10	2	2.5	D
58160440	Rice, white, with peas, no added fat	8000_Mixed	36	26	10	1	3.5	B
27213600	Beef and rice with cheese sauce	8000_Mixed	36	25	11	1	3.5	C
27540295	Buffalo chicken submarine sandwich	8000_Mixed	35	40	-5	4	3.5	B
58104120	Nachos with cheese	8000_Mixed	35	40	-5	4	3	C

28141250	Chicken with rice and vegetable, diet frozen meal	8000_Mixed	35	38	-3	4	4	B
58306050	Cheese enchilada with beans and rice (frozen meal)	8000_Mixed	35	37	-2	4	3	C
28143080	Chicken with noodles and cheese sauce, diet frozen meal	8000_Mixed	35	36	-1	4	4	A
28340180	Chicken or turkey broth, less or reduced sodium, canned or ready-to-serve	8000_Mixed	35	36	-1	4	3.5	B
58104520	Chimichanga, meatless	8000_Mixed	35	36	-1	3	3.5	C
58302030	Macaroni with veal, cheese, and sauce (diet frozen meal)	8000_Mixed	35	36	-1	4	4	B
58302050	Beef and noodles with meat sauce and cheese, diet frozen meal	8000_Mixed	35	36	-1	4	3.5	C
28340630	Chicken or turkey vegetable soup with rice, stew type, chunky style	8000_Mixed	35	35	0	4	3.5	B
27111407	Chili con carne with beans, canned	8000_Mixed	35	34	1	4	3.5	C
58104500	Chimichanga with meat	8000_Mixed	35	34	1	3	2.5	D
58104540	Chimichanga, meatless, with sour cream	8000_Mixed	35	34	1	3	3	C
58104550	Chimichanga with chicken and sour cream	8000_Mixed	35	33	2	3	2.5	D
32202200	Egg and cheese on biscuit	8000_Mixed	35	32	3	3	1.5	E
58100360	Chilaquiles, tortilla casserole with salsa, cheese, and egg	8000_Mixed	35	32	3	3	2.5	D
27220080	Ham croquette	8000_Mixed	35	31	4	3	1.5	E
71801000	Potato soup, NS as to made with milk or water	8000_Mixed	35	31	4	3	3	C
58107230	White pizza, thick crust	8000_Mixed	35	30	5	2	2	D
14710200	Beer cheese soup, made with milk	8000_Mixed	35	29	6	3	2	D
27510331	Bacon cheeseburger, 1 medium patty, plain, on white bun	8000_Mixed	35	29	6	3	2	D
27520520	Pork sandwich	8000_Mixed	35	29	6	3	4	B
58164500	Rice, white, with cheese and/or cream based sauce, NS as to fat	8000_Mixed	35	29	6	2	3	C
27540130	Chicken barbecue sandwich	8000_Mixed	35	28	7	3	3.5	C
32202085	Egg, cheese and bacon on bagel	8000_Mixed	35	28	7	3	2	D
58106510	Pizza with meat, NS as to type of crust	8000_Mixed	35	28	7	2	2	D
58116115	Empanada, Mexican turnover, filled with cheese and vegetables	8000_Mixed	35	28	7	2	2	D
58164520	Rice, white, with cheese and/or cream based sauce, fat added	8000_Mixed	35	28	7	2	3	C
58161110	Rice casserole with cheese	8000_Mixed	35	27	8	2	3	D
27220210	Ham and noodles, no sauce	8000_Mixed	35	26	9	2	3	C
58101800	Ground beef with tomato sauce and taco seasonings on a cornbread crust	8000_Mixed	35	26	9	3	3	D
58160312	Rice with peas and carrots, fat not added in cooking	8000_Mixed	35	26	9	1	3.5	C
58145136	Macaroni or noodles with cheese and meat, prepared from Hamburger Helper mix	8000_Mixed	35	25	10	2	3.5	C
75142000	Cucumber and vegetable namasu	8000_Mixed	34	39	-5	2	3.5	B
58109130	Pizza, with meat, gluten-free thick crust	8000_Mixed	34	37	-3	4	2.5	D
58146683	Pasta, whole grain, with cream sauce, ready-to-heat	8000_Mixed	34	37	-3	4	3	D
75651060	Vegetable chicken or turkey soup, canned, undiluted	8000_Mixed	34	36	-2	4	3	C
28141060	Chicken patty with vegetable (diet frozen meal)	8000_Mixed	34	35	-1	4	3.5	C
58111110	Wonton, fried, filled with meat, poultry, or seafood,	8000_Mixed	34	35	-1	4	3	C
58111130	Wonton, fried, filled with meat, poultry, or seafood, and vegetable	8000_Mixed	34	35	-1	4	3	C

73211110	Sweet potato and pumpkin casserole, Puerto Rican style	8000_Mixed	34	35	-1	2	3.5	C
75651110	Vegetable chicken rice soup, canned, prepared with water or ready-to-serve	8000_Mixed	34	35	-1	4	3.5	B
58104530	Chimichanga with chicken	8000_Mixed	34	34	0	3	2.5	D
28133220	Veal with peppers in sauce, rice (diet frozen meal)	8000_Mixed	34	33	1	4	4	B
75418220	Creamed christophine, Puerto Rican style	8000_Mixed	34	32	2	2	3	C
28315120	Beef vegetable soup with noodles, stew type, chunky style	8000_Mixed	34	31	3	4	3.5	B
58200250	Wrap sandwich, filled with vegetables	8000_Mixed	34	30	4	3	3.5	C
58100015	Burrito, taco, or quesadilla with egg, potato, and breakfast meat	8000_Mixed	34	27	7	3	3	D
58101741	Soft taco with egg, potato, and cheese	8000_Mixed	34	27	7	3	3	D
58126140	Turnover, meat- and bean-filled, no gravy	8000_Mixed	34	27	7	2	2	D
58126180	Turnover, meat-, potato-, and vegetable-filled, no gravy	8000_Mixed	34	27	7	2	3	D
58121620	Dumpling, vegetable	8000_Mixed	34	26	8	1	3	C
58126310	Turnover, chicken, with gravy	8000_Mixed	34	26	8	2	3	D
58146422	Pasta with cream sauce and poultry, home recipe	8000_Mixed	34	26	8	2	3.5	C
28310330	Pho	8000_Mixed	34	25	9	1	3.5	C
32201000	Fried egg sandwich	8000_Mixed	34	24	10	3	3.5	C
58160322	Rice with tomatoes, fat not added in cooking	8000_Mixed	34	24	10	1	3.5	C
58160410	Rice, white, with corn, no added fat	8000_Mixed	34	24	10	1	3.5	B
58160710	Rice, white, with other vegetables, no added fat	8000_Mixed	34	24	10	1	3.5	C
58103250	Tamale, plain, meatless, no sauce, Mexican style	8000_Mixed	34	23	11	1	3.5	C
58301150	Zucchini lasagna, diet frozen meal	8000_Mixed	33	37	-4	4	3.5	B
58303100	Rice, with broccoli, cheese sauce, frozen side dish	8000_Mixed	33	36	-3	4	3.5	C
58106830	Pizza with beans and vegetables, thick crust	8000_Mixed	33	35	-2	4	3	D
58109120	Pizza, with meat, gluten-free thin crust	8000_Mixed	33	35	-2	4	2	D
27111420	Chili con carne without beans	8000_Mixed	33	34	-1	4	3	C
28143130	Chicken and vegetable entree with noodles, frozen meal	8000_Mixed	33	34	-1	4	4	C
28143190	Chicken in mushroom sauce, white and wild rice, vegetable, frozen meal	8000_Mixed	33	34	-1	4	3.5	C
75651050	Vegetable chicken or turkey soup, prepared with water or ready-to-serve	8000_Mixed	33	34	-1	4	3	C
27510246	Cheeseburger, on wheat bun, 1 large patty	8000_Mixed	33	33	0	4	2	D
27564390	Frankfurter or hot dog sandwich, fat free, plain, on whole wheat bread	8000_Mixed	33	33	0	4	3.5	C
28145100	Turkey with gravy, dressing, vegetable and fruit, diet frozen meal	8000_Mixed	33	33	0	4	4	B
41602090	Split pea and ham soup, canned, reduced sodium, prepared with water or ready-to-serve	8000_Mixed	33	33	0	4	3.5	B
58100145	Burrito with meat, beans, and sour cream, from fast food	8000_Mixed	33	33	0	4	3.5	C
58306200	Chicken fajitas, diet frozen meal	8000_Mixed	33	32	1	4	4	A
58104510	Chimichanga with beef, cheese, lettuce and tomato	8000_Mixed	33	30	3	3	3	D
58164210	Rice dessert or salad with fruit	8000_Mixed	33	29	4	3	3	C
58124500	Pastry, filled with potatoes and peas, fried	8000_Mixed	33	28	5	2	2	D

27513070	Roast beef submarine sandwich, on roll, au jus	8000_Mixed	33	27	6	2	3.5	C
58161200	Rice, cooked with coconut milk	8000_Mixed	33	27	6	2	1.5	D
27113200	Creamed chipped or dried beef	8000_Mixed	33	26	7	3	3	C
27513020	Roast beef sandwich, with gravy	8000_Mixed	33	26	7	2	3	D
58126170	Turnover filled with meat and vegetable, no potatoes, no gravy	8000_Mixed	33	26	7	2	3	D
58146392	Pasta with cream sauce and added vegetables, from home recipe	8000_Mixed	33	26	7	2	3	C
58155810	Stewed rice, Puerto Rican style	8000_Mixed	33	26	7	1	2.5	D
58164530	Rice, white, with gravy, NS as to fat	8000_Mixed	33	26	7	2	3	C
27213300	Beef and rice with cream sauce	8000_Mixed	33	25	8	1	3.5	C
58100010	Burrito, taco, or quesadilla with egg and breakfast meat	8000_Mixed	33	25	8	3	3	D
58164550	Rice, white, with gravy, fat added	8000_Mixed	33	25	8	2	3	C
58165040	Rice, white, with vegetables and gravy, no added fat	8000_Mixed	33	24	9	1	3.5	C
58103120	Tamale with meat	8000_Mixed	33	23	10	1	3	D
58103130	Tamale with chicken	8000_Mixed	33	23	10	1	3	D
28320130	Ham, rice, and potato soup, Puerto Rican style	8000_Mixed	33	22	11	2	3.5	C
28340700	Bird's nest soup	8000_Mixed	33	20	13	2	3	C
28316020	Beef and mushroom soup, canned, low sodium	8000_Mixed	32	36	-4	4	3.5	A
58304200	Ravioli, cheese-filled, with tomato sauce, diet frozen meal	8000_Mixed	32	36	-4	4	3.5	C
14640044	Cheese sandwich, reduced fat Cheddar cheese, on white bread, with mayonnaise	8000_Mixed	32	35	-3	4	2	D
75649020	Vegetable soup, canned, undiluted	8000_Mixed	32	35	-3	4	3.5	C
27510196	Cheeseburger, on wheat bun, 1 small patty	8000_Mixed	32	34	-2	4	2	D
58104180	Nachos with meat, cheese, and sour cream	8000_Mixed	32	34	-2	4	3.5	C
27510242	Cheeseburger, on wheat bun, 1 medium patty	8000_Mixed	32	33	-1	4	2	D
27510258	Double cheeseburger, on wheat bun, 2 medium patties	8000_Mixed	32	33	-1	4	2	D
27564400	Frankfurter or hot dog sandwich, fat free, plain, on whole grain white bread	8000_Mixed	32	33	-1	4	3.5	C
28140150	Chicken divan, frozen meal	8000_Mixed	32	33	-1	4	3.5	C
28340179	Beef broth, less or reduced sodium, canned or ready-to-serve	8000_Mixed	32	33	-1	4	3.5	B
58306100	Chicken enchilada, diet frozen meal	8000_Mixed	32	33	-1	4	3.5	C
58402020	Beef dumpling soup, home recipe, canned or ready-to-serve	8000_Mixed	32	32	0	4	3.5	C
58402030	Beef rice soup, home recipe, canned or ready-to-serve	8000_Mixed	32	32	0	4	3.5	C
75656060	Vegetable beef soup, chunky style	8000_Mixed	32	32	0	4	3.5	C
14620330	Topping from meat and vegetable pizza	8000_Mixed	32	31	1	4	1.5	E
75608200	Onion soup, made from dry mix	8000_Mixed	32	31	1	4	3	C
28315130	Beef vegetable soup with rice, stew type, chunky style	8000_Mixed	32	29	3	4	3.5	B
28152030	Seafood newburg with rice, vegetable (frozen meal)	8000_Mixed	32	26	6	4	3	C
58100130	Burrito with beef and cheese, no beans	8000_Mixed	32	26	6	3	1.5	D
27520530	Pork sandwich, with gravy	8000_Mixed	32	25	7	3	3.5	C
58100340	Burrito with eggs, sausage, cheese and vegetables	8000_Mixed	32	25	7	3	2.5	D

58101740	Soft taco with egg and potato	8000_Mixed	32	25	7	3	3	D
58106530	Pizza with meat, thick crust	8000_Mixed	32	25	7	2	2	D
58163510	Rice dressing	8000_Mixed	32	25	7	2	3	C
27513030	Roast beef sandwich dipped in egg, fried, with gravy and spread	8000_Mixed	32	24	8	2	3	D
58100250	Burrito with chicken, rice, and cheese	8000_Mixed	32	24	8	3	3.5	C
58146402	Pasta with cream sauce and meat, home recipe	8000_Mixed	32	24	8	2	3	C
58160000	Biryani with vegetables	8000_Mixed	32	23	9	1	3.5	C
58148118	Macaroni or pasta salad, made with any type of fat free dressing	8000_Mixed	32	22	10	1	3.5	C
58151170	Sushi roll, avocado	8000_Mixed	32	22	10	1	3	C
58151230	Sushi roll, vegetable	8000_Mixed	32	22	10	1	3	C
58160292	Rice with corn, fat not added in cooking	8000_Mixed	32	21	11	1	3	C
58150320	Rice, fried, with chicken	8000_Mixed	32	20	12	1	3.5	C
14640038	Cheese sandwich, reduced fat American cheese, on white bread, with mayonnaise	8000_Mixed	31	39	-8	4	1.5	D
58130016	Lasagna with meat, frozen	8000_Mixed	31	34	-3	4	3.5	C
58146333	Pasta with tomato-based sauce, meat, and added vegetables, ready-to-heat	8000_Mixed	31	34	-3	4	3.5	B
58201045	Jelly sandwich, reduced sugar jelly, on wheat bread	8000_Mixed	31	34	-3	4	3.5	C
58201055	Jelly sandwich, reduced sugar jelly, on whole wheat bread	8000_Mixed	31	34	-3	4	3.5	C
27540235	Chicken fillet, broiled, sandwich with lettuce, tomato, and spread	8000_Mixed	31	33	-2	4	4	A
27540270	Chicken fillet, broiled, sandwich, with lettuce, tomato, and non-mayonnaise type spread	8000_Mixed	31	33	-2	4	4	A
27564362	Frankfurter or hot dog sandwich, fat free, plain, on whole wheat bun	8000_Mixed	31	33	-2	4	3.5	C
58106736	Pizza with extra meat and extra vegetables, thin crust	8000_Mixed	31	33	-2	4	2	D
58130011	Lasagna with meat	8000_Mixed	31	33	-2	4	3.5	C
27510263	Double cheeseburger, on wheat bun, 2 large patties	8000_Mixed	31	32	-1	4	2	D
28144100	Chicken and vegetable entree with noodles and cream sauce, frozen meal	8000_Mixed	31	32	-1	4	3.5	C
27510255	Double cheeseburger, on wheat bun, 2 small patties	8000_Mixed	31	31	0	4	2	D
27564302	Frankfurter or hot dog sandwich, reduced fat or light, plain, on whole wheat bun	8000_Mixed	31	31	0	4	3.5	D
27564330	Frankfurter or hot dog sandwich, reduced fat or light, plain, on whole wheat bread	8000_Mixed	31	31	0	4	3.5	D
28110660	Meatballs, Swedish, in gravy, with noodles, diet frozen meal	8000_Mixed	31	31	0	4	4	B
28310110	Beef, broth, bouillon, or consomme	8000_Mixed	31	31	0	4	3	C
28360100	Meat broth, Puerto Rican style	8000_Mixed	31	31	0	4	3	C
58145140	Macaroni or noodles with cheese and tomato	8000_Mixed	31	30	1	3	3.5	C
14640068	Cheese sandwich, reduced fat Cheddar cheese, on white bread, with butter	8000_Mixed	31	29	2	3	1.5	E
58104535	Chimichanga with meat and sour cream	8000_Mixed	31	29	2	3	2	D
58109030	Pizza, with meat, whole wheat thin crust	8000_Mixed	31	29	2	3	2	D
27118130	Stewed dried beef, Puerto Rican style	8000_Mixed	31	27	4	3	0.5	E
58107050	Pizza, no cheese, thin crust	8000_Mixed	31	26	5	2	2	D

27513060	Roast beef sandwich with bacon and cheese sauce	8000_Mixed	31	25	6	3	1.5	E
58106780	Pizza with meat and vegetables, prepared from frozen, lowfat, thin crust	8000_Mixed	31	24	7	2	3.5	C
58107100	Pizza, no cheese, thick crust	8000_Mixed	31	24	7	2	3	D
58164560	Rice, white, with soy-based sauce, NS as to fat	8000_Mixed	31	24	7	1	3	C
58164580	Rice, white, with soy-based sauce, fat added	8000_Mixed	31	24	7	1	3	C
27212100	Beef and noodles with tomato-based sauce	8000_Mixed	31	23	8	2	3.5	B
58103200	Tamale, plain, meatless, no sauce, Puerto Rican style or Carribean Style	8000_Mixed	31	23	8	2	2	E
58164510	Rice, white, with cheese and/or cream based sauce, no added fat	8000_Mixed	31	23	8	2	3	C
27220010	Meat loaf made with ham	8000_Mixed	31	22	9	3	2	D
58150330	Rice, fried, with pork	8000_Mixed	31	19	12	1	3.5	B
74701000	Tomato sandwich	8000_Mixed	30	35	-5	4	3.5	C
74605000	Tomato rice soup, canned, undiluted	8000_Mixed	30	34	-4	4	3	C
27540186	Chicken fillet sandwich, fried, on wheat bun, with cheese	8000_Mixed	30	33	-3	4	2.5	D
58104100	Nachos with cheese, meatless, no beans	8000_Mixed	30	33	-3	4	3.5	C
58106345	Pizza with cheese and extra vegetables, thin crust	8000_Mixed	30	33	-3	4	3	D
58146323	Pasta with tomato-based sauce and meat, ready-to-heat	8000_Mixed	30	33	-3	4	3.5	C
58304250	Manicotti, cheese-filled, with tomato sauce, diet frozen meal	8000_Mixed	30	33	-3	4	3.5	C
75603020	Celery soup, cream of, prepared with water, home recipe, canned or ready-to-serve	8000_Mixed	30	33	-3	4	3.5	C
75603030	Celery soup, cream of, canned, undiluted	8000_Mixed	30	33	-3	4	3	C
27520130	Bacon, chicken, and tomato club sandwich, with lettuce and spread	8000_Mixed	30	32	-2	4	3.5	D
27520135	Bacon, chicken, and tomato club sandwich, with cheese, lettuce and spread	8000_Mixed	30	32	-2	4	3.5	D
27540285	Chicken, bacon, and tomato club sandwich, with lettuce and spread	8000_Mixed	30	32	-2	4	3.5	D
58145114	Macaroni or noodles with cheese, made from dry mix	8000_Mixed	30	32	-2	4	3.5	C
14640020	Cheese sandwich, reduced fat Cheddar cheese, on white bread, no spread	8000_Mixed	30	31	-1	4	2	D
14640032	Cheese sandwich, Cheddar cheese, on white bread, with mayonnaise	8000_Mixed	30	31	-1	4	1.5	D
58301030	Veal lasagna, diet frozen meal	8000_Mixed	30	31	-1	4	3.5	C
58306010	Beef enchilada dinner, NFS, frozen meal	8000_Mixed	30	31	-1	4	3.5	C
27564340	Frankfurter or hot dog sandwich, reduced fat or light, plain, on whole grain white bread	8000_Mixed	30	30	0	4	3.5	C
27520156	Bacon, lettuce, tomato, and cheese submarine sandwich, with spread	8000_Mixed	30	28	2	4	2	D
28340110	Chicken or turkey broth, bouillon, or consomme	8000_Mixed	30	28	2	4	3	C
41601160	Bean and ham soup, canned, reduced sodium, prepared with water or ready-to-serve	8000_Mixed	30	27	3	4	4	B
58125180	Cheese quiche, meatless	8000_Mixed	30	27	3	3	1	D
58109040	Pizza, with meat, whole wheat thick crust	8000_Mixed	30	26	4	3	2.5	D
58104900	Taquito or flauta with egg	8000_Mixed	30	24	6	3	2.5	D
58132710	Spaghetti with tomato sauce and frankfurters or hot dogs	8000_Mixed	30	24	6	3	3	C

58116310	Cheese turnover, Puerto Rican style	8000_Mixed	30	23	7	2	1	E
58126110	Turnover, meat-filled, no gravy	8000_Mixed	30	23	7	2	2	D
27520500	Pork sandwich, on white roll, with onions, dill pickles and barbecue sauce	8000_Mixed	30	22	8	3	3	D
58106910	Pizza with seafood, thin crust	8000_Mixed	30	22	8	2	1.5	D
58106915	Pizza with seafood, regular crust	8000_Mixed	30	22	8	2	2	D
58106920	Pizza with seafood, thick crust	8000_Mixed	30	22	8	2	2	D
58113110	Dumpling, fried, pork	8000_Mixed	30	22	8	2	3	D
58121510	Dumpling, meat-filled	8000_Mixed	30	22	8	2	2	D
58147330	Macaroni or noodles, creamed, with cheese	8000_Mixed	30	21	9	2	3	C
58116210	Meat pie, Puerto Rican style	8000_Mixed	30	20	10	2	0.5	E
58155610	Rice meal fritter, Puerto Rican style	8000_Mixed	30	20	10	1	2	D
27540185	Chicken fillet sandwich, fried, on wheat bun	8000_Mixed	29	33	-4	4	3.5	D
74605010	Tomato rice soup, prepared with water	8000_Mixed	29	33	-4	4	3	C
14640200	Cheese sandwich, hoagie	8000_Mixed	29	31	-2	4	1.5	E
27510359	Cheeseburger, 1/3 lb meat, with mayonnaise or salad dressing, and mushrooms, on bun	8000_Mixed	29	31	-2	4	3	D
27510576	Hamburger, on wheat bun, 1 small patty	8000_Mixed	29	31	-2	4	3.5	D
27510632	Hamburger, on wheat bun, 1 medium patty	8000_Mixed	29	31	-2	4	3.5	D
27540280	Chicken fillet, broiled, sandwich with cheese, on bun, with lettuce, tomato and spread	8000_Mixed	29	31	-2	4	4	A
58134620	Tortellini, cheese-filled, meatless, with tomato sauce	8000_Mixed	29	31	-2	4	3.5	C
27510636	Hamburger, on wheat bun, 1 large patty	8000_Mixed	29	30	-1	4	3.5	D
27520165	Bacon, breaded fried chicken fillet, and tomato club with lettuce and spread	8000_Mixed	29	30	-1	4	3	D
28113140	Beef with spaetzle or rice, vegetable, frozen meal	8000_Mixed	29	30	-1	4	3.5	C
58106441	Pizza with chicken and vegetables, thin crust	8000_Mixed	29	30	-1	4	2	D
58106737	Pizza with extra meat and extra vegetables, thick crust	8000_Mixed	29	30	-1	4	3	D
58304050	Spaghetti with meat and mushroom sauce, diet frozen meal	8000_Mixed	29	30	-1	4	3.5	C
58402010	Beef noodle soup, canned or ready-to-serve	8000_Mixed	29	30	-1	4	3.5	C
27510652	Double hamburger, on wheat bun, 2 small patties	8000_Mixed	29	29	0	4	3.5	D
28110120	Beef with potatoes (frozen meal, large meat portion)	8000_Mixed	29	29	0	4	3	D
14640185	Grilled cheese sandwich, reduced fat Cheddar cheese, on white bread	8000_Mixed	29	28	1	3	1.5	E
28141200	Chicken teriyaki with rice, vegetable (frozen meal)	8000_Mixed	29	28	1	4	3	C
28315100	Beef vegetable soup with potato, stew type	8000_Mixed	29	28	1	4	3.5	C
28340610	Chicken or turkey vegetable soup, stew type	8000_Mixed	29	28	1	4	3.5	B
28340640	Chicken or turkey vegetable soup with noodles, stew type, chunky style, canned or ready-to-serve	8000_Mixed	29	28	1	4	3.5	B
58125110	Quiche with meat, poultry or fish	8000_Mixed	29	26	3	3	1	E
27146400	Chicken kiev	8000_Mixed	29	25	4	3	1.5	D
58104450	Chimichanga with beef and tomato	8000_Mixed	29	24	5	3	2	D

32202034	Egg, cheese, and sausage on bun	8000_Mixed	29	23	6	3	2	D
27120080	Ham stroganoff	8000_Mixed	29	21	8	3	2.5	D
27510130	Beef barbecue submarine sandwich, on bun	8000_Mixed	29	21	8	3	3.5	C
58150340	Rice, fried, with beef	8000_Mixed	29	18	11	1	3.5	B
58163210	Rice, creamed	8000_Mixed	29	18	11	1	3	C
14640026	Cheese sandwich, American cheese, on white bread, with mayonnaise	8000_Mixed	28	33	-5	4	1.5	E
58106300	Pizza, cheese, with vegetables, from frozen, thin crust	8000_Mixed	28	32	-4	4	3.5	C
74601000	Tomato soup, NFS	8000_Mixed	28	32	-4	4	3.5	C
27420020	Ham or pork salad	8000_Mixed	28	31	-3	4	2	D
27510380	Triple cheeseburger (3 patties, 1/4 lb meat each), with mayonnaise or salad dressing and tomatoes and/or catsup, on bun	8000_Mixed	28	31	-3	4	2	D
28160300	Meat loaf dinner, NFS, frozen meal	8000_Mixed	28	31	-3	4	3.5	C
28160310	Meat loaf with potatoes, vegetable, frozen meal	8000_Mixed	28	31	-3	4	3.5	C
58100325	Burrito with beans, meatless, from fast food	8000_Mixed	28	31	-3	4	3.5	C
74602030	Tomato soup, canned, undiluted	8000_Mixed	28	31	-3	4	3	C
28145610	Turkey with gravy, dressing, potatoes, vegetable, dessert, frozen meal	8000_Mixed	28	30	-2	4	3.5	C
27510659	Double hamburger, on wheat bun, 2 large patties	8000_Mixed	28	29	-1	4	2	D
58106411	Pizza with chicken, thin crust	8000_Mixed	28	29	-1	4	2	D
58106720	Pizza with meat and vegetables, from restaurant or fast food, thin crust	8000_Mixed	28	29	-1	4	2	D
58106738	Pizza with extra meat and extra vegetables, medium crust	8000_Mixed	28	29	-1	4	3.5	D
27510657	Double hamburger, on wheat bun, 2 medium patties	8000_Mixed	28	28	0	4	3.5	D
41601110	Bean and ham soup, chunky style, canned or ready-to-serve	8000_Mixed	28	28	0	4	3.5	B
58100370	Chilaquiles, tortilla casserole with salsa and cheese, no egg	8000_Mixed	28	28	0	4	2.5	C
58106443	Pizza with chicken and vegetables, thick crust	8000_Mixed	28	28	0	4	3	D
58134613	Tortellini, meat-filled, with tomato sauce, canned	8000_Mixed	28	28	0	4	3.5	B
58106442	Pizza with chicken and vegetables, regular crust	8000_Mixed	28	27	1	4	3.5	D
58131520	Ravioli, cheese-filled, with tomato sauce	8000_Mixed	28	27	1	4	3.5	C
75609000	Pea soup, NFS	8000_Mixed	28	27	1	4	3.5	C
75609020	Pea soup, prepared with water	8000_Mixed	28	27	1	4	3.5	C
28340510	Chicken or turkey noodle soup, chunky style, canned or ready-to-serve	8000_Mixed	28	26	2	4	3.5	B
58404510	Chicken or turkey soup with dumplings and potatoes, home recipe, canned, or ready-to-serve	8000_Mixed	28	26	2	4	3.5	B
58404520	Chicken or turkey soup with dumplings, home recipe, canned or ready-to-serve	8000_Mixed	28	26	2	4	3.5	B
14640056	Cheese sandwich, Cheddar cheese, on white bread, with butter	8000_Mixed	28	25	3	3	1.5	E
58107232	White pizza, cheese, with meat and vegetables, thin crust	8000_Mixed	28	25	3	3	2	D
75604020	Corn soup, cream of, prepared with water	8000_Mixed	28	24	4	3	3.5	B
58104905	Taquito or flauta with egg and breakfast meat	8000_Mixed	28	22	6	3	2	D
28345120	Chicken or turkey soup, cream of, prepared with milk	8000_Mixed	28	21	7	3	3	C

58146382	Pasta with cream sauce, home recipe	8000_Mixed	28	21	7	2	3	C
58100155	Burrito with beef, rice, and cheese	8000_Mixed	28	20	8	3	3	D
27220190	Sausage and noodles with cream or white sauce	8000_Mixed	28	18	10	2	3	C
58123110	Sweet bread dough, filled with meat, steamed	8000_Mixed	28	18	10	1	3.5	C
58165070	Rice, white, with vegetables, soy-based sauce, no added fat	8000_Mixed	28	18	10	1	3	C
27510676	Double hamburger, 2 medium patties, with condiments, on bun, from fast food / restaurant	8000_Mixed	27	30	-3	4	3.5	D
27510681	Double hamburger, 2 medium patties, with condiments, on bun, from fast food / restaurant (Burger King Double WHOPPER)	8000_Mixed	27	30	-3	4	3.5	D
27510682	Double hamburger, 2 medium patties, with condiments, on bun, from fast food / restaurant (Wendy's 1/2 lb Double)	8000_Mixed	27	30	-3	4	3.5	D
27510690	Double hamburger (2 patties, 1/4 lb meat each), with mayonnaise or salad dressing and tomatoes and/or catsup, on double-decker bun	8000_Mixed	27	30	-3	4	3.5	D
58106320	Pizza, cheese, with vegetables, from restaurant or fast food, thin crust	8000_Mixed	27	30	-3	4	2	D
58106350	Pizza with cheese and extra vegetables, thick crust	8000_Mixed	27	30	-3	4	3	D
58106358	Pizza, cheese, with fruit, thin crust	8000_Mixed	27	30	-3	4	2	D
58121610	Dumpling, potato- or cheese-filled	8000_Mixed	27	30	-3	4	3	C
74602010	Tomato soup, prepared with water, or ready-to-serve	8000_Mixed	27	30	-3	4	3.5	C
74606010	Tomato vegetable soup, prepared with water	8000_Mixed	27	30	-3	4	3.5	C
14640050	Cheese sandwich, American cheese, on white bread, with butter	8000_Mixed	27	29	-2	4	1	E
28130000	Veal dinner, NFS, frozen meal	8000_Mixed	27	29	-2	4	3.5	C
28133110	Veal, breaded, with spaghetti, in tomato sauce, frozen meal	8000_Mixed	27	29	-2	4	3.5	C
58106347	Pizza with cheese and extra vegetables, medium crust	8000_Mixed	27	29	-2	4	3	D
27510266	Cheeseburger, 1 large patty, with condiments, on bun, from fast food / restaurant	8000_Mixed	27	28	-1	4	3	D
28133340	Veal parmigiana with vegetable, fettuccine alfredo, dessert, frozen meal	8000_Mixed	27	28	-1	4	3.5	C
41602010	Pea and ham soup, chunky style, canned or ready-to-serve	8000_Mixed	27	28	-1	4	3.5	B
41602030	Split pea and ham soup	8000_Mixed	27	28	-1	4	3.5	B
58302060	Spaghetti or noodles with beef in tomato-based sauce, lowfat, reduced sodium (diet frozen meal)	8000_Mixed	27	28	-1	4	3.5	B
58303200	Rice, with green beans, water chestnuts, in sherry mushroom sauce, frozen side dish	8000_Mixed	27	28	-1	4	3.5	B
58304060	Spaghetti with meat sauce, diet frozen meal	8000_Mixed	27	28	-1	4	3.5	B
75607050	Mushroom soup, cream of, low sodium, prepared with water	8000_Mixed	27	28	-1	4	3.5	B
27510346	Bacon cheeseburger, 1 large patty, with condiments, on bun, from fast food / restaurant	8000_Mixed	27	27	0	4	3	D
27564410	Frankfurter or hot dog sandwich, fat free, plain, on multigrain bread	8000_Mixed	27	27	0	4	3.5	C
28110110	Beef with potatoes (frozen meal)	8000_Mixed	27	27	0	4	3.5	C
58106413	Pizza with chicken, thick crust	8000_Mixed	27	27	0	4	3	D
58106735	Pizza with extra meat and extra vegetables, NS as to type of crust	8000_Mixed	27	27	0	4	3.5	D
58403030	Chicken noodle soup, canned, low sodium, ready-to-serve	8000_Mixed	27	27	0	4	3.5	B
27510451	Double bacon cheeseburger, 2 medium patties, with condiments, on bun, from fast food / restaurant	8000_Mixed	27	26	1	4	2	D

27510465	Double bacon cheeseburger, 2 medium patties, with condiments, on bun, from fast food / restaurant (Wendy's Baconator)	8000_Mixed	27	26	1	4	2	D
28143220	Chicken in barbecue sauce, with rice, vegetable and dessert, reduced fat and sodium (diet frozen meal)	8000_Mixed	27	26	1	4	4	A
28310210	Chili beef soup	8000_Mixed	27	26	1	4	3.5	C
58106412	Pizza with chicken, regular crust	8000_Mixed	27	26	1	4	3	D
58106462	Pizza with chicken and fruit, regular crust	8000_Mixed	27	26	1	4	3.5	D
14640125	Grilled cheese sandwich, Cheddar cheese, on white bread	8000_Mixed	27	25	2	3	1.5	E
58145112	Macaroni or noodles with cheese, made from packaged mix	8000_Mixed	27	25	2	4	3	C
27520410	Cuban sandwich, with spread	8000_Mixed	27	23	4	3	3.5	D
35001000	Scrambled eggs, sausage, hash brown potatoes (frozen meal)	8000_Mixed	27	23	4	4	3	D
58310110	Frozen breakfast, NFS (frozen meal)	8000_Mixed	27	23	4	4	3	D
35002000	Scrambled eggs, bacon, home fried potatoes (frozen meal)	8000_Mixed	27	22	5	4	3	D
58100410	Burrito with beef, cheese, and sour cream	8000_Mixed	27	21	6	3	3	C
58107210	White pizza, NS as to type of crust	8000_Mixed	27	20	7	2	2	D
58107225	White pizza, regular crust	8000_Mixed	27	20	7	2	2	D
58122220	Gnocchi, potato	8000_Mixed	27	18	9	1	3	C
27220020	Ham and noodles with cream or white sauce	8000_Mixed	27	16	11	2	3	C
58150110	Rice, fried, meatless	8000_Mixed	27	16	11	1	3	C
58150310	Rice, fried, NFS	8000_Mixed	27	16	11	1	3	C
58104830	Taquito or flauta with chicken	8000_Mixed	26	30	-4	4	3.5	C
58104835	Taquito or flauta with chicken and cheese	8000_Mixed	26	30	-4	4	3.5	C
58106250	Pizza, extra cheese, thin crust	8000_Mixed	26	29	-3	4	2	E
58106330	Pizza, cheese, with vegetables, from restaurant or fast food, thick crust	8000_Mixed	26	29	-3	4	3	D
58106360	Pizza, cheese, with fruit, thick crust	8000_Mixed	26	29	-3	4	3	D
27510430	Double bacon cheeseburger (2 patties, 1/4 lb meat each), with mayonnaise or salad dressing, and tomato and/or catsup, on bun	8000_Mixed	26	28	-2	4	2	D
27510435	Double bacon cheeseburger (2 patties,1/3 lb meat each), with mayonnaise or salad dressing, on bun	8000_Mixed	26	28	-2	4	1.5	D
58304010	Spaghetti and meatballs dinner, NFS, frozen meal	8000_Mixed	26	28	-2	4	3.5	B
58306150	Chicken enchilada with salsa, rice, vegetable, and dessert (diet frozen meal)	8000_Mixed	26	28	-2	4	3.5	C
27510355	Cheeseburger, 1/3 lb meat, with mayonnaise or salad dressing, tomato and/or catsup on bun	8000_Mixed	26	27	-1	4	3	D
27510560	Hamburger, 1/4 lb meat, with mayonnaise or salad dressing, and tomato and/or catsup, on bun	8000_Mixed	26	27	-1	4	3.5	C
27510615	Whopper (Burger King)	8000_Mixed	26	27	-1	4	3.5	C
27510616	Hamburger, 1 medium patty, with condiments, on bun, from fast food / restaurant (Wendy's 1/4 lb Single)	8000_Mixed	26	27	-1	4	3.5	C
27540290	Chicken submarine sandwich, with lettuce, tomato and spread	8000_Mixed	26	27	-1	4	4	B
28110230	Sirloin, chopped, or swiss steak with gravy, vegetable, potatoes, dessert or muffin (frozen meal)	8000_Mixed	26	27	-1	4	4	B
41602050	Split pea soup	8000_Mixed	26	27	-1	4	3.5	C

58106650	Pizza with extra meat, thin crust	8000_Mixed	26	27	-1	4	1.5	D
58301080	Lasagna with cheese and meat sauce, reduced fat and sodium (diet frozen meal)	8000_Mixed	26	27	-1	4	3.5	B
58401010	Barley soup, home recipe, canned, or ready-to-serve	8000_Mixed	26	27	-1	4	3.5	B
27540291	Chicken submarine sandwich, with cheese, lettuce, tomato and spread	8000_Mixed	26	26	0	4	4	B
28143200	Chicken in soy-based sauce, rice and vegetables, frozen meal	8000_Mixed	26	26	0	4	3.5	B
58112510	Dumpling, steamed, filled with meat, poultry, or seafood	8000_Mixed	26	26	0	4	3.5	C
58132113	Pasta with tomato sauce and cheese, canned	8000_Mixed	26	26	0	4	3.5	C
58132313	Pasta with tomato sauce and meat or meatballs, canned	8000_Mixed	26	26	0	4	3.5	C
75651120	Vegetable chicken noodle soup, prepared with water or ready-to-serve	8000_Mixed	26	26	0	4	3	C
27510446	Double bacon cheeseburger, 2 medium patties, plain, on bun, from fast food / restaurant	8000_Mixed	26	25	1	4	2	D
28133410	Veal parmigiana with potatoes, vegetable (frozen meal)	8000_Mixed	26	25	1	4	3.5	C
28140100	Chicken dinner, NFS, frozen meal	8000_Mixed	26	25	1	4	3.5	C
28140710	Chicken, fried, with potatoes, vegetable, frozen meal	8000_Mixed	26	25	1	4	3.5	C
28143170	Chicken in cream sauce with noodles and vegetable, frozen meal	8000_Mixed	26	25	1	4	3.5	C
28145710	Turkey tetrazzini, frozen meal	8000_Mixed	26	25	1	4	3.5	B
58104730	Quesadilla with meat	8000_Mixed	26	24	2	3	1.5	E
58104740	Quesadilla with chicken	8000_Mixed	26	24	2	3	1.5	E
58107222	White pizza, cheese, with meat, thin crust	8000_Mixed	26	23	3	3	2	D
58124220	Pastry, egg and cheese filled	8000_Mixed	26	19	7	2	2	D
27520330	Ham and egg sandwich	8000_Mixed	26	15	11	3	3.5	C
27540170	Chicken patty sandwich, miniature, with spread	8000_Mixed	25	29	-4	4	3	D
27540190	Chicken patty sandwich, with lettuce and spread	8000_Mixed	25	29	-4	4	3	D
58106220	Pizza, cheese, from restaurant or fast food, thin crust	8000_Mixed	25	29	-4	4	2	E
58201015	Jelly sandwich, regular jelly, on wheat bread	8000_Mixed	25	29	-4	4	3	D
58201025	Jelly sandwich, regular jelly, on whole wheat bread	8000_Mixed	25	29	-4	4	3	D
27510300	Double cheeseburger (2 patties), with mayonnaise or salad dressing, on double-decker bun	8000_Mixed	25	28	-3	4	2	D
27510370	Double cheeseburger (2 patties, 1/4 lb meat each), with mayonnaise or salad dressing, on bun	8000_Mixed	25	28	-3	4	2	D
58104820	Taquito or flauta with meat	8000_Mixed	25	28	-3	4	3	C
58104825	Taquito or flauta with meat and cheese	8000_Mixed	25	28	-3	4	3	C
58106260	Pizza, extra cheese, thick crust	8000_Mixed	25	28	-3	4	2.5	D
58106305	Pizza, cheese with vegetables, from frozen, thick crust	8000_Mixed	25	28	-3	4	3	D
58106500	Pizza with meat, prepared from frozen, thin crust	8000_Mixed	25	28	-3	4	3	D
58106730	Pizza with meat and vegetables, from restaurant or fast food, thick crust	8000_Mixed	25	28	-3	4	2.5	D
51182020	Bread stuffing made with egg	8000_Mixed	25	27	-2	4	2.5	C
75607040	Mushroom soup, with meat broth, prepared with water	8000_Mixed	25	27	-2	4	3	C

27510440	Bacon cheeseburger, 1/4 lb meat, with mayonnaise or salad dressing, and tomato and/or catsup, on bun	8000_Mixed	25	26	-1	4	2	D
28110350	Salisbury steak with gravy, potatoes, vegetable, dessert, frozen meal	8000_Mixed	25	26	-1	4	3.5	C
28110500	Beef, sliced, with gravy, barley and wild rice, vegetables (diet frozen meal)	8000_Mixed	25	26	-1	4	4	B
28143020	Chicken and vegetable entree with rice, diet frozen meal	8000_Mixed	25	26	-1	4	3.5	C
58106660	Pizza with extra meat, thick crust	8000_Mixed	25	26	-1	4	2	D
74602050	Tomato soup, instant type, prepared with water	8000_Mixed	25	26	-1	4	3	C
27510171	Whopper Jr with cheese (Burger King)	8000_Mixed	25	25	0	4	3	D
27510257	Double cheeseburger, on white bun, 2 medium patties	8000_Mixed	25	25	0	4	2	D
27510265	Double cheeseburger, (2 patties, 1 oz each), plain, on miniature bun	8000_Mixed	25	25	0	4	2	D
27510401	Double cheeseburger, from fast food, 2 medium patties	8000_Mixed	25	25	0	4	2	D
27520150	Bacon, lettuce, and tomato sandwich with spread	8000_Mixed	25	25	0	4	3	C
27564242	Frankfurter or hot dog sandwich, chicken and/or turkey, plain, on whole wheat bun	8000_Mixed	25	25	0	4	3	D
27564280	Frankfurter or hot dog sandwich, chicken and/or turkey, plain, on whole grain white bread	8000_Mixed	25	25	0	4	3.5	D
27564350	Frankfurter or hot dog sandwich, reduced fat or light, plain, on multigrain bread	8000_Mixed	25	25	0	4	3.5	D
27564363	Frankfurter or hot dog sandwich, fat free, plain, on whole grain white bun	8000_Mixed	25	25	0	4	3.5	D
28110370	Salisbury steak with gravy, macaroni and cheese, vegetable, frozen meal	8000_Mixed	25	25	0	4	3	C
28143040	Chicken chow mein with rice, diet frozen meal	8000_Mixed	25	25	0	4	3.5	C
58100110	Burrito with beef and beans	8000_Mixed	25	25	0	4	3.5	D
58106655	Pizza with extra meat, medium crust	8000_Mixed	25	25	0	4	2	D
58145119	Macaroni or noodles with cheese, made from reduced fat packaged mix	8000_Mixed	25	25	0	4	3.5	C
58406010	Turkey noodle soup	8000_Mixed	25	25	0	4	3	C
14640008	Cheese sandwich, Cheddar cheese, on white bread, no spread	8000_Mixed	25	24	1	3	1.5	D
27510431	Double bacon cheeseburger, 2 small patties, with condiments, on bun, from fast food / restaurant (Burger King Bacon Double Cheeseburger)	8000_Mixed	25	24	1	4	2	D
27510480	Cheeseburger (hamburger with cheese sauce), 1/4 lb meat, with grilled onions, on rye bun	8000_Mixed	25	24	1	4	3.5	C
58104760	Quesadilla with vegetables and meat	8000_Mixed	25	24	1	3	2	D
58104770	Quesadilla with vegetables and chicken	8000_Mixed	25	24	1	3	2	D
58400000	Soup, NFS	8000_Mixed	25	24	1	4	3	C
28110520	Beef, sliced, with gravy, potatoes, vegetable, dessert (frozen meal)	8000_Mixed	25	23	2	4	3.5	C
58403010	Chicken or turkey noodle soup, canned or ready-to-serve	8000_Mixed	25	23	2	4	3	C
27220120	Sausage and rice with tomato-based sauce	8000_Mixed	25	16	9	2	2.5	C
58156310	Rice with Spanish sausage, Puerto Rican style	8000_Mixed	25	16	9	2	1.5	D
27510340	Double cheeseburger (2 patties), with mayonnaise or salad dressing and tomatoes and/or catsup, on bun	8000_Mixed	24	27	-3	4	2	D
27513040	Roast beef submarine sandwich, with lettuce, tomato and spread	8000_Mixed	24	27	-3	4	3.5	C

27517010	Wrap sandwich filled with beef patty, cheese, tomato and/or catsup, and spread and/or sauce	8000_Mixed	24	27	-3	4	2	D
58106205	Pizza, cheese, from frozen, thick crust	8000_Mixed	24	27	-3	4	2.5	D
58106230	Pizza, cheese, from restaurant or fast food, thick crust	8000_Mixed	24	27	-3	4	2.5	D
58106359	Pizza, cheese, with fruit, medium crust	8000_Mixed	24	27	-3	4	3.5	D
58106550	Pizza with pepperoni, from restaurant or fast food, thin crust	8000_Mixed	24	27	-3	4	1.5	E
14640062	Cheese sandwich, reduced fat American cheese, on white bread, with butter	8000_Mixed	24	26	-2	4	1.5	E
27510000	Beef sandwich, NFS	8000_Mixed	24	26	-2	4	3.5	C
27510175	Cheeseburger, 1 small patty, with condiments, on bun, from fast food / restaurant (Wendy's Jr. Cheeseburger Deluxe)	8000_Mixed	24	26	-2	4	3	D
27510389	Big Mac (McDonalds)	8000_Mixed	24	26	-2	4	2	D
27510425	Double bacon cheeseburger (2 patties, 1/4 lb meat each), with mayonnaise or salad dressing, on bun	8000_Mixed	24	26	-2	4	1.5	D
27513041	Roast beef submarine sandwich, with cheese, lettuce, tomato and spread	8000_Mixed	24	26	-2	4	3.5	C
27520120	Bacon and cheese sandwich, with spread	8000_Mixed	24	26	-2	4	1.5	E
27540175	Chicken fillet sandwich, fried, on white bun	8000_Mixed	24	26	-2	4	3.5	D
28340690	Chicken or turkey vegetable soup with potato and cheese, chunky style, canned or ready-to-serve	8000_Mixed	24	26	-2	4	3	C
58106200	Pizza, cheese, from frozen, thin crust	8000_Mixed	24	26	-2	4	2.5	D
58106325	Pizza, cheese, with vegetables, from restaurant or fast food, medium crust	8000_Mixed	24	26	-2	4	3	D
58106560	Pizza with pepperoni, from restaurant or fast food, thick crust	8000_Mixed	24	26	-2	4	2	D
58112110	Dim sum, meat filled (egg roll-type)	8000_Mixed	24	26	-2	4	3	D
58306070	Cheese enchilada, frozen meal	8000_Mixed	24	26	-2	4	2.5	D
58304300	Cannelloni, cheese-filled, with tomato sauce, diet frozen meal	8000_Mixed	24	25	-1	4	3	C
58306020	Beef enchilada, chili gravy, rice, refried beans, frozen meal	8000_Mixed	24	25	-1	4	3.5	C
14640100	Grilled cheese sandwich, NFS	8000_Mixed	24	24	0	4	1	E
14640105	Grilled cheese sandwich, American cheese, on white bread	8000_Mixed	24	24	0	4	1	E
27510262	Double cheeseburger, on white bun, 2 large patties	8000_Mixed	24	24	0	4	2	D
27510360	Bacon cheeseburger, with mayonnaise or salad dressing, tomato and/or catsup, on bun	8000_Mixed	24	24	0	4	2	D
27510405	Double cheeseburger, from fast food, 2 large patties	8000_Mixed	24	24	0	4	2	D
27564270	Frankfurter or hot dog sandwich, chicken and/or turkey, plain, on whole wheat bread	8000_Mixed	24	24	0	4	2.5	D
28330110	Scotch broth (lamb, vegetables, and barley)	8000_Mixed	24	24	0	4	3	C
27510245	Cheeseburger, on white bun, 1 large patty	8000_Mixed	24	23	1	4	2	D
27510261	Cheeseburger, from fast food, 1 large patty	8000_Mixed	24	23	1	4	2	D
28101000	Frozen dinner, NFS	8000_Mixed	24	23	1	4	3.5	C
28110000	Beef dinner, NFS, frozen meal	8000_Mixed	24	23	1	4	3.5	C
28110200	Sirloin, chopped, dinner, NFS (frozen meal)	8000_Mixed	24	23	1	4	3	C
28110300	Salisbury steak dinner, NFS, frozen meal	8000_Mixed	24	23	1	4	3.5	C
28110310	Salisbury steak with gravy, potatoes, vegetable, frozen meal	8000_Mixed	24	23	1	4	3.5	C

58104720	Quesadilla, just cheese, from fast food	8000_Mixed	24	23	1	3	1.5	E
58104750	Quesadilla with vegetables	8000_Mixed	24	23	1	4	2	D
58106640	Pizza with extra meat, NS as to type of crust	8000_Mixed	24	23	1	4	2	D
58404040	Chicken or turkey rice soup, reduced sodium, canned, prepared with water or ready-to-serve	8000_Mixed	24	23	1	4	3.5	B
58101733	Taco or tostada with meat and beans, from fast food	8000_Mixed	24	22	2	4	3.5	C
58146443	Pasta with cream sauce and seafood, ready-to-heat	8000_Mixed	24	22	2	4	3	C
58304020	Spaghetti and meatballs with tomato sauce, sliced apples, bread, frozen meal	8000_Mixed	24	22	2	4	3	C
73210110	Pumpkin fritters, Puerto Rican style	8000_Mixed	24	22	2	2	2.5	D
58107224	White pizza, cheese, with meat, thick crust	8000_Mixed	24	21	3	4	2	D
32202000	Egg, cheese, ham, and bacon on bun	8000_Mixed	24	17	7	3	2	D
27220030	Ham and rice with mushroom sauce	8000_Mixed	24	16	8	2	3	C
27220170	Sausage and rice with cheese sauce	8000_Mixed	24	15	9	3	3	C
27320020	Ham pot pie	8000_Mixed	24	15	9	3	3	D
58122250	Kishke, stuffed derma	8000_Mixed	24	14	10	2	1	E
14640014	Cheese sandwich, reduced fat American cheese, on white bread, no spread	8000_Mixed	23	26	-3	4	2	D
27510250	Cheeseburger, 1/4 lb meat, with mayonnaise or salad dressing, on bun	8000_Mixed	23	26	-3	4	2	D
27510280	Double cheeseburger (2 patties), with mayonnaise or salad dressing, on bun	8000_Mixed	23	26	-3	4	2	D
27510350	Cheeseburger, 1/4 lb meat, with mayonnaise or salad dressing, and tomato and/or catsup, on bun	8000_Mixed	23	26	-3	4	2	D
27540132	Chicken fillet sandwich, NFS	8000_Mixed	23	26	-3	4	3	D
58106516	Pizza with pepperoni, from frozen, thick crust	8000_Mixed	23	26	-3	4	2.5	D
14640155	Grilled cheese sandwich, reduced fat American cheese, on white bread	8000_Mixed	23	25	-2	4	1.5	E
27510386	Double cheeseburger (Burger King)	8000_Mixed	23	25	-2	4	2	D
27510670	Double hamburger (2 patties), with mayonnaise or salad dressing and tomatoes, on bun	8000_Mixed	23	25	-2	4	3	D
27540140	Chicken fillet, breaded, fried, sandwich	8000_Mixed	23	25	-2	4	2	D
58106514	Pizza with pepperoni, from frozen, medium crust	8000_Mixed	23	25	-2	4	2	D
58106540	Pizza with pepperoni, from restaurant or fast food, NS as to type of crust	8000_Mixed	23	25	-2	4	2	D
58106555	Pizza with pepperoni, from restaurant or fast food, medium crust	8000_Mixed	23	25	-2	4	2	D
58106606	Pizza with meat other than pepperoni, from frozen, thick crust	8000_Mixed	23	25	-2	4	2.5	D
58107205	White pizza, cheese, thin crust	8000_Mixed	23	25	-2	4	2	D
28110620	Beef short ribs, boneless, with barbecue sauce, potatoes, vegetable, frozen meal	8000_Mixed	23	24	-1	4	4	B
58106733	Pizza with extra meat and extra vegetables, prepared from frozen, thin crust	8000_Mixed	23	24	-1	4	3	D
27510235	Cheeseburger submarine sandwich with lettuce, tomato and spread	8000_Mixed	23	23	0	4	3.5	C
27510400	Bacon cheeseburger, 1/4 lb meat, with tomato and/or catsup, on bun	8000_Mixed	23	23	0	4	1.5	D

27515020	Steak and cheese submarine sandwich, with lettuce and tomato	8000_Mixed	23	23	0	4	3.5	C
27518000	Wrap sandwich filled with beef patty, bacon, cheese, tomato and/or catsup, and spread and/or sauce	8000_Mixed	23	23	0	4	1.5	D
27564303	Frankfurter or hot dog sandwich, reduced fat or light, plain, on whole grain white bun	8000_Mixed	23	23	0	4	3	D
28141010	Chicken, fried, with potatoes, vegetable, dessert, frozen meal, large meat portion	8000_Mixed	23	23	0	4	3	C
28317010	Beef stroganoff soup, chunky style, home recipe, canned or ready-to-serve	8000_Mixed	23	23	0	4	3	C
58145113	Macaroni or noodles with cheese, canned	8000_Mixed	23	23	0	4	3.5	C
58310410	Sausage rice links and whole wheat pancakes (frozen meal)	8000_Mixed	23	23	0	4	2	D
27510254	Double cheeseburger, on white bun, 2 small patties	8000_Mixed	23	22	1	4	2	D
27510281	Bacon cheeseburger, 1 small patty, with condiments, on bun, from fast food / restaurant (Wendy's Jr. Bacon Cheeseburger)	8000_Mixed	23	22	1	4	2	D
27540361	Turkey and bacon submarine sandwich, with cheese, lettuce, tomato and spread	8000_Mixed	23	22	1	4	3.5	C
28110220	Sirloin, chopped, with gravy, mashed potatoes, vegetable, frozen meal	8000_Mixed	23	22	1	4	3	C
58132713	Pasta with tomato sauce and frankfurters or hot dogs, canned	8000_Mixed	23	22	1	4	3.5	B
27510950	Reuben sandwich, corned beef sandwich with sauerkraut and cheese, with spread	8000_Mixed	23	21	2	4	2	D
58130013	Lasagna with meat, canned	8000_Mixed	23	21	2	4	3.5	C
58131323	Ravioli, meat-filled, with tomato sauce or meat sauce, canned	8000_Mixed	23	21	2	4	3.5	C
27520510	Pork barbecue sandwich or Sloppy Joe, on bun	8000_Mixed	23	19	4	3	3	D
58145150	Macaroni or noodles with cheese and pork or ham	8000_Mixed	23	19	4	4	3	D
27541000	Turkey, ham, and roast beef club sandwich, with lettuce, tomato and spread	8000_Mixed	23	18	5	3	3	C
27120120	Sausage gravy	8000_Mixed	23	15	8	3	2.5	D
58103210	Tamale, meatless, with sauce, Puerto Rican or Caribbean style	8000_Mixed	23	14	9	2	0.5	E
58156210	Rice with vienna sausage, Puerto Rican style	8000_Mixed	23	14	9	2	1.5	D
27418310	Corned beef with tomato sauce and onion, Puerto Rican style	8000_Mixed	23	13	10	3	2	D
58151400	Sushi, topped with crab	8000_Mixed	23	12	11	2	3	C
27135040	Veal with butter sauce	8000_Mixed	23	10	13	1	3	D
58106210	Pizza, cheese, from restaurant or fast food, NS as to type of crust	8000_Mixed	22	25	-3	4	2.5	D
58106225	Pizza, cheese, from restaurant or fast food, medium crust	8000_Mixed	22	25	-3	4	2.5	D
27510230	Cheeseburger, with mayonnaise or salad dressing, and tomato and/or catsup, on bun	8000_Mixed	22	24	-2	4	3	D
27510231	Whopper with cheese (Burger King)	8000_Mixed	22	24	-2	4	2	D
27510233	Cheeseburger, 1 medium patty, with condiments, on bun, from fast food / restaurant (Wendy's 1/4 lb Single with cheese)	8000_Mixed	22	24	-2	4	2	D
27520166	Bacon, breaded fried chicken fillet, and tomato club sandwich with cheese, lettuce and spread	8000_Mixed	22	24	-2	4	3	D
28110390	Salisbury steak, potatoes, vegetable, dessert, diet frozen meal	8000_Mixed	22	24	-2	4	3.5	B
28355410	Shrimp soup, cream of, NS as to prepared with milk or water	8000_Mixed	22	24	-2	4	3	C
28355420	Shrimp soup, cream of, prepared with milk	8000_Mixed	22	24	-2	4	3	C

58100125	Burrito with meat and beans, from fast food	8000_Mixed	22	24	-2	4	3.5	C
58106240	Pizza, extra cheese, NS as to type of crust	8000_Mixed	22	24	-2	4	2.5	D
58106255	Pizza, extra cheese, regular crust	8000_Mixed	22	24	-2	4	2.5	D
58106310	Pizza, cheese, with vegetables, NS as to type of crust	8000_Mixed	22	24	-2	4	3	D
58106512	Pizza with pepperoni, from frozen, thin crust	8000_Mixed	22	24	-2	4	2.5	D
58106604	Pizza with meat other than pepperoni, from frozen, medium crust	8000_Mixed	22	24	-2	4	2.5	D
58106760	Pizza with meat and fruit, thick crust	8000_Mixed	22	24	-2	4	3	D
75601020	Asparagus soup, cream of, prepared with water	8000_Mixed	22	24	-2	4	3	C
75649050	Vegetable soup, made from dry mix	8000_Mixed	22	24	-2	4	3	C
27510140	Cheeseburger slider, from fast food	8000_Mixed	22	23	-1	4	2	D
27510260	Cheeseburger, 1/4 lb meat, with mushrooms in sauce, on bun	8000_Mixed	22	23	-1	4	3	D
27510320	Cheeseburger, 1/4 lb meat, with tomato and/or catsup, on bun	8000_Mixed	22	23	-1	4	2	D
58106602	Pizza with meat other than pepperoni, from frozen, thin crust	8000_Mixed	22	23	-1	4	2.5	D
58106750	Pizza with meat and fruit, thin crust	8000_Mixed	22	23	-1	4	2	D
27510371	Double cheeseburger, from fast food, 2 small patties	8000_Mixed	22	22	0	4	2	D
27510390	Double bacon cheeseburger (2 patties, 1/4 lb meat each), on bun	8000_Mixed	22	22	0	4	2	D
27510667	Double hamburger, 2 small patties, with condiments, on bun, from fast food / restaurant	8000_Mixed	22	22	0	4	3.5	C
27564122	Frankfurter or hot dog sandwich, beef and pork, plain, on whole wheat bun	8000_Mixed	22	22	0	4	2	D
28145010	Turkey with dressing, gravy, potato (frozen meal)	8000_Mixed	22	22	0	4	3	C
27564502	Frankfurter or hot dog sandwich, with meatless chili, on whole wheat bun	8000_Mixed	22	21	1	4	3	D
28141630	Chicken cordon bleu with vegetable, rice (frozen meal)	8000_Mixed	22	21	1	4	3.5	B
28345040	Chicken or turkey soup, cream of, canned, reduced sodium, undiluted	8000_Mixed	22	21	1	4	3.5	C
58104710	Quesadilla, just cheese, meatless	8000_Mixed	22	21	1	4	1.5	E
58162310	Rice pilaf	8000_Mixed	22	21	1	4	3	C
58407000	Instant soup, NFS	8000_Mixed	22	21	1	4	3.5	B
27112010	Salisbury steak with gravy	8000_Mixed	22	20	2	4	3	C
27564160	Frankfurter or hot dog sandwich, beef and pork, plain, on whole grain white bread	8000_Mixed	22	20	2	4	2	D
28140250	Chicken, boneless, with gravy, dressing, rice, vegetable, dessert, frozen meal	8000_Mixed	22	20	2	4	3.5	C
27564540	Frankfurter or hot dog sandwich, with meatless chili, on whole grain white bread	8000_Mixed	22	19	3	4	3	D
28143210	Chicken in orange sauce with almond rice, diet frozen meal	8000_Mixed	22	19	3	4	4	B
58104800	Taquito or flauta with cheese	8000_Mixed	22	19	3	4	3.5	C
58100560	Enchilada with ham and cheese, no beans	8000_Mixed	22	18	4	4	3.5	C
58163110	Rice with gravy	8000_Mixed	22	10	12	1	3	C
58151100	Sushi, NFS	8000_Mixed	22	9	13	2	3	C
58151180	Sushi roll, California	8000_Mixed	22	9	13	2	3	C
27510220	Cheeseburger, with mayonnaise or salad dressing, on bun	8000_Mixed	21	24	-3	4	2	D

58106233	Pizza, cheese, stuffed crust	8000_Mixed	21	24	-3	4	2	D
27510145	Cheeseburger, 1 miniature patty, with condiments, on miniature bun, from fast food / restaurant	8000_Mixed	21	23	-2	4	2	D
27510550	Double hamburger (2 patties), with mayonnaise or salad dressing and tomatoes, on double-decker bun	8000_Mixed	21	23	-2	4	3	D
27510660	Double hamburger (2 patties), with mayonnaise or salad dressing, on bun	8000_Mixed	21	23	-2	4	3	D
27540151	Chicken fillet, breaded, fried, sandwich with cheese, lettuce, tomato and spread	8000_Mixed	21	23	-2	4	2	D
58106620	Pizza with meat other than pepperoni, from restaurant or fast food, thin crust	8000_Mixed	21	23	-2	4	1.5	E
58106702	Pizza with meat and vegetables, from frozen, medium crust	8000_Mixed	21	23	-2	4	2	D
27510195	Cheeseburger, on white bun, 1 small patty	8000_Mixed	21	22	-1	4	2	D
27510240	Cheeseburger, 1/4 lb meat, plain, on bun	8000_Mixed	21	22	-1	4	2	D
27510312	Bacon cheeseburger, 1 medium patty, with condiments, on bun, from fast food / restaurant	8000_Mixed	21	22	-1	4	1.5	D
27510330	Double cheeseburger (2 patties), with tomato and/or catsup, on bun	8000_Mixed	21	22	-1	4	2	D
27510376	Double cheeseburger, 2 small patties, with condiments, on bun, from fast food / restaurant	8000_Mixed	21	22	-1	4	2	D
27510570	Hamburger, 2-1/2 oz meat, with mayonnaise or salad dressing and tomatoes, on bun	8000_Mixed	21	22	-1	4	3.5	C
27510630	Hamburger, 1/4 lb meat, with mayonnaise or salad dressing, on bun	8000_Mixed	21	22	-1	4	3	D
58126150	Turnover, meat- and cheese-filled, tomato-based sauce	8000_Mixed	21	22	-1	4	3	D
27510155	Cheeseburger, NFS	8000_Mixed	21	21	0	4	2	D
27510191	Cheeseburger slider	8000_Mixed	21	21	0	4	2	D
27510215	Cheeseburger, from fast food, 1 medium patty	8000_Mixed	21	21	0	4	2	D
27510225	Cheeseburger, 1 medium patty, with condiments, on bun, from fast food / restaurant	8000_Mixed	21	21	0	4	2	D
27510232	Quarter Pounder with cheese (McDonalds)	8000_Mixed	21	21	0	4	2	D
27510241	Cheeseburger, on white bun, 1 medium patty	8000_Mixed	21	21	0	4	2	D
27510385	Double bacon cheeseburger (2 patties), with tomato and/or catsup, on bun	8000_Mixed	21	21	0	4	2	D
27510475	Double bacon cheeseburger, 2 large patties, with condiments, on bun, from fast food / restaurant	8000_Mixed	21	21	0	4	1.5	E
27510486	Triple cheeseburger, 3 medium patties, with condiments, on bun, from fast food / restaurant	8000_Mixed	21	21	0	4	2	D
28110340	Salisbury steak with gravy, potatoes, vegetable, soup or macaroni and cheese, dessert, frozen meal	8000_Mixed	21	21	0	4	3.5	C
52204000	Cornbread stuffing	8000_Mixed	21	21	0	4	3	C
27510305	Bacon cheeseburger, 1 medium patty, plain, on bun, from fast food / restaurant	8000_Mixed	21	20	1	4	1.5	E
27510410	Chiliburger, on bun	8000_Mixed	21	20	1	4	3.5	D
27510658	Double hamburger, on white bun, 2 large patties	8000_Mixed	21	20	1	4	2	D
27510675	Double hamburger, from fast food, 2 large patties	8000_Mixed	21	20	1	4	2	D
28140810	Chicken, fried, with potatoes, vegetable, dessert, frozen meal	8000_Mixed	21	20	1	4	3	D
28141640	Chicken kiev with rice-vegetable mixture (frozen meal)	8000_Mixed	21	20	1	4	3	C
58101323	Taco or tostada with meat, from fast food	8000_Mixed	21	20	1	4	3.5	C

58127500	Vegetable submarine sandwich, with fat free spread	8000_Mixed	21	20	1	4	3.5	C
27564220	Frankfurter or hot dog sandwich, meat and poultry, plain, on whole grain white bread	8000_Mixed	21	19	2	4	2	D
58400200	Rice soup, NFS	8000_Mixed	21	19	2	4	3	C
58404010	Chicken or turkey rice soup, canned, or ready-to-serve	8000_Mixed	21	19	2	4	3	C
32202130	Egg and steak on biscuit	8000_Mixed	21	18	3	3	1.5	E
35003000	Scrambled eggs, sausage, pancakes (frozen meal)	8000_Mixed	21	16	5	4	3	C
27520420	Midnight sandwich, with spread	8000_Mixed	21	14	7	3	3.5	D
55610200	Dumpling, fried, Puerto Rican style	8000_Mixed	21	14	7	3	2	D
58164540	Rice, white, with gravy, no added fat	8000_Mixed	21	9	12	1	3	C
27540150	Chicken fillet, breaded, fried, sandwich with lettuce, tomato and spread	8000_Mixed	20	23	-3	4	3	D
27540147	Chicken fillet sandwich, fried, from fast food, with cheese	8000_Mixed	20	22	-2	4	2	D
27540176	Chicken fillet sandwich, fried, on white bun; with cheese	8000_Mixed	20	22	-2	4	2	D
27540180	Chicken patty sandwich or biscuit	8000_Mixed	20	22	-2	4	2	D
28140740	Chicken patty or nuggets, boneless, breaded, with pasta and tomato sauce, fruit, dessert, frozen meal	8000_Mixed	20	22	-2	4	3	D
58106700	Pizza with meat and vegetables, from frozen, thin crust	8000_Mixed	20	22	-2	4	2	D
75607090	Mushroom soup, cream of, canned, reduced sodium, NS as to made with milk or water	8000_Mixed	20	22	-2	4	3.5	B
75607140	Mushroom soup, cream of, canned, reduced sodium, prepared with water	8000_Mixed	20	22	-2	4	3.5	B
75607150	Mushroom soup, cream of, canned, reduced sodium, undiluted	8000_Mixed	20	22	-2	4	3	C
27510160	Cheeseburger, from fast food, 1 small patty	8000_Mixed	20	21	-1	4	2	D
27510387	Double cheeseburger (McDonalds)	8000_Mixed	20	21	-1	4	2	D
27510388	McDouble (McDonalds)	8000_Mixed	20	21	-1	4	2	D
27510391	Double cheeseburger, 2 small patties, with condiments, on bun, from fast food / restaurant (Wendy's Double Stack)	8000_Mixed	20	21	-1	4	2	D
27510520	Hamburger, with mayonnaise or salad dressing, and tomato and/or catsup, on bun	8000_Mixed	20	21	-1	4	3.5	C
27510590	Hamburger, with mayonnaise or salad dressing, on bun	8000_Mixed	20	21	-1	4	3	D
27510700	Meatball and spaghetti sauce submarine sandwich	8000_Mixed	20	21	-1	4	3.5	D
58101457	Soft taco with chicken, from fast food	8000_Mixed	20	21	-1	4	3.5	D
58106725	Pizza with meat and vegetables, from restaurant or fast food, medium crust	8000_Mixed	20	21	-1	4	2.5	D
58106755	Pizza with meat and fruit, medium crust	8000_Mixed	20	21	-1	4	3	D
58131523	Ravioli, cheese-filled, with tomato sauce, canned	8000_Mixed	20	21	-1	4	3.5	C
58146433	Pasta with cream sauce, poultry, and added vegetables, ready-to-heat	8000_Mixed	20	21	-1	4	3	C
27510406	Double cheeseburger, 2 medium patties, with condiments, on bun, from fast food / restaurant	8000_Mixed	20	20	0	4	2	D
27510412	Double cheeseburger, 2 medium patties, with condiments, on bun, from fast food / restaurant (McDonald's Double Quarter Pounder with Cheese)	8000_Mixed	20	20	0	4	2	D
27510413	Double cheeseburger, 2 medium patties, with condiments, on bun, from fast food / restaurant (Wendy's 1/2 lb Double with cheese)	8000_Mixed	20	20	0	4	2	D

27510705	Chiliburger, with or without cheese, on bun	8000_Mixed	20	20	0	4	3.5	C
27564150	Frankfurter or hot dog sandwich, beef and pork, plain, on whole wheat bread	8000_Mixed	20	20	0	4	2	D
27564182	Frankfurter or hot dog sandwich, meat and poultry, plain, on whole wheat bun	8000_Mixed	20	20	0	4	2	D
27564243	Frankfurter or hot dog sandwich, chicken and/or turkey, plain, on whole grain white bun	8000_Mixed	20	20	0	4	2	D
27564290	Frankfurter or hot dog sandwich, chicken and/or turkey, plain, on multigrain bread	8000_Mixed	20	20	0	4	3.5	D
27510655	Double hamburger, on white bun, 2 medium patties	8000_Mixed	20	19	1	4	3	D
27510671	Double hamburger, from fast food, 2 medium patties	8000_Mixed	20	19	1	4	3	D
28110380	Salisbury steak with gravy, macaroni and cheese, frozen meal	8000_Mixed	20	19	1	4	3.5	C
28110640	Meatballs, Swedish, in sauce, with noodles, frozen meal	8000_Mixed	20	19	1	4	3	C
27510649	Double hamburger, on white bun, 2 small patties	8000_Mixed	20	18	2	4	3	D
58101300	Taco or tostada with beef, cheese and lettuce	8000_Mixed	20	18	2	4	3.5	C
14620320	Topping from meat pizza	8000_Mixed	20	17	3	4	1	E
27564503	Frankfurter or hot dog sandwich, with meatless chili, on whole grain white bun	8000_Mixed	20	17	3	4	3	D
27510110	Beef barbecue sandwich or Sloppy Joe, on bun	8000_Mixed	20	15	5	3	2	D
58124230	Pastry, meat / poultry-filled	8000_Mixed	20	10	10	2	2	D
14640002	Cheese sandwich, American cheese, on white bread, no spread	8000_Mixed	19	22	-3	4	1.5	E
58104745	Quesadilla with chicken, from fast food	8000_Mixed	19	22	-3	4	1.5	D
14640000	Cheese sandwich, NFS	8000_Mixed	19	21	-2	4	1.5	E
27540210	Chicken fillet wrap sandwich, fried, from fast food	8000_Mixed	19	21	-2	4	2	D
58106565	Pizza with pepperoni, stuffed crust	8000_Mixed	19	21	-2	4	2	D
58106630	Pizza with meat other than pepperoni, from restaurant or fast food, thick crust	8000_Mixed	19	21	-2	4	2	D
58106633	Pizza, with meat other than pepperoni, stuffed crust	8000_Mixed	19	21	-2	4	2	D
27510210	Cheeseburger, plain, on bun	8000_Mixed	19	20	-1	4	2	D
27510270	Double cheeseburger (2 patties), plain, on bun	8000_Mixed	19	20	-1	4	2	D
27510552	Whopper Jr (Burger King)	8000_Mixed	19	20	-1	4	3.5	C
58106734	Pizza with extra meat and extra vegetables, prepared from frozen, thick crust	8000_Mixed	19	20	-1	4	2.5	D
27510310	Cheeseburger with tomato and/or catsup, on bun	8000_Mixed	19	19	0	4	2	D
27510375	Double cheeseburger (2 patties, 1/4 lb meat each), with tomato and/or catsup, on bun	8000_Mixed	19	19	0	4	1.5	D
27564210	Frankfurter or hot dog sandwich, meat and poultry, plain, on whole wheat bread	8000_Mixed	19	19	0	4	2	D
58126290	Turnover, meat- and cheese-filled, lower in fat	8000_Mixed	19	19	0	4	3.5	D
58126410	Turnover, filled with egg, meat, and cheese, lower in fat	8000_Mixed	19	19	0	4	3.5	D
75608030	Onion soup, cream of, canned, undiluted	8000_Mixed	19	19	0	4	2.5	C
75657000	Vegetable broth, bouillon	8000_Mixed	19	19	0	4	3	C
27510445	Bacon cheeseburger, 1/3 lb meat, with tomato and/or catsup, on bun	8000_Mixed	19	18	1	4	2	D

27510680	Double hamburger (2 patties, 1/4 lb meat each), with tomato and/or catsup, on bun	8000_Mixed	19	18	1	4	3	D
28141600	Chicken a la king with rice, frozen meal	8000_Mixed	19	18	1	4	3	C
58126280	Turnover, chicken- or turkey-, and vegetable-filled, lower in fat	8000_Mixed	19	18	1	4	3.5	C
27564550	Frankfurter or hot dog sandwich, with meatless chili, on multigrain bread	8000_Mixed	19	17	2	4	2.5	D
27220150	Sausage and rice with mushroom sauce	8000_Mixed	19	10	9	3	3	C
58111200	Puffs, fried, crab meat and cream cheese filled	8000_Mixed	19	10	9	2	1.5	E
75607020	Mushroom soup, cream of, prepared with water	8000_Mixed	18	22	-4	4	3	C
27520370	Hot ham and cheese sandwich, on bun	8000_Mixed	18	20	-2	4	3	D
58106610	Pizza with meat other than pepperoni, from restaurant or fast food, NS as to type of crust	8000_Mixed	18	20	-2	4	2	D
58106625	Pizza with meat other than pepperoni, from restaurant or fast food, medium crust	8000_Mixed	18	20	-2	4	2	D
14710110	Cheddar cheese soup, canned, undiluted	8000_Mixed	18	19	-1	4	2	D
27510170	Cheeseburger (Burger King)	8000_Mixed	18	19	-1	4	3	D
27510290	Double cheeseburger (2 patties), plain, on double-decker bun	8000_Mixed	18	19	-1	4	2	D
27510420	Taco burger, on bun	8000_Mixed	18	19	-1	4	3.5	D
27540146	Chicken fillet sandwich, fried, from fast food	8000_Mixed	18	19	-1	4	3	D
27540160	Chicken fillet sandwich, NS as to fried or grilled, from fast food	8000_Mixed	18	19	-1	4	3	D
27540360	Turkey and bacon submarine sandwich, with lettuce, tomato and spread	8000_Mixed	18	19	-1	4	3.5	C
58106705	Pizza with meat and vegetables, from frozen, thick crust	8000_Mixed	18	19	-1	4	2	D
58146413	Pasta with cream sauce, meat, and added vegetables, ready-to-heat	8000_Mixed	18	19	-1	4	3	C
27510311	Cheeseburger, 1 oz meat, plain, on miniature bun	8000_Mixed	18	18	0	4	2	D
58101347	Soft taco with meat, from fast food	8000_Mixed	18	18	0	4	3	D
58101357	Soft taco with meat and sour cream, from fast food	8000_Mixed	18	18	0	4	3	D
58106740	Pizza with meat and fruit, NS as to type of crust	8000_Mixed	18	18	0	4	3	D
58147510	Flavored pasta	8000_Mixed	18	18	0	4	3.5	B
58302000	Macaroni and cheese, diet frozen meal	8000_Mixed	18	18	0	4	3.5	C
27510229	Quarter Pounder (McDonalds)	8000_Mixed	18	17	1	4	3.5	D
27510276	Bacon cheeseburger, 1 small patty, with condiments, on bun, from fast food / restaurant	8000_Mixed	18	17	1	4	2	D
27510605	Hamburger, from fast food, 1 large patty	8000_Mixed	18	17	1	4	3	D
27510606	Hamburger, 1 medium patty, with condiments, on bun, from fast food / restaurant	8000_Mixed	18	17	1	4	3.5	D
27510620	Hamburger, 1/4 lb meat, with tomato and/or catsup, on bun	8000_Mixed	18	17	1	4	3.5	D
27510635	Hamburger, on white bun, 1 large patty	8000_Mixed	18	17	1	4	3	D
27564470	Frankfurter or hot dog sandwich, with chili, on whole wheat bread	8000_Mixed	18	17	1	4	2.5	D
27564530	Frankfurter or hot dog sandwich, with meatless chili, on whole wheat bread	8000_Mixed	18	17	1	4	2.5	D
32202050	Egg, cheese, and sausage on biscuit	8000_Mixed	18	17	1	4	1.5	E
58134680	Tortellini, cheese-filled, no sauce	8000_Mixed	18	17	1	4	4	B

58145117	Macaroni or noodles with cheese, Easy Mac type	8000_Mixed	18	17	1	4	3.5	C
58304230	Ravioli, cheese-filled, with vegetable and fruit (frozen meal)	8000_Mixed	18	17	1	4	3.5	C
27564002	Frankfurter or hot dog sandwich, NFS, plain, on whole wheat bun	8000_Mixed	18	16	2	4	2	D
27564062	Frankfurter or hot dog sandwich, beef, plain, on whole wheat bun	8000_Mixed	18	16	2	4	2	D
27564480	Frankfurter or hot dog sandwich, with chili, on whole grain white bread	8000_Mixed	18	16	2	4	2.5	D
28140320	Chicken and noodles with vegetable, dessert, frozen meal	8000_Mixed	18	16	2	4	3.5	C
28345010	Chicken or turkey soup, cream of, canned, reduced sodium, NS as to made with milk or water	8000_Mixed	18	16	2	4	3.5	B
28345030	Chicken or turkey soup, cream of, canned, reduced sodium, made with water	8000_Mixed	18	16	2	4	3.5	B
58407010	Instant soup, noodle	8000_Mixed	18	16	2	4	3.5	B
28140910	Chicken, fried, with potatoes, vegetable, cornbread, dessert (frozen meal)	8000_Mixed	18	15	3	4	3.5	C
27560700	Sausage on biscuit, diet	8000_Mixed	18	6	12	3	3	D
58164570	Rice, white, with soy-based sauce, no added fat	8000_Mixed	18	6	12	1	3	C
75607000	Mushroom soup, NFS	8000_Mixed	17	21	-4	4	3	C
27520155	Bacon, lettuce, and tomato submarine sandwich, with spread	8000_Mixed	17	18	-1	4	4	C
27520340	Ham salad sandwich	8000_Mixed	17	18	-1	4	3	D
27560410	Puerto Rican sandwich	8000_Mixed	17	18	-1	4	1.5	E
28150000	Fish dinner, NFS (frozen meal)	8000_Mixed	17	18	-1	4	3	C
28150650	Fish, breaded, or fish sticks, with pasta, vegetable and dessert (frozen meal)	8000_Mixed	17	18	-1	4	3	C
28355430	Shrimp soup, cream of, prepared with water	8000_Mixed	17	18	-1	4	3	C
58106505	Pizza with meat, prepared from frozen, thick crust	8000_Mixed	17	18	-1	4	2	D
58146393	Pasta with cream sauce and added vegetables, ready-to-heat	8000_Mixed	17	18	-1	4	3	C
27510165	Cheeseburger, 1 small patty, with condiments, on bun, from fast food / restaurant	8000_Mixed	17	17	0	4	2	D
27510172	Cheeseburger (McDonalds)	8000_Mixed	17	17	0	4	2	D
27510173	Cheeseburger, 1 small patty, with condiments, on bun, from fast food / restaurant (Wendy's Kid's Cheeseburger)	8000_Mixed	17	17	0	4	2	D
27510174	Cheeseburger, 1 small patty, with condiments, on bun, from fast food / restaurant (Wendy's Jr. Cheeseburger)	8000_Mixed	17	17	0	4	2	D
27510573	Hamburger slider	8000_Mixed	17	17	0	4	3.5	D
27510661	Double hamburger, from fast food, 2 small patties	8000_Mixed	17	17	0	4	3.5	D
27541001	Turkey, ham, and roast beef club sandwich with cheese, lettuce, tomato, and spread	8000_Mixed	17	17	0	4	4	B
75607130	Mushroom soup, made from dry mix	8000_Mixed	17	17	0	4	3	C
27510521	Hamburger, NFS	8000_Mixed	17	16	1	4	3.5	D
27510601	Hamburger, from fast food, 1 medium patty	8000_Mixed	17	16	1	4	3.5	D
27510631	Hamburger, on white bun, 1 medium patty	8000_Mixed	17	16	1	4	3.5	D
27564030	Frankfurter or hot dog sandwich, NFS, plain, on whole wheat bread	8000_Mixed	17	16	1	4	2	D
27564090	Frankfurter or hot dog sandwich, beef, plain, on whole wheat bread	8000_Mixed	17	16	1	4	2	D

27564123	Frankfurter or hot dog sandwich, beef and pork, plain, on whole grain white bun	8000_Mixed	17	16	1	4	2	D
58305010	Mosticolli with meatballs, sauce, bread (frozen meal)	8000_Mixed	17	16	1	4	3	C
71801020	Potato soup, prepared with water	8000_Mixed	17	16	1	4	3	C
27510500	Hamburger, plain, on bun	8000_Mixed	17	15	2	4	3.5	D
27564040	Frankfurter or hot dog sandwich, NFS, plain, on whole grain white bread	8000_Mixed	17	15	2	4	2	D
27564100	Frankfurter or hot dog sandwich, beef, plain, on whole grain white bread	8000_Mixed	17	15	2	4	2	D
28340530	Chicken soup	8000_Mixed	17	15	2	4	3	C
27520110	Bacon sandwich, with spread	8000_Mixed	17	14	3	4	1.5	E
27560705	Sausage balls, made with biscuit mix and cheese	8000_Mixed	17	14	3	4	0.5	E
58101530	Soft taco with beef, cheese, lettuce, tomato and salsa	8000_Mixed	17	14	3	4	3	D
27520380	Ham and cheese on English muffin	8000_Mixed	17	11	6	3	1.5	D
27214600	Creamed dried beef on toast	8000_Mixed	17	7	10	2	2	D
27540145	Chicken fillet biscuit, from fast food	8000_Mixed	16	19	-3	4	1.5	E
27510506	Hamburger, 1 miniature patty, with condiments, on miniature bun, from fast food / restaurant	8000_Mixed	16	17	-1	4	3.5	D
27560500	Pepperoni and salami submarine sandwich, with lettuce, tomato and spread	8000_Mixed	16	17	-1	4	3	D
58106710	Pizza with meat and vegetables, NS as to type of crust	8000_Mixed	16	17	-1	4	2.5	D
58146423	Pasta with cream sauce and poultry, ready-to-heat	8000_Mixed	16	17	-1	4	2.5	C
27510530	Hamburger, 1/4 lb meat, plain, on bun	8000_Mixed	16	16	0	4	3	D
27510575	Hamburger, on white bun, 1 small patty	8000_Mixed	16	16	0	4	3	D
27510650	Double hamburger (2 patties), plain, on bun	8000_Mixed	16	16	0	4	3	D
27564170	Frankfurter or hot dog sandwich, beef and pork, plain, on multigrain bread	8000_Mixed	16	16	0	4	2	D
28110330	Salisbury steak with gravy, whipped potatoes, vegetable, dessert, frozen meal	8000_Mixed	16	16	0	4	3	C
58126160	Turnover, cheese-filled, tomato-based sauce	8000_Mixed	16	16	0	4	1.5	D
27513010	Roast beef sandwich	8000_Mixed	16	15	1	4	2	D
27517000	Hamburger wrap sandwich, from fast food	8000_Mixed	16	15	1	4	2	D
27564183	Frankfurter or hot dog sandwich, meat and poultry, plain, on whole grain white bun	8000_Mixed	16	15	1	4	2	D
27564230	Frankfurter or hot dog sandwich, meat and poultry, plain, on multigrain bread	8000_Mixed	16	15	1	4	2	D
58126270	Turnover, chicken- or turkey-, and cheese-filled, no gravy	8000_Mixed	16	15	1	4	3.5	C
27564443	Frankfurter or hot dog sandwich, with chili, on whole grain white bun	8000_Mixed	16	14	2	4	3	D
27564490	Frankfurter or hot dog sandwich, with chili, on multigrain bread	8000_Mixed	16	14	2	4	2.5	D
28141201	Teriyaki chicken with rice and vegetable, diet frozen meal	8000_Mixed	16	14	2	4	3.5	C
28340520	Chicken soup, canned, undiluted	8000_Mixed	16	14	2	4	3	C
32202025	Egg, cheese and ham on bagel	8000_Mixed	16	14	2	4	2	D
58403020	Chicken noodle soup, canned, undiluted	8000_Mixed	16	14	2	4	3	C

74604100	Tomato beef rice soup, prepared with water	8000_Mixed	16	14	2	4	3	C
28140920	Chicken, fried, with potatoes, vegetable, vegetable soup, dessert (frozen meal)	8000_Mixed	16	13	3	4	3.5	C
58127350	Croissant sandwich with bacon, egg, and cheese	8000_Mixed	16	13	3	4	2	D
32202010	Egg, cheese, and ham on English muffin	8000_Mixed	16	11	5	4	3	D
32202035	Egg, extra cheese, and extra sausage, on bun	8000_Mixed	16	11	5	4	1.5	D
32202070	Egg, cheese, and bacon on biscuit	8000_Mixed	16	11	5	4	1.5	D
32202080	Egg, cheese, and bacon on English muffin	8000_Mixed	16	11	5	4	3	D
58100017	Burrito, taco, or quesadilla with egg, potato, and breakfast meat, from fast food	8000_Mixed	16	11	5	4	2	D
32202030	Egg, cheese, and sausage on English muffin	8000_Mixed	16	9	7	4	2	D
27520360	Ham and cheese sandwich, on bun, with lettuce and spread	8000_Mixed	15	16	-1	4	2	D
27510510	Hamburger, with tomato and/or catsup, on bun	8000_Mixed	15	15	0	4	3	D
27510536	Hamburger, 1 small patty, with condiments, on bun, from fast food / restaurant	8000_Mixed	15	15	0	4	3	D
27510553	Hamburger (McDonalds)	8000_Mixed	15	15	0	4	3	D
27510555	Hamburger, 1 small patty, with condiments, on bun, from fast food / restaurant (Wendy's Jr. Hamburger)	8000_Mixed	15	15	0	4	3	D
58146403	Pasta with cream sauce and meat, ready-to-heat	8000_Mixed	15	15	0	4	2.5	C
58128000	Biscuit with gravy	8000_Mixed	15	14	1	3	2	D
58146200	Pasta, meat-filled, with gravy, canned	8000_Mixed	15	14	1	4	3	C
74603010	Tomato beef soup, prepared with water	8000_Mixed	15	14	1	4	3	C
74604010	Tomato beef noodle soup, prepared with water	8000_Mixed	15	14	1	4	3	C
27510450	Cheeseburger, 1/4 lb meat, with ham, on bun	8000_Mixed	15	13	2	4	1.5	D
27510540	Double hamburger (2 patties), with tomato and/or catsup, on bun	8000_Mixed	15	13	2	4	3	D
28120310	Pork with rice, vegetable, in soy-based sauce (diet frozen meal)	8000_Mixed	15	13	2	4	3.5	C
58101400	Soft taco with beef, cheese, and lettuce	8000_Mixed	15	13	2	4	3	D
58127330	Croissant sandwich with sausage, egg, and cheese	8000_Mixed	15	13	2	4	1.5	D
58134623	Tortellini, cheese-filled, meatless, with tomato sauce, canned	8000_Mixed	15	13	2	4	3.5	C
58306500	Chicken burritos, diet frozen meal	8000_Mixed	15	13	2	4	1.5	E
27564050	Frankfurter or hot dog sandwich, NFS, plain, on multigrain bread	8000_Mixed	15	12	3	4	2	D
27564110	Frankfurter or hot dog sandwich, beef, plain, on multigrain bread	8000_Mixed	15	12	3	4	2	D
14640300	Cheese spread sandwich	8000_Mixed	14	16	-2	4	1.5	D
41502000	Beans and franks, frozen dinner	8000_Mixed	14	14	0	4	3	C
27510501	Hamburger slider, from fast food	8000_Mixed	14	13	1	4	3.5	D
27510531	Hamburger, from fast food, 1 small patty	8000_Mixed	14	13	1	4	3.5	D
27510551	Hamburger (Burger King)	8000_Mixed	14	13	1	4	3.5	D
27520540	Ham and tomato club sandwich, with lettuce and spread	8000_Mixed	14	13	1	4	3	D
27510610	Hamburger, 1 oz meat, with tomato and/or catsup, on miniature bun	8000_Mixed	14	12	2	4	3.5	D
27515080	Steak sandwich, plain, on biscuit	8000_Mixed	14	12	2	3	1.5	E
27540350	Turkey submarine sandwich, with cheese, lettuce, tomato and spread	8000_Mixed	14	12	2	4	4	B

58100013	Burrito, taco, or quesadilla with egg and breakfast meat, from fast food	8000_Mixed	14	11	3	4	1.5	D
32202020	Egg, cheese, and ham on biscuit	8000_Mixed	14	9	5	4	1.5	E
58146383	Pasta with cream sauce, ready-to-heat	8000_Mixed	13	14	-1	4	2.5	D
58163310	Flavored rice mixture	8000_Mixed	13	14	-1	4	3	C
27510600	Hamburger, 1 oz meat, plain, on miniature bun	8000_Mixed	13	13	0	4	3.5	D
27520320	Ham and cheese sandwich, with lettuce and spread	8000_Mixed	13	13	0	4	2	D
27520390	Ham and cheese submarine sandwich, with lettuce, tomato and spread	8000_Mixed	13	12	1	4	3.5	C
27560000	Luncheon meat sandwich, NFS, with spread	8000_Mixed	13	12	1	4	3.5	C
27564003	Frankfurter or hot dog sandwich, NFS, plain, on whole grain white bun	8000_Mixed	13	12	1	4	1.5	E
27564063	Frankfurter or hot dog sandwich, beef, plain, on whole grain white bun	8000_Mixed	13	12	1	4	1.5	E
58163380	Flavored rice and pasta mixture	8000_Mixed	13	12	1	4	3.5	C
27560370	Frankfurter or hot dog with chili and cheese, on bun	8000_Mixed	13	10	3	4	1.5	E
58127290	Croissant sandwich with bacon and egg	8000_Mixed	13	9	4	4	1.5	E
58145160	Macaroni or noodles with cheese and frankfurters or hot dogs	8000_Mixed	13	9	4	4	2.5	C
58408010	Wonton soup	8000_Mixed	13	1	12	1	3	C
51182010	Bread stuffing	8000_Mixed	12	14	-2	4	2.5	C
27560120	Bologna and cheese sandwich, with spread	8000_Mixed	12	13	-1	4	1.5	E
27520350	Ham and cheese sandwich, with spread, grilled	8000_Mixed	12	11	1	4	1.5	E
27560910	Cold cut sumarine sandwich, with cheese, lettuce, tomato and spread	8000_Mixed	12	11	1	4	3	D
58145115	Macaroni or noodles with cheese, from boxed mix with already prepared cheese sauce	8000_Mixed	12	11	1	4	3	C
27347100	Chicken or turkey pot pie	8000_Mixed	12	10	2	4	3	C
27360050	Meat pie, NFS	8000_Mixed	12	10	2	4	3	C
58126130	Turnover, meat- and cheese-filled, no gravy	8000_Mixed	12	10	2	4	3	D
32202045	Egg, cheese, and steak on bagel	8000_Mixed	12	9	3	4	2	D
27564520	Frankfurter or hot dog sandwich, with meatless chili, on wheat bread	8000_Mixed	12	8	4	4	2.5	D
27560380	Pochito (frankfurter or hot dog and beef chili wrapped in tortilla)	8000_Mixed	12	6	6	4	2	D
58108050	Pizza rolls	8000_Mixed	11	12	-1	4	2	D
58407040	Instant soup, rice	8000_Mixed	11	9	2	4	3	C
27564501	Frankfurter or hot dog sandwich, with meatless chili, on wheat bun	8000_Mixed	11	8	3	4	3	D
58128250	Dressing with meat and vegetables	8000_Mixed	11	8	3	4	2	D
27560360	Frankfurter or hot dog, with chili, on bun	8000_Mixed	11	6	5	4	2	D
32202060	Egg and sausage on biscuit	8000_Mixed	11	6	5	4	1.5	D
32202075	Egg, cheese, and bacon griddle cake sandwich	8000_Mixed	11	6	5	4	1.5	D
58151110	Sushi, no vegetables, no seafood (no fish or shellfish)	8000_Mixed	11	1	10	1	3	C
58126300	Turnover, meat- and cheese-filled, tomato-based sauce, lower in fat	8000_Mixed	10	10	0	4	3	C
58163330	Flavored rice mixture with cheese	8000_Mixed	10	8	2	4	3	C

55701000	Cake made with glutinous rice	8000_Mixed	10	7	3	2	2	D
58127310	Croissant sandwich with ham, egg, and cheese	8000_Mixed	10	7	3	4	1.5	D
27564380	Frankfurter or hot dog sandwich, fat free, plain, on wheat bread	8000_Mixed	10	6	4	4	3.5	D
32202090	Egg and bacon on biscuit	8000_Mixed	10	6	4	4	1.5	D
27560310	Corny dog, with chili, on bun	8000_Mixed	10	2	8	3	2	D
58201000	Jelly sandwich, regular jelly, NFS	8000_Mixed	9	11	-2	4	2.5	D
58201005	Jelly sandwich, regular jelly, on white bread	8000_Mixed	9	11	-2	4	2.5	D
27520300	Ham sandwich, with spread	8000_Mixed	9	10	-1	4	3	D
58126400	Turnover, filled with egg, meat and cheese	8000_Mixed	9	9	0	4	2.5	D
27520310	Ham sandwich with lettuce and spread	8000_Mixed	9	8	1	4	3.5	D
27564361	Frankfurter or hot dog sandwich, fat free, plain, on wheat bun	8000_Mixed	9	7	2	4	3.5	C
27564504	Frankfurter or hot dog sandwich, with meatless chili, on multigrain bun	8000_Mixed	9	5	4	4	3	D
27564510	Frankfurter or hot dog sandwich, with meatless chili, on white bread	8000_Mixed	9	3	6	4	2	D
32202055	Egg, cheese, and sausage griddle cake sandwich	8000_Mixed	9	3	6	4	1.5	D
27563010	Meat spread or potted meat sandwich	8000_Mixed	8	8	0	4	2	D
27564241	Frankfurter or hot dog sandwich, chicken and/or turkey, plain, on wheat bun	8000_Mixed	8	6	2	4	3	D
27120210	Frankfurter or hot dog, with chili, no bun	8000_Mixed	8	5	3	4	2	D
27420470	Sausage and peppers, no sauce	8000_Mixed	8	4	4	4	2	D
27564260	Frankfurter or hot dog sandwich, chicken and/or turkey, plain, on wheat bread	8000_Mixed	8	4	4	4	2	D
27211400	Corned beef hash	8000_Mixed	8	3	5	4	3	D
27214500	Corned beef patty	8000_Mixed	8	3	5	4	3	D
27260110	Hash, NS as to type of meat	8000_Mixed	8	3	5	4	3	D
27510910	Corned beef sandwich	8000_Mixed	8	2	6	4	3.5	D
58201035	Jelly sandwich, reduced sugar jelly, on white bread	8000_Mixed	7	6	1	4	3	C
27564301	Frankfurter or hot dog sandwich, reduced fat or light, plain, on wheat bun	8000_Mixed	7	5	2	4	3.5	D
27564441	Frankfurter or hot dog sandwich, with chili, on wheat bun	8000_Mixed	7	5	2	4	3	D
27564442	Frankfurter or hot dog sandwich, with chili, on whole wheat bun	8000_Mixed	7	5	2	4	3	D
27564460	Frankfurter or hot dog sandwich, with chili, on wheat bread	8000_Mixed	7	4	3	4	2.5	D
28321130	Bacon soup, cream of, prepared with water	8000_Mixed	7	4	3	4	3	C
27564320	Frankfurter or hot dog sandwich, reduced fat or light, plain, on wheat bread	8000_Mixed	7	3	4	4	3.5	D
27564500	Frankfurter or hot dog sandwich, with meatless chili, on white bun	8000_Mixed	7	3	4	4	3	D
58310210	Sausage and french toast, frozen meal	8000_Mixed	7	2	5	4	3	D
32202110	Egg and ham on biscuit	8000_Mixed	7	1	6	4	1.5	D
27560510	Salami sandwich, with spread	8000_Mixed	6	5	1	4	1.5	E
27560720	Sausage and spaghetti sauce sandwich	8000_Mixed	6	4	2	4	2	D
27564370	Frankfurter or hot dog sandwich, fat free, plain, on white bread	8000_Mixed	6	3	3	4	3	D
27564444	Frankfurter or hot dog sandwich, with chili, on multigrain bun	8000_Mixed	6	3	3	4	3	D

28345110	Chicken or turkey soup, cream of, NS as to prepared with milk or water	8000_Mixed	6	3	3	4	3	C
28345130	Chicken or turkey soup, cream of, prepared with water	8000_Mixed	6	3	3	4	3	C
28345140	Chicken or turkey soup, cream of, canned, undiluted	8000_Mixed	6	3	3	4	2.5	C
58400100	Noodle soup, NFS	8000_Mixed	6	3	3	4	3	C
27120110	Sausage with tomato-based sauce	8000_Mixed	6	2	4	4	1.5	D
27511010	Pastrami sandwich	8000_Mixed	6	1	5	4	3	D
27564121	Frankfurter or hot dog sandwich, beef and pork, plain, on wheat bun	8000_Mixed	5	2	3	4	2	D
27564181	Frankfurter or hot dog sandwich, meat and poultry, plain, on wheat bun	8000_Mixed	5	2	3	4	2	D
58407030	Soup, mostly noodles	8000_Mixed	5	2	3	4	3	C
58407035	Soup, mostly noodles, reduced sodium	8000_Mixed	5	2	3	4	3.5	B
58407050	Instant soup, noodle with egg, shrimp or chicken	8000_Mixed	5	2	3	4	3	C
27564450	Frankfurter or hot dog sandwich, with chili, on white bread	8000_Mixed	5	1	4	4	2	D
27564140	Frankfurter or hot dog sandwich, beef and pork, plain, on wheat bread	8000_Mixed	4	2	2	4	1.5	E
27560670	Sausage and cheese on English muffin	8000_Mixed	4	1	3	4	1.5	E
27564001	Frankfurter or hot dog sandwich, NFS, plain, on wheat bun	8000_Mixed	4	1	3	4	1.5	E
27564061	Frankfurter or hot dog sandwich, beef, plain, on wheat bun	8000_Mixed	4	1	3	4	1.5	E
27564200	Frankfurter or hot dog sandwich, meat and poultry, plain, on wheat bread	8000_Mixed	4	1	3	4	2	E
27564364	Frankfurter or hot dog sandwich, fat free, plain, on multigrain bun	8000_Mixed	4	1	3	4	3.5	C
58127270	Croissant sandwich with sausage and egg	8000_Mixed	4	1	3	4	1.5	E
27500050	Sandwich, NFS	8000_Mixed	3	1	2	4	2	D
27500100	Meat sandwich, NFS	8000_Mixed	3	1	2	4	2	D
27560110	Bologna sandwich, with spread	8000_Mixed	3	1	2	4	2	D
27560330	Frankfurter or hot dog, with cheese, plain, on bun	8000_Mixed	3	1	2	4	1.5	D
27560400	Chicken frankfurter or hot dog, plain, on bun	8000_Mixed	3	1	2	4	3	D
27560710	Sausage sandwich	8000_Mixed	3	1	2	4	2	D
27564244	Frankfurter or hot dog sandwich, chicken and/or turkey, plain, on multigrain bun	8000_Mixed	3	1	2	4	3	D
27564440	Frankfurter or hot dog sandwich, with chili, on white bun	8000_Mixed	3	1	2	4	3	D
28111010	Corned beef hash with apple slices, vegetable (frozen meal)	8000_Mixed	3	1	2	4	2.5	D
32202120	Egg, cheese and sausage on bagel	8000_Mixed	3	1	2	4	2	D
75607030	Mushroom soup, canned, undiluted	8000_Mixed	3	1	2	4	2.5	C
27420040	Frankfurters or hot dogs and sauerkraut	8000_Mixed	2	1	1	4	1.5	D
27520170	Bacon on biscuit	8000_Mixed	2	1	1	4	1	E
27560650	Sausage on biscuit	8000_Mixed	2	1	1	4	1.5	E
27564020	Frankfurter or hot dog sandwich, NFS, plain, on wheat bread	8000_Mixed	2	1	1	4	1.5	E
27564080	Frankfurter or hot dog sandwich, beef, plain, on wheat bread	8000_Mixed	2	1	1	4	1.5	E
27564124	Frankfurter or hot dog sandwich, beef and pork, plain, on multigrain bun	8000_Mixed	2	1	1	4	2	D

27564184	Frankfurter or hot dog sandwich, meat and poultry, plain, on multigrain bun	8000_Mixed	2	1	1	4	2	D
27564250	Frankfurter or hot dog sandwich, chicken and/or turkey, plain, on white bread	8000_Mixed	2	1	1	4	2	D
27564304	Frankfurter or hot dog sandwich, reduced fat or light, plain, on multigrain bun	8000_Mixed	2	1	1	4	3	D
27120250	Frankfurters or hot dogs with tomato-based sauce	8000_Mixed	1	1	0	4	1.5	E
27520250	Ham on biscuit	8000_Mixed	1	1	0	4	1	E
27560300	Corn dog, frankfurter or hot dog with cornbread coating	8000_Mixed	1	1	0	4	2	D
27560320	Frankfurter or hot dog, plain, on bun	8000_Mixed	1	1	0	4	1.5	E
27560340	Frankfurter or hot dog, with catsup and/or mustard, on bun	8000_Mixed	1	1	0	4	1.5	E
27560350	Pig in a blanket, frankfurter or hot dog wrapped in dough	8000_Mixed	1	1	0	4	1.5	E
27560660	Sausage griddle cake sandwich	8000_Mixed	1	1	0	4	1.5	E
27564000	Frankfurter or hot dog sandwich, NFS, plain, on white bun	8000_Mixed	1	1	0	4	1.5	E
27564004	Frankfurter or hot dog sandwich, NFS, plain, on multigrain bun	8000_Mixed	1	1	0	4	1.5	E
27564010	Frankfurter or hot dog sandwich, NFS, plain, on white bread	8000_Mixed	1	1	0	4	1.5	E
27564060	Frankfurter or hot dog sandwich, beef, plain, on white bun	8000_Mixed	1	1	0	4	1.5	E
27564064	Frankfurter or hot dog sandwich, beef, plain, on multigrain bun	8000_Mixed	1	1	0	4	1.5	E
27564070	Frankfurter or hot dog sandwich, beef, plain, on white bread	8000_Mixed	1	1	0	4	1.5	E
27564120	Frankfurter or hot dog sandwich, beef and pork, plain, on white bun	8000_Mixed	1	1	0	4	2	D
27564130	Frankfurter or hot dog sandwich, beef and pork, plain, on white bread	8000_Mixed	1	1	0	4	1.5	E
27564180	Frankfurter or hot dog sandwich, meat and poultry, plain, on white bun	8000_Mixed	1	1	0	4	2	D
27564190	Frankfurter or hot dog sandwich, meat and poultry, plain, on white bread	8000_Mixed	1	1	0	4	1.5	E
27564240	Frankfurter or hot dog sandwich, chicken and/or turkey, plain, on white bun	8000_Mixed	1	1	0	4	2	D
27564300	Frankfurter or hot dog sandwich, reduced fat or light, plain, on white bun	8000_Mixed	1	1	0	4	3	D
27564310	Frankfurter or hot dog sandwich, reduced fat or light, plain, on white bread	8000_Mixed	1	1	0	4	3	D
27564360	Frankfurter or hot dog sandwich, fat free, plain, on white bun	8000_Mixed	1	1	0	4	3.5	C
27564418	Frankfurter or hot dog sandwich, reduced sodium	8000_Mixed	1	1	0	4	2	D
58127210	Croissant sandwich, filled with ham and cheese	8000_Mixed	1	1	0	4	1.5	E
58310310	Pancakes and sausage, frozen meal	8000_Mixed	1	1	0	4	2	D
75121000	Pepper, hot chili, raw	8600_SauceCondiment	100	100	0	1	4.5	A
75226700	Pimiento	8600_SauceCondiment	100	100	0	3	4	A
74402110	Salsa, pico de gallo	8600_SauceCondiment	100	96	4	1	3.5	C
74404060	Spaghetti sauce, fat free	8600_SauceCondiment	99	99	0	3	3.5	C
75412030	Eggplant dip	8600_SauceCondiment	98	100	-2	1	4.5	A
75511010	Hot pepper sauce	8600_SauceCondiment	98	99	-1	3	2.5	C
28522050	Mole verde sauce	8600_SauceCondiment	96	97	-1	1	3.5	B
27150155	Spaghetti sauce with seafood and added vegetables	8600_SauceCondiment	96	91	5	3	4	B

74402310	Green tomato-chile sauce, raw (Salsa de tomate verde cruda)	8600_SauceCondiment	95	91	4	1	3.5	B
75226103	Peppers, hot, cooked, from canned, fat not added in cooking	8600_SauceCondiment	94	94	0	3	2.5	D
74420110	Puerto Rican seasoning without ham and tomato sauce	8600_SauceCondiment	93	100	-7	2	4	A
75226111	Hot peppers, cooked	8600_SauceCondiment	93	96	-3	2	4.5	A
75226100	Peppers, hot, cooked, NS as to form, fat not added in cooking	8600_SauceCondiment	92	90	2	1	4	A
75226101	Peppers, hot, cooked, from fresh, fat not added in cooking	8600_SauceCondiment	92	90	2	1	4	A
75226102	Peppers, hot, cooked, from frozen, fat not added in cooking	8600_SauceCondiment	92	90	2	1	4	A
41420200	Natto	8600_SauceCondiment	91	97	-6	3	5	A
63408200	Guacamole with tomatoes and chili peppers	8600_SauceCondiment	90	88	2	1	4.5	A
63408010	Guacamole with tomatoes	8600_SauceCondiment	89	88	1	1	4.5	A
63408010	Guacamole with tomatoes	8600_SauceCondiment	88	86	2	1	4.5	A
27150151	Spaghetti sauce with seafood	8600_SauceCondiment	88	85	3	4	3.5	C
63409010	Guacamole, NFS	8600_SauceCondiment	86	83	3	1	4.5	A
74406060	Buffalo sauce	8600_SauceCondiment	84	90	-6	4	0.5	E
41205070	Hummus, plain	8600_SauceCondiment	84	83	1	1	4	C
41205075	Hummus, flavored	8600_SauceCondiment	84	83	1	1	4	C
74402210	Taco sauce	8600_SauceCondiment	83	91	-8	4	3	C
75111500	Garlic, raw	8600_SauceCondiment	82	80	2	1	4.5	A
61204200	Lemon juice, 100%, canned or bottled	8600_SauceCondiment	81	89	-8	1	3.5	A
27150200	Oyster sauce	8600_SauceCondiment	80	72	8	2	3.5	C
61204600	Lemon juice, frozen	8600_SauceCondiment	79	91	-12	1	3.5	A
74404020	Spaghetti sauce with added vegetables	8600_SauceCondiment	79	87	-8	4	3.5	C
74402300	Salsa made with fruit	8600_SauceCondiment	78	80	-2	4	3.5	C
27141035	Spaghetti sauce with poultry and added vegetables	8600_SauceCondiment	78	77	1	2	4	B
61204000	Lemon juice, 100%, NS as to form	8600_SauceCondiment	77	88	-11	1	3.5	A
61204010	Lemon juice, 100%, freshly squeezed	8600_SauceCondiment	77	88	-11	1	3.5	A
74403050	Tomato sauce, low sodium	8600_SauceCondiment	77	88	-11	4	4	A
75226090	Peppers, hot, cooked, NS as to form, NS as to fat added in cooking	8600_SauceCondiment	77	76	1	2	4	A
75226091	Peppers, hot, cooked, from fresh, NS as to fat added in cooking	8600_SauceCondiment	77	76	1	2	4	A
75226110	Peppers, hot, cooked, NS as to form, fat added in cooking, NS as to type of fat	8600_SauceCondiment	77	76	1	2	4	A
75226112	Peppers, hot, cooked, from frozen, fat added in cooking, NS as to type of fat	8600_SauceCondiment	77	76	1	2	4	A
61207000	Lime juice, 100%, NS as to form	8600_SauceCondiment	76	87	-11	1	3.5	A
61207010	Lime juice, 100%, freshly squeezed	8600_SauceCondiment	76	87	-11	1	3.5	A
75226093	Peppers, hot, cooked, from canned, NS as to fat added in cooking	8600_SauceCondiment	76	78	-2	3	2.5	D
75226113	Peppers, hot, cooked, from canned, fat added in cooking, NS as to type of fat	8600_SauceCondiment	76	78	-2	3	2.5	D
74404050	Spaghetti sauce, reduced sodium	8600_SauceCondiment	75	87	-12	4	4	A
27141030	Spaghetti sauce with poultry	8600_SauceCondiment	75	72	3	3	3.5	C
74402100	Salsa, NFS	8600_SauceCondiment	72	76	-4	4	3.5	C

74402150	Salsa, red, commercially-prepared	8600_SauceCondiment	72	76	-4	4	3.5	C
74402200	Salsa, red, homemade	8600_SauceCondiment	72	75	-3	4	3.5	C
11440060	Tzatziki dip	8600_SauceCondiment	72	72	0	1	3.5	C
75506010	Mustard	8600_SauceCondiment	72	70	2	3	3	D
61207200	Lime juice, 100%, canned or bottled	8600_SauceCondiment	71	81	-10	1	3.5	A
81312100	Curry sauce	8600_SauceCondiment	71	76	-5	2	3	C
12350110	Spinach and artichoke dip	8600_SauceCondiment	69	77	-8	3	3.5	C
41420110	Miso	8600_SauceCondiment	67	66	1	3	0.5	E
75217400	Garlic, cooked	8600_SauceCondiment	67	63	4	1	4.5	A
11440050	Spinach dip, yogurt based	8600_SauceCondiment	66	71	-5	3	3	C
81302070	Pesto sauce	8600_SauceCondiment	66	66	0	2	1	E
74404010	Spaghetti sauce	8600_SauceCondiment	65	74	-9	4	3.5	C
74403010	Tomato sauce	8600_SauceCondiment	65	72	-7	4	3.5	C
11440030	Onion dip, yogurt based	8600_SauceCondiment	65	69	-4	3	3	C
74404030	Spaghetti sauce with meat, canned, no extra meat added	8600_SauceCondiment	65	65	0	3	3.5	C
27162060	Spaghetti sauce with meat and added vegetables	8600_SauceCondiment	65	62	3	2	3.5	C
11440040	Ranch dip, yogurt based	8600_SauceCondiment	63	68	-5	3	2.5	D
11440070	Vegetable dip, yogurt based	8600_SauceCondiment	63	67	-4	3	2.5	D
75236500	Yeast extract spread	8600_SauceCondiment	63	66	-3	4	0.5	D
27162040	Spaghetti sauce with meat	8600_SauceCondiment	63	59	4	2	3.5	C
11440020	Dill dip, yogurt based	8600_SauceCondiment	62	67	-5	3	2.5	D
27111050	Spaghetti sauce with beef or meat other than lamb or mutton, homemade	8600_SauceCondiment	62	60	2	2	3.5	C
41420300	Soy sauce	8600_SauceCondiment	62	58	4	3	0.5	E
27130040	Spaghetti sauce with lamb or mutton, homemade	8600_SauceCondiment	61	60	1	2	3.5	C
27162050	Spaghetti sauce with combination of meats, homemade	8600_SauceCondiment	61	59	2	2	3.5	C
11440010	Chipotle dip, yogurt based	8600_SauceCondiment	60	65	-5	3	2.5	D
41420350	Soy sauce, reduced sodium	8600_SauceCondiment	60	57	3	3	0.5	E
74410110	Puerto Rican seasoning with ham	8600_SauceCondiment	60	57	3	2	3	C
28510010	Gravy or sauce, poultry-based from Puerto Rican-style chicken fricasse	8600_SauceCondiment	59	57	2	2	3	C
74402350	Salsa verde or salsa, green	8600_SauceCondiment	58	60	-2	4	3	C
74402260	Enchilada sauce, green	8600_SauceCondiment	57	59	-2	4	3	C
41205055	Layer dip	8600_SauceCondiment	57	55	2	2	3	C
41205050	Bean dip, made with refried beans	8600_SauceCondiment	56	61	-5	4	4	B
75503090	Horseradish	8600_SauceCondiment	56	57	-1	4	3.5	C
12350100	Spinach dip	8600_SauceCondiment	53	59	-6	3	2.5	C
41205100	Black bean sauce	8600_SauceCondiment	51	53	-2	2	0.5	E
74404090	Vodka sauce with tomatoes and cream	8600_SauceCondiment	51	52	-1	3	3	C
27150190	Lobster sauce	8600_SauceCondiment	50	45	5	2	2	D

42204050	Peanut sauce	8600_SauceCondiment	49	49	0	3	1	E
74402250	Enchilada sauce, red	8600_SauceCondiment	48	53	-5	4	3	C
14620200	Cheese dip	8600_SauceCondiment	47	47	0	2	2.5	D
14650100	Cheese sauce	8600_SauceCondiment	47	47	0	2	2.5	D
14630300	Welsh rarebit	8600_SauceCondiment	47	44	3	3	3	D
28500050	Gravy, giblet	8600_SauceCondiment	47	41	6	3	3.5	C
12350110	Spinach and artichoke dip	8600_SauceCondiment	46	51	-5	3	1.5	C
14620130	Seafood dip	8600_SauceCondiment	44	44	0	3	1.5	E
14650195	Alfredo sauce with seafood and added vegetables	8600_SauceCondiment	43	33	10	3	2.5	D
41420100	Miso sauce	8600_SauceCondiment	42	51	-9	4	1	E
12350240	Spinach dip, regular	8600_SauceCondiment	42	46	-4	3	1.5	E
14620110	Artichoke dip	8600_SauceCondiment	42	46	-4	3	1.5	E
89901050	Tomato sauce, for use with vegetables	8600_SauceCondiment	41	46	-5	2	2	D
12350020	Dip, sour cream base, reduced calorie	8600_SauceCondiment	41	44	-3	3	2	D
81302030	Orange sauce (for duck)	8600_SauceCondiment	41	41	0	2	3	C
74415110	Puerto Rican seasoning with ham and tomato sauce	8600_SauceCondiment	41	34	7	3	3	C
12350010	Dip, NFS	8600_SauceCondiment	40	45	-5	4	1	E
12350230	Ranch dip, regular	8600_SauceCondiment	40	45	-5	4	1	E
12350000	Dip, sour cream base	8600_SauceCondiment	40	41	-1	3	1.5	D
14650150	Cheese sauce made with lowfat cheese	8600_SauceCondiment	40	34	6	2	2.5	D
64401000	Vinegar	8600_SauceCondiment	40	33	7	2	3.5	A
83110010	Mayonnaise-type salad dressing, cholesterol-free	8600_SauceCondiment	39	48	-9	4	1	E
83107000	Mayonnaise, regular	8600_SauceCondiment	39	47	-8	4	0.5	E
83107200	Mayonnaise, made with tofu	8600_SauceCondiment	39	47	-8	4	2	D
12350220	Onion dip, regular	8600_SauceCondiment	39	43	-4	3	1.5	D
75506100	Honey mustard dip	8600_SauceCondiment	39	41	-2	2	2	D
14620120	Shrimp dip, cream cheese base	8600_SauceCondiment	39	28	11	3	1.5	D
81308100	Fry sauce	8600_SauceCondiment	38	48	-10	4	1.5	E
12350210	Dill dip, regular	8600_SauceCondiment	38	43	-5	4	1	E
12350250	Vegetable dip, regular	8600_SauceCondiment	38	43	-5	4	1	E
83204030	Mayonnaise, reduced fat, with olive oil	8600_SauceCondiment	38	43	-5	4	1.5	D
14630100	Cheese fondue	8600_SauceCondiment	38	34	4	3	1.5	D
42204100	Gravy, vegetarian	8600_SauceCondiment	38	33	5	2	3	C
83100200	Salad dressing, NFS, for sandwiches	8600_SauceCondiment	37	46	-9	4	1	E
12350200	Chipotle dip, regular	8600_SauceCondiment	37	41	-4	4	1	E
13411000	White sauce or gravy	8600_SauceCondiment	37	32	5	2	3	C
28500150	Gravy, redeye	8600_SauceCondiment	37	32	5	3	3.5	B
81330210	Adobo fresco	8600_SauceCondiment	37	30	7	1	0.5	E
13412000	Milk gravy, quick gravy	8600_SauceCondiment	37	29	8	1	3	C

14620150	Cheese dip with chili pepper	8600_SauceCondiment	35	40	-5	4	2.5	D
74405010	Tomato relish	8600_SauceCondiment	35	38	-3	2	3.5	C
81302060	Horseradish sauce	8600_SauceCondiment	33	42	-9	4	1	E
74402010	Tomato chili sauce	8600_SauceCondiment	33	35	-2	4	2	D
81302050	Tartar sauce	8600_SauceCondiment	32	39	-7	4	2	D
27150210	Fish sauce	8600_SauceCondiment	32	32	0	4	0.5	E
14650185	Alfredo sauce with poultry and added vegetables	8600_SauceCondiment	32	24	8	3	2.5	D
12350245	Spinach dip, light	8600_SauceCondiment	31	36	-5	4	2.5	D
28500030	Gravy, poultry, low sodium	8600_SauceCondiment	31	32	-1	4	4	A
28500060	Gravy, beef or meat, low sodium	8600_SauceCondiment	31	32	-1	4	4	A
14650190	Alfredo sauce with seafood	8600_SauceCondiment	31	21	10	3	2	D
14650175	Alfredo sauce with meat and added vegetables	8600_SauceCondiment	29	21	8	3	2.5	D
83108100	Mayonnaise, imitation, cholesterol free	8600_SauceCondiment	27	34	-7	4	1.5	D
28510020	Gravy, meat-based, from Puerto-Rican style stuffed pot roast	8600_SauceCondiment	27	19	8	2	2	D
83204000	Mayonnaise, light	8600_SauceCondiment	26	34	-8	4	2	D
27150200	Oyster sauce	8600_SauceCondiment	26	26	0	4	0.5	C
14650180	Alfredo sauce with poultry	8600_SauceCondiment	26	17	9	3	2	D
28500080	Gravy, poultry, home recipe	8600_SauceCondiment	26	16	10	2	2.5	C
28510030	Gravy, meat-based, from Puerto-Rican style beef stew	8600_SauceCondiment	26	16	10	2	2.5	C
12350215	Dill dip, light	8600_SauceCondiment	25	29	-4	4	2	D
12350235	Ranch dip, light	8600_SauceCondiment	25	29	-4	4	2	D
12350255	Vegetable dip, light	8600_SauceCondiment	25	29	-4	4	2	D
55502000	Flour and water gravy	8600_SauceCondiment	25	11	14	1	3.5	B
83204060	Mayonnaise-type salad dressing, low-calorie or diet, cholesterol-free	8600_SauceCondiment	24	31	-7	4	1.5	D
12350225	Onion dip, light	8600_SauceCondiment	24	27	-3	4	2.5	D
75534550	Wasabi paste	8600_SauceCondiment	24	23	1	4	0.5	E
28500020	Gravy, meat, with fruit	8600_SauceCondiment	24	21	3	3	3.5	C
83204020	Mayonnaise, reduced calorie or diet, cholesterol-free	8600_SauceCondiment	23	31	-8	4	1.5	D
14620100	Dip, cream cheese base	8600_SauceCondiment	23	19	4	3	0.5	E
14650170	Alfredo sauce with meat	8600_SauceCondiment	23	14	9	3	2	D
81302010	Hollandaise sauce	8600_SauceCondiment	23	9	14	2	0.5	E
81302020	Bernaise sauce	8600_SauceCondiment	23	9	14	2	0.5	E
74401110	Ketchup, reduced sodium	8600_SauceCondiment	22	37	-15	4	3.5	B
89901030	Gravy, for use with vegetables	8600_SauceCondiment	21	12	9	3	2.5	D
28500070	Gravy, beef or meat, home recipe	8600_SauceCondiment	21	11	10	2	2.5	C
83110000	Mayonnaise-type salad dressing	8600_SauceCondiment	20	27	-7	4	2	D
83108000	Vegan mayonnaise	8600_SauceCondiment	20	24	-4	4	2.5	D
74406100	Steak sauce	8600_SauceCondiment	20	23	-3	4	1.5	E

14650165	Alfredo sauce with added vegetables	8600_SauceCondiment	20	15	5	3	2.5	D
12350205	Chipotle dip, light	8600_SauceCondiment	18	22	-4	4	2	D
43103100	Sesame sauce	8600_SauceCondiment	18	20	-2	2	2	D
28500010	Gravy, meat or poultry, with wine	8600_SauceCondiment	16	15	1	4	3.5	C
81301000	Garlic sauce	8600_SauceCondiment	16	5	11	2	0.5	E
81301020	Lemon-butter sauce	8600_SauceCondiment	16	5	11	2	0.5	E
74406500	Cocktail sauce	8600_SauceCondiment	15	16	-1	4	2	D
28500040	Gravy, beef	8600_SauceCondiment	15	13	2	4	3	C
28501010	Gravy, beef, fat free	8600_SauceCondiment	15	13	2	4	3	C
28520010	Gravy, NFS	8600_SauceCondiment	15	13	2	4	3	C
74403000	Hot Thai sauce	8600_SauceCondiment	13	17	-4	2	0.5	E
28522000	Mole sauce	8600_SauceCondiment	12	16	-4	4	3	C
81312000	Tartar sauce, reduced fat/calorie	8600_SauceCondiment	11	17	-6	4	2.5	D
83204050	Mayonnaise-type salad dressing, light	8600_SauceCondiment	11	17	-6	4	2	D
81302040	Sandwich spread	8600_SauceCondiment	11	14	-3	4	1	E
14650160	Alfredo sauce	8600_SauceCondiment	11	5	6	4	1.5	D
28520000	Gravy, made with soy sauce	8600_SauceCondiment	10	5	5	2	2.5	D
89901010	Cream sauce, for use with vegetables	8600_SauceCondiment	9	3	6	4	1	E
89901040	Soy based sauce, for use with vegetables	8600_SauceCondiment	8	3	5	2	2	D
83300700	Mayonnaise, fat free	8600_SauceCondiment	7	9	-2	4	2.5	D
41420250	Hoisin sauce	8600_SauceCondiment	6	11	-5	4	1	E
91361070	Plum sauce, Asian style	8600_SauceCondiment	6	7	-1	2	1.5	E
28500000	Gravy, poultry	8600_SauceCondiment	5	2	3	4	3	C
28501110	Gravy, poultry, fat free	8600_SauceCondiment	5	2	3	4	3	C
41420450	Worcestershire sauce	8600_SauceCondiment	3	10	-7	4	1.5	E
83203250	Mayonnaise-type salad dressing, fat-free	8600_SauceCondiment	3	9	-6	4	2.5	D
63409020	Chutney	8600_SauceCondiment	3	4	-1	3	1.5	E
74401010	Ketchup	8600_SauceCondiment	2	13	-11	4	2.5	D
74406050	Barbecue sauce, reduced sodium	8600_SauceCondiment	2	12	-10	4	2.5	D
41420410	Teriyaki sauce, reduced sodium	8600_SauceCondiment	1	6	-5	4	1	E
41420400	Teriyaki sauce	8600_SauceCondiment	1	5	-4	4	0.5	E
28500100	Gravy, mushroom	8600_SauceCondiment	1	1	0	4	3	C
74406010	Barbecue sauce	8600_SauceCondiment	1	1	0	4	1	E
91361010	Sweet and sour sauce	8600_SauceCondiment	1	1	0	4	2.5	D
91361050	Duck sauce	8600_SauceCondiment	1	1	0	4	1.5	E
11830150	Cocoa powder, not reconstituted	9000_SavorySweet	91	84	7	2	5	A
91511090	Gelatin dessert, dietetic, with fruits and vegetables, sweetened with low calorie sweetener	9000_SavorySweet	89	95	-6	1	3.5	A
91511100	Gelatin salad, dietetic, with vegetables, sweetened with low calorie sweetener	9000_SavorySweet	86	84	2	2	3.5	B

54403010	Popcorn, air-popped, unbuttered	9000_SavorySweet	82	78	4	1	5	A
54206010	Puffed rice cake without salt	9000_SavorySweet	80	78	2	1	4	A
54403000	Popcorn, popped in oil, unbuttered	9000_SavorySweet	76	74	2	1	4	D
54403090	Popcorn, popped in oil, unsalted	9000_SavorySweet	75	73	2	1	4	C
11830165	Chocolate beverage powder, light, dry mix, not reconstituted	9000_SavorySweet	72	81	-9	4	3	D
41310900	Bean chips	9000_SavorySweet	72	71	1	2	5	B
54403046	Popcorn, popped in oil, with added butter or margarine	9000_SavorySweet	71	71	0	1	2.5	D
91701010	Almonds, chocolate covered	9000_SavorySweet	69	78	-9	4	3	D
54304500	Cracker, high fiber, no added fat	9000_SavorySweet	68	71	-3	4	4.5	B
91511070	Gelatin dessert, dietetic, with fruit and sour cream, sweetened with low calorie sweetener	9000_SavorySweet	67	66	1	3	4	A
54403040	Popcorn, air-popped, with added butter or margarine	9000_SavorySweet	66	62	4	1	4	C
11460200	Yogurt, frozen, chocolate, nonfat milk	9000_SavorySweet	65	72	-7	4	3.5	C
11460400	Yogurt, frozen, chocolate, nonfat milk, with low-calorie sweetener	9000_SavorySweet	65	72	-7	4	3.5	C
11460300	Yogurt, frozen, flavors other than chocolate, nonfat milk	9000_SavorySweet	64	71	-7	4	3.5	C
11460410	Yogurt, frozen, flavors other than chocolate, nonfat milk, with low-calorie sweetener	9000_SavorySweet	64	71	-7	4	3.5	C
53248000	Cookie, whole wheat, dried fruit, nut	9000_SavorySweet	63	66	-3	2	2	D
53233500	Cookie, oat bran	9000_SavorySweet	61	72	-11	4	4	C
91703500	Nuts, carob-coated	9000_SavorySweet	60	69	-9	4	1.5	D
91728500	Sugared pecans, sugar and egg white coating	9000_SavorySweet	60	69	-9	4	3	D
54207010	Crispbread, wheat, low sodium	9000_SavorySweet	60	64	-4	4	4.5	B
54222000	Crispbread, rye, low sodium	9000_SavorySweet	60	64	-4	4	4.5	B
54305010	Crackers, crispbread	9000_SavorySweet	60	64	-4	4	4.5	B
54322000	Crispbread, rye, no added fat	9000_SavorySweet	60	64	-4	4	4.5	B
71201300	Potato based snacks, reduced fat, low sodium, all flavors	9000_SavorySweet	60	57	3	2	4	C
73410210	Sweet potato chips	9000_SavorySweet	59	68	-9	4	4.5	B
51808050	Breadsticks, hard, gluten free	9000_SavorySweet	59	67	-8	4	4	C
54340100	Crackers, gluten free, plain	9000_SavorySweet	59	67	-8	4	4	C
54340110	Crackers, gluten free, flavored	9000_SavorySweet	59	67	-8	4	4	C
54204010	Cracker, 100% whole wheat, low sodium	9000_SavorySweet	59	65	-6	4	4	B
54210010	Cracker, multigrain, low sodium	9000_SavorySweet	59	65	-6	4	4	B
54337100	Crackers, whole wheat and bran	9000_SavorySweet	58	65	-7	4	3	C
91742010	Sesame Crunch, Sahadi	9000_SavorySweet	57	67	-10	4	2.5	D
54203010	Crackers, toast thins (rye, wheat, white flour), low sodium	9000_SavorySweet	57	64	-7	4	3.5	C
54337000	Cracker, 100% whole wheat	9000_SavorySweet	56	64	-8	4	3	C
54337050	Cracker, 100% whole wheat, reduced fat	9000_SavorySweet	56	63	-7	4	3	C
54318500	Rice cake	9000_SavorySweet	56	62	-6	4	4	A
11830110	Cocoa powder with nonfat dry milk and low calorie sweetener, dry mix, not reconstituted	9000_SavorySweet	56	61	-5	4	2	E

11830170	Cocoa (or chocolate) flavored beverage powder with low-calorie sweetener, dry mix, not reconstituted	9000_SavorySweet	56	61	-5	4	2	E
91708010	Date candy	9000_SavorySweet	55	62	-7	4	2.5	C
54403083	Popcorn, ready-to-eat packaged, low sodium	9000_SavorySweet	55	60	-5	4	4	C
11830120	Cocoa, whey, and low calorie sweetener, fortified, dry mix, not reconstituted	9000_SavorySweet	54	60	-6	4	1	E
71202000	Potato chips, unsalted	9000_SavorySweet	54	60	-6	4	4	C
54403070	Popcorn, popped in oil, lowfat	9000_SavorySweet	54	58	-4	4	4.5	C
54403082	Popcorn, ready-to-eat packaged, plain, light	9000_SavorySweet	54	58	-4	4	4	C
53452100	Pastry, fruit-filled	9000_SavorySweet	54	50	4	2	3.5	C
54403054	Popcorn, microwave, low sodium	9000_SavorySweet	53	59	-6	4	3	C
54403055	Popcorn, microwave, unsalted	9000_SavorySweet	53	59	-6	4	3	C
71202100	Potato chips, reduced fat, unsalted	9000_SavorySweet	53	58	-5	4	4.5	A
11830115	Hot chocolate / Cocoa, dry mix, no sugar added, not reconstituted	9000_SavorySweet	53	57	-4	4	2	E
54403086	Popcorn, ready-to-eat packaged, butter flavored, light	9000_SavorySweet	53	57	-4	4	3	C
54403088	Popcorn, ready-to-eat packaged, cheese flavored, light	9000_SavorySweet	53	57	-4	4	3	C
54403061	Popcorn, microwave, kettle corn, light	9000_SavorySweet	53	56	-3	4	4.5	C
91511110	Gelatin dessert, dietetic, with fruit and whipped topping, sweetened with low calorie sweetener	9000_SavorySweet	53	50	3	2	3.5	B
91304080	Topping, fruit, unsweetened	9000_SavorySweet	52	60	-8	4	3.5	B
54401010	Salty snacks, corn or cornmeal base, nuts or nuggets, toasted	9000_SavorySweet	52	57	-5	4	3	C
54401011	Corn nuts	9000_SavorySweet	52	57	-5	4	3	C
54403081	Popcorn, ready-to-eat packaged, plain	9000_SavorySweet	52	56	-4	4	3	D
54403053	Popcorn, microwave, plain, light	9000_SavorySweet	52	55	-3	4	3	D
54403057	Popcorn, microwave, butter flavored, light	9000_SavorySweet	52	55	-3	4	3	D
54403060	Popcorn, popped in oil, lowfat, reduced sodium	9000_SavorySweet	52	54	-2	4	4	C
54402500	Salty snacks, wheat- and corn-based chips	9000_SavorySweet	51	57	-6	4	3	C
54402600	Salty snacks, multigrain, whole grain, chips (made with whole corn, whole wheat, rice flour, and whole oat flour)	9000_SavorySweet	51	57	-6	4	3	C
54420210	Multigrain chips (Sun Chips)	9000_SavorySweet	51	57	-6	4	3	C
54440020	Cracker chips	9000_SavorySweet	51	57	-6	4	3	C
54403059	Popcorn, microwave, kettle corn	9000_SavorySweet	51	56	-5	4	3	C
54420100	Oriental party mix, with peanuts, sesame sticks, chili rice crackers and fried green peas	9000_SavorySweet	51	55	-4	4	5	C
13250200	Mousse, chocolate, lowfat, reduced calorie, prepared from dry mix, water added	9000_SavorySweet	51	51	0	2	3.5	C
91511080	Gelatin dessert, dietetic, with fruit and cream cheese, sweetened with low calorie sweetener	9000_SavorySweet	51	46	5	2	3.5	B
91501090	Gelatin dessert with fruit, vegetable, and nuts	9000_SavorySweet	50	58	-8	2	3.5	B
56205240	Rice dessert bar, frozen, chocolate, nondairy, chocolate covered	9000_SavorySweet	50	57	-7	4	3.5	C
71202500	Potato chips, lightly salted	9000_SavorySweet	50	57	-7	4	4	C
71201010	White potato, chips	9000_SavorySweet	50	56	-6	4	3.5	C
71202510	Potato chips, restructured, lightly salted	9000_SavorySweet	50	56	-6	4	4	C

54403080	Popcorn, ready-to-eat packaged, NFS	9000_SavorySweet	50	55	-5	4	2.5	D
54403085	Popcorn, ready-to-eat packaged, butter flavored	9000_SavorySweet	50	55	-5	4	2.5	D
71220000	Vegetable chips	9000_SavorySweet	49	58	-9	4	4.5	C
54403050	Popcorn, flavored	9000_SavorySweet	49	55	-6	4	2.5	D
54403087	Popcorn, ready-to-eat packaged, cheese flavored	9000_SavorySweet	49	55	-6	4	2.5	D
54403092	Popcorn, ready-to-eat packaged, other flavored	9000_SavorySweet	49	55	-6	4	2.5	D
56205200	Rice, frozen dessert, nondairy, flavors other than chocolate	9000_SavorySweet	49	55	-6	4	3.5	B
71200110	Potato chips, barbecue flavored	9000_SavorySweet	49	55	-6	4	3.5	D
71200120	Potato chips, sour cream and onion flavored	9000_SavorySweet	49	55	-6	4	3.5	D
71200140	Potato chips, other flavored	9000_SavorySweet	49	55	-6	4	3.5	D
71200210	Potato chips, ruffled, barbecue flavored	9000_SavorySweet	49	55	-6	4	3.5	D
53116500	Cake or cupcake, pumpkin, without icing or filling	9000_SavorySweet	49	51	-2	2	2	D
13220220	Pudding, chocolate, made from dry mix, sugar free	9000_SavorySweet	49	43	6	2	3.5	C
11461270	Yogurt, frozen, cone, flavors other than chocolate, lowfat milk	9000_SavorySweet	48	54	-6	4	3.5	C
71200130	Potato chips, cheese flavored	9000_SavorySweet	48	54	-6	4	3.5	D
71200220	Potato chips, ruffled, sour cream and onion flavored	9000_SavorySweet	48	54	-6	4	3.5	D
71200230	Potato chips, ruffled, cheese flavored	9000_SavorySweet	48	54	-6	4	3.5	D
71200240	Potato chips, ruffled, other flavored	9000_SavorySweet	48	54	-6	4	3.5	D
54319010	Puffed rice cake	9000_SavorySweet	48	53	-5	4	4	C
54401020	Salty snacks, corn or cornmeal base, corn chips, corn-cheese chips	9000_SavorySweet	48	53	-5	4	2.5	D
54401026	Corn chips, flavored	9000_SavorySweet	48	53	-5	4	2.5	D
54401031	Corn chips, plain (Fritos)	9000_SavorySweet	48	53	-5	4	2.5	D
54319020	Popcorn cake	9000_SavorySweet	48	52	-4	4	4	C
53104100	Cake or cupcake, carrot, without icing or filling	9000_SavorySweet	48	50	-2	2	2	D
13210810	Pumpkin pudding, Puerto Rican style	9000_SavorySweet	48	43	5	2	3.5	C
91727010	Nuts, chocolate covered, not almonds or peanuts	9000_SavorySweet	47	55	-8	4	1.5	E
54403000	Popcorn, popped in oil, unbuttered	9000_SavorySweet	47	53	-6	4	2	D
71200010	Potato chips, NFS	9000_SavorySweet	47	53	-6	4	3.5	C
71200100	Potato chips, plain	9000_SavorySweet	47	53	-6	4	3.5	C
71200200	Potato chips, ruffled, plain	9000_SavorySweet	47	53	-6	4	3.5	C
71201015	White potato chips, regular cut	9000_SavorySweet	47	53	-6	4	3.5	C
71201020	White potato chips, ruffled, rippled, or crinkle cut	9000_SavorySweet	47	53	-6	4	3.5	C
71204000	Potato puffs, cheese-filled	9000_SavorySweet	47	53	-6	4	3.5	C
71201050	Potato chips, reduced fat	9000_SavorySweet	47	51	-4	4	4	C
53102600	Cake or cupcake, banana, without icing or filling	9000_SavorySweet	47	48	-1	2	2	D
54401021	Corn chips, plain	9000_SavorySweet	46	51	-5	4	2	E
91732100	Planters Peanut Bar	9000_SavorySweet	45	55	-10	4	1.5	E
56205230	Rice dessert bar, frozen, flavors other than chocolate, nondairy, carob covered	9000_SavorySweet	45	51	-6	4	3	C

54305020	Crackers, flatbread	9000_SavorySweet	45	48	-3	4	4	C
53102100	Cake or cupcake, applesauce, without icing or filling	9000_SavorySweet	45	46	-1	2	2	D
53117100	Cake or cupcake, spice, without icing or filling	9000_SavorySweet	45	46	-1	2	2	D
53120500	Cake, whole wheat, with fruit and nuts, without icing	9000_SavorySweet	45	44	1	2	2	E
11460160	Yogurt, frozen, chocolate, lowfat milk	9000_SavorySweet	44	51	-7	4	3.5	C
54204020	Crackers, wheat, reduced sodium	9000_SavorySweet	44	48	-4	4	4	B
54204030	Crackers, woven wheat, reduced sodium	9000_SavorySweet	44	48	-4	4	4	B
71205000	White potato, sticks	9000_SavorySweet	44	48	-4	4	3	D
71205020	Potato sticks, plain	9000_SavorySweet	44	48	-4	4	3	D
91407150	Bean paste, sweetened	9000_SavorySweet	44	48	-4	2	4	C
91301082	Chocolate syrup, thin type, sugar free	9000_SavorySweet	44	41	3	4	3.5	C
54328120	Crackers, whole grain, sandwich, peanut butter filled	9000_SavorySweet	43	48	-5	4	2.5	D
54200100	Crackers, butter, reduced sodium	9000_SavorySweet	43	47	-4	4	4	B
54318000	Chips, rice	9000_SavorySweet	43	47	-4	4	4	C
91716010	Halvah, plain	9000_SavorySweet	42	53	-11	4	1.5	E
54401035	Corn chips, flavored (Fritos)	9000_SavorySweet	42	52	-10	4	2	D
51306000	Breadsticks, hard, whole wheat	9000_SavorySweet	42	47	-5	4	3	C
54401090	Corn chips, reduced sodium	9000_SavorySweet	42	47	-5	4	2.5	D
71205040	Potato sticks, fry shaped	9000_SavorySweet	42	46	-4	4	2.5	E
71205030	Potato sticks, flavored	9000_SavorySweet	42	45	-3	4	2.5	E
71980200	Taro chips	9000_SavorySweet	42	45	-3	4	3.5	C
53415200	Fritter, banana	9000_SavorySweet	42	37	5	3	3	D
53452450	Cheese pastry puffs	9000_SavorySweet	42	35	7	3	2	D
11460190	Yogurt, frozen, NS as to flavor, nonfat milk	9000_SavorySweet	41	49	-8	4	3	C
71200400	Potato chips, baked, plain	9000_SavorySweet	41	45	-4	4	4	C
71203010	Potato chips, popped, plain	9000_SavorySweet	41	45	-4	4	4	C
91406000	Jam, preserve, marmalade, sugar free, all flavors	9000_SavorySweet	41	45	-4	4	3.5	B
71201090	White potato, chips, fat free, made with Olean	9000_SavorySweet	41	43	-2	4	4.5	A
53116550	Cake or cupcake, raisin-nut	9000_SavorySweet	41	42	-1	3	1.5	E
53415220	Fritter, berry	9000_SavorySweet	41	37	4	3	2.5	D
91701030	Almonds, yogurt-covered	9000_SavorySweet	40	50	-10	4	1.5	E
91728000	Nut roll, fudge or nougat, caramel and nuts	9000_SavorySweet	40	48	-8	4	1.5	E
91731100	Peanuts, sugar-coated	9000_SavorySweet	40	48	-8	4	1.5	E
91732000	Peanut bar	9000_SavorySweet	40	48	-8	4	1.5	E
54403091	Popcorn, ready-to-eat packaged, kettle corn, light	9000_SavorySweet	40	45	-5	4	3	D
54337060	Crackers, woven wheat, reduced fat	9000_SavorySweet	40	44	-4	4	3	C
71203030	Potato chips, popped, NFS	9000_SavorySweet	40	44	-4	4	4	C
53440500	Strudel, cherry	9000_SavorySweet	40	43	-3	2	2.5	D
71200410	Potato chips, baked, flavored	9000_SavorySweet	40	43	-3	4	3.5	C

71201250	White potato, chips, restructured, baked	9000_SavorySweet	40	43	-3	4	4	C
71905410	Plantain chips	9000_SavorySweet	40	43	-3	4	3	C
53118350	Cake, sweetpotato, with icing	9000_SavorySweet	40	42	-2	2	1.5	E
13140710	Creamsicle, light	9000_SavorySweet	40	40	0	4	3.5	A
54325050	Crackers, saltine, whole wheat	9000_SavorySweet	39	44	-5	4	2.5	D
71201100	White potato, chips, restructured	9000_SavorySweet	39	43	-4	4	3	D
71203020	Potato chips, popped, flavored	9000_SavorySweet	39	43	-4	4	3.5	C
71211000	White potato skins, chips	9000_SavorySweet	39	43	-4	4	3	D
71201080	White potato, chips, fat free	9000_SavorySweet	39	41	-2	4	5	C
11461280	Yogurt, frozen, cone, chocolate, lowfat milk	9000_SavorySweet	38	44	-6	4	3.5	C
54325060	Crackers, saltine, multigrain	9000_SavorySweet	38	43	-5	4	2.5	D
71200300	Potato chips, restructured, plain	9000_SavorySweet	38	41	-3	4	2.5	D
71200310	Potato chips, restructured, flavored	9000_SavorySweet	38	41	-3	4	2.5	D
71201060	Potato chips, fat free	9000_SavorySweet	38	41	-3	4	4	C
13160160	Fat free ice cream, no sugar added, flavors other than chocolate	9000_SavorySweet	38	40	-2	4	3.5	B
53387000	Pie, Toll house chocolate chip	9000_SavorySweet	38	38	0	3	1	E
53451500	Turnover, guava	9000_SavorySweet	38	34	4	2	3	C
53420310	Wheat flour fritter, without syrup	9000_SavorySweet	38	33	5	3	1.5	D
55801000	Funnel cake with sugar	9000_SavorySweet	38	33	5	2	2	D
56116000	Noodles, chow mein	9000_SavorySweet	38	33	5	1	2	D
53451750	Turnover, pumpkin	9000_SavorySweet	38	32	6	2	3	C
13210110	Pudding, bread	9000_SavorySweet	38	29	9	2	3	C
13210300	Custard	9000_SavorySweet	38	28	10	1	3.5	C
91701020	Almonds, sugar-coated	9000_SavorySweet	37	50	-13	4	1.5	E
53210910	Cookie, graham cracker with marshmallow	9000_SavorySweet	37	48	-11	4	1.5	E
91501110	Gelatin dessert with fruit and whipped topping	9000_SavorySweet	37	44	-7	3	3	C
91405000	Jelly, sugar free, all flavors	9000_SavorySweet	37	40	-3	4	3.5	B
53115310	Cake or cupcake, nut, without icing or filling	9000_SavorySweet	37	38	-1	3	1.5	E
58124210	Pastry, cheese-filled	9000_SavorySweet	37	36	1	3	2	D
13220210	Pudding, flavors other than chocolate, made from dry mix, sugar free	9000_SavorySweet	37	30	7	2	3.5	C
53415120	Fritter, apple	9000_SavorySweet	37	30	7	3	2.5	D
91304060	Topping, nuts and syrup	9000_SavorySweet	36	50	-14	4	2	D
91731150	Peanuts, yogurt covered	9000_SavorySweet	36	45	-9	4	1	E
54401120	Salty snacks, corn or cornmeal base, tortilla chips, fat free, made with Olean	9000_SavorySweet	36	38	-2	4	3.5	C
71201200	Potato chips, restructured, reduced fat, lightly salted	9000_SavorySweet	36	38	-2	4	3	D
53415300	Crisp, blueberry	9000_SavorySweet	36	37	-1	2	2.5	D
53310000	Pie, raspberry, one crust	9000_SavorySweet	36	35	1	2	3.5	C
58157210	Rice pudding made with coconut milk, Puerto Rican style	9000_SavorySweet	36	32	4	2	1.5	D

91550300	Pineapple custard, Puerto Rican style	9000_SavorySweet	36	31	5	1	3	C
32120200	Zabaglione	9000_SavorySweet	36	29	7	2	2.5	D
11830140	Chocolate, instant, dry mix, fortified with vitamins and minerals, not reconstituted, Puerto Rican style	9000_SavorySweet	35	51	-16	4	1	E
44201000	Carob chips	9000_SavorySweet	35	40	-5	4	1.5	E
53124120	Cake, zucchini, with icing	9000_SavorySweet	35	37	-2	2	1.5	E
53440300	Strudel, berry	9000_SavorySweet	35	34	1	2	3	C
55801010	Funnel cake with sugar and fruit	9000_SavorySweet	35	33	2	2	2	D
53346000	Pie, peanut butter cream	9000_SavorySweet	35	30	5	2	2	D
53540250	Breakfast bar, cereal crust with fruit filling, fat free	9000_SavorySweet	34	45	-11	4	1.5	E
53104000	Cake, carrot, NS as to icing	9000_SavorySweet	34	36	-2	2	1.5	E
53303000	Pie, blackberry, two crust	9000_SavorySweet	34	31	3	2	3	C
53452200	Pastry, Italian, with cheese	9000_SavorySweet	34	30	4	2	2.5	D
53452170	Pastry, cookie type, fried	9000_SavorySweet	34	28	6	2	2	D
53344200	Mixed fruit tart filled with custard or cream cheese	9000_SavorySweet	34	27	7	3	3	C
11830210	Milk, malted, dry mix, fortified, not reconstituted, flavors other than chocolate	9000_SavorySweet	33	43	-10	4	0.5	E
91406500	Jam, preserve, marmalade, sweetened with fruit juice concentrates, all flavors	9000_SavorySweet	33	40	-7	3	3	C
91406600	Jam, preserve, marmalade, reduced sugar, all flavors	9000_SavorySweet	33	40	-7	3	2.5	C
54403089	Popcorn, ready-to-eat-packaged, kettle corn	9000_SavorySweet	33	39	-6	4	2	E
91731000	Peanuts, chocolate covered	9000_SavorySweet	33	38	-5	4	1	E
11460430	Yogurt, frozen, chocolate, whole milk	9000_SavorySweet	33	37	-4	4	3	C
11461220	Frozen yogurt bar, chocolate	9000_SavorySweet	33	37	-4	4	3	C
54402080	Tortilla chips, reduced sodium	9000_SavorySweet	33	36	-3	4	3.5	C
13160400	Fat free ice cream, flavors other than chocolate	9000_SavorySweet	33	35	-2	4	3.5	B
13160420	Fat free ice cream, NS as to flavor	9000_SavorySweet	33	35	-2	4	3.5	B
54304150	Crackers, cheese, whole grain	9000_SavorySweet	33	35	-2	4	2.5	D
13161600	Fudgesicle, light	9000_SavorySweet	33	34	-1	4	3.5	B
13210530	Pudding, tapioca, chocolate, made with milk	9000_SavorySweet	33	31	2	2	3	C
58149160	Noodle pudding, with milk	9000_SavorySweet	33	31	2	1	2.5	D
53303070	Pie, blackberry, individual size or tart	9000_SavorySweet	33	30	3	2	2.5	D
13250000	Mousse	9000_SavorySweet	33	27	6	3	2	D
58118110	Cornstarch coconut dessert, Puerto Rican style	9000_SavorySweet	33	27	6	2	1.5	D
91703060	Caramel with nuts, chocolate covered	9000_SavorySweet	32	42	-10	4	1.5	E
91716110	Halvah, chocolate covered	9000_SavorySweet	32	42	-10	4	1	E
11460100	Frozen yogurt, chocolate	9000_SavorySweet	32	37	-5	4	3	C
53116510	Cake or cupcake, pumpkin, with icing or filling	9000_SavorySweet	32	33	-1	2	1	E
54403051	Popcorn, microwave, NFS	9000_SavorySweet	32	33	-1	4	1.5	E
53102700	Cake or cupcake, banana, with icing or filling	9000_SavorySweet	32	31	1	2	1	E
53415100	Crisp, apple, apple dessert	9000_SavorySweet	32	31	1	3	4	C

53452150	Pastry, Chinese, made with rice flour	9000_SavorySweet	32	30	2	2	3	C
53440800	Strudel, cheese and fruit	9000_SavorySweet	32	29	3	2	2.5	D
91700500	M&M's Almond Chocolate Candies	9000_SavorySweet	31	41	-10	4	1	E
91405500	Jelly, reduced sugar, all flavors	9000_SavorySweet	31	37	-6	3	2.5	C
54309000	Crackers, oat	9000_SavorySweet	31	36	-5	4	2.5	D
54334000	Crackers, toast thins (rye, pumpernickel, white flour)	9000_SavorySweet	31	36	-5	4	2.5	D
91501120	Gelatin dessert with fruit and vegetables	9000_SavorySweet	31	35	-4	3	3.5	B
91520100	Yokan	9000_SavorySweet	31	35	-4	2	3	C
91703080	Caramel, all flavors, sugar free	9000_SavorySweet	31	35	-4	4	2.5	D
54401100	Salty snacks, corn or cornmeal base, tortilla chips, light (baked with less oil)	9000_SavorySweet	31	33	-2	4	2.5	D
13230140	Pudding, chocolate, ready-to-eat, sugar free	9000_SavorySweet	31	32	-1	4	3.5	C
53104260	Cake or cupcake, carrot, with icing or filling	9000_SavorySweet	31	32	-1	2	1	E
54403001	Popcorn, NFS	9000_SavorySweet	31	32	-1	4	1.5	E
54403052	Popcorn, microwave, plain	9000_SavorySweet	31	32	-1	4	1.5	E
54403056	Popcorn, microwave, butter flavored	9000_SavorySweet	31	32	-1	4	1.5	E
54403058	Popcorn, microwave, cheese flavored	9000_SavorySweet	31	31	0	4	1.5	E
54403062	Popcorn, microwave, other flavored	9000_SavorySweet	31	31	0	4	1.5	E
53303510	Pie, berry, not blackberry, blueberry, boysenberry, huckleberry, raspberry, or strawberry; one crust	9000_SavorySweet	31	30	1	2	2.5	D
53312000	Pie, strawberry, one crust	9000_SavorySweet	31	28	3	2	3	C
53310050	Pie, raspberry, two crust	9000_SavorySweet	31	27	4	2	3	C
91304070	Topping, peanut butter, thick, fudge type	9000_SavorySweet	30	42	-12	4	2	D
13120750	Ice cream cone with nuts, chocolate ice cream	9000_SavorySweet	30	37	-7	4	2.5	D
91733200	Peanut Bar, chocolate covered candy	9000_SavorySweet	30	36	-6	4	1	E
41410015	Soy chips	9000_SavorySweet	30	32	-2	4	2.5	D
53106000	Cake, chocolate, devil's food, or fudge, pudding-type mix (oil, eggs, and water added to dry mix), without icing or filling	9000_SavorySweet	30	31	-1	3	1.5	E
53124110	Cake or cupcake, zucchini	9000_SavorySweet	30	31	-1	2	1	E
53410300	Cobbler, berry	9000_SavorySweet	30	28	2	2	2.5	C
91560100	Haupia	9000_SavorySweet	30	26	4	2	1.5	D
53347600	Pie, squash	9000_SavorySweet	30	25	5	2	3	C
13210270	Custard, Puerto Rican style	9000_SavorySweet	30	23	7	1	3	C
44202000	Carob syrup	9000_SavorySweet	29	43	-14	4	2.5	D
54337010	Crackers, woven wheat	9000_SavorySweet	29	34	-5	4	2.5	D
54337020	Crackers, woven wheat, plain (Triscuit)	9000_SavorySweet	29	34	-5	4	2.5	D
54337030	Crackers, woven wheat, flavored (Triscuit)	9000_SavorySweet	29	34	-5	4	2.5	D
54338010	Crackers, wheat, plain (Wheat Thins)	9000_SavorySweet	29	34	-5	4	2.5	D
11461250	Frozen yogurt cone, chocolate	9000_SavorySweet	29	33	-4	4	3	C
54338000	Crackers, wheat	9000_SavorySweet	29	33	-4	4	2.5	D
91770030	Dietetic or low calorie candy, chocolate covered	9000_SavorySweet	29	33	-4	4	0.5	E

53105700	Cake, chocolate, devil's food, or fudge, pudding type mix, made by "cholesterol free" recipe (water, oil and egg whites added to dry mix), without icing or filling	9000_SavorySweet	29	32	-3	3	1.5	E
91739510	Raisins, carob covered	9000_SavorySweet	29	32	-3	4	2	E
53102200	Cake or cupcake, applesauce, with icing or filling	9000_SavorySweet	29	30	-1	2	1	E
53117200	Cake or cupcake, spice, with icing or filling	9000_SavorySweet	29	29	0	2	1	E
91511020	Gelatin dessert, sugar free, with fruit	9000_SavorySweet	29	29	0	4	3.5	A
53452120	Pastry, made with bean or lotus seed paste filling, baked	9000_SavorySweet	29	27	2	1	2.5	D
53314000	Pie, strawberry, individual size or tart	9000_SavorySweet	29	25	4	2	2.5	D
53304050	Pie, blueberry, one crust	9000_SavorySweet	29	24	5	3	2.5	C
53341070	Pie, banana cream, individual size or tart	9000_SavorySweet	29	22	7	2	2.5	C
53343070	Pie, coconut cream, individual size or tart	9000_SavorySweet	29	22	7	2	3	C
13210500	Pudding, tapioca, made from home recipe, made with milk	9000_SavorySweet	29	21	8	1	3	C
91108010	Sugar substitute, stevia, liquid	9000_SavorySweet	29	18	11	2	3.5	B
13120780	Ice cream cone, chocolate covered, with nuts, chocolate ice cream	9000_SavorySweet	28	35	-7	4	2	D
13120700	Ice cream cone with nuts, flavors other than chocolate	9000_SavorySweet	28	34	-6	4	2.5	D
91403000	Fruit butter, all flavors	9000_SavorySweet	28	32	-4	3	3	C
13110310	Ice cream, no sugar added, NS as to flavor	9000_SavorySweet	28	30	-2	4	3	C
13110320	Ice cream, no sugar added, flavors other than chocolate	9000_SavorySweet	28	30	-2	4	3	C
13140580	Light ice cream, no sugar added, cone, chocolate	9000_SavorySweet	28	30	-2	4	3	C
53206020	Cookie, chocolate chip, made from home recipe or purchased at a bakery	9000_SavorySweet	28	29	-1	2	0.5	E
53415500	Crisp, peach	9000_SavorySweet	28	28	0	2	2.5	D
54403020	Popcorn, popped in oil, buttered	9000_SavorySweet	28	27	1	4	1.5	E
13241000	Banana pudding	9000_SavorySweet	28	26	2	3	3	C
13210450	Firni, Indian pudding	9000_SavorySweet	28	24	4	1	3	C
53303500	Pie, berry, not blackberry, blueberry, boysenberry, huckleberry, raspberry, or strawberry; two crust	9000_SavorySweet	28	24	4	2	2.5	D
53441110	Baklava	9000_SavorySweet	28	24	4	2	1	E
51165060	Coffee cake, yeast type, made from home recipe or purchased at a bakery	9000_SavorySweet	28	22	6	2	2.5	D
53313000	Pie, strawberry-rhubarb, two crust	9000_SavorySweet	28	21	7	2	2.5	D
13161000	Milk dessert bar, frozen, made from lowfat milk	9000_SavorySweet	27	34	-7	4	4	C
54338020	Crackers, wheat, flavored (Wheat Thins)	9000_SavorySweet	27	34	-7	4	2.5	D
91705070	Chocolate, milk, with peanuts	9000_SavorySweet	27	34	-7	4	0.5	E
51184100	Breadsticks, hard, reduced sodium	9000_SavorySweet	27	32	-5	4	4	B
53102300	Cake, applesauce, diet, without icing	9000_SavorySweet	27	30	-3	4	4	C
63113030	Cherry pie filling	9000_SavorySweet	27	30	-3	2	2.5	C
54402200	Snack mix	9000_SavorySweet	27	29	-2	4	2	D
54408310	Pretzels, hard, peanut butter filled	9000_SavorySweet	27	29	-2	4	1.5	E
13110330	Ice cream, no sugar added, chocolate	9000_SavorySweet	27	28	-1	4	3.5	C

91501070	Gelatin dessert with fruit and sour cream	9000_SavorySweet	27	28	-1	3	3	C
71201210	Potato chips, restructured, fat free	9000_SavorySweet	27	27	0	4	3	D
13140575	Light ice cream, no sugar added, cone, flavors other than chocolate	9000_SavorySweet	27	26	1	4	3.5	C
53410800	Cobbler, peach	9000_SavorySweet	27	24	3	2	3	C
53410850	Cobbler, pear	9000_SavorySweet	27	24	3	2	3	D
13210710	Pudding, cornmeal, milk, and molasses	9000_SavorySweet	27	23	4	1	3	C
53360000	Pie, sweet potato	9000_SavorySweet	27	23	4	3	2	D
53440600	Strudel, cheese	9000_SavorySweet	27	23	4	2	2	D
58123120	Sweet bread dough, filled with bean paste, meatless, steamed	9000_SavorySweet	27	23	4	1	3.5	C
53115400	Cake, oatmeal, without icing	9000_SavorySweet	27	22	5	2	1.5	E
53302000	Pie, apricot, two crust	9000_SavorySweet	27	22	5	2	2.5	D
53303570	Pie, berry, not blackberry, blueberry, boysenberry, huckleberry, raspberry, or strawberry, individual size or tart	9000_SavorySweet	27	22	5	2	2.5	D
53304070	Pie, blueberry, individual size or tart	9000_SavorySweet	27	22	5	2	2.5	D
53430750	Tamale, sweet, with fruit	9000_SavorySweet	27	22	5	2	3	C
53309000	Pie, raisin, two crust	9000_SavorySweet	27	20	7	2	2.5	D
53309070	Pie, raisin, individual size or tart	9000_SavorySweet	27	20	7	2	2.5	D
53452130	Pastry, made with bean paste and salted egg yolk filling, baked	9000_SavorySweet	27	19	8	1	1.5	E
53344070	Pie, custard, individual size or tart	9000_SavorySweet	27	18	9	2	3	C
91705310	Chocolate, sweet or dark, with almonds	9000_SavorySweet	26	32	-6	4	1	E
91734500	Peanut butter morsels	9000_SavorySweet	26	32	-6	4	1	E
54102100	Graham crackers, reduced fat	9000_SavorySweet	26	31	-5	4	2.5	D
11461100	Yogurt, frozen, carob-coated	9000_SavorySweet	26	30	-4	4	1.5	E
11461320	Frozen yogurt cone, chocolate, waffle cone	9000_SavorySweet	26	29	-3	4	3	C
13140570	Light ice cream, no sugar added, cone, NS as to flavor	9000_SavorySweet	26	27	-1	4	3	C
54401065	Cheese flavored corn snacks, reduced fat	9000_SavorySweet	26	27	-1	4	2	D
54401085	Tortilla chips, flavored	9000_SavorySweet	26	27	-1	4	2.5	D
53100050	Cake batter, raw, chocolate	9000_SavorySweet	26	26	0	3	2	D
53610200	Coffee cake, crumb or quick-bread type, cheese-filled	9000_SavorySweet	26	23	3	2	1.5	E
53203500	Cookie, biscotti	9000_SavorySweet	26	22	4	1	2	D
53115600	Cake, poppyseed, without icing	9000_SavorySweet	26	21	5	2	1.5	E
13210150	Bread pudding made with evaporated milk and rum	9000_SavorySweet	26	20	6	3	2	D
53311000	Pie, rhubarb, two crust	9000_SavorySweet	26	20	6	3	2.5	D
53342070	Pie, chocolate cream, individual size or tart	9000_SavorySweet	26	20	6	2	2	D
53450300	Turnover or dumpling, berry	9000_SavorySweet	26	20	6	2	2	D
91731010	M&M's Peanut Chocolate Candies	9000_SavorySweet	25	35	-10	4	0.5	E
91303000	Molasses	9000_SavorySweet	25	32	-7	2	1	D
91734000	Peanut butter, chocolate covered	9000_SavorySweet	25	32	-7	4	0.5	E
91705040	Chocolate, milk, with nuts, not almond or peanuts	9000_SavorySweet	25	30	-5	4	0.5	E

54102110	Crackers, graham, fat free	9000_SavorySweet	25	29	-4	4	2.5	D
54103000	Crackers, breakfast biscuit	9000_SavorySweet	25	29	-4	4	2.5	D
54301100	Crackers, butter, reduced fat	9000_SavorySweet	25	29	-4	4	2	E
54338100	Crackers, wheat, reduced fat	9000_SavorySweet	25	29	-4	4	2	E
13161630	Light ice cream, bar or stick, with low-calorie sweetener, chocolate coated	9000_SavorySweet	25	28	-3	4	3	C
54401210	Salty snacks, corn based puffs and twists, cheese puffs and twists, lowfat	9000_SavorySweet	25	27	-2	4	2	D
54420200	Multigrain mixture, bread sticks, sesame nuggets, pretzels, rye chips	9000_SavorySweet	25	27	-2	4	1.5	E
13252500	Barfi or Burfi, Indian dessert	9000_SavorySweet	25	26	-1	2	1.5	E
63420205	Frozen fruit juice bar, no sugar added	9000_SavorySweet	25	26	-1	4	3.5	A
53105275	Cake or cupcake, chocolate, devil's food or fudge, without icing or filling	9000_SavorySweet	25	25	0	3	2	D
53260500	Cookie, sugar wafer, sugar free	9000_SavorySweet	25	25	0	4	4	C
53344300	Dessert pizza	9000_SavorySweet	25	25	0	3	3	C
91501080	Gelatin dessert with fruit and cream cheese	9000_SavorySweet	25	24	1	3	3	C
53348000	Pie, strawberry cream	9000_SavorySweet	25	23	2	3	2.5	C
53118310	Cake, sponge, chocolate, with icing	9000_SavorySweet	25	22	3	2	1.5	E
53307050	Pie, peach, one crust	9000_SavorySweet	25	22	3	2	3	C
53410500	Cobbler, cherry	9000_SavorySweet	25	22	3	2	2.5	D
53610100	Coffee cake, crumb or quick-bread type	9000_SavorySweet	25	22	3	1	1.5	E
13210370	Creme brulee	9000_SavorySweet	25	21	4	1	3	C
53305010	Pie, cherry, one crust	9000_SavorySweet	25	21	4	2	2.5	D
53311050	Pie, rhubarb, one crust	9000_SavorySweet	25	21	4	2	3	C
53348070	Pie, strawberry cream, individual size or tart	9000_SavorySweet	25	21	4	3	2.5	D
53453170	Empanada, Mexican turnover, pumpkin	9000_SavorySweet	25	17	8	2	3	D
11460510	Frozen yogurt, soft serve, chocolate	9000_SavorySweet	24	31	-7	4	2.5	D
54403006	Popcorn, movie theater, unbuttered	9000_SavorySweet	24	27	-3	4	0.5	E
53260200	Cookie, oatmeal, sugar free	9000_SavorySweet	24	25	-1	4	2	E
54401170	Tortilla chips, low fat, unsalted	9000_SavorySweet	24	25	-1	4	4	A
53347000	Pie, pumpkin	9000_SavorySweet	24	24	0	4	2.5	D
53347070	Pie, pumpkin, individual size or tart	9000_SavorySweet	24	24	0	4	2.5	D
13210180	Pudding, Mexican bread	9000_SavorySweet	24	23	1	3	2	D
53102500	Cake, banana, NS as to icing	9000_SavorySweet	24	23	1	2	2	D
53111500	Cake, graham cracker, without icing	9000_SavorySweet	24	22	2	3	1.5	E
53347100	Pie, raspberry cream	9000_SavorySweet	24	21	3	3	2.5	D
53200100	Cookie, batter or dough, raw	9000_SavorySweet	24	20	4	3	1	E
58118210	Cornmeal coconut dessert, Puerto Rican style	9000_SavorySweet	24	19	5	2	1	E
53306070	Pie, mince, individual size or tart	9000_SavorySweet	24	17	7	2	2	D
53451000	Turnover or dumpling, peach	9000_SavorySweet	24	17	7	2	2	D

53122080	Cake, shortcake, biscuit type, with fruit	9000_SavorySweet	24	15	9	1	2.5	D
91713060	Fudge, peanut butter, with nuts	9000_SavorySweet	23	34	-11	4	1	E
54403120	Popcorn, caramel coated, with nuts	9000_SavorySweet	23	31	-8	4	1.5	E
91705060	Milk chocolate candy, with almonds	9000_SavorySweet	23	28	-5	4	0.5	E
53260600	Cookie, peanut butter, sugar free	9000_SavorySweet	23	26	-3	4	2	D
54102050	Crackers, oatmeal	9000_SavorySweet	23	26	-3	4	2.5	D
54339000	Crackers, corn	9000_SavorySweet	23	26	-3	4	2.5	D
53116560	Cake, raisin-nut, with icing	9000_SavorySweet	23	25	-2	3	1.5	E
54401055	Cheese flavored corn snacks	9000_SavorySweet	23	25	-2	4	1.5	E
54401050	Salty snacks, corn or cornmeal base, corn puffs and twists; corn-cheese puffs and twists	9000_SavorySweet	23	24	-1	4	1.5	E
53205500	Cookie, butterscotch chip	9000_SavorySweet	23	23	0	3	0.5	E
13210250	Pudding, chocolate, low calorie, containing artificial sweetener, NS as to from dry mix or ready-to-eat	9000_SavorySweet	23	22	1	4	3	C
13250100	Mousse, not chocolate	9000_SavorySweet	23	22	1	3	2	D
53441210	Basbousa	9000_SavorySweet	23	22	1	2	2.5	D
53224250	Cookie, lemon bar	9000_SavorySweet	23	21	2	2	1.5	E
53410100	Cobbler, apple	9000_SavorySweet	23	21	2	2	2.5	C
13210190	Pudding, Mexican bread, low fat	9000_SavorySweet	23	20	3	3	2	D
53105500	Cake, chocolate, with icing, diet	9000_SavorySweet	23	20	3	4	2.5	E
53345000	Pie, lemon cream	9000_SavorySweet	23	20	3	2	2	D
53307500	Pie, pear, two crust	9000_SavorySweet	23	18	5	2	2.5	D
53307070	Pie, peach, individual size or tart	9000_SavorySweet	23	17	6	2	2.5	D
53450500	Turnover or dumpling, cherry	9000_SavorySweet	23	17	6	2	2.5	D
53122070	Cake, shortcake, biscuit type, with whipped cream and fruit	9000_SavorySweet	23	16	7	2	2	D
53311070	Pie, rhubarb, individual size or tart	9000_SavorySweet	23	16	7	3	2.5	D
53420400	Sopaipilla, without syrup or honey	9000_SavorySweet	23	15	8	2	1.5	E
91407100	Guava paste	9000_SavorySweet	22	31	-9	3	1.5	D
91734100	Reese's Peanut Butter Cup	9000_SavorySweet	22	30	-8	4	0.5	E
53223100	Cookie, granola	9000_SavorySweet	22	27	-5	4	1	E
53231400	Cookie, multigrain, high fiber	9000_SavorySweet	22	27	-5	4	1	E
54102080	Crackers, graham, with raisins	9000_SavorySweet	22	27	-5	4	2	D
91361020	Fruit sauce	9000_SavorySweet	22	27	-5	2	2	D
53415600	Crisp, rhubarb	9000_SavorySweet	22	26	-4	3	2	D
54326000	Crackers, multigrain	9000_SavorySweet	22	26	-4	4	1.5	E
13120130	Ice cream bar or stick, rich ice cream, chocolate covered, with nuts	9000_SavorySweet	22	24	-2	4	1.5	D
54328000	Crackers, sandwich	9000_SavorySweet	22	24	-2	4	1.5	E
54328100	Crackers, sandwich, peanut butter filled	9000_SavorySweet	22	24	-2	4	1.5	E
13210610	Pudding, coconut	9000_SavorySweet	22	23	-1	3	3	C
53410900	Cobbler, rhubarb	9000_SavorySweet	22	23	-1	2	2.5	D

53610170	Coffee cake, crumb or quick-bread type, with fruit	9000_SavorySweet	22	21	1	2	2	D
53115100	Cake or cupcake, marble, without icing or filling	9000_SavorySweet	22	20	2	3	1.5	E
53385500	Pie, oatmeal	9000_SavorySweet	22	20	2	3	1.5	E
53390000	Pie, shoo-fly	9000_SavorySweet	22	20	2	2	1.5	E
53105160	Cake, chocolate, devil's food, or fudge, without icing or filling, made from home recipe or purchased ready-to-eat	9000_SavorySweet	22	18	4	2	1.5	E
53345070	Pie, lemon cream, individual size or tart	9000_SavorySweet	22	18	4	2	2	D
13210160	Diplomat pudding, Puerto Rican style	9000_SavorySweet	22	17	5	3	1.5	D
53346500	Pie, pineapple cream	9000_SavorySweet	22	17	5	2	2.5	C
53116600	Cake, rice flour, without icing or filling	9000_SavorySweet	22	16	6	2	2	D
53308000	Pie, pineapple, two crust	9000_SavorySweet	22	16	6	3	2.5	D
53370000	Pie, chiffon, not chocolate	9000_SavorySweet	22	16	6	2	2	D
53450000	Turnover or dumpling, apple	9000_SavorySweet	22	15	7	2	2	D
63420110	Fruit juice bar, frozen, flavor other than orange	9000_SavorySweet	21	33	-12	4	3	C
63430100	Sorbet, fruit, noncitrus flavor	9000_SavorySweet	21	33	-12	4	3	C
91718000	Honey-combed hard candy with peanut butter	9000_SavorySweet	21	31	-10	4	0.5	E
91718050	Honey-combed hard candy with peanut butter, chocolate covered	9000_SavorySweet	21	27	-6	4	1	E
54102010	Graham crackers	9000_SavorySweet	21	26	-5	4	2	D
54102015	Graham crackers (Teddy Grahams)	9000_SavorySweet	21	26	-5	4	2	D
91705050	Milk chocolate candy, with fruit and nuts	9000_SavorySweet	21	26	-5	4	1	E
53366000	Pie, yogurt, frozen	9000_SavorySweet	21	25	-4	3	2	D
54402700	Pita chips	9000_SavorySweet	21	23	-2	4	2.5	D
54430010	Yogurt chips	9000_SavorySweet	21	23	-2	4	2	D
13130340	Light ice cream, no sugar added, chocolate	9000_SavorySweet	21	22	-1	4	3	C
13160150	Fat free ice cream, no sugar added, chocolate	9000_SavorySweet	21	22	-1	4	3	C
54401075	Tortilla chips, plain	9000_SavorySweet	21	22	-1	4	3.5	C
54401080	Salty snacks, corn or cornmeal base, tortilla chips	9000_SavorySweet	21	22	-1	4	3.5	C
54420010	Multigrain mixture, pretzels, cereal and/or crackers, nuts	9000_SavorySweet	21	22	-1	4	2.5	D
91501040	Gelatin dessert with fruit and whipped cream	9000_SavorySweet	21	22	-1	3	3	C
91550100	Coconut cream cake, Puerto Rican style	9000_SavorySweet	21	22	-1	2	1	E
13252100	Coconut custard, Puerto Rican style	9000_SavorySweet	21	20	1	2	1.5	E
53118500	Cake, torte	9000_SavorySweet	21	20	1	3	2	D
53120400	Cake, white, eggless, lowfat	9000_SavorySweet	21	20	1	2	1.5	E
53115410	Cake or cupcake, oatmeal	9000_SavorySweet	21	19	2	2	1.5	E
53116570	Cake, Ravani	9000_SavorySweet	21	19	2	2	2	D
53308500	Pie, prune, one crust	9000_SavorySweet	21	19	2	2	2	D
13210820	Fresh corn custard, Puerto Rican style	9000_SavorySweet	21	18	3	2	0.5	E
53301500	Pie, apple, one crust	9000_SavorySweet	21	18	3	2	2.5	D
91511050	Gelatin dessert, dietetic, with cream cheese, sweetened with low calorie sweetener	9000_SavorySweet	21	13	8	3	3.5	B

63203700	Blueberry pie filling	9000_SavorySweet	20	31	-11	4	2.5	D
41480010	Tofu, frozen dessert, chocolate	9000_SavorySweet	20	27	-7	4	3	D
91733000	Peanut brittle	9000_SavorySweet	20	27	-7	4	1	E
13140110	Light ice cream, bar or stick, chocolate covered, with nuts	9000_SavorySweet	20	25	-5	4	1.5	E
53260300	Cookie, sandwich, sugar free	9000_SavorySweet	20	22	-2	4	2.5	D
51168000	Bread, Spanish coffee	9000_SavorySweet	20	21	-1	4	3.5	C
54402610	Potato chips, restructured, multigrain	9000_SavorySweet	20	21	-1	4	3	D
13220120	Pudding, chocolate, made from dry mix	9000_SavorySweet	20	19	1	3	2.5	C
53102000	Cake, applesauce, NS as to icing	9000_SavorySweet	20	19	1	2	1.5	E
53105050	Cake, chocolate, devil's food, or fudge, made from home recipe or purchased ready-to-eat, NS as to icing	9000_SavorySweet	20	19	1	2	1	E
53119000	Cake, pineapple, upside down	9000_SavorySweet	20	17	3	2	1	E
53104300	Cake, carrot, diet	9000_SavorySweet	20	16	4	4	3	D
53420300	Air filled fritter or fried puff, without syrup, Puerto Rican style	9000_SavorySweet	20	16	4	2	1	E
53430700	Tamale, sweet	9000_SavorySweet	20	15	5	2	2.5	D
41480000	Tofu, frozen dessert, flavors other than chocolate	9000_SavorySweet	19	30	-11	4	2.5	D
91734450	Reese's Crispy Crunchy Bar	9000_SavorySweet	19	28	-9	4	0.5	E
54401110	Tortilla chips, nacho cheese flavor (Doritos)	9000_SavorySweet	19	27	-8	4	2.5	D
91726150	MARS Almond Bar	9000_SavorySweet	19	27	-8	4	1	E
53521250	Doughnut, wheat	9000_SavorySweet	19	23	-4	4	2	D
53115320	Cake or cupcake, nut, with icing or filling	9000_SavorySweet	19	21	-2	3	1	E
54403005	Popcorn, movie theater, with added butter	9000_SavorySweet	19	21	-2	4	0.5	E
54319005	Crackers, rice and nuts	9000_SavorySweet	19	20	-1	4	3.5	C
54327950	Crackers, cylindrical, peanut-butter filled	9000_SavorySweet	19	20	-1	4	1.5	E
54328110	Crackers, sandwich, reduced fat, peanut butter filled	9000_SavorySweet	19	19	0	4	2	D
91802000	Chewing gum, sugar free	9000_SavorySweet	19	18	1	4	3.5	C
53340500	Pie, cherry, made with cream cheese and sour cream	9000_SavorySweet	19	17	2	2	2	D
53450800	Turnover or dumpling, lemon	9000_SavorySweet	19	12	7	2	2.5	D
91708020	Soft fruit confections	9000_SavorySweet	18	30	-12	4	2	D
91705200	Chocolate, semi-sweet morsel	9000_SavorySweet	18	25	-7	4	0.5	E
54328210	Crackers, sandwich, cheese filled (Ritz)	9000_SavorySweet	18	24	-6	4	1.5	E
91407120	Sweet potato paste	9000_SavorySweet	18	24	-6	2	1.5	D
91705300	Chocolate, sweet or dark	9000_SavorySweet	18	24	-6	4	0.5	E
13130630	Light ice cream, soft serve cone, chocolate	9000_SavorySweet	18	23	-5	4	3	C
13142000	Milk dessert bar or stick, frozen, with coconut	9000_SavorySweet	18	23	-5	4	2.5	D
54301020	Crackers, butter, flavored	9000_SavorySweet	18	23	-5	4	1.5	E
54336100	Crackers, wonton	9000_SavorySweet	18	22	-4	4	2	D
91304250	Topping, milk chocolate with cereal	9000_SavorySweet	18	22	-4	4	0.5	E
53108000	Cake, cupcake, chocolate, NS as to icing	9000_SavorySweet	18	20	-2	3	1.5	E

53115450	Cake or cupcake, peanut butter	9000_SavorySweet	18	20	-2	4	1	E
61113500	Lemon pie filling	9000_SavorySweet	18	19	-1	2	1.5	E
13230120	Pudding, flavors other than chocolate, ready-to-eat, sugar free	9000_SavorySweet	18	18	0	4	3.5	C
53121260	Cake, yellow, with icing, made from home recipe or purchased ready-to-eat	9000_SavorySweet	18	17	1	2	1.5	E
54201010	Crackers, matzo, reduced sodium	9000_SavorySweet	18	17	1	4	4	A
54305000	Crispbread, wheat, no added fat	9000_SavorySweet	18	17	1	4	4	A
54307000	Crackers, matzo	9000_SavorySweet	18	17	1	4	4	A
53100070	Cake batter, raw, not chocolate	9000_SavorySweet	18	16	2	3	2	D
53410860	Cobbler, pineapple	9000_SavorySweet	18	16	2	2	2.5	C
32120100	Egg dessert, Puerto Rican style	9000_SavorySweet	18	15	3	2	1.5	E
53420250	Cream puff, no filling or icing	9000_SavorySweet	18	15	3	4	2	D
13136000	Ice cream sandwich, made with light, no sugar added ice cream	9000_SavorySweet	18	14	4	4	4	A
53305700	Pie, lemon, not cream or meringue	9000_SavorySweet	18	14	4	2	1.5	E
53305720	Pie, lemon, not cream or meringue, individual size or tart	9000_SavorySweet	18	14	4	2	1.5	E
91713040	Fudge, chocolate, with nuts	9000_SavorySweet	17	31	-14	4	1	E
91705500	Mexican chocolate, tablet	9000_SavorySweet	17	29	-12	4	0.5	E
53105300	Cake or cupcake, German chocolate, with icing or filling	9000_SavorySweet	17	26	-9	4	1	E
53205600	Cookie, caramel coated, with nuts	9000_SavorySweet	17	25	-8	4	1	E
54401081	Cheese flavored corn snacks (Cheetos)	9000_SavorySweet	17	25	-8	4	1.5	E
54401112	Tortilla chips, other flavors (Doritos)	9000_SavorySweet	17	25	-8	4	2.5	D
53105750	Cake, chocolate, devil's food, or fudge, pudding type mix, made by "cholesterol free" recipe (water, oil and egg whites added to dry mix), with "light" icing, coating or filling	9000_SavorySweet	17	23	-6	4	1.5	E
54301010	Crackers, butter, plain	9000_SavorySweet	17	23	-6	4	1.5	E
91304300	Topping, chocolate, hard coating	9000_SavorySweet	17	23	-6	4	0.5	E
91104100	Sugar, cinnamon	9000_SavorySweet	17	22	-5	2	1	D
91304090	Topping, chocolate flavored hazelnut spread	9000_SavorySweet	17	22	-5	4	0.5	E
13120140	Ice cream bar, chocolate	9000_SavorySweet	17	21	-4	4	1.5	E
13210750	Pudding, pumpkin	9000_SavorySweet	17	21	-4	4	3	C
53106050	Cake, chocolate, devil's food, or fudge, pudding-type mix (oil, eggs, and water added to dry mix), with icing, coating, or filling	9000_SavorySweet	17	21	-4	4	1.5	E
53300170	Pie, individual size or tart, NFS	9000_SavorySweet	17	21	-4	4	2.5	D
53301070	Pie, apple, individual size or tart	9000_SavorySweet	17	21	-4	4	2.5	D
53302070	Pie, apricot, individual size or tart	9000_SavorySweet	17	21	-4	4	2.5	D
53308070	Pie, pineapple, individual size or tart	9000_SavorySweet	17	21	-4	4	2.5	D
91402000	Jam, preserve, all flavors	9000_SavorySweet	17	21	-4	3	2	D
53105200	Cake, chocolate, devil's food, or fudge, standard-type mix (eggs and water added to dry mix), with icing, coating, or filling	9000_SavorySweet	17	20	-3	3	1.5	E
54205010	Cracker, snack, low sodium	9000_SavorySweet	17	20	-3	4	3	D
54202010	Crackers, saltine, low sodium	9000_SavorySweet	17	19	-2	4	3.5	C

54205100	Cracker, snack, reduced fat, reduced sodium	9000_SavorySweet	17	19	-2	4	3.5	C
54406010	Onion flavored rings	9000_SavorySweet	17	18	-1	4	1.5	E
53121060	Cake, yellow, made from home recipe or purchased ready-to- eat, NS as to icing	9000_SavorySweet	17	17	0	2	1.5	E
54404000	Popcorn chips, plain	9000_SavorySweet	17	17	0	4	3.5	D
54404010	Popcorn chips, other flavors	9000_SavorySweet	17	17	0	4	3.5	D
54404020	Popcorn chips, sweet flavors	9000_SavorySweet	17	17	0	4	3.5	C
53121275	Cake or cupcake, yellow, without icing or filling	9000_SavorySweet	17	15	2	3	1.5	E
13210350	Flan	9000_SavorySweet	17	12	5	2	2.5	D
13210260	Rice flour cream, Puerto Rican style	9000_SavorySweet	17	11	6	1	2.5	D
53270100	Cookies, Puerto Rican style	9000_SavorySweet	17	8	9	2	1	E
11460250	Yogurt, frozen, flavors other than chocolate, with sorbet or sorbet-coated	9000_SavorySweet	16	23	-7	4	3	C
53110000	Cake, fruit cake, light or dark, holiday type cake	9000_SavorySweet	16	22	-6	4	2.5	D
91705410	Chocolate, white, with almonds	9000_SavorySweet	16	22	-6	4	0.5	E
54001000	Crackers, NFS	9000_SavorySweet	16	21	-5	4	1.5	E
54102070	Crackers, Cuca	9000_SavorySweet	16	21	-5	4	1.5	E
54301000	Cracker, snack	9000_SavorySweet	16	21	-5	4	1.5	E
91301060	Maple syrup	9000_SavorySweet	16	21	-5	2	1.5	D
91703600	Espresso coffee beans, chocolate-covered	9000_SavorySweet	16	21	-5	4	0.5	E
11461210	Frozen yogurt bar, vanilla	9000_SavorySweet	16	20	-4	4	3	C
51161100	Roll, sweet, with fruit and nuts, no frosting	9000_SavorySweet	16	20	-4	4	2	E
51161200	Roll, sweet, with nuts, no frosting	9000_SavorySweet	16	19	-3	4	2	D
53510200	Danish pastry, with nuts	9000_SavorySweet	16	19	-3	4	1.5	E
54328105	Crackers, sandwich, peanut butter filled (Ritz)	9000_SavorySweet	16	19	-3	4	1.5	E
54328200	Crackers, sandwich, cheese filled	9000_SavorySweet	16	18	-2	4	1.5	E
63420200	Fruit juice bar, frozen, sweetened with low calorie sweetener, flavors other than orange	9000_SavorySweet	16	18	-2	4	3.5	A
53107000	Cake, cupcake, NS as to type or icing	9000_SavorySweet	16	17	-1	2	1	E
53107200	Cake, cupcake, NS as to type, with icing	9000_SavorySweet	16	17	-1	2	1	E
53260400	Cookie, sugar or plain, sugar free	9000_SavorySweet	16	17	-1	4	2	D
53344000	Pie, custard	9000_SavorySweet	16	17	-1	4	3	C
51185000	Croutons	9000_SavorySweet	16	16	0	4	1.5	E
53113000	Cake, jelly roll	9000_SavorySweet	16	16	0	3	1.5	E
53116490	Cake, pumpkin, NS as to icing	9000_SavorySweet	16	16	0	3	1.5	E
54408485	Pretzels, soft, gluten free	9000_SavorySweet	16	16	0	4	2.5	D
53341500	Pie, buttermilk	9000_SavorySweet	16	13	3	3	1.5	E
53121160	Cake, yellow, without icing, made from home recipe or purchased ready-to-eat	9000_SavorySweet	16	12	4	2	1.5	E
54305500	Crispbread, wheat or rye, extra crispy	9000_SavorySweet	16	10	6	4	4.5	B
53116350	Cake, pound, Puerto Rican style	9000_SavorySweet	16	6	10	2	0.5	E

11830260	Milk, malted, dry mix, not reconstituted	9000_SavorySweet	15	28	-13	4	0.5	E
11830160	Chocolate beverage powder, dry mix, not reconstituted	9000_SavorySweet	15	27	-12	4	1	E
91713020	Fudge, chocolate, chocolate-coated, with nuts	9000_SavorySweet	15	25	-10	4	0.5	E
91718110	Butterfinger Crisp	9000_SavorySweet	15	24	-9	4	0.5	E
54301030	Crackers, butter (Ritz)	9000_SavorySweet	15	23	-8	4	1.5	E
54403160	Popcorn, chocolate coated	9000_SavorySweet	15	22	-7	4	1.5	E
91703050	Caramel with nuts and cereal, chocolate covered	9000_SavorySweet	15	22	-7	4	0.5	E
11460500	Frozen yogurt, soft serve, vanilla	9000_SavorySweet	15	21	-6	4	2.5	D
51161050	Roll, sweet, frosted	9000_SavorySweet	15	20	-5	4	2	E
11459990	Frozen yogurt, NFS	9000_SavorySweet	15	19	-4	4	3	C
11460420	Yogurt, frozen, NS as to flavor, whole milk	9000_SavorySweet	15	19	-4	4	3	C
11461260	Frozen yogurt cone, vanilla	9000_SavorySweet	15	19	-4	4	3	C
53234010	Cookie, peanut butter, with oatmeal	9000_SavorySweet	15	19	-4	4	1.5	E
53235000	Cookie, peanut butter sandwich	9000_SavorySweet	15	19	-4	4	1.5	E
11460000	Frozen yogurt, vanilla	9000_SavorySweet	15	18	-3	4	3	C
11460440	Yogurt, frozen, flavors other than chocolate, whole milk	9000_SavorySweet	15	18	-3	4	3	C
13110130	Ice cream, rich, chocolate	9000_SavorySweet	15	18	-3	4	1.5	D
13110470	Gelato, chocolate	9000_SavorySweet	15	18	-3	4	1.5	D
53234000	Cookie, peanut butter	9000_SavorySweet	15	17	-2	4	1	E
53385000	Pie, pecan	9000_SavorySweet	15	17	-2	4	2	D
53385070	Pie, pecan, individual size or tart	9000_SavorySweet	15	17	-2	4	2	D
11460150	Yogurt, frozen, NS as to flavor, lowfat milk	9000_SavorySweet	15	16	-1	4	3	C
54304000	Crackers, cheese	9000_SavorySweet	15	15	0	4	1.5	E
54304020	Crackers, cheese (Goldfish)	9000_SavorySweet	15	15	0	4	1.5	E
91351020	Topping, dietetic	9000_SavorySweet	15	13	2	4	3.5	B
53216000	Cookie, coconut and nut	9000_SavorySweet	15	12	3	2	0.5	E
53118550	Cake, tres leche	9000_SavorySweet	15	11	4	2	1.5	D
53386000	Pie, pudding, flavors other than chocolate	9000_SavorySweet	15	11	4	3	2.5	C
53107100	Cake, cupcake, NS as to type, without icing	9000_SavorySweet	15	8	7	2	1.5	E
53420410	Sopaipilla with syrup or honey	9000_SavorySweet	15	8	7	2	1.5	E
91703250	TWIX Chocolate Fudge Cookie Bars	9000_SavorySweet	14	23	-9	4	1	E
54420220	Snack mix, plain (Chex Mix)	9000_SavorySweet	14	22	-8	4	2.5	D
13120400	Ice cream bar or stick with fruit	9000_SavorySweet	14	20	-6	4	3	C
53204830	Cookie, brownie, lowfat, with icing	9000_SavorySweet	14	20	-6	4	2	D
11461300	Frozen yogurt cone, vanilla, waffle cone	9000_SavorySweet	14	18	-4	4	3	C
91718300	Ladoo, round ball, Asian-Indian dessert	9000_SavorySweet	14	18	-4	4	2.5	E
91739600	Raisins, yogurt covered	9000_SavorySweet	14	18	-4	4	0.5	E
53233030	Cookie, oatmeal, fat free, with raisins	9000_SavorySweet	14	17	-3	4	2.5	D
53234100	Cookie, peanut butter, with chocolate	9000_SavorySweet	14	17	-3	4	1	E

53521300	Doughnut, wheat, chocolate covered	9000_SavorySweet	14	17	-3	4	1.5	E
51184000	Breadsticks, hard, NFS	9000_SavorySweet	14	16	-2	4	2.5	D
53233040	Cookie, oatmeal, reduced fat, NS as to raisins	9000_SavorySweet	14	16	-2	4	2.5	E
11460170	Yogurt, frozen, flavors other than chocolate, lowfat milk	9000_SavorySweet	14	15	-1	4	3	C
53106500	Cake, cream, without icing or topping	9000_SavorySweet	14	12	2	3	1.5	E
53121300	Cake, yellow, pudding-type mix (oil, eggs, and water added to dry mix), without icing	9000_SavorySweet	14	12	2	3	1.5	E
13252600	Tiramisu	9000_SavorySweet	14	10	4	3	1	E
53120160	Cake, white, without icing, made from home recipe or purchased ready-to-eat	9000_SavorySweet	14	10	4	2	1.5	E
54319500	Rice paper	9000_SavorySweet	14	4	10	3	3.5	C
91708150	Yogurt covered fruit snacks candy, with added vitamin C	9000_SavorySweet	13	24	-11	4	1	E
91731060	M&M's Peanut Butter Chocolate Candies	9000_SavorySweet	13	24	-11	4	0.5	E
91703030	Caramel, with nuts	9000_SavorySweet	13	23	-10	4	1	E
91713050	Fudge, peanut butter	9000_SavorySweet	13	23	-10	4	0.5	E
11830100	Hot chocolate / Cocoa, dry mix, not reconstituted	9000_SavorySweet	13	22	-9	4	1	E
91715100	SNICKERS Bar	9000_SavorySweet	13	21	-8	4	0.5	E
91708160	Yogurt covered fruit snacks candy rolls, with high vitamin C	9000_SavorySweet	13	20	-7	4	2	E
91739010	Raisins, chocolate covered	9000_SavorySweet	13	19	-6	4	1	E
13120120	Ice cream bar or stick, rich chocolate ice cream, thick chocolate covering	9000_SavorySweet	13	18	-5	4	1	E
53452400	Pastry, puff	9000_SavorySweet	13	18	-5	4	2	D
91726000	Nougat, plain	9000_SavorySweet	13	18	-5	4	2	D
13120100	Ice cream bar, vanilla, chocolate coated	9000_SavorySweet	13	17	-4	4	1.5	E
53452420	Pastry, puff, custard or cream filled, iced or not iced	9000_SavorySweet	13	17	-4	4	2	D
11830200	Milk, malted, dry mix, unfortified, not reconstituted, flavors other than chocolate	9000_SavorySweet	13	15	-2	4	1	E
53109000	Cake, cupcake, not chocolate, NS as to icing	9000_SavorySweet	13	15	-2	3	1.5	E
13140115	Light ice cream bar, chocolate	9000_SavorySweet	13	14	-1	4	2	D
53233020	Cookie, oatmeal, with fruit filling	9000_SavorySweet	13	14	-1	4	2	D
54304110	Crackers, cheese, reduced sodium	9000_SavorySweet	13	13	0	4	1.5	E
54440010	Bagel chips	9000_SavorySweet	13	13	0	4	2.5	D
54401121	Tortilla chips, reduced fat, plain	9000_SavorySweet	13	12	1	4	3.5	C
54401122	Tortilla chips, reduced fat, flavored	9000_SavorySweet	13	12	1	4	3.5	C
53341750	Pie, chess	9000_SavorySweet	13	8	5	3	0.5	E
91108000	Sugar substitute, herbal extract sweetener, powder	9000_SavorySweet	13	1	12	2	3.5	B
54401111	Tortilla chips, cool ranch flavor (Doritos)	9000_SavorySweet	12	20	-8	4	3	C
51161150	Roll, sweet, with fruit and nuts, frosted	9000_SavorySweet	12	18	-6	4	1.5	E
91760200	Toffee, chocolate-coated, with nuts	9000_SavorySweet	12	18	-6	4	0.5	E
11461200	Frozen yogurt sandwich	9000_SavorySweet	12	16	-4	4	2.5	D
53120000	Cake, white, standard-type mix (egg whites and water added), NS as to icing	9000_SavorySweet	12	16	-4	3	1	E

53120200	Cake, white, standard-type mix (egg whites and water added to mix), with icing	9000_SavorySweet	12	16	-4	3	1	E
13120792	Ice cream cone, chocolate, prepackaged	9000_SavorySweet	12	15	-3	4	1	E
53121200	Cake, yellow, standard-type mix (eggs and water added to dry mix), with icing	9000_SavorySweet	12	15	-3	3	1.5	E
53301080	Pie, apple, fried pie	9000_SavorySweet	12	15	-3	4	2.5	D
53305080	Pie, cherry, fried pie	9000_SavorySweet	12	15	-3	4	2.5	D
53307080	Pie, peach, fried pie	9000_SavorySweet	12	15	-3	4	2.5	D
54304005	Crackers, cheese (Cheez-It)	9000_SavorySweet	12	15	-3	4	1.5	E
54202020	Crackers, saltine, reduced sodium	9000_SavorySweet	12	14	-2	4	2.5	D
53104550	Cheesecake with fruit	9000_SavorySweet	12	13	-1	4	2.5	D
54408487	Pretzels, soft, gluten free, coated or flavored	9000_SavorySweet	12	12	0	4	2	D
13130320	Light ice cream, no sugar added, NS as to flavor	9000_SavorySweet	12	11	1	4	3	C
13130330	Light ice cream, no sugar added, flavors other than chocolate	9000_SavorySweet	12	11	1	4	3	C
51161270	Pan Dulce, with sugar topping	9000_SavorySweet	12	11	1	4	3	D
53114100	Cake or cupcake, lemon, with icing or filling	9000_SavorySweet	12	10	2	3	1	E
53123500	Cake, shortcake, with whipped topping and fruit, diet	9000_SavorySweet	12	8	4	4	2	D
91200005	Sugar substitute, liquid, NFS	9000_SavorySweet	12	8	4	4	3.5	B
91734400	Reese's Fast Break	9000_SavorySweet	11	25	-14	4	0.5	E
53105270	Cake or cupcake, chocolate, devil's food or fudge, with icing or filling	9000_SavorySweet	11	18	-7	4	1.5	E
91715000	Fudge, caramel and nut, chocolate-coated candy	9000_SavorySweet	11	17	-6	4	0.5	E
53220000	Cookie, fruit-filled bar	9000_SavorySweet	11	16	-5	4	2	E
53220010	Cookie, fruit-filled bar, fat free	9000_SavorySweet	11	16	-5	4	2	E
53220020	Cookie, date bar	9000_SavorySweet	11	16	-5	4	2	E
53220030	Cookie, fig bar	9000_SavorySweet	11	16	-5	4	2	E
53220040	Cookie, fig bar, fat free	9000_SavorySweet	11	16	-5	4	2	E
53251100	Cookie, rugelach	9000_SavorySweet	11	16	-5	4	2	E
91302010	Honey	9000_SavorySweet	11	16	-5	2	1	D
91401000	Jelly, all flavors	9000_SavorySweet	11	16	-5	3	2	D
91705010	Milk chocolate candy, plain	9000_SavorySweet	11	16	-5	4	0.5	E
91705020	Milk chocolate candy, with cereal	9000_SavorySweet	11	16	-5	4	0.5	E
91705420	Chocolate, white, with cereal	9000_SavorySweet	11	16	-5	4	0.5	E
91760500	Truffles	9000_SavorySweet	11	15	-4	4	0.5	E
13120300	Ice cream bar, cake covered	9000_SavorySweet	11	14	-3	4	2	D
13120710	Ice cream cone, chocolate covered, with nuts, flavors other than chocolate	9000_SavorySweet	11	14	-3	4	1	E
53120330	Cake, white, pudding-type mix (oil, egg whites, and water added to dry mix), without icing	9000_SavorySweet	11	14	-3	4	1.5	E
53440000	Strudel, apple	9000_SavorySweet	11	14	-3	4	2.5	D
13130310	Light ice cream, chocolate	9000_SavorySweet	11	13	-2	4	3	C
13142110	Light ice cream cone, chocolate, prepackaged	9000_SavorySweet	11	13	-2	4	3	D

53105100	Cake, chocolate, devil's food, or fudge, standard-type mix (eggs and water added to dry mix), without icing or filling	9000_SavorySweet	11	13	-2	4	1.5	E
53108100	Cake, cupcake, chocolate, without icing or filling	9000_SavorySweet	11	13	-2	4	1.5	E
53306000	Pie, mince, two crust	9000_SavorySweet	11	13	-2	4	2.5	D
53415400	Crisp, cherry	9000_SavorySweet	11	13	-2	3	2	D
13142100	Light ice cream cone, vanilla, prepackaged	9000_SavorySweet	11	12	-1	4	3	C
13220110	Pudding, flavors other than chocolate, made from dry mix	9000_SavorySweet	11	12	-1	3	2.5	C
53108220	Snack cake, chocolate, with icing or filling, reduced fat and calories	9000_SavorySweet	11	12	-1	4	2.5	E
13140100	Light ice cream bar, vanilla, chocolate coated	9000_SavorySweet	11	11	0	4	1.5	D
51161280	Pan Dulce, with raisins and icing	9000_SavorySweet	11	11	0	4	3	D
53120260	Cake, white, with icing, made from home recipe or purchased ready-to-eat	9000_SavorySweet	11	11	0	2	1	E
53260030	Cookie, chocolate chip, sugar free	9000_SavorySweet	11	10	1	4	1.5	E
54304100	Crackers, cheese, reduced fat	9000_SavorySweet	11	10	1	4	1.5	E
51161250	Pan Dulce, no topping	9000_SavorySweet	11	9	2	4	3	D
53109220	Snack cake, not chocolate, with icing or filling, reduced fat and calories	9000_SavorySweet	11	9	2	4	3	D
53116650	Cake, Quezadilla, El Salvadorian style	9000_SavorySweet	11	9	2	4	3	D
91601010	Italian Ice, no sugar added	9000_SavorySweet	11	9	2	4	3.5	A
91611100	Popsicle, no sugar added	9000_SavorySweet	11	9	2	4	3.5	A
91621050	Snow cone, no sugar added	9000_SavorySweet	11	9	2	4	3.5	A
63420105	Frozen fruit juice bar	9000_SavorySweet	10	22	-12	4	3	C
53100100	Cake or cupcake, NS as to type	9000_SavorySweet	10	17	-7	4	1.5	E
53105260	Cake, chocolate, devil's food, or fudge, with icing, coating, or filling, made from home recipe or purchased ready-to-eat	9000_SavorySweet	10	17	-7	4	1.5	E
53112000	Cake, ice cream and cake roll, chocolate	9000_SavorySweet	10	17	-7	4	1.5	E
13121120	Banana split	9000_SavorySweet	10	16	-6	4	2.5	D
53104580	Cheesecake -type dessert, made with yogurt, with fruit	9000_SavorySweet	10	16	-6	4	2.5	C
13120790	Ice cream cone, vanilla, prepackaged	9000_SavorySweet	10	14	-4	4	1	E
53237000	Cookie, raisin	9000_SavorySweet	10	14	-4	4	1	E
91304020	Topping, chocolate	9000_SavorySweet	10	14	-4	4	1.5	E
51160010	Roll, sweet, toasted	9000_SavorySweet	10	12	-2	4	1.5	E
51160100	Roll, sweet, cinnamon bun, no frosting	9000_SavorySweet	10	12	-2	4	2	E
51161000	Pan Dulce, with fruit, no frosting	9000_SavorySweet	10	12	-2	4	2	E
51165000	Coffee cake, yeast type	9000_SavorySweet	10	12	-2	4	2	E
53109300	Cake, Dobos Torte	9000_SavorySweet	10	12	-2	4	1	E
53206050	Cookie, rich, chocolate chip, with chocolate filling	9000_SavorySweet	10	12	-2	4	1	E
53235500	Cookie, with peanut butter filling, chocolate-coated	9000_SavorySweet	10	12	-2	4	0.5	E
53243100	Cookie, rich, all chocolate, with chocolate filling or chocolate chips	9000_SavorySweet	10	12	-2	4	1	E
53241600	Cookie, butter or sugar, with fruit and/or nuts	9000_SavorySweet	10	11	-1	4	1.5	E
54313000	Crackers, oyster	9000_SavorySweet	10	11	-1	4	2	D

54325000	Crackers, saltine	9000_SavorySweet	10	11	-1	4	2	D
54406200	Shrimp chips	9000_SavorySweet	10	11	-1	4	2	E
53120060	Cake, white, made from home recipe or purchased ready-to-eat, NS as to icing	9000_SavorySweet	10	10	0	2	1	E
91511060	Gelatin dessert, dietetic, with sour cream, sweetened with low calorie sweetener	9000_SavorySweet	10	10	0	4	3.5	B
53224000	Cookie, ladyfinger	9000_SavorySweet	10	8	2	4	2	D
53341000	Pie, banana cream	9000_SavorySweet	10	8	2	4	2.5	D
53420000	Cream puff, eclair, custard or cream filled, NS as to icing	9000_SavorySweet	10	8	2	4	1.5	D
54401150	Salty snacks, corn or cornmeal base, tortilla chips, lowfat, baked without fat	9000_SavorySweet	10	6	4	4	3.5	C
91351010	Syrup, dietetic	9000_SavorySweet	10	6	4	4	3.5	B
91713090	Fudge, divinity	9000_SavorySweet	9	22	-13	4	1	E
91718200	Chocolate-flavored sprinkles	9000_SavorySweet	9	17	-8	4	0.5	E
13110110	Ice cream, chocolate	9000_SavorySweet	9	16	-7	4	2	D
13120121	Ice cream bar or stick, rich ice cream, thick chocolate covering	9000_SavorySweet	9	12	-3	4	1	E
13122100	Ice cream pie, no crust	9000_SavorySweet	9	12	-3	4	2	D
53120275	Cake or cupcake, white, without icing or filling	9000_SavorySweet	9	12	-3	4	1.5	E
53226500	Cookie, marshmallow, with rice cereal, no bake	9000_SavorySweet	9	12	-3	4	2	D
53226600	Cookie, marshmallow and peanut butter, with oat cereal, no bake	9000_SavorySweet	9	12	-3	4	2	D
53234250	Cookie, peanut butter with rice cereal, no bake	9000_SavorySweet	9	12	-3	4	2	D
91706100	Coconut candy, no chocolate covering	9000_SavorySweet	9	12	-3	4	0.5	E
53521100	Doughnut, chocolate, raised or yeast, with chocolate icing	9000_SavorySweet	9	11	-2	4	1.5	E
13130100	Light ice cream, NFS	9000_SavorySweet	9	10	-1	4	3	C
13130300	Light ice cream, vanilla	9000_SavorySweet	9	10	-1	4	3	C
53391150	Pie shell, chocolate wafer	9000_SavorySweet	9	10	-1	4	1.5	E
53520160	Doughnut, chocolate, with chocolate icing	9000_SavorySweet	9	10	-1	4	1.5	E
91800100	Chewing gum, NFS	9000_SavorySweet	9	10	-1	4	3	C
53203000	Cookie, applesauce	9000_SavorySweet	9	9	0	4	1.5	E
53206550	Cookie, chocolate, made with oatmeal and coconut, no bake	9000_SavorySweet	9	9	0	4	1.5	E
53233000	Cookie, oatmeal	9000_SavorySweet	9	9	0	4	1.5	E
53233010	Cookie, oatmeal, with raisins	9000_SavorySweet	9	9	0	4	1.5	E
53233100	Cookie, oatmeal, with chocolate and peanut butter, no bake	9000_SavorySweet	9	9	0	4	1.5	E
53236100	Cookie, pumpkin	9000_SavorySweet	9	9	0	4	1.5	E
53304000	Pie, blueberry, two crust	9000_SavorySweet	9	9	0	4	3	C
53391100	Pie shell, graham cracker	9000_SavorySweet	9	8	1	4	1.5	E
54205030	Cracker, cheese, low sodium	9000_SavorySweet	9	8	1	4	1.5	E
54308000	Crackers, milk	9000_SavorySweet	9	8	1	4	2	D
54319000	Crackers, rice	9000_SavorySweet	9	7	2	4	3.5	C
53420100	Cream puff, eclair, custard or cream filled, not iced	9000_SavorySweet	9	6	3	4	1.5	D
91713100	Fudge, brown sugar, penuche	9000_SavorySweet	8	21	-13	4	0.5	E

91735000	Pralines	9000_SavorySweet	8	21	-13	4	0.5	E
91736000	Pineapple candy, Puerto Rican style	9000_SavorySweet	8	21	-13	4	1	D
91703300	TWIX Peanut Butter Cookie Bars	9000_SavorySweet	8	17	-9	4	0.5	E
13121200	Ice cream sundae, prepackaged type, flavors other than chocolate	9000_SavorySweet	8	15	-7	4	2.5	D
13127010	Dippin' Dots, flash frozen ice cream snacks, chocolate	9000_SavorySweet	8	15	-7	4	2	D
53382000	Pie, chocolate-marshmallow	9000_SavorySweet	8	15	-7	4	1	E
54403110	Popcorn, caramel coated	9000_SavorySweet	8	15	-7	4	1	E
91501100	Gelatin salad with vegetables	9000_SavorySweet	8	15	-7	4	3.5	C
91734300	Reese's Sticks	9000_SavorySweet	8	15	-7	4	0.5	E
13110112	Ice cream, chocolate, with additional ingredients	9000_SavorySweet	8	14	-6	4	1.5	D
13120110	Ice cream candy bar	9000_SavorySweet	8	14	-6	4	1	E
13120770	Ice cream cone, scooped, chocolate	9000_SavorySweet	8	14	-6	4	2	D
13120775	Ice cream cone, scooped, chocolate, waffle cone	9000_SavorySweet	8	14	-6	4	2	D
53204600	Cookie, brownie, with peanut butter fudge icing	9000_SavorySweet	8	14	-6	4	1.5	E
13110000	Ice cream, NFS	9000_SavorySweet	8	13	-5	4	2	D
13127000	Dippin' Dots, flash frozen ice cream snacks, flavors other than chocolate	9000_SavorySweet	8	13	-5	4	2	D
13140660	Light ice cream, sundae, soft serve, chocolate or fudge topping, without whipped cream	9000_SavorySweet	8	13	-5	4	2.5	D
13140900	Fudgesicle	9000_SavorySweet	8	13	-5	4	2.5	C
11461000	Yogurt, frozen, chocolate-coated	9000_SavorySweet	8	12	-4	4	1	E
51161020	Roll, sweet, with fruit, frosted	9000_SavorySweet	8	12	-4	4	1.5	E
53520120	Doughnut, chocolate	9000_SavorySweet	8	10	-2	4	1	E
53233060	Cookie, oatmeal, with chocolate chips	9000_SavorySweet	8	9	-1	4	1.5	E
53386250	Pie, pudding, chocolate, with chocolate coating, individual size	9000_SavorySweet	8	9	-1	4	1.5	E
53521120	Doughnut, chocolate, raised or yeast	9000_SavorySweet	8	8	0	4	1.5	E
13252590	Trifle	9000_SavorySweet	8	6	2	4	2.5	D
53103600	Cake, butter, with icing	9000_SavorySweet	8	6	2	3	0.5	E
54336000	Crackers, water	9000_SavorySweet	8	4	4	4	3.5	C
54408475	Pretzels, soft, from school lunch	9000_SavorySweet	8	4	4	4	4	B
63420100	Fruit juice bar, frozen, orange flavor	9000_SavorySweet	7	19	-12	4	3	C
63430110	Sorbet, fruit, citrus flavor	9000_SavorySweet	7	19	-12	4	3	C
11830250	Milk, malted, dry mix, unfortified, not reconstituted, chocolate	9000_SavorySweet	7	15	-8	4	1.5	E
91703150	Toblerone, milk chocolate with honey and almond nougat	9000_SavorySweet	7	15	-8	4	0.5	E
53209050	Cookie, chocolate-covered, chocolate sandwich	9000_SavorySweet	7	14	-7	4	0.5	E
91718100	Butterfinger	9000_SavorySweet	7	14	-7	4	0.5	E
13110100	Ice cream, vanilla	9000_SavorySweet	7	13	-6	4	2	D
13110102	Ice cream, vanilla, with additional ingredients	9000_SavorySweet	7	13	-6	4	2	D
13110200	Ice cream, soft serve, vanilla	9000_SavorySweet	7	13	-6	4	2.5	C
13110220	Ice cream, soft serve, NS as to flavor	9000_SavorySweet	7	13	-6	4	2	D

13120760	Ice cream cone, chocolate covered or dipped, chocolate ice cream	9000_SavorySweet	7	13	-6	4	2	D
13120720	Ice cream cone, chocolate covered or dipped, flavors other than chocolate	9000_SavorySweet	7	12	-5	4	2	D
13120735	Ice cream cone, scooped, vanilla, waffle cone	9000_SavorySweet	7	12	-5	4	2	D
13120782	Ice cream cone, soft serve, vanilla	9000_SavorySweet	7	12	-5	4	3	D
13121000	Ice cream sundae, NFS	9000_SavorySweet	7	12	-5	4	2	D
13121300	Ice cream sundae, hot fudge topping	9000_SavorySweet	7	12	-5	4	2	D
53115200	Cake or cupcake, marble, with icing or filling	9000_SavorySweet	7	12	-5	4	1.5	E
53210900	Cookie, graham cracker with chocolate and marshmallow	9000_SavorySweet	7	12	-5	4	1	E
13140600	Light ice cream, sundae, soft serve, chocolate or fudge topping, with whipped cream	9000_SavorySweet	7	11	-4	4	2	D
53206000	Cookie, chocolate chip	9000_SavorySweet	7	11	-4	4	1	E
53211000	Cookie bar, with chocolate, nuts, and graham crackers	9000_SavorySweet	7	11	-4	4	1	E
53227000	Cookie, marshmallow pies, non-chocolate coating	9000_SavorySweet	7	11	-4	4	1.5	E
53242500	Cookie, toffee bar	9000_SavorySweet	7	11	-4	4	1	E
91404000	Marmalade, all flavors	9000_SavorySweet	7	11	-4	3	1.5	D
91726410	Nougat, chocolate covered	9000_SavorySweet	7	11	-4	4	1.5	E
13122500	Ice cream pie, with cookie crust, fudge topping, and whipped cream	9000_SavorySweet	7	10	-3	4	2	D
53105600	Cake, chocolate, devil's food, or fudge, pudding-type mix, made by "Lite" recipe (eggs and water added to dry mix, no oil added to dry mix), with icing, coating, or filling	9000_SavorySweet	7	10	-3	4	1.5	E
53121280	Cake, yellow, pudding-type mix (oil, eggs, and water added to dry mix), NS as to icing	9000_SavorySweet	7	10	-3	4	1.5	E
53121330	Cake, yellow, pudding-type mix (oil, eggs, and water added to dry mix), with icing	9000_SavorySweet	7	10	-3	4	1.5	E
53201000	Cookie, NFS	9000_SavorySweet	7	10	-3	4	1	E
53205260	Cookie, bar, with chocolate	9000_SavorySweet	7	10	-3	4	1	E
13135000	Light ice cream sandwich, vanilla	9000_SavorySweet	7	9	-2	4	2.5	D
13135010	Light ice cream sandwich, chocolate	9000_SavorySweet	7	9	-2	4	2	D
53610250	Coffee cake, crumb or quick-bread type, custard filled	9000_SavorySweet	7	9	-2	4	2	D
53520150	Doughnut, cake type, chocolate covered, dipped in peanuts	9000_SavorySweet	7	8	-1	4	1	E
51166100	Croissant, cheese	9000_SavorySweet	7	6	1	4	1.5	E
51187020	Anisette toast	9000_SavorySweet	7	6	1	4	3	D
51188500	Zwieback toast	9000_SavorySweet	7	6	1	4	3	D
51161030	Roll, sweet, with fruit, frosted, diet	9000_SavorySweet	7	5	2	4	0.5	E
53365000	Pie, vanilla cream	9000_SavorySweet	7	5	2	4	2.5	D
53511000	Danish pastry, with cheese	9000_SavorySweet	7	5	2	4	2	D
13210290	Pudding, flavors other than chocolate, low calorie, containing artificial sweetener, NS as to from dry mix or ready-to-eat	9000_SavorySweet	7	4	3	4	3	C
51187000	Melba toast	9000_SavorySweet	7	3	4	4	4	C
91713080	Fudge, vanilla, with nuts	9000_SavorySweet	6	20	-14	4	0.5	E
91734200	Reese's Pieces	9000_SavorySweet	6	18	-12	4	0.5	E

53420210	Cream puff, eclair, custard or cream filled, iced, reduced fat	9000_SavorySweet	6	14	-8	4	2	D
13110210	Ice cream, soft serve, chocolate	9000_SavorySweet	6	12	-6	4	2.5	D
91706000	Coconut candy, chocolate covered	9000_SavorySweet	6	12	-6	4	0.5	E
13120730	Ice cream cone, scooped, vanilla	9000_SavorySweet	6	11	-5	4	2.5	D
13120740	Ice cream cone, NFS	9000_SavorySweet	6	11	-5	4	2	D
13140450	Light ice cream, cone, NFS	9000_SavorySweet	6	11	-5	4	3	C
13140500	Light ice cream, cone, flavors other than chocolate	9000_SavorySweet	6	11	-5	4	3	C
13140550	Light ice cream, cone, chocolate	9000_SavorySweet	6	11	-5	4	3	C
53108200	Snack cake, chocolate, with icing or filling	9000_SavorySweet	6	11	-5	4	1.5	E
53208000	Cookie, marshmallow, chocolate-covered	9000_SavorySweet	6	11	-5	4	1.5	E
53208200	Cookie, marshmallow pie, chocolate covered	9000_SavorySweet	6	11	-5	4	1.5	E
53226000	Cookie, marshmallow, with coconut	9000_SavorySweet	6	11	-5	4	1.5	E
13130610	Light ice cream, soft serve, chocolate	9000_SavorySweet	6	10	-4	4	3	C
53120350	Cake, white, pudding-type mix (oil, egg whites, and water added to dry mix), with icing	9000_SavorySweet	6	10	-4	4	1.5	E
53204840	Cookie, brownie, reduced fat, NS as to icing	9000_SavorySweet	6	10	-4	4	2.5	D
53204860	Cookie, brownie, fat free, NS as to icing	9000_SavorySweet	6	10	-4	4	2.5	D
53209010	Cookie, sugar wafer, chocolate-covered	9000_SavorySweet	6	10	-4	4	0.5	E
53209015	Cookie, chocolate sandwich	9000_SavorySweet	6	10	-4	4	1	E
53209100	Cookie, chocolate, sandwich, with extra filling	9000_SavorySweet	6	10	-4	4	1	E
53209500	Cookie, chocolate and vanilla sandwich	9000_SavorySweet	6	10	-4	4	1	E
54102020	Graham crackers, chocolate covered	9000_SavorySweet	6	10	-4	4	0.5	E
13126000	Ice cream, fried	9000_SavorySweet	6	9	-3	4	2	D
53114000	Cake or cupcake, lemon, without icing or filling	9000_SavorySweet	6	8	-2	4	1.5	E
53233050	Cookie, oatmeal sandwich, with creme filling	9000_SavorySweet	6	8	-2	4	1.5	E
53233080	Cookie, oatmeal sandwich, with peanut butter and jelly filling	9000_SavorySweet	6	8	-2	4	1.5	E
53237010	Cookie, raisin sandwich, cream-filled	9000_SavorySweet	6	8	-2	4	1.5	E
53307000	Pie, peach, two crust	9000_SavorySweet	6	8	-2	4	2.5	C
53239100	Pocky	9000_SavorySweet	6	7	-1	4	1.5	E
53386500	Pie, pudding, flavors other than chocolate, with chocolate coating, individual size	9000_SavorySweet	6	7	-1	4	1.5	E
53453150	Empanada, Mexican turnover, fruit-filled	9000_SavorySweet	6	7	-1	4	2.5	D
13140000	Light ice cream bar, vanilla	9000_SavorySweet	6	6	0	4	1.5	D
53247050	Cookie, vanilla wafer, reduced fat	9000_SavorySweet	6	6	0	4	1.5	E
53510000	Danish pastry, plain or spice	9000_SavorySweet	6	6	0	4	1.5	E
53610000	Coffee cake, NFS	9000_SavorySweet	6	6	0	4	1.5	E
53391000	Pie shell	9000_SavorySweet	6	5	1	4	1.5	E
53452500	Pastry, mainly flour and water, fried	9000_SavorySweet	6	5	1	4	1.5	E
51167000	Brioche	9000_SavorySweet	6	3	3	4	1	E
53386050	Pie, pudding, flavors other than chocolate, individual size or tart	9000_SavorySweet	6	3	3	4	2	D

54408030	Pretzels, hard, plain, unsalted	9000_SavorySweet	6	3	3	4	3.5	C
13220230	Pudding, ready-to-eat, chocolate, reduced fat	9000_SavorySweet	5	13	-8	4	3	C
13220235	Pudding, ready-to-eat, chocolate, fat free	9000_SavorySweet	5	13	-8	4	3	C
53204850	Cookie, brownie, fat free, cholesterol free, with icing	9000_SavorySweet	5	13	-8	4	2	E
13120510	Ice cream sandwich, chocolate	9000_SavorySweet	5	11	-6	4	1.5	E
13120786	Ice cream cone, soft serve, vanilla, waffle cone	9000_SavorySweet	5	10	-5	4	3	D
13121100	Ice cream sundae, fruit topping	9000_SavorySweet	5	10	-5	4	2.5	D
13130700	Soft serve, blended with candy or cookies, from fast food	9000_SavorySweet	5	10	-5	4	2.5	D
13161500	Milk dessert sandwich bar, frozen, made from lowfat milk	9000_SavorySweet	5	10	-5	4	3	C
13161520	Milk dessert sandwich bar, frozen, with low-calorie sweetener, made from lowfat milk	9000_SavorySweet	5	10	-5	4	2	D
53209020	Cookie, chocolate sandwich, reduced fat	9000_SavorySweet	5	10	-5	4	1.5	E
91746010	Sugar-coated chocolate discs	9000_SavorySweet	5	10	-5	4	0.5	E
91746150	Easter egg, candy coated chocolate	9000_SavorySweet	5	10	-5	4	0.5	E
53243010	Cookie, vanilla sandwich, extra filling	9000_SavorySweet	5	9	-4	4	1	E
13121500	Ice cream sundae, fudge topping, with cake, with whipped cream	9000_SavorySweet	5	8	-3	4	2	E
51161070	Roll, sweet, with fruit, frosted, fat free	9000_SavorySweet	5	8	-3	4	2	D
13210410	Pudding, rice	9000_SavorySweet	5	5	0	4	3	C
53222020	Cookie, cone shell, ice cream type, wafer or cake	9000_SavorySweet	5	4	1	4	3.5	C
53236000	Cookie, Pizzelle	9000_SavorySweet	5	4	1	4	3.5	C
53305000	Pie, cherry, two crust	9000_SavorySweet	5	4	1	4	2.5	D
53305070	Pie, cherry, individual size or tart	9000_SavorySweet	5	4	1	4	2.5	D
53510100	Danish pastry, with fruit	9000_SavorySweet	5	3	2	4	1.5	E
53520100	Doughnut, cake type, plain	9000_SavorySweet	5	3	2	4	1	E
53500100	Breakfast pastry, NFS	9000_SavorySweet	5	2	3	4	1.5	E
58157110	Spicy rice pudding, Puerto Rican style	9000_SavorySweet	5	2	3	2	2.5	C
91703350	Bar None candy bar	9000_SavorySweet	4	13	-9	4	1	E
53206500	Cookie, chocolate, made with rice cereal	9000_SavorySweet	4	11	-7	4	0.5	E
53121270	Cake or cupcake, yellow, with icing or filling	9000_SavorySweet	4	10	-6	4	1.5	E
13120500	Ice cream sandwich, vanilla	9000_SavorySweet	4	9	-5	4	1.5	D
13120784	Ice cream cone, soft serve, chocolate	9000_SavorySweet	4	9	-5	4	2.5	D
53207000	Cookie, chocolate or fudge	9000_SavorySweet	4	9	-5	4	1	E
53210000	Cookie, chocolate wafer	9000_SavorySweet	4	9	-5	4	1	E
91304030	Topping, fruit	9000_SavorySweet	4	9	-5	4	2.5	D
91705090	Chocolate candy with fondant and caramel	9000_SavorySweet	4	9	-5	4	0.5	E
13120788	Ice cream cone, soft serve, chocolate, waffle cone	9000_SavorySweet	4	8	-4	4	2.5	D
53109270	Cake, cupcake, chocolate, with or without icing, fruit filling or cream filling, lowfat, cholesterol free	9000_SavorySweet	4	8	-4	4	2	D
53204500	Cookie, brownie, with cream cheese filling, without icing	9000_SavorySweet	4	8	-4	4	1	E
13140630	Light ice cream, sundae, soft serve, fruit topping, with whipped cream	9000_SavorySweet	4	7	-3	4	2.5	D

53104570	Cheesecake, diet, with fruit	9000_SavorySweet	4	7	-3	4	2.5	D
53301750	Pie, apple, diet	9000_SavorySweet	4	7	-3	4	3	D
53381000	Pie, lemon meringue	9000_SavorySweet	4	7	-3	4	2.5	D
53381070	Pie, lemon meringue, individual size or tart	9000_SavorySweet	4	7	-3	4	2.5	D
91102010	Sugar, brown	9000_SavorySweet	4	7	-3	2	0.5	E
91104200	Sugar, raw	9000_SavorySweet	4	7	-3	2	0.5	E
13120550	Ice cream cookie sandwich	9000_SavorySweet	4	6	-2	4	1.5	D
53101250	Cake, angel food, with fruit and icing or filling	9000_SavorySweet	4	6	-2	4	2.5	D
53206030	Cookie, chocolate chip, reduced fat	9000_SavorySweet	4	6	-2	4	1.5	E
91301200	Sugar, brown, and water syrup	9000_SavorySweet	4	6	-2	2	1	D
53520110	Doughnut, cake type	9000_SavorySweet	4	3	1	4	1	E
53520200	Churros	9000_SavorySweet	4	3	1	4	1.5	E
53520600	Cruller, NFS	9000_SavorySweet	4	3	1	4	1	E
53521220	Doughnut, chocolate cream-filled	9000_SavorySweet	4	3	1	4	1.5	E
53520500	Doughnut, Asian	9000_SavorySweet	4	2	2	4	1	E
53103550	Cake, butter, without icing	9000_SavorySweet	4	1	3	3	1	E
53204800	Cookie, brownie, diet, NS as to icing	9000_SavorySweet	4	1	3	4	3	C
13130590	Light ice cream, soft serve, NS as to flavor	9000_SavorySweet	3	9	-6	4	3	C
13130600	Light ice cream, soft serve, flavors other than chocolate	9000_SavorySweet	3	9	-6	4	3	C
13130620	Light ice cream, soft serve cone, flavors other than chocolate	9000_SavorySweet	3	8	-5	4	3	C
13130640	Light ice cream, soft serve cone, NS as to flavor	9000_SavorySweet	3	8	-5	4	3	C
13140670	Light ice cream, sundae, soft serve, fruit topping, without whipped cream	9000_SavorySweet	3	8	-5	4	3	C
53112100	Ice cream cake	9000_SavorySweet	3	8	-5	4	2	D
13170000	Baked Alaska	9000_SavorySweet	3	7	-4	4	2	D
53209000	Cookie, chocolate, chocolate sandwich or chocolate-coated or striped	9000_SavorySweet	3	7	-4	4	1	E
53247500	Cookie, vanilla with caramel, coconut, and chocolate coating	9000_SavorySweet	3	6	-3	4	0.5	E
53104520	Cheesecake, diet	9000_SavorySweet	3	5	-2	4	2.5	D
53111000	Cake or cupcake, gingerbread	9000_SavorySweet	3	5	-2	4	1.5	E
53207020	Cookie, chocolate or fudge, reduced fat	9000_SavorySweet	3	5	-2	4	1.5	E
53239050	Cookie, shortbread, with icing or filling	9000_SavorySweet	3	5	-2	4	1	E
53104600	Cheesecake, chocolate	9000_SavorySweet	3	4	-1	4	1	E
53206010	Cookie, chocolate chip, with raisins	9000_SavorySweet	3	4	-1	4	1	E
53240000	Cookie, animal	9000_SavorySweet	3	4	-1	4	2	D
91109000	Blue Agave liquid sweetener, sugar substitute	9000_SavorySweet	3	4	-1	2	1.5	D
91302020	Agave liquid sweetener	9000_SavorySweet	3	4	-1	2	1.5	D
32401000	Meringues	9000_SavorySweet	3	3	0	2	1	E
51166200	Croissant, chocolate	9000_SavorySweet	3	3	0	4	1	E
53300100	Pie, NFS	9000_SavorySweet	3	3	0	4	2.5	C

53301000	Pie, apple, two crust	9000_SavorySweet	3	3	0	4	2.5	C
53521130	Doughnut, yeast type, with chocolate icing	9000_SavorySweet	3	3	0	4	1	E
53521110	Doughnut, yeast type	9000_SavorySweet	3	2	1	4	1.5	E
51166500	Croissant, fruit	9000_SavorySweet	3	1	2	4	1.5	E
54408480	Pretzels, soft, multigrain	9000_SavorySweet	3	1	2	4	3.5	C
91501060	Gelatin dessert with sour cream	9000_SavorySweet	3	1	2	3	3	C
91511030	Gelatin dessert, dietetic, with whipped topping, sweetened with low calorie sweetener	9000_SavorySweet	3	1	2	4	3.5	A
91713010	Fudge, chocolate, chocolate-coated	9000_SavorySweet	2	10	-8	4	0.5	E
91746100	M&M's Milk Chocolate Candies	9000_SavorySweet	2	10	-8	4	0.5	E
91746120	Sixlets	9000_SavorySweet	2	10	-8	4	0.5	E
53204000	Cookie, brownie, NS as to icing	9000_SavorySweet	2	6	-4	4	1.5	E
53204010	Cookie, brownie, without icing	9000_SavorySweet	2	6	-4	4	1.5	E
53238000	Cookie, sandwich-type, not chocolate or vanilla	9000_SavorySweet	2	6	-4	4	1.5	E
53242000	Cookie, sugar wafer	9000_SavorySweet	2	6	-4	4	0.5	E
53243000	Cookie, vanilla sandwich	9000_SavorySweet	2	6	-4	4	1.5	E
53247000	Cookie, vanilla wafer	9000_SavorySweet	2	6	-4	4	1.5	E
54102200	Graham crackers, sandwich, with filling	9000_SavorySweet	2	6	-4	4	1.5	E
13140700	Creamsicle	9000_SavorySweet	2	5	-3	4	2.5	C
53205250	Cookie, butterscotch, brownie	9000_SavorySweet	2	5	-3	4	1.5	E
53245000	Cookie, vanilla waffle creme	9000_SavorySweet	2	5	-3	4	0.5	E
53109100	Cake, cupcake, not chocolate, without icing or filling	9000_SavorySweet	2	4	-2	4	1.5	D
53121100	Cake, yellow, standard-type mix (eggs and water added to dry mix), without icing	9000_SavorySweet	2	4	-2	4	1.5	D
53237500	Cookie, rum ball, no bake	9000_SavorySweet	2	4	-2	4	1	E
13110460	Gelato, vanilla	9000_SavorySweet	2	3	-1	4	1.5	E
53202000	Cookie, almond	9000_SavorySweet	2	3	-1	4	1	E
53235600	Cookie, Pfeffernusse	9000_SavorySweet	2	3	-1	4	1	E
53239000	Cookie, shortbread	9000_SavorySweet	2	3	-1	4	1	E
53520135	Doughnut, cake type, with icing	9000_SavorySweet	2	3	-1	4	1	E
53223000	Cookie, gingersnaps	9000_SavorySweet	2	2	0	4	2	D
53230000	Cookie, molasses	9000_SavorySweet	2	2	0	4	1.5	D
53231000	Cookie, Lebkuchen	9000_SavorySweet	2	2	0	4	1.5	D
53520000	Doughnut, NFS	9000_SavorySweet	2	2	0	4	1	E
53520130	Doughnut, cake type, powdered sugar	9000_SavorySweet	2	2	0	4	1	E
53520140	Doughnut, cake type, chocolate icing	9000_SavorySweet	2	2	0	4	1	E
53520170	Doughnut holes	9000_SavorySweet	2	2	0	4	1	E
53521230	Doughnut, custard-filled, with icing	9000_SavorySweet	2	2	0	4	1.5	E
54102060	Crackers, Cuban	9000_SavorySweet	2	2	0	4	1.5	D
51188100	Pannetone	9000_SavorySweet	2	1	1	4	2	D

53521210	Doughnut, custard-filled	9000_SavorySweet	2	1	1	4	1.5	E
54408432	Pretzels, soft, ready-to-eat, topped with cheese	9000_SavorySweet	2	1	1	4	3.5	C
54408470	Pretzels, soft, filled with cheese	9000_SavorySweet	2	1	1	4	3.5	C
91301090	Sorghum syrup	9000_SavorySweet	1	14	-13	4	1	D
91715200	Baby Ruth	9000_SavorySweet	1	13	-12	4	0.5	E
91707010	Fondant, chocolate covered	9000_SavorySweet	1	9	-8	4	1	E
13150000	Sherbet, all flavors	9000_SavorySweet	1	8	-7	4	3	C
13160410	Fat free ice cream, chocolate	9000_SavorySweet	1	8	-7	4	2.5	D
54403150	Popcorn, sugar syrup or caramel-coated, fat free	9000_SavorySweet	1	8	-7	4	1	E
63430150	Sorbet	9000_SavorySweet	1	8	-7	4	2.5	C
63430500	Fruit juice bar with cream, frozen	9000_SavorySweet	1	8	-7	4	3	C
91305010	Icing, chocolate	9000_SavorySweet	1	8	-7	4	0.5	E
91706400	Coconut candy, Puerto Rican style	9000_SavorySweet	1	8	-7	4	0.5	E
91705030	Kit Kat	9000_SavorySweet	1	7	-6	4	0.5	E
91705430	Kit Kat White	9000_SavorySweet	1	7	-6	4	0.5	E
91713030	Fudge, chocolate	9000_SavorySweet	1	7	-6	4	0.5	E
91801000	Chewing gum, regular	9000_SavorySweet	1	7	-6	4	1.5	E
51165100	Coffee cake, yeast type, fat free, cholesterol free, with fruit	9000_SavorySweet	1	6	-5	4	3	D
53102800	Cake or cupcake, Black Forest	9000_SavorySweet	1	6	-5	4	1.5	E
53120100	Cake, white, standard-type mix (egg whites and water added to mix), without icing	9000_SavorySweet	1	6	-5	4	1.5	E
53204100	Cookie, brownie, with icing or filling	9000_SavorySweet	1	6	-5	4	1	E
91703400	Whatchamacallit	9000_SavorySweet	1	6	-5	4	0.5	E
13121400	Ice cream sundae, caramel topping	9000_SavorySweet	1	5	-4	4	2	D
13230130	Pudding, chocolate, ready-to-eat	9000_SavorySweet	1	5	-4	4	3	C
53215500	Cookie, coconut	9000_SavorySweet	1	5	-4	4	0.5	E
53225000	Cookie, macaroon	9000_SavorySweet	1	5	-4	4	0.5	E
91306030	Chocolate dip	9000_SavorySweet	1	5	-4	4	1	E
91703040	Caramel candy, chocolate covered	9000_SavorySweet	1	5	-4	4	0.5	E
13200110	Pudding, chocolate, NFS	9000_SavorySweet	1	4	-3	4	3	C
13210220	Pudding, chocolate, NS as to from dry mix or ready-to-eat	9000_SavorySweet	1	4	-3	4	3	C
13210520	Pudding, tapioca, made from dry mix	9000_SavorySweet	1	4	-3	4	3	C
13230500	Pudding, tapioca, ready-to-eat	9000_SavorySweet	1	4	-3	4	3	C
53103000	Cake, Boston cream pie	9000_SavorySweet	1	4	-3	4	2	D
53120270	Cake or cupcake, white, with icing or filling	9000_SavorySweet	1	4	-3	4	1.5	E
53226550	Cookie, marshmallow, with rice cereal and chocolate chips	9000_SavorySweet	1	4	-3	4	0.5	E
53342000	Pie, chocolate cream	9000_SavorySweet	1	4	-3	4	1	E
91611050	Ice pop filled with ice cream, all flavor varieties	9000_SavorySweet	1	4	-3	4	3	C
53116270	Cake, pound, chocolate	9000_SavorySweet	1	3	-2	4	1	E

91723010	Marshmallow, chocolate covered	9000_SavorySweet	1	3	-2	4	1	E
91726140	MILKY WAY MIDNIGHT Bar	9000_SavorySweet	1	3	-2	4	0.5	E
91726425	3 Musketeers Truffle Crisp Bar	9000_SavorySweet	1	3	-2	4	0.5	E
13110120	Ice cream, rich, flavors other than chocolate	9000_SavorySweet	1	2	-1	4	1.5	E
13110140	Ice cream, rich, NS as to flavor	9000_SavorySweet	1	2	-1	4	1.5	E
13230200	Pudding, ready-to-eat, chocolate and non-chocolate flavors combined	9000_SavorySweet	1	2	-1	4	3	C
53104400	Cake or cupcake, coconut, with icing or filling	9000_SavorySweet	1	2	-1	4	1	E
53241510	Marie biscuit	9000_SavorySweet	1	2	-1	4	1.5	E
53261000	Cookie, gluten free	9000_SavorySweet	1	2	-1	4	0.5	E
53530000	Breakfast tart	9000_SavorySweet	1	2	-1	4	1.5	D
91306040	Dessert dip	9000_SavorySweet	1	2	-1	4	1.5	E
91705400	Chocolate, white	9000_SavorySweet	1	2	-1	4	0.5	E
13120050	Ice cream bar, vanilla	9000_SavorySweet	1	1	0	4	1	E
13120310	Ice cream bar, stick or nugget, with crunch coating	9000_SavorySweet	1	1	0	4	1	E
13140650	Light ice cream, sundae, soft serve, not fruit or chocolate topping, with whipped cream	9000_SavorySweet	1	1	0	4	2	D
13140680	Light ice cream, sundae, soft serve, not fruit or chocolate topping, without whipped cream	9000_SavorySweet	1	1	0	4	2.5	D
13210280	Pudding, flavors other than chocolate, NFS	9000_SavorySweet	1	1	0	4	2.5	C
13220240	Pudding, ready-to-eat, flavors other than chocolate, reduced fat	9000_SavorySweet	1	1	0	4	3	C
13220245	Pudding, ready-to-eat, flavors other than chocolate, fat free	9000_SavorySweet	1	1	0	4	3	C
13230110	Pudding, flavors other than chocolate, ready-to-eat	9000_SavorySweet	1	1	0	4	2.5	C
13230510	Pudding, ready-to-eat, tapioca, fat free	9000_SavorySweet	1	1	0	4	3	C
13252200	Milk dessert or milk candy, Puerto Rican style	9000_SavorySweet	1	1	0	4	1.5	E
51160000	Roll, sweet, no frosting	9000_SavorySweet	1	1	0	4	3	D
51160110	Roll, sweet, cinnamon bun, frosted	9000_SavorySweet	1	1	0	4	0.5	E
51166000	Croissant	9000_SavorySweet	1	1	0	4	1.5	E
53101100	Cake, angel food, without icing or filling	9000_SavorySweet	1	1	0	4	2	D
53101200	Cake, angel food, with icing or filling	9000_SavorySweet	1	1	0	3	1	E
53101300	Cake, angel food, chocolate, without icing	9000_SavorySweet	1	1	0	4	2	D
53104500	Cheesecake	9000_SavorySweet	1	1	0	4	1	E
53109200	Snack cake, not chocolate, with icing or filling	9000_SavorySweet	1	1	0	4	1	E
53109210	Cake, cupcake, not chocolate, with icing or filling, lowfat, cholesterol free	9000_SavorySweet	1	1	0	4	2	E
53109250	Cake, cupcake, not chocolate, with fruit and cream filling	9000_SavorySweet	1	1	0	4	1.5	E
53114200	Cake, lemon, lowfat, without icing	9000_SavorySweet	1	1	0	4	1.5	E
53114250	Cake, lemon, lowfat, with icing	9000_SavorySweet	1	1	0	4	1.5	E
53116000	Cake, pound, without icing or filling	9000_SavorySweet	1	1	0	4	1.5	E
53116020	Cake, pound, with icing or filling	9000_SavorySweet	1	1	0	4	1	E
53116380	Cake, pound, fat free, cholesterol free	9000_SavorySweet	1	1	0	4	2	D

53116390	Cake, pound, reduced fat, cholesterol free	9000_SavorySweet	1	1	0	4	2	D
53118100	Cake, sponge, without icing or filling	9000_SavorySweet	1	1	0	4	1.5	E
53118200	Cake, sponge, with icing or filling	9000_SavorySweet	1	1	0	4	1	E
53118300	Cake, sponge, chocolate	9000_SavorySweet	1	1	0	4	1	E
53118410	Rum cake, without icing	9000_SavorySweet	1	1	0	4	1.5	E
53123070	Cake, shortcake, sponge type, with whipped cream and fruit	9000_SavorySweet	1	1	0	4	2	D
53123080	Cake, shortcake, sponge type, with fruit	9000_SavorySweet	1	1	0	4	2	D
53206100	Cookie, chocolate chip sandwich	9000_SavorySweet	1	1	0	4	1	E
53207050	Cookie, chocolate, with chocolate filling or coating, fat free	9000_SavorySweet	1	1	0	4	1.5	E
53209005	Cookie, chocolate, with icing or coating	9000_SavorySweet	1	1	0	4	0.5	E
53222010	Cookie, fortune	9000_SavorySweet	1	1	0	4	2	D
53222100	Cookie, cone shell, ice cream type, brown sugar	9000_SavorySweet	1	1	0	4	2	D
53228000	Cookie, meringue	9000_SavorySweet	1	1	0	2	0.5	E
53239010	Cookie, shortbread, reduced fat	9000_SavorySweet	1	1	0	4	1.5	E
53240010	Cookie, animal, with frosting or icing	9000_SavorySweet	1	1	0	4	0.5	E
53241500	Cookie, butter or sugar	9000_SavorySweet	1	1	0	4	1	E
53243050	Cookie, vanilla sandwich, reduced fat	9000_SavorySweet	1	1	0	4	1.5	E
53244010	Cookie, butter or sugar, with chocolate icing or filling	9000_SavorySweet	1	1	0	4	0.5	E
53244020	Cookie, butter or sugar, with icing or filling other than chocolate	9000_SavorySweet	1	1	0	4	1	E
53246000	Cookie, tea, Japanese	9000_SavorySweet	1	1	0	4	2	D
53260150	Cookie, lemon wafer, lowfat	9000_SavorySweet	1	1	0	4	2	D
53343000	Pie, coconut cream	9000_SavorySweet	1	1	0	4	2	D
53420200	Cream puff, eclair, custard or cream filled, iced	9000_SavorySweet	1	1	0	4	1.5	E
53511500	Danish pastry, with cheese, fat free, cholesterol free	9000_SavorySweet	1	1	0	4	2	D
53520510	Beignet	9000_SavorySweet	1	1	0	4	1	E
53520700	French cruller	9000_SavorySweet	1	1	0	4	1.5	E
53521140	Doughnut, jelly	9000_SavorySweet	1	1	0	4	1.5	E
53530010	Breakfast tart, lowfat	9000_SavorySweet	1	1	0	4	3	C
53610120	Coffee cake, crumb or quick-bread type, reduced fat, cholesterol free	9000_SavorySweet	1	1	0	4	1.5	E
54101010	Cracker, animal	9000_SavorySweet	1	1	0	4	2	D
54202050	Crackers, saltine, fat free, low sodium	9000_SavorySweet	1	1	0	4	3	D
54301200	Cracker, snack, fat free	9000_SavorySweet	1	1	0	4	2.5	D
54325010	Crackers, saltine, reduced fat	9000_SavorySweet	1	1	0	4	2.5	D
54408000	Pretzels, NFS	9000_SavorySweet	1	1	0	4	2	D
54408010	Pretzels, hard	9000_SavorySweet	1	1	0	4	2	D
54408015	Pretzels, hard, NFS	9000_SavorySweet	1	1	0	4	2	D
54408016	Pretzels, hard, plain, salted	9000_SavorySweet	1	1	0	4	2	D
54408017	Pretzels, hard, plain, lightly salted	9000_SavorySweet	1	1	0	4	3	D

54408020	Pretzels, soft	9000_SavorySweet	1	1	0	4	3	C
54408035	Pretzels, hard, flavored	9000_SavorySweet	1	1	0	4	2	E
54408040	Pretzels, soft, unsalted	9000_SavorySweet	1	1	0	4	3.5	C
54408070	Pretzels, hard, multigrain	9000_SavorySweet	1	1	0	4	2	D
54408080	Pretzel, gluten free	9000_SavorySweet	1	1	0	4	1	E
54408081	Pretzels, hard, plain, gluten free	9000_SavorySweet	1	1	0	4	1	E
54408082	Pretzels, hard, flavored, gluten free	9000_SavorySweet	1	1	0	4	1	E
54408105	Pretzel chips, hard, plain	9000_SavorySweet	1	1	0	4	1	E
54408110	Pretzel chips, hard, flavored	9000_SavorySweet	1	1	0	4	0.5	E
54408190	Pretzels, hard, coated, NFS	9000_SavorySweet	1	1	0	4	1	E
54408200	Pretzels, hard, chocolate coated	9000_SavorySweet	1	1	0	4	1	E
54408210	Pretzels, hard, white chocolate coated	9000_SavorySweet	1	1	0	4	0.5	E
54408250	Pretzels, hard, yogurt coated	9000_SavorySweet	1	1	0	4	0.5	E
54408290	Pretzels, hard, filled, NFS	9000_SavorySweet	1	1	0	4	0.5	E
54408300	Pretzels, hard, cheese filled	9000_SavorySweet	1	1	0	4	0.5	E
54408400	Pretzels, soft, NFS	9000_SavorySweet	1	1	0	4	2	D
54408405	Pretzels, soft, ready-to-eat, NFS	9000_SavorySweet	1	1	0	4	2	D
54408410	Pretzels, soft, ready-to-eat, salted, buttered	9000_SavorySweet	1	1	0	4	2	D
54408411	Pretzels, soft, ready-to-eat, unsalted, buttered	9000_SavorySweet	1	1	0	4	3.5	C
54408415	Pretzels, soft, ready-to-eat, salted, no butter	9000_SavorySweet	1	1	0	4	3	D
54408416	Pretzels, soft, ready-to-eat, unsalted, no butter	9000_SavorySweet	1	1	0	4	3.5	C
54408420	Pretzels, soft, ready-to-eat, cinnamon sugar coated	9000_SavorySweet	1	1	0	4	2.5	D
54408422	Pretzels, soft, ready-to-eat, coated or flavored	9000_SavorySweet	1	1	0	4	2	D
54408430	Pretzels, soft, ready-to-eat, topped with meat	9000_SavorySweet	1	1	0	4	3	D
54408456	Pretzels, soft, from frozen, unsalted	9000_SavorySweet	1	1	0	4	3.5	C
91101000	Sugar, NFS	9000_SavorySweet	1	1	0	2	0.5	E
91101010	Sugar, white, granulated or lump	9000_SavorySweet	1	1	0	2	0.5	E
91101020	Sugar, white, confectioner's, powdered	9000_SavorySweet	1	1	0	2	0.5	E
91105010	Fructose sweetener, sugar substitute, dry powder	9000_SavorySweet	1	1	0	4	0.5	E
91106000	Sugar substitute, sugar-aspartame blend, dry powder	9000_SavorySweet	1	1	0	4	0.5	E
91106010	Sugar substitute and sugar blend	9000_SavorySweet	1	1	0	4	0.5	E
91107000	Sugar substitute, sucralose, powder	9000_SavorySweet	1	1	0	4	1	E
91200000	Sugar substitute, powder, NFS	9000_SavorySweet	1	1	0	4	0.5	E
91200020	Sugar substitute, saccharin-based, dry powder	9000_SavorySweet	1	1	0	4	0.5	E
91200030	Brown sugar substitute, saccharin-based, dry powder	9000_SavorySweet	1	1	0	4	0.5	E
91200040	Sugar substitute, saccharin, powder	9000_SavorySweet	1	1	0	4	0.5	E
91200110	Sugar substitute, saccharin, liquid	9000_SavorySweet	1	1	0	4	3.5	A
91201010	Sugar substitute, aspartame, powder	9000_SavorySweet	1	1	0	4	1	E
91300010	Syrup, NFS	9000_SavorySweet	1	1	0	4	2	D

91300100	Pancake syrup	9000_SavorySweet	1	1	0	4	2	D
91301020	Cane and corn pancake syrup	9000_SavorySweet	1	1	0	4	2.5	C
91301030	Corn syrup	9000_SavorySweet	1	1	0	4	1	D
91301040	Buttered blends syrup	9000_SavorySweet	1	1	0	4	1	E
91301050	Blueberry syrup	9000_SavorySweet	1	1	0	4	1.5	E
91301080	Chocolate syrup	9000_SavorySweet	1	1	0	4	1.5	E
91301081	Chocolate syrup, light	9000_SavorySweet	1	1	0	4	2.5	C
91301100	Simple syrup	9000_SavorySweet	1	1	0	2	2	D
91301130	Strawberry drink syrup	9000_SavorySweet	1	1	0	4	1.5	D
91301250	Maple and corn and/or cane pancake syrup blends	9000_SavorySweet	1	1	0	4	2	D
91301510	Pancake syrup, light	9000_SavorySweet	1	1	0	4	2.5	D
91304010	Topping, butterscotch or caramel	9000_SavorySweet	1	1	0	4	1.5	E
91304040	Topping, marshmallow	9000_SavorySweet	1	1	0	4	2	D
91304050	Hard sauce	9000_SavorySweet	1	1	0	4	0.5	E
91305020	Icing, white	9000_SavorySweet	1	1	0	4	1	E
91306020	Caramel dip, regular	9000_SavorySweet	1	1	0	4	0.5	E
91306025	Caramel dip, light	9000_SavorySweet	1	1	0	4	1.5	E
91361040	Dessert sauce	9000_SavorySweet	1	1	0	2	2.5	D
91501010	Gelatin dessert	9000_SavorySweet	1	1	0	4	3	C
91501015	Gelatin snacks	9000_SavorySweet	1	1	0	4	2.5	D
91501020	Gelatin dessert with fruit	9000_SavorySweet	1	1	0	4	3.5	C
91501030	Gelatin dessert with whipped cream	9000_SavorySweet	1	1	0	4	3	C
91501050	Gelatin dessert with cream cheese	9000_SavorySweet	1	1	0	4	3	C
91511010	Gelatin dessert, sugar free	9000_SavorySweet	1	1	0	4	3.5	A
91601000	Italian Ice	9000_SavorySweet	1	1	0	4	2.5	C
91610900	Popsicle, NFS	9000_SavorySweet	1	1	0	4	3	C
91611000	Popsicle	9000_SavorySweet	1	1	0	4	3	C
91612000	Freezer pop	9000_SavorySweet	1	1	0	4	3	C
91621000	Snow cone	9000_SavorySweet	1	1	0	4	2.5	C
91700010	Candy, NFS	9000_SavorySweet	1	1	0	4	1.5	E
91702010	Butterscotch morsels	9000_SavorySweet	1	1	0	4	0.5	E
91703010	Caramel, chocolate-flavored roll	9000_SavorySweet	1	1	0	4	1.5	E
91703020	Caramel, flavor other than chocolate	9000_SavorySweet	1	1	0	4	1	E
91703070	Rolo	9000_SavorySweet	1	1	0	4	0.5	E
91703200	TWIX Caramel Cookie Bars	9000_SavorySweet	1	1	0	4	0.5	E
91707000	Fondant	9000_SavorySweet	1	1	0	4	0.5	E
91708000	Fruit peel, candied	9000_SavorySweet	1	1	0	4	1	E
91708030	Fruit leather and fruit snacks candy	9000_SavorySweet	1	1	0	4	1.5	E
91708040	Fun Fruits Creme Supremes	9000_SavorySweet	1	1	0	4	0.5	E

91708070	Tamarind candy	9000_SavorySweet	1	1	0	4	0.5	E
91708100	Fruit snacks candy, with high vitamin C	9000_SavorySweet	1	1	0	4	1	E
91709000	Gumdrops, chocolate covered	9000_SavorySweet	1	1	0	4	1	E
91713070	Fudge, vanilla	9000_SavorySweet	1	1	0	4	0.5	E
91715300	100 GRAND Bar	9000_SavorySweet	1	1	0	4	0.5	E
91721000	Licorice	9000_SavorySweet	1	1	0	4	1	E
91723000	Marshmallow	9000_SavorySweet	1	1	0	4	1.5	D
91723020	Marshmallow, candy-coated	9000_SavorySweet	1	1	0	4	1	E
91723050	Marshmallow, coconut-coated	9000_SavorySweet	1	1	0	4	1	E
91726110	Nougat, with caramel, chocolate covered	9000_SavorySweet	1	1	0	4	0.5	E
91726130	MILKY WAY Bar	9000_SavorySweet	1	1	0	4	0.5	E
91726420	3 MUSKETEERS Bar	9000_SavorySweet	1	1	0	4	0.5	E
91745010	Gumdrops	9000_SavorySweet	1	1	0	4	1.5	E
91745020	Hard candy	9000_SavorySweet	1	1	0	4	1.5	E
91745040	Butterscotch hard candy	9000_SavorySweet	1	1	0	4	0.5	E
91745100	Skittles	9000_SavorySweet	1	1	0	4	0.5	E
91746200	M&M's Pretzel Chocolate Candies	9000_SavorySweet	1	1	0	4	0.5	E
91750000	Taffy	9000_SavorySweet	1	1	0	4	0.5	E
91760000	Toffee, plain	9000_SavorySweet	1	1	0	4	1	E
91760100	Toffee, chocolate covered	9000_SavorySweet	1	1	0	4	0.5	E
91760700	Wax candy, liquid filled	9000_SavorySweet	1	1	0	4	2.5	C
91770000	Dietetic or low calorie candy, NFS	9000_SavorySweet	1	1	0	4	3	C
91770010	Dietetic or low calorie gumdrops	9000_SavorySweet	1	1	0	4	1	E
91770020	Dietetic or low calorie hard candy	9000_SavorySweet	1	1	0	4	3	C
91770050	Dietetic or low calorie mints	9000_SavorySweet	1	1	0	4	3	C

[a]non-integer NOVA scores for mixed dishes have been rounded to the nearest whole number

HSR Health Star Rating; FCS Food Compass Score; NS Not specified; NFS Not further specified
